# Proceedings of the 23^rd^ Paediatric Rheumatology European Society Congress: part two: Genoa, Italy. 28 September – 01 October 2016

**DOI:** 10.1186/s12969-017-0142-8

**Published:** 2017-05-30

**Authors:** Olga Lomakina, Ekaterina Alekseeva, Sania Valieva, Tatiana Bzarova, Irina Nikishina, Elena Zholobova, Svetlana Rodionovskaya, Maria Kaleda, Yasuo Nakagishi, Masaki Shimizu, Mao Mizuta, Akihiro Yachie, Yuko Sugita, Nami Okamoto, Kousuke Shabana, Takuji Murata, Hiroshi Tamai, Eve M. Smith, Peng Yin, Andrea L. Jorgensen, Michael W. Beresford, on behalf of On behalf of the UK JSLE Cohort Study, Eve M. Smith, Antonio Eleuteri, Beatrice Goilav, Laura Lewandowski, Angel Phuti, Dawn Wahezi, Tamar Rubinstein, Caroline Jones, Paul Newland, Stephen Marks, Rachel Corkhill, Diana Ekdawy, Clarissa Pilkington, Kjell Tullus, Chaim Putterman, Chris Scott, Antony C. Fisher, Michael W. Beresford, Eve M. Smith, Laura Lewandowski, Angel Phuti, Andrea Jorgensen, Chris Scott, Michael W. Beresford, Ezgi Deniz Batu, Can Kosukcu, Ekim Taskiran, Sema Akman, Kubra Ozturk, Betul Sozeri, Erbil Unsal, Zelal Ekinci, Yelda Bilginer, Mehmet Alikasifoglu, Seza Ozen, Hanna Lythgoe, Michael W. Beresford, Hermine I. Brunner, Gaurav Gulati, Jordan T. Jones, Mekibib Altaye, Jamie Eaton, Mark Difrancesco, Joo Guan Yeo, Jingyao Leong, Loshinidevi D/O Thana Bathi, Thaschawee Arkachaisri, Salvatore Albani, Nagla Abdelrahman, Michael W Beresford, Valentina Leone, UK JSLE study group supported by the National Institute of Health Research Clinical Research Network, Noortje Groot, D. Shaikhani, I. E. M. Bultink, M. Bijl, R. J. E. M. Dolhain, Y. K. O. Teng, E. Zirkzee, K. de Leeuw, R. Fritsch-Stork, S. S. M. Kamphuis, Rachael D. Wright, Eve M. Smith, Michael W. Beresford, Reem Abdawani, Laila Al Shaqshi, Ibrahim Al Zakwani, Natali W. Gormezano, David Kern, Oriany L. Pereira, Gladys C. C. Esteves, Adriana M. Sallum, Nadia E. Aikawa, Rosa M. Pereira, Clovis A. Silva, Eloisa Bonfa, Jessica Beckmann, Nora Bartholomä, Nils Venhoff, Philipp Henneke, Ulrich Salzer, Ales Janda, Alina Lucica Boteanu, Sandra Garrote Corral, Alberto Sifuentes Giraldo, Mariluz Gámir Gámir, Antonio Zea Mendoza, Amra Adrovic, Reyhan Dedeoglu, Sezgin Sahin, Kenan Barut, Aida Koka, Funda Oztunc, Ozgur Kasapcopur, Ana Luisa Rodriguez-Lozano, Francisco Rivas-Larrauri, Silvestre García de la Puente, Andressa G. F. Alves, Maria F. D. A. Giacomin, Juliana Farhat, Alfésio L. F. Braga, Adriana M. E. Sallum, Lúcia M. D. A. Campos, Luiz A. A. Pereira, Ana J. D. F. C. Lichtenfels, Clóvis A. Silva, Sylvia C. L. Farhat, Banu Acar, Z. Birsin Ozcakar, Nilgün Çakar, Nermin Uncu, Gökçe Gür, Semanur Özdel, Fatoş Yalçınkaya, Christiaan Scott, Nicky Brice, Peter Nourse, Laura Lewandowski, Christine Arango, Angela C. Mosquera, Clara Malagon, Ana P. Sakamoto, Clovis A. Silva, Marco F. C. D. Silva, Ananadreia S. Lopes, Gleice C. S. Russo, Adriana E. M. Sallum, Katia Kozu, Eloisa Bonfá, Claudia Saad-Magalhães, Rosa M. R. Pereira, Claudio A. Len, Maria T. Terreri, Deepti Suri, Siyaram Didel, Amit Rawat, Surjit Singh, Despoina Maritsi, MArgarita Onoufriou, Olga Vougiouka, Maria Tsolia, Edi Paleka Bosak, Mandica Vidović, Mirta Lamot, Lovro Lamot, Miroslav Harjaček, Erika Van Nieuwenhove, Adrian Liston, Carine Wouters, Fatemeh Tahghighi, Vahid Ziaee, Seid-Reza Raeeskarami, Francisca Aguiar, Sandra Pereira, Mariana Rodrigues, Cláudia Moura, Gustavo Rocha, Hercília Guimarães, Iva Brito, Francisca Aguiar, Rita Fonseca, Mariana Rodrigues, Iva Brito, Gerd Horneff, Ariane Klein, Kirsten Minden, Hans-Iko Huppertz, Frank Weller-Heinemann, Jasmin Kuemmerle-Deschner, J-Peter Haas, Anton Hospach, BIKER collaborative group, Ricardo Menendez-Castro, Boris Huegle, Johannes-Peter Haas, Joost Swart, Gabriella Giancane, Francesca Bovis, Elio Castagnola, Andreas Groll, Gerd Horneff, Hans-Iko Huppertz, Daniel J. Lovell, Tom Wolfs, Michael Hofer, Ekaterina Alekseeva, Violeta Panaviene, Susan Nielsen, Jordi Anton, Florence Uettwiller, Valda Stanevicha, Maria Trachana, Denise Pires Marafon, Constantin Ailioaie, Elena Tsitsami, Sylvia Kamphuis, Troels Herlin, Pavla Doležalová, Gordana Susic, Berit Flatø, Flavio Sztajnbok, Angela Pistorio, Alberto Martini, Nico Wulffraat, Nicolino Ruperto, Marco Gattorno, Antonio Brucato, Martina Finetti, George Lazaros, Silvia Maestroni, Mara Carraro, Davide Cumetti, Alessandra Carobbio, Monia Lorini, Alessandro Rimini, Renzo Marcolongo, Anna Valenti, Gian Luca Erre, Riccardo Belli, Fiorenzo Gaita, Maria Pia Sormani, Nicolino Ruperto, Massimo Imazio, Alberto Martini, Mario Abinun, Nicola Smith, Tim Rapley, Flora McErlane, Lianne Kearsley-Fleet, Kimme L. Hyrich, Helen Foster, Nicolino Ruperto, Daniel J. Lovell, Nikolay Tzaribachev, Andrew Zeft, Rolando Cimaz, Valda Stanevicha, Gerd Horneff, John Bohnsack, Thomas Griffin, Ruy Carrasco, Maria Trachana, Jason Dare, Ivan Foeldvari, Richard Vehe, Francesca Bovis, Teresa Simon, Alberto Martini, Hermine Brunner, S. Verazza, S. Davì, A. Consolaro, A. Insalaco, V. Gerloni, R. Cimaz, F. Zulian, S. Pastore, F. Corona, G. Conti, P. Barone, M. Cattalini, E. Cortis, L. Breda, A. N. Olivieri, A. Civino, R. Podda, D. Rigante, F. La Torre, G. D’Angelo, M. Jorini, R. Gallizzi, M. C. Maggio, R. Consolini, A. De Fanti, M. G. Alpigiani, A. Martini, A. Ravelli, on behalf of Italian Pediatric Rheumatology Study Group, Betul Sozeri, Aysenur Pac Kısaarslan, Zubeyde Gunduz, Ruhan Dusunsel, Ismail Dursun, Hakan Poyrazoglu, Ekaterina Kuchinskaya, Farida Abduragimova, Mikhail Kostik, Erik Sundberg, Soley Omarsdottir, Lena Klevenvall, Helena Erlandsson-Harris, Gokalp Basbozkurt, Ozge Erdemli, Dogan Simsek, Fatih Yazici, Yildirim Karsioglu, Aysen Tezcaner, Dilek Keskin, Huseyin Ozkan, Cengizhan Acikel, Seza Ozen, Erkan Demirkaya, Ilonka Orbán, Krisztina Sevcic, Valentin Brodszky, Emese Kiss, Ismaiel A. Tekko, Madeleine Rooney, James McElnay, Cliff Taggart, Helen McCarthy, Ryan F. Donnelly, Drug Delivery Group, Mario Abinun, Mary Slatter, Zohreh Nademi, Mark Friswell, Helen Foster, Sharmila Jandial, Flora McErlane, Terence Flood, Sophie Hambleton, Andrew Gennery, Andrew Cant, Martina Finetti, Francesca Bovis, Joost Swart, Pavla Doležalová, Elena Tsitsami, Maria Trachana, Erkan Demirkaya, Phoi-Ngoc Duong, Isabelle Koné-Paut, Olga Vougiouka, Denise Pires Marafon, Rolando Cimaz, Giovanni Filocamo, María Luz Gamir, Valda Stanevicha, Helga Sanner, Laura Carenini, Nico Wulffraat, Alberto Martini, Nicolino Ruperto, Mesut Topdemir, Gokalp Basbozkurt, Yildirim Karslioglu, Huseyin Ozkan, Cengizhan Acikel, Erkan Demirkaya, Faysal Gok, Elena Zholobova, Nadezhda Tsurikova, Elena Ligostaeva, Navdha R. Ramchurn, Mark Friswell, O. Kostareva, I. Nikishina, S. Arsenyeva, S. Rodionovskaya, M. Kaleda, D. Alexeev, Ismail Dursun Dursun, Betul Sozeri, Aysenur Pac Kısaarslan, Ruhan Dusunsel, Hakan Poyrazoglu, Hakan Poyrazoglu, Sara Murias, Estefania Barral, Rosa Alcobendas, Eugenia Enriquez, Agustin Remesal, Jaime de Inocencio, Tania M. Castro, Simone A. Lotufo, Tatjana Freye, Raffaella Carlomagno, Thomas Zumbrunn, Jan Bonhoeffer, Elvira Cannizzaro Schneider, Daniela Kaiser, Michaël Hofer, Véronique Hentgen, Andreas Woerner, Juvenile Inflammatory Rheumatism (JIR) Cohort, Tobias Schwarz, Jens Klotsche, Martina Niewerth, Gerd Horneff, Johannes-Peter Haas, Anton Hospach, Hans-Iko Huppertz, Gerd Ganser, Kirsten Minden, ICON study group, Jerold Jeyaratnam, Nienke ter Haar, Ozgur Kasapcopur, Donato Rigante, Fatma Dedeoglu, Ezgi Baris, Sebastiaan Vastert, Nico Wulffraat, Joost Frenkel, Jonathan S. Hausmann, Kathleen G. Lomax, Ari Shapiro, Karen L. Durrant, P. A. Brogan, M. Hofer, J. B. Kuemmerle-Deschner, B. Lauwerys, A. Speziale, K. Leon, X. Wei, R. M. Laxer, Sara Signa, Marta Rusmini, Elena Campione, Sabrina Chiesa, Alice Grossi, Alessia Omenetti, Roberta Caorsi, Gianmaria Viglizzo, Alberto Martini, Isabella Ceccherini, Marco Gattorno, Silvia Federici, Joost Frenkel, Seza Ozen, Helen Lachmann, Martina Finetti, Alberto Martini, Nicola Ruperto, Marco Gattorno, on behalf of PRINTO and Eurofever Registry, Silvia Federici, Federica Vanoni, Seza Ozen, Michaël Hofer, Joost Frenkel, Helen Lachmann, Alberto Martini, Nicola Ruperto, Marco Gattorno, on behalf of PRINTO and Eurofever Project, Sonia Melo Gomes, Ebun Omoyinmi, Juan I. Arostegui, Eva Gonzalez-Roca, Despina Eleftheriou, Nigel Klein, Paul Brogan, Stefano Volpi, Elettra Santori, Paolo Picco, Claudia Pastorino, Roberta Caorsi, Gillian Rice, Alessandra Tesser, Alberto Martini, Yanick Crow, Fabio Candotti, Marco Gattorno, Kenan Barut, Sezgin Sahin, Amra Adrovic, Ada B. Sinoplu, Gozde Yucel, Gizem Pamuk, Ozgur Kasapcopur, Laura O. Damian, Cecilia Lazea, Mihaela Sparchez, Paulina Vele, Laura Muntean, Adriana Albu, Simona Rednic, Calin Lazar, Leonardo O. Mendonça, Alessandra Pontillo, Jorge Kalil, Fabio M. Castro, Myrthes T. Barros, Manuela Pardeo, Virginia Messia, Fabrizio De Benedetti, Antonella Insalaco, Giorgia Malighetti, Chiara Gorio, Francesca Ricci, Ilaria Parissenti, Paola Montesano, Barbara Bonafini, Veronica Medeghini, Marco Cattalini, Lucio Giordano, Giulia Zani, Rosalba Ferraro, Donatella Vairo, Silvia Giliani, Marco Cattalini, Maria Cristina Maggio, Girolamo Luppino, Giovanni Corsello, Maria Isabel Gonzalez Fernandez, Berta Lopez Montesinos, Adriana Rodriguez Vidal, Juan I. Arostegui Gorospe, Inmaculada Calvo Penades, Nadia K. Rafiq, Karen Wynne, Khalid Hussain, Paul A. Brogan, Elizabeth Ang, Nicholas Ng, Ayla Kacar, Ozge Altug Gucenmez, Balahan Makay, Sevket Erbil Unsal, Yasin Sahin, Kenan Barut, Tufan Kutlu, Fugen Cullu-Cokugras, Sezgin Sahin, Amra Adrovic, Hasret Ayyildiz-Civan, Ozgur Kasapcopur, Tulay Erkan, Reem Abdawani, Sana Al Zuhbi, Eiman Abdalla, Ricardo A. Russo, María M. Katsicas, Roberta Caorsi, Francesca Minoia, Gianmaria Viglizzo, Alice Grossi, Sabrina Chiesa, Paolo Picco, Angelo Ravelli, Marco Gattorno, Sagar Bhattad, Amit Rawat, Anju Gupta, Deepti Suri, Vignesh Pandiarajan, Ritambhra Nada, Kaara Tiewsoh, Philip Hawkins, Dorota Rowczenio, Surjit Singh, Sarka Fingerhutova, Jana Franova, Leona Prochazkova, Eva Hlavackova, Pavla Dolezalova, Havva Evrengül, Selçuk Yüksel, Mustafa Doğan, Dolunay Gürses, Harun Evrengül, Silvia De Pauli, Serena Pastore, Anna Monica Bianco, Giovanni Maria Severini, Andrea Taddio, Alberto Tommasini, Svetlana O. Salugina, Evgeny Fedorov, Elena Kamenets, Ekaterina Zaharova, Maria Kaleda, Svetlana O. Salugina, Evgeny Fedorov, Elena Kamenets, Ekaterina Zaharova, Maria Kaleda, Tatiana Sleptsova, Ekaterina Alexeeva, Kirill Savostyanov, Alexander Pushkov, Tatyana Bzarova, Saniya Valieva, Rina Denisova, Kseniya Isayeva, Evgeniya Chistyakova, Olga Lomakina, Margarita Soloshenko, Elena Kaschenko, Utako Kaneko, Chihaya Imai, Akihiko Saitoh, Vitor A. Teixeira, Filipa O. Ramos, Manuela Costa, Yonatan Butbul Aviel, Shafe Fahoum, Riva Brik, Zeynep Birsin Özçakar, Nilgün Çakar, Nermin Uncu, Banu Acar Celikel, Fatos Yalcinkaya, Benedetta Schiappapietra, Sergio Davi’, Federica Mongini, Luisa Giannone, Cecilia Bava, Maria Giannina Alpigiani, Alberto Martini, Angelo Ravelli, Alessandro Consolaro, Dragana S. Lazarevic, Jelena Vojinovic, Gordana Susic, Jelena Basic, Gabriella Giancane, Valentina Muratore, Valentina Marzetti, Neus Quilis, Belen Serrano Benavente, Alessandra Alongi, Adele Civino, Lorenzo Quartulli, Alessandro Consolaro, Alberto Martini, Angelo Ravelli, Giedre Januskeviciute, Pieter van Dijkhuizen, Valentina Muratore, Gabriella Giancane, Benedetta Schiappapietra, Alberto Martini, Angelo Ravelli, Alessandro Consolaro, N. Groot, W. van Dijk, I. E. M. Bultink, M. Bijl, R. J. E. M. Dolhain, Y. K. O. Teng, E. Zirkzee, K. de Leeuw, R. Fritsch-Stork, S. S. M. Kamphuis, Noortje Groot, A. Kardolus, I. E. M. Bultink, M. Bijl, R. J. E. M. Dolhain, Y. K. O. Teng, E. Zirkzee, K. de Leeuw, R. Fritsch-Stork, S. S. M. Kamphuis, Raul Gutiérrez Suárez, Ellen B. Nordal, Veronika G. Rypdal, Lillemor Berntson, Maria Ekelund, Kristiina Aalto, Suvi Peltoniemi, Marek Zak, Susan Nielsen, Mia Glerup, Troels Herlin, Ellen D. Arnstad, Anders Fasth, Marite Rygg, the Nordic Study Group of Pediatric Rheumatology (NoSPeR), Ana Catarina Duarte, Sandra Sousa, Lídia Teixeira, Ana Cordeiro, Mª José Santos, Ana Filipa Mourão, Maria José Santos, Mónica Eusébio, Ana Lopes, Filipa Oliveira-Ramos, Manuel Salgado, Paula Estanqueiro, José Melo-Gomes, Fernando Martins, José Costa, Carolina Furtado, Ricardo Figueira, Iva Brito, Jaime C. Branco, João E. Fonseca, Helena Canhão, Ana F. Mourão, Maria Jose Santos, Mónica Eusébio, Ana Lopes, Filipa Oliveira-Ramos, Manuel Salgado, Paula Estanqueiro, José Melo-Gomes, Fernando Martins, José Costa, Carolina Furtado, Ricardo Figueira, Iva Brito, Jaime C. Branco, João E. Fonseca, Helena Canhão, Andrea Coda, Samuel Cassidy, Kerry West, Gordon Hendry, Debra Grech, Julie Jones, Fiona Hawke, Davinder Singh Grewal, Andrea Coda, Julie Jones, Debra Grech, Davinder Singh Grewal, Charlene Foley, Orla Killeen, Emma MacDermott, Douglas Veale, Ursula Fearon, Dilek Konukbay, Erkan Demirkaya, Ela Tarakci, Nilay Arman, Kenan Barut, Sezgin Şahin, Amra Adrovic, Ozgur Kasapcopur, Jane Munro, Alessandro Consolaro, Esi Morgan, Meredith Riebschleger, Jennifer Horonjeff, Vibeke Strand, Clifton Bingham, Ma. Theresa M. Collante, Margarita Ganeva, Stefan Stefanov, Albena Telcharova, Dimitrina Mihaylova, Radoslava Saraeva, Reni Tzveova, Radka Kaneva, Adelina Tsakova, Katya Temelkova, GRANT Medical University, Sofia 68/2015, Maria Mercedes C. Picarelli, Luiz C. Danzmann, Florencia Barbé-Tuana, Lucas K. Grun, Marcus H. Jones, Marijan Frković, Karla Ištuk, Ika Birkić, Saša Sršen, Marija Jelušić, Nicola Smith, Sharmila Jandial, Alan Easton, Rachael Quarmby, Raju Khubchandani, Mercedes Chan, Tim Rapley, Helen Foster, Radoslav Srp, Katerina Kobrova, Jana Franova, Sarka Fingerhutova, Dana Nemcova, Jozef Hoza, Michal Uher, Melania Saifridova, Lenka Linkova, Pavla Dolezalova, Sirirat Charuvanij, Isree Leelayuwattanakul, Thita Pacharapakornpong, Sakda A.-O. Vallipakorn, Butsabong Lerkvaleekul, Soamarat Vilaiyuk, Valentina Muratore, Gabriella Giancane, Stefano Lanni, Alessandra Alongi, Maria Giannina Alpigiani, Alberto Martini, Angelo Ravelli, Alessandro Consolaro, Alessandra Alongi, Francesca Bovis, Francesca Minoia, Sergio Davì, Alberto Martini, Nicolino Ruperto, Randy Q. Cron, Angelo Ravelli, Chiara Passarelli, Manuela Pardeo, Elisa Pisaneschi, Antonio Novelli, Fabrizio De Benedetti, Claudia Bracaglia, Claudia Bracaglia, Denise Pires Marafon, Ivan Caiello, Kathy de Graaf, Florence Guilhot, Walter Ferlin, Sergio Davi’, Grant Schulert, Angelo Ravelli, Alexi A. Grom, Robert Nelson, Cristina de Min, Fabrizio De Benedetti, Dirk Holzinger, Christoph Kessel, Ndate Fall, Alexei Grom, Wilco de Jager, Sebastiaan Vastert, Raffaele Strippoli, Claudia Bracaglia, Erik Sundberg, Anna Horne, Stephan Ehl, Sandra Ammann, Kai Lehmberg, Fabrizio De Benedetti, Karin Beutel, Dirk Foell, Francesca Minoia, AnnaCarin Horne, Francesca Bovis, Sergio Davì, Laura Pagani, Graciela Espada, Yi-jin Gao, Antonella Insalaco, Kai Lehmberg, Helga Sanner, Susan Shenoi, Sheila Weitzman, Nicolino Ruperto, Alberto Martini, Randy Q. Cron, Angelo Ravelli, Giusi Prencipe, Ivan Caiello, Antonia Pascarella, Claudia Bracaglia, Walter G. Ferlin, Laurence Chatel, Raffaele Strippoli, Cristina de Min, Fabrizio De Benedetti, Philippe Jacqmin, Kathy De Graaf, Maria Ballabio, Robert Nelson, Zoë Johnson, Walter Ferlin, Geneviève Lapeyre, Fabrizio de Benedetti, de Min Cristina, Hiroyuki Wakiguchi, Shunji Hasegawa, Reiji Hirano, Fumiko Okazaki, Tamaki Nakamura, Hidenobu Kaneyasu, Shouichi Ohga, Kazuko Yamazaki, Tomo Nozawa, Taichi Kanetaka, Shuichi Ito, Shumpei Yokota, Kirsty McLellan, Ishbel MacGregor, Neil Martin, Joyce Davidson, Jasmin Kuemmerle-Deschner, Sandra Hansmann, Nico Wulffraat, Andreas Eikelberg, Iris Haug, Sabrina Schuller, Susanne M. Benseler, Single Hub and Access point for paediatric Rheumatology in Europe (SHARE), Liliia S. Nazarova, Kseniia V. Danilko, Viktor A. Malievsky, Tatiana V. Viktorova, Angela Mauro, Ebun Omoyinmi, Angela Barnicoat, Paul Brogan, Charlene Foley, Orla Killeen, Emma MacDermott, Douglas Veale, Charlene Foley, Orla Killeen, Emma MacDermott, Douglas Veale, Sonia Melo Gomes, Ebun Omoyinmi, Jane Hurst, Nathalie Canham, Despina Eleftheriou, Nigel Klein, Sandrine Lacassagne, Paul Brogan, Anastasia Wiener, Boris Hügle, Bernd Denecke, Ivan Costa-Filho, Johannes Peter Haas, Klaus Tenbrock, David Popp, Arjan Boltjes, Frank Rühle, Stefanie Herresthal, Wilco de Jager, Femke van Wijk, Joachim Schultze, Monika Stoll, Luisa Klotz, Thomas Vogl, Johannes Roth, Estefania Quesada-Masachs, Daniel Álvarez de la Sierra, Marina Garcia Prat, Ana M. Marín Sánchez, Ricardo Pujol Borrell, Sara Marsal Barril, Mónica Martínez Gallo, Consuelo Modesto Caballero, Iryna Chyzheuskaya, Lyudmyla M. Byelyaeva, Rostislav M. Filonovich, Helena K. Khrustaleva, Larisa I. Zajtseva, Tamara M. Yuraga, Iryna Chyzheuskaya, Lyudmyla M. Byelyaeva, Rostislav M. Filonovich, Helena K. Khrustaleva, Larisa I. Zajtseva, Tamara M. Yuraga, Thomas Giner, Lukas Hackl, Julia Albrecht, Reinhard Würzner, Juergen Brunner, Serena Pastore, Marta Minute, Fulvio Parentin, Alessandra Tesser, Agostino Nocerino, Andrea Taddio, Alberto Tommasini, Mette Nørgaard, Troels Herlin, Mikel Alberdi-Saugstrup, Marek S. Zak, Susan M. Nielsen, Troels Herlin, Ellen Nordal, Lillemor Berntson, Anders Fasth, Marite Rygg, Klaus G. Müller, Nordic Study Group of Pediatric Rheumatology (NoSPeR), Mojca Zajc Avramovič, Vita Dolžan, Nataša Toplak, Tadej Avčin, N. Ruperto, D. J. Lovell, C. Wallace, M. Toth, I. Foeldvari, J. Bohnsack, D. Milojevic, C. Rabinovich, D. Kingsbury, K. Marzan, P. Quartier, K. Minden, E. Chalom, G. Horneff, R. M. Kuester, J. Dare, M. Heinrich, H. Kupper, J. Kalabic, A. Martini, H. I. Brunner, on behalf of PRINTO and PRCSG, Alessandro Consolaro, Gerd Horneff, Ruben Burgos-Vargas, Tamas Constantin, Ivan Foeldvari, Jelena Vojinovic, Joke Dehoorne, Violeta Panaviene, Gordana Susic, Valda Stanevica, Katarzyna Kobusinska, Zbigniew Zuber, Richard Mouy, Ingrida Rumba-Rozenfelde, Pavla Dolezalova, Chantal Job-Deslandre, Nico Wulffraat, Ronald Pederson, Jack Bukowski, Tina Hinnershitz, Bonnie Vlahos, Alberto Martini, Nicolino Ruperto, Paula Keskitalo, Salla Kangas, Paula Vähäsalo, Raul A. Chavez Valencia, David Martino, Jane Munro, Anne-Louise Ponsonby, Rachel Chiaroni-Clarke, Braydon Meyer, Roger C. Allen, Jonathan D. Akikusa, Jeffrey M. Craig, Richard Saffrey, Justine A. Ellis, Sergio Davì, Francesca Minoia, AnnaCarin Horne, Nico Wulffraat, Carine Wouters, Carol Wallace, Yosef Uziel, Gary Sterba, Rayfel Schneider, Ricardo Russo, Athimalaipet V. Ramanan, Jana Pachlopnik Schmid, Seza Ozen, Kim E Nichols, Paivi Miettunen, Daniel J. Lovell, Kai Lehmberg, Toshiyuki Kitoh, Raju Khubchandani, Norman T. Ilowite, Jan-Inge Henter, Alexei A Grom, Fabrizio De Benedetti, Edward M. Behrens, Tadej Avcin, Maurizio Aricò, Alberto Martini, Nicolino Ruperto, Randy Q. Cron, Angelo Ravelli, Sriharsha Grevich, Peggy Lee, Sarah Ringold, Brian Leroux, Hannah Leahey, Megan Yuasa, Jessica Foster, Jeremy Sokolove, Lauren Lahey, William Robinson, Joshua Newson, Anne Stevens, Stephanie J. W. Shoop, Kimme L. Hyrich, Suzanne M. M. Verstappen, Wendy Thomson, Janet E. McDonagh, CAPS, Timothy Beukelman, Yuki Kimura, Marc Natter, Norm Ilowite, Kelly Mieszkalski, Grendel Burrell, Brian Best, Helen Bristow, Shannon Carr, Anne Dennos, Rachel Kaufmann, Laura Schanberg, for the CARRA Registry Investigators, Ilaria Parissenti, Antonella Insalaco, Andrea Taddio, Angela Mauro, Manuela Pardeo, Francesca Ricci, Gabriele Simonini, Marco Cattalini, Paola Montesano, Ilaria Parissenti, Francesca Ricci, Barbara Bonafini, Veronica Medeghini, Francesca Lancini, Marco Cattalini, Margaux Gerbaux, Phu-Quoc Lê, Laurence Goffin, Valérie Badot, Céline La, Laure Caspers, François Willermain, Alina Ferster, Maria Ceci, Francesco Licciardi, Marco Turco, Francesca Santarelli, Davide Montin, Claudia Toppino, Maria Cristina Maggio, Clotilde Alizzi, Bruno Papia, Beatrice Vergara, Umberto Corpora, Luca Messina, Giovanni Corsello, Maria Tsinti, Vasiliko Dermentzoglou, Panagiotis Tziavas, Elena Tsitsami, Marija Perica, Mandica Vidović, Lovro Lamot, Miroslav Harjaček, Lana Tambić Bukovac, Mustafa Çakan, Nuray Aktay Ayaz, Gonca Keskindemirci, Paivi Miettunen, Michael Lang, Catherine Laing, Susanne Benseler, Tommy Gerschman, Nadia Luca, Heinrike Schmeling, Anastasia Dropol, Jaymi Taiani, Nicole Johnson, Brian Rusted, Panagiota Nalbanti, Maria Trachana, Polyxeni Pratsidou, Grigoris Pardalos, Vasiliki Tzimouli, Anna Taparkou, Maria Stavrakidou, Fotios Papachristou, Florence Kanakoudi-Tsakalidou, Peter Bale, Emily Robinson, Jason Palman, Clarissa Pilkington, Elizabeth Ralph, Kimberly Gilmour, Clare Heard, Lucy R. Wedderburn, Raffaella Carlomagno, Yara Barrense-Dias, Antonarakis Gregory, Dhouib Amira, Scolozzi Paolo, Hanquinet Sylviane, Hofer Michaël, Nataliya Panko, Salah Shokry, Liudmila Rakovska, Sally Pino, Adriana Diaz-Maldonado, Pilar Guarnizo, Sofia Torreggiani, Paolo Cressoni, Umberto Garagiola, Giancarla Di Landro, Giampietro Farronato, Fabrizia Corona, Giovanni Filocamo, Susan Shenoi, Samantha Bell, Parveen Bhatti, Lee Nelson, Beth A. Mueller, T. A. Simon, A. Baheti, N. Ray, Z. Guo, Nicolino Ruperto, Hermine I. Brunner, Anasuya Hazra, Thomas Stock, Ronnie Wang, Charles Mebus, Christine Alvey, Manisha Lamba, Sriram Krishnaswami, Umberto Conte, Min Wang, Nikolay Tzaribachev, Ivan Foeldvari, Gerd Horneff, Daniel Kingsbury, Elena Koskova, Elzbieta Smolewska, Richard K. Vehe, Zbigniew Zuber, Alberto Martini, Daniel Lovell, Tomohiro Kubota, Masaki Shimizu, Junko Yasumura, Yasuo Nakagishi, Toshitaka Kizawa, Masato Yashiro, Hiroyuki Wakiguchi, Tsuyoshi Yamatou, Yuichi Yamasaki, Syuji Takei, Yoshifumi Kawano, Ulrika Järpemo Nykvist, Bo Magnusson, Rikard Wicksell, Karin Palmblad, Gunnar L. Olsson, Vahid Ziaee, Mohammadreza Modaressi, Mohammad-Hassan Moradinejad, Valentina Seraya, Elena Zholobova, Alisa Vitebskaya, Veronica Moshe, Gil Amarilyo, Liora Harel, Phillip J Hashkes, Amir Mendelson, Noa Rabinowicz, Yonit Reis, Yosef Uziel, Zane Dāvidsone, Arina Lazareva, Ruta Šantere, Dace Bērziņa, Valda Staņēviča, Giulia Camilla Varnier, Alessandro Consolaro, Clarissa Pilkington, Susan Maillard, Cristina Ferrari, Silvia Zaffarano, Alberto Martini, Angelo Ravelli, Juvenile Dermatomyositis Research Group and European Federation of Immunological Societies, Judith Wienke, Felicitas Bellutti Enders, Lucas L. van den Hoogen, Jorre S. Mertens, Timothy R. Radstake, Henny G. Hotten, Ruth Fritsch, Wilco de Jager, Lucy Wedderburn, Kiran Nistala, Clarissa Pilkington, Berent Prakken, Annet van Royen-Kerkhof, Femke van Wijk, Mohammad Alhemairi, Mohammed Muzaffer, Pieter Van Dijkhuizen, Claire T. Deakin, Stefania Simou, Lucy R. Wedderburn, Maria De Iorio, Qiong Wu, Tania Amin, Stefania Simou, Lee Dossetter, Lucy R. Wedderburn, Clarissa Pilkington, Juvenile Dermatomyositis Research Group (JDRG), Raquel Campanilho-Marques, Claire Deakin, Stefania Simou, Lucy R. Wedderburn, Clarissa A. Pilkington, on behalf of Juvenile Dermatomyositis Research Group (JDRG), Silvia Rosina, Alessandro Consolaro, Pieter van Dijkhuizen, Kiran Nistala, Nicola Ruperto, Clarissa Pilkington, Angelo Ravelli, Sirisucha Soponkanaporn, Stefania Simou, Claire T. Deakin, Lucy R. Wedderburn, on behalf of the UK Juvenile Dermatomyositis Research Group (JDRG), Zehra S. Arıcı, Gökçen D. Tuğcu, Ezgi D. Batu, Hafize E. Sönmez, Deniz Doğru-Ersöz, Yelda Bilginer, Beril Talim, Nural Kiper, Seza Özen, Alexander Solyom, Boris Hügle, Balahan Makay, Bo Magnusson, Ezgi Batu, John Mitchell, Ariana Kariminejad, Fatemeh Hadipour, Zahra Hadipour, Marta Torcoletti, Carlo Agostoni, Maja Di Rocco, Pranoot Tanpaiboon, Andrea Superti-Furga, Luisa Bonafé, Nur Arslan, Norberto Guelbert, Mikhail Kostik, Karoline Ehlert, Giedre Grigelioniene, Ratna Puri, Seza Ozen, Edward Schuchman, Clara Malagon, Pilar Gomez, Angela C. Mosquera, Tatiana Gonzalez, Ricardo Yepez, Camilo Vargas, GRIP study group, Falcini Fernanda, Gemma Lepri, Alessandra Ferrari, Donato Rigante, Marco Matucci-Cerinic, Antonella Meini, Gian Marco Moneta, Ivan Caiello, Emiliano Marasco, Rebecca Nicolai, Manuela Pardeo, Claudia Bracaglia, Antonella Insalaco, Luisa Bracci-Laudiero, Fabrizio De Benedetti, Olga Kopchak, Mikhail Kostik, Alexander Mushkin, Alexey Maletin, Balahan Makay, Ezgi D. Batu, Boris Hügle, Nur Arslan, Alexander Solyom, John Mitchell, Edward Schuchman, Seza Ozen, Bo Magnusson, Clara Malagon, Pilar Gomez, Catalina Mosquera, Tatiana Gonzalez, Ricardo Yepez, Camilo Vargas, GRIP study group, Rita A. Amorim, Claudio A. Len, Juliana Molina, Gustavo Moreira, Flávia H. Santos, Melissa Fraga, Livia Keppeke, Vanessa M. Silva, Camila Hirotsu, Sergio Tufik, Maria Teresa Terreri, Vinícius L. Braga, Maria Beatriz Fonseca, Claudio A. Len, Melissa Fraga, Vania Schinzel, Maria Teresa R. Terreri, Juliana Molina, Claudio A. Len, Liliana Jorge, Liana Guerra, Flávia H. Santos, Maria Teresa Terreri, Edson Amaro Junior, Maria Beatriz Fonseca, Vinícius L. Braga, Claudio A. Len, Melissa Fraga, Vania Schinzel, Maria Teresa R. Terreri, Clotilde Alizzi, Maria Cristina Maggio, Maria Cristina Castiglione, Alessandra Tricarico, Giovanni Corsello, Emily Boulter, Andre Schultz, Kevin Murray, Fernanda Falcini, Gemma Lepri, Stefano Stagi, Eleonora Bellucci, Marco Matucci-Cerinic, Ingrid H. R. Grein, Noortje Groot, Gecilmara Pileggi, Natália B. F. Pinto, Aline L. de Oliveira, Nico Wulffraat, Iryna Chyzheuskaya, Lyudmila Belyaeva, Rostislav Filonovich, Helena Khrustaleva, Larisa Zajtseva, Jaanika Ilisson, Chris Pruunsild, Mikhail Kostik, Olga Kopchak, Alexander Mushkin, Alexey Maletin, Olivier Gilliaux, Francis Corazza, Christophe Lelubre, Alina Ferster, Raul Gutiérrez Suárez, on behalf of PANLAR Pediatric Rheumatology Study Group, Zoilo Morel, Graciela Espada, Clara Malagon, Claudia Saad-Magalhães C, Luis Lira, Mabel Ladino, Ruth Eraso, Ivonne Arroyo, Flavio Sztajnbok, Clovis Silva, Carlos Rose, PANLAR Pediatric Rheumatology Study Group

**Affiliations:** 1 Rheumatology, Scientific Center of Children’s Health, Moscow, Russian Federation; 2 Rheumatology, V.A. Nasonova Research Institute of Rheumatology, Moscow, Russian Federation; 3 Rheumatology, Sechenov First Moscow State Medical University, Moscow, Russian Federation; 4 Department of Pediatric Rheumatology, Hyogo Prefectural Kobe Children’s Hospital, Kobe, Japan; 5 Department of Pediatrics, School of Medicine, Institute of Medical Pharmaceutical and Health Sciences, Kanazawa University, Kanazawa, Japan; 6 Osaka Medical and Pharmaceutical University, Takatsuki-city, Japan; 7 Women and Children’s Health, Institute of Translational Medicine, University of Liverpool, Liverpool, UK; 8 Department of Biostatistics, Institute of Translational Medicine, University of Liverpool, Liverpool, UK; 9 Department of Paediatric Rheumatology, Alder Hey Children’s NHS Foundation Trust, Liverpool, UK; 10 University of Liverpool, Liverpool, UK; 11 Albert Einstein College of Medicine, New York, USA; 12 National Institute of Health, Maryland, USA; 13 University of Cape Town, Cape Town, South Africa; 14 Alder Hey Children’s NHS Foundation Trust, Liverpool, UK; 15 Great Ormond Street Hospital, London, UK; 16 Albert Einstein College of Medicine, New York, UK; 17 University of Cape Town, Cape Town, South Africa; 18 Alder Hey Children’s NHS Foundation Trust, Liverpool, UK; 19 Department of Women and Children’s Health, Institute of Translational Medicine, University of Liverpool, Liverpool, UK; 20 Pediatric Rheumatology, National Institute of Health, Maryland, USA; 21 Paediatric Rheumatology, University of Cape Town, Cape Town, South Africa; 22 Department of Biostatistics, Institute of Translational Medicine, University of Liverpool, Liverpool, UK; 23 Paediatric Rheumatology, University of Cape Town, Cape Town, South Africa; 24 Department of Women’s & Children’s Health, Institute of Translational Medicine, University of Liverpool, Liverpool, UK; 25 Department of Pediatrics, Division of Rheumatology, Hacettepe University Faculty of Medicine, Ankara, Turkey; 26 Department of Medical Genetics, Hacettepe University Faculty of Medicine, Ankara, Turkey; 27 Department of Pediatrics, Division of Nephrology-Rheumatology, Akdeniz University Faculty of Medicine, Antalya, Turkey; 28 Department of Pediatrics, Division of Rheumatology, Kocaeli University Faculty of Medicine, Kocaeli, Turkey; 29 Department of Pediatrics, Division of Rheumatology, Erciyes University Faculty of Medicine, Kayseri, Turkey; 30 Department of Pediatrics, Division of Rheumatology, Dokuz Eylul University Faculty of Medicine, İzmir, Turkey; 31 Institute of Translational Medicine, University of Liverpool, Liverpool, UK; 32 NIHR Alder Hey Clinical Research Facility, Alder Hey Children’s NHS Foundation Trust, Liverpool, UK; 33 Pediatrics, Cincinnati Children’s Hosptial Medical Center, Cincinnati, USA; 34 Internal Medicine, University of Cincinnati, Cincinnati, USA; 35 Pediatrics, University of Kansas, Kansas, USA; 36 Pediatrics, Cinccinnati Children’s Hospital Medical Center, Cincinnati, USA; 37 Pediatrics, Cincinnati Children’sHosptial MEdical Center, Cincinnati, USA; 38 Radiology, University of Cincinnati, Cincinnati, USA; 39 Rheumatology and Immunology Service, Department of Paediatric Subspecialties, KK Women’s and Children’s Hospital, Singapore, Singapore; 40 SingHealth Translational Immunology and Inflammation Centre, Singapore Health Services, Singapore, Singapore; 41 Duke-National University of Singapore Medical School, Singapore, Singapore; 42 Leeds Teaching Hospitals, Leeds, UK; 43 NIHR Alder Hay clinical research facility, Liverpool, UK; 44 Sophia Children’s Hospital - Erasmus University Medical Centre, Rotterdam, Netherlands; 45 Institute of Translational Medicine, University of Liverpool, Liverpool, UK; 46 Department of Paediatric Rheumatology, Alder Hey Children’s NHS Foundation Trust, Liverpool, UK; 47 Child Health, Sultan Qaboos University Hospital, Muscat, Oman; 48 Child Health, OMSB, Muscat, Oman; 49 Pharmacy, Sultan Qaboos University, Muscat, Oman; 50 Pediatric Rheumatology Division, Faculdade de Medicina da Universidade de São Paulo, São Paulo, SP Brazil; 51 Pediatric Rheumatology Unit, Faculdade de Medicina da Universidade de São Paulo, São Paulo, SP Brazil; 52 Center for Pediatric and Adolescent Medicine, University Medical Center, Freiburg im Breisgau, Germany; 53 Rheumatology and Clinical Immunology, University Medical Center, Freiburg im Breisgau, Germany; 54 Rheumatology Pediatric Unit, University Hospital Ramón y Cajal, Madrid, Spain; 55 Rheumatology, University Hospital Ramón y Cajal, Madrid, Spain; 56 Pediatric Rheumatology, Istanbul University, Cerrahpasa Medical School, Istanbul, Turkey; 57 Pediatric Cardiology, Istanbul University, Cerrahpasa Medical School, Istanbul, Turkey; 58 Immunology, Instituto Nacional de Pediatria, Mexico City, Mexico; 59 Research Methodology, Instituto Nacional de Pediatria, Mexico City, Mexico; 60 Pediatria, Instituto da Criança FMUSP, São Paulo, Brazil; 61 Laboratório experimental de poluição, São Paulo, Brazil; 62 USP, São Paulo, Brazil; 63 Instituto da Criança FMUSP, São Paulo, Brazil; 64 Department of Pediatric Rheumatology, Ankara Child Health, Hematology, Oncology Education and Research Hospital, Ankara, Turkey; 65 Department of Pediatric Rheumatology, Ankara University School of Medicine, Ankara, Turkey; 66 Department of Pediatric Nephrology, Ankara Child Health, Hematology, Oncology Education and Research Hospital, Ankara, Turkey; 67 Paediatric Rheumatology, University of Cape Town, Cape Town, South Africa; 68 Paediatric Nephrology, University of Cape Town, Cape Town, South Africa; 69 Systemic Autoimmunity Branch, National Institute of Arthritis and Musculoskeletal and Skin Diseases, National Institutes of Health, Bethesda, USA; 70 Pediatric Rheumatology, El Bosque University, Bogota, Colombia; 71 Pediatric Rheumathology Unit, Depatment of Pediatrics, Universidade Federal de São Paulo, São Paulo, Brazil; 72 Pediatric Rheumathology Unit, Faculdade de Medicina da Universidade de São Paulo, São Paulo, Brazil; 73 Division of Rheumatology, Faculdade de Medicina da Universidade de São Paulo, São Paulo, Brazil; 74 Pediatric Rheumatology Division, São Paulo State University (UNESP) – Faculdade de Medicina de Botucatu, Botucatu, Brazil; 75 Department of Pediatrics, PGIMER, Chandigarh, India; 76 Pediatrics, P & A Kyriakou Childrens Hospital, Athens, Greece; 77 Pediatrics, Makarios III Children;s Hospital, Nicosia, Cyprus; 78 Pediatrics, P & A Kyriakou Children’s Hospital, Athens, Greece; 79 Clinical Hospital Center Sestre Milosrdnice, Zagreb, ZAGREB, Croatia; 80 Translational immunology laboratory, VIB TRANSLATIONAL IMMUNOLOGY/KULEUVEN AUTOIMMUNE GENETICS, Leuven, Belgium; 81 Department of Pediatrics, University Hospital Leuven, Leuven, Belgium; 82 Microbiology and immunology, University of Leuven, Leuven, Belgium; 83 Pediatric Rheumatology, Tehran University, Tehran, Islamic Republic of Iran; 84 Rheumatology, Centro Hospitalar São João, Oporto, Portugal; 85 Pediatrics, Centro Hospitalar São João, Oporto, Portugal; 86 Faculty of Medicine of Porto University, Oporto, Portugal; 87 Pediatric Cardiology, Centro Hospitalar São João, Oporto, Portugal; 88 Neonatology, Centro Hospitalar São João, Oporto, Portugal; 89 Rheumatology, Centro Hospitalar São João, Oporto, Portugal; 90 Faculty of Medicine of Porto University, Oporto, Portugal; 91 Pediatric Rheumatology Unit, Centro Hospitalar São João, Oporto, Portugal; 92 ASKLEPIOS, Sankt Augustin, Germany; 93 Charite, Berlin, Germany; 94 Prof Hess Children’s Hospital, Bremen, Germany; 95 Prof. Hess Children’s Hospital, Bremen, Germany; 96 University, Tuebingen, Germany; 97 German Centre Pediatric and Adolescent Rheumatology, Garmisch-Partenkirchen, Germany; 98 Olgahospital, Stuttgart, Germany; 99 German Center for Pediatric and Adolescent Rheumatology, Garmisch-Partenkirchen, Germany; 100 Pediatria II, Reumatologia; PRINTO, Istituto Giannina Gaslini, GENOA, Italy; 101 Rheumatology Unit, Giannina Gaslini Institute, Genoa, Italy; 102 Internal Medicine Division, Research Foundation, and Clinical Pharmacology, Ospedale Papa Giovanni XXIII, Bergamo, Italy; 103 Cardiology Department, Maria Vittoria Hospital and Department of Public Health and Pediatrics, Torino, Italy; 104 Department of Cardiology, University of Athens Medical School, Hippokration Hospital, Athens, Greece; 105 Cardiology Unit, Giannina Gaslini Institute, Genoa, Italy; 106 Clinical Immunology, Department of Medicine, Azienda Ospedaliera-Università, Padua, Italy; 107 Rheumatology Unit, Azienda Ospedaliero-Universitaria di Sassari, Sassari, Italy; 108 University Division, Department of Medical Sciences, Città della Scienza e della Salute, Torino, Italy; 109 University of Genoa, Genoa, Italy; 110 Paediatric Immunology, Great North Children’s Hospital, Newcastle upon Tyne, UK; 111 Musculoskeletal Research Group, Institute of Cellular Medicine, Newcastle University, Manchester, UK; 112 Institute of Health and Society, Newcastle University, Manchester,, UK; 113 Paediatric Rheumatology, Great North Children’s Hospital, Newcastle Upon Tyne, UK; 114 Arthritis Research UK Centre for Epidemiology, Centre for Musculoskeletal Research, The University of Manchester, Manchester, UK; 115 NIHR Manchester Musculoskeletal Biomedical Research Unit, Central Manchester University Hospitals NHS Foundation Trust, Manchester, UK; 116 Pediatria II, Reumatologia; PRINTO, Istituto Giannina Gaslini, Genoa, Italy; 117 Cincinnati Children’s Hospital Medical Center, Cincinnati, USA; 118 RHEKITZ - Rheumatologische Kinderpraxen Tzaribachev, Bad Branstedt, Germany; 119 Cleveland Clinic, Cleveland, USA; 120 Azienda Ospedaliero-Universitaria Meyer, Florence, Italy; 121 Riga Stradins University, Riga, Latvia; 122 Asklepios Klinik Zentrum für Allgemeine Paediatrie und Neonatologie, Sankt Augustin, Germany; 123 University of Utah School of Medicine, Salt Lake City, USA; 124 Levine Children’s Hospital at Carolinas Medical Center, Charlotte, USA; 125 Specially for Children, Austin, USA; 126 Hippokration General Hospital, Thessaloniki, Greece; 127 University of Arkansas Medical Center, Little Rock, USA; 128 Hamburg Centre for Pediatric Rheumatology, Hamburg, Germany; 129 University of Minnesota, Minneapolis, USA; 130 Bristol-Myers Squibb, Princeton, USA; 131 Istituto Gaslini, Genova, Italy; 132 Ospedale Bambin Gesù, Roma, Italy; 133 Istituto Pini, Milano, Italy; 134 Ospedale Meyer, Firenze, Italy; 135 Ospedale Salus Pueri, Padova, Italy; 136 Ospedale Burlo Garofalo, Trieste, Italy; 137 Ospedale Maggiore Policlinico, Milano, Italy; 138 Policlinico, Messina, Italy; 139 Policlinico, Catania, Italy; 140 Clinica Pediatrica, Brescia, Italy; 141 Ospedale di Orvieto, Orvieto, Italy; 142 Policlinico, Chieti, Italy; 143 Seconda Università, Napoli, Italy; 144 Ospedale Panico, Tricase, Italy; 145 Ospedale Regionale Microcitemia, Cagliari, Italy; 146 Policlinico Gemelli, Roma, Italy; 147 Ospedale Perrino, Brindisi, Italy; 148 Ospedale delle Marche, Ancona, Italy; 149 Ospedale Salesi, Ancona, Italy; 150 Policlinico Martino, Messina, Italy; 151 Ospedale dei Bambini, Palermo, Italy; 152 Ospedale Santa Chiara, Pisa, Italy; 153 Ospedale S.Maria Nuova, Reggio Emilia, Italy; 154 Pediatric Rheumatology, ERCIYES UNIVERSITESI TIP FAKULTESI HASTANESI, Kayseri, Turkey; 155 SAINT-PETERSBURG STATE PEDIATRIC MEDICAL UNIVERSITY, Saint-Petersburg, Russian Federation; 156 Women’s and Children’s Health, Karolinska Institutet, Stockholm, Sweden; 157 Paediatric Rheumatology unit, Karolinska Hospital, Stockholm, Sweden; 158 Department of Medicine, Rheumatology unit, Karolinska Institutet, Stockholm, Sweden; 159 Pediatrics, Gulhane Military Medical Academy, Ankara, Turkey; 160 Engineering Sciences, Middle East Technical University, Ankara, Turkey; 161 Pediatric Rheumatology, Gulhane Military Medical Academy, Ankara, Turkey; 162 Institute of Health Sciences, Gulhane Military Medical Academy, Ankara, Turkey; 163 Pathology, Gulhane Military Medical Academy, Ankara, Turkey; 164 Orthopedics and Traumatology, Gulhane Military Medical Academy, Ankara, Turkey; 165 Pediatric Rheumatology, Hacettepe University, Ankara, Turkey; 166 Department of Clinical Immunology, Adult and Paediatric Rheumatology, National Institute of Rheumatology and Physiotherapy, Budapest, Hungary; 167 Department of Health Economics, Corvinus University of Budapest, Budapest, Hungary; 168 School of Pharmacy, Queen’s University Belfast, Belfast, UK; 169 Centre for Infection and Immunity, Queen’s University Belfast, Belfast, UK; 170 Paediatric Immunology, Great North Children’s Hospital, Newcastle upon Tyne, UK; 171 Children’s BMT Unit, Great North Children’s Hospital, Newcastle upon Tyne, UK; 172 Paediatric Rheumatology, Great North Children’s Hospital, Newcastle upon Tyne, UK; 173 Pediatria II, Reumatologia; PRINTO, Istituto Giannina Gaslini, GENOA, Italy; 174 Pediatrics, Gulhane Military Medical Academy, Ankara, Turkey; 175 Pathology, Gulhane Military Medical Academy, Ankara, Turkey; 176 Orthopedics and Traumatology, Gulhane Military Medical Academy, Ankara, Turkey; 177 Institute of Health Sciences, Gulhane Military Medical Academy, Ankara, Turkey; 178 Pediatric Rheumatology, Gulhane Military Medical Academy, Ankara, Turkey; 179 Departament of Pediatric Rheumatology, First Moscow Medical State University I.M. Sechenov, Moscow, Russian Federation; 180 Department of Pediatrics, Regional children’s hospital, Rostov-on-Don, Russian Federation; 181 Paediatric Medicine, Great North Children’s Hospital, Newcastle upon Tyne, UK; 182 Paediatric Rheumatology, Great North Children’s Hospital, Newcastle upon Tyne, UK; 183 Paediatric, V.A. Nasonova Research Institute of Rheumatology, Moscow, Russian Federation; 184 Paediatric, Central Children Hospital FMBA, Moscow, Russian Federation; 185 Pediatric Rheumatology, ERCIYES UNIVERSITESI TIP FAKULTESI HASTANESI, Kayseri, Turkey; 186 Pediatric Rheumatology, University Hospital La Paz, Madrid, Spain; 187 Pediatric Rheumatology, University Hospital 12 de Octubre, Madrid, Spain; 188 Pediatrics, HOSPITAL MUNICIPAL INFANTIL MENINO JESUS, São Paulo, Brazil; 189 Pediatric Rheumatology, University of Basel Children’s Hospital, Basel, Switzerland; 190 Unité romande de rhumatologie pédiatrique, CHUV (Lausanne), University of Lausanne and HUG (Geneva), Lausanne and Geneva, Switzerland; 191 Clinical Trial Unit, University of Basel Hospital, Basel, Switzerland; 192 Pediatrics Infectious Diseases and Vaccines, University of Basel Children’s Hospital, Basel, Switzerland; 193 Pediatric Rheumatology, Children’s Hospital Zurich, Zurich, Switzerland; 194 Pediatric Rheumatology, Children’s Hospital Lucerne, Lucerne, Switzerland; 195 Centre de référence des maladies autoinflammatoires CeRéMAI, Versailles Hospital, Versailles, France; 196 Department of Pediatric Rheumatology, St. Josef-Stift, Sendenhorst, Germany; 197 German Rheumatism Research Center, Berlin, Germany; 198 Institute for Social Medicine, Epidemiology and Health Economics, Charité Universitätsmedizin Berlin, Berlin, Germany; 199 Department of Pediatrics, Asklepios Klinik Sankt Augustin, Sankt Augustin, Germany; 200 German Centre for Rheumatology in Children and Adolescents, Garmisch-Partenkirchen, Germany; 201 Department of Pediatrics, Klinikum Stuttgart, Stuttgart, Germany; 202 Prof. Hess-Kinderklinik, Klinikum Bremen-Mitte, Bremen, Germany; 203 Children’s University Hospital, Charité Universitätsmedizin Berlin, Berlin, Germany; 204 University Medical Center Utrecht, Utrecht, Netherlands; 205 Cerrahpasa Medical School-Istanbul University, Istanbul, Turkey; 206 Università Cattolica Sacro Cuore, Rome, Italy; 207 Boston Children’s Hospital, Boston, USA; 208 Boston Children’s Hospital, Boston, USA; 209 Beth Israel Deaconess Medical Center, Boston, USA; 210 Novartis Pharmaceuticals Corporation, East Hanovar, USA; 211 Flince Research, Brooklyn, USA; 212 Autoinflammatory Alliance, San Fracisco, USA; 213 UCL Institute of Child Health, and Great Ormond Street Hospita, London, UK; 214 Unité Romande de Rhumatologie Pédiatrique, Hospitalier Universitaire Vaudois, Lausanne, Switzerland; 215 University Hospital Tuebingen, Tuebingen, Germany; 216 Cliniques Universitaires Saint Luc and Université Catholique de Louvain, Brussels, Belgium; 217 Novartis Pharma AG, Basel, Switzerland; 218 Novartis Pharmaceuticals Corporation, New Jersey, USA; 219 Novartis Pharma, Beijing, China; 220 University of Toronto, The Hospital for Sick Children, Toronto, Canada; 221 U.O. Pediatria II, Genova, Italy; 222 U.O. Genetica Medica, IIRCCS G. Gaslini, Genova, Italy; 223 Clinica Dermatologica, Policlinico Universitario di Roma “Tor - Vergata”, Roma, Italy; 224 UO Dermatologia, IIRCCS G. Gaslini, Genova, Italy; 225 Pediatria 2, Istituto Giannina Gaslini, Genova, Italy; 226 Department of Paediatrics, University Medical Center Utrecht, Utrecht, Netherlands; 227 Department of Pediatric Nephrology and Rheumatology, Hacettepe University, Ankara, Turkey; 228 National Amyloidosis Centre, Royal Free Campus, University College Medical School, London, UK; 229 Pediatria 2, Istituto Giannina Gaslini, GENOVA, Italy; 230 Departement of Pediatric of Southern Switzerland, ORBV, Bellinzona, Switzerland; 231 Department of Pediatric Nephrology and Rheumatology, Hacettepe University, Ankara, Turkey; 232 Pediatric Rheumatology Unit CHUV, University of Lausanne, Lausanne, Switzerland; 233 Department of Paediatrics, University Medical Center Utrecht, Utrecht, Netherlands; 234 National Amyloidosis Centre, Royal Free Campus, University College Medical School, London, UK; 235 Rheumatology, Institute of Child Health, London, UK; 236 Rheumatology, Great Ormond Street Hospital, London, UK; 237 Immunology, Hospital Clinic, Barcelona, Spain; 238 Infectious Diseases, Great Ormond Street Hospital, London, UK; 239 Allergy and Immunology, Lausanne University Hospital, Lausanne, Switzerland; 240 U.O. Pediatria 2, IST. G. GASLINI, Genova, Italy; 241 Genetic Medicine, Manchester Academic Health Science Centre, University of Manchester, Manchester, UK; 242 Università degli studi di Trieste, Trieste, Italy; 243 Institute Imagine, University Paris Descartes, Paris, France; 244 Pediatric Rheumatology, Istanbul University, Cerrahpasa Medical School, Istanbul, Turkey; 245 Rheumatology, Emergency Clinical County Hospital Cluj, Cluj-Napoca, Romania; 246 1st Pediatric, University of Medicine and Pharmacy Cluj, Cluj-Napoca, Romania; 247 2nd Pediatric, University of Medicine and Pharmacy Cluj, Cluj-Napoca, Romania; 248 Rheumatology, University of Medicine and Pharmacy Cluj, Cluj-Napoca, Romania; 249 2nd Internal Medicine, University of Medicine and Pharmacy Cluj, Cluj-Napoca, Romania; 250 Department of Clinical Immunology and Allergy, University of São Paulo, Sao Paulo, Brazil; 251 Department of Immunogenetics, University of São Paulo, Sao Paulo, Brazil; 252 University of São Paulo, Sao Paulo, Brazil; 253 Division of Rheumatology, Ospedale Pediatrico Bambino Gesù IRCCS, Roma, Italy; 254 Clinical and Experimental Sciences, Immunology and Rheumatology Unit, University of Brescia, Brescia, Italy; 255 Clinical and Experimental Sciences, Pediatric Clinic, University of Brescia, Brescia, Italy; 256 Child Neurology and Psichiatry, Spedali Civili di Brescia, Brescia, Italy; 257 Clinical and Experimental Sciences, Pediatric Clinic, University of Brescia, Brescia, Italy; 258 Department of Pathology, University of Brescia, Angelo Nocivelli Institute for Molecular Medicine, Brescia, Italy; 259 University Department Pro.Sa.M.I. “G. D’Alessandro”, University of Palermo, Palermo, Italy; 260 Pediatric Rheumatology Unit, Hospital Universitario y Politecnico La Fe, Valencia, Spain; 261 Immunology. Biomedical Diagnostic Center, Hospital Clinic, Barcelona, Spain; 262 Paediatric Rheumatology University College London, Institute of Child Health, London, UK; 263 Developmental Endocrinology Research Group, Molecular Genetics Unit, University College London, Institute of Child Health, London, UK; 264 Paediatrics, Singapore, Singapore; 265 Khoo Teck Puat-University Children’s Medical Institute, Singapore, Singapore; 266 Division of Pediatric Rheumatology, Department of Pediatrics, Faculty of Medicine, Dokuz Eylul University, Izmir, Turkey; 267 Pediatric Gastroenterology, Istanbul University, Cerrahpasa Medical School, Istanbul, Turkey; 268 Pediatric Rheumatology, Istanbul University, Cerrahpasa Medical School, Istanbul, Turkey; 269 Child Health, Sultan Qaboos University Hospital, Muscat, Oman; 270 Ophthalmology, Sultan Qaboos University Hospital, Muscat, Oman; 271 Immunology & Rheumatology, HOSPITAL DE PEDIATRÍA GARRAHAN, Buenos Aires, Argentina; 272 Second division of Pediatrics, G. Gaslini Institute, Genova, Italy; 273 Dermatology division, G. Gaslini Institute, Genova, Italy; 274 Department of Human Genetics, G. Gaslini Institute, Genova, Italy; 275 PEDIATRICS, PGIMER, CHANDIGARH,INDIA, CHANDIGARH, India; 276 Histopathology, PGIMER, CHANDIGARH,INDIA, CHANDIGARH, India; 277 Medicine, UCL, Royal Free Hospital, London, UK; 278 DEPARTMENT OF PAEDIATRICS AND ADOLESCENT MEDICINE, GENERAL UNIVERSITY HOSPITAL IN PRAGUE, PRAGUE, Czech Republic; 279 Department of Paediatric Rheumatology, University Hospital Brno, Brno, Czech Republic; 280 Department of Rheumatology, St Ann’s Hospital Brno, University Hospital, Brno, Czech Republic; 281 Department of Allergology and Immunology, St Ann’s Hospital Brno, University Hospital, Brno, Czech Republic; 282 Pediatric Nephrology, Pamukkale University School of Medicine, DENIZLI, Turkey; 283 Pediatric Rheumatology, Pamukkale University School of Medicine, DENIZLI, Turkey; 284 Pediatric Cardiology, Pamukkale University School of Medicine, DENIZLI, Turkey; 285 Cardiology, Pamukkale University School of Medicine, DENIZLI, Turkey; 286 Pediatrics, UNIVERSITY OF TRIESTE, Trieste, Italy; 287 Pediatrics, IRCCS Burlo Garofolo, Trieste, Italy; 288 Genetics, IRCCS Burlo Garofolo, Trieste, Italy; 289 Nasonova Research Institute of Rheumatology, Moscow, Russian Federation; 290 FSBI “Research Centre for Medical Genetics”, Moscow, Russian Federation; 291 Nasonova Research Institute of Rheumatology, Moscow, Russian Federation; 292 FSBI “Research Centre for Medical Genetics”, Moscow, Russian Federation; 293 Scientific Centre of Children’s Health, Moscow, Russian Federation; 294 I.M. Sechenov First Moscow State Medical University, Moscow, Russian Federation; 295 Pediatrics, Niigata University, Niigata, Japan; 296 Serviço de Reumatologia e Doenças Ósseas Metabólicas, Lisbon, Portugal; 297 Unidade de Reumatologia Pediátrica, Serviço de Reumatologia e Departamento de Pediatria, Hospital de Santa Maria, Centro Hospitalar Lisboa Norte, Lisbon, Portugal; 298 Instituto de Medicina Molecular, Faculdade de Medicina de Lisboa, Lisbon, Portugal; 299 Department of Pediatrics B, Pediatric Rheumatology Service, Ruth Children’s Hospital, Rambam Medical Center, Haifa, Israel; 300 Department of Pediatrics B, Pediatric Rheumatology Service, Ruth Children’s Hospital, Rambam Medical Center, Haifa, Israel, appaport Faculty of Medicine, Technion-lsrael Institute of Technology, Haifa, Israel; 301 Department of Pediatrics B, Pediatric Rheumatology Service, Ruth Children’s Hospital, Rambam Medical Center, Haifa, Israel, Rappaport Faculty of Medicine, Technion-lsrael Institute of Technology, Haifa, Israel; 302 Pediatric Rheumatology, Ankara University, Ankara, Turkey; 303 Pediatric Rheumatology, Child Health, Hematology and Oncology Education and Research Hospital, Ankara, Turkey; 304 Pediatric Rheumatology, Kırıkkale University, Kırıkkale, Turkey; 305 UNIVERSITA’ DI GENOVA, GENOVA, Italy; 306 Pediatria II, IRCCS G.GASLINI, GENOVA, Italy; 307 Pediatric Rheumatology, CLINIC OF PEDIATRICS, CLINICAL CENTER NIS, Nis, Serbia; 308 Faculty of Medicine, University of Nis, Nis, Serbia; 309 Institute of Rheumatology, Belgrade, Serbia; 310 Institute for Biochemistry, Faculty of Medicine, University of Niš, Nis, Serbia; 311 Pediatria II, Reumatologia, Istituto Giannina Gaslini, Genoa, Italy; 312 UOC Pediatria, AO “Card.G.Panico”, Tricase, Italy; 313 Klaipeda Children’s Hospital, Klaipeda, Lithuania; 314 Istituto Giannina Gaslini, Genoa, Italy; 315 Sophia Children’s Hospital - Erasmus University Medical Centre, Rotterdam, Netherlands; 316 Sophia Children’s Hospital - Erasmus University Medical Centre, Rotterdam, Netherlands; 317 Pediatric Rheumatology, Shriner Hospital For Children Mexico City, Mexico, Mexico; 318 Department of Pediatrics, University Hospital of North Norway, Tromsø, Norway; 319 Institute of Clinical Medicine, The Arctic University of Norway UIT, Tromsø, Norway; 320 Department of Women’s and Children’s Health, Uppsala University Hospital, Uppsala, Sweden; 321 Department of Pediatrics, Ryhov County Hospital, Jonkoping, Sweden; 322 Department of Pediatrics, Helsinki University Children’s Hospital, Helsinki, Finland; 323 Pediatric Clinic II, Copenhagen University Clinic Rigshospitalet, Copenhagen, Denmark; 324 Department of Pediatrics, Århus University Hospital, Århus, Denmark; 325 Department of Laboratory Medicine, Children’s and Women’s Health, NTNU, Trondheim, Norway; 326 Department of Pediatrics, Levanger Hospital, Levanger, Norway; 327 Department of Pediatrics, University of Gothenburg, Gothenburg, Sweden; 328 Department of Pediatrics, St Olavs Hospital, Trondheim, Norway; 329 Rheumatology, Hospital Garcia de Orta, Almada, Portugal; 330 Rheumatology Research Unit, Instituto de Medicina Molecular da Faculdade de Medicina de Lisboa, Lisbon, Portugal; 331 Rheumatology, Hospital Garcia de Orta, Almada, Portugal; 332 Portuguese Society of Rheumatology, Lisbon, Portugal; 333 CEDOC, Nova Medical School, Lisbon, Portugal; 334 Rheumatology, Lisbon Medical Centre, Hospital de Santa Maria, Centro Hospitalar de Lisboa Norte, Lisbon, Portugal; 335 Pediatrics, Centro Hospitalar da Universidade de Coimbra, Coimbra, Portugal; 336 Rheumatology, Portuguese Institute of Rheumatology, Lisbon, Portugal; 337 Rheumatology, Unidade de Saude do Alto Minho, ULSAM, Hospital Conde de Bertiandos, Ponte de Lima, Portugal; 338 Rheumatology, Hospital do Divino Espírito Santo, Ponta Delgada, São Miguel, Portugal; 339 Rheumatology, Hospital Dr. Nélio Mendonça, Funchal, Portugal; 340 Rheumatology, Centro Hospitalar São João, Porto, Portugal; 341 EPIDOC, Nova Medical School, Lisbon, Portugal; 342 Rheumatology, HOSPITAL EGAS MONIZ, CHLO, EPE, Lisbon, Portugal; 343 Rheumatology, Instituto de Medicina Molecular, UIR, Lisbon, Portugal; 344 Rheumatology, CEDOC, Nova Medical School, Lisbon, Portugal; 345 Rheumatology, Hospital Garcia de Orta, Lisbon, Portugal; 346 Portuguese Society of Rheumatology, Lisbon, Portugal; 347 Nova Medical School, Lisbon, Portugal; 348 Rheumatology, Lisbon Medical Centre - Santa maria Hospital, Centro Hospitalar de Lisboa Norte, EPE, Lisbon, Portugal; 349 Pediatric, Centro Hospitalar de Coimbra, Coimbra, Portugal; 350 Rheumatology, Portuguese Institute of Rheumatology, Lisbon, Portugal; 351 Rheumatology, Centro Hospitalar do Alto Minho, ULSAM, Ponte de Lima, Portugal; 352 Rheumatology, Hospital do Divino Espírito Santo, Ponta Delgada, São Miguel, Portugal; 353 Rheumatology, Hospital Dr. Nélio Mendonça, Funchal, Madeira, Portugal; 354 Rheumatology, centro Hospitalar de São João, Porto, Portugal; 355 CEDOC, Lisbon, Portugal; 356 EPIDOC, Nova Medical School, Lisbon, Portugal; 357 School of Health and Science, Faculty of Health and Medicine, The University of Newcastle, Ourimbah, Australia; 358 School of Health and Science, Faculty of Health and Medicine, The University of Newcastle, Ourimbah, Australia; 359 Physiotherapy, The West Meads Children’s Hospital, Westmead, Australia; 360 Institute for Applied Health Research, School of Health & Life Sciences, Glasgow Caledonian University, Glasgow, UK; 361 Sydney Children Hospital (SCH) Randwick, Sydney, Australia; 362 Paediatrics and Child Health, The University of Sydney, Sydney, Australia; 363 The University of NSW, The University of Sydney, Sydney, Australia; 364 School of Health and Science, Faculty of Health and Medicine, The University of Newcastle, Ourimbah, Australia; 365 Paediatrics and Child Health, The University of Sydney, Sydney, Australia; 366 Sydney Children Hospital (SCH) Randwick, Sydney, Australia; 367 The University of NSW, The University of Sydney, Sydney, Australia; 368 National Centre for Paediatric Rheumatology, Our Lady’s Children’s Hospital Crumlin, Dublin, Ireland; 369 Rheumatology, St Vincent’s University Hospital, Dublin, Ireland; 370 Rheumatology, Trinity College, Dublin, Ireland; 371 Department of Child Health and Disease, Gulhane Military Medical Academy, Ankara, Turkey; 372 Department of Child Rheumatology, Gulhane Military Medical Academy, Ankara, Turkey; 373 Faculty of Health Sciences, Division of Physiotherapy and Rehabilitation, Istanbul University, Istanbul, Turkey; 374 Medical Faculty of Cerrahpasa, Department of Pediatric Rheumatology, Istanbul University, Istanbul, Turkey; 375 Rheumatology, Royal Children’s Hospital, Melbourne, Australia; 376 Istituto G. Gaslini, Genova, Italy; 377 Rheumatology, Cincinnati Children’s Hospital Medical Center, Cincinatti, USA; 378 Pediatrics & Communicable Diseases, University of Michigan, Michigan, USA; 379 Columbia University Medical Center, New York, USA; 380 Immunology/Rheumatology, Stanford University, Stanford, USA; 381 Rheumatology/Allergy, Johns Hopkins Arthritis Center, Baltimore, USA; 382 University of Santo Tomas Hospital, Manila, Philippines; 383 Pediatric Rheumatology, University Children’s Hospital, Medical University Sofia, Sofia, Bulgaria; 384 Medical Chemistry and Biochemistry, Molecular Medicine Center, Medical University Sofia, Sofia, Bulgaria; 385 Central Clinical Laboratory, University Hospital “Alexandrovska”, Medical University Sofia, Sofia, Bulgaria; 386 Rheumatology Service, Hospital São Lucas da PUCRS, Porto Alegre, Brazil; 387 Internal Medicine, UNIVERSIDADE LUTERANA DO BRAZIL, Canoas, Brazil; 388 Molecular biology, UNIVERSIDADE FEDERAL DO RIO GRANDE DO SUL, Porto Alegre, Brazil; 389 Pediatrics, Hospital São Lucas da PUCRS, Porto Alegre, Brazil; 390 Division of Paediatric Rheumatology and Immunology, University Hospital Centre Zagreb, Zagreb, Croatia; 391 Musculoskeletal Research Group, Institute of Cellular Medicine, Newcastle University, Newcastle Upon Tyne, UK; 392 Paediatric Rheumatology, Great North Children’s Hospital, Newcastle Upon Tyne, UK; 393 BoxModel Digital Media, Newcastle Upon Tyne, UK; 394 Everything Different, Newcastle Upon Tyne, UK; 395 Department of Pediatrics, Jaslok Hospital and Research Center, Mumbai, India; 396 Paediatric Rheumatology, University of Alberta, Edmonton, Canada; 397 Institute of Health and Society, Newcastle University, Newcastle Upon Tyne, UK; 398 Department of Paediatrics and Adolescent Medicine, General University Hospital and 1st Medical Faculty Charles University in Prague, Prague, Czech Republic; 399 Department of Pediatrics, Masaryk University Brno and Faculty Hospital Brno, Brno, Czech Republic; 400 Institute of Biostatistics and Analyses, Faculty of Medicine and the Faculty of Science of the Masaryk University, Brno, Czech Republic; 401 Department of Pediatrics, Siriraj Hospital, Mahidol University, Bangkok, Thailand; 402 Pediatrics, Faculty of medicine Ramathibodi hospital, Mahidol university, Bangkok, Thailand; 403 Section for clinical epidemiology and biostatistics, Faculty of medicine Ramathibodi hospital, Mahidol university, Bangkok, Thailand; 404 Pediatria II, Reumatologia, Istituto Giannina Gaslini, Genoa, Italy; 405 Istituto G. Gaslini, Genova, Italy; 406 Dipartimento Universitario Pro.Sa.M.I. “G. D’Alessandro”, Università degli Studi di Palermo, Palermo, Italy; 407 Division of Pediatric Rheumatology, Children’s of Alabama, Birmingham, USA; 408 Unit of Medical Genetics, Laboratory of Cytogenetics and Molecular Genetics, Roma, Italy; 409 Division of Rheumatology, Ospedale Pediatrico Bambino Gesù IRCCS, Roma, Italy; 410 Division of Rheumatology, Ospedale Pediatrico Bambino Gesù IRCCS, Roma, Italy; 411 SA Novimmune, Geneva, Switzerland; 412 University of Genova, Istituto Giannina Gaslini, Genova, Italy; 413 Division of Pediatric Rheumatology, Cincinnati Children’s Hospital Medical Center, Ohio, USA; 414 Department of Pediatric Rheumatology and Immunology, University Children’s Hospital Muenster, Muenster, Germany; 415 Department of Pediatric Rheumatology and Immunology, University Children’s Hospital Muenster, Muenster, Germany; 416 Divisions of Rheumatology, Cincinnati Children’s Hospital Medical Center, Cincinnati, USA; 417 Laboratory of Translational Immunolgy, Utrecht, Netherlands; 418 Department of Pediatric Rheumatology and Immunology, University Medical Center Utrecht, Utrecht, Netherlands; 419 Department of Cellular Biotechnology and Hematology, Sapienza University of Rome, Rome, Italy; 420 UO Reumatologia, IRCCS Ospedale Pediatrico Bambino Gesù, Rome, Italy; 421 Department of Women’s and Children’s Health, Karolinska Institutet, Stockholm, Sweden; 422 Childhood Cancer Research Unit, Karolinska University Hospital Solna, Stockholm, Sweden; 423 Center for Chronic Immunodeficiency, University Hospital Freiburg, Freiburg, Germany; 424 Center for Chronic Immunodeficiency, University Hospital Freiburg, Freiburg, Germany; 425 Department of Pediatric Hematology and Oncology, University Medical Center Hamburg Eppendorf, Hamburg, Germany; 426 UO Reumatologia, RCCS Ospedale Pediatrico Bambino Gesù, Rome, Italy; 427 Department of Pediatric Hematology and Oncology, Children’s Hospital, Technische Universität München, Munich, Germany; 428 Istituto G. Gaslini, Genoa, Italy; 429 Karolinska University Hospital, Stockholm, Sweden; 430 Hospital de Ninos Ricardo Gutierrez, Buenos Aires, Argentina; 431 Children’s Hospital of Fudan, Shanghai, China; 432 Ospedale Pediatrico Bambino Gesù, Rome, Italy; 433 University Medical Center, Hamburg, Germany; 434 Rikshospitalet, Oslo, Norway; 435 Seattle Children’s Hospital, Seattle, WA USA; 436 Hospital for Sick Children, Toronto, Canada; 437 University of Alabama, Birmingham, AL USA; 438 Division of Rheumatology, Bambino Gesù Children’s Hospital, Rome, Italy; 439 NovImmune SA, Geneva, Switzerland; 440 Department of Cellular Biotechnologies and Haematology, Sapienza University of Rome, Rome, Italy; 441 MnS, Dinant, Belgium; 442 NovImmune S.A., Geneva, Switzerland; 443 Division of Rheumatology, Ospedale Pediatrico Bambino Gesù IRCCS, Roma, Italy; 444 Department of Pediatrics, Yamaguchi University Graduate School of Medicine, Ube, Japan; 445 Pediatrics, Yokohama City University School of Medicine, Yokohama, Japan; 446 Pediatrics, Saitama MedicalCenter, Saitama Medical University, Kawagoe, Japan; 447 Royal Hospital for Children, Glasgow, UK; 448 UNIKINDERKLINK TUEBINGEN, Tuebingen, Germany; 449 Paediatrics, Division of Rheumatology, UNIKINDERKLINK TUEBINGEN, Tuebingen, Germany; 450 Paediatrics, Division of Rheumatology, Wilhelmina Kinderziekenhuis, Utrecht, Netherlands; 451 Paediatrics, Division of Rheumatology, ALBERTA CHILDRENS HOSPITAL, Calgary, Canada; 452 Bashkir State Medical University, Ufa, Russia; 453 Institute of Biochemistry and Genetics, Ufa, Russian Federation; 454 Rheumatology Unit, Second University of Naples, Naples, Italy; 455 Infection, Inflammation and Rheumatology Section, Institute of Child Health, UCL, London, UK; 456 Clinical Genetics Department, Great Ormond Street Hospital, London, UK; 457 Infection, Inflammation and Rheumatology Section, Institute of Child Health, UCL, London, UK; 458 National Centre for Paediatric Rheumatology, Our Lady’s Children’s Hospital Crumlin, Dublin, Ireland; 459 Rheumatology, St Vincent’s University Hospital, Dublin, Ireland; 460 National Centre for Paediatric Rheumatology, Our Lady’s Children’s Hospital Crumlin, Dublin, Ireland; 461 Rheumatology, St Vincent’s University Hospital, Dublin, Ireland; 462 Rheumatology, Institute of Child Health, London, UK; 463 Rheumatology, Great Ormond Street Hospital, London, UK; 464 Genetics, Great Ormond Street Hospital, London, UK; 465 Genetics, London Northwest Healthcare NHS Trust, London, UK; 466 Infectious Diseases, Great Ormond Street Hospital, London, UK; 467 Pediatrics, RWTH AACHEN UNIVERSITY, Aachen, Germany; 468 Pediatrics, Deutsches Zentrum für Kinderrheumatologie, Garmisch-Partenkirchen, Germany; 469 IZKF, RWTH AACHEN UNIVERSITY, Aachen, Germany; 470 Institute of Immunology, Muenster, Germany; 471 Division of Pediatrics, UMC Utrecht, Utrecht, Netherlands; 472 Institute of Human Genetics, Muenster, Germany; 473 LIMES-Institute, Bonn, Germany; 474 Multiplex Core Facility, UMC Utrecht, Utrecht, Netherlands; 475 Institute of Neurology, Muenster, Germany; 476 Paediatric Rheumatology Unit, Hospital Universitario Vall d’Hebron, Barcelona, Spain; 477 Immunology, Hospital Universitario Vall d’Hebron, Barcelona, Spain; 478 Rheumatology, Hospital Universitario Vall d’Hebron, Barcelona, Spain; 479 Pediatrics, 4th City Children’s Clinical Hospital, Minsk, Belarus; 480 Pediatrics, Belarusian Medical Academy of Postgraduate Education, Minsk, Belarus; 481 Pediatric, Belarusian Medical Academy of Postgraduate Education, Minsk, Belarus; 482 Pediatric, 4th City Children’s Clinical Hospital, Minsk, Belarus; 483 Scientific Research Laboratory, Belarusian Medical Academy of Postgraduate Education, Minsk, Belarus; 484 Pediatrics, 4th City Children’s Clinical Hospital, Minsk, Belarus; 485 Pediatrics, Belarusian Medical Academy of Postgraduate Education, Minsk, Belarus; 486 Pediatric, Belarusian Medical Academy of Postgraduate Education, Minsk, Belarus; 487 Pediatric, 4th City Children’s Clinical Hospital, Minsk, Belarus; 488 Scientific Research Laboratory, Belarusian Medical Academy of Postgraduate Education, Minsk, Belarus; 489 Pediatrics, Medical University Innsbruck, Innsbruck, Austria; 490 Division of Hygiene and Medical Microbiology, Medical University Innsbruck, Innsbruck, Austria; 491 Pediatric, IRCCS Burlo Garofolo, Trieste, Italy; 492 Pediatric, University of Trieste, Trieste, Italy; 493 University of Trieste, Trieste, Italy; 494 Pediatric, S. Maria Misericordia Hospital, University of Udine, Udine, Italy; 495 Pediatric, University of Trieste, IRCCS Burlo Garofolo, Trieste, Italy; 496 IRCCS Burlo Garofolo, Trieste, Italy; 497 Department of Physiotherapy, Department of Pediatrics, Aarhus University Hospital, Aarhus N, Denmark; 498 Department of Pediatrics, Aarhus University Hospital, Aarhus N, Denmark; 499 Department of pediatrics and adolscent medicine, Copenhagen University Hospital, Rigshospitalet, Copenhagen, Denmark; 500 Department of pediatrics, Naestved hospital, Naestved, Denmark; 501 Institute of Inflammation Research, Copenhagen University Hospital, Rigshospitalet, Copenhagen, Denmark; 502 Department of Pediatrics, Aarhus University Hospital, Aarhus, Denmark; 503 Department of Pediatrics, University Hospital of North Norway, Tromsø, Norway; 504 Department of Women’s and Children’s Health, Uppsala University, Uppsala, Sweden; 505 Department of Pediatrics, University of Gothenburg, Gothenburg, Sweden; 506 Department of Laboratory Medicine, Children’s and Women’s Health, Norwegian University of Science and Technology and Department of Pediatrics, St.Olavs Hospital, Trondheim, Norway; 507 Department of Allergology, Rheumatology and Clinical Immunology, PAEDIATRIC CLINIC OF UNIVERSITY CLINICAL CENTRE LJUBLJANA, Ljubljana, Slovenia; 508 Institute of Biochemistry, Medical Faculty, University of Ljubljana, Ljubljana, Slovenia; 509 PRINTO-IRCCS, Genova, Italy; 510 PRCSG, Cincinnati, USA; 511 Hôpital Necker-Enfants Malades, Paris, France; 512 AbbVie Deutschland GmbH & Co. KG, Ludwigshafen, Germany; 513 Paediatric Rheumatology International Trials Organisation (PRINTO), Genoa, Italy; 514 Pfizer, Collegeville, PA USA; 515 Department of Pediatrics, Oulu University Hospital and University of Oulu, Oulu, Finland; 516 Medical Research Center Oulu, Oulu University Hospital and University of Oulu, Oulu, Finland; 517 Murdoch Childrens Research Institute, Melbourne, Australia; 518 Royal Childrens Hospital, Melbourne, Australia; 519 Istituto Giannina Gaslini, Genoa, Italy; 520 Karolinska Institute, Stockholm, Sweden; 521 Wilhelmina Children’s Hospital, Utrecht, Netherlands; 522 University Hospital Leuven, Leuven, Belgium; 523 Seattle Children’s Hospital, Seattle, USA; 524 Meir Medical Centre, Kfar Saba, Israel; 525 Mount Sinai Medical Center, Miami, USA; 526 Hospital for Sick Children, Toronto, Canada; 527 Hospital de Pediatria Juan P Garrahan, Buenos Aires, Argentina; 528 Bristol Royal Hospital for Children, Bristol, UK; 529 University Children’s Hospital, Zurich, Switzerland; 530 Hacettepe University, Ankara, Turkey; 531 14St Jude Children’s Research Hospital, Memphis, USA; 532 University of Calgary, Calgary, Canada; 533 Cincinnati Children’s Hospital Medical Center, Cincinnati, USA; 534 University Medical Center, Hamburg, Germany; 535 Aichi Medical University, Nagakute, Japan; 536 Jaslok Hospital and Research Centre, Mumbai, India; 537 Albert Einstein College of Medicine and Children’s Hospital at Montefiore, New York, USA; 538 Ospedale Pediatrico Bambino Gesù, Rome, Italy; 539 The Children’s Hospital of Philadelphia, Philadelphia, USA; 540 University Children’s Hospital, Ljubljana, Slovenia; 541 Azienda Sanitaria Provinciale 7, Ragusa, Italy; 542 University of Alabama at Birmingham, Birmingham, USA; 543 Pediatrics, University of Washington, Seattle, WA USA; 544 Seattle Children’s Hospital, Seattle, WA USA; 545 School of Dentistry, University of Washington, Seattle, WA USA; 546 Seattle Children’s Research Institute, Seattle, WA USA; 547 Medicine, Stanford University, Stanford, CA USA; 548 Stanford University, Stanford, CA USA; 549 Arthritis Research UK Centre for Epidemiology, The University of Manchester, Manchester, UK; 550 NIHR Manchester Musculoskeletal Biomedical Research Unit, Central Manchester University Hospitals NHS Foundation Trust and University of Manchester Partnership, Manchester, UK; 551 Arthritis Research UK Centre for Genetics and Genomics, The University of Manchester, Manchester, Manchester, UK; 552 Centre for MSK Research, Faculty of Medical and Human Sciences, The University of Manchester, Manchester, UK; 553 University of Alabama at Birmingham, Birmingham, AL USA; 554 Hackensack University Medical Center, Hackensack, NJ USA; 555 Harvard, Boston, MA USA; 556 Montefiore, New York, NY USA; 557 CARRA, Durham, NC USA; 558 Duke, Durham, NC USA; 559 Clinical and Experimental Sciences, Pediatric Clinic, University of Brescia, Brescia, Italy; 560 Pediatric Rheumatology, Ospedale Pediatrico Bambino Gesù, Rome, Italy; 561 Institute of Child and Maternal Health, IRCCS “Burlo Garofolo” and University of Trieste, Trieste, Italy; 562 Pediatric Rheumatology, Anna Meyer Children’s Hospital, University of Florence, Florence, Italy; 563 Clinical and Experimental Sciences, Pediatric Clinic, University of Brescia, Brescia, Italy; 564 Physiatry, Spedali Civili di Brescia, Brescia, Italy; 565 Department of Hematology and Oncology, Hôpital Universitaire Des Enfants Reine Fabiola, Université Libre de Bruxelles, Brussels, Belgium; 566 Department of Rheumatology, Hôpital Erasme, Université Libre de Bruxelles, Brussels, Belgium; 567 Department of Ophtalmology, Hôpital Saint Pierre, Université Libre de Bruxelles, Brussels, Belgium; 568 Department of Pediatrics, University of Turin, Regina Margherita Children’s Hospital, Turin, Italy; 569 Department of Pediatrics, University of Turin, Regina Margherita Children’s Hospital, Turin, Italy; 570 University Department Pro.Sa.M.I. “G. D’Alessandro”, University of Palermo, Palermo, Italy; 571 Pediatric Rheumatology Unit, 1st Department of Pediatrics, PEDIATRIC HOSPITAL PAIDON AGHIA SOFIA, University of Athens, Medical School, Athens, Greece; 572 Department of Pediatric and Adolescent Rheumatology, Children’s Hospital Srebrnjak, Zagreb, Croatia; 573 Department of Pediatric and Adolescent Rheumatology, Clinical Hospital Centre Sisters of Charity, Zagreb, Croatia; 574 Department of Pediatric and Adolescent Rheumatology, Children’s Hospital Srebrnjak, Zagreb, Croatia; 575 Clinic of Pediatric Rheumatology, Kanuni Sultan Suleyman Research and Training Hospital, İstanbul, Turkey; 576 Paediatrics, University of Calgary, Calgary, Canada; 577 University of Calgary, Calgary, Canada; 578 McCaig Institute, Calgary, Canada; 579 Department of Art, University of Calgary, Calgary, Canada; 580 1st Department of Pediatrics, Aristotle University,Thessaloniki, Hippokration Hospital, Thessaloniki, Greece; 581 Paediatric Rheumatology, Great Ormond Street Hospital, London, UK; 582 Institute of Child Health, London, UK; 583 Immunology Department, Great Ormond Street Hospital, London, UK; 584 Arthritis Research UK Centre for Adolescent Rheumatology, London, UK; 585 Pediatric rheumatology of Western Switzerland, CHUV and HUG, Lausanne and Geneva, Switzerland; 586 Pediatric rheumatology of Western Switzerland, CHUV, Lausanne, Switzerland; 587 Maxillo-facial surgery, Geneva, Switzerland; 588 Pediatric radiology, HUG, Geneva, Switzerland; 589 V.N. Karazin Kharkiv National University, Kharkiv, Ukraine; 590 Reumatología Pediátrica, Hospital Universitario Infantil de San José, Bogotá, Colombia; 591 Reumatología Pediátrica, Care For Kids. Cuidado y Atención Reumatológica para niños, Bogotá, Colombia; 592 Reumatología Pediátrica, Centro Integral de Reumatología. CIREI, Bogotá, Colombia; 593 Reumatología Pediátrica, Hospital de la Misericordia, Bogotá, Colombia; 594 Reumatología Pediátrica, Instituto de Ortopedia Infantil Roosvelt, Bogotá, Colombia; 595 Reumatología Pediátrica, Fundación Cardio Infantil, Bogotá, Colombia; 596 Reumatología Pediátrica, Riesgo de Fractura S.A. CAYRE, Bogotá, Colombia; 597 Reumatologia Pediatrica, IRCCS Fondazione Ca’ Granda Ospedale Maggiore Policlinico, Milano, Italy; 598 Maxillo-Facial and Odontostomatologic Unit, IRCCS Fondazione Ca’ Granda Ospedale Maggiore Policlinico, Milano, Italy; 599 Department of Biomedical, Surgical and Dental Sciences, University of Milan, Milano, Italy; 600 Department of Pediatric Rheumatology, Seattle Children’s Hospital, Seattle, WA USA; 601 Department of Epidemiology, University of Washington, School of Public Health, Seattle, WA USA; 602 Department of Epidemiology, University of Washington, School of Public Health, Seattle, WA USA; 603 Public Health Sciences Division, Fred Hutchison Cancer Research Center, Seattle, WA USA; 604 Department of Epidemiology, University of Washington, School of Public Health, Seattle, WA USA; 605 Bristol-Myers Squibb, Princeton, USA; 606 Mu Sigma, Bangalore, India; 607 Istituto G. Gaslini, Pediatria II, PRINTO, Genoa, Italy; 608 Pediatric Rheumatology Collaborative Study Group, Cincinnati Children’s Hospital Medical Center, Cincinnati, USA; 609 Pfizer Inc, Collegeville, USA; 610 Pfizer Inc, Groton, USA; 611 Pfizer Inc, New York, USA; 612 Pediatric Rheumatology, Klinikum Bad Bramstedt, Bad Bramstedt, Germany; 613 Hamburger Zentrum für Kinder- und Jugendrheumatologie, Hamburg, Germany; 614 Asklepios Klinik, Sankt Augustin, Germany; 615 Randall Children’s Hospital, Portland, USA; 616 Institute of Rheumatic Diseases, Piestany, Slovakia; 617 Department of Pediatric Cardiology and Rheumatology, Medical University of Lodz, Lodz, Poland; 618 University of Minnesota Masonic Children’s Hospital, Minneapolis, USA; 619 St Louis Children’s Hospital ODS Rheumatology and Neurology, Krakow, Poland; 620 Pediatrics, Kagoshima University Hospital, Kagoshima, Japan; 621 Pediatrics, Kanazawa University, Kanazawa, Japan; 622 Pediatrics, Graduate School of Biomedical and Health Sciences, Hiroshima, Japan; 623 Pediatrics, Hyogo Prefectural Kobe Children’s Hospital, Kobe, Japan; 624 Pediatrics, Sapporo Medical University School of Medicine, Sapporo, Japan; 625 Pediatrics, Okayama University Hospital, Okayama, Japan; 626 School of Health Sciences, Faculty of Medicine, Kagoshima University, Kagoshima, Japan; 627 Behavioral Medicine Pain Treatment Service, Karolinska Institute, Stockholm, Sweden; 628 Ped rheum unit, Astrid Lindgren Children’s hospital, Karolinska University hospital, Stockholm, Sweden; 629 Behavioral Medicine Pain Treatment Service, Department of Physiology and Pharmacology Karolinska Institute, Stockholm, Sweden; 630 Ped rheum unit, Astrid Lindgren Children’s hospital, Department of Women’s and Children’s health, Karolinska Institute, Stockholm, Sweden; 631 Pediatrics, Tehran University of Medical Sciences, Tehran, Islamic Republic of Iran; 632 Pediatric Rheumatology Research Group, Rheumatology Research Center, Tehran, Islamic Republic of Iran; 633 Pediatric Rheumatology, Children’s Medical Center, Pediatric Center of Excellence, Tehran, Islamic Republic of Iran; 634 I.M.SECHENOV FIRST MOSCOW STATE MEDICAL UNIVERSITY, Moscow, Russian Federation; 635 Pediatric Rheumatology Unit, Department of Pediatrics, Meir Medical Center, Sackler Faculty of Medicine, Tel Aviv University, Kfar Saba, Israel; 636 Pediatric Rheumatology Unit, Schneider Children’s Medical Center, Petah Tikva, Sackler Faculty of Medicine, Tel Aviv University, Tel Aviv, Israel; 637 Pediatric Rheumatology Unit, Zedek Medical Center, Shaare Hebrew University, Medical School, Jerusalem, Israel; 638 Pediatric Rheumatology Unit, Department of Pediatrics, Meir Medical Center, Kfar-Saba, Sackler Faculty of Medicine, Tel Aviv University, Tel Aviv, Israel; 639 Paediatrics, Riga Stradiņš University, Riga, Latvia; 640 Paediatrics, Children’s University Hospital, Riga, Latvia; 641 IRCCS G.GASLINI, GENOVA, ITALY, Genova, Italy; 642 Paediatric Rheumatology, Great Ormond Street Hospital, London, UK; 643 Laboratory of translational immunology, UMC UTRECHT, Utrecht, Netherlands; 644 Pediatrics, Centre Hospitalier Universitaire Vaudois, Lausanne, Switzerland; 645 Rheumatology & clinical immunology, UMC UTRECHT, Utrecht, Netherlands; 646 Pediatric rheumatology, UCL Institute of Child Health, London, UK; 647 Pediatric rheumatology & immunology, UMC UTRECHT, Utrecht, Netherlands; 648 KING ABDUL AZIZ UNIVERSITY, JEDDAH, Saudi Arabia; 649 Paediatric rheumatology, University Medical Centre Utrecht, Wilhelmina Children’s Hospital, Utrecht, Netherlands; 650 Paediatric rheumatology, Istituto Giannina Gaslini, Genova, Italy; 651 Paediatric rheumatology, University College London Institute of Child Health, London, UK; 652 Paediatric rheumatology, Great Ormond Street Hospital for Children, London, UK; 653 Department of Statistical Science, University College London, London, UK; 654 Paediatric Rheumatology Department, Great Ormond Street Hospital for Children, London, UK; 655 Infection, Immunity, Inflammation, and Physiological Medicine Programme, Institute of Child Health, University College London, London, UK; 656 Infection, Inflammation and Rheumatology Section, Institute of Child Health – UCL, London, UK; 657 Rheumatology, Great Ormond Street Hospital for Children NHS Trust, London, UK; 658 Rheumatology, Santa Maria Hospital – CHLN, Lisbon, Portugal; 659 Rheumatology, Instituto Português de Reumatologia, Lisbon, Portugal; 660 Arthritis Research UK Centre for Adolescent Rheumatology, UCL, UCLH, GOSH NHS Trust, London, UK; 661 Rheumatology, Giannina Gaslini Institute, Genova, Italy; 662 Rheumatology, Great Ormond Street Hospital, London, UK; 663 Infection Inflammation and Rheumatology Section, Institute of Child Health, University College London, London, UK; 664 Paediatrics, Faculty of Medicine Ramathibodi Hospital, Mahidol University, Bangkok, Thailand; 665 Department of Pediatric Rheumatology, Hacettepe University Faculty of Medicine, Ankara, Turkey; 666 Department of Pediatric Pulmonology, Hacettepe University Faculty of Medicine, Ankara, Turkey; 667 Department of Pediatric Pathology, Hacettepe University Faculty of Medicine, Ankara, Turkey; 668 Plexcera Therapeutics LLC, New York, USA; 669 German Center for Pediatric Rheumatology, Garmisch-Partenkirchen, Germany; 670 Dokuz Eylul University Hospital, Izmir, Turkey; 671 Karolinska University Hospital, Stockholm, Sweden; 672 Hacettepe University Hospital, Ankara, Turkey; 673 Montreal Children’s Hospital, Montreal, Canada; 674 Kariminejad-Najmabadi Pathology & Genetics Center, Tehran, Islamic Republic of Iran; 675 Sarem Cell Research Center & Hospital, Tehran, Islamic Republic of Iran; 676 IRCCS Policlinico, University of Milan, Milan, Italy; 677 Istituto Giannina Gaslini, Genoa, Italy; 678 Children’s National Medical Center, Washington, DC USA; 679 Lausanne University Hospital (CHUV), Lausanne, Switzerland; 680 Children’s Hospital of Cordoba, Cordoba, Argentina; 681 State Pediatric Medical Academy, St. Petersburg, Russian Federation; 682 University Medical Center Greifswald, Greifswald, Germany; 683 Sir Ganga Ram University Hospital, New Delhi, India; 684 Icahn School of Medicine at Mt. Sinai, New York, USA; 685 Pediatric Rheumatology, El Bosque University, Bogota, Colombia; 686 Pediatric Rheumatology, Universidad Libre, Cali, Colombia; 687 Pediatric Rheumatology, Universidad de Cartagena, Cartagena, Colombia; 688 Pediatric Rheumatology, Universidad del Valle, Cali, Colombia; 689 Section of Rheumatology, Transition Care, UNIVERSITY OF FLORENCE, Florence, Italy; 690 Pediatric Clinic, University of Brescia and Spedali Civili di Brescia, Brescia, Italy; 691 Institute of Pediatrics, Università Cattolica Sacro Cuore, Roma, Italy; 692 Division of Rheumatology, Bambino Gesù Children’s Hospital, Rome, Italy; 693 Institute of Translational Pharmacology, CNR, Rome, Italy; 694 Hospital Pediatry, Saint Petersburg State Pediatric Medical University, Saint Petersburg, Russian Federation; 695 Pediatry, Kirov’s regional children’s hospital, Kirov, Russian Federation; 696 Pediatric Surgery, FGBU Saint-Petersburg Science-Research Institute of Phthisiopulmonology Ministry of public health, Saint Petersburg, Russian Federation; 697 Pediatric Rheumatology, Dokuz Eylul University Hospital, Izmir, Turkey; 698 Pediatric Rheumatology, Hacettepe University Hospital, Ankara, Turkey; 699 German Center for Pediatric Rheumatology, Garmisch-Partenkirchen, Germany; 700 Pediatric Metabolism and Nutrition, Dokuz Eylul University Hospital, Izmir, Turkey; 701 Clinical Research, Plexcera Therapeutics LLC, New York, USA; 702 Pediatric Endocrinology, Montreal Children’s Hospital, Montreal, Canada; 703 Genetics and Genomic Sciences, Icahn School of Medicine at Mt. Sinai, New York, USA; 704 Pediatric Rheumatology, Karolinska University Hospital, Stockholm, Sweden; 705 Pediatric Rheumatology, El Bosque University, Bogota, Colombia; 706 Pediatric Rheumatology, Universidad Libre, Cali, Colombia; 707 Pediatric Rheumatology, Universidad de Cartagena, Cartagena, Colombia; 708 Pediatric Rheumatology, Universidad del Valle, Cali, Colombia; 709 Pediatrics, Universidade Federal de São Paulo, São Paulo, Brazil; 710 Sleep Institute, Universidade Federal de São Paulo, São Paulo, Brazil; 711 University of Minho, Braga, Portugal; 712 Pediatrics, Universidade Federal de São Paulo, São Paulo, Brazil; 713 Pediatrics, Universidade Federal de São Paulo, São Paulo, Brazil; 714 Brain Institute, Hospital Israelita Albert Einstein, São Paulo, Brazil; 715 University of Minho, Braga, Portugal; 716 Pediatrics, Universidade Federal de São Paulo, São Paulo, Brazil; 717 Azienda Ospedaliera di Rilievo Nazionale e di Alta specializzazione “Civico-G.Di Cristina-Benfratelli”, Palermo, Italy; 718 Rheumatology and Metabolic Medicine, Perth, Australia; 719 Respiratory Medicine, Princess Margaret Hospital, Perth, Australia; 720 Telethon Kids Institute, Perth, Australia; 721 School of Paediatric and Child Health, University of Western Australia, Perth, Australia; 722 Section of Rheumatology, Florence, Italy; 723 Health Sciences Department, Anna Meyer Children’s University Hospital, University of Florence, Florence, Italy; 724 Pediatric Rheumatology Department, Hospital Pequeno Príncipe, Curitiba, Brazil; 725 Pediatric Immunology and Rheumatology Department, Wilhemina Children’s Hospital/University Medical Centre Utrecht, Utrecht, Netherlands; 726 Pediatric Immunology Department, Sophia Children’s Hospital - Erasmus MC, Rotterdam, Netherlands; 727 Pediatric Immunology and Rheumatology Department, Ribeirão Preto Medical School - University of São Paulo, São Paulo, Brazil; 728 Belarusian Medical Academy of Postgraduate Education, Minsk, Belarus; 729 4th City Children’s Clinical Hospital of Minsk, Minsk, Belarus; 730 Children’s Clinic, Tartu University Hospital, Tartu, Estonia; 731 Children’s Clinic, Institute of Clinical Medicine, Tartu University, Tartu, Estonia; 732 Hospital Pediatry, Saint Petersburg State Pediatric Medical University, Saint Petersburg, Russian Federation; 733 Pediatry, Kirov’s regional children’s hospital, Kirov, Russian Federation; 734 Pediatric Surgery, FGBU Saint-Petersburg Science-Research Institute of Phthisiopulmonology Ministry of public health, Saint Petersburg, Russian Federation; 735 Department of Pediatrics, Hôpital Civil Marie Curie - CHU de Charleroi, Charleroi, Belgium; 736 Laboratory of Immunology, CHU Brugmann, Brussels, Belgium; 737 Department of Internal Medicine, Hôpital Civil Marie Curie - CHU de Charleroi, Charleroi, Belgium; 738 Department of Pediatric Hematology and Oncology, Hôpital Universitaire des Enfants Reine Fabiola, Université Libre de Bruxelles (ULB), Brussels, Belgium; 739 Pediatric Rheumatology, Shriner Hospital For Children Mexico City, Mexico, Mexico; 740 Hospital de Clínicas, Universidad Nacional de Asunción, Asunción, Paraguay; 741 Rheumatology Section, Children’s Hospital Ricardo Gutierrez, Bueno Aires, Argentina; 742 Pediatric Rheumatology, Universidad El Bosque, Bogotá, Colombia; 743 Pediatric Rheumatology Division, UNESP, Botucatu Medical School, Brazil, Botucatu, Brazil; 744 Pediatric Rheumatology, Chilean Society of Rheumatology, Santiago, Chile; 745 Pediatric Rheumatology, Hospital San Juan de Dios.University of Chile, Santiago, Chile; 746 Pediatric Rheumatology Division, Hospital Pablo Tobón Uribe, Antioquia University, Antioquia, Colombia; 747 Pediatric Rheumatology, Hospital San Juan, San Juan, Puerto Rico; 748 Pediatric Rheumatology Division, Adolescent Health Care Unit, Universida de do Estado do Rio de Janeiro, Rio de Janeiro, Brazil; 749 Pediatric Rheumatology Unit, Faculdade de Medicina da Universidade de São Paulo (FMUSP), São Paulo, Brazil; 750 Pediatric Rheumatology Division, Nemours/Alfred I. duPont Hospital for Children, Wilmington, DE USA

## ᅟ

### P178 Features of drug therapy of patients with systemic juvenile idiopathic arthritis, according to the Russian register of the Russian union of pediatricians

#### Olga Lomakina^1^, Ekaterina Alekseeva^1^, Sania Valieva^1^, Tatiana Bzarova^1^, Irina Nikishina^2^, Elena Zholobova^3^, Svetlana Rodionovskaya^2^, Maria Kaleda^2^

##### ^1^Rheumatology, Scientific Center of Children’s Health, Moscow, Russian Federation; ^2^Rheumatology, V.A. Nasonova Research Institute of Rheumatology, Moscow, Russian Federation; ^3^Rheumatology, Sechenov First Moscow State Medical University, Moscow, Russian Federation

###### **Presenting author:** Olga Lomakina

**Introduction:** Systemic juvenile arthritis - a rare chronic disease. Register - it's an important tool to monitor the effectiveness and safety of GIBP.

**Objectives:** Our aim was to study features of the drug therapy of children with systemic juvenile idiopathic arthritis (sJIA)

**Methods:** We conducted a retrospective data analysis included in the Register of sJIA cases, for the period from 2002 to 2015

**Results:** The indicators of 384 children with sJIA are studied. Prior to the diagnosis verification, all patients were prescribed to intake antipyretic agents, 98% —antibiotics. After the diagnosis, non-steroidal anti-inflammatory drugs (NSAIDs) were intaken by 282 (73.4%) patients: diclofenac sodium — by 163 (40.1%), nimesulide — by 88 (22.9%) patients. The average duration of NSAID intake from 2002 to 2015 decreased from 81.5 ± 115.3 to 3.3 ± 3.7 months (p < 0.001). Prior to the diagnosis verification, glucocorticoids were received intravenously or intramuscularly by 265 (69.0%) patients, orally — 176 (45.8%). Totally, glucocorticoids were received by 330 (85.9%) patients: methylprednisolone — 300 of 384 (78.1%), prednisolone — 174 (45.3%), there were totally 1855 prescriptions in 668 cases. The average duration of glucocorticoid intake from 2002 to 2015 decreased from 13.7 ± 26.7 to 5.0 ± 3,8 months (p < 0.001). As a disease-modifying drug, methotrexate was intaken by 237 (61.7%), Cyclosporin — by 193 (50.6%) patients. There were totally 809 cases of genetically engineered biological preparations (GIBP) in 430 patients: in 2002–2005–8, in 2011–2015–602 in 397 cases (p = 0.001). Tocilizumab is intaken by 210 (52.9%) of 397 patients, kanakinumab — 37 (9.3%) patients. The disease duration from the manifestation to the prescription of immunosuppressive drugs from 2002 to 2015 decreased from 27.3 ± 23.9 to1.0 ± 0 months (p < 0.001), GIBP prescriptions — from 70.7 ± 26.3 to 0.5 ± 0.7 months, respectively (p < 0.001)

**Conclusion:** In 13 years there have been positive changes in the antirheumatic therapy in children with sJIA — the duration of NSAIDs and glucocorticoids intake reduced, the period between diagnosis verification and immunosuppressants and GIBP prescription decreased. However, it is still widely used antibiotics, non-selective NSAIDs and glucocorticoids.

**Disclosure of Interest**

None Declared

### P179 Bicipital synovial cyst associated with systemic juvenile idiopathic arthritis: clinical description, sonographic and pathological findings

#### Yasuo Nakagishi^1^, Masaki Shimizu^2^, Mao Mizuta^2^, Akihiro Yachie^2^

##### ^1^Department of Pediatric Rheumatology, Hyogo Prefectural Kobe Children’s Hospital, Kobe, Japan; ^2^Department of Pediatrics, School of Medicine, Institute of Medical Pharmaceutical and Health Sciences, Kanazawa University, Kanazawa, Japan

###### **Presenting author:** Yasuo Nakagishi

**Introduction:** Bicipital synovial cyst is a rare manifestation of systemic juvenile idiopathic arthritis (s-JIA). It remains unclear how bicipital synovial cysts arise.

**Objectives:** To describe the presentation and clinical course of bicipital synovial cysts in 2 patients with s-JIA and to assess how bicipital synovial cysts arise.

**Methods:** We report 2 patients with bicipital synovial cyst associated with s-JIA. We performed sonographic examinations of bicipital synovial cyst. Furthermore, we investigated pathological examination using biopsy specimen.

**Results: Patient 1:** Eight-year-old boy was diagnosed as s-JIA at the age of 4. His disease course was steroid-dependent and tocilizumab (TCZ) was started from the age of 5. However, his disease relapsed at the age of 7. He presented with fever and swelling of upper left arm. USG revealed a hyperechogenic cyst along the margin of the biceps muscle. The biopsy of the cyst revealed cyst was surrounded by granulation tissue with abundant macrophages infiltrate and there were no synovial tissues. The cyst disappeared with control of disease activity.

**Patient 2:** Twelve-year-old boy was diagnosed as s-JIA at the age of 8. His disease course was steroid-dependent and tocilizumab (TCZ) was started from the age of 12. At the time to start TCZ, he presented with swelling of upper right arm. Ultrasonography revealed a hyperechogenic cyst in the fascia of biceps muscle. The biopsy of the cyst revealed cyst was surrounded by granulation and fibrous tissue with abundant macrophages, lymphocytes and neutrophils infiltrate. There were no synovial tissues. The cyst disappeared with control of disease activity.

**Conclusion:** These findings indicate bicipital synovial cysts arise as follows: the fluid arises within the shoulder joint and then descends into the contiguous bicipital tendon sheath. The tendon eventually ruptures, leading to collection of fluid in the bicipital area and synovial cysts arise from the biceps muscle fasciitis. Bicipital synovial cyst is a rare manifestation of s-JIA but synovial cyst should be considered in the differential diagnosis in all children with s-JIA presenting with swelling of the upper arm.

**Disclosure of Interest**

None Declared

### P180 Systemic juvenile idiopathic arthritis with MEFV gene mutations may have a good response to colchicine: suggestion from 5 cases in our center

#### Yuko Sugita, Nami Okamoto, Kousuke Shabana, Takuji Murata, Hiroshi Tamai

##### Osaka Medical and Pharmaceutical University, Takatsuki-city, Japan

###### **Presenting author:** Yuko Sugita

**Introduction:** Systemic juvenile idiopathic arthritis (SJIA) is considered to be an autoinflammatory disease in which the dysregulation of innate immune response is suggested to be the main pathogenesis, and there are some reports that show SJIA patients have a significantly higher frequency of *MEFV* gene mutations, which is associated to the activation of IL-1β pathway, than healthy control population^1,2^.

**Objectives:** Since the effectiveness of colchicine, which is useful in familial Mediterranean fever, in SJIA patients with *MEFV* gene mutations is not established, we sought to examine whether colchicine is effective in such patients.

**Methods:** We searched for gene mutations that is responsible for autoinflammatory disease in SJIA patients who had persistent clinical symptoms (e.g., skin rashes and arthritis) or flare even under treatment with glucocorticoid, disease-modifying antirheumatic drugs (DMARDS), and IL-6 receptor inhibitor (tocilizumab). We obtained informed consent from the patient or their caregivers, and the gene mutation analysis was performed at Medicine Department of Pediatrics, Kyoto University (Dr. Ryuta Nishikomori). Patients with *MEFV* gene mutations were administered colchicine.

**Results:** The five SJIA patients with *MEFV* gene mutations are summarized in Table 1. All the cases required treatment with IL-6 receptor inhibitor. Case 1 had multiple gene mutations besides *MEFV* gene such as *NOD2*, *PSTPIP1*, and *NLRP12* which is known to be associated with other autoinflammatory diseases. Case 2 once achieved drug free remission, but had a flare and started colchicine during the flare which was not effective to control the activated disease. Case 3 and 4 had recurrent diseases but became stable after the initiation of colchicine administration. In case 5, colchicine was administered after the identification of *MEFV* gene mutation, and was effective to urticarial rashes. Case 3, 4, and 5 is now stable without glucocorticoid after the initiation of colchicine.

**Conclusion:** To SJIA patients with *MEFV* gene mutations who are resistant to standard treatment such as glucocorticoid, DMARDS, and biologic agents, colchicine may be a useful therapeutic options.

**References**

1. N. A. Ayaz, S. Özen, Y. Bilginer, M. Ergüven, E. Taşkıran, E. Yılmaz, et al. MEFV mutations in systemic onset juvenile idiopathic arthritis. Rheumatology 2009;48:23–25.

2. Hala M. Lotfy, Manal E. Kandil, Marianne Samir Makboul Issac, Samia Salah, Nagwa Abdallah Ismail, Mohamed A. Abdel Mawla. MEFV Mutations in Egyptian Children with Systemic-Onset Juvenile Idiopathic Arthritis. Molecular Diagnosis & Therapy. 2014; 18(5): 549-557.

**Disclosure of Interest**

None Declared.

**Table 1 (abstract P180).** Summary of the five SJIA patients with *MEFV* gene mutations

	Sex	Age of onset	*MEFV* mutations	recurrent disease	Effectiveness of colchicine	Other treatments
case 1	F	6	L110P, E148Q	+	+	TCZ, TAC, PSL
case 2	F	13	L110P, E148Q, P369S, R408Q	+	−	TCZ, CyA, PSL
case 3	F	3	L110P, E148Q, G304R	+	+	TCZ
case 4	F	7	G304R	+	+	TCZ
case 5	M	2	E148Q	−	+	TCZ

*F* female, *M* male, *TCZ* tocilizumab, *TAC* tacrolimus, *PSL* prednisolone, *CyA* Cyclosporin A

## Poster Session: Systemic lupus erythematosus and antiphospholipid syndrome I

### P181 Predictors of recovery from lupus nephritis in children

#### Eve M. Smith^1^, Peng Yin^2^, Andrea L. Jorgensen^2^, Michael W. Beresford^1,3^ on behalf of On behalf of the UK JSLE Cohort Study

##### ^1^Women and Children's Health, Institute of Translational Medicine, University of Liverpool, Liverpool, UK ^; 2^Department of Biostatistics, Institute of Translational Medicine, University of Liverpool, Liverpool, UK; ^3^Department of Paediatric Rheumatology, Alder Hey Children's NHS Foundation Trust, Liverpool, UK

###### **Presenting author:** Eve M. Smith

**Introduction:** Within an adult-onset Systemic Lupus Erytematosus (SLE) context, proteinuria has been shown to take a significant period of time to normalise, with 53% of lupus nephritis (LN) patients requiring up to 2 years to recover, and 74% recovering by 5 years [1]. The recovery time from proteinuria in Juvenile-onset SLE (JSLE) has not been well described.

**Objectives:** 1) To describe time to recovery from proteinuria, and to elucidate if clinical/demographic factors at LN onset differentiate patients who do not fully recover. 2) To determine factors at LN onset which influence time to proteinuria recovery.

**Methods:** Participants of the UK JSLE Cohort Study, between 1995-2015, were included if they had biopsy defined LN or active LN based upon the renal domain of the BILAG score (A/B) AND proteinuria of >50 mg/mmol. Univariate logistic regression modelling compared clinical/demographic factors at the time of LN onset in patients who did/did-not recover from proteinuria during the follow-up period. Covariates with p-value <0.2 were included in a multivariable logistic regression model, and backward stepwise variable selection applied. Univariate Cox proportional hazard (Cox PH) regression modelling was used to explore factors associated with time to proteinuria recovery, followed by the same multivariable model selection procedure.

**Results:** 64/350 (18%) JSLE patients fulfilled the inclusion criteria. 25 (39%) recovered from proteinuria within a median of 17 months (min 2.4, max 78). The remaining 39 (61%) had not recovered after a median of 22 months (min 2.3, max 132). The final multivariable logistic regression model showed ethnicity, eGFR, Azathioprine and cardiorespiratory or haematological involvement at time of LN onset to be significant factors differentiating patients who did/did not recover (see Table 2, section A). Using Cox PH regression modelling, age, eGFR and haematological involvement were found to be significantly associated with time to proteinuria recovery (see Table 2, section B).

**Conclusion:** A significant proportion of children with LN have on-going proteinuria after 2 years. Poor prognostic factors include ethnicity, young age, low GFR, azathioprine use and concomitant haematological involvement. Consideration of such factors may help to improve LN outcomes.

**Reference**

1. Touma, Zahi, Urowitz, Murray B, Ibanez, Dominique, Gladman, Dafna D. Time to Recovery From Proteinuria in Lupus Nephritis Patients Receiving Standard of Care Treatment. Arthritis Rheum 2011;63 Suppl 10 :599 DOI: 10.3899/jrheum.130005.

**Disclosure of Interest**

None Declared.

**Table 2 (abstract P181).** See text for description

A) Factors differentiating those who do / do not recover during follow up	Odds ratio (95% CI)	p-value	Interpretation
Ethnicity (Caucasian vs non-Caucasian)	14.19 (2.52, 122.63)	0.007	Non-Caucasians – less likely to recover from proteinuria
eGFR	1.04 (1.02, 1.08)	0.007	Low eGFR at proteinuria onset - less likely to recover
Azathioprine use	0.093 (0.01, 0.78)	0.044	Use of Azathioprine at proteinuria onset – less likely to recover
Haematological involvement	0.13 (0.03, 0.53)	0.007	Haematological involvement – less likely to recover
Cardiorespiratory involvement	11.22 (1.57, 107.54)	0.022	Cardiorespiratory involvement - more likely to recover
B) Factors influencing time to recovery from proteinuria	HR (95% CI)	p-value	
Age (years)	1.38 (1.1, 1.8)	0.007	Younger patients - less likely to recover
eGFR	1.0 (1.0, 1.1)	0.036	Lower eGFR - less likely to recover
Haematological involvement	0.3 (0.1, 0.8)	0.016	Haematological involvement - less likely to recover

### P182 Are urine biomarkers able to predict changes in lupus nephritis disease activity over time? A Markov state-space model of lupus nephritis urine biomarker dynamics

#### Eve M. Smith^1^, Antonio Eleuteri^1^, Beatrice Goilav^2^, Laura Lewandowski^3^, Angel Phuti^4^, Dawn Wahezi^2^, Tamar Rubinstein^2^, Caroline Jones^5^, Paul Newland^5^, Stephen Marks^6^, Rachel Corkhill^1^, Diana Ekdawy^1^, Clarissa Pilkington^6^, Kjell Tullus^6^, Chaim Putterman^7^, Chris Scott^8^, Antony C. Fisher^1^, Michael W. Beresford^1,9^

##### ^1^University of Liverpool, Liverpool, UK, ^2^Albert Einstein College of Medicine, New York, USA, ^3^National Institute of Health, Maryland, USA, ^4^University of Cape Town, Cape Town, South Africa, ^5^Alder Hey Children’s NHS Foundation Trust, Liverpool, UK, ^6^Great Ormond Street Hospital, London, UK, ^7^Albert Einstein College of Medicine, New York, UK, ^8^ University of Cape Town, Cape Town, South Africa, ^9^Alder Hey Children's NHS Foundation Trust, Liverpool, UK

###### **Presenting author:** Eve M. Smith

**Introduction:** A urine ‘biomarker panel’ comprising alpha-1-acid glycoprotein (AGP), ceruloplasmin (CP), transferrin, vascular cell adhesion molecule-1 (VCAM-1), monocyte chemoattractant protein 1 (MCP-1) and lipocalin like prostaglandin D synthase (LPGDS) has been shown to perform to an ‘excellent’ level cross-sectionally for identification of active Lupus Nephritis (LN). The ability of this panel to predict LN flare and remission warrants further investigation.

**Objectives:** To model urine biomarker dynamics in LN, assessing ability to predict flare and remission.

**Methods:** The six novel urinary biomarkers were quantified by ELISA in participants of the UK JSLE Cohort Study (Cohort 1), the Einstein Lupus Cohort (Cohort 2), and the University of Cape Town Lupus Cohort (Cohort 3). Patients were categorised as having active LN (renal domain BILAG score of A, B or C & previous histological confirmation of LN, State 2) or inactive LN (renal domain BILAG score D or E, State 1). A baseline homogeneous Markov model of state transitions was fitted, quoting a corrected Akaike Information Criterion score (AICc). A lower AICc score suggested better model accuracy. Urine biomarkers were explored as factors predicting state transitions. Bayesian multiple imputation of missing variables was used.

**Results:** The study included 184 observations from 57 Cohort 1 patients, 27 from 13 Cohort 2 patients, and 33 from 10 Cohort 3 patients. Across the data set there were 10 transitions from inactive to active LN (1-2 transition), 18 from active to inactive LN (2-1 transition), with 93 and 43 remaining inactive and active respectively. A baseline homogeneous multi-state Markov model of LN activity was fitted, producing a AICc score of 149.0. Each urine biomarker was individually added to the model, identifying AGP and CP as covariates producing the lowest AICc’s. Addition of both biomarkers as predictors of all state transitions led to a worsening of AICc, 157.2. The confidence intervals of hazard ratios for CP on the 1-2 transition and for AGP on the 2-1 transition included the value 1. Re-fitting of the model, whereby AGP only had an effect on 1-2 transitions, and CP on 2-1 transitions, produced a lower AICc, 135.0, favouring this model (see Table 3). Inputting individual patient AGP/CP values to the model can provide 3, 6 and 12 month probabilities of state transition.

**Conclusion:** Within an internationally derived Markov state-space model of LN urine biomarker disease dynamics, AGP was predictive of active LN flare or remaining active, whereas CP was predictive of remission or remaining in-active. To improve patient outcomes, this model must be tested in a larger, prospective, rigorously conducted clinical trial of biomarker led LN monitoring.

**Disclosure of Interest**

None Declared.

**Table 3 (abstract P182).** See text for description

Model characteristics	LN state transition	Baseline	Log AGP	Log CP	AICc
		Hazard ratios for changing state for each covariate and 95% confidence intervals			
AGP and CP as predictors of all LN state transitions	1-2	0.25 (0.03, 1.89)	1.80 (1.08, 3.01)	0.46 (0.17,1.28)	157.2
	2-1	0.19 (0.03, 1.41)	1.22 (0.78, 1.91)	0.33 (0.18, 0.62)	
AGP as predictor of 1-2 transition & CP for 2-1	1-2	0.59 (0.24, 1.45)	1.49 (1.10, 2.02)	-	135.0
	2-1	2.11 (0.90, 4.94)	-	0.60 (0.39, 0.93)	

### P183 Growing international evidence for urine biomarker panels identifying lupus nephritis in children – verification from the South African Western Cape lupus cohort

#### Eve M. Smith^1^, Laura Lewandowski^2^, Angel Phuti^3^, Andrea Jorgensen^4^, Chris Scott^5^, Michael W. Beresford^6^

##### ^1^Department of Women and Children's Health, Institute of Translational Medicine, University of Liverpool, Liverpool, UK; ^2^Pediatric Rheumatology, National Institute of Health, Maryland, USA; ^3^Paediatric Rheumatology, University of Cape Town, Cape Town, South Africa; ^4^Department of Biostatistics, Institute of Translational Medicine, University of Liverpool, Liverpool, UK; ^5^Paediatric Rheumatology, University of Cape Town, Cape Town, South Africa; ^6^Department of Women’s & Children’s Health, Institute of Translational Medicine, University of Liverpool, Liverpool, UK

###### **Presenting author:** Eve M. Smith

**Introduction:** The Paediatric Lupus Erythematosus in South Africa (SA) Cohort has collected clinical data in the Western Cape since 2013. They have demonstrated that their patients often initially present with severe lupus nephritis (LN). Monitoring of such patients can be problematic due to geographical, economic and social constraints faced by families. A range of urine biomarkers for LN have been explored within the UK JSLE Cohort study, with a panel of four (alpha-1-acid glycoprotein (AGP), lipocalin like prostaglandin D synthase (LPGDS), transferrin and ceruloplasmin) demonstrating an ‘excellent’ ability to identify active LN.

**Objectives:** To assess whether the same/different biomarker combinations are of importance for identifying active LN in the Paediatric Lupus Erythematosus in SA Cohort.

**Methods:** Participants of the Paediatric Lupus Erythematosus in SA Cohort attending Red Cross Memorial and Groote Schuur Hospital Hospitals, Cape Town, aged <18 years at diagnosis, were recruited between January 2015-16. Healthy controls (HC’s) with non-inflammatory non-infective diagnoses were also recruited. Patients were categorised as having active LN (renal domain of the BILAG score of A/B & previous histological confirmation of LN) or in-active LN (renal domain of the BILAG score of D/E). Novel urinary biomarkers; AGP, LPGDS, transferrin, ceruloplasmin, vascular cell adhesion molecule-1 (VCAM-1), and monocyte chemoattractant protein 1 (MCP-1) were quantified by ELISA. Measurements were standardized to urinary creatinine. The Mann Whitney-U test was used to compare biomarker levels between groups. Binary logistic regression modeling and receiver operating curve analysis was used to assess combinations of biomarkers for active LN identification.

**Results:** 23 JSLE patients (20 females, 3 males) with a median age of 13.5 years [IQR 12.7-14.9] and disease duration of 2.6 years [IQR 1.8-4.0] were included. 18 HC’s (14 females, 4 males) had a median age of 11 years [IQR 10-12]. All novel urine biomarkers were significantly higher in active than in-active LN patients (corrected p-values, *p*_*c*_ all *<0.01*), with no difference seen between in-active LN patients and HC’s (all *p*_*c*_ *= 1.0*, see Table below). Inclusion of all novel biomarkers in a binary logistic regression model and application of step AIC function to get a final model identified VCAM-1, MCP-1 and LPGDS to be the best combination of biomarkers identifying active LN within the SA cohort, with a combined area under the curve (AUC) of 0.96.

**Conclusion:** This is the first study to look at urine biomarkers in an African JSLE population, highlighting their potential utility in this population. The optimal panel of biomarkers may differ to that of the UK, but requires further investigation in a prospective longitudinal study given the samples size. In SA, it is anticipated that use of a point of care testing device to monitor LN activity in the home or local clinic could help to improve patient monitoring, treatment and outcomes.

**Disclosure of Interest**

None Declared.

**Table 4 (abstract P183).** See text for description

Marker (ngmgCr)	Active LN [med, IQR], n = 9	Inactive LN [med, IQR], n = 14	HC [med, IQR], n = 18	Act vs Inact (*p*_*c*_)	Inact vs HC (*p*_*c*_)
VCAM-1	59 [42-119]	4 [2-11]	4 [2-6]	*0.0003*	*1.0*
MCP-1 (pgmgCr)	1020 [410-3642]	219 [150-334]	296 [186-448]	*0.0258*	*1.0*
LPGDS	2683 [1640-3602]	601 [151-900]	577 [314-765]	*0.0054*	*1.0*
AGP	145435 [54746-250367]	680 [394-2985]	605 [408-1458]	*0.0002*	*1.0*
CP	51714 [34861-177503]	1901 [1140-3276]	1700 [1324-2999]	*0.00003*	*1.0*
TF	63630 [38071-156026]	433 [221-1020]	425 [234-928]	*0.00003*	*1.0*

### P184 Whole exome sequencing in early onset systemic lupus erythematosus

#### Ezgi Deniz Batu^1^, Can Kosukcu^2^, Ekim Taskiran^2^, Sema Akman^3^, Kubra Ozturk^4^, Betul Sozeri^5^, Erbil Unsal^6^, Zelal Ekinci^4^, Yelda Bilginer^1^, Mehmet Alikasifoglu^2^, Seza Ozen^1^

##### ^1^Department of Pediatrics, Division of Rheumatology, Hacettepe University Faculty of Medicine, Ankara, Turkey; ^2^Department of Medical Genetics, Hacettepe University Faculty of Medicine, Ankara, Turkey; ^3^Department of Pediatrics, Division of Nephrology-Rheumatology, Akdeniz University Faculty of Medicine, Antalya, Turkey; ^4^Department of Pediatrics, Division of Rheumatology, Kocaeli University Faculty of Medicine, Kocaeli, Turkey; ^5^Department of Pediatrics, Division of Rheumatology, Erciyes University Faculty of Medicine, Kayseri, Turkey; ^6^Department of Pediatrics, Division of Rheumatology, Dokuz Eylul University Faculty of Medicine, İzmir, Turkey

###### **Presenting author:** Ezgi Deniz Batu

**Introduction:** Systemic lupus erythematosus (SLE) is a multisystemic autoimmune disorder with different genetic and environmental factors playing role in its pathogenesis. Early onset SLE, familial SLE, and syndromic SLE are rare situations which may lead to identification of monogenic defects responsible for the disease. İdentification of monogenic causes through these cases can help us to understand the pathogenic mechanisms in SLE.

**Objectives:** We aimed to discover monogenic defects causing SLE by performing whole exome sequencing (WES) in familial or early-onset SLE cases.

**Methods:** We enrolled 12 pediatric SLE cases (from 7 different families) who had disease onset before 5 years of age and a family history consistent with an autosomal recessive inheritance (affected siblings or parenteral consanguinity). Whole exome sequencing and bioinformatic analyses were performed in six index cases and the suspected mutations were confirmed by Sanger sequencing. Only C1Q gene was analyzed in patient 4 since he had similar features with the first three cases.

**Results:** There was consanguinity in all families. The characteristics of index SLE cases are presented in Table 5. We have demonstrated a homozygous nonsense mutation (c.622C > T/p.Gln208Ter) in *C1QA* gene in two patients; homozygous nonsense mutation (c.79C > T/p.Gln27Ter) in *C1QC* gene in one; homozygous missense mutation (c.100G > A/p.Gly34Arg) in *C1QC* gene in one; homozygous stop codon mutation (c.1945G > C/p.Ala649Pro) in *C1S* gene in one; homozygous frameshift mutation (c.290_291delCA/p.Thr97Ilefs*2) in *DNASE1L3* gene in one patient. There was a candidate novel gene in one patient and functional studies on this gene are ongoing.

**Conclusion:** Five of our patients had homozygous mutations in the genes coding for early complement proteins. The risk to develop pediatric SLE is estimated to be 93% for C1q and 66% for C1s/r. There are less than 90 published cases with homozygous C1q deficiency. The clinical presentations are variable; however, they usually had cutaneous involvement, normal C3, C4 levels and negative anti-dsDNA which was the case in our patients. C1s deficiency is much rarer. The nonsense mutation in C1S gene of our patients was novel. *DNASE1L3* gene encodes for DNase1 enzyme which functions as an endonuclease cleaving DNA. Deletion in *DNASE1L3* gene has been previously reported to be associated with SLE in the study on seven SLE families where it was shown that protein encoded by the mutant *DNASE1L3* completely lacked DNase activity. The variant in *DNASE1L3* gene detected in our patient was the same as the one reported. We suggest that monogenic causes/associations should be sought for an early-onset SLE.

**Disclosure of Interest**

None Declared.

**Table 5 (abstract P184).** Characteristics of pediatric patients with early-onset systemic lupus erythematosus

	Pt 1	Pt 2	Pt 3	Pt 4	Pt 5	Pt 6	Pt 7
SLE in sibling	+	+	-	-	+	-	+
Mutant genes	*C1QA*	*C1QC*	*C1QC*	*C1QA*	*C1S*	*DNASE1L3*	Novel gene (?)
Malar/discoid rash/photosensitivity	+	+	+	+	+	+	-
Oral ulcers	+	+	+	-	+	-	-
Arthritis	+	+	-	-	+	+	-
Nephritis	-	-	-	-	+	+	-
Hematologic involvement	+	+	+	-	+	+	-
Decrease in C3, C4	-	-	-	-	+	+	+
Autoantibodies*	Anti-SM	RF	Anti-SM, anti-SSA, LA	Anti-SM, U1RNP	AntidsDNA, Anti-SSA, U1RNP, anti-histon	AntidsDNA, Anti-CL	AntidsDNA, DC

*Pt* patients, *SLE* systemic lupus erythematosus, *CL* cardiolipin, *LA* lupus anticoagulant, *RF* rheumatoid factor

^*^ ANA was positive in all patients

### P185 Fatigue in JSLE: analysis of prevalence and associations in a large, national cohort of patients

#### Hanna Lythgoe^1,2^, Michael W. Beresford^2^

##### ^1^Institute of Translational Medicine, University of Liverpool, Liverpool, UK; ^2^NIHR Alder Hey Clinical Research Facility, Alder Hey Children's NHS Foundation Trust, Liverpool, UK

###### **Presenting author:** Hanna Lythgoe

**Introduction:** Fatigue is widely recognised as a common, debilitating symptom for patients with systemic lupus erythematosus (SLE) with significant impact on health-related quality of life. The cause of fatigue in SLE patients is poorly understood and likely to be multifactorial. Evidence is conflicting regarding whether fatigue in SLE patients is associated with factors such as disease activity, pain and mood and there are very few studies of fatigue in children with JSLE.

**Objectives:** To define the prevalence of fatigue and its associations within a large, national cohort of children with JSLE.

**Methods:** Patients meeting ≥4 ACR criteria in the UK JSLE Cohort Study were included. Data from the paediatric BILAG (p-BILAG) (completed by clinician), Short Form-36 (SF36) (completed by patient) and the Childhood Health Assessment Questionnaire (CHAQ) was analysed. The SF36 includes a vitality domain measuring energy/fatigue which cumulates in a score from 0–100 where higher scores indicate less fatigue; following review of published normative data we used a conservative estimate of 50 as a cut-off for significant fatigue. Associations between variables was assessed using Spearman’s rank correlation co-efficients. Correlation co-efficients of 0-0.19, 0.2-0.39,0.4-0.59, 0.6-0.79, 0.8-1 were considered as very weak, weak, moderate, strong and very strong respectively.

**Results:** 350 patients were included, for whom there were 1428 and 355 completed p-BILAGs and SF36s respectively. 81% of patients had suffered with fatigue at some point during their disease course, with patients suffering some degree of fatigue on 42% of all pBILAG scores.

Correlation was moderate to strong between fatigue and disease activity where disease activity is recorded by the patient or physician but was weak/very weak when disease activity is measured by ESR as a biochemical indicator of disease activity (Table 6). Moderate to strong associations are seen when comparing fatigue with pain and physical function. Strong correlations are seen on all correlations between patient-reported fatigue and patient-reported disease activity, pain and physical function. Weak/very weak correlations are seen when comparing fatigue with mood and anaemia.

Data from the SF36 vitality scores had a mean score of 56.7 (SD 25.5) which is lower than published normative mean scores in young adults aged 16–19 years (mean 69.5, SD 19.8) with a mean difference of 12.8 (confidence interval 9.8–15.8). Almost half (45.3%) of patients had an SF36 score of less than 50 during their disease course, indicating significant fatigue.

**Conclusion:** Fatigue affects most patients with JSLE and almost half suffer with significant fatigue. Fatigue is associated with increased disease activity, pain and functional disability. Importantly, the strongest associations are seen when these are measured using patient-reported outcomes. These associations need further evaluation of their causality in order to consider future treatment strategies for this resistant and problematic symptom.

**Disclosure of Interest**

None Declared.

**Table 6 (abstract P185).** Associations of BILAG fatigue scores and SF36 vitality scores

		Spearman’s rank correlation coefficients	
Assessed association	Measure used as comparator	BILAG fatigue	SF36 vitality
Disease activity	Physician global activity VAS score	0.53	-0.5
	Patient global VAS score	0.48	**-0.72**
	ESR	0.25	-0.12
Pain	SF36 bodily pain	-0.45	**0.69**
Physical function	CHAQ	0.45	**0.62**
Anaemia	Haemoglobin	0.18	-0.21
Mood	Mood disorder, anxiety disorder or organic depressive illness on p-BILAG	0.17	-0.23

*VAS* visual analogue scale, *ESR* erythrocyte sedimentation rate. Strong correlations are shown in bold. All correlation co-efficients were statistically significant (P < 0.05)

### P186 Evidence of altered blood brain barrier permeability in systemic lupus erythematosus using magnetic resonance imaging

#### Hermine I. Brunner^1^, Gaurav Gulati^2^, Jordan T. Jones^3^, Mekibib Altaye^4^, Jamie Eaton^5^, Mark Difrancesco^6^

##### ^1^Pediatrics, Cincinnati Children's Hosptial Medical Center, Cincinnati, USA; ^2^Internal Medicine, University of Cincinnati, Cincinnati, USA; ^3^Pediatrics, University of Kansas, Kansas, USA; ^4^Pediatrics, Cinccinnati Children's Hospital Medical Center, Cincinnati, USA; ^5^Pediatrics, Cincinnati Children'sHosptial MEdical Center, Cincinnati, USA; ^6^Radiology, University of Cincinnati, Cincinnati, USA

###### **Presenting author:** Hermine I. Brunner

**Introduction:** Neurocognitive dysfunction is a common manifestation of childhood-onset Systemic Lupus Erythematosus (cSLE). Murine models suggest that loss of the blood-brain barrier (BBB) integrity allows brain-reactive proteins to enter the CNS and contribute to SLE-associated pathology. Contrast magnetic resonance imaging (MRI) can provide a measure of BBB integrity, but has risk associated with gadolinium use. We have previously identified multiple areas of gray matter (GM) loss on structural MRI in cSLE patients with neurocognitive deficits. Our aim was to evaluate safe, non-invasive MRI-methods of measuring regional BBB permeability and its relationship with neurocognitive function and regional GM volume in cSLE.

**Objectives:** To evaluate safe, non-invasive MRI-methods of measuring regional BBB permeability and its relationship with neurocognitive function and regional GM volume in cSLE.

**Methods:** Twelve cSLE patients and 12 healthy controls (age, gender, race and socioeconomic status matched) were enrolled. Those with diseases or medications (except prednisone) affecting neurocognitive function were excluded. Cognitive performance was assessed using the *cSLE Neurocognitive Battery*, which probes four cognitive domains: working memory, psychomotor speed, attention, and visuoconstructional ability. Performance in each of these was standardized and expressed as a Z-score. We almost concurrently performed arterial spin labeling (ASL) and diffusion-weighted imaging to measure regional BBB permeability. Voxel-based morphometric analysis was done to measure regional GM volume. Voxel-wise comparisons of capillary permeability were made between the cSLE and control groups. Correlation analysis was performed between regional BBB permeability and cognitive performance Z-scores, as well as local GM volume for the cSLE group.

**Results:** Among the cSLE patients (11 females, 7 African American, mean age 18 ± 6.8 years), 9 were treated with prednisone (median dose 5 mg/d). None was diagnosed with active neuropsychiatric SLE. Group comparison revealed clusters of voxels with significantly greater BBB permeability for cSLE patients than controls, in three regions: the parahippocampal gyrus, the right fusiform and inferior occipital region, and the caudate head. Correlations between BBB permeability and regional GM volume or overall and individual domain Z-scores for neurocognitive performance were not statistically significant, although locations of significant increases in permeability for cSLE closely match our previously identified areas of GM loss and functional changes associated with clinically overt neurocognitive impairment.

**Conclusion:** We present imaging evidence of altered regional BBB permeability in cSLE, using a novel non-invasive MRI technique. The absence of correlation with GM volume or cognitive performance Z-scores, yet similar location to GM loss in previous work in our cSLE cohort suggests that BBB breakdown may precede clinically overt neurocognitive impairment and brain tissue loss. Longitudinal studies are needed to confirm the change in GM volume in relation to BBB permeability over time.

**Disclosure of Interest**

None Declared.

### P187 Imbalance of regulatory immunome influences disease activity of juvenile systemic lupus erythematosus

#### Joo Guan Yeo^1,2^, Jingyao Leong^2^, Loshinidevi D/O Thana Bathi^2^, Thaschawee Arkachaisri^1,3^, Salvatore Albani^1,2,3^

##### ^1^Rheumatology and Immunology Service, Department of Paediatric Subspecialties, KK Women’s and Children’s Hospital, Singapore, Singapore; ^2^SingHealth Translational Immunology and Inflammation Centre, Singapore Health Services, Singapore, Singapore; ^3^Duke-National University of Singapore Medical School, Singapore, Singapore

###### **Presenting author:** Joo Guan Yeo

**Introduction:** The pathogenesis of Systemic Lupus Erythematosus (SLE) hinges on multiple disturbances that perturb the fine balance between immunity and regulation. Traditional investigational approaches have largely been focused on the pathogenic or protective role of individual cell types or even individual molecules. For a multifactorial disease like SLE, this mono-dimensional approach is inadequate. A holistic understanding of the immunome is a critical unmet need.

**Objectives:** Here, we aim to use a multi-pronged and multi-dimensional approach to study both the regulatory and inflammatory components sides of the immune balance concurrently. This approach may have immediate translational potential as it can lead to the identification of immune cells subsets which are relevant mechanistically and clinically.

**Methods:** Peripheral blood mononuclear cells from 6 juvenile SLE patients, stratified by disease activity (SLE disease activity index), out of a cohort of 58 were studied with multi-parametric, multi-dimensional mass cytometry (Cytometry by time-of-flight). Analysis was performed using a machine learning custom software through an unbiased, unsupervised approach based on dimensional reductions followed by automated cell classification and clustering and subsequent stratifications of cell clusters with disease activity.

**Results:** We found clear differences in the composition of the immunome between active and inactive disease with a prevalence of regulatory immune cells subsets and expansion of the IL-10 secreting cells (of B and monocyte/macrophage lineages) observed in inactive disease. Among these subsets, the regulatory B (CD19 + IL10+) cells appear relevant with a higher percentage of them present in inactive disease (median 5% versus 2.6%). The significance of these changes will be further determined with increasing sample size and functional characterisation of these immune subsets through transcriptome analysis.

**Conclusion:** In accordance with our original hypothesis, the imbalance between regulatory and effector immune functions is a cross dimensional feature which spans across both the innate and adaptive arms of the juvenile SLE immunome. Mechanistic features found here may hold a dual translational valency as potential signatures predictive of clinical fate as well as potential targets for intervention.

**Disclosure of Interest**

None Declared.

### P188 Disease activity status in juvenile systemic lupus erythematosus

#### Nagla Abdelrahman^1^, Michael W Beresford^2^, Valentina Leone^1^ and UK JSLE study group supported by the National Institute of Health Research Clinical Research Network

##### ^1^Leeds Teaching Hospitals, Leeds, UK; ^2^NIHR Alder Hay clinical research facility, Liverpool, UK

###### **Presenting author:** Nagla Abdelrahman

**Introduction:** A key aim in the management of Juvenile-onset Systemic Lupus Erythematosus (JSLE) is to achieve inactive disease (ID) and clinical remission (CR). There are no universally agreed definitions of ID & CR; however an evidence based, internationally-accepted consensus process to define ID & CR was reached and published in May 2012 (Mina R, Klein-Gitelman MS, Ravelli A, et al. *Arthritis Care Res)*

**Objectives:** To describe the proportion of patients participating in the UK JSLE Cohort Study that meet the definition criteria of ID and CR. In addition, to identify any association between reaching ID/CR status and the patient’s paediatric British Isles Lupus Activity Group (BILAG) disease activity scores at base line and time to diagnosis.

**Methods:** A retrospective data analysis of prospectively collected data from the UK JSLE Cohort Study (collecting data from 21 sites across the UK) was undertaken. Patients fulfilling American College of Rheumatology (ACR) classification criteria for SLE, aged ≤17 years at diagnosis and having a minimum of 12 months follow up were included. Patient status in meeting definitions of ID and CR as defined by Mina et al were assessed at first, 1 year and last follow up clinic visits. Patients failing to achieve ID/CR were considered being active disease (AD). Patients achieving ID at 1 year and last visit were identified and analyzed against BILAG scores on first visit and time to diagnosis. Continuous data presented as mean (range).

**Results:** 233 patients were included on first visit. Mean age at diagnosis was 12.6 (1.8-17.9) years, mean follow up 4.7 (1-15) years, 82% were females. Data from 93 and 209 patients were available for analysis at 1-year and last visits respectively.

79 patients (85%) at 1 year and 130 (62%) at last follow up were in AD while only 6 (6%) and 18 (9%) achieved ID respectively; the remaining 8 (9%) and 61(29%) patients could not be classified at these time points due to incomplete data.

The percentage of AD decreased over time from 91% on the first visit, to 85% at 1 year and to 62% in the last follow up. Ratio of AD:ID disease patients fell from 35:1 in first visit, to 13:1 at 1 year and to 7:1 at last follow up visit.

Notably, 20 (25%) at 1 year and 15 (12%) of the patients at last follow up visit were in AD because of isolated low lymphocyte count. No statistically significant difference was found comparing patients in AD to patients in ID at 1 year and last follow up in relation to time to diagnosis and BILAG scores

**Conclusion:** The majority of patients failed to achieve the suggested criteria for ID/CR reflecting the high burden of JSLE despite the expanding use of aggressive treatments. However, a significant proportion of patients had isolated blood tests abnormalities with limited clinical significance, which may suggest some limits of the ID criteria. Some of the laboratory requirements to meet the criteria for ID may be over-sensitive and/or not always reflect disease activity state. Larger prospective studies may be required to identify differences between patients achieving and not achieving ID with regards to disease activity at diagnosis, time to diagnosis and treatment received.

**Disclosure of Interest**

None Declared

### P189 Adult outcomes in a large cohort of childhood-onset SLE patients: clinical outcome and quality of life - the CHILL-NL study

#### Noortje Groot, D. Shaikhani, I. E. M. Bultink, M. Bijl, R. J. E. M. Dolhain, Y. K. O. Teng, E. Zirkzee, K. de Leeuw, R. Fritsch-Stork, S. S. M. Kamphuis

##### Sophia Children's Hospital - Erasmus University Medical Centre, Rotterdam, Netherlands

###### **Presenting author:** Noortje Groot

**Introduction:** Systemic lupus erythematosus (SLE) is a rare autoimmune disease which can affect any organ system. SLE with an onset in childhood (cSLE) is thought to be more severe than SLE. Only a few small studies address the adult outcomes of patients with cSLE.

**Objectives:** To investigate the clinical outcomes and health-related quality of life (HRQOL) of 111 adults with cSLE.

**Methods:** Adults with cSLE were seen for a single study visit containing a structured history and physical examination. Medical information since disease onset was requested, scrutinized and added to the data obtained during the visit. Disease activity and damage were calculated with SLEDAI-2 K and SLICC Damage Index (SDI). HRQOL was assessed with the SF-36. Outcomes were compared to 40 patients with adult-onset SLE (SLE).

**Results:** Almost all 111 cSLE patients were female (91%) and white (69%). Median age was 33 years, median disease duration was 20 years. A vast majority of patients (87%) used immunosuppressive drugs, of whom 60% still used prednisone. Herewith, disease activity was relatively low (median SLEDAI 4).

Many patients (62%) had developed damage (SDI range 1-8), most commonly in the musculoskeletal (41%) and neuropsychiatric system (33%), and kidneys (23%). SDI scores were similar to the SLE patients. We found that 51% of 45 patients who ever had neuropsychiatric (NP) involvement also had NP damage, and 24% of the 67 patients with nephritis ever had developed renal damage. At (very) young age, 7 cSLE patients had a cerebrovascular accident (median 19 yrs), 5 cSLE patients had a myocardial infarction (median 39 yrs), and 6 cSLE patients had replacement arthroplasty (median 33 yrs).

Compared to the Dutch norm data, HRQOL of cSLE patients was impaired in all but one of the eight SF36-domains. Scores of SLE patients were comparable to those of cSLE patients. Remarkably, mental health scores were similar between patients and Dutch norm data. Patients with and without damage had similar HRQOL scores in all but the physical functioning domain.

**Conclusion:** In this large, mainly white cohort of adults with cSLE, the majority had developed damage at a young age. Not many patients had achieved drug-free remission, and prednisone use was common even after a median disease duration of 20 years. Neuropsychiatric involvement led to neuropsychiatric damage in half of the patients, renal involvement led to renal damage in 1/4 of the patients. Overall, HRQOL was impaired, except for patients’ mental health.

**Disclosure of Interest**

None Declared.

**Table 7 (abstract P189).** Patient characteristics

	cSLE n = 111	SLE n = 40	p*
Female	91% (101/111)	93% (37/40)	
Ethnicity			
White	69% (77/111)	68% (27/40)	
Non White	31% (34/111)	32% (13/40)	
Age at study visit (*median (range))*	33 (18 – 65)	40 (25 – 76)	p = 0.000
Age at diagnosis in yrs (*median (range))*	14 (4 – 17)	28 (18 – 69)	p = 0.000
Disease duration in yrs (*median (range))*	20 (1 – 55)	11 (1 – 34)	p = 0.000
Current SLEDAI-2 K score (*median (range))*	4 (0 – 14)	4 (0 – 10)	
SDI-score (*median (range))*	1 (0 – 8)	1 (0 – 7)	
Patients with SLICC-DI ≥ 1	62% (69/111)	60% (24/40)	p = 0.05
Musculoskeletal	41% (28/69)	46% (11/24)	p = 0.027
Renal	23% (16/69)	8% (2/24)	
Neuropsychiatric	33%(23/69)	13% (3/24)	
Cardiovascular	13% (9/69)	33% (8/24)	
Patients currently using any immunosuppressive drug	87% (97/111)	93% (37/40)	
Current prednisone use	60% (58/97)	59% (22/37)	
Renal involvement (ever)	60% (67/111)	48% (19/40)	
Renal damage on SDI	24% (16/67)	11% (2/19)	n.s.
Neuropsychiatric involvement (ever)	41% (45/111)	30% (12/40)	n.s.
Neuropsychiatric damage on SDI	51% (23/45)	25% (3/12)	
Myocardial infarction	5% (5/111)	3% (1/40)	
Cerebrovascular accident	6% (7/111)	3% (1/40)	

^†^ *p* < 0.05 compared to Dutch norm; *if no *p*-values are given, differences between cSLE and SLE were not statistically significant

### P190 Urinary biomarker production in juvenile lupus nephritis – role of the podocytes

#### Rachael D. Wright^1^, Eve M. Smith^1^, Michael W. Beresford^1, 2^

##### ^1^Institute of Translational Medicine, University of Liverpool, Liverpool, UK; ^2^Department of Paediatric Rheumatology, Alder Hey Children's NHS Foundation Trust, Liverpool, UK

###### **Presenting author:** Rachael D. Wright

**Introduction:** Lupus nephritis (LN) is a severe manifestation of juvenile-onset systemic lupus erythematosus (JSLE); it occurs in up to 80% of patients and can lead to end-stage renal disease (ESRD) in 10-15% requiring dialysis or transplantation. LN is relapsing-remitting in character and each flare increases the risk of ESRD. LN is caused by the binding of autoantibodies present in the circulation to antigens expressed on native kidney cells leading to an inflammatory response.

Previous data have identified urinary biomarkers (α1-acid glycoprotein (AGP), caeruloplasmin (CP), transferrin (Tf), lipocalin-type prostaglandin D2 synthase (L-PGDS) and vascular cell adhesion molecule (VCAM)-1) in JSLE patients that indicate when a flare of LN is occurring without the need for an invasive biopsy. A deeper understanding of the pathways leading to biomarker release in the kidney and the effects these have on native kidney cells may identify new kidney specific targets for therapy.

Podocytes are specialised epithelial cells of the glomerulus that play important roles in selective filtration. They constitutively express toll-like receptors and the expression of receptors for TNF-α increase following inflammatory activation indicating they are potentially able to respond to cytokine stimulation.

**Objectives:** This study aimed to determine the role of podocytes in the production of urinary biomarkers following exposure to pro-inflammatory cytokines, in particular those known to be involved in JSLE, or urine/serum from LN patients.

**Methods:** Conditionally immortalised human podocytes (n = 3/group) were cultured at 33 °C until 70% confluent and then thermoswitched to 37 °C for 10-14 days for differentiation to occur; these were then incubated with TNF-α, IL-1β, IFN-α, IFN-γ (10 ng/mL) or LPS (1 μg/mL) for 48 hours and RNA was collected for analysis. Podocytes were also cultured with 10% urine (n = 5/group) or 10% serum (n = 8-9/group) from BILAG defined inactive and active LN patients, and age- and sex-matched healthy controls for 48 hours and RNA collected. Samples were analysed for a modulation in the genes involved in biomarker production by qRT-PCR, normalised to the geometric mean of housekeeping genes – β-actin, TBP and YWHAZ.

**Results:** Increased mRNA expression for CP was seen in response to IFN-α (0.593 ± 0.227; p = 0.05), a cytokine known to be increased in JSLE, compared to healthy controls (0.019 ± 0.02); no significant changes in mRNA expression were noted of the other biomarkers were present following stimulation with the other cytokines. Podocytes treated with urine from all groups’ demonstrated markedly increased cell death compared to untreated podocytes. Although initially attributed to the acidity of the urine, increased cell death was still noted despite titration as low as 1%. Expression of mRNA for all biomarkers were unchanged following treatment with 10% sera from any of the patient groups.

**Conclusion:** These data demonstrate that podocytes play a role in the production of CP following treatment with pro-inflammatory stimuli but this cannot be replicated using patient sera. It can therefore be hypothesised that the role played by podocytes in LN is not in biomarker production but perhaps loss of the podocytes resulting in decreased barrier function and thus passive loss of biomarkers into urine. Further work is required into the role that podocytes do play and into the roles played by other native kidney cells in urinary biomarker production in JSLE.

**Disclosure of Interest**

None Declared.

### P191 Neonatal outcomes of pregnancies complicated by systemic lupus erythematosus

#### Reem Abdawani^1^, Laila Al Shaqshi^2^, Ibrahim Al Zakwani^3^

##### ^1^Child Health, Sultan Qaboos University Hospital, Muscat, Oman; ^2^Child Health, OMSB, Muscat, Oman; ^3^Pharmacy, Sultan Qaboos University, Muscat, Oman

###### **Presenting author:** Reem Abdawani

**Introduction:** SLE predominantly affects women of child bearing age. The effect of SLE on pregnancy and pregnancy on SLE remains controversial. Although most studies on the incidence and prevalence of SLE have been performed using Caucasian cohorts, it appears that individual race and ethnicity may exhibit differences on disease.

**Objectives:** To determine maternal and neonatal outcomes in pregnancies complicated by SLE compared to those with normal pregnancies in Arab women from Sultanate of Oman. To analyze the effect of clinical and serological variables of SLE on pregnancy outcome and neonatal morbidity.

**Methods:** A retrospective analysis of 147 pregnant mothers with their corresponding infants was conducted in SQUH. 56 pregnancies (38%) in SLE mothers were compared to 91 (62%) pregnancies in healthy control mothers. The extracted data include demographic characteristics, lupus disease activity at onset of pregnancy, disease flares, clinical manifestation, autoantibody profile, medications, obstetric status and complications. In addition, data collected included neonatal outcomes including gestational age, gender, birth weight and Apgar score which was compared to the control group.

**Results:** The mean age of the SLE group and control group were comparable 29 ± 5 years versus 31 ± 5 years. The number of mothers who were nulliparous and grand multiparous were also comparable in the two groups. However, SLE mothers were more likely associated with gestational diabetes (28% vs. 10%; p = 0.004), polyhydramnios (7.1% vs. 0; p = 0.010) and have pre-term labor (29% vs. 1.1%; p < 0.001). The SLE mothers were also more likely to be associated with a worse previous obstetrical history including previous pre-term labor (8.9% vs. 1.1%; p = 0.030) abortions (43% vs. 15%; p < 0.001) and still birth/intrauterine fetal deaths (7.1% vs. 0; p = 0.010).

Among the pregnancies, the male female ratio was 1:1.14 which was not significantly different in the two study cohorts. However, infants born to SLE mothers compared to normal health controls, had higher incidence of pre-term births 29% vs 1.1% (P value >0.001), lower mean birth weight 2.69 ± 0.65 versus 3.00 ± 0.29 kg (p < 0.001) and higher incidence of intrauterine growth retardation (birth weight <2500gm) 32% versus 1.1% (P value <0.001) respectively. However, there was not difference in low Apgar scores at birth in both cohorts.

The most common clinical feature includes musculoskeletal (48%), cutaneous (38%), hematological (25%) followed by lupus nephritis (18%). The autoantibody profile in the SLE mothers included positive ANA (95%) followed by dsDNA (52%), antiphospholipid antibodies (33%), anti SSA antibody (36%) and anti SSB antibody (8%). The treatment consisted of hydroxychloroquine (n = 41; 73%), prednisolone (n = 38; 68%) and azathioprine (n = 17; 30%). Despite a high percentage of SLE mothers being on steroids during pregnancy, the majority were on low dose prednisolone 5-10 mg (85%). Up to 80% of SLE mothers were in clinical remission or with mild disease activity, SLEDAI score (0-5) at time of pregnancy and only 19% had disease flare during pregnancy.

The clinical and laboratory features of infants born to SLE include liver (25%), hematological (11%)and cutanous (3.6%) Of note, none of the neonates developed cardiac manifestation despite 32% and 7% of babies had circulating anti SSA (Ro) antibody and anti SSB (La) antibodies, which is typically associated with congenital heart block. The mean duration of positive circulating maternal antibody profile in neonates is 6.6 ± 2.9 months with range 2-15 months.

**Conclusion:** Arab SLE mothers from Sultanate of Oman were associated with worse maternal outcomes when compared to normal pregnant mothers. This result is comparable to other SLE pregnancy outcomes from around the world.

**Disclosure of Interest**

None Declared.

### P192 Autoimmune hemolytic anemia in systemic lupus erythematosus at diagnosis: distinct features in 336 pediatric and 1,830 adult patients

#### Natali W. Gormezano^1^, David Kern^1^, Oriany L. Pereira^1^, Gladys C. C. Esteves^1^, Adriana M. Sallum^2^, Nadia E. Aikawa^1^, Rosa M. Pereira^1^, Clovis A. Silva^1, 2^, Eloisa Bonfa^1^

##### ^1^Pediatric Rheumatology Division, Faculdade de Medicina da Universidade de São Paulo, São Paulo, SP, Brazil; ^2^Pediatric Rheumatology Unit, Faculdade de Medicina da Universidade de São Paulo, São Paulo, SP, Brazil

###### **Presenting author:** Adriana M. Sallum

**Introduction:** Autoimmune hemolytic anemia (AIHA) is an uncommon autoimmune disorder characterized by autoantibodies targeting red blood cells and has been described in 3-20% of childhood-onset systemic lupus erythematosus (cSLE) and in 4-10% of adult-onset SLE. However, no large study evaluated AIHA in both populations.

**Objectives:** The objective of the present study was to determine the overall prevalence of AIHA, and to compare clinical and laboratorial features in a large population of children and adult lupus patients at diagnosis.

**Methods:** This retrospective study evaluated medical charts of 336 cSLE and 1,830 aSLE patients (ACR criteria) followed in the same tertiary hospital. Demographic data, arthritis characteristics, clinical features and disease activity (SLEDAI-2 K) were recorded. AIHA was defined according to the presence of anemia (hemoglobin <10 g/dL) and evidence of hemolysis (reticulocytosis and positive direct antiglobulin test-DAT/Coombs test) at SLE diagnosis. Evans syndrome (ES) was defined by the combination of immune thrombocytopenia (platelet count <100,000/mm^3^) and AIHA.

**Results:** The frequency of AIHA at diagnosis was significantly higher in cSLE patients compared to aSLE [49/336(14%) vs. 49/1830(3%), p = 0.0001], with similar frequency of ES [3/336(0.9%) vs. 10/1830(0.5%), p = 0.438]. Compared to adults, cSLE patients had more often multiple hemorrhagic manifestations (41% vs. 7%, p = 0.041), constitutional involvement (84% vs. 31%, p < 0.001), fever (65% vs. 26%, p < 0.001), weight loss > 2 kg (39% vs. 6%, p < 0.001), hepatomegaly (25% vs. 0%, p < 0.001) and splenomegaly (21% vs. 2%, p = 0.004). Other major organ involvements were common but with similar frequencies in adult and children (p = 0.05). The median of hemoglobin levels was reduced in cSLE *versus* aSLE patients [8.3(2.2-10) vs. 9.5(6.6-10)g/dL, p = 0.002] with a higher frequency of erythrocyte transfusion due to bleeding (24% vs. 5%, p = 0.025). Autoantibody profiles and immunosuppressive treatments were similar in both groups (p > 0.05). Median SLEDAI-2 K was also comparable in adults and children (p = 0.161).

**Conclusion:** We identified that AIHA at SLE diagnosis has distinct features characterized in cSLE by high prevalence and severity of this hematological manifestation and an almost universal association with constitutional symptoms.

**Disclosure of Interest**

None Declared.

### P193 B cell-bound complement activation products in the diagnosis and monitoring of systemic lupus erythematosus

#### Jessica Beckmann^1^, Nora Bartholomä^2^, Nils Venhoff^2^, Philipp Henneke^1^, Ulrich Salzer^2^, Ales Janda^1^

##### ^1^Center for Pediatric and Adolescent Medicine, University Medical Center, Freiburg im Breisgau, Germany; ^2^Rheumatology and Clinical Immunology, University Medical Center, Freiburg im Breisgau, Germany

###### **Presenting author:** Ales Janda

This abstract is not included here as it has already been published.

### P194 Neuropsychiatric manifestations in pediatric-onset systemic lupus erythematosus: experience of a tertiary center

#### Alina Lucica Boteanu^1^, Sandra Garrote Corral^2^, Alberto Sifuentes Giraldo^1^, Mariluz Gámir Gámir^1^, Antonio Zea Mendoza^2^

##### ^1^Rheumatology Pediatric Unit, University Hospital Ramón y Cajal, Madrid, Spain; ^2^Rheumatology, University Hospital Ramón y Cajal, Madrid, Spain

###### **Presenting author:** Alina Lucica Boteanu

**Introduction:** The neuropsychiatric (NP) manifestations are an important cause of morbidity and mortality in patients with pediatric-onset systemic lupus erythematosus (pSLE). The prevalence of these manifestations ranges from 29% to 95% in different series, but data in the spanish pediatric population are scarce.

**Objectives:** To analyze the clinical and immunological features of patients with pSLE and NP followed in a Spanish tertiary center.

**Methods:** We performed a retrospective study of 62 patients with pSLE diagnosed between 1985 and 2015 in our center. The American College of Rheumatology NPSLE case definitions were used to classify the manifestations. The demographic, clinical and immunological data were obtained through review of their medical charts. Continuous variables were analyzed using Student's t-test (or Mann–Whitney U test if the number was < 10 or the sample did not have a normal distribution) and discrete variables by Pearson's χ2 test (or Fisher's exact test when number in the category was < 5).

**Results:** Twenty eight (45%) developed NP manifestations. The mean age of the patientes with NP manifestations was 13.9 years and female:male ratio was 2.5: 1. These manifestations were presented at the beginning of the disease in 7 cases (25%) and in the first 2 years in 65% of the cases. The most common presentations of NPSLE were the cental manifestation (89,2%) with seizures in 13 cases, headache in 8 cases, mood disorder/depression in 5 cases, psychosis in 4 cases, cerebrovascular disease in 5 cases and aseptic meningitis in 4 cases. There was more than one NP manifestation in 48% of the patients, with an average of 2.4 manifestations/patient. The comparison of patients with and without NPSLE demonstrated significant diffences (p <0.05) in the number of males, titers of anti-DNA antibodies, positivity for anti-β2 -glicoprotein I (β2GPI) and consumption of complement (C3, C4).50% of the patients with NP manifestations had cyioglobuline positive. There were 2 cases of mortality in NPSLE (7.1%) during the follow-up period, one as a result of infection of the central nervous system and another due to sepsis associated with intestinal thrombosis.

**Conclusion:** In our series 45% of the patients had NPSLE manifestations and they frequently occured early during the course of the disease. 100% of the male patients presented NP manifestations during the course of the disease. The clinical spectrum of NPSLE was wide in our cases and most of them had more than one manifestation. Patients with NPSLE showed a high disease activity as measured by levels of anti DNA antibodies and hypocomplementemia. Although NPSLE has been associated with positive antiphospholipid antibodies in other series, specifically anticardiolipin antibodies and lupus anticoagulant, we only found significant association with anti-β2GPI IgG antibodies

**Disclosure of Interest**

None Declared.

### P195 Pulmonary hypertension in patients with juvenile lupus erythematosus

#### Amra Adrovic^1^, Reyhan Dedeoglu^2^, Sezgin Sahin^2^, Kenan Barut^1^, Aida Koka^2^, Funda Oztunc^2^, Ozgur Kasapcopur^1^

##### ^1^Pediatric Rheumatology, Istanbul University, Cerrahpasa Medical School, Istanbul, Turkey; ^2^Pediatric Cardiology, Istanbul University, Cerrahpasa Medical School, Istanbul, Turkey

###### **Presenting author:** Amra Adrovic

**Introduction:** Juvenile systemic lupus erythematosus (jSLE) is a chronic multisystemic autoimmune disease characterized with variable clinical course. Vital organ involvement is the most important morbidity and mortality factor of the disease. Pulmonary hypertension (PH) is defined as a mean pulmonary artery pressure (PAP) ≥ 25 mmHg at rest or a right ventricular systolic pressure (RVSP) > 40 mmHg. Non-specific clinical features and insignificant findings are the main reasons of the delayed diagnosis in patients with PH. Timely echocardiographic (ECHO) examination enables early diagnosis of the disease. Pulmonary hypertension has been reported to be associated with poor prognosis in several studies among adults with SLE. The prevalence of PH in adult SLE patients is estimated to be 1.8% to 14%. Studies on pulmonary hypertension among patients with jSLE are spare.

**Objectives:** The aim of this study is to explore the right ventricle functions and to determinate the frequency of pulmonary hypertension in patients with jSLE, using non-invasive methods (Pulsed wave and tissue Doppler ECHO).

**Methods:** Patients with diagnosis of jSLE followed up at our department were included in the study. Pulse wave and tissue Doppler ECHO was performed to all included patients and to healthy controls. Measurements obtained by ECHO include: peak velocity of the tricuspid insufficiency (TRVmax), end diastolic velocity of pulmonary insufficiency (PIV), tricuspid annular plane systolic excursion (TAPSE), right ventricle diastolic function measurement (Lat E, A, E’ wave, E/E’ ratio). Bernoulli equation (4 × TRVmax2) was used to calculate the estimated PAP.

**Results:** A total of 38 jSLE patients and 40 healthy controls were included in the study. Mean age of patients was 16.0 ± 2.59 years, mean age at diagnosis was 10,63 ± 3,51 years and mean disease duration was 57.02 ± 33.6 months. Mean age of control group was 15 ± 3.49 years. Echocardiographic measurements of patients and healthy controls are shown in Table 8. TRV max and PIV were significantly higher in jSLE patients comparing to healthy controls with p < 0.05 and p < 0.001, respectively. Tissue Doppler ECO measurements of right cardiac diastolic functions (Lat E, E’ wave and E/E’ ratio) were significantly different in jSLE patients, comparing to healthy controls.

**Conclusion:** This study confirms that pulmonary hypertension is uncommon among patients with jSLE. However, patients with jSLE have a compromised right cardiac contractile functions and higher pulmonary artery pressure comparing to healthy controls. These results point out the importance of echocardiographic examination in patients with jSLE, regarding right cardiac functions and pulmonary hypertension.

**Disclosure of Interest**

None Declared

**Table 8 (abstract P195).** Echocardiographic measurements in jSLE patients and healthy controls

Pulsed wave and tissue Doppler echocardiographic measurements	jSLE patients N = 38	Healthy controls N = 40	MW	**p**
Lat E wave (cm/s)	13,441 ± 1,463	16,767 ± 2,605	164,000	0,000
Lat A wave (cm/s)	7,963 ± 2,039	8,667 ± 1,857	416,000	0,079
Lat S wave(cm/s)	10,471 ± 1,986	11,340 ± 2,256	417,000	0,112
Lat E’wave (cm/s)	6,772 ± 1,297	5,875 ± 0,720	272,000	**0,002**
Lat E/E’ ratio	5,227 ± 1,192	5,841 ± 0,677	260,000	**0,001**
TRV max (m/s)	2,340 ± 0,277	2,044 ± 0,411	299,000	**0,011**
PIV (m/s)	1,469 ± 0,295	1,214 ± 0,128	250,000	**0,000**
TAPSE (mm)	23,018 ± 3,422	21,800 ± 1,701	269,000	0,214

### P196 Associated features to damage in 80 juvenile systemic lupus erythematosus patients from a tertiary pediatric centre

#### Ana Luisa Rodriguez-Lozano^1^, Francisco Rivas-Larrauri^1^, Silvestre García de la Puente^2^

##### ^1^Immunology, Instituto Nacional de Pediatria, Mexico City, Mexico; ^2^Research Methodology, Instituto Nacional de Pediatria, Mexico City, Mexico

###### **Presenting author:** Ana Luisa Rodriguez-Lozano

**Introduction:** Patients with juvenile systemic lupus erythematosus (JSLE) have more aggressive disease^1^ in addition to the improvement on survival in the last decades^2,3^, have led patients to be exposed to the disease for a longer time and to endangering them to develop damage.

**Objectives:** To assess damage in a large cohort of patients, and to identify the main features associated with the development of damage.

**Methods:** An ambispectic cohort was designed, patients aged ≤16 years old were followed up at least for two years. Every 3 to 6 months all patients were assessed for disease activity (DA) with SLEDAI-MEX^4^, and anually for damage with the Pediatric Systemic Lupus International Collaborating Clinics/American College of Rheumatology (SDI)^5^. Demographic features are reported as median (min-max). A bivariate analysis was performed for Damage ≥2, (dichotomus variable) with U Mann Whitney and Pearson chi square, and for Damage (numeric variable) with Pearson correlation. Statistical analysis was accomplished with SPSS 16.0, Chicago IL. Ethics and Research Committee have approved the protocol.

**Results:** Eigthy five out of 97 identified JSLE patients were included, misclassification issues were found in 5, leading to 80 patients analyzed, 30% correspond to male patients, F:M ratio 2.3:1. At diagnosis: age had a median of 11.87 yr, min-max (2.18 - 16.3 yr), and SLEDAI-MEX 12 (2 - 29). The main features at diagnosis are: nefritis 64%, hemolytic anemia 53%, lymphopenia 51%, hematuria 51%,proteinuria 48%, malar rash 46%, oral ulceration 35%, fatigue 32%, fotosensitivity 30%, thrombocytopenia 28%, fever 27% and neurolupus 20%. After at least two years of follow up the disease duration was 3.91 yr (2 - 9.4) and SDI 2 (0 - 6). Regarding the distribution of damage, 62% of patients have damage ≥2. Bivariate Analysis: for Damage, a significant Pearson correlation with (p ≤ .005) proteinuria and SLEDAI-MEX at the diagnosis time was found. Table 9 shows the variables analized for Damage ≥ 2. The highly associated variables with Damage ≥2 are SLEDAI-MEX >6, hemolytic anemia and nephritis, and to a lesser extent, gender, specifically being male. We did not find any association with age group, the presence of thrombocytopenia, lymphopenia nor with the presence of anticardiolipins antibodies.

It has been published before^6^ that cumulative disease activity over time, cumulative steroid dose, acute thrombocytopenia and presence of antiphospholipid (APL) antibodies are the main risk factors for development of damage, however, in the clinical setting it is not helpful to have to wait several months in order to calculate the first two variables, plus in our population we did not find any association with acute thrombocytopenia nor with the presence of ALP antibodies.

**Conclusion:** The presence of SLEDAI-MEX >6, proteinuria (>0.5 g/d) and hemolytic anemia, identified at the diagnosis time, can be important factors for the development of damage in patients with JSLE. Pediatric rheumatologists must be aware that the presence of those factors should lead to a close follow up in order to reduce the likelihood of damage.

**Trial registration identifying number:**

**References**

1. Tucker LB, Uribe AG, Fernández M, Vilá LM, McGwin G, Apte M, Fessler BJ, et al. Adolescent onset of lupus results in more aggressive disease and worse outcomes: results of a nested matched case - control study within LUMINA, a multiethnic US cohort (LUMINA LVII). Lupus 2008;17:314-22.

2. Lee PY, Yeh KW, Yao TC, Lee WI, Lin YJ, Huang JL.The outcome of patients with renal involvement in pediatric-onset systemic lupus erythematosus--a 20-year experience in Asia. Lupus. 2013;22:1534-40.

3. Blancas-Galicia L, Guevara-Cruz M, Berrón-Pérez R, Berrón-Ruiz L, Gutiérrez-Castrellón P, Espinosa-Rosales FJ. Survival of Mexican patients with paediatric-onset systemic lupus erythematosus and abnormal electroencephalogram. Allergol Immunopathol. 2013;41:108-13.

4. Uribe GA, Vilá ML, McGwin Jr G, Sánchez ML, et al. The systemic lupus activity measurement-revised, the Mexican systemic lupus erythematosus disease activity index (SLEDAI), and a modified SLEDAI-2 K are adequate instruments to measure disease activity in systemic lupus erythematosus. J Rheumatol 2004; 31: 1934-40

5. Gutierrez –Suárez R, Ruperto N, Gastaldi R, et al., A proposal for a pediatric version of the Systemic Lupus International Collaborating Clinics/American College of Rheumatology Damage Index based on the analysis of 1015 patients with Juvenile-Onset Systemic Lupus Erythematosus. Arthritis and Rheumatism 2006; 54: 2989-96

6. Brunner H, Silverman E, To T, Bombardier C, Feldman B. Risk factors for damage in childhood onset systemic lupus erythematosus. Arthritis and Rheumatism 2002; 42: 436-444.

**Disclosure of Interest**

None Declared.

**Table 9 (abstract P196).** Variables tested for association with Damage ≥2

Variable	Damage ≥ 2	Pearson* p Value
Gender:		
Fem / Male	51.8% **/** 54.2%	1.281 .038
Adolescent		
No / Yes	57.1% / 47.4%	.764 .382
SLEDAI-MEX		
< 6 / >6	20% **/** 63.3%	11.295 .001
Hemolytic anemia		
No / Yes	36.8% / 66.7%	7.116 .008
Trombocytopenia		
No / Yes	54.1% / 47.4%	.263 .608
APL		
Neg / Pos	42.9% / 61.0%	2.486 .115

*Pearson chi square

### P197 Can individual exposure to air pollution trigger disease activity and aggravate nephritis in childhood-onset systemic lupus erythematosus patients?

#### Andressa G. F. Alves^1^, Maria F. D. A. Giacomin^1^, Juliana Farhat^2^, Alfésio L. F. Braga^3^, Adriana M. E. Sallum^4^, Lúcia M. D. A. Campos^4^, Luiz A. A. Pereira^3^, Ana J. D. F. C. Lichtenfels^2^, Clóvis A. Silva^4^, Sylvia C. L. Farhat^4^

##### ^1^Pediatria, Instituto da Criança FMUSP, São Paulo, Brazil; ^2^Laboratório experimental de poluição, São Paulo, Brazil; ^3^USP, São Paulo, Brazil, ^4^Instituto da Criança FMUSP, São Paulo, Brazil

###### **Presenting author:** Andressa G. F. Alves

**Introduction:** Exposure to outdoor pollutants has been associated with an increase in hospitalizations due to the activation of our pediatric rheumatic diseases and with an elevated risk of disease activity in our childhood-onset systemic lupus erythematosus (cSLE). However individual real-time exposure, evaluating indoor and outdoor pollutants, were not studied in adult and cSLE populations.

**Objectives:** To investigate the association between daily individual real-time exposure to air pollutants and SLEDAI-2 K parameters of disease activity in cSLE patients.

**Methods:** A longitudinal and observational panel study (repeated measures from April 2013 to June 2015) was carried out in 108 consecutive appointments with 9 cSLE patients without rhinitis and chronic pulmonary diseases. Over four consecutive weeks, daily individual measures of nitrogen dioxide (NO_2_) by a passive sampler during seven days, fine particulate matter (PM_2,5_) by a real-time laser photometer, ambient temperature and humidity were obtained. This cycle was repeated every 2.5 months along one year, and disease activity parameters were collected weekly. Generalized estimation equation models were used, considering the fixed effects of repetitive measures, to assess the impact of these pollutants on the risk of increased serum anti-dsDNA, moderate/severe disease activity (SLEDAI-2 K ≥ 8), presence of nephritis and altered urine casts, and decrease in serum levels of complement. The models were adjusted for inflammatory indicators, body mass index, infections, medication, and weather variables.

**Results:** For an interquartile range (IQR) increase of 18.12 μg/m^3^ in PM_2.5_ daily concentration, an increase was observed in the risk of: nephritis on the 3rd, 4th, 10th and 14th days after exposure; haematuria only on the 4th day; leukocyturia from 3-7days after exposure; increased anti-dsDNA levels on the 4th, 10th and 11th days after exposure; SLEDAI-2 K ≥ 8 on the 4th and 11th days after exposure. Moreover, for an IQR increase in PM_2.5_ daily concentration, an increase was observed in the 24 h-urine protein on the same day of exposure and from the 6th to the 10th day as well as a decrease in C3 serum levels on the same day and on the 4th and on the 6th day after exposure. An IQR increase in PM_2.5_ 7-day moving average (from lag0 to lag6) was associated with increased risks for: leukocyturia (3.4; 95%IC 2.6-4.3); increased anti-dsDNA levels (3.1; 95%CI 2.1-4.0) and SLEDAI ≥ 8 (1.5 95%CI 1.1-1.8). An increase of 36.3 mg (95%IC 20.2-52.3) in 24 h- urine protein was also observed. An IQR increase (63.12 μg/m^3^) in NO_2_ 7-day cumulative effect was associated with an increased risk for SLEDAI ≥ 8 (2.6; 95%CI 1.9-3.3) and with a 6.4 mg/dl (95%CI; -10.9 -1.9) decrease in C3 serum levels.

**Conclusion:** This was the first prospective study suggesting that exposure to outdoor/indoor air pollution may trigger disease activity and may aggravate nephritis in c-SLE patients.

**References**

1. Vidotto JP, Pereira LAA, Braga ALF et al. Atmospheric pollution: influence on hospital admission in pediatric rheumatic disease. Lupus. 2012;21:526-33.

2. Fernandes EC, Silva CA, Braga ALF, Sallum AM, Campos LM, Farhat SC. Exposure to air pollutants increased disease activity in childhood-nset systemic lupus erythematosus patients. Arthritis Care Res. 2015; 67:1609-14.

**Disclosure of Interest**

None Declared.

### P198 Clinical characteristics and long-term outcomes of systemic lupus erythematosus in children

#### Banu Acar^1^, Z. Birsin Ozcakar^2^, Nilgün Çakar^1^, Nermin Uncu^1^, Gökçe Gür^3^, Semanur Özdel^2^, Fatoş Yalçınkaya^2^

##### ^1^Department of Pediatric Rheumatology, Ankara Child Health, Hematology, Oncology Education and Research Hospital, Ankara, Turkey; ^2^Department of Pediatric Rheumatology, Ankara University School of Medicine, Ankara, Turkey; ^3^Department of Pediatric Nephrology, Ankara Child Health, Hematology, Oncology Education and Research Hospital, Ankara, Turkey

###### **Presenting author:** Banu Acar

**Introduction:** Systemic lupus erythematosus (SLE) is a systemic multisystem autoimmune disease characterized by the presence of autoantibodies and multiorgan system involvement.

**Objectives:** The aim of this study was to describe the presenting clinical manifestations, clinical course and prognosis of SLE in children.

**Methods:** We performed a retrospective review of medical records of SLE patients. Between 1995 and 2016, 52 children with SLE were evaluated. Medical records were systematically reviewed, including demographic data, clinical features, and laboratory findings.

**Results:** The study consisted of 52 patients with childhood SLE. There were 44 female and 8 male patients. The mean age of onset of the disease was 12,2 +/- 2,7 years (range 4-17). The mean duration of follow-up was 5,5 years (range 1-14). Table 10 summarizes the frequency of the clinical features of disease noticed in our patients. The most common clinical feature was arthritis (65,4%), followed by constitutional symptoms (48,1%), malar rash (36,5%), weight loss (21,2%), photosensitivity (15,4%), oral ulcers (9,6%), and alopecia (7,7%).

Renal involvement accounted 3/4 of the patients (75%). Hematuria and proteinuria were most frequent presenting findings (38% and 36,5% respectively). 13 patients had nephrotic syndrome. Renal biopsy was performed in 40 patients. According to WHO classification: 1 patient had class V nephritis, 16 had class IV, 6 had class III, 15 had class II and 2 had class V nephritis. All patients with class IV nephritis were treated with pulse metilyprednisolon and intravenous cyclophosphamide as an induction therapy then shifted to either azathioprine or mycofenolate mofetil. Neurological involvement was seen in eleven patients (21,2%); 5 patients had headache, 3 had acute confusional state, 3 had chorea, 1 had seizure, and 1 had hemiparesis. Hematological abnormalities were detected in 35 patients (67,3%) at the time of diagnosis. Anemia was the most common hematological manifestations at onset (73%). Hemolytic anemia with positive Coomb’s test was detected in 20 patients (38,5%). Sixteen patients had leucopenia (32%), and 19 patients (38%) had thrombocytopenia. Antiphospholipid syndrome developed in three patients. ANA was detected in 47 patients (90,4%) and 80,8% of them had elevated anti-dsDNA titer. Complements (C3 and C4) were low in 36 patients (72%).

None of our patients died. At the last follow up none of them had renal failure but, proteinuria persisted in 8 of them (15,4%). All neurological findings of our patients are in remission apart from sequelae.

**Conclusion:** Clinical outcome was favorable in our study. Renal involvement is common but progression to ESKD, at least in the short term, is rare.

**Disclosure of Interest**

None Declared

**Table 10 (abstract P198).** Clinical features of systemic lupus erythematosus patients at presentation

Characteristics	n (%)
Fever, fatigue	25 (48,1%)
Rash	19 (36,5%)
Weight loss	11 (21,2%)
Alopecia	4 (7,7%)
Arthritis-arthralgia	34 (65,4%)
Renal involvement	39 (75%)
Nervous system involvement	11 (21,2%)
Hematological involvement	35 (67,3%)
Cardiovascular involvement	11 (21,2%)

### P199 Two cases of paediatric systemic lupus erythematosus with severe large-vessel central nervous system vasculitis

#### Christiaan Scott^1^, Nicky Brice^1^, Peter Nourse^2^, Laura Lewandowski^3^

##### ^1^Paediatric Rheumatology, University of Cape Town, Cape Town, South Africa; ^2^Paediatric Nephrology, University of Cape Town, Cape Town, South Africa; ^3^Systemic Autoimmunity Branch, National Institute of Arthritis and Musculoskeletal and Skin Diseases, National Institutes of Health, Bethesda, USA

###### **Presenting author:** Christiaan Scott

**Introduction:** Cerebrovascular disease is a recognised manifestation of neuropsychiatric SLE in children; however angiogram- positive large vessel vasculitis is uncommon.

**Objectives:** To describe two cases of paediatric systemic lupus erythematousus (SLE) with extensive large- vessel central nervous system (CNS) vasculitis and their response to cyclophosphamide therapy.

**Methods:** We reviewed and summarised two rare cases of large- vessel CNS vasculitis in 2 patients with known SLE and review existing literature on the various forms of CNS vasculitis in paediatric SLE patients.

**Results:** An 11 year old girl was diagnosed with SLE in May 2014. Initial manifestations of polyarthritis and discoid skin lesions showed good response to prednisone, chloroquine and azathioprine. She represented in April 2015 after a six month period of medication non-compliance with a flare of polyarthritis and frequent headaches refractory to simple analgesia. A single-photon emission computerized tomography (SPECT) scan of her brain showed diffuse perfusion abnormalities, and MRI and MR angiogram revealed extensive vasculopathy with marked narrowing of the right internal carotid artery and bilateral carotid siphons, but no features of ischaemia. A lumbar puncture demonstrated a raised opening pressure of 35 cm H20, but a normal cell count and protein. Following pulse dose of IV methylprednisolone and six months of IV cyclophosphamide at 750 mg/m2, her headaches resolved and repeat imaging showed significant interval improvement. She is currently on myfortec maintenance therapy.

A 16 year old boy was diagnosed with SLE in 2009 after presenting with a malar rash, arthritis and bilateral testicular ‘torsion’. Histology of a left orchidectomy showed vasculitis and haemorrhagic infarction. He was subsequently lost to follow-up and represented in December 2014 with isolated polyarthritis that responded well to prednisone, methotrexate and chloroquine. One year later he was seen at routine follow-up with a month long history of a refractory frontal headache and loss of vision in his left eye. He was noted to be severely hypertensive. Ophthalmology exam confirmed a retro-bulbar optic neuritis which resolved completely after treatment with IV methylprednisolone. His hypertension proved very difficult to control, requiring multiple agents. Exhaustive renal investigations for a cause were negative. Cardiac evaluation with ECG and ECHO were normal. However his brain MRI and MR angiogram showed extensive vasculitis, with marked attenuation of all the major cerebral vessels, but no evidence of ischaemia. A lumbar puncture demonstrated normal opening pressure and CSF analysis was normal. He is currently receiving monthly IV cyclophosphamide infusions at 750 mg/m2 and his headaches have resolved. The hypertension is presumed to be of central origin.

**Conclusion:** Angiogram-positive large vessel vasculitis is a rare form of neuropsychiatric SLE in children, however it should always be considered in cases of severe recurrent headache with or without accompanying neurological signs.

**Disclosure of Interest**

None Declared.

### P200 Comparison between SLICC and ACR 1997 classification criteria in patients with juvenile systemic lupus erythematosus evaluated in a pediatric rheumatology service in Bogotá, Bolombia

#### Christine Arango, Angela C. Mosquera, Clara Malagon

##### Pediatric Rheumatology, El Bosque University, Bogota, Colombia

###### **Presenting author:** Christine Arango

**Introduction:** Systemic lupus erythematosus (SLE) is a multisystemic disease. Around 20% of cases occur in the pediatric population. Over the years various proposals of classification criteria have been published. In 1971 Cohen et al published the preliminary criteria, which were later on reviewed and updated in 1982 and 1997 by the American College of Rheumatology (ACR). In 1994 Ferraz et al evaluated the 1982 ACR criteria in the pediatric population with a sensitivity of 96,1% and a specificity of 100%. Recently the SLICC group published a new criteria proposal with a sensitivity of 97% (vs 83% ACR 1997) and a specificity of 84% (vs 96% ACR 1997) in the adult population. The comparison between both criteria in the pediatric population in a multicenter study found a higher sensitivity (98,7% vs 76,7% ACR 1997) with a lower specificity (85,3% vs 93,4% ACR 1997). At the moment the SLICC criteria performance in the Colombian population is not known.

**Objectives:** Evaluate the sensitivity and specificity of SLICC criteria in a pediatric population with juvenile systemic lupus erythematosus (jSLE) in Bogotá, Colombia.

**Methods:** Diagnostic test study. Retrospective evaluation of features present in the first month of diagnosis in patients with jSLE seen in a pediatric rheumatology service in Bogotá, Colombia between May 2007 and March 2016. Controls were included with the following diagnosis: juvenile idiopathic arthritis (n = 24), dermatomyositis (n = 7), autoimmune hematologic disorders (n = 6), antiphospholipid syndrome (n = 6), systemic vasculitis (n = 5), overlapping syndromes (n = 4), undifferentiated connective tissue disease (n = 2) and autoimmune hepatitis (n = 1).

**Results:** n = 110. 55 cases and 55 controls. Mean age at onset in cases was 12,8 years (7-15 years) and in controls was 11,1 years (2-16 years). Sex distribution in cases was 83,6% girls, F:M ratio 5,4:1 and in controls 65,5% girls, F:M 1,8:1. The most prevalent features in cases were lymphopenia < 1500 in 54,5% (vs 10,9%, p = 0,000), arthritis in 47,2% (vs 52,7%, p = 0,352), proteinuria in 41,8% (vs 7,2%, p = 0,000), lymphopenia < 1000 in 36,3% (vs 3,6%, p = 0,000 and malar rash in 34,5% (vs 9%, p = 0,001). Antinuclear antibodies were positive in 94,4% (vs 34,5%, p = 0,000), anti DNA antibodies in 57,4% (vs 3,7%, p = 0,000), IgM anticardiolipin in 29% (vs 12,7%, p = 0,03), anti Smith in 29% (vs 0%, p = 0,000), IgG anticardiolipin in 27,2% (vs 9%, p = 0,012), lupus anticoagulant in 23,6% (vs 10,9%, p = 0,064) and false positive syphilis test in 16,3% (vs 0%, p = 0,001). There was hypocomplementemia of C3 and C4 in 80% and 69% of cases respectively (vs 12,7% and 15,4%, p = 0,000). During the first month of diagnosis 78,1% of cases completed ACR criteria and 89,1% completed SLICC criteria. The sensitivity of SLICC criteria was higher (89% vs 78%) with a lower specificity (87% vs 96%).

**Conclusion:** In this group of pediatric patients the sensitivity of SLICC criteria during the first month of diagnosis was higher and the specificity was lower than ACR 1997. These findings correlate with what has been reported in other pediatric groups and in adults. The severity of the disease in children is higher and the prompt diagnosis allows an early treatment. Knowledge about and implementation of these new criteria should be promoted.

**Disclosure of Interest**

None Declared.

### P201 A multicenter cohort study of initial digital vasculitis in childhood-onset systemic lupus erythematosus: a rare and worrying condition

#### Ana P. Sakamoto^1^, Clovis A. Silva^2^, Marco F. C. D. Silva^2^, Ananadreia S. Lopes^1^, Gleice C. S. Russo^1^, Adriana E. M. Sallum^2^, Katia Kozu^2^, Eloisa Bonfá^3^, Claudia Saad-Magalhães^4^, Rosa M. R. Pereira^3^, Claudio A. Len^1^, Maria T. Terreri^1^

##### ^1^Pediatric Rheumathology Unit, Depatment of Pediatrics, Universidade Federal de São Paulo, São Paulo, Brazil; ^2^Pediatric Rheumathology Unit, Faculdade de Medicina da Universidade de São Paulo, São Paulo, Brazil; ^3^Division of Rheumatology, Faculdade de Medicina da Universidade de São Paulo, São Paulo, Brazil; ^4^Pediatric Rheumatology Division, São Paulo State University (UNESP) – Faculdade de Medicina de Botucatu, Botucatu, Brazil

###### **Presenting author:** Clovis A. Silva

**Introduction:** Childhood-onset Systemic Lupus Erythematosus (cSLE) is characterized by more severe and cumulative acute organ and system involvement comparing to adult SLE. Mucocutaneous involvement is one of the most common manifestations.

Data on cSLE patients are limited to case reports and small series. There are no published data characterizing igital vasculitis (DV) in a large population of childhood lupus patients.

**Objectives:** To assess DV as an initial manifestation in a large multicenter study, evaluating the possible association with demographic and clinical features, laboratorial exams, treatment and outcomes in cSLE onset.

**Methods:** Multicenter cohort study including 852 cSLE patients (ACR criteria) followed in ten Pediatric Rheumatology centers in São Paulo State, Brazil.

**Results:** DV was observed in 25/852 (3%) cSLE patients. Periungual hemorrhage was diagnosed in 12 (48%), periungual infarction in 7 (28%), tip finger ulceration in 4 (16%), painful nodules in 1 (4%) and gangrene in 1 (4%). A poor outcome, with digital resorption, occurred in 5 (20%). Comparison of patients with and without DV revealed higher frequency of malar rash (80% vs. 53%,p = 0.008), discoid rash (16% vs. 4%,p = 0.017), photosensitivity (76% vs. 45%,p = 0.002) and other cutaneous vasculitides (80% vs. 19%,p < 0.0001), whereas the frequency of overall constitutional features (32% vs. 61%,p = 0.003), fever (32% vs. 56%,p = 0.020) and hepatomegaly (4% vs. 23%, p = 0.026) were lower in these patients. Frequency of female gender, severe multi-organ involvement, autoantibodies profile and low complement were alike in both groups (p > 0.05). SLEDAI-2 K median, DV scoring excluded, was significantly lower in patients with DV compared to those without this manifestation [10(0-28) vs. 14(0-58),p = 0.004]. Visceral vasculitis or death were not observed in this cSLE cohort. The frequency of cyclophosphamide use (0% vs. 18%,p = 0.014) was significantly lower in the DV group.

**Conclusion:** In conclusion, our large multicenter study identified clinical DV as one of the rare initial manifestation of active cSLE associated with a mild multisystemic disease, in spite of digital resorption in some of these patients.

**Disclosure of Interest**

None Declared.

### P202 Serum levels of functional mannose binding lectin in patients with pediatric onset

#### Deepti Suri, Siyaram Didel, Amit Rawat, Surjit Singh

##### Department of Pediatrics, PGIMER, Chandigarh, India

###### **Presenting author:** Deepti Suri

**Introduction:** Complement system plays a crucial role in the pathogenesis of SLE. Excessive activation of complement causes tissue injury and SLE occurrence is typically associated with congenital deficiencies of the early components of the classical complement pathway. Mannose-binding lectin (MBL) is a recognition molecule of the lectin pathway of complement activation.It is recognized as one of the important causative factor in the etio-pathogenesis of SLE and MBL-2 gene has evolved as a candidate gene for SLE susceptibility

**Objectives:** The present study was done to assess the functional MBL level (fMBL) in pediatric SLE patients and its co-relation with disease severity and outcome.

**Methods:** Functional MBL (fMBL) level was assessed by ELISA in 28 children with pediatric onset SLE and disease in remission and under follow up at Pediatric Rheumatology Clinic between January 2012 to December 2012.These levels were compared with age and sex matched healthy controls. These levels were correlated with duration of illness, complementary level and anti-dsDNA antibody level. Functional MBL (fMBL) levels were also assessed in parentsand siblings of patients with very low levels.

**Results:** A total of 31 consecutive children in clinical remission were initially evaluated. On computing their SLEDAI scores 3 children were found to have SLEDAI equal or more than 4 and were excluded from the analysis.(12) Functional MBL levels were assessed in 28 patients and an equal number of age and sex-matched normal healthy controls. Female: male ratio was 2.5:1 in both the groups while the mean age in case and controls was 14.75 ± 3.6 years and 15.21 ± 4.16 years respectively. There was no statistically significant difference between the mean fMBL in the cases as well control groups. The mean fMBL level in study subjects was 696.16 ± 408.98 U/ml while it was 579.52 ± 393.87 U/ml among controls. As the data was skewed median fMBL levels were also computed and found to be 901.03 U/ml (range 369.81–1028.06 U/ml) in cases and 576.05 U/ml(193.97–974.62 U/ml) in controls. The levels were higher in patients even in remission when compared to control population though not found to be statistically significant. We defined less than 40 U/ml as very low fMBL levels(4cases,14.4% of total cases), low fMBL level between 40- 100U/ml(2,7.2%), intermediate level 101-1000 U/ml(14,50.4%) and high fMBL levels as level more than 1000 U/ml(8,28.8%). Four out of 28 (14.3%) children were found to have very low fMBL levels. A statistically significant correlation of fMBL levels with the age of these patients at the time of diagnosis was found using Pearson correlation test.

Serum complements (C3, C4) were assessed in all patients. None of the patients had low C3 levels. However, low C4 (≤7.7 mg/dl) were found in 3 (10.71%) children. A negative correlation of fMBL was found with respect to C4 levels in patients, although it was not statistically significant. High levels (>40 IU/ml) were found in 9 children (32.14%). Correlation of fMBL level with respect to Anti-ds DNA Ab levels revealed that fMBL level positively correlated with Anti-ds DNA antibodies level. Higher fMBL levels were found in patients with higher anti-ds DNA Antibody.

**Conclusion:** Large variation exists in fMBL levels in pediatric onset SLE even when in remission.

While very low fMBL levels were found in some children with younger age of onset of

SLE, multisystem involvement and severe course of the disease. Positive correlation of MBL levels with anti-ds DNA titers suggest that its values vary with activity and could

be a potential biomarker of the disease

**Disclosure of Interest**

None Declared.

### P203 Immune status against hepatitis a in newly diagnosed, previously vaccinated JSLE patients

#### Despoina Maritsi^1^, MArgarita Onoufriou^2^, Olga Vougiouka^3^, Maria Tsolia^3^

##### ^1^Pediatrics, P & A Kyriakou Childrens Hospital, Athens, Greece; ^2^Pediatrics, Makarios III Children;s Hospital, Nicosia, Cyprus; ^3^Pediatrics, P & A Kyriakou Children's Hospital, Athens, Greece

###### **Presenting author:** Despoina Maritsi

**Introduction:** Hepatitis A is a vaccine preventable disease with intermediate endimicity in Greece. National vaccination against Hepatitis A Virus (HAV) was introduced in 2005; however approximately 25% of the population remains unvaccinated. Juvenile SLE(jSLE) patients are susceptible to infections due to their defective immune system and the immunosuppressive treatment they receive.

**Objectives:** The aim of this study is to define the immune status against HAV in patients with jSLE, prior to commencement of treatment and one and three years after treatment, and compare this to healthy controls.

**Methods:** Single-center prospective study including 21 patients newly diagnosed with SLE and 76 healthy controls. All patients had two doses of the inactivated Hepatitis A vaccine in early childhood. Exclusion criteria were underlying immunodeficiency, recent blood transfusion (<6 months) and previous treatment with immunomodulating agents. Demographic and clinical and laboratory data as well as data regarding immunization status, vaccine history and mean time between the doses of the vaccine were collected. Seroprotection rates (>20 mIU/ml) and anti-HAV IgG titers were measured at enrollment and at timely intervals afterwards. Data were analyzed using SPSS 19.0.

**Results:** The two groups had similar characteristics, vaccination history and immunization status. No significant differences were detected in terms of vaccine type, time interval between the two groups as well as mean time from last vaccination to blood sampling. Seroprotection rates at enrollment were 93% for the SLE and 98% for the control group. Mean anti-HAV IgG antibodies were significantly lower in the SLE compared to the control group(167mIU/ml versus 249mIU/ml) (p < 0.01). Same results were found at 1 and three years’ follow up (Table 11). During the follow up period the SLE group had greater decrease in antibody levels as indicated from the significant interaction effect of analysis.

**Conclusion:** Although seroprotection rates were similar between the two groups mean anti-HAV IgG titers were significantly lower in the SLE group at enrollment and at one and three years’ follow-up. Low antibody titers could be attributed to disease, disease activity or medications. Further studies are required to address the question of long-term immunity conveyed by immunizations given at an early stage in children with rheumatic diseases. However, evaluation of immunization status against all vaccine preventable diseases in such patients may be beneficiary.

**Disclosure of Interest**

None Declared.

**Table 11 (abstract P203).** Demographic characteristics, seroprotection rates and mean anti-HAV IgG titers for the SLE and the control group

Parameters	SLE group	Control group	P value
Study sample, n	21	76	0.9*
Age, years, mean (SD)	11.3 (2.3)	10 (2.7)	0.8*
Gender n (%)			
female	20 (95%)	72 (95%)	0.6+
male	1 (5%)	4 (5%)	
Fully vaccinated	16 (71%)	62 (83%)	0.7+
Partially vaccinated (other than HAV)	5 (29%)	14 (17%)	
Type of vaccine given			
-HAVRIX	34 (81%)	119 (78%)	0.84+
-VAQTA	8 (19%)	33 (22%)	
Mean interval between doses (months)	19	23	0.3*
Mean time from last dose to sampling (years)	6	5.4	0.7*
Seroprotection rate at diagnosis (%)	95	98	0.4+
Seroprotection rate at 1 year (%)	90	97	0.1+
Seroprotection rate at 3 years (%)	86	96	0.07+
Mean IgG titers at diagnosis (mIU/ml)	167	249	<0.01*
Mean anti HAV IgG titers at 1 year	131	218	
Mean anti HAV IgG titers at 3 years	121	202	

P-value: * Student t-test, +Pearson’s chi-square test

### P204 Recurrent angioedema in a patient with systemic lupus erythematosus

#### Edi Paleka Bosak, Mandica Vidović, Mirta Lamot, Lovro Lamot, Miroslav Harjaček

##### Clinical Hospital Center Sestre Milosrdnice, Zagreb, ZAGREB, Croatia

###### **Presenting author:** Edi Paleka Bosak

**Introduction:** Systemic lupus erythematosus (SLE) is an autoimmune rheumatic disease that results from the interaction of multiple enviromental, immunological and genetic factors, causing inflammation and eventually damage in multiple organs. Its prevalence ranges from approximately 40 cases per 100,000 and 15–20% of cases present in children under 16 years of age. It has been suggested that peadiatric lupus patients have more aggressive disease course and an increased rate of more unusual initial clinical presentations compared with their adult counterparts. Clinical feature may include urticarial vasculitis with prevalence of up to 2,2%, while angioedema is very rare.

**Objectives:** We present a 15 year old female patient with SLE, urticarial vasculitis and angioedema.

**Methods:** The disease started with nonspecific symptoms of malaise and headache and the patient was even put on klonazepam because of irregular electroencephalogram (EEG). She also developed autoimmune thrombocytopaenia and prednisone therapy was included for several months. Few months prior to admittance to our Department because of recurrent urticarial rash and angioedema allergy tests were performed and all were negative.

During an episode of urticarial rash and vascullitis, skin biopsy was performed and leukocytoclastic vasculitis was diagnosed. Direct immnofolurescency showed IgA, IgG, IgM, C3 and C1q depostis. With symptoms that included malaise, fever, poliarthralgias, headache that developed, laboratory tests and skin biopsy findings SLE was suspected.

**Results:** Antinuclear antibodies (ANA) titer was positive up to 1: 1280 (anti Ro 3+, anti Ro-52 3+ i anti La 1+) and with low concentration of C3 and C4 ACR-criteria were met for SLE. Patient was treated with "pulse“ doses of metilprednisolone followed by maintenance doses. Nevertheless, she developed angioedema of lips, tounge nad eyelids with obstructive breathing. She recived adrenaline, glucocorticoides and antihistamines with poor effect and only after infusion of fresh frozen plasma, symptoms decreased. At the time, levels of C1 esterase inhibitor (C1-INH) were normal, while C1-INH function and C1q levels are still expected

**Conclusion:** Angioedema is an unusual occurence in SLE, especially in children and it's mechanisms are heterogenous.SLE can be associated with hereditary and acquired deficinecies or dysfunction of C1-INH which leads to hyperactivation of complement classic pathway, bradykinin synthesis and incrased vascular permeability. Anti C1-INH autoantibodies seem to be uncommon in acquired angioedema (AAE) in SLE. In our patient, angioedema is thought to be most likely of the acquired form secondary to SLE since in hereditary angioedema (HAE) there is usually a family history of angioedema and earlier onset of symptoms. Although angioedema is generally self-limited disease, it may be life threatening causing the airway obstruction with mortality rate 15-33%.

**Disclosure of Interest**

None Declared.

### P205 Ikaros deficiency in early onset lupus

#### Erika Van Nieuwenhove^1,2,3^, Adrian Liston^1,3^, Carine Wouters^2,3^

##### ^1^Translational immunology laboratory, VIB TRANSLATIONAL IMMUNOLOGY/KULEUVEN AUTOIMMUNE GENETICS, Leuven, Belgium; ^2^Department of Pediatrics, University Hospital Leuven, Leuven, Belgium; ^3^Microbiology and immunology, University of Leuven, Leuven, Belgium

###### **Presenting author:** Erika Van Nieuwenhove

**Introduction:** Kuehn et al. identified novel *IKZF1* mutations as the cause of an autosomal dominant form of common variable immunodeficiency (CVID), characterised by a progressive loss of B cells. Ikaros is a transcriptional regulator required for B cell development but Woijcik et al. also highlight the role of Ikaros in regulating quiescence of the peripheral B cell pool as well as in maintenance of B cell tolerance in an ikaros deficient murine model.

**Objectives:** To report a novel *IKZF1* mutation in a patient presenting with early-onset severe systemic lupus erythematosus.

**Methods:** Genomic DNA was isolated from heparinized whole blood. Paired-end sequencing was performed on an Illumina HiSeq 2500 (Genomics Core Facility). Generated sequence reads were aligned to Human Reference Genome Build hg19 using Burrows-Wheeler Aligner software and GATK Haplotype Caller was applied for base calling. ANNOVAR software was used for annotation. Found variants were confirmed by Sanger sequencing (LGC Genomics Facility, Berlin, Germany).

Flow cytometry was carried out using peripheral blood mononuclear cells (PBMCs) on our previously described immune phenotyping platform (Carr et al. 2015).

NIH3T3 cells (1x105)) were seeded onto cover slips in 6 well plates and allowed to attach overnight. Cells were transfected with plasmids containing either wild type Ikaros (NM_006060) or Ikaros with the L188V point mutation using Lipofectamine 3000 Reagent (Invitrogen) according to the manufacturer’s instructions. Cells were fixed in 4% paraformaldehyde and permeabilised in 0.1% Triton X-100 in PBS at RT, followed by blocking. Cells were incubated for 2 hours with mouse anti-HA monoclonal antibody (Eurogentec) and/or rabbit anti-Flag polyclonal affinity antibody (Sigma). Cells were subsequently incubated for one hour with DAPI and either SA-488 (green) or Alexa Fluore 555 (red) in blocking buffer. Finally cells were mounted on slides using Fluoromount-G (SouthernBiotech). Images were collected on a confocal microscope with a 60X immersion objective.

**Results:** The patient presented at 12 years of age with severe asthenia, weight loss, adrenal insufficiency, hepatosplenomegaly, pancytopenia, highly increased levels of anti-nuclear, anti-dsDNA, antiphospholipid auto-antibodies and complement activation. IgG levels were initially normal, however initiation of corticosteroid therapy (with hydroxychloroquine and anticoagulation) to control autoimmunity resulted in a progressive and persistent decline in B cell numbers and IgG levels, prompting subcutaneous Ig substitution.

A private heterozygous mutation was detected in *IKZF1* in both father and index patient (NM_006060:exon5:c.C562G:p.L188V). Flow cytometry confirmed the low level of B cells. Confocal microscopy showed diffuse nuclear staining in NIH-3 T3 cells transfected with the L188V mutated Ikaros, rather than the punctate staining pattern that is characteristic of wild type Ikaros upon colocalisation to pericentromeric heterochromatin.

**Conclusion:** We identified a mutation in *IKZF1* as a new cause for an autosomal dominant form of early onset monogenic systemic lupus. Our findings greatly expand the clinical spectrum of Ikaros deficiency from CVID to early-onset SLE. The variable presentation of Ikaros deficiency highlights the broadening spectrum from primary immunodeficiency to autoimmunity.

**Disclosure of Interest**

None Declared.

### P206 Systemic lupus erythematosus and neurologic problems in children

#### Fatemeh Tahghighi, Vahid Ziaee, Seid-Reza Raeeskarami

##### Pediatric Rheumatology, Tehran University, Tehran, Islamic Republic of Iran

###### **Presenting author:** Fatemeh Tahghighi

**Introduction:** Systemic lupus erythematosus is a multisystem involvement disorder with various clinical presentations. Central nervous system (CNS) lupus is a serious but potentially treatable illness. Children with SLE often have neurologic symptoms, and SLE is sometimes diagnosed after patients present for treatment of a neurologic event.

**Objectives:** Systemic lupus erythematosus is a multisystem involvement disorder with various clinical presentations. Central nervous system (CNS) lupus is a serious but potentially treatable illness. Children with SLE often have neurologic symptoms, and SLE is sometimes diagnosed after patients present for treatment of a neurologic event

**Methods:** We studied 62 patients with Juvenile systemic lupus erythematosus (JSLE) were followed at The Children's Medical center of Tehran University between 2004 and 2014 for evidences of central nervous system (CNS) involvement. In this study CNS involvement included evidence of organic brain syndrome, objective neurologic signs or symptoms referable to CNS.

**Results:** Thirty-eight(61.2%) of 62 children had CNS involvement.Thirty patients(48.3%) had CNS involvement at the onset of JSLE. Eight patients(12.9%) had late onset CNS manifestations 1 to 2 years after the diagnosis of JSLE. The most frequently observed symptoms were headache(46.77%), behavior disorder(8.06%), dizziness(8.06%), and alteration of consciousness(4.8%). The most frequently observed neurologic sign was seizure(9.6%). Neuropsychiatric manifestations included organic brain syndrome, functional psychosis, and personality disorder. All of pataints with CNS manifestations are alive (100%). A residual neurologic abnormality as a seizure disorder, was present in 2(3.22%).

**Conclusion:** Although CNS involvement with SLE in children is serious but carries a favorable prognosis.

**Disclosure of Interest**

None Declared.

### P207 Neonatal lupus erythematosus presenting with heart block and pneumonitis: a difficult case

#### Francisca Aguiar^1^, Sandra Pereira^2^, Mariana Rodrigues^2,3^, Cláudia Moura^3,4^, Gustavo Rocha^3,5^, Hercília Guimarães^3,5^, Iva Brito^1,3^

##### ^1^Rheumatology, Centro Hospitalar São João, Oporto, Portugal; ^2^Pediatrics, Centro Hospitalar São João, Oporto, Portugal; ^3^Faculty of Medicine of Porto University, Oporto, Portugal; ^4^Pediatric Cardiology, Centro Hospitalar São João, Oporto, Portugal; ^5^Neonatology, Centro Hospitalar São João, Oporto, Portugal

###### **Presenting author:** Francisca Aguiar

**Introduction:** Neonatal lupus erythemathosus (NLE) is a rare auto-immune condition of neonates related to the transplacental passage of maternal autoantibodies (anti-SSA/Ro, SSB/La and rarely anti-U1RNP) to the foetus after the 16th week of gestation leading to lesions in target organs. The most common findings include skin lesions and/or congenital heart block, but hepatobiliary disease and haematological abnormalities can also occur. Other systemic features as pneumonitis have been occasionally documented.

**Objectives:** Our aim is to describe a case of Acute Lupus Pneumonitis in a newborn with NLE.

**Methods:** A boy born to a 35-year-old mother with systemic lupus erythemathosus with positivity for anti-SSA and anti-SSB, and a previous child with NLE with congenital heart block who died at the age of two due to myocarditis. The pregnancy was surveilled, the mother was treated with prednisolone and hydroxychloroquine and a prenatal diagnosis of heart block was made. The mother received dexamethasone and salbutamol but refused endovenous immunoglobulin and plasmapheresis.

**Results:** The child was born during the 36th week of gestation, by cesarean, with an Apgar 1´/5´of 9/10, complete heart block was confirmed and he was admitted to the Department of Neonatology. 24 hours after birth a temporary pacemaker was inserted, which was only replaced by a definitive pacemaker when the patient was 14 days old due to concurrent sepsis. Empiric antibiotic intravenous therapy with meropenem and vancomycin was started. At D22 he developed hypoxemic acute respiratory failure requiring mechanical ventilation, with chest radiographs showing diffuse bilateral cotton-like infiltrates. In the following 48 hours there was clinical worsening, despite the broad-spectrum antibiotics, absence of fever and progressive normalization of leukocyte count and C-reactive protein level. At this time pediatric rheumatology evaluation was requested. The neonate had a history of transitory rough skin rash on chest and back after birth; thrombocytopenia probably in the context of neonatal sepsis whithout other hematologic abnormalities; hepatomegaly without elevation of liver enzymes. The immunology panel showed positivity for antinuclear antibodies at titer 1/320 (speckled pattern), positivity for antibody anti-SSA and also anti-dsDNA, with absence of anti-SSB, anti-RNP. The clinical picture was interpreted as NLE-related pneumonitis and the patient received intravenous methylprednisolone pulses (30 mg/kg/day) on 3 consecutive days, followed by oral prednisolone in progressively lower doses, with excellent clinical and imagiological response: after the second pulse he started to ventilate spontaneously and after 7 days supplementary oxygen was no longer needed.

**Conclusion:** This case represented a challenge as pulmonary manifestations presented in a neonate with NLE with congenital heart block that was being treated for neonatal sepsis. Besides the rarity of NLE-related pneumonitis, treatment with corticosteroids had a lot of risks, especially in this patient. However, the imagiological and immunological findings and the fact that there was a good response to corticotherapy and not to broad spectrum antibiotics made this diagnosis the most probable, with a successful outcome.

**Disclosure of Interest**

None Declared.

### P208 Association between haematological manifestations and immunological characteristics in juvenile systemic lupus erythematosus

#### Francisca Aguiar^1^, Rita Fonseca^1^, Mariana Rodrigues^2,3^, Iva Brito^2,3^

##### ^1^Rheumatology, Centro Hospitalar São João, Oporto, Portugal; ^2^Faculty of Medicine of Porto University, Oporto, Portugal; ^3^Pediatric Rheumatology Unit, Centro Hospitalar São João, Oporto, Portugal

###### **Presenting author:** Francisca Aguiar

**Introduction:** Although haematological abnormalities are common in juvenile systemic lupus erythematosus (jSLE), their frequency varies in different populations. In addition, the relationship of these abnormalities with various organ system involvement has not been widely studied in jSLE populations.

**Objectives:** To assess haematological manifestations in jSLE patients and study its associations with clinical and immunological characteristics.

**Methods:** Retrospective observational study including patients with SLE that are or were followed in our Paediatric Rheumatology Unit. Clinical, demographic and laboratory characteristics were retrospectively collected by consulting medical records. All patients fulfilled the clinical and laboratory criteria of the American College of Rheumatology (ACR). The haematologic manifestations were defined according to the nomenclature and classification of ACR. Patients with different haematologic manifestations were compared using Student t-test, Mann-Whitney test, Chi-square or Fisher test (SPSS 23.0). Significance level was set as <0.05.

**Results:** 42 patients were included, 92.9% (39) were female, with a mean age at diagnosis of 13.7 ± 3.6 years. Median period between onset of symptoms and diagnosis of SLE was 0.3 [0-1.2] years and median duration of follow-up was 15 [0.8-46] years. The majority (59.5%) had haematological manifestations (31.0% leukopenia, 28.6% lymphopenia, 28.6% thrombocytopenia, 23.8% haemolytic anaemia). 84.3% of the patients also had mucocutaneous manifestations, 78.9% musculoskeletal symptoms, 50% renal involvement, 21% serositis and 13.2% neuropsychiatric involvement. Patients with haematological manifestations were younger at diagnosis (12.8 ± 3.6 vs 15.1 ± 3.3 years, p = 0.04), had more frequently malar rash (96% vs 52.9%, p = 0.001), renal involvement (64.0% vs 29.4%, p = 0.03), positive coombs' test (57.9% vs 21.4%, p = 0.028) and higher SLEDAI (4.0 [0.0-14.0] vs 0.0 [0.0-6.0], p < 0.001). Analysing by type of haematological manifestation, patients with haemolytic anaemia were more frequently female (70% vs 30%, p = 0.01), although all male patients presented this abnormality, had less frequently photosensivity (50% vs 84.4%, p = 0.04) and had higher SLEDAI (4.0 [2.0-14.0] vs 1.5 [0.0-14.0], p = 0.007). Those with thrombocytopenia had less frequently mucosal ulcers (18.2% vs 53.8%, p = 0.048) but more frequently renal involvement (75% vs 40% p = 0.043).Patients with leukopenia had more frequently malar rash (100% vs 69.0%, p = 0.022) and positivity for anti-SSa/Ro (53.8% vs 17.2%, p = 0.021). Those with lymphopenia had more frequently malar rash (100% vs 70%, p = 0.032) and positive coombs' test (89.0% vs 40%, p = 0.024).

**Conclusion:** Haematologic manifestations are very common in jSLE and in our study these abnormalities were associated with higher disease activity and certain organ involvement and/or immunological markers. These results should be confirmed in studies with larger sample sizes, as this information may help in better management planning of jSLE patients.

**Disclosure of Interest**

None Declared.

## Poster Session: Treatment

### P209 Comparison of treatment response, remission rate and drug adherence in polyarticular juvenile idiopathic arthritis patients treated with etanercept, adalimumab or tocilizumab

#### Gerd Horneff^1^, Ariane Klein^1^, Kirsten Minden^2^, Hans-Iko Huppertz^3^, Frank Weller-Heinemann^4^, Jasmin Kuemmerle-Deschner^5^, J-Peter Haas^6^, Anton Hospach^7^ and BIKER collaborative group

##### ^1^ASKLEPIOS, Sankt Augustin, Germany; ^2^Charite, Berlin, Germany; ^3^Prof Hess Children's Hospital, Bremen, Germany; ^4^Prof. Hess Children's Hospital, Bremen, Germany; ^5^University, Tuebingen, Germany; ^6^German Centre Pediatric and Adolescent Rheumatology, Garmisch-Partenkirchen, Germany; ^7^Olgahospital, Stuttgart, Germany

###### **Presenting author:** Gerd Horneff

**Introduction:** Head to head studies on biologics in polyarticular JIA are not available.

**Objectives:** To compare treatment response, remission rates and drug adherence in polyJIA pts treated with Adalimumab (ADA), Etanercept (ETA), or Tocilizumab (TOC).

**Methods:** Data prospectively collected within the BIKER registry were analyzed in polyJIA pts starting approved biologics ADA, ETA or TOC from 2011-2015. Baseline patients’ characteristics, treatment response, safety and drug survival were compared.

**Results:** 236 patients started ADA, 419 ETA and 74 TOC with several differences in baseline patients’ characteristics: The JADAS10 (mean+/-SD) in the ADA/ETA/TOC cohort was 12.1+/-7.6;13.8+/-7.1;15.1+/-7.4 (ADA vs ETA p < 0.004) and the CHAQ-disability index was 0.43 +/-0.58;0.59 +/- 0.6;0.63+/-0.55 (ADA vs ETA p < 0,001). Uveitis was more frequent in the ADA cohort (Odd’s ratio 5.73; p < 0.0001). PedACR30/50/70/90 criteria already at month 3 was reached in 69%/60%/42%/24% with ETA, 68%/61%/44%/27% with ADA and 61%/51%/33%/ 25% with TOC. JADAS remission was reached in 52 (25.7%), 130 (35.3%), and 16 (29.4%) of patients of the ADA/ ETA/TOC cohort (Odd’s ratio for ETA 1.6; 95%CI 1.14-2.25; p = 0,006, for ADA 0.67; 95% CI 0.47-0.97; p = 0.03). JADAS minimal disease activity was reached in 99 (49.1%), 211 (57.3%), and 26 (50.9%) of patients of the ADA/ETA/TOC cohorts. In the ETA cohort it was used in 95.5% as a first biologic, in the ADA cohort in 51.7 and in the TOC cohort in 20.2% only. There were no important efficacy differences in first line or second line use of the 3 biologics. 142 (60.4%)/207 (49.4%)/23 (31.1%) patients discontinued ADA/ETA/TOC (Odd’s ratio for ADA 1.73; p = 0.006; for TOC 0.395 ; p = 0.0003). Drug survival rates of patients on monotherapy with a biologic compared to combination therapy with methotrexate revealed no significant difference. During 4 years of observation under ETA/ADA/TOC 996/386/103 adverse events and 148/119/26 serious AE were reported.

**Conclusion:** In clinical practice, ETA is the most frequently used as first biologic, ADA is used equally as first or second while TOC is used usually as a second or third line biologic. ADA/ETA/TOC showed comparable efficacy in polyarticular JIA. Remission was reached more frequently upon ETA and TOC than upon ADA. Overall, tolerance was acceptable. Interestingly, drug adherence was highest with TOC and lowest with ADA. Since patient cohorts differed at baseline these results are preliminary needwell controlled head to head studies for confirmation.

**Disclosure of Interest**

None Declared.

**Table 12 (abstract P209).** Patients’ characteristics at baseline

	Etanercept-cohort N = 419	Adalimumab -cohort N = 236	Odd’s ratio# (95%CI); p	Tocilizumab-cohort N = 74	Odd’s ratio# (95%CI); p
Female, n (%)	332 (79.2%)	192 (81.4%)	1.1 (0.7-1.5); 0.78	51 (68.8%)	0.59 (0.38-0.94); 0.027
Age at baseline, mean+/- SD	10.49+/-4.36	11.8 +/-4.0	n.a.; <0.0001	13.90+/-3.63	n.a.; <0.0001
JIA Category n(%) RF + PA/FR-PA/ExOA	37 (8.8%)/224 (53.5%)/158 (37.7%)	23 (9.7%)/128 (54.2%)/85 (36.0%)	n.s.	9 (12.2%)/47 (63.5%)/18 (24.3%)	0.58 (0.43-0.99); 0.03
First biologic used	400 (95.5%)	122 (51.7%)	0.08 (0.05-0.14); <0.0001	15 (20.2%)	0.02 (0.01-0.03); <0.0001
Co-Med MTX, n(%)	309 (73.7)	127 (53.8)	0.51 (0.37-0.71); <0.0001	34 (45.9)	0.43 (0.26-0.69); 0.0004
JADAS10 [0-40] mean +/-SD	13.8 +/- 7.1	12.1 +/-7.6	n.a.; 0.004	15.1 +/- 7.4	n.s
CHAQ-DI [0-3] mean +/-SD	0.59 +/- 0.6	0.43 +/-0.58	n.a.; <0.0001	0.63+/-0.55	n.s.
Uveitis before start of biologic	23 (5.5%)	59 (25%)	5.73 (3.43-5.99); 0,0001	0	n.a.

# compared to the Etanercept cohort, n.a. not available, n.s. not significant

### P210 Secondary loss of efficacy of adalimumab in JIA: patients show high levels of anti-drug antibodies

#### Ricardo Menendez-Castro, Boris Huegle, Johannes-Peter Haas

##### German Center for Pediatric and Adolescent Rheumatology, Garmisch-Partenkirchen, Germany

###### **Presenting author:** Johannes-Peter Haas

**Introduction:** Juvenile idiopathic arthritis (JIA) is the most common rheumatic disease in childhood. Anti-tumor necrosis factor (TNF) agents are the most frequently used biologic agents in the long-term treatment of JIA. Monoclonal anti-TNF drugs including adalimumab have been shown to induce formation of anti-drug antibodies (ADA) which are associated with reduced drug levels and higher disease activity in rheumatoid arthritis and inflammatory bowel diseases.

**Objectives:** This study investigated frequency and levels of anti-drug antibodies in JIA patients with initial treatment response to Adalimumab (ADM) and secondary loss of efficacy (LOE).

**Methods:** 53 consecutive JIA patients admitted to the German Center for Pediatric and Adolescent Rheumatology from January 2015 until December 2015 were included in this study. Inclusion criteria were: 1) diagnosis of JIA, 2) treatment with ADM, and 3) achieving remission according to the Wallace criteria. Exclusion criteria were concomitant treatment with intravenous immunoglobulins and obvious non-compliance due to corresponding information and/or equally negative levels of ADM and ADA. Secondary LOE was defined as any inflammatory joint activity after achieving remission. Other reasons for testing were arthralgia (but no arthritis), severe local reaction and explicit parental request. ADA and drug levels were determined by commercial ELISA kits. Data were analyzed using descriptive statistics and univariate parametric/non-parametric tests.

**Results:** Main patient characteristics and descriptive statistics are summarized in the table below.

In JIA patients with secondary LOE 19/25 (76.0%) had positive (>10.0 AU/ml) ADA with median level (range) 93.3 (1.0-754.4) AU/ml. By contrast, 1/28 (3.6%) of the JIA patients without secondary LOE had positive ADA with a median level (range) 1.3 (0.4-10.7) AU/ml (p < 0.001). Furthermore, JIA patients with secondary LOE showed significantly lower drug levels than JIA patients without secondary LOE with a median ADM trough level (range) 0.9 (0.4-23.5) μg/ml versus 11.7 (0.4-31.2) μg/ml (P < 0.001).

**Conclusion:** In JIA patients, as seen in adult rheumatic disease as well, the formation of ADA against adalimumab obviously is a major factor causing reduced drug levels and secondary LOE.

**Disclosure of Interest**

None Declared.

**Table 13 (abstract P210).** See text for description

	25 JIA patients with secondary LOE	28 JIA patients without secondary LOE
median age (range)	14.4 (6.0-27.0) years	13.8 (7.6-21.5) years
Sex	21 female, 4 male	18 female, 10 male
median ADM dose (range)	25.8 (10.7-58.8) mg/m^2^ every other week	24.3 (6.6-37.1) mg/m^2^ every other week
median duration of anti-TNF therapy (range)	2.2 (0.2-6.0) years	1.3 (0.2-10.4) years
MTX comedication	12/25 (48.0%)	15/28 (53.6%)
median MTX dose (range)	10.0 (3.9-12.1) mg/m^2^ weekly	10.8 (7.7-13.4) mg/m^2^ weekly

### P211 Risk of infections in juvenile idiopathic arthritis patients treated with biologic agents and/or methotrexate: results from pharmachild registry

#### Joost Swart, Gabriella Giancane, Francesca Bovis, Elio Castagnola, Andreas Groll, Gerd Horneff, Hans-Iko Huppertz, Daniel J. Lovell, Tom Wolfs, Michael Hofer, Ekaterina Alekseeva, Violeta Panaviene, Susan Nielsen, Jordi Anton, Florence Uettwiller, Valda Stanevicha, Maria Trachana, Denise Pires Marafon, Constantin Ailioaie, Elena Tsitsami, Sylvia Kamphuis, Troels Herlin, Pavla Doležalová, Gordana Susic, Berit Flatø, Flavio Sztajnbok, Angela Pistorio, Alberto Martini, Nico Wulffraat, Nicolino Ruperto

##### Pediatria II, Reumatologia; PRINTO, Istituto Giannina Gaslini, GENOA, Italy

###### **Presenting author:** Joost Swart

**Introduction:** Pharmachild is an international registry involving over 100 PRINTO/PRES centres in 38 countries. The registry was set up to evaluate long term safety and efficacy of treatments in children with JIA

**Objectives:** To evaluate the occurrence of infection in JIA children and their relationship with biologics ± MTX and other immunosuppressive treatments

**Methods:** Retrospective and prospective evaluation of moderate to very severe and serious infections. We investigated, by means of bivariate and multivariate modeling, the relationship between infections and the drugs used in the 6 month-history prior to the infection and, for patients without infections, in the 6 months prior to the last follow-up visit. The patients receiving only NSAIDs or intra-articular steroid injections were used as a reference

**Results:** We analyzed 6969 patients, for a total of 689 (10%) infections. Patients were systemic 639 (9%), polyarticular course 4587 (66%) and persistent oligoarthritis 1473 (25%). Patients with infections were treated in the previous 6 months primarily with MTX (82%), corticosteroids (32%), anti-TNF (52%), anti IL1/IL6 (8%), rituximab (2%) or other biologics (2%).Earlier age at diagnosis (≤7.7 years), ANA positivity, and systemic JIA category significantly increased the risk for infection (OR 2.3, 1.7 and 2.2, respectively). The analysis of the impact of the single immunosuppressive drugs showed that the risk for infection is increased by corticosteroids (OR 3.7), MTX (OR 2.8), cyclosporine (OR 5.3) and thalidomide (OR 13.7, 3/5 patients with infections). The same results were observed for biologics, in particular rituximab (OR 16.3, 14/22 patients with infections), IL-1 inhibitors as anakinra (OR 3.3) and, among TNF-α inhibitors, infliximab (OR 1.6). Conversely, abatacept resulted protective (OR 0.4). The multivariate analysis (table) revealed that the addition of steroids to both MTX and biologics (groups MTX + steroids and MTX + steroids + biologics) increased the risk of infections more significantly (OR 11.9 and 10.5, respectively) than only MTX (5.1) or MTX + Biologics (OR 3.7). A great increase in the risk for infection was associated with rituximab ± steroids ± DMARDS (OR 112, 95% CI 11-1000). The multivariate results were confirmed by removing rituximab patients from the model

**Conclusion:** Pharmachild showed that MTX and biologics increase the risk of infection in JIA patients. This risk is significantly enhanced by the addition of steroids to immunosuppressive therapy. We recommend monitoring for infections in JIA patients on immunosuppressive therapy

**Disclosure of Interest**

J. Swart: None Declared, G. Giancane: None Declared, F. Bovis: None Declared, E. Castagnola Consultant for: Pfizer, Astellas Pharma, Basilea Pharmaceutica, A. Groll: None Declared, G. Horneff Speaker Bureau of: Abbvie,Pfizer, Chugai Roche and Novartis, H.-I. Huppertz: None Declared, D. Lovell Grant / Research Support from: National Institutes of Health, NIAMS, Consultant for: Astra-Zeneca, Bristol Meyers Squibb, AbbVee, Pfizer, Roche, Novartis, UBC, Forest Research Institute, Horizon, Johnson & Johnson, Biogen, Takeda, Genentech, Glaxo Smith Kline, Boehringer Ingelheim, Celgene, Jannsen, T. Wolfs: None Declared, M. Hofer Consultant for: Novartis, E. Alekseeva: None Declared, V. Panaviene: None Declared, S. Nielsen: None Declared, J. Anton: None Declared, F. Uettwiller: None Declared, V. Stanevicha: None Declared, M. Trachana: None Declared, D. Pires Marafon: None Declared, C. Ailioaie: None Declared, E. Tsitsami: None Declared, S. Kamphuis: None Declared, T. Herlin: None Declared, P. Doležalová Grant / Research Support from: AbbVie, Roche, Medac, Novartis, Pfizer, Consultant for: Roche, Speaker Bureau of: Pfizer, Novartis, Medac, G. Susic: None Declared, B. Flatø: None Declared, F. Sztajnbok: None Declared, A. Pistorio: None Declared, A. Martini Grant / Research Support from: The G. Gaslini Hospital, which is the public Hospital where I work as full time public employee, has received contributions from the following industries for the coordination activity of the PRINTO network: BMS, GlaxoSmithKline (GSK), Hoffman-La Roche, Novartis, Pfizer, Sanofi Aventis, Schwarz Biosciences, Abbott, Francesco Angelini S.P.A., Sobi, Merck Serono. This money has been reinvested for the research activities of the hospital in fully independent manners besides any commitment with third parties., Speaker Bureau of: I received speaker’s bureaus and consulting fees from the following pharmaceutical companies: Abbvie, Boehringer, Celgene, CrescendoBio, Janssen, Meddimune, Novartis, NovoNordisk, Pfizer, Sanofi Aventis, Vertex, Servier., N. Wulffraat: None Declared, N. Ruperto Grant / Research Support from: The G. Gaslini Hospital, which is the public Hospital where I work as full time public employee, has received contributions from the following industries for the coordination activity of the PRINTO network: BMS, GlaxoSmithKline (GSK), Hoffman-La Roche, Novartis, Pfizer, Sanofi Aventis, Schwarz Biosciences, Abbott, Francesco Angelini S.P.A., Sobi, Merck Serono. This money has been reinvested for the research activities of the hospital in fully independent manners besides any commitment with third parties., Speaker Bureau of: I received speaker’s bureaus and consulting fees from the following pharmaceutical companies: AbbVie, Amgen, Biogenidec, Alter, AstraZeneca, Baxalta Biosimilars, Biogenidec, Boehringer, BMS, Celgene, CrescendoBio, EMD Serono, Hoffman-La Roche, Italfarmaco, Janssen, MedImmune, Medac, Novartis, Novo Nordisk, Pfizer, Sanofi Aventis, Servier, Takeda, UCB Biosciences GmbH.

**Table 14 (abstract P211).** Risk factors for infections in JIA patients by multivariate analysis. (REF: reference item)

	Patients with infections 689	Multivariate analysis OR (95%CI)
Drug Therapy Only NSAIDs and Intraarticular Steroids (REF)		
-Rituximab ± Steroids ± DMARDS	12/16 (75%)	111.8 (10.7-999.9)
-MTX + Biologics + Steroids	95/422 (23%)	10.5 (6.5-17.0)
-MTX + Steroids	47/207 (23%)	11.9 (7.0-20.2)
-Other combinations	102/689 (15%)	7.6 (4.8-12.0)
-Only MTX	157/1418 (11%)	5.1 (3.3-7.9)
-MTX + Biologics	181/2055 (9%)	3.7 (2.4-5.8)
-Only Biologics	71/1077 (7%)	2.7 (1.7-4.3)
Diagnosis (Oligo Persistent arthritis as REF)		
-Other JIA categories with polyarticular course		1.3 (1.1-1.6)
-Systemic arthritis		1.7 (1.3-2.3)
Age at JIA diagnosis (cut-off ≤7.7)		2.3 (1.9-2.7)
ANA (2 positive >1:160)		1.6 (1.3-1.9)

### P212 Treatment of idiopathic recurrent pericarditis with anakinra. The airtrip randomized clinical trial

#### Marco Gattorno^1^, Antonio Brucato^2^, Martina Finetti^1^, George Lazaros^3,4^, Silvia Maestroni^2^, Mara Carraro^3^, Davide Cumetti^2^, Alessandra Carobbio^2^, Monia Lorini^2^, Alessandro Rimini^5^, Renzo Marcolongo^6^, Anna Valenti^2^, Gian Luca Erre^7^, Riccardo Belli^3^, Fiorenzo Gaita^8^, Maria Pia Sormani^9^, Nicolino Ruperto^1^, Massimo Imazio^3^, Alberto Martini^1^

##### ^1^Rheumatology Unit, Giannina Gaslini Institute, Genoa, Italy; ^2^Internal Medicine Division, Research Foundation, and Clinical Pharmacology, Ospedale Papa Giovanni XXIII, Bergamo, Italy; ^3^Cardiology Department, Maria Vittoria Hospital and Department of Public Health and Pediatrics, Torino, Italy; ^4^Department of Cardiology, University of Athens Medical School, Hippokration Hospital, Athens, Greece; ^5^Cardiology Unit, Giannina Gaslini Institute, Genoa, Italy; ^6^Clinical Immunology, Department of Medicine, Azienda Ospedaliera-Università, Padua, Italy; ^7^Rheumatology Unit, Azienda Ospedaliero-Universitaria di Sassari, Sassari, Italy; ^8^University Division, Department of Medical Sciences, Città della Scienza e della Salute, Torino, Italy; ^9^University of Genoa, Genoa, Italy

###### **Presenting author:** Marco Gattorno

**Introduction:** Colchicine resistant and corticosteroid dependent (unable to withdraw the drugs without relapse) recurrent pericarditis is a major management challenge. Anakinra, an interleukin (IL)-1 beta recombinant receptor antagonist, is promising in this setting.

**Objectives:** To determine the efficacy and safety of anakinra in colchicine resistant and corticosteroid dependent recurrent pericarditis in the first randomized controlled study.

**Methods:** Investigator-initiated, double-blind, placebo 33 controlled, randomized withdrawal trial among 21 consecutive patients with colchicine resistant and corticosteroid dependent recurrent pericarditis enrolled in 3 Italian referral centers between June and November 2014. At enrolment, mean age was 45.4 years (SD 14.3 years), 67% were females. Included patients had recurrent pericarditis (with ≥3 previous recurrences), elevation of C-reactive protein, colchicine resistance and corticosteroid-dependence. Anakinra was administered at 2 mg/kg/day up to 100 mg for 2 months and then patients were randomized to continue anakinra (N = 11) or switched to placebo (N = 10) for 6 months or until a recurrence. The primary outcomes were recurrent pericarditis and time to recurrence after randomization.

**Results:** Recurrent pericarditis occurred in 9 of 10 patients (90%, incidence rate: 2.06% patients/year) assigned to placebo and 2 of 11 patients (18.2%, incidence rate: 0.11% patients/year) assigned to anakinra (hazard ratio: 0.08 95% CI 0.02-0.41). Median time to flare was 72 days from randomization in the placebo group and was not reached in the anakinra group (p = 0.0002).

**Conclusion:** Anakinra is safe and efficacious to control and prevent recurrences in colchicine-resistant and corticosteroid-dependent patients with recurrent pericarditis

**Trial registration identifying number:** ClinicalTrials.gov Identifier: NCT02219828

**Disclosure of Interest**

M. Gattorno Grant / Research Support from: SOBI provided the drug and placebo and the insurance to cover for the patients enrolled in the study, A. Brucato: None Declared, M. Finetti: None Declared, G. Lazaros: None Declared, S. Maestroni: None Declared, M. Carraro: None Declared, D. Cumetti: None Declared, A. Carobbio: None Declared, M. Lorini: None Declared, A. Rimini: None Declared, R. Marcolongo: None Declared, A. Valenti: None Declared, G. L. Erre: None Declared, R. Belli: None Declared, F. Gaita: None Declared, M. P. Sormani: None Declared, N. Ruperto: None Declared, M. Imazio: None Declared, A. Martini: None Declared

### P213 Immunomodulatory therapy for severe juvenile idiopathic arthritis – benefits and risks of biologic disease modifying antirheumatic drugs and haematopoietic stem cell transplantation

#### Mario Abinun

##### Paediatric Immunology, Great North Children's Hospital, Newcastle upon Tyne, UK

**Introduction:** The last 3 decades, since the era of methotrexate and ‘dismantling the pyramid’ [1], and in particular in the current era of new biologic response modifying agents [2], have brought dramatic improvement in treatment of children with juvenile idiopathic arthritis (JIA); however, a substantial percentage (>30%) still carries on with active disease in adulthood, and a small minority remains refractory to current therapies, with significant morbidity and cumulative side effects, especailly from infections.

**Objectives:** Report treatment failures and significant infectious side-effect, including deaths, in a cohort of patients with severe, refractory JIA referred for haematopoietic stem cell transplantation (HSCT).

**Methods:** Data analysis from our centre, for the period 2000-2016.

**Results:** In the period 2000-2007, of the 13 referred patients 5 declined HSCT or were deferred after independent assessment; 2 died before HSCT; 6 underwent autologous T cell depleted HSCT [3], of which 3 are in long-term (>10 yrs) complete remission (CR), 1 relapsed within 6 months post-HSCT, and 2 died. All 4 deaths were caused by infections, 3 from central venous catheter related bacterial sepsis (1 during HSCT procedure, and 2 in patients receiving high-dose steroids, methotrexate and TNF-blocking biologics) and 1 from disseminated adenovirus infection (during HSCT procedure).

In the period 2007-2016, 4 of 5 referred patients underwent allogeneic HSCT [4] of which 3 are in long-term (1-4 yrs) CR, and 1 is only 2 months post-HSCT; 1 patient was deferred after independent assessment.

**Conclusion:** Severe infections are emerging as an important mortality risk factor in children with severe, refractory JIA treated with combined and multiple immunosuppressive and anti-inflammatory therapies. In this small group of patients, these risks should be carefully assessed towards the risks (and potential benefits) from allogeneic HSCT.

**References**

1. Levinson JE, Wallace CA. Dismantling the pyramid. J Rheumatol (Suppl) 1992:33:6-10

2. Sandborg C, Mellins ED. A new era in the treatment of systemic juvenile idiopathic arthritis. N Engl J Med 2012;367:25

3. Abinun M et al. Autologous T cell depleted haematopoietic stem cell transplantation in children with severe juvenile idiopathic arthritis in the UK (2000-2007). Moll Immunol 2009;47:46-51

4. Silva J et al. Allogeneic haematopoietic stem cell transplantation for systemic onset juvenile idiopathic arthritis. Biol Blood Marrow Transplant 2015;21:S46 (Abs No 25)

**Disclosure of Interest**

None Declared.

### P214 Clinical experience and decision making in relation to the use of biologics in adult JIA

#### Nicola Smith^1^, Tim Rapley^2^, Flora McErlane^3^, Lianne Kearsley-Fleet^4^, Kimme L. Hyrich^4,5^, Helen Foster^1,3^

##### ^1^Musculoskeletal Research Group, Institute of Cellular Medicine, Newcastle University, Manchester, UK; ^2^Institute of Health and Society, Newcastle University, Manchester, UK; ^3^Paediatric Rheumatology, Great North Children’s Hospital, Newcastle Upon Tyne, UK; ^4^Arthritis Research UK Centre for Epidemiology, Centre for Musculoskeletal Research, The University of Manchester, Manchester, UK; ^5^NIHR Manchester Musculoskeletal Biomedical Research Unit, Central Manchester University Hospitals NHS Foundation Trust, Manchester, UK

###### **Presenting author:** Nicola Smith

**Introduction:** Juvenile Idiopathic Arthritis (JIA) is the most common chronic inflammatory musculoskeletal disease of childhood in the UK. More than 30% of patients will continue to have active disease as adults, with some requiring a biologic for the first time after the age of 18 years. Prior to December 2015, there was no national UK approval for the use of biologics in this older population, albeit they were used in adults with JIA.

**Objectives:** This mixed-methods study aimed to explore the clinical experience and decision-making of healthcare professionals in relation to the use of biologics in adults with JIA.

**Methods:** Multi-method research was undertaken comprising of an online survey and one-to-one telephone interviews. An online survey of 205 healthcare professionals working in UK hospitals who have recruited adults with JIA to British Society for Rheumatology Biologics Register (BSRBR-RA) (response rate: 81 (39%) including rheumatology consultants (49), rheumatology specialist nurses (29), research nurses (2) and staff associates (1)), ascertained their experience in relation to four distinct areas: (1) education, training and practice; (2) factors suggesting a diagnosis of JIA; (3) use and choice of biologics; and (4) treatment challenges; and this was explored further through follow-up one-to-one telephone interviews (N = 10). The survey data was analysed using descriptive statistics and free-text comments using qualitative techniques; and the interview data was analysed qualitatively. This study had ethical approval with informed consent from all participants.

**Results:** Most (95%) survey participants currently treat adults with JIA and many (63%) also treat children. Most had not received any education or training in the specific management of adults with JIA (65%) although 56% had received some training in paediatrics (described as formal education, attendance at study days or meetings; in practice learning and team/colleague support). Many stated further need for training in JIA in adults (52%) and children (58%). Participants reported using all currently approved biologics for either JIA in children or rheumatoid arthritis; etanercept, adalimumab and tocilizumab being the most common. Factors influencing choice included: guidelines and funding; disease-associated factors; previous treatment; previous experience of clinician; and patient choice. Factors suggesting a diagnosis of JIA included: onset of symptoms before 16 years of age; antinuclear antibodies positivity; anterior uveitis; and other factors associated with disease presentation and pattern. The terminology used for adult JIA diagnosis varied and was influenced by disease associated factors; previous diagnosis; and preferred treatment plan. The challenges encountered in treating adults with JIA included: access to biologic therapy; disease awareness; access to support from colleagues; problems associated with transitional care services; and patient expectations. Potential improvements to aid the diagnosis and management of adults with JIA were offered and these included: the introduction of national guidelines; further education/training and support; and the introduction or improvement of transition service links.

**Conclusion:** Improved access to education and training as well as treatment guidelines relevant to the adult JIA context are likely to improve clinical experience and decision-making in relation to the management of adults with JIA.

**Disclosure of Interest**

None Declared.

### P215 Long-term effectiveness and safety of abatacept in juvenile idiopathic arthritis: interim results from the abatacept in JIA registry

#### Nicolino Ruperto^1^, Daniel J. Lovell^2^, Nikolay Tzaribachev^3^, Andrew Zeft^4^, Rolando Cimaz^5^, Valda Stanevicha^6^, Gerd Horneff^7^, John Bohnsack^8^, Thomas Griffin^9^, Ruy Carrasco^10^, Maria Trachana^11^, Jason Dare^12^, Ivan Foeldvari^13^, Richard Vehe^14^, Francesca Bovis^1^, Teresa Simon^15^, Alberto Martini^1^, Hermine Brunner^2^

##### ^1^Pediatria II, Reumatologia; PRINTO, Istituto Giannina Gaslini, Genoa, Italy; ^2^Cincinnati Children’s Hospital Medical Center, Cincinnati, USA; ^3^RHEKITZ - Rheumatologische Kinderpraxen Tzaribachev, Bad Branstedt, Germany; ^4^Cleveland Clinic, Cleveland, USA; ^5^Azienda Ospedaliero-Universitaria Meyer, Florence, Italy; ^6^Riga Stradins University, Riga, Latvia; ^7^Asklepios Klinik Zentrum für Allgemeine Paediatrie und Neonatologie, Sankt Augustin, Germany; ^8^University of Utah School of Medicine, Salt Lake City,USA; ^9^Levine Children's Hospital at Carolinas Medical Center, Charlotte, USA; ^10^Specially for Children, Austin, USA; ^11^Hippokration General Hospital, Thessaloniki, Greece; ^12^University of Arkansas Medical Center, Little Rock, USA; ^13^Hamburg Centre for Pediatric Rheumatology, Hamburg, Germany; ^14^University of Minnesota, Minneapolis, USA; ^15^Bristol-Myers Squibb, Princeton, USA

###### **Presenting author:** Nicolino Ruperto

**Introduction:** Abatacept (ABA) is a widely used biologic in children with juvenile idiopathic arthritis (JIA).

**Objectives:** The purpose of this study was to describe the longitudinal effectiveness and safety of ABA in JIA patients (pts).

**Methods:** Using a standardized protocol, clinical sites in the Pediatric Rheumatology Collaborative Study Group (PRCSG) and Paediatric Rheumatology International Trial Organization (PRINTO) enrolled JIA pts currently on or starting ABA in this longitudinal registry. Planned duration of follow-up is 10 years. Data shown are those collected through March 31, 2016 (up to 3 yrs follow-up).

**Results:** *Overview*. Of 315 JIA pts enrolled, 308 provided data for this. The total person-years (p-y) of observation were mean 231.9 y on ABA and 50.0 y post-ABA. In this registry, 35 (11%) of pts were new starts on ABA (≤1 mo of treatment), 207 (67%) had received ABA treatment for 1 mo–1 y, 52 (18%) for 1–2 y and 14 (4%) > 2 y. ABA was continued during follow-up in 224 pts (73%)

***Baseline.*** Of 308 pts, 246 (80%) were female, mean/median at enrollment was age: 13.2/13.8 y, disease duration: 5.4/4.4 y, and active joints: 2.7/0. Baseline clinical, functional and HRQoL scores are shown in Table 15 **:** In the treated pts reflecting real world use, 40 had a history of uveitis and 12 had active uveitis (16% history and active uveitis). JIA subtype was systemic 2%, oligoarticular 21%, polyarticular RF– 53%, polyarticular RF+ 10%, psoriatic 4%, enthesitis-related 4%, undifferentiated 6%. Of these pts, 265 (86%) were taking a concomitant medication for JIA (64% MTX, 49% NSAIDs, 17% systemic steroids, 5% leflunomide, 5% hydroxychloroquine, 1% cyclosporine, 1% sulfasalazine.)

***Follow-up safety***. A total of 30 AEs were seen (18 serious; all single occurrences) in 25 pts (0.8% of study population) for an AE rate 12.9 per 100 p-y of exposure (95% CI 8.8-18.2). There were 12 infections of special interest (5.0/100 p-y, 95% CI 2.8-8.8); 2 discontinued ABA due to a safety event (anaphylaxis). No new autoimmune diseases, deaths, malignancies or tuberculosis cases were reported.

**Conclusion:** In this JIA cohort with low MD Global, low number of active joints and with CID seen in above 30% at baseline, abatacept demonstrated persistent effectiveness over 2 years. Abatacept was well tolerated and no new safety signals were seen.

**Disclosure of Interest**

N. Ruperto Grant / Research Support from: The G. Gaslini Hospital, which is the public Hospital where I work as full time public employee, has received contributions from the following industries for the coordination activity of the PRINTO network: BMS, GlaxoSmithKline (GSK), Hoffman-La Roche, Novartis, Pfizer, Sanofi Aventis, Schwarz Biosciences, Abbott, Francesco Angelini S.P.A., Sobi, Merck Serono. This money has been reinvested for the research activities of the hospital in fully independent manners besides any commitment with third parties., Speaker Bureau of: I received speaker’s bureaus and consulting fees from the following pharmaceutical companies: AbbVie, Amgen, Biogenidec, Alter, AstraZeneca, Baxalta Biosimilars, Biogenidec, Boehringer, BMS, Celgene, CrescendoBio, EMD Serono, Hoffman-La Roche, Italfarmaco, Janssen, MedImmune, Medac, Novartis, Novo Nordisk, Pfizer, Sanofi Aventis, Servier, Takeda, UCB Biosciences GmbH., D. Lovell Grant / Research Support from: National Institutes of Health, NIAMS, Consultant for: Astra-Zeneca, Bristol Meyers Squibb, AbbVee, Pfizer, Roche, Novartis, UBC, Forest Research Institute, Horizon, Johnson & Johnson, Biogen, Takeda, Genentech, Glaxo Smith Kline, Boehringer Ingelheim, Celgene, Jannsen, Speaker Bureau of: Genentech, N. Tzaribachev: None Declared, A. Zeft: None Declared, R. Cimaz: None Declared, V. Stanevicha: None Declared, G. Horneff Speaker Bureau of: Abbvie,Pfizer, Chugai Roche and Novartis, J. Bohnsack: None Declared, T. Griffin: None Declared, R. Carrasco: None Declared, M. Trachana: None Declared, J. Dare: None Declared, I. Foeldvari: None Declared, R. Vehe: None Declared, F. Bovis: None Declared, T. Simon Employee of: Bristol-Myers Squibb, A. Martini Grant / Research Support from: The G. Gaslini Hospital, which is the public Hospital where I work as full time public employee, has received contributions from the following industries for the coordination activity of the PRINTO network: BMS, GlaxoSmithKline (GSK), Hoffman-La Roche, Novartis, Pfizer, Sanofi Aventis, Schwarz Biosciences, Abbott, Francesco Angelini S.P.A., Sobi, Merck Serono. This money has been reinvested for the research activities of the hospital in fully independent manners besides any commitment with third parties., Speaker Bureau of: I received speaker’s bureaus and consulting fees from the following pharmaceutical companies: Abbvie, Boehringer, Celgene, CrescendoBio, Janssen, Meddimune, Novartis, NovoNordisk, Pfizer, Sanofi Aventis, Vertex, Servier., H. Brunner: None Declared

**Table 15 (abstract P215).** Follow-up effectiveness

Endpoints	Baseline n = 308	3 months n = 247	6 months n = 215	12 months n = 121	24 months n = 35
Clinical					
MD Global	1.9/1.0	1.46/1.0	1.49/0.5	1.13/0.5	0.89/0.5
CID,^1^%	33	32	38	50	37
JAMAR Functional^2^	5.4/3.0	4.8/3.0	4.3/2.0	3.6/0.5	2.7/2.0
JAMAR HRQoL	6.9/6.0	6.0/5.0	5.6/4.0	4.9/3.0	5.1/4.0

Data are mean/median unless otherwise indicated

*CID* clinical inactive disease (Wallace criteria), *JAMAR Functional* Juvenile Arthritis Multidimensional Assessment Report Functionality Scale Child (range 0–15), *JAMAR HRQoL* Juvenile Arthritis Multidimensional Assessment Report HRQoL Scale Child (range 0–15), *MD Global* MD Global Disease Activity (VAS 0–10), *VAS* visual analog scale

1. Wallace C, et al. *Arthritis Care Res* 2011;**63**:929–36.

2. Filocamo G, et al. *J Rheumatol* 2011;**38**:938–53.

### P216 Disease activity states, reasons for discontinuation and adverse events in 1038 Italian children with juvenile idiopathic arthritis treated with etanercept

#### S. Verazza^1^, S. Davì^1^, A. Consolaro^1^, A. Insalaco^2^, V. Gerloni^3^, R. Cimaz^4^, F. Zulian^5^, S. Pastore^6^, F. Corona^7^, G. Conti^8^, P. Barone^9^, M. Cattalini^10^, E. Cortis^11^, L. Breda^12^, A. N. Olivieri^13^, A. Civino^14^, R. Podda^15^, D. Rigante^16^, F. La Torre^17^, G. D'Angelo^18^, M. Jorini^19^, R. Gallizzi^20^, M. C. Maggio^21^, R. Consolini^22^, A. De Fanti^23^, M. G. Alpigiani^1^, A. Martini^1^, A. Ravelli^1^ and on behalf of Italian Pediatric Rheumatology Study Group

##### ^1^Istituto Gaslini, Genova, Italy; ^2^Ospedale Bambin Gesù, Roma, Italy; ^3^Istituto Pini, Milano, Italy; ^4^Ospedale Meyer, Firenze, Italy; ^5^Ospedale Salus Pueri, Padova, Italy; ^6^Ospedale Burlo Garofalo, Trieste, Italy; ^7^Ospedale Maggiore Policlinico, Milano, Italy; ^8^Policlinico, Messina, Italy; ^9^Policlinico, Catania, Italy; ^10^Clinica Pediatrica, Brescia, Italy; ^11^Ospedale di Orvieto, Orvieto, Italy; ^12^Policlinico, Chieti, Italy; ^13^Seconda Università, Napoli, Italy; ^14^Ospedale Panico, Tricase, Italy; ^15^Ospedale Regionale Microcitemia, Cagliari, Italy; ^16^Policlinico Gemelli, Roma, Italy; ^17^Ospedale Perrino, Brindisi, Italy; ^18^Ospedale delle Marche, Ancona, Italy; ^19^Ospedale Salesi, Ancona, Italy; ^20^Policlinico Martino, Messina, Italy; ^21^Ospedale dei Bambini, Palermo, Italy; ^22^Ospedale Santa Chiara, Pisa, Italy; ^23^Ospedale S.Maria Nuova, Reggio Emilia, Italy

###### **Presenting author:** S. Verazza

**Introduction:** The advent of biologic medications has increased considerably the potential for treatment benefit in juvenile idiopathic arthritis (JIA), with clinical remission being now achievable in a substantial proportion of patients. However, there is a need of data from the real world of clinical practice to evaluate thoroughly the efficacy and safety profile of the biologic agents currently approved.

**Objectives:** To evaluate the outcome of etanercept (ETN) therapy in Italian children with JIA.

**Methods:** This is a multicenter, observational study that includes all children with JIA who were given ETN at Italian pediatric rheumatology centers after January 2000. Based on the status of ETN therapy at study start, patients were classified in 2 groups: patients still receiving ETN (Group 1); patients discontinued from ETN (Group 2). Patients in Group 1 underwent both retrospective and cross-sectional assessments, patients in Group 2 only retrospective assessments. Outcome of ETN therapy was assessed as follows: a) in Group 1, by evaluating the state of disease activity at cross-sectional visit through formal definitions or JADAS10 and cJADAS10 cut-offs; b) in Group 2, by evaluating reasons for ETN discontinuation. ETN-related side effects were recorded in both groups.

**Results:** A total of 1038 patients, 422 in Group 1 and 616 in Group 2 were enrolled in the study. Median treatment duration was 2.5 years in Group 1 and 2.6 years in Group 2. In group 1, the frequency of inactive disease (ID) by Wallace criteria was 41.8% and the frequency of low (or minimal) disease activity (LDA) by Magni-Manzoni criteria was 63.6%. The frequency of ID, LDA, moderate disease activity, and high disease activity on JADAS10 was 46.4%, 17.5%, 24.4%, and 11.6%, respectively. The frequency of the same disease activity states on cJADAS10 was 48.6%, 9.7%, 27.7%, and 14%. In Group 2, reasons for ETN discontinuation included disease remission (52.4%), lack of efficacy (29%), and side effects (21.4%).Serious adverse events were seen in 18 patients of the entire study population and included inflammatory bowel disease (10 pts), tuberculosis (1 pt), varicella complicated by purpura fulminans (1 pt), CMV hepatitis (1 pt), acute pancreatitis (1 pt), papilledema (1 pt), bladder carcinoma (1 pt), thyroid carcinoma (1 pt); 1 patient died of streptococcal sepsis.

**Conclusion:** Around half of the patients treated with ETN achieved clinical remission and another 10-15% had low disease activity. Serious adverse events were rare and were mostly represented by the development of inflammatory bowel diseases.

**Disclosure of Interest**

None Declared.

### P217 Successful treatment refractory pediatric takayasu's arteritis with anti-interleukin 6 receptor monoclonal antibody (tocilizumab): a case report

#### Betul Sozeri^1^, Aysenur Pac Kısaarslan^1^, Zubeyde Gunduz^1^, Ruhan Dusunsel^1^, Ismail Dursun^1^, Hakan Poyrazoglu^1^

##### ^1^Pediatric Rheumatology, ERCIYES UNIVERSITESI TIP FAKULTESI HASTANESI, Kayseri, Turkey

###### **Presenting author:** Betul Sozeri

**Introduction:** The goals of medical therapy of patients with Takayasu arteritis are to control active inflammation and to normalize clinical and laboratory parameters while preventing further vascular damage. Corticosteroids and conventional immunosuppressive agents are not always safe or efficacious. The complex formed by interleukin-6 (IL-6) and soluble IL-6 receptor appears to play a pivotal role in the pathogenesis of TA.

**Objectives:** Herein, we report a child with TA to share the efficacy and safety of tocilizumab.

**Methods:** Case report

**Results:** A- 14-year old girl who fulfilled the classification criteria for TA was identified. The interval from first symptom onset to diagnosis was 7 months. PVAS at presentation was 13; DEI.Tak was 10 and ITAS2010 was 10. She consisting of thickening of the aortic arch, descending aorta and superior mesenteric artery wall, obstruction of the right main carotid artery and superior mesenteric artery failed to respond to corticosteroids, methotrexate, and cyclophosphamide. After 1 year, she had severe manifestations (blurred vision and severe headache) and elevated both CRP and ESR the disease, and stenosis of the left vertebral artery in MRI angio which was considered as relapse. The patient started TCZ infusions (8 mg/kg for 2 weeks), and a rapid clinical remission was observed, associated with a drastic reduction of inflammatory markers. Corticosteroids were withdrawn, the patient's weight and height improved. 2 months later, TCZ infusions were extended, with no significant side effects. Ischemic manifestations resolved, and new lesions were not observed in MRI during 8 months on TCZ. At the last follow-up, PVAS was 0, ITAS2010 was 0 and DEI.Tak was 0. ESR was 5 mm/hr, and CRP was 3 mg/dL.

**Conclusion:** TA in children is a rare but potentially life-threatening condition. The treatment of TA in children is weak, it is essential to treat TA aggressively as soon as the diagnosis is secured to reduce mortality and morbidity. TCZ appears to be effective in the management of patients with TA, in particular in patients refractory to corticosteroids and/or conventional immunosuppressive drugs.

**Trial registration identifying number:** -

**Disclosure of Interest**

None Declared.

### P218 Efficacy of rituximab in childhood-onset systemic lupus erythematosus: a retrospective study of 16 patients

#### Ekaterina Kuchinskaya, Farida Abduragimova, Mikhail Kostik

##### SAINT-PETERSBURG STATE PEDIATRIC MEDICAL UNIVERSITY, Saint-Petersburg, Russian Federation

###### **Presenting author:** Ekaterina Kuchinskaya

**Introduction:** Systemic lupus erythematosus in children (juvenile-onset SLE, jSLE) has an unpredictable course and a more severe phenotype compared to adults. There are no standards for treatment of jSLE now. Several studies of B-cell targeted therapy with rituximab (RTX) in adults have demonstrated its high efficiency in various rheumatic diseases. Robust data on the use of rituximab in jSLE are still lacking and dependent on small cohort studies.

**Objectives:** To evaluate the efficacy and safety of rituximab in children with jSLE.

**Methods:** Our retrospective study includes 16 children with diagnosis of jSLE who were treated by RTX at Saint-Petersburg State Pediatric Medical University. Onset age of jSLE ranged from 5 to 16 years. RTX was administered at a dose of 375 mg/m2 weekly for 4 weeks. Repeated courses were in 10/16 patients. Interval between the RTX courses usually was 6-12 months, depended on disease activity and presence of side effects of corticosteroids. Indications for RTX included severe life-threatening jSLE course, low efficiency of traditional treatment with corticosteroids and immunosuppressive drugs, severe side effects of corticosteroids and/or impossibility tapering corticosteroids’ dose. We assessed disease activity using immunological markers (the ANA titer, antidsDNA antobodies level, lymphocyte subtypes), urine protein loss, serum creatinine, urea and c-reactive protein levels, complete blood count, oral daily corticosteroid dosage, and activity indexes included SLEDAI, ECLAM and Pediatric SLICC/ACR Damage Index measured before and after treatment. Descriptive statistics were reported in terms of medians and interquartile ranges (IQRs) for continuous variables and in terms of absolute frequencies and percentages for categorical variables. Statistical analysis was performed with Wilcoxon and Friedman tests.

**Results:** During RTX course statistically significant improvement of disease activity markers was registered: median SLEDAI decreased from 12 (6-26) to 2 (0-4); p = 0,001, ECLAM from 3 (1,5-5,5) to 1 (0-1); p = 0,002, SLICC/ACR Damage Index from 1 (0,5-3) to 1 (0-1); p = 0,015, anti-dsDNA levels from 126,6 IU/l (21-214) to 14 IU/l (1-60); p = 0,046, oral corticosteroid dosage from 0,9 (0,2-1,1) to 0,17 (0,1-0,25) mg/kg; p = 0,0015 and from 33,75 (7,5-50,0) to 10,0 (7,5-13,75) mg/day; p = 0,0018, CD19 count from 26% (11-35,2) to 2% (0,3-7,7); p = 0,04. During the course of RTX only 3 SAE were noted: one case of pneumonia and two patients died due to initial severity of disease and generalized mycosis as complication.

**Conclusion:** Rituximab is an effective and relatively safe therapeutic option in children with severe refractory jSLE

**Disclosure of Interest**

None Declared.

### P219 Is cytokine profiling of value for choice of biologic treatment in Ghosal Hematodiaphysal dysplasia?

#### Erik Sundberg^1^, Soley Omarsdottir^2^, Lena Klevenvall^1^, Helena Erlandsson-Harris^3^

##### ^1^Women´s and Children´s Health, Karolinska Institutet, Stockholm, Sweden; ^2^Paediatric Rheumatology unit, Karolinska Hospital, Stockholm, Sweden; ^3^Department of Medicine, Rheumatology unit, Karolinska Institutet, Stockholm, Sweden

###### **Presenting author:** Erik Sundberg

**Introduction:** Ghosal Hematodiaphysal dysplasia (GHDD) is a rare disease associated with mutation in the TBXAS1 gene encoding thromboxane synthase. It is characterized by pronounced anemia, increased bone density, hypo cellular bone marrow, diaphyseal and metaphyseal widening and splenomegaly. Corticosteroids have been described as a suitable treatment. A 7-years old boy was admitted to the Paediatric Rheumatology unit due to pain in the extremities. He had earlier been evaluated for iron therapy resistant anemia with moderately increased ESR. Pain was recorded as moderate to severe, leading to significant disability together with tiredness. The diagnosis of GHDD was confirmed by the clinical presentation, typical X-ray abnormalities and mutation of TBXAS1. Per oral steroids was effective in reducing pain and disability and improved anemia to some extent. ESR was normalized as a result of treatment.

**Objectives:** The patient responded well to p.o. steroid therapy although it was deemed as an unsuitable long term solution due to its known adverse effects. TNF blockade by etanercept, was then evaluated as a steroid sparing drug. Etanercept proved to be unsufficient for this purpose since symptoms, primarily pain, markedly increased when tapering p.o. steroids. Thus, our aim was to rapidly identify an effective pharmacological therapy strategy with minor adverse effects that could replace p o steroids by investigating the patients’ cytokine profile.

**Methods:** Blood sampling was performed during a treatment free period, before p.o. steroids treatment initiation. Blood was also collected in parallel from two healthy controls. Fresh whole blood was stimulated with LPS (1 ng/ml) for 4 h together with ATP (3 mM) for 1 hour. ATP was added since low dose LPS is not sufficient to induce IL-1b release alone. Supernatants were collected after centrifugation in 10 min 1000 g and stored in -80 °C until analysis. IL-1b, IL-6, IL-8 and TNF levels were recorded with Cytometric Bead Array (CBA) according to manufactures instructions.

**Results:** TNF was not detectable in the unstimulated patient sample nor in the unstimulated healthy control samples. LPS/ATP stimulation induced lower TNF production in the patient as compared to the healthy controls (1144 pg/ml vs 1867, 3531 pg/ml). A similar pattern was evident for IL-1b production, with no detectable levels in unstimulated samples and lower levels in the LPS/ATP stimulated patient sample (11 461 pg/ml) as compared to the healthy controls (18 786, 25 508 pg/ml). The IL-6 level in the unstimulated patient sample was low (17 pg/ml), as in the control samples (7, 3 pg/ml). LPS/ATP stimulated samples had similar IL-6 levels in patients and controls, with slightly higher levels in patients (11 986 pg/ml) than in controls (8 893, 10 122 pg/ml). Interestingly IL-8 production was increased in the untreated patient sample (1153 pg/ml) as compared to controls (162, 324 pg/ml). Furthermore, LPS/ATP stimulation resulted in markedly increased IL-8 release in the patient sample (13 007 pg/ml) as compared to controls (4 473, 5 830 pg/ml).

**Conclusion:** Cytokine profiling may prove important for characterizing cytokine patterns in single patients with rare inflammatory diseases. Our patient was hypo responsive to LPS/ATP stimulation as compared to two healthy controls with regard to TNF and IL-1b production. In contrast, our patient was hyper responsive with regard to IL-8 production upon stimulation and also exhibited elevated IL-8 production in the unstimulated sample. Thus we are now aiming to institute IL-8 targeted therapy, once available, and are in the meantime opting for IL-6 blockade rather than IL-1 blocking therapy.

**Disclosure of Interest**

None Declared.

### P220 Effectiveness of etanercept release system with microspheres in rat model of arthritis

#### Gokalp Basbozkurt^1^, Ozge Erdemli^2^, Dogan Simsek^3^, Fatih Yazici^4^, Yildirim Karsioglu^5^, Aysen Tezcaner^2^, Dilek Keskin^2^, Huseyin Ozkan^6^, Cengizhan Acikel^4^, Seza Ozen^7^, Erkan Demirkaya^3^

##### ^1^Pediatrics, Gulhane Military Medical Academy, Ankara, Turkey; ^2^Engineering Sciences, Middle East Technical University, Ankara, Turkey; ^3^Pediatric Rheumatology, Gulhane Military Medical Academy, Ankara, Turkey; ^4^Institute of Health Sciences, Gulhane Military Medical Academy, Stockholm, Ankara; ^5^Pathology, Gulhane Military Medical Academy, Ankara, Turkey; ^6^Orthopedics and Traumatology, Gulhane Military Medical Academy, Ankara, Turkey, ^7^Pediatric Rheumatology, Hacettepe University, Ankara, Turkey

###### **Presenting author:** Gokalp Basbozkurt

**Introduction:** The utilize of delivery systems such as liposomes and polymeric microspheres has been proposed to prolong the exposure of the drug in the joint cavity. Various polymeric materials in different drug delivery systems have been investigated to retain the drug efficacy within the joint. Drug loaded microspheres were directly injected into the joints and demonstrated significant a local anti-inflammatory effect.

**Objectives:** In this study, we aimed to investigate the in-vivo performance of single intra-articular (IA) treatment of etanercept (ETN)-loaded policaprolacton (PCL) and ETN-loaded polyethylene glycol-PCL (PEG-PCL-PEG) microspheres and to compare the IA treatment groups with systemic single dose of ETN in adjuvant induced arthritis rat model.

**Methods:** Animal experiments were carried out with 120 male Sprague-Dawley rats, weighting 200 and 250 g at the Gulhane Military Medical Academy,Turkey.

Rats were divided into seven main groups which were as follows:

· Saline Injected (negative control) (n = 8)

· Complete Freund’s Adjuvant (CFA) injected (positive control) (n = 24)

· Empty PCL microsphere injected (empty microsphere 1) (n = 16)

· Empty PEG-PCL-PEG microsphere injected (empty microsphere 2) (n = 16)

· ETN-loaded PCL microsphere injected (IA Treatment 1) (n = 16)

· ETN-loaded MPEG-PCL-MPEG microsphere injected (IA Treatment 2) (n = 16)

· Intra-peritoneal ETN injected (Systemic Treatment) (n = 24)

Arthritis was induced with injection of 0.15 ml CFA into the right knee joint cavity in all groups except negative control group. Chronic arthritis was proofed by clinical findings such as swelling, pain, limitation of motion at 3 weeks after IA CFA injection.

Rats from negative control (n = 8) and positive control (n = 24) groups were sacrificed after injection of IA saline and CFA each time periods at 3 weeks (n = 3, n = 8), 8 weeks (n = 3, n = 8), and 12 weeks (n = 2, n = 8). Additionally, in each time period empty and ETN-loaded PCL and PEG-PCL-PEG microspheres injected groups (n = 64) (8 and 12 weeks after IA injection), and also systemic treatment groups (n = 24) (2, 8, 12 weeks after IA injection) was sacrificed. Knee joints were dissected, freed from muscles and removed. Histopathological examination was carried out by a single (blinded) observer, focusing mainly on polymorphonuclear cell infiltration, tissue proliferation, and cartilage deterioration. The severity of the inflammation was graded into four grades: 0 = no change; 1 = mild change; 2 = moderate change, and 3 = serious change.

**Results:** There was no arthritis in saline injected group. Experimental arthritis was occurred in all CFA injected groups. It was found various grade (Grade1-3) inflammation findings in histopathological evaluation. Both ETN release systems significantly decreased inflammation degrees in rats with chronic arthritis. However, it was found difference between PCL and PEG-PCL-PEG of etanercept release systems. PCL and PEG-PCL-PEG release systems were indicated effective as anti-inflammatory 8 weeks and 12 weeks, respectively.

**Conclusion:** When we used etanercept with the release systems of microspheres, we found that the effectiveness of etanercept were lasting 8 weeks with PCL, and 12 weeks with PEG-PCL-PEG. Our study also showed that TNF-alpha inhibitor release systems had anti-inflammatory effects in experimental arthritis rat model.

**Disclosure of Interest**

None Declared.

### P221 Long- term folow-up on efectiveness and safety of biological treatment in patients with juvenile idiopathic arthritis: Hungarian data from the national institute of rheumatology and physiotherapy, registry

#### Ilonka Orbán^1^, Krisztina Sevcic^1^, Valentin Brodszky^2^, Emese Kiss^1^

##### ^1^Department of Clinical Immunology, Adult and Paediatric Rheumatology, National Institute of Rheumatology and Physiotherapy, Budapest, Hungary; ^2^Department of Health Economics, Corvinus University of Budapest, Budapest, Hungary

###### **Presenting author:** Ilonka Orbán

**Introduction:** For the most common chronic paediatric rheumatic disease, Juvenile Idiopathic Arthritis (JIA), the biological treatment was a reverberating success for the disease management and improved the quality of life of the patients.

**Objectives:** To report the efficacy and safety of biological treatment in a cohort of patients with JIA under biological treatment in a single paediatric rheumatologic centre, in a prospective observational study.

**Methods:** The JIA-ILAR criteria were used for the diagnosis. Patients with JIA polyarticular course, who failed to respond or did not tolerate methotrexate, and were treated with biological agents at the Paediatric Rheumatologic Centre of the National Institute of Rheumatology and Physiotherapy, Budapest, Hungary, from 2002-2015 were enrolled in an open label observational study. At baseline patients and disease characteristics were registered. Disease activity was studied before the start of the treatment and after every three months in the first year of treatment, then every six months in the second year and then yearly, according to the JIA core set of the ACR paediatric definition of improvement (ACR Pedi). Adverse events (AEs), reason for the switch to the other biologic treatment were documented.

**Results:** In this study 137 Caucasian JIA patients were evaluated, from the 416 registered patients treated with biologic at the submission. Gender: female, n(%): 108(79%), mean current age of patients: 20,5 years (y)(2,5-49y). JIA subtypes were represented: 2,9% systemic; 7,2% polyarticular RF positive; 58,39% polyarticular RF negative; 7,29% oligoarticular extended; 8%, enthesitis related; 14,5% arthritis psoriatica and 1,4% other forms. Mean disease duration was: 19,5y (1-29 y). The initially biologic percentage of patients were: 75,1% etanercept; 21,1% adalimumab; 2,1% infliximab; 1,4% tocilizumab. The percentage of patients with switched therapy to one or more: 18,2% (to adalimumab: 8, to abatacept: 4, to tocilizumab: 8, to etanercept: 2, to infliximab: 2, to canakinumab:1 patient). Reason of the therapy was inefficacity or intolerance to the previous drug. All disease activity parameters improved significantly in the first three months of the treatment. The statistically interpreted data (110 patients) suggest a significant success in the treatment of JIA patients on biological treatment after one and two years in the mean value of six response variables of the JIA core set: (i) number of active joints (joints with swelling not caused by deformity, or joints with limited motion and with pain, tenderness or both): 12/2/1; (ii) number of joints with limited motion: 17/6/6; (iii) overall assessment of disease activity by the physician through the visual analogue scale (VAS) (range 0–100 mm): 53/23/22; (iv) overall assessment of well-being by the patient or the parents of the patient through the VAS (range 0–100 mm): 50/22/19; (v) ESR: 22/13/13 mm/h; (vi) Hungarian version of childhood HAQ (CHAQ) (range 0–3 in each items): 0,98/0,65/0,50 and they keep these results. Severe adverse events were uncommon.

**Conclusion:** Biological treatment throughout several years is very effective and safe treatment for JIA patients. Sometimes we have to try more agents for effective therapy. More knowledge of the pathogenesis of JIA is needed to help us in choosing the best agent.A Paediatric rheumatology centre in an adult Rheumatology centre may be a god resolution for JIA patients transitioned to adulthood.

**Disclosure of Interest**

None Declared.

### P222 Hydrogel forming microneedles a promising mimimally invasive tool to deliver clinically relevant doses of methotrexate to treat paediatric rheumatoid arthritis

#### Ismaiel A. Tekko^1^, Madeleine Rooney^2^, James McElnay^1^, Cliff Taggart^2^, Helen McCarthy^1^, Ryan F. Donnelly^1^ and Drug Delivery Group

##### ^1^School of Pharmacy, Queen`s University Belfast, Belfast, UK; ^2^Centre for Infection and Immunity, Queen`s University Belfast, Belfast, UK

###### **Presenting author:** Ismaiel A. Tekko

**Introduction:** Methotrexate (MTX) is the only disease modifying drug used in the treatment of Rheumatic Arthritis in children, other than biologics. However its oral absorption is variable, and nausea and vomiting frequent (which is believed it is due to high bolus drug peaks in blood after drug administration), resulting in discontinuation. Regular subcutaneous injections are painful and very stressful for children causing considerable anxiety. Transdermal delivery is an attractive alternative delivery route and can help in avoiding the above mentioned drawbacks. However, skin form a fierce barrier for delivery of such hydrophilic drug.

**Objectives:** To design and develop a hydrogel forming microneedles (MN) and evaluate their efficiency as a minimally invasive drug delivery system in delivering clinically relevant doses in sustained controlled manner in-vitro.

**Methods:** Hydrogel-forming MN array was fabricated from a hydrogel composed of 20% w/w copolymers of (methyl vinyl ether and maleic acid) and 7.5%w/w poly ethylene glycol (PEG, MW 10,000 Da) by casting method using silicon mould with an array of 121 (11 x11) microneedles. The formed MN arrays were characterised for their mechanical strength, swelling properties, penetration depth after insertion into isolated cadaver full-thickness pig skin. Rapidly dissolving polymeric films containing various concentrations of MTX was prepared and tested. The ability of MN combined with MTX-containing films to deliver MTX was evaluated in-vitro using dermatomed pig skin.

**Results:** Hydrogel forming MN was formed measuring (729.5 ± 11.2 um) in height and 300 um width at the baseplate and MN interspacing 250-300 um. The fabricated MN were strong enough to pierce the stratum corneum and penetrate through the skin without breaking or bending by applying 20 N/MN array and removed intact without depositing any polymer in the skin. The penetration depth was around 72% of the MN length. These MN can swell without dissolving and the swelling percentage was around 300% of its original weigh after 1 hour from immersion in phosphate buffer saline (pH 7.4). The fabricated MN were able to deliver various substantial amounts of MTX in a sustained and controlled and manner.

**Conclusion:** hydrogel forming MN formed from 20% w/w copolymers of (methyl vinyl ether and maleic acid) and 7.5%w/w poly ethylene glycol (PEG, MW 10,000 Da) can be used as a minimally invasive drug delivery system to deliver clinically relevant various doses of MTX in a sustained control manner and without depositing any polymer residue in the skin after application.

**Acknowledgment**

This Research project is kindly supported by a grant from ARUK.

**Disclosure of Interest**

None Declared.

### P223 Allogeneic haematopoietic stem cell transplantation (HSCT) in children with severe, refractory rheumatic disorders

#### Mario Abinun^1^, Mary Slatter^2^, Zohreh Nademi^2^, Mark Friswell^3^, Helen Foster^3^, Sharmila Jandial^3^, Flora McErlane^3^, Terence Flood^1^, Sophie Hambleton^1^, Andrew Gennery^1^, Andrew Cant^1^

##### ^1^Paediatric Immunology, Great North Children's Hospital, Newcastle upon Tyne, UK; ^2^Children's BMT Unit, Great North Children's Hospital, Newcastle upon Tyne, UK; ^3^Paediatric Rheumatology, Great North Children's Hospital, Newcastle upon Tyne, UK

###### **Presenting author:** Mario Abinun

**Introduction:** In spite of unprecedented effects of biologic disease modifying antirheumatic drugs (DMARDs) over the last 15 years in treating childhood rheumatic and autoinflammatory disorders, a small minority still remain refractory to multiple, combined immunosuppressive and anti-inflammatory therapies, whilst accumulating serious side effects.

**Objectives:** Present results from our centre of curative allogeneic haematopoietic stem cell transplantation (HSCT) in a group of children with such severe, refractory diseases.

**Methods:** Data were collected for 13 children referred for HSCT in the period 2006-2016 – 8 with rheumatic disorders (juvenile idiopathic arthritis (JIA) 4, systemic lupus erythematosus (SLE) 3, paniculiitis 1, and 5 with autoinflammatory diseases (early onset colitis (EOC) 2, 1 each with mevalonic kinase deficiency (MKD), congenital sideroblastic anaemia, B cell immunodeficiency, periodic fevers, developmental delay (SIFD), and hypercalprotectinaemia/hyperzinkaemia (PAPA-like)).

**Results:** 12/13 (age 1-17 yrs at HSCT) are alive, 8 long-term survivors (follow-up 1-9 yrs) with markedly improved quality of life and/or cured. Transplant-related complications were graft-versus-host disease (GvHD) (1/13 significant), virus reactivations (6/13), 2nd autoimmune disease post-HSCT (5/12 survivors), and 1 child (with EOC) died from multi-organ failure.

**Conclusion:** Our outcome results of allogeneic HSCT for this small cohort of patients with otherwise high morbidity are very encouraging (1,2). Further studies on larger groups of selected patients with longer follow-up are now needed for more objective evaluation of this potentially curative treatment (3).

**References**

1. Silva J et al. Allogeneic haematopoietic stem cell transplantation for systemic onset juvenile idopathic arthritis. Biol Blood Marrow Transplant 2015;21:s46 (Abs No 25)

2. Abinun M et al. Allogeneic haematopoietic stem cell transplantation in children with severe, refrctory rheumatic diorders - single centre outcome 2006-2015. Cln Exp Immunol 2015;182(S1):8 (Abs No 11)

3. Abinun M. Haematopoietic stem cell transplantation for rheumatological cnditions. Paediatrics Child Health 2011;21(12):558-62

**Disclosure of Interest**

None Declared.

### P224 Assessing the clinical relevance and risk minimization of antibodies to biologics in juvenile idiopathic arthritis (JIA) (ABIRISK). Baseline characteristic and 6 months preliminary data of children treated with etanercept, adalimumab or tocilizumab

#### Martina Finetti, Francesca Bovis, Joost Swart, Pavla Doležalová, Elena Tsitsami, Maria Trachana, Erkan Demirkaya, Phoi-Ngoc Duong, Isabelle Koné-Paut, Olga Vougiouka, Denise Pires Marafon, Rolando Cimaz, Giovanni Filocamo, María Luz Gamir, Valda Stanevicha, Helga Sanner, Laura Carenini, Nico Wulffraat, Alberto Martini, Nicolino Ruperto

##### ^1^Pediatria II, Reumatologia; PRINTO, Istituto Giannina Gaslini, GENOA, Italy

###### **Presenting author:** Martina Finetti

**Introduction:** ABIRISK is a project funded by Innovative Medicine Initiative (IMI), which has the aim to investigate anti-drug antibody formation in the treatment of JIA with biologics. A major limitation to the use of biologics is the development of anti-drug antibodies (ADA) in a subset of patients that may decrease the efficacy of biologics by neutralizing them or modifying their clearance.

**Objectives:** The aim of this project is to improve the capability to predict biologics immunogenicity and to minimize immunization risk against biological drugs in juvenile idiopathic arthritis (JIA) patients. We present herein the baseline and 6-month data of the JIA patients enrolled in the study.

**Methods:** Patients with juvenile idiopathic arthritis (JIA by ILAR criteria) followed by 21 PRINTO centres in 11 countries were prospectively enrolled and treated with anti-TNFα or anti-IL6 drugs (Etanercept, Adalimumab or Tocilizumab). Patient’s data were obtained from the Pharmachild registry, a pharmacovigilance data repository for children with JIA treated with methotrexate (MTX) ± biologics. For each patient detailed clinical information and biologic samples (serum and RNA) were collected before therapy start and at periodic visits during biologics regimen until 18 months of follow-up.

**Results:** 116 patients were included in this analysis. Three patients were considered twice because treated with two different sequential biologics. 82/116 (71%) was females, the median onset age was 5.4 years (range 2.4-10.3) and the disease duration at enrolment was 2.6 years (range 1.1-6.2). Distribution of JIA subtypes was as follows: 8 systemic (7%), 30 (26%) oligoarthritis persistent, 49 (42%) polyarthritis or extended oligoarthritis, 6 (5%) polyarthritis RF+, 16 (14%) arthritis with enthesitis and 7 (6%) undifferentiated arthritis. Sixty-four patients (55%) started treatment with Adalimumab, 33 (28%) with Etanercept, and 19 (16%) with Tocilizumab. Disease activity of the enrolled patients (assessed with JADAS27) decreased significantly at 3 and 6 months after therapy start (p < 0.0001). (Table 16)

**Conclusion:** Preliminary data show an early statistically significant improvement of disease activity at 3 and 6-month after biologics treatment start. Analysis of collected samples and clinical related information will help to develop predictors of immunogenicity risk and to determine which patients will respond best to which biologic.

**Trial registration identifying number:** ClinicalTrials.gov Identifier: NCT02116504

**Disclosure of Interest**

M. Finetti: None Declared, F. Bovis: None Declared, J. Swart: None Declared, P. Doležalová Grant / Research Support from: AbbVie, Roche, Medac, Novartis, Pfizer, Speaker Bureau of: Pfizer, Novartis, Medac, E. Tsitsami: None Declared, M. Trachana: None Declared, E. Demirkaya: None Declared, P.-N. Duong: None Declared, I. Koné-Paut Speaker Bureau of: Pfizer, Abbvie and Chugai, O. Vougiouka: None Declared, D. Pires Marafon: None Declared, R. Cimaz: None Declared, G. Filocamo: None Declared, M. L. Gamir: None Declared, V. Stanevicha: None Declared, H. Sanner: None Declared, L. Carenini: None Declared, N. Wulffraat Grant / Research Support from: Novartis, Sobi, Pfizer, Roche, AbbVie, Speaker Bureau of: Novartis, Sobi, Pfizer, Roche, AbbVie, A. Martini Grant / Research Support from: The G. Gaslini Hospital, which is the public Hospital where I work as full time public employee, has received contributions from the following industries for the coordination activity of the PRINTO network: BMS, GlaxoSmithKline (GSK), Hoffman-La Roche, Novartis, Pfizer, Sanofi Aventis, Schwarz Biosciences, Abbott, Francesco Angelini S.P.A., Sobi, Merck Serono. This money has been reinvested for the research activities of the hospital in fully independent manners besides any commitment with third parties., Speaker Bureau of: I received speaker’s bureaus and consulting fees from the following pharmaceutical companies: Abbvie, Boehringer, Celgene, CrescendoBio, Janssen, Meddimune, Novartis, NovoNordisk, Pfizer, Sanofi Aventis, Vertex, Servier., N. Ruperto Grant / Research Support from: The G. Gaslini Hospital, which is the public Hospital where I work as full time public employee, has received contributions from the following industries for the coordination activity of the PRINTO network: BMS, GlaxoSmithKline (GSK), Hoffman-La Roche, Novartis, Pfizer, Sanofi Aventis, Schwarz Biosciences, Abbott, Francesco Angelini S.P.A., Sobi, Merck Serono. This money has been reinvested for the research activities of the hospital in fully independent manners besides any commitment with third parties., Speaker Bureau of: I received speaker’s bureaus and consulting fees from the following pharmaceutical companies: AbbVie, Amgen, Biogenidec, Alter, AstraZeneca, Baxalta Biosimilars, Biogenidec, Boehringer, BMS, Celgene, CrescendoBio, EMD Serono, Hoffman-La Roche, Italfarmaco, Janssen, MedImmune, Medac, Novartis, Novo Nordisk, Pfizer, Sanofi Aventis, Servier, Takeda, UCB Biosciences GmbH.

**Table 16 (abstract P224).** Measure of disease activity of the Abirisk patients entered into the Pharmachild registry. Observed data are presented as median (1st-3rd quartile).

	Baseline N = 116	Three Months N = 69	Six Months N = 41	p-value
JADAS27	11.5 (6.5-16)	2 (0.5-5)	1 (0-8)	<.0001
ESR mm/h	19 (9-37)	7.5 (5-15)	7 (5-16)	<.0001
CRP standardized	9.1 (2-37.5)	2 (1-7.1)	2 (2-10)	0.0002
Nr of active joints	3 (1-6)	0 (0-1)	0 (0-0.5)	<.0001
Nr of LOM	2 (1-6)	0 (0-2)	0 (0-1.5)	<.0001
MDgloVAS	4 (3-6)	0.5 (0-1.5)	0 (0-1.75)	<.0001
VAS pain	3 (1-5)	0.25 (0-1.5)	0 (0-1.5)	<.0001
VAS well-being	3 (1-5)	0 (0-1.5)	0.5 (0-2.5)	<.0001

### P225 Effectiveness of mesenchymal stem cell therapy in experimental inflammatory arthritis and comparison with the other intra-articular treatments

#### Mesut Topdemir^1^, Gokalp Basbozkurt^1^, Yildirim Karslioglu^2^, Huseyin Ozkan^3^, Cengizhan Acikel^4^, Erkan Demirkaya^5^, Faysal Gok^5^

##### ^1^Pediatrics, Gulhane Military Medical Academy, Ankara, Turkey; ^2^Pathology, Gulhane Military Medical Academy, Ankara, Turkey; ^3^Orthopedics and Traumatology, Gulhane Military Medical Academy, Ankara, Turkey; ^4^Institute of Health Sciences, Gulhane Military Medical Academy, Ankara, Turkey; ^5^Pediatric Rheumatology, Gulhane Military Medical Academy, Ankara, Turkey

###### **Presenting author:** Mesut Topdemir

**Introduction:** Chronic arthritis is a clinical entity which is characterized with inflammatory joint involvement, affects life quality and may cause sequels without treatment. Mesenchymal stem cell (MSC) treatment is recently considered as an alternative treatment for chronic inflammatory diseases which are refractory to treatment due to their immunomodulatory, anti-inflammatory, and regenerative capacities.

**Objectives:** In this study, we aimed to evaluate the histopathological effects of MSC’s on synovial inflammation in rats that have experimental arthritis induced by adjuvants and compare the effectiveness of the treatment with intra-articular (IA) triamcinolone hexacetonide acid (TH) and IA etanercept treatment.

**Methods:** In this study, 48 Sprague-Dawley male rats were used. Experimental arthritis model was formed by IA administration of Complete Freund Adjuvant (CFA) into the rats’ right knee joint space. IA treatments were administered at 3rd week when the arthritis became chronic. A semi-quantitive grading system was used for detection of the arthritis severity. Histopathological examination was carried out by a single (blinded) observer, focusing mainly on polymorphonuclear cell infiltration, tissue proliferation, and cartilage weakening. The severity of the inflammation was classified into the four grades: 0 = no change; 1 = mild change; 2 = moderate change, and 3 = serious change.

**Results:** There were no statistically significant differences between the placebo group (IA saline group) and treatment group (sacrificed three weeks after IA MSC treatment). Placebo group was compared with the treatment group which sacrificed 6 weeks after IA MSC, a week after IA triamsolon and 2 weeks after IA etanercept treatment and statistically significant differences were detected.

**Conclusion:** In this study, IA TH treatment has found as the most efficient treatment in the chronic arthritis murine model. It was observed that MSC administration was efficient chronic inflammatory arthritis. Further investigations are needed for MSC sources, treatment intervals, administration methods and dosages.

**Disclosure of Interest**

None Declared.

### P226 Comparative efficacy methotrexate and sulfasalazine in patients with oligoarticular juvenile idiopathic arthritis

#### Elena Zholobova^1^, Nadezhda Tsurikova^2*^, Elena Ligostaeva^2^

##### ^1^Departament of Pediatric Rheumatology, First Moscow Medical State University I.M. Sechenov, Moscow, Russian Federation; ^2^Department of Pediatrics, Regional children's hospital, Rostov-on-Don, Russian Federation

###### **Presenting author:** Nadezhda Tsurikova

**Introduction:** Juvenile idiopathic arthritis(JIA) is a chronic autoimmune disease in children and adolescents with primary joints involvement and various manifestations. Treatment with methotrexate or sulfasalazine of early oligoarticular JIA represents a serious challenge.

**Objectives:** To compare efficacy and safety methotrexate and sulfasalazine treatment in children with early oligoarticular JIA.

**Methods:** It was the prospective observational study. 80 patients with oligoarticular JIA (the disease duration was less than 6 months) were included. They were not treated with DMARDs before. All patients were divided into two groups: the I-st group included patients who were treated with methotrexate 12.5-15 mg/m2/week (13.8 mg/m2/week): 25 girls and 15 boys; mean age was 4.9 years (4-12); disease duration was 3.1 months (2-6)). The II-nd group included patients who were treated with sulfasalazine 30-40 mg/kg/day (36 mg/kg/day): 26 girls and 14 boys; mean age was 6.7 years (4-13); disease duration was 3.4 months (2-6). Efficacy end points included the American College of Rheumatology (ACR) Pediatric criteria for improvement 30 (ACR30), ACR50, ACR70, ACR90 at 12 and 24 weeks of treatment.

**Results:** The ACR Pedi 30 improvement were achieved by 92.5% (n = 37) of patients in the I-st group at week 12 (n = 40). At week 12 3 (7.5%) patients were switched to sulfasalazine therapy (2 patients because of insufficient response, 1 - because of bad tolerance). The ACR Pedi 30, 50 and 70 improvement were achieved by 85% (n = 34), 85% (n = 34) and 10% (n = 4) of patients in the I-st methotrexate group at week 24 (n = 37), respectively. At weeks 24 2(5%) patients were also switched to sulfasalazine therapy, 1(2.5%) patient was prescribed anti-tumor necrosis factor alpha therapy. The ACR Pedi 30 improvement were achieved by 87.5% (n = 35) of patients in the II-nd group at week 12 (n = 40). At week 12 5 (12.5%) patients in the II-nd group were switched to methotrexate therapy because of absent ACRpedi-30 response. At weeks 24 3(7.5%) patients were also switched to methotrexate therapy because of absent ACRpedi-30 response and deterioration. The ACR Pedi 50 improvement was achieved by 80% (n = 32) of patients in the II-nd sulfasalazine group at week 24 (n = 35). There were no serious adverse events(SAE) in both groups.

**Conclusion:** According to the results of our research, treatment with methotrexate and sulfasalazine in children with early oligoarticular JIA has reliably sufficient efficiency, but treatment with methotrexate was more effective in comparison with treatment with sulfasalazine therapy. There were no SAE in both groups.

**Disclosure of Interest**

None Declared.

### P227 Blood monitoring in patients with JIA – a retrospective survey

#### Navdha R. Ramchurn^1*^, Mark Friswell^2^

##### ^1^Paediatric Medicine, Great North Children's Hospital, Newcastle upon Tyne, UK; ^2^Paediatric Rheumatology, Great North Children's Hospital, Newcastle upon Tyne, UK

###### **Presenting author:** Navdha R. Ramchurn

**Introduction:** Etanercept is licenced in the UK for use as monotherapy in JIA^1^. The manufacturer advises regular monitoring to detect clinically significant neutropaenia^2^. In our institution, this has meant checking the neutrophil count, alanine aminotransferase (ALT) and creatinine every 3 months.

**Objectives:** To determine the efficacy of regular blood test monitoring in Etanercept monotherapy in detecting clinically significant neutropaenia or liver function anomalies

**Methods:** We conducted a retrospective survey of our database of JIA patients at the Great North Children’s Hospital, a tertiary paediatric rheumatology centre in Newcastle, UK. All current patients were reviewed and those currently or previously taking Etanercept were identified. Data was collected on demographics, diagnosis, date Etanercept started/stopped & number of blood tests. Abnormalities in ALT or white cell count were recorded, including any resulting action. In addition we looked at the reasons for stopping Etanercept.

**Results:** 137 patients received Etanercept during the study period, with 155 distinct episodes of Etanercept therapy. 75% were female & median age was 9.7 years at the start of therapy. 56% had poly-articular JIA, 12% extended oligoarticular, 13% oligo-articular, 9% psoriatic, 7% ERA, & 4% systemic onset. There was no significant difference in demographics between those on monotherapy and those also on MTX.

72 patients were or had been taking Etanercept as monotherapy, with 77 distinct episodes of monotherapy. 5 episodes were excluded from further analysis as therapy had been started within 3 months with no test yet, leaving 72 episodes in 68 patients.

Of these 72 episodes, median duration of Etanercept monotherapy was 18 months (range 3- 85). Twenty-four had no detected neutropenia or abnormal ALT. A total of 31 (43%) abnormal results were detected with a median time from onset of treatment of 5 months (range 0-52). 19 patients had similar abnormalities on MTX therapy before starting Etanercept monotherapy and 12 had new onset abnormalities on Etanercept monotherapy. Eight developed neutropaenia, 5 were noted to have a raised ALT and 1 patient had both.

All these episodes were asymptomatic and resolved without any intervention. No patient had their Etanercept stopped due to abnormal blood results.

34 of the 72 patients on monotherapy had Etanercept discontinued during the study period. 9 had been in remission for >2 yrs, 9 developed Chronic Anterior Uveitis and were switched to an alternative Biologic, 7 were switched to an alternative Biologic for inefficacy, 5 had MTX added in, 4 stopped for other reasons including parental choice.

**Conclusion:** In our study Etanercept monotherapy is rarely associated with neutropaenia or deranged liver function and no patient had the treatment interrupted or discontinued as a result. It may be reasonable to consider the frequency of such monitoring given the distress and discomfort of regular venepuncture.

**References**

1. National Institute for Health and Care Excellence. Abatacept, adalimumab, etanercept and tocilizumab for treating juvenile idiopathic arthritis (TA373). 2015

2. Enbrel website. Available at: https://www.enbrel.com/hcp/. Accessed on 19/05/2016

**Disclosure of Interest**

None Declared.

### P228 Longterm experience of abatacept therapy in juvenile idiopathic arthritis in a single center

#### O. Kostareva^1^, I. Nikishina^1^, S. Arsenyeva^1^, S. Rodionovskaya^2^, M. Kaleda^1^, D. Alexeev^1^

##### ^1^Paediatric, V.A. Nasonova Research Institute of Rheumatology, Moscow, Russian Federation; ^2^Paediatric, Central Children Hospital FMBA, Moscow, Russian Federation

###### **Presenting author:** O. Kostareva

**Introduction:** The most complicated problem of Biologics therapy of juvenile idiopathic arthritis (JIA) in real clinical practice to do optimal choice for certain kind of disease.TNF-inhibitors are well studied in contrast to Abatacept (ABA).

**Objectives:** to evaluate in retrospective study results of longterm experience of ABA using in different JIA types and to determine the predictable features for good respond.

**Methods:** The study involved prospective cohort of JIA patients (pts) treated by ABA in our clinic from 2004 to 2016. Analyze included drug survival with Kaplan-Meier, reasons of withdrawals and adverse event (AE) rates.

**Results:** 104 pts with different JIA types were included in prospective cohort study.At the start of ABA pts average age was 11.1 ± 3.7 years (range 3.1-17.9), mean disease duration initiation was 5.6 ± 3.7 yrs (range 0.3-15.8). JIA subtypes were as follows: seronegative polyarthritis (Poly-RFneg) - 68(65%), seropositive polyarthritis (Poly-RFpos) - 20(19%), oligoarthritis persistent (Olygo) 7(7%), systemic arthritis without current systemic manifestation (soJIA) - 9(9%). 24(23%) had uveitis; mean disease duration before uveitis was 3.4 ± 3.4 yrs (range 0.17-12.5). ABA used as first-line biological agent in 86(82.69%), other line - 18 (17.3%).Inactive disease (ID) status was achieved in 62 (60%) cases, more often in biologics-naive patients - 86 (83%) vs 18 (17%), previously treated with other biologics. Frequency of ID achievement was higher in Poly-RFpos, Oligo and JIA associated with uveitis: in 60%, 100%, 79% respectively. ID frequency depended on the initial active joints count: if it was <5 ID was achieved in 93%; 5-15 – in 64%, >15 - 48%. The achievement of ID status during first year of treatment was observed more often in pts with disease duration less than 5 yrs. Results were comparable at the second year of treatment (52% vs 37% in the first year; 12% vs 37% in the second year). 61(59%) patients continue ABA therapy, in 43 cases ABA was cancelled (in 4 cases due to infusion/allergy reactions, in 27 cases due to the inefficacy, in 11 cases due to some non-medical reasons). 32% patient (33 cases) failed to comply with the infusion schedule due to organization problems in their regional clinic. The correct dose regimen and interval between infusions during the whole course of treatment influenced higher respond, thus ACRpedi70-90% improvement and ID after 12 months were achieved predominantly in patients who had no deviations in infusion regimen. The average survival rate of ABA was higher in biologics-naive (62% vs 56%) and in Poly-RFneg (66%) in comparison to Poly-RFpos (60%), Olygo (43%) and soJIA (33%). As it has appeared, efficiency of ABA significantly increased after 12 months of therapy. Uveitis remission was identified in 57% of cases after 6 months, in 75% of cases – after 12 months, in 62% of cases – after 18 months, in 60% of cases – after 24 four months. 7 pts had adverse events (3 pts had infusion reactions, 2 pts (the both with early ANA-positive oligoarthicular onset) developed uveitis de-novo, 1 patient had verruca vulgaris) but no serious events were observed.

**Conclusion:** Long-term therapy with ABA is supposed to be highly efficient for JIA, including patients with uveitis despite of frequent expert opinion, that it is less efficient than other Biologics. It was observed, that positive effect under ABA has continued to increase even after 24 month period of therapy. The efficiency of ABA depends on the correct infusion regimen and it is higher in biologics-naïve patients. Drug survival rate of ABA seemed to be better in biologics-naive pts, especially in Poly-RFneg subtype and the worst in soJIA. ABA showed a good safety profile in real clinical practice.

**Disclosure of Interest**

None Declared

### P229 Canakinumab is safe in the treatment of renal transplant patient having amyloidosis secondary to hyper- Ig-D syndrome

#### Ismail Dursun Dursun, Betul Sozeri, Aysenur Pac Kısaarslan, Ruhan Dusunsel, Hakan Poyrazoglu, Hakan Poyrazoglu

##### Pediatric Rheumatology, ERCIYES UNIVERSITESI TIP FAKULTESI HASTANESI, Kayseri, Turkey

###### **Presenting author:** Ruhan Dusunsel

**Introduction:** Canakinumab, an anti-Il-1b human monoclonal antibody, is very effective drug in reducing the frequency of episodes and improving clinical symptoms of patients with Hyper- Ig-D syndrome (HIDS). However, it’s effect on the renal transplant is unknown.

**Objectives:** Herein, we report a child with HIDS and renal transplant to share the efficacy and safety of canakinumab.

**Methods:** case report

**Results:** A ten year-old boy who followed HIDS since the age of 3, received kidney transplant from a living donor (mother) 2,5 years ago. He was on etanercept before transplant. It was discontinued after transplant due to ineffectiveness. Firstly, Anakinra was started 6 months after renal tx for HIDS attacks. After a short-term remission with anakinra HIDS attacks restarted. He experienced with acute disseminated encephalomyelitis that required plasmapheresis, IVIG and pulse methyl prednisolone. Since, he had severe diarrhea and weight loss due to gastrointestinal amyloidosis, anakinra was switched to canakinumab.

Canakinumab markedly reduced the frequency of diarrhea, increased appetite and rapidly normalized the acute phase reactants include CRP and SAA. After 6 months, he has on treatment canakinumab on 6 months. There was not reveal any adverse effects and graft dysfunction on canakinumab.

**Conclusion:** Canakinumab is a safe biologic agent to maintain disease control in children with HIDS even in renal transplantation course. We think that canakinumab can be successfully and safety used in renal transplant patients.

**Disclosure of Interest**

None Declared.

### P230 Biological therapy in chronic non-bacterial osteitis: experience of two tertiary centers

#### Sara Murias^1^, Estefania Barral^2^, Rosa Alcobendas^1^, Eugenia Enriquez^2^, Agustin Remesal^1^, Jaime de Inocencio^2^

##### ^1^Pediatric Rheumatology, University Hospital La Paz, Madrid, Spain; ^2^Pediatric Rheumatology, University Hospital 12 de Octubre, Madrid, Spain

###### **Presenting author:** Sara Murias

**Introduction:** Chronic non-bacterial osteitis (CNO) is a rare disease of unkwown etiology, recently considered an autoinflammatory disease. There is considerable heterogenity among centers regarding its management.

**Objectives:** To describe the clinical and therapeutical characteristics of 12 patients with CNO refractory to conventional therapy (NSAIDs and bisphosphonates) followed at 2 tertiary centres who received biological therapy.

**Methods:** Clinical chart review.

**Results:** 11 patients were included (9 females). Their mean age at onset was 8.7 years and their mean diagnosis delay was 12 months. All of them showed bone pain and 4 presented fever. Involved bone locations are described in the attached table, being femur (involved in 7/11), clavicle (4/11), vertebrae (5/11), tibia (4/11) and pelvis (4/11) the most frequently affected bones. C-Reactive Protein (CRP) was elevated in 10 patients (mean 18.8 mg*/L*) and ESR in 8 (mean 50 mm/h). IgD was determined in 4 cases, being increased in 2. *Propionibacterium acnes* was isolated in bone biopsy from 1 patient. Six children showed skin lesions, including psoriasis in 4. All patients received NSAIDs, 8 Pamidronate and all eventually received biological therapy with anti-TNF agents. At the last follow-up visit, 8 patients were still on biologicals (6, infliximab, 2 adalimumab) and 10 out of 11 remained asymptomatic.

**Conclusion:** CNO diagnosis is often delayed due to lack of suspicion of the disease. This series suggests that biological therapy may represent an effective alternative in refractory cases. Further research into the disease is needed to ascertain the precise role of biological therapy in its management.

**Disclosure of Interest**

None Declared

### P231 Vitamin D deficiency and musculoskeletal pain in children and adolescents: a causal relationship?

#### Tania M. Castro, Simone A. Lotufo

##### Pediatrics, HOSPITAL MUNICIPAL INFANTIL MENINO JESUS, São Paulo, Brazil

###### **Presenting author:** Tania M. Castro

**Introduction:** The most common chronic pain conditions in pediatric rheumatology settings include limb pain and fibromyalgia. Vitamin D is associated in decreasing the risk of many chronic illnesses, including autoimmune diseases, infectious diseases, cardiovascular disease, and bone pain. Although vitamin D deficiency has been linked to musculoskeletal pain in children and adolescents, their causal relationship is not yet well established.

**Objectives:** The aim of this prospective study was to determine if a causal relationship exists between musculoskeletal pain in children and adolescents and serum vitamin D levels, and correlates these levels with markers of bone metabolism (calcium [Ca], phosphate [P], alkaline phosphatase [ALP] and parathormone [PTH]).

**Methods:** We studied 80 patients aged 2.5-17.7 presenting in Pediatric Rheumatology Outpatient Clinic of Hospital Municipal Infantil Menino Jesus, Brazil. All patients fulfilled the diagnostic criteria of limb pain or fibromyalgia. Children and adolescents were investigated for Ca, P, ALP, PTH and vitamin D levels. Associations between vitamin D and the other laboratory variables were verified using Fisher's exact test due to sample size.

**Results:** The mean age of the participants was 10.2 years with the majority (57.5%) being females. A low level of vitamin D was presented in 88.7% patients with limb pain. ALP was elevated in 62 patients (79.5%) of which 53 (85.4%) had vitamin D deficiency (<20 ng/mL) / insufficiency (21-29 ng/mL) not statistically significant. No patient with normal levels of vitamin D had altered ALP. The patient who presented the lowest level of vitamin D (6.7 ng/mL) had the highest level of ALP (1147 U/L). Hypocalcemia was not identified in any patients. Hypophosphatemia was observed in one patient who had vitamin D insufficiency. Parathormone levels were normal in 88.4% of the patients with vitamin D deficiency/insufficiency.

**Conclusion:** We found a high frequency of hypovitaminosis D in children and adolescents with musculoskeletal pain. Raised levels of ALP reflected increased bone turnover in patients with deficient/insufficient levels of vitamin D. This suggests that ALP could be an early marker in patients with hypovitaminosis D.

**Disclosure of Interest**

None Declared.

### P232 Vaccine coverage in pediatric patients with rheumatic diseases with and without immunosuppression in Switzerland

#### Tatjana Freye^1^, Raffaella Carlomagno^2^, Thomas Zumbrunn^3^, Jan Bonhoeffer^4^, Elvira Cannizzaro Schneider^5^, Daniela Kaiser^6^, Michaël Hofer^2^, Véronique Hentgen^7^, Andreas Woerner^1^ and Juvenile Inflammatory Rheumatism (JIR) Cohort

##### ^1^Pediatric Rheumatology, University of Basel Children’s Hospital, Basel, Switzerland; ^2^Unité romande de rhumatologie pédiatrique, CHUV (Lausanne), University of Lausanne and HUG (Geneva), Lausanne and Geneva, Switzerland; ^3^Clinical Trial Unit, University of Basel Hospital, Basel, Switzerland; ^4^Pediatrics Infectious Diseases and Vaccines, University of Basel Children’s Hospital, Basel, Switzerland; ^5^Pediatric Rheumatology, Children’s Hospital Zurich, Zurich, Switzerland; ^6^Pediatric Rheumatology, Children’s Hospital Lucerne, Lucerne, Switzerland; ^7^Centre de référence des maladies autoinflammatoires CeRéMAI, Versailles Hospital, Versailles, France

###### **Presenting author:** Tatjana Freye

**Introduction:** Pediatric patients with rheumatic diseases (PedRD) are more susceptible to invasive infectious diseases, due to their underlying disease and immunosuppressive treatment. Vaccination reduces the risk of infection and its associated morbidity and mortality. Coverage rates in PedRD patients are scarce.

**Objectives:** To assess the vaccine coverage of PedRD patients in Switzerland.

**Methods:** Multicenter retrospective prevalence study based on the *Juvenile Inflammatory Rheumatism (JIR)* cohort, an international registry for pediatric rheumatic diseases. PedRD patients treated in the Swiss pediatric rheumatology centers in Basel, Geneva, Lausanne, Lucerne and Zurich were included in the study. Vaccine coverage was assessed at the date of diagnosis and as point prevalence on 12 April 2016 for routine vaccinations and specifically recommended ones for PedRD patients according to the Swiss Federal Office of Public Health (FOPH). Routine vaccinations include age-related immunization against diphtheria (D), tetanus (T), pertussis (Pa), polio (IPV), haemophilus influenzae type b (HIB), hepatitis B (HBV), human papillomavirus (HPV), varicella-zoster virus (VZV), measles, mumps and rubella (MMR). Specific vaccine recommendations for PedRD contain inoculation against pneumococci (PCV), seasonal influenza and HPV, HBV plus VZV as soon as possible after the diagnosis is made.

**Results:** 620 patients were included in the study. Of those, no data on vaccinations were available in 270 patients (44%). In 102 patients (17%) no recent vaccination card was available. 242 patients with updated vaccination card and informed consent were further analyzed. Their median age was 6.6 years (IQR 3.0-10.4) at diagnosis and 11.0 years (IQR 7.0-14.3) at the day of analysis; 152 (63%) were female.

At the time of diagnosis, 186 patients (77%) were fully immunized according to the routine schedule. Point prevalence on 12 April 2016 was 5.4% lower. In the 96 patients (40%) with immunosuppressive medication (conventional disease-modifying antirheumatic drugs [n = 34], biological disease-modifying antirheumatic drugs [n = 85], systemic steroids [n = 24]), 60 (62.5%) were fully immunized with routine vaccines. Only 31(13%) PedRD patients were fully vaccinated according to routine and specific vaccine recommendations.

Influenza vaccination was documented in 42 (17%) PedRD patients (ten with immunosuppressive therapy). Pneumococcal vaccination was listed in 134 (55%) PedRD patients (43 patients with immunosuppressive medication). HBV vaccination was completed in 111 (46%) patients. Seven (44%) of 16 female PedRD patients above 16 years received a complete series of HPV vaccine. History of VZV disease was assessed in 138 (57%) patients with a negative history in 36 (26%) patients. Of these, 15 (42%) patients (seven with immunosuppressive treatment) were not vaccinated against VZV.

In comparison to the age specific vaccine coverage of the general population in Switzerland in 2014, PedRD vaccination rates were 3.5% lower and 12.5% lower in PedRD patients with immunosuppressive therapy.

**Conclusion:** Documentation of administered vaccines in the medical records of pediatric rheumatology clinics in Switzerland is limited. Age-specific vaccine coverage rates appear lower in PedRD patients as compared to the general population. Close collaboration between specialists and primary care providers and repetitive evaluation of vaccine coverage could optimize the protection of this vulnerable population from vaccine preventable diseases.

**Disclosure of Interest**

T. Freye: None Declared, R. Carlomagno: None Declared, T. Zumbrunn: None Declared, J. Bonhoeffer: None Declared, E. Cannizzaro Schneider: None Declared, D. Kaiser: None Declared, M. Hofer Grant / Research Support from: Abbvie, Novartis, Consultant for: Novartis, V. Hentgen: None Declared, A. Woerner: None Declared.

### P233 Use of glucocorticoids in first-line treatment of juvenile idiopathic rheumatoid factor negative polyarthritis: data from a German inception cohort

#### Tobias Schwarz^1^, Jens Klotsche^2,3^, Martina Niewerth^2^, Gerd Horneff^4^, Johannes-Peter Haas^5^, Anton Hospach^6^, Hans-Iko Huppertz^7^, Gerd Ganser^1^, Kirsten Minden^2,8^ and ICON study group

##### ^1^Department of Pediatric Rheumatology, St. Josef-Stift, Sendenhorst, Germany; ^2^German Rheumatism Research Center, Berlin, Germany; ^3^Institute for Social Medicine, Epidemiology and Health Economics, Charité Universitätsmedizin Berlin, Berlin, Germany; ^4^Department of Pediatrics, Asklepios Klinik Sankt Augustin, Sankt Augustin, Germany; ^5^German Centre for Rheumatology in Children and Adolescents, Garmisch-Partenkirchen, Germany; ^6^Department of Pediatrics, Klinikum Stuttgart, Stuttgart, Germany; ^7^Prof. Hess-Kinderklinik, Klinikum Bremen-Mitte, Bremen, Germany; ^8^Children's University Hospital, Charité Universitätsmedizin Berlin, Berlin, Germany

###### **Presenting author:** Tobias Schwarz

**Introduction:** Treatment of polyarticular juvenile idiopathic arthritis (JIA) requires disease modifying antirheumatic drug (DMARD) therapy as a rule, in most cases initially with methotrexate (MTX). The additional benefit of systemically or locally administered glucocorticoids (GC), however, is controversially discussed.

**Objectives:** To investigate the impact of intra-articular or systemically administered GC, in addition to DMARD therapy with MTX, in the first-line treatment of rheumatoid factor negative polyarticular JIA (RF- PA).

**Methods:** Patients with JIA were enrolled in the German inception cohort ICON at 11 large German pediatric rheumatology units. Laboratory and clinical parameters such as JIA core set criteria, data on the medication used, as well as the Pediatric Quality of Life Inventory (PedsQL) were collected at defined time points. The following three treatment strategies of the first three months of observation were compared: group 1) MTX plus high-dose systemic GC (>0,2 mg/kg body weight) without intra-articular GC in >4 joints, group 2) MTX plus intra-articular GC in >4 joints without high-dose systemic GC, and group 3) MTX without high-dose systemic GC or intra-articular GC in >4 joints. A propensity score was estimated by logistic regression analysis to model potential differences in clinical characteristics at treatment start between the three groups. Weights were calculated as ratios of the estimated probabilities and balanced samples of patients were obtained by using an inverse probability weight in the statistical analyses. Outcome variables were considered at the one-year and two-year follow-up.

**Results:** Of the 954 patients with JIA enrolled in the inception cohort, 181 patients were diagnosed with RF- PA and were almost DMARD naive at study inclusion. Patients had a mean age of 6.7 years and a mean disease duration of 1.5 months at enrolment. The mean clinical Juvenile Arthritis Disease Activity Score (cJADAS-10) was 14.8, the mean C-HAQ 0.96 and the mean PedsQL 61.9 at study inclusion. Almost 90% of the patients were treated according to one of the defined strategies: group 1 n = 41, group 2 n = 57, group 3 n = 61.

At one-year and two-years of follow-up there were no significant differences between the three groups with regard to the physicians global assessment of disease activity, the cJADAS-10, and the proportion of patients with inactive disease. Altogether, 49% and 54% of patients had attained inactive disease at the 1-year and 2-year follow-up, respectively. At one-year of follow-up, patients of group 3 were treated to a higher proportion with biological DMARDs (49.7%) than patients of group 2 (20.4%). At two-years of follow-up, however, the difference was not statistically significant any more.

Patients additionally treated with high-dose or multi-local intra-articular GC reported a similar functional status as patients of group 3, patients of group 2 however had a slightly higher health-related quality of life than patients of the other two groups. Regarding growth and body mass index, there was no difference between the patients treated with high-dose systemic GC and the other two groups at one-year and two-years of follow-up.

**Conclusion:** The three treatment strategies were similarly successful in reaching an inactive disease. Patients treated with multi-local intra-articular GC tended to have a better quality of life at follow-up. This should be confirmed in further studies.

**Disclosure of Interest**

None Declared.

## Poster Session: Autoinflammatory diseases II

### P234 The safety and efficacy of live-attenuated vaccines in patients using IL-1 or IL-6 blockade

#### Jerold Jeyaratnam^1^, Nienke ter Haar^1^, Ozgur Kasapcopur^2^, Donato Rigante^3^, Fatma Dedeoglu^4^, Ezgi Baris^4^, Sebastiaan Vastert^1^, Nico Wulffraat^1^, Joost Frenkel^1^

##### ^1^University Medical Center Utrecht, Utrecht, Netherlands; ^2^Cerrahpasa Medical School-Istanbul University, Istanbul, Turkey; ^3^Università Cattolica Sacro Cuore, Rome, Italy; ^4^Boston Children’s Hospital, Boston, USA

###### **Presenting author:** Jerold Jeyaratnam

**Introduction:** Vaccination has contributed greatly to child health and has been considered generally safe. However, existing guidelines recommend withholding patients using biologicals from receiving live-attenuated vaccines, as there is no safety data available.

**Objectives:** To investigate the safety and efficacy of live-attenuated vaccines in patients using interleukin (IL)-1 or IL-6 blocking agents.

**Methods:** A large number of international (paediatric) rheumatologists were asked to send in cases of patients who received a live-attenuated vaccine while using IL-1 blockade (anakinra, canakinumab or rilonacept) or IL-6 blockade (tocilizumab).

**Results:** Nine patients have been included in this study so far (6 female, 3 male). The median age at vaccination was 9 years. Six patients suffered from systemic juvenile idiopathic arthritis (sJIA). The tree other patients suffered from Mevalonate Kinase Deficiency (MKD), Familial Mediterranean Fever (FMF) and Chronic Infantile Neurologic Cutaneous and Articular (CINCA) syndrome. Patients were vaccinated with Measles, Mumps and Rubella (MMR) booster, varicella zoster booster or first vaccine, or oral polio vaccine.

All characteristics are summarized in Table 17. In all but one patient (pt1, 3-9) the underlying disease was well controlled at the time of vaccination. In one patient (pt2) disease was only partially controlled.

Adverse events were noted in three patients, of which one (pt8) was a serious adverse event leading to hospitalization. Two patients suffered from infection after vaccination, of which one was caused by the micro-organism of the vaccine (pt8). Four patients experienced a flare of their disease. In three patients (pt1,2,6) the vaccination did not lead to a flare or adverse events. Anakinra was stopped two or three days before vaccination and restarted days to weeks later in these three patients.

**Conclusion:** In this small case series, 3/9 patients experienced an adverse event potentally caused by the vaccine strain. Four patients experienced a flare of their disease. There were no adverse events in patients whose anakinra treatment was interrupted around the vaccination, but there’s not yet sufficient data to promote such an approach. Before considering any prospective trial, more retrospective data are needed. We therefore invite centers to contribute cases to our study.

**Disclosure of Interest**

None Declared.

**Table 17 (abstract P234).** Characteristics of patients who received live-attenuated vaccines

Patient	Disease	Biological	Other medication	Vaccination	Adverse events	Flare
1	CINCA	Anakinra 1.5 mg/kg/day	Methyl-prednisolone	MMR booster	-	-
2	MKD	Anakinra 1.4 mg/kg/day	-	MMR booster	-	-
3	FMF	Canakinumab 4 mg/kg/monthly	Prednisone Colchicine	MMR booster	-	FMF attack after 1 week
4	sJIA	Canakinumab 4 mg/kg/2 months	Prednisone Methotrexate Ibuprofen (if necessary)	MMR booster	Pneumonia 1 week after vaccination	Fever and rash attacks
5	sJIA	Anakinra 1.7 mg/kg/2 days	Methotrexate Indometacine	MMR booster	-	A bit more exanthema due to stop of anakinra
6	sJIA	Anakinra 40 mg/3 days	-	MMR booster	-	-
7	sJIA	Tocilizumab 132 mg/14 days	Prednisone Indometacine	Varicella zoster (first vaccine)	-	Exanthema, subfebrile temperature, malaise
8	sJIA	Anakinra 4 mg/kg/day	Prednisone Methotrexate Leflunomide Thalidomide	Varicella zoster (booster)	Hospitalization due to varicella infection and immune-suppression, treatment with acyclovir	-
9	sJIA	Tocilizumab 12 mg/kg/monthly	Prednisone Ibuprofen (if necessary)	Oral polio vaccine	Diarrhea	-

### P235 Impact of familial Mediterranean fever, MKD/HIDS, and TRAPS on patients and families, data from patient interviews

#### Jonathan S. Hausmann^1,2^, Kathleen G. Lomax^3^, Ari Shapiro^4^, Karen L. Durrant^5^

##### ^1^Boston Children’s Hospital, Boston, USA; ^2^Beth Israel Deaconess Medical Center, Boston, USA; ^3^Novartis Pharmaceuticals Corporation, East Hanovar, USA; ^4^Flince Research, Brooklyn, USA; ^5^Autoinflammatory Alliance, San Fracisco, USA

###### **Presenting author:** Kathleen G. Lomax

**Introduction:** Patients with the rare autoinflammatory diseases of familial mediterranean fever (FMF), mevalonate kinase deficiency (MKD)/hyperimmunoglobulin D syndrome (HIDS), and tumour necrosis factor receptor-associated periodic syndrome (TRAPS) often have long diagnostic journeys. Patient and family experiences along this path to diagnosis are poorly understood.

**Objectives:** To understand the experiences of patients and families with autoinflammatory diseases through diagnosis and treatment.

**Methods:** We employed 90-minute semi-structured qualitative interviews and 5-day written/video diaries of patients and families with autoinflammatory diseases

**Results:** Twelve US families participated in the fall of 2015 including 4 TRAPS patients, 5 MKD/HIDS patients, and 5 FMF patients. The patients’ ages ranged from 1-28 years. Caregivers reported an insidious onset of symptoms, but were initially reassured by their healthcare providers (HCPs) that symptoms were related to normal childhood illnesses. Most parents in this study (86%) realised that something was seriously wrong only after medical crises and hospitalisations. The diagnostic path began in earnest then, including many specialist visits (often with long waits for appointments), extensive testing, and many misdiagnoses including Lyme disease, meningitis, H1N1 influenza, systemic lupus erythematosus, systemic juvenile idiopathic arthritis, atypical Kawasaki’s disease, leukaemia, lymphoma, bone cancer, and Crohn’s disease. Most parents (92%) lost faith in the medical system’s ability to find an answer to their children’s symptoms, while they also struggled with unsupportive school officials and dismissive friends and relatives. Parents and patients frequently felt a loss of self-confidence and increasing alienation in the face of criticism and disbelief. Patients and caregivers report holding onto a memory of what normal life was like prior to the onset of symptoms, and mourning their subsequent loss of normalcy. Receiving the diagnosis of an autoinflammatory disease provided vindication and relief, as well as a focus for further education and treatment. Even after diagnosis, patients and caregivers reported continuing confusion about what to expect in the future. Many (64%) reported disease symptoms between flares that were rarely recognised as such by their HCPs. Uncertainties about their diseases led patients and caregivers to seek online support communities, but struggled finding patients whose symptoms mirrored their own.

**Conclusion:** Patients with autoinflammatory diseases often encounter long diagnostic delays, causing significant stress and confusion for the patient and their families. Distrust of the medical establishment may persist even after diagnosis. Loss of normalcy is a core tragedy for many families. Confusion and uncertainty continue to mark these families’ lives, even after diagnosis. Initiatives that improve the speed and accuracy of diagnosis, provide more comprehensive patient education, and support patients and their families through their illness have the potential to greatly improve the lives of patients with autoinflammatory diseases.

**Disclosure of Interest**

J. Hausmann Consultant for: Novartis, K. Lomax Employee of: Novartis, A. Shapiro Consultant for: Novartis, K. Durrant: None Declared.

### P236 Efficacy and safety of canakinumab in patients aged one to six years with cryopyrin-associated periodic syndromes: results of an open-label, phase III extension study

#### P. A. Brogan^1^, M. Hofer^2^, J. B. Kuemmerle-Deschner^3^, B. Lauwerys^4^, A. Speziale^5^, K. Leon^6^, X. Wei^7^, R. M. Laxer^8^

##### ^1^UCL Institute of Child Health, and Great Ormond Street Hospita, London, UK; ^2^Unité Romande de Rhumatologie Pédiatrique, Hospitalier Universitaire Vaudois, Lausanne, Switzerland; ^3^University Hospital Tuebingen, Tuebingen, Germany; ^4^Cliniques Universitaires Saint Luc and Université Catholique de Louvain, Brussels, Belgium; ^5^Novartis Pharma AG, Basel, Switzerland; ^6^Novartis Pharmaceuticals Corporation, New Jersey, USA; ^7^Novartis Pharma, Beijing, China; ^8^University of Toronto, The Hospital for Sick Children, Toronto, Canada

###### **Presenting author:** P. A. Brogan

**Introduction:** Cryopyrin-Associated Periodic Syndrome (CAPS), is a rare hereditary auto inflammatory disorder representing 3 phenotypes: familial cold auto-inflammatory syndrome (FCAS), Muckle-Wells syndrome (MWS), and chronic infantile neurologic cutaneous and articular syndrome/neonatal onset multisystem inflammatory disease (CINCA/NOMID)^1^. Canakinumab (CAN), a fully human anti–IL–1β monoclonal antibody has been shown to be effective over 56 weeks in patients with CAPS aged <24 months (core study)^2^. Here we report the results of an extension study (up to 152 weeks) to this core study (NCT01302860).

**Objectives:** To assess the long-term efficacy (in terms of relapse) and safety of CAN in CAPS pts who completed the core study.

**Methods:** CAPS pts who completed the core study received 2 mg/kg subcutaneous CAN every 8 weeks, in continuation with the core study. Pts who received a dose adjustment in the core study were continued on the same dose in the extension phase. Efficacy was evaluated by investigator clinical assessment of autoinflammatory disease activity, C-reactive protein (CRP) and serum amyloid A (SAA) levels. Safety was assessed in terms of adverse events (AEs).

**Results:** Of 17 pts (aged ≤6 years), enrolled in the extension study, 12 (70.6%), 4 (23.5%) and 1 (5.9%) had MWS, NOMID and FCAS phenotypes, respectively. All 17 pts were complete responders; 16 (94.1%) had no flare, and 1 (5.9%) NOMID patient had a flare at the end of extension study. Physician global assessment improved over the extension study, with decline in severity from baseline of the core study to the end of extension study (EOS). The number of pts with absent autoinflammatory disease activity improved from 4 (23.5%) to 11 (64.7%); minimal activity increased from 5.9% (1 pt) to 29.4% (5 pts); mild or moderate activity decreased from 47.1% (8 pts) to 5.9% (1 pt); moderate activity decreased from 23.5% (4 pts) to 0 patients. This improvement was also observed in the assessment of skin rash; proportion of patients with no skin disease increased from 29.4% (5 pts) at baseline of core study to 94.1% (16 pts) at EOS. The mean decrease in CRP and SAA levels from core study baseline was −10.4 and −54.36 mg/L, respectively at EOS. Overall, 10 (58.8%) pts had AEs suspected to be related to CAN; the most common were diarrhoea, pneumonia, rhinitis and cough (3 pts each). Eight (47.1%) pts experienced at least 1 serious AE (4 MWS and 4 NOMID pts), with pneumonia being the most common (2 [11.8%]). No deaths occurred during the study.

**Conclusion:** Canakinumab effectively maintained clinical and serological efficacy in CAPS pts in the extension study. No new safety findings were observed, and the safety profile of canakinumab was consistent with previous studies, which corroborates the long-term use of canakinumab in the treatment of CAPS patients.

**References**

1. Kuemmerle-Deschner JB, et al. *Arthritis Res Ther*. 2011;13(1):R34.

2. Brogan, P et al. *Arthritis Rheumatol.*2015;67 (suppl 10).

**Trial registration identifying number:** NCT01576367

**Disclosure of Interest**

P. Brogan Grant / Research Support from: Novartis, Roche and SOBI, Consultant for: Novartis Roche and SOBI, M. Hofer Consultant for: Novartis, J. Kuemmerle-Deschner Grant / Research Support from: Novartis, Consultant for: Novartis, SOBI, Baxalta, B. Lauwerys: None Declared, A. Speziale Employee of: Novartis, K. Leon Employee of: Novartis, X. Wei Employee of: Novartis, R. Laxer Grant / Research Support from: Novartis for Database funding.

### P237 Clinical response to ustekinumab in severe erytrodermic psoriasis caused by CARD-14 mutation (CARD-14 mediated psoriasis; CAMPS)

#### Sara Signa^1^, Marta Rusmini^2^, Elena Campione^3^, Sabrina Chiesa^1^, Alice Grossi^2^, Alessia Omenetti^1^, Roberta Caorsi^1^, Gianmaria Viglizzo^4^, Alberto Martini^1^, Isabella Ceccherini^2^, Marco Gattorno^1^

##### ^1^U.O. Pediatria II, Genova, Italy; ^2^U.O. Genetica Medica, IIRCCS G. Gaslini, Genova, Italy; ^3^Clinica Dermatologica, Policlinico Universitario di Roma “Tor - Vergata”, Roma, Italy; ^4^UO Dermatologia, IIRCCS G. Gaslini, Genova, Italy

###### **Presenting author:** Sara Signa

**Introduction:** Autosomal dominant gain of function mutations in caspase recruitment domain family member 14 (CARD14) were found to cause plaque psoriasis in two families and severe generalized pustular psoriasis as a monogenic form of childhood (CARD14-mediated psoriasis, CAMPS). CARD14 mutations have also been implicated in pityriasis rubra pilaris. The therapeutic approach in CAMPS includes drugs used for the treatment of moderate-to-severe psoriasis, such as, methotrexate, cyclosporine and biological agents, such as anti-TNF antibodies. Ustekinumab is a monoclonal antibody that notably targets the p40 subunit of both IL-12 and IL-23, which are understood to play a role in activation of NFkB pathway.

**Objectives:** i) to describe the case of a family presenting with an unusual form of severe erytrodermic psoriasis without pustulosis in which whole exome sequencing (WES) analysis revealed a novel CARD-14 mutation ii) to report the clinical and immunological response to ustekinumab.

**Methods:** The probands are two 7 year-old twins suffering from non-specific dermatitis since the age of 9 months with the appearence of localized erythroderma with a gradual diffusion over all the skin surface by the second year of life. These cutaneous lesions are severely itchy and associated with onychodystrophies and fissures in both children, and ectropion in the twin sister. There are three pairs of twins in the family, five of them presenting psoriasis and two of them presenting psoriatic arthritis. The children presented poor clinical response to topic and systemic therapy with antihistamine, steroid, retinoid, cyclosporine and etanercept. Furthermore we have evaluated IFNgamma, IL-17A and IL-22 producing CD4+ T cells following peripheral blood mononuclear cells (PBMCs) stimulation with PMA/ionomycin by intracellular cytokine assay.

**Results:** WES of the family revealed in the affected members a novel missense mutation of CARD-14 gene (c.446 T > G, leading to the missense aminoacid substitution p.L149R). We treated the children with ustekinumab at dosage of 0.75 mg/kg at week 0 and 4 and every 12 weeks. At week 4 and week 16 both twins showed a dramatic improvement of their clinical conditions, with no significant collateral effect. IL-17A and IL-22 levels of stimulated PBMCs at week 16 were decreased after ustekinumab treatment, consistent with the clinical findings

**Conclusion:** CARD14 gain of function mutations can give rise to unusual clinical phenotype like diffuse erytrodermic psoriasis and can be associated to arthritis. Ustekinumab could be a powerful therapeutic option for this unusual and refractory form of disease, also in pediatric age.

**Disclosure of Interest**

None Declared.

### P238 The longitudinal Eurofever project: an update on enrollment

#### Silvia Federici^1^, Joost Frenkel^2^, Seza Ozen^3^, Helen Lachmann^4^, Martina Finetti^1^, Alberto Martini^1^, Nicola Ruperto^1^, Marco Gattorno^1^ and on behalf of PRINTO and Eurofever Registry

##### ^1^Pediatria 2, Istituto Giannina Gaslini, Genova, Italy; ^2^Department of Paediatrics, University Medical Center Utrecht, Utrecht, Netherlands; ^3^Department of Pediatric Nephrology and Rheumatology, Hacettepe University, Ankara, Turkey; ^4^National Amyloidosis Centre, Royal Free Campus, University College Medical School, London, UK

###### **Presenting author:** Silvia Federici

**Introduction:** In 2008 the Paediatric Rheumathology European Society (PReS) promoted an International Project (EAHC, Project No2007332) for the study of Autoinflammatory Diseases (AIDs) named Eurofever whose main purpose is to create a web-based registry for the collection of clinical, laboratory and response to treatment information in patients with AIDs.

**Objectives:** To implement the Registry with the new recently described AIDs and genes and modify the web-system to make it suitable for the collection of longitudinal data and for a better collection of data regarding treatment.

**Methods:** With the technical help of Paediatric Rheumatology INternational Trial Organization (PRINTO) web-masters, we were able to revise the electronic case report forms bringing 2 main novelties: i) inclusion of the more recently described AIDs (DADA2, CAMPS, SAVI) and the relative clinical manifestation ii) modification of drug layout for a better collection of data regarding treatment. In this process information on safety and efficacy have been included. Moreover, starting from February 2015 we started the longitudinal collection of follow-up data for the patients already included in the Registry with particular focus on modification of the clinical picture, onset of complication/sequelae, treatment, adverse event if present, laboratory and instrumental findings.

**Results:** Up to date 3541 patients have been enrolled in the Registry from 108 centers in 39 countries. At present baseline demographic information from 3541 (M:F = 1749:1792) patients are available. In 75% complete clinical information from disease onset to diagnosis and response to treatment is also available. The following patients has been enrolled: FMF 955 pts (738 with complete clinical data); TRAPS 289 pts (229 with complete clinical data); CAPS 312 pts (216 with complete clinical data); MKD 200 pts (167 with complete clinical data); Blau’s disease 72 pts (22 with complete clinical data); PAPA 33 pts (25 with complete clinical data); NLRP-12 mediated periodic fever 13 pts (9 with complete clinical data); DIRA 4 pts (3 with complete clinical data); CANDLE 1 pts (with complete clinical data), DITRA 1 pt and Majeed 3 pts (2 with complete clinical data), DADA2 2 pts (complete), SAVI 1 pt. Among multifactorial autoinflammatory diseases: PFAPA 668 pts (409 with complete clinical data); CRMO 499 pts (461 with complete clinical data); pediatric Bechet disease 206 pts (163 with complete clinical data) and 280 patients with undefined periodic fever (217 with complete clinical data). Detailed longitudinal data on treatment efficacy and safety are available for 205 patients.

**Conclusion:** A common registry for collection of patients with Autoinflammatory disease is available and the enrolment is on going. This project represents the first attempt of a common registry for different Autoinflammatory Syndromes. The longitudinal collection and analysis of data coming from the Registry will improve our knowledge in the field of Autoinflammation both on the natural history of the single disease and the efficacy and safety of the treatment commonly used in the clinical practice with particular regards to biologics.

**Disclosure of Interest**

None Declared.

### P239 Results of the Eurofever Delphi survey for the classification criteria for monogenic periodic fever syndromes

#### Silvia Federici^1^, Federica Vanoni^2^, Seza Ozen^3^, Michaël Hofer^4^, Joost Frenkel^5^, Helen Lachmann^6^, Alberto Martini^1^, Nicola Ruperto^1^, Marco Gattorno^1^ and on behalf of PRINTO and Eurofever Project

##### ^1^Pediatria 2, Istituto Giannina Gaslini, GENOVA, Italy; ^2^Departement of Pediatric of Southern Switzerland, ORBV, Bellinzona, Switzerland; ^3^Department of Pediatric Nephrology and Rheumatology, Hacettepe University, Ankara, Turkey; ^4^Pediatric Rheumatology Unit CHUV, University of Lausanne, Lausanne, Switzerland; ^5^Department of Paediatrics, University Medical Center Utrecht, Utrecht, Netherlands; ^6^National Amyloidosis Centre, Royal Free Campus, University College Medical School, London, UK

###### **Presenting author:** Silvia Federici

**Introduction:** Provisional evidence-based classification criteria for inherited periodic fever (TRAPS, FMF, MKD and CAPS) have been recently developed based on the Eurofever registry. However, no consensus on how to combine clinical criteria, laboratory test and results of molecular analysis has been reached so far.

**Objectives:** To understand which variables physicians involved in the clinical care of patients with Autoinflammatory diseases (AIDs) consider as the most important for the classification of patients with inherited periodic fever.

**Methods:** Two following Delphi surveys were sent to health professionals (Clinicians and Genetists) working in the field of autoinflammation. In the first open survey 124 experts involved in the Eurofever registry could list all the variables they consider useful for the diagnosis of each monogenic periodic fever. The variables may be of any type (i.e. clinical features, laboratory or instrumental tests, genetic tests) and each expert could complete the survey for one or more disease on the basis of his expertise. In the second survey 162 experts (including American experts) were asked to select, from a list of items coming from the first survey, the 10 top variables and to rank them by assigning a score from 10 to 1 in order of importance. There was no possibility to assign the same rank to multiple variables. This process has been conducted in parallel with an analogous process for PFAPA syndrome

**Results:** The overall rate of response to the first and second Delphi surveys, including PFAPA syndrome, was respectively of 86% (107/124 experts) and 87% (141/162 experts). The variables corresponding to the 3rd quartile considering the total score obtained by the variables after the second Delphi survey were respectively: 20 for MKD (with a total score ranging from 637 to 72), 21 for CAPS (with a total score ranging from 554 to 66), 17 for FMF (with a total score ranging from 626 to 80), and 20 for TRAPS (with a total score ranging from 637 to 82).

**Conclusion:** Our process led to the identification of those features that are considered to be the most important as candidate variable to be included in a new set of evidence based classification criteria for monogenic periodic fever. The next step of this project (now ongoing) is the evaluation by a panel of experts of 360 real patients affected by AIDs (PFAPA, monogenic periodic fever and undefined periodic fever) randomly selected from EUROFEVER Registry. Those patients reaching the 80% of consensus among experts in the evaluation process will be used for the subsequent statistical analysis aimed at identify the best evidence-based classification criteria (in term of sensitivity and specificity) for PFAPA and Monogenic periodic fevers.

**Disclosure of Interest**

None Declared.

### P240 Recommendations for genetic testing of NLRP3 mutation-negative CAPS patients through comparison of sequencing methods

#### Sonia Melo Gomes^1,2^, Ebun Omoyinmi^1^, Juan I. Arostegui^3^, Eva Gonzalez-Roca^3^, Despina Eleftheriou^1, 2^, Nigel Klein^1, 4^, Paul Brogan^1, 2^

##### ^1^Rheumatology, Institute of Child Health, London, UK; ^2^Rheumatology, Great Ormond Street Hospital, London, UK; ^3^Immunology, Hospital Clinic, Barcelona, Spain; ^4^Infectious Diseases, Great Ormond Street Hospital, London, UK

###### **Presenting author:** Sonia Melo Gomes

**Introduction:** A high percentage of CAPS patients are negative for mutations in NLRP3 exons 3, 4, and 6. Somatic mosaicism has been shown to account for up to 70% of these patients who are ‘mutation-negative’ by conventional sequencing methods. In addition, there is significant clinical overlap between known autoinflammatory syndromes, and true genetic heterogeneity is also a possibility.

**Objectives:** The aim of this study was therefore to establish a stepwise approach for further genetic screening of mutation-negative CAPS patients, through comparison of different sequencing methods.

**Methods:** Four different sequencing methods were used for comparison: 1. Sanger sequencing of NLRP3 exons 3, 4, and 6; 2. Massively parallel sequencing (MPS) of NLRP3 exons 3, 4, and 6 (the current gold standard for detection of somatic mosaicism); 3. Our group’s targeted gene panel for vasculitis and inflammation (Vasculitis and Inflammation Panel, VIP), which sequences a total of 166 genes including most of the known genes linked to monogenic autoinflammatory diseases (AID); and 4. Whole Exome Sequencing (WES).

**Results:** Four patients with a clinical diagnosis of CAPS who were NLRP3 “mutation negative” on initial screening were chosen for comparison; the clinical phenotypes were compatible with Muckle Wells syndrome (n = 2); and CINCA (n = 2). Four different possible scenarios were thus available for comparison as Patient 1 had a low level somatic NLRP3 mutation, Patient 2 had a moderate-high level somatic NLRP3 mutation, Patient 3 had a mutation in another exon of the NLRP3 gene, and Patient 4 had a mutation in another known autoinflammatory gene (NOD2), therefore changing the diagnosis to Blau Syndrome. The results are presented in the table.

**Conclusion:** This study illustrates the diagnostic utility of 4 different genetic screening strategies for CAPS. Our VIP panel was the only technique capable of identifying the causative mutation in all cases. Sanger sequencing of NLRP3 exons 3, 4, and 6 will by definition fail to detect other important pathogenic mutations, as in the case of patient 3 with an exon 5 NLRP3 mutation. Sanger sequencing also lacked sensitivity for the detection of somatic mosaicism. While WES has the advantage of potentially identifying novel causative genes, it is not sensitive enough to detect mosaicism at an allelic frequency lower than 10%, as illustrated by patient 1, and is more expensive. Patient 4 illustrates the important phenotypic overlap for AID.

Other NLRP3 exons besides 3, 4, and 6 should be routinely screened for germline and somatic NLRP3 mutations before a CAPS patient can be truly designated as mutation negative. A targeted gene panel, such as the VIP panel, that can successfully identify even low level somatic mutations and covers most known autoinflammatory genes to date, is the most cost-effective approach. WES and eventually Whole Genome Sequencing could be reserved for the identification of novel genes, where no answers are revealed following a targeted panel screen. Therefore we currently recommend initial screening with a targeted next generation sequencing gene panel as a first tier test, followed by WES in case no germline or somatic mutation is identified in known autoinflammatory genes.

**Disclosure of Interest**

None Declared.

**Table 18 (abstract P240).** See text for description

Patient	Clinical CAPS phenotype	Gene	Exon	Mutation	Type	Sanger detection	MPS detection	VIP detection	WES detection
1- ♂, 8y	MWS	NLRP3	3	p.E567K	Mosaic 4%	No	Yes	Yes	No
2- ♂, 7y	MWS	NLRP3	3	p.F566L	Mosaic 14.5%	No	Yes	Yes	Yes
3- ♂, 2y	CINCA	NLRP3	5	p.G779V	Heterozygous	No	No	Yes	Yes
4-♀, 16y	CINCA	NOD2	4	p.D512Y	Heterozygous	No	No	Yes	Yes

### P241 Type I interferonopathies: diagnostic approach and preliminary results of treatment with a JAK1/2 inhibitor

#### Stefano Volpi^1,2^, Elettra Santori^1^, Paolo Picco^2^, Claudia Pastorino^2^, Roberta Caorsi^2^, Gillian Rice^3^, Alessandra Tesser^4^, Alberto Martini^2^, Yanick Crow^3,5^, Fabio Candotti^1^, Marco Gattorno^2^

##### ^1^Allergy and Immunology, Lausanne University Hospital, Lausanne, Switzerland; ^2^U.O. Pediatria 2, IST. G. GASLINI, Genova, Italy; ^3^Genetic Medicine, Manchester Academic Health Science Centre, University of Manchester, Manchester, UK; ^4^Università degli studi di Trieste, Trieste, Italy; ^5^Institute Imagine, University Paris Descartes, Paris, France

###### **Presenting author:** Stefano Volpi

**Introduction:** Defective regulation of type I interferon response is associated with severe inflammatory phenotypes and autoimmunity. Type I interferonopathies are a clinically heterogenic group of Mendelian diseases with a constitutive activation of this pathway that might present as atypical, severe, early onset rheumatic diseases. Skin vasculopathy with chilblains and livedo reticularis, interstitial lung disease, and panniculitis are common. STING associated vasculopathy with onset in infancy (SAVI) and Chronic Atypical Neutrophilic Dermatosis with Lipodystrophy and Elevated temperature (CANDLE) are examples of such. Currently, diagnosis and pharmacological management of these diseases is challenging.

**Objectives:** To test blood interferon signature as a screening tool for type I interferonopathies in children with early-onset SLE and vasculopathy and to test a JAK 1/2 inhibitor as a compassionate treatment.

**Methods:** We collected blood samples from a cohort of more than 40 pediatric rheumatologic patients and scored them according to a qPCR based IFN gene signature assay. An IFN score was calculated for each patient using the median fold change of gene expression related to a healthy control. Patients were selected based on the presence of the following features: i) atypical or incomplete SLE-like symptoms occurring in infancy or in preprepubertal age; ii) vasculopathy (skin ulcers, chilblains, strokes) iii) panniculitis with or without lypodystrophy iv) interstitial lung disease in the context of systemic inflammation. Molecular analysis (targeted or unbiased) was performed in patients with a positive score. A compassionate treatment of the patients with SAVI using Ruxolitinib, a JAK1/2 inhibitor was started.

**Results:** We identified 2 patients with heterozygous mutations in *TMEM173* and 1 patient with a homozygous mutation in *DNASE1L3*. Molecular screening in other patients with a positive signature is currently ongoing. In one patient with SAVI we observed a good response to Ruxolitinib that allowed steroid tapering and clinical control of both skin vasculopathy and lung interstitial disease. Follow up of the second patient with SAVI is on-going.

**Conclusion:** Combination of peripheral blood interferon signature and molecular analysis is an effective strategy for the identification of type I interferonopathies. Furthermore, we believe that targeting interferon receptor signaling represents a promising therapeutic option for these patients.

**Disclosure of Interest**

None Declared.

### P242 MEFV mutations and diagnostic delay in pediatric FMF according to patients’ age at onset

#### Kenan Barut, Sezgin Sahin, Amra Adrovic, Ada B. Sinoplu, Gozde Yucel, Gizem Pamuk, Ozgur Kasapcopur

##### Pediatric Rheumatology, Istanbul University, Cerrahpasa Medical School, Istanbul, Turkey

###### **Presenting author:** Kenan Barut

**Introduction:** Familial Mediterranean Fever is an autosomal recessive inflammatory disease characterized with recurrent polyserositis attacks. Severity of clinical manifestations depends on the MEFV mutation type. Approximately 87% of FMF patients carry a mutation on the exon 10 (M694V, M680I, V726A, M694I). The other common site of the mutation is exon 2 (E148Q). As being well-known, M694V mutation is associated with the most severe phenotype of the disease. Previous studies reported M694V to be related to younger age at disease onset.

**Objectives:** We aimed to assess the MEFV mutation type according to patients’ age in a single center large cohort of pediatric FMF patients. Additionally, we tried to evaluate the association between M694V homozygosity and patients’ age at disease onset.

**Methods:** This study included FMF patients followed up at Department of Pediatric Rheumatology, Cerrahpasa Medical Faculty in the time period of November 2014-March 2015. A total of 708 patients followed up at our department with diagnosis of FMF and under colchicum treatment for at least 6 months were included in the study. Diagnosis of FMF was established according to international pediatric FMF diagnostic criteria. Data on the age at disease onset were obtained from patients’ parents. Age of patients were divided in 4 different groups: ≤2, >2- ≤ 5, >5- ≤ 10 and >10- ≤ 18 years. MEFV mutations were divided in 5 different groups: M694V/M694V, M694V/N, M694V/Exon 10 mutations, Exon 10 /N, other exon mutations and no mutation. We evaluated distribution of MEFV mutations according to patients’ age of disease onset.

**Results:** A total of 708 FMF patients were included in the study: female/ male ratio 346/362 (48.9/51.1%). Mean age of patients at investigation was 12.3 ± 4.4 years, mean age at disease onset was 4.8 ± 3.4 years, and mean age at diagnosis was 7.3 ± 3.8 years. A group of patients with age at disease onset ≤2 years consists of 203 patients (mean age 1.5 ± 0.4). Mean age at disease onset was 4.4 ± 2.5 years and mean time to diagnosis was 2.9 years in the group of patients with age at disease onset ≤2 years. In the group of patients with age at disease onset between 2 and 5 years, which consists of 259 patients, mean patients’ age was 3.8 ± 0.9 years, mean age at diagnosis was 6.5 ± 2.9 years and mean time to diagnosis was 2.7 years. There were 192 patients in the patients’ group with age at diagnosis between 5 and 10 years. In this patients group, mean age was 7.7 ± 1.5 years, age at diagnosis was 9.7 ± 2.3 years and time to diagnosis was 2 years. Group of patients with age at diagnosis between 10 and 18 years included 53 patients with mean age at disease onset 12.7 ± 1.7, mean age at diagnosis 14.1 ± 1.9 and mean time to diagnosis of 1.4 years. It was shown that time to diagnosis decrease with increasing of patients’ age. M694V homozygosity was the most commonly seen mutation in the patients group of ≤2 years with 66 (32.5%) of 203 patients being positive for the mentioned mutation. Frequency of M694V homozygosity was 53/259 (20.5%), 25/192 (13.2%) and 7/53 (13.2%) in >2- ≤ 5, >5- ≤ 10 and >10- ≤ 18 patients’ group, respectively. In general, frequency of M694V mutation was 132/203 (65%), 136/259 (52.5%), 83/192 (43.2%) and 31/53 (58.5%) in ≤2, >2- ≤ 5, >5- ≤ 10 and >10- ≤ 18 patients’ group, respectively.

**Conclusion:** M694V mutation is particularly shown to be associated with younger age at disease onset and sever clinical course of the disease. In our study, the frequency of M694V homozygosity was significantly higher in patients ≤2 years, comparing to other patients group. Moreover, time to diagnosis was found to be longer in this group. The possible explanation is the lack of prominent clinical features and common clinical symptoms mimicking infections in childhood, e.g. fever, abdominal pain.

**Disclosure of Interest**

None Declared.

### P243 Childhood-onset SAPHO- a rare presentation

#### Laura O. Damian^1^, Cecilia Lazea^2^, Mihaela Sparchez^3^, Paulina Vele^1^, Laura Muntean^4^, Adriana Albu^5^, Simona Rednic^4^, Calin Lazar^2^

##### ^1^Rheumatology, Emergency Clinical County Hospital Cluj, Cluj-Napoca, Romania; ^2^1st Pediatric, University of Medicine and Pharmacy Cluj, Cluj-Napoca, Romania; ^3^2nd Pediatric, University of Medicine and Pharmacy Cluj, Cluj-Napoca, Romania; ^4^Rheumatology, University of Medicine and Pharmacy Cluj, Cluj-Napoca, Romania; ^5^2nd Internal Medicine, University of Medicine and Pharmacy Cluj, Cluj-Napoca, Romania

###### **Presenting author:** Laura O. Damian

**Introduction:** SAPHO syndrome (acronym for synovitis, acne, hyperostosis, pustulosis, osteitis) is considered to be the adult variant of chronic recurrent multifocal osteitis. However, SAPHO may rarely occur in children.

**Objectives:** To retrospectively characterize the patients with a juvenile-onset SAPHO presenting into a tertiary referral rheumatology unit, trying to identify their clinical features and clinical course.

**Methods:** The medical records of the patients with SAPHO syndrome, children or adults, seen over the last five years (between 2011 and 2016) were studied. In all, the age of onset, the familial history and the types of joint or bone involvement were recorded. After identification, the patients were called again to assess the evolution.

**Results:** We identified 5 cases of juvenile-onset SAPHO, all males, aged 11 to 15 yrs, with acne, enthesitis and arthritis. In all cases clavicular osteitis with pain and deformity was a constant finding. All patients had a familial history of acne (and in one case of SAPHO syndrome) and were HLA B27 -negative. The acne severity in children generally increased with the age as entering adolescence, but conglobated acne was seen as early as the age of 12. In a child with a 2-years history of seronegative symmetric arthritis, the onset of severe acne at 14, hydrosadenitis, sternoclavicular arthritis, clavicular osteitis and lower limbs bone pain and periostitis pointed to the diagnosis. Other associated features in this cases series were large vessel vasculitis, erythema nodosum, recurrent streptococcal infections and guttate psoriasis. The therapy consisted generally in NSAIDS, methotrexate and/or sulphasalazine. Glucocorticoids were generally avoided due to acne exacerbation. Clarithromycin courses were employed in all patients. Two former patients, young adults, had only minor flares and scarring acne after the age of twenty.

**Conclusion:** SAPHO may be encountered in children as well, with a clinical picture similar to that in adults; however, vasculitis and erythema nodosum could be more frequently associated. The frequence and intensity of disease flares may regress after adolescence.

**Disclosure of Interest**

None Declared.

### P244 One-year experience of a single autoinflammatory disease center in Brazil

#### Leonardo O. Mendonça^1^, Alessandra Pontillo^2^, Jorge Kalil^1^, Fabio M. Castro^1^, Myrthes T. Barros^3^

##### ^1^Department of Clinical Immunology and Allergy, University of São Paulo, Sao Paulo, Brazil; ^2^Department of Immunogenetics, University of São Paulo, Sao Paulo, Brazil; ^3^University of São Paulo, Sao Paulo, Brazil

###### **Presenting author:** Leonardo O. Mendonça

**Introduction:** Autoinflammatory diseases are recently recognized disorders that gained great clinical importance in recent years. In immune basis have theoretical absence of T-self-reactive cells and absence or low tittles of self-antibodies. Pathologic inflammation are, or should be in your majority secondary to innate immunity cells as the mutations found.

Clinically they are characterized by sterile inflammation attacks. By the time they were well defined only in the pediatric age group.

**Objectives:** This work proposes to review clinical features, main diagnostics and main drugs used to clinical control since the clinic creation in 2015.

**Methods:** All the patient’s data were collected spontaneously and aleatory since they were followed by us from 2015 on. Data were collected after consent term was assigned.

**Results:** A total of 28 patients were found followed at our unit of autoinflammatory disase. As a preferential adult service, almost 70% were older than 16 years at the moment they come to our service. Of all, 75% are female and among adults, woman is also majority representing 78% of them.The most common syndrome was FMF, with more than 21% carrying this diagnosis. Just one patient had a long disease history that had stablished lung and renal amyloidosis and now she is on canakinumab with good response.CAPS, NOD2 and Bechet had 10% each one. All the CAPS patients under 16 years are stable with canakinumab. The adult patient with CAPS presented with an atypical clinical manifestation also was submitted to canakinumab injection without new crisis. NOD2 mutations were found in 3 patients, all female and all had a persistent diarrhea, persistent positive calprotectin. Of all, 2 had skin rash, similar to psoriasis and one had arthralgia in crisis with unspecific skin rash. All receiving anti TNF agents. Bechet disease was found in one female under 16 years stable on colchicine. Older than 16 years we have followed 2 patients and one on tocilizumab and other high doses corticosteroids. CRMO was found in two patients and just the children required biologics as a permanent treatment due to the frequency and gravity of crisis.Just one family was diagnosed with TRAPS (TNF receptor associated periodic syndrome), one female adult patient and his son. The children get response to colchicine therapy and the adult required anti-TNF therapy.

**Conclusion:** The diagnosis of these rare disease is clinical and are a challenge to the general pediatrician and internal medicine. After the spread of genetic tests there have been a false definition that to make sure it’s an autoinflammatory disease we need genetic confirmation. That’s our fight in teaching to clinical immunology and allergy residents.

In adulthood the presentation is atypical and so many other inflammatory, infectious and neoplastic disease begin at the same age and can be an aid to diagnosis or a false diagnosis. Other consideration is that some patients haven’t been recognized for years and are nowadays with a different phenotype what makes the diagnosis more difficult.

According to our findings we suggest that adult’s patients that present with or without recurrent fever with one of the findings need further investigations to any autoinflammatory disease: recurrent sterile pleural, pericardial and peritoneal effusion; chronic diarrhea with positive calprotectin; cronic inflammatory central nervous system; chronic recurrent osteomyelitis and chronic neutrophilic dermatosis.

**Disclosure of Interest**

None Declared.

### P245 Cronic non-bacterial osteomyelitis (CNO) and polyarteritis nodosa: a report of 2 cases with an unusual association

#### Manuela Pardeo, Virginia Messia, Fabrizio De Benedetti, Antonella Insalaco

##### Division of Rheumatology, Ospedale Pediatrico Bambino Gesù IRCCS, Roma, Italy

###### **Presenting author:** Manuela Pardeo

**Introduction:** Chronic nonbacterial osteomyelitis (CNO) is the most common autoinflammatory bone disorder in childhood with unknown etiology. CNO may manifest with inflammatory bone lesions alone or in association with other disorders such as psoriasis, palmoplantar pustulosis and inflammatory bowel disease. We describe the occurrence of CNO in association with polyarteritis nodosa (PAN), a systemic necrotizing vasculitis targeting medium-sized arteries of unknown pathogenesis.

**Objectives:** To describe the association of CNO e PAN in two pediatric patients.

**Methods:** We retrospectively collected clinical data of two patients with CNO associated with polyarteritis nodosa

**Results: Case 1** An 10-year-old boy presented with history of 2 prolonged episodes of fever associated with transient rash and swelling in the 3rd interphalangeal joint of the right hand. Both episodes resolved, about one month later, after treatment with ibuprofen. He was admitted to our hospital for the third episode characterized by prolonged fever, malaise, arthralgia, myalgia, lymphadenopathy and elevated inflammatory markers. We performed whole-body magnetic resonance (WB-MRI) and TC-99 bone scintigraphy (BS) that revealed multifocal sclerotic bone lesions. Bone biopsy showed findings consistent with chronic osteomyelitis. After 2 weeks since the hospitalization he developed subcutaneous nodules suggestive of vasculitis, with histological features of PAN. He was treated with glucocorticoids and mycophenolate mofetil as for PAN and showed a very satisfactory response. At 6 months the patient was completely asymptomatic with decrease in the total number of bone lesions at WB-MRI. **Case 2** An 8-year-old girl had been seen in our division since 3 years of age because of prolonged fever, myalgia, abdominal pain, diarrhea, vomit and elevated inflammatory markers. A skin biopsy, performed because of the later appearance of vasculitic rash, was suggestive for PAN. She was successfully treated with intravenous pulses of metilprednisolone and cyclophosphamide. She reached remission and discontinued all treatments after two years. Three years later she developed severe persistent back pain with sacroiliitis, with no skin or other features suggesting vasculitis, unresponsive to non-steroidal anti-inflammatory drugs. WB-MRI and BS showed multiple inflammatory bone lesions suggestive of CNO that was confirmed by bone biopsy. She was started on pamidronate with good response.

**Conclusion:** To the best of our knowledge, the association between CNO and PAN has never been reported. This unusual observation raises some questions. It remains to be defined whether this is a casual association, whether the association of PAN with a CNO, or in general bone inflammation, may represent a defined disease entity or whether CNO might represent a symptom common to other inflammatory diseases. In the third hypothesis, PAN should be added among the systemic diseases that may be associated with CNO

**Disclosure of Interest**

M. Pardeo: None Declared, V. Messia: None Declared, F. De Benedetti Grant / Research Support from: Novartis, Pfizer, Novimmune, Hoffmann-La Roche, SOBI, Abbvie, A. Insalaco: None Declared.

### P246 Vitamin D deficiency in children with PFAPA syndrome

#### Giorgia Malighetti^1^, Chiara Gorio^1^, Francesca Ricci^2^, Ilaria Parissenti^2^, Paola Montesano^2^, Barbara Bonafini^2^, Veronica Medeghini^2^, Marco Cattalini^2^

##### ^1^Clinical and Experimental Sciences, Immunology and Rheumatology Unit, University of Brescia, Brescia, Italy; ^2^Clinical and Experimental Sciences, Pediatric Clinic, University of Brescia, Brescia, Italy

###### **Presenting author:** Marco Cattalini

**Introduction:** Periodic fever, aphthous stomatitis, pharyngitis and cervical adenitis (PFAPA) is an autoinflammatory disease characterized by regularly recurrent fever episodes with pharyngitis, cervical adenitis and/or oral aphthosis. The pathogenesis of the disease is still a matter of debate, recently some data seem to suggest a possible correlation with vitamin D deficiency

**Objectives:** these are the preliminary results of an ongoing study to evaluate 25-hydroxyvitamin D serum concentrations and sonographic bone density in children with PFAPA syndrome and to assess the effects of vitamin D supplementation on the disease course

**Methods:** We enrolled 20 patients (17 males, 3 females) fullfilling Thomas PFAPA criteria. At the time of enrollment we collected demographic, anthropometric and clinical data and we measured 25-hydroxyvitamin D, calcium, phosphate, PTH and ALP serum levels. We also evaluated bone mineral density by means of phalangeal Quantitative Ultrasound (DBM Sonic Bone Profiler) that provides ADSOS (amplitude-dependent speed of sound) Z-score and BTT (bone transmission time) Z-score as bone density parameters. The reduction of those parameters lower than -1 SD suggests a decrease of bone density, with values lower than 2 SD indicative of osteoporosis. Patients with vitamin D levels lower or equal to 20 ng/mL were instructed to take vitamin D supplementation (600 UI/day). The parents of the children were then provided with a standardized diary to register the clinical course of PFAPA and were asked to come back 6 months later for evaluation

**Results:** At baseline 13/18 PFAPA children had a vitamin D deficiency. In two children the parents refused to test the vitamin D levels. Quantitative ultrasound showed a reduced bone mineral density in 10 children, with 6 children showing ADSOS and BTT z-score lower than 1SD and 4 children showing values lower than 2 SD. 3 patients had reduced bone mineral density with normal levels of vitamin D.

All the patients with vitamin D deficiency were put on vitamin D, no matter of the results of bone mineral density evaluation. 8/20 patients completed the 6 months f/up, 8 are still in the follow/up period, 4 were lost at follow/up for clinical remission after tonsillectomy (3 were on vitamin D supplementation). Of the 8 patients completing the 6 months f/up, 5 patients had vitamin D deficiency at baseline and 3 patients had normal vitamin D levels. The 5 children who received supplementation had normalization of the 25-hydroxyvitamin D serum level. Sonographic parameters reached normal values only in one patient, while one patient with normal bone mineal density at baseline showed worsening of the sonographic parameters. In 2 children on vitamin D supplementation we noticed the remission of disease, in 2 children there was a reduction in the frequency of PFAPA episodes, 2 didn't show any significant clinical modification. The three patients without vitamin D deficiency at baseline, showed normal levels of vitamin D at follow/up and no changes in disease characteristics. There was no apparent correlation between bone mineral density parameters and disease outcome.

**Conclusion:** Although very preliminary and limited to a small number of patients, our data seem to confirm a role for vitamin D deficiency on the occurrence of PFAPA syndrome. Vitamin D supplementation may be a possibility in children with PFAPA, at least in those with concurrent vitamin D deficiency. Larger studies are needed to further confirm this role and to better understand a possible role of bone mineral density determination on such children.

**Disclosure of Interest**

None Declared.

### P247 Ethosuximide-induced lupus-like syndrome: a case report

#### Lucio Giordano^1^, Giulia Zani^2^, Rosalba Ferraro^3^, Donatella Vairo^3^, Silvia Giliani^3^, Marco Cattalini^2^

##### ^1^Child Neurology and Psichiatry, Spedali Civili di Brescia, Brescia, Italy; ^2^Clinical and Experimental Sciences, Pediatric Clinic, University of Brescia, Brescia, Italy; ^3^Department of Pathology, University of Brescia, Angelo Nocivelli Institute for Molecular Medicine, Brescia, Italy

###### **Presenting author:** Marco Cattalini

**Introduction:** Drug-induced lupus erythematosus is an autoimmune disorder caused by a drug overreaction. To our knowledge only one case of possible correlation between the use of ethosuximide and SLE has been described so far

**Objectives:** To describe a case of drug induced lupus and the use of interferon signature as a marker of disease activity in clinical practice

**Methods:** we describe a case of drug induce lupus followed clinically, with routine immunological work-up and with serial determinations of interferon signature, by means of quantitative reverse transcription PCR, measuring 6 different interferon I stimulated genes (IFI27, IFI44L, IFIT1, ISG15,RSAD2,SIGLEC1), as previously described^1^

**Results:** M.L. is a 16-year-old girl who was suffering since birth from a very mild cognitive delay, focal epilepsy and ESES (continuous spike wave epilepsy during sleep) since 9 y/o. Extensive neurological work-up failed to revealed any underlying disease. The girl was put on Ethosuximide with good seizures control. Two years later she started to complain from generalized arthralgia and myalgia, transient hands and feet swelling, and recurrent, evanescent rash on cheeks. The girl was then referred to our rheumatology unit. Physical exam was normal but lab-works showed ANA 1:640 homogeneous, positive anti ds-DNA antibodies, reduced complement levels and mild proteinuria. We also run an in-house interferon signature assay, revealing a very high expression of IFN-stimulated genes, similarly to children with clear-cut SLE. For this reason a drug-induced lupus was hypothesized and the collegial decision was to stop ethosuximide. In the following 6 months the girl remained free of seizures, the myalgia and rash disappeared, all the laboratory abnormalities normalized. We also found a decrease in the interferon signature, testifying a lower expression level of the 6 interferon-stimulated genes analyzed

**Conclusion:** We herein present a case of what we believed was a drug-induced lupus. Interferon signature may be very useful, together with classic immunological work-up, to follow the disease course in these patients.

^1^Rice GI et al. “Assessment of interferon-related biomarkers in Aicardi-Goutières syndrome associated with mutations in TREX1, RNASEH2A, RNASEH2B, RNASEH2C, SAMHD1, and ADAR: a case-control study”, Lancet Neurol. 2013 Dec;12(12):1159-69

**Disclosure of Interest**

None Declared.

### P248 Platelet count and MPV as predictive markers of atherosclerosis in familial Mediterranean fever

#### Maria Cristina Maggio, Girolamo Luppino, Giovanni Corsello

##### University Department Pro.Sa.M.I. “G. D’Alessandro”, University of Palermo, Palermo, Italy

###### **Presenting author:** Maria Cristina Maggio

**Introduction:** Familial Mediterranean Fever (FMF) is an auto inflammatory syndrome, characterized by recurrent febrile episodes, arthritis, oral aphthous stomatitis, rash, serositis, abdominal and thoracic pain. Long-term outcome is conventionally linked to the severity of the recurrent attacks and to the risk of systemic amyloidosis. However recent studies highlighted the role of chronic inflammatory diseases in the insurance of atherosclerosis. Risk factors for atherosclerosis are also recently identified in a higher medium platelet volume (MPV).

**Objectives:** We evaluated platelet parameters (MPV; platelet distribution width: PDW) in children affected by FMF and considered them as indexes or atherosclerotic risk. We evaluated the efficacy of colchicine treatment on clinical and biochemical parameters.

**Methods:** We enrolled 33 children (8,6 ± 4,6 years; 12 F and 21 M), followed since 2012, with the diagnosis of FMF, confirmed by genetic study. We evaluated platelet parameters (count, MPV, PDW), CRP, ESR, leukocyte count, neutrophils percentage, serum amyloid-A (SAA) during the acute phase, during the remission and far from febrile events., All the patients, with the exception of 5, receive colchicine treatment. Biochemical parameters were matched with those of 23 healthy controls, matched for sex and age.

**Results:** Platelet count was significantly higher in acute phase (379,800 ± 111,600) than in free-disease periods (328,400 ± 88,200) and in controls (314,200 ± 72,800). MPV was lower in acute phase (8,18 ± 0,85) than in free-disease periods (8,22 ± 0,67); these parameter was significantly lower than in the control group (8,5 ± 0,82). PDV was lower in acute phase (37,97 ± 15,45) than in free-disease periods (45,1 ± 9,2) as in control group (45,96 ± 6,07). In acute phase, MPV was directly correlated with CRP, ESR, SAA; showed a statistically significant correlation with MCV (p < 0.05). During the free-disease periods, MPV was directly correlated with: CRP, ESR; inversely with SAA and MCV (p < 0.05).

SAA was maintained in the normal range in free-disease periods in all the patients, with the exception of 5 who had an incomplete response to colchicine.

**Conclusion:** Long-term complications of FMF include kidney amyloidosis and atherosclerosis, however we do not have a valid predictive marker of thrombosis in these children. An increase in MPV and PDW, expression of platelet activation, can predict an increased risk of thrombosis. In our children these parameters are adequately controlled by colchicine, preventing the endothelial dysfunction.

**Disclosure of Interest**

None Declared.

### P249 PAPA syndrome with latent tuberculosis successfully treated with canakinumab

#### Maria Isabel Gonzalez Fernandez^1^, Berta Lopez Montesinos^1^, Adriana Rodriguez Vidal^1^, Juan I Arostegui Gorospe^2^, Inmaculada Calvo Penades^1^

##### ^1^Pediatric Rheumatology Unit, Hospital Universitario y Politecnico La Fe, Valencia, Spain; ^2^Immunology. Biomedical Diagnostic Center, Hospital Clinic, Barcelona, Spain

###### **Presenting author:** Maria Isabel Gonzalez Fernandez

**Introduction:** Pyogenic sterile arthritis pyoderma gangrenosum and acne (PAPA) syndrome is a rare autosomal dominant autoinflammatory disease caused by mutations in PSTPIP1 gene.

Steroids, anti-TNFa and anti-IL-1 agents have been proposed as treatment options, with variability in response being observed.

There is evidence that IL-1B secretion in PAPA is increased and correlates with disease activity.

**Objectives:** To present the case of a child with PAPA syndrome and latent tuberculosis successfully treated with canakinumab.

**Methods:** An 8-year-old male arrived to our Pediatric Rheumatology Unit diagnosed with juvenile idiopathic arhritis (JIA). The patient presented recurrent arthritis of his knees from the age of 3 years. The first episode was treated as septic arthritis because of elevated acute phase reactants and purulent synovial fluid. Cultures were negative. Subsequently he was treated with intraarticular steroid injections, systemic steroids and methotrexate, persisting activity of the disease. At the age of 4 years the patient developed ulcerated cutaneous lesions in right underarm region and groin that lasted for several months. He also had history of frequent intense local vaccine reactions. His mother was diagnosed with a systemic JIA and his grandmother with a rheumatoid arthritis. Father with psoriasis.

When the patient was assessed in our unit, he presented arthritis of his left knee. The synovial fluid was purulent with 131090 leucocytes (94% neutrophils). The blood tests showed elevated acute phase reactants. The Mantoux test was positive with 17 mm of induration. The possibility of a pathergy phenomenon was thought, but IGRA tests were also positive. Chest X-ray and high resolution thoracic tomography did not show pathological findings. Synovial fluid cultures were negative, including mycobacteria, and PCR of *Mycobacterium tuberculosis* was not detected. In the gastric fluid there was no presence of acid-fast bacilli and cultures were negative. He was treated with isoniazid because of latent tuberculosis infection.

**Results:** The study of mutations in the PSTPIP1 gene revealed the mutation E250Q in heterozygosity, that was also present in the mother and in the grandmother.

An intraarticular steroid injection was performed in the knee and treatment with canakinumab 2 mg/Kg/4 weeks was started after 4 weeks of treatment with isoniazid, with good response.

The patient has received treatment with canakinumab now for 34 months with good control of the articular disease. One year after treatment onset he had eczematous recurrent lesions in antecubital flexure at site of puncture for blood test, that were thought to be a clorhexidine contact dermatitis. No other cutaneous lesions apart from dry scaly lesions in the scalp and eczematous psoriasiform lesions in the pinna have appeared during the follow-up. Normal blood tests with no elevation of inflammatory parameters. Good physical growth. The treatment has been well tolerated without significant infections. The patient is currently on canakinumab 2 mg/Kg/8 weeks.

**Conclusion:** We present a family with PAPA syndrome with one member treated with canakinumab and good control of the disease during 34-month follow-up on treatment. Long-term efficacy and control of cutaneous disease, that is described to appear later on the course of the disease, will be established during follow-up.

A particular feature in this case is the difficulty related to the latent tuberculosis infection because of the possible initial interpretation of Mantoux test as pathergy phenomenon and the treatment with isoniazide before starting biological treatment.

**Disclosure of Interest**

None Declared.

### P250 Successful use to tocilizumab to treat pigmentary hypertrichosis and non-autoimmune insulin-dependent diabetes mellitus (PHID) syndrome

#### Nadia K. Rafiq^1^, Karen Wynne^1^, Khalid Hussain^2^, Paul A. Brogan^1^

##### ^1^Paediatric Rheumatology University College London, Institute of Child Health, London, UK; ^2^Developmental Endocrinology Research Group, Molecular Genetics Unit, University College London, Institute of Child Health, London, UK

###### **Presenting author:** Nadia K. Rafiq

**Introduction:** Autoinflammation is increasingly recognised as a feature of Pigmentary hypertrichosis and non-autoimmune insulin-dependent diabetes mellitus (PHID) syndrome. We previously reported a 16-year-old girl of Pakistani origin with PHID syndrome, associated with severe autoinflammation that was recalcitrant to treatment with blockade of interleukin-1 (IL-1) and tumor necrosis-α (anti TNF)^1^. Herein we report the therapeutic response to IL-6 blockade with Tocilizumab.

**Objectives:** To report the first case of successful treatment of PHID syndrome using IL-6 blockade with Tocilizumab.

**Methods:** Retrospective medical records review of a single case of PHID, caused by *SLC29A3* mutation, documenting clinical and serological response to Tocilizumab over 23 months. We noted improvement in clinical features such as change in sclerotic skin, reported levels of fatigue, and acute phase response (C-reactive protein [CRP], erythrocyte sedimentation rate [ESR] and serum Amyloid A [SAA]).

**Results:** We have previously published on the lack of response to Anakinra, Adalimumab and Methotrexate^1^. Immediately prior to commencing Tocilizumab, there was evidence of significant systemic inflammation: SAA 178 mg/L; CRP 54 mg/L; and ESR 86 mm/hr. We therefore decided to trial treatment with intravenous Tocilizumab 8 mg/kg, every 2 weeks. Within 12 weeks of treatment her inflammatory markers drastically improved: CRP <5 mg/L, ESR 22 mm/hr and SAA 8.4 mg/L. Her dose was increased to 12 mg/kg after three initial doses, due to ongoing fatigue and sclerotic skin changes. She subsequently reported marked clinical improvement in her energy levels, improved appetite, reduced fevers, less skin tightness and night sweats. Methotrexate was discontinued 9 months after commencing Tocilizumab due to continued excellent clinical and serological response, which has been sustained over 23 months. The cutaneous lesions remain significantly improved (decreased sclerosis and hypertrichosis); her weight and height have improved but remain below the 0.4th centile. Tocilizumab had no impact on her diabetes.

**Conclusion:** PHID syndrome is associated with recessive mutations in *SLC29A3* which encodes for the equilibrative nucleoside transporter hENT3 expressed in mitochondria, causing PHID and H syndromes, familial Rosai-Dorfman disease, and histiocytosis-lymphadenopathy-plus syndrome.

Tocilizumab is a humanised, monoclonal, antihuman IL-6 receptor (IL-6R) antibody that binds to membrane and soluble IL-6R, inhibiting IL-6–mediated signaling. It has been used to successfully treat rheumatoid arthritis, systemic JIA, and polyarticular JIA. There are no published reports of the treatment of PHID syndrome using IL-6 blockade.

We report the first case of PHID syndrome treated with tocilizumab, with successful outcome in relation to systemic inflammation, cutaneous skin lesions, and overall quality of life. Linear growth and diabetes did not improve; however, no adverse events occurred. Whilst the mechanism of autoinflammation of PHID remains uncertain, we suggest that tocilizumab should be the first choice when considering treatment of the autoinflammatory component of this genetic disease. At the time of writing, her similarly affected sister is now also commencing tocilizumab.

*Written informed consent was taken for the publication of this report.*

**Reference**

1. Sennippan S et al. Pigmentary hypertrichosis and non-autoimmune insulin dependent diabetes mellitus (PHID) syndrome is associated with severe chronic inflammation and cardiomyopathy, and represents a new monogenic autoinflammatory syndrome. Journal of Pediatric Endocrinology and Metabolism 2013; 26:877–882

**Disclosure of Interest**

None Declared.

### P251 Utility of Jansson score in a cohort of patients with chronic non-bacterial osteitis in Asia

#### Elizabeth Ang^1^, Nicholas Ng^2^

##### ^1^Paediatrics, Singapore, Singapore; ^2^Khoo Teck Puat-University Children's Medical Institute, Singapore, Singapore

###### **Presenting author:** Nicholas Ng

**Introduction:** We describe a cohort of 6 patients in Singapore with Chronic Non-bacterial Osteitis (CNO) and retrospectively calculated their Jansson Score.

**Objectives:** We aim to document the characteristics of CNO in an Asian Paediatric population and describe the utility of the Jansson Clinical Score for these patients.

**Methods:** We looked retrospectively into the case records of six patients who have been undergoing treatment for CNO in the Paediatric Rheumatology Unit of the University Hospital. Inclusion criteria were the diagnosis of CNO, evidence of at least one lesion of osteitis confirmed by imaging and onset of symptoms before age 18 years.

**Results:** Six patients (4 males) with CNO were identified, and their diagnosis confirmed by the exclusion of other conditions and response to therapy for CNO. Age range at diagnosis was 5 to 16 years. Duration of follow-up was 8 months to 8 years. The condition was unifocal in 3 (50%) of the patients. The clavicle was the most frequently affected bone (50%), followed by long bones (femur, tibia), pelvis and small bones of the foot. Fifty percent of the patients had concomitant arthritis. Five had fever at presentation. Three had extra-osseous manifestations in the form of recurrent aphthous ulcers. All patients underwent radio-isotope bone scans and 5 received targeted MRIs. Only one patient underwent a bone biopsy. All received initial therapy with NSAIDs, with 2 patients receiving therapy with NSAIDs alone. The other 4 patients received methotrexate (4), anti-TNF alpha agents (2) and pamidonate (1). Five patients had treatment with prednisolone during the course of their disease. At the last medical encounter, disease was in remission in 2 patients (1 without medicine, 1 while on infliximab). Jansson defines scores of 0-28 as “probably not CNO”, 29 to 38 as “uncertain diagnosis” and 39 to 63 as “probably CNO”. Our patients had Jansson Clinical Scores of 26 to 54. Based on this score, only one patient would have received the diagnosis of “probably CNO”, 4 with “uncertain diagnosis” and 1 with the score of “probably not CNO”. The patient who received the diagnosis of “probably not CNO” had a score of 26, mulftifocal disease, received treatment with NSAIDs, prednisolone, methotrexate, pamidronate and entered remission finally with Etanercept alone.

**Conclusion:** The Jansson Clinical Score for CNO predicted the diagnosis only in 1 of 6 of our patients. Majority of the patients received scores within the category of “uncertain diagnosis” while the patient whose clinical course was most consistent with difficult to treat multifocal disease requiring biologics had the lowest score. We propose that in our population, the score limits within each category be lowered in order to more accurately predict patients with CNO, and avoid unnecessary bone biopsies that would otherwise be indicated if this score was applied.

**Disclosure of Interest**

None Declared.

### P252 Investigating the frequency of restless leg syndrome and growing pain in familial Mediterranean fever

#### Ayla Kacar, Ozge Altug Gucenmez, Balahan Makay, Sevket Erbil Unsal

##### Division of Pediatric Rheumatology, Department of Pediatrics, Faculty of Medicine, Dokuz Eylul University, Izmir, Turkey

###### **Presenting author:** Ozge Altug Gucenmez

**Introduction:** The majority of the Familial Mediterranean Fever (FMF) patients complains about leg pain that induced by exercise and this is also regarded among the minor diagnostic criteria of the disease itself. Restless Leg Syndrome (RLS) is a disorder characterized by irritating feeling in the legs and the uncontrolled stimulation of moving the legs while resting in order to remove this feeling. Growing pain (GP) is also one of the frequently seen pain syndromes of the childhood period.

**Objectives:** As to our knowledge, there is no study evaluating the frequency of RLS and GP in FMF patients. Therefore, the objective of this study was to investigate the frequencies of RLS and GP in children who have FMF and also to evaluate the effects of these symptoms on quality of life and sleeping quality.

**Methods:** Sixty patients with FMF were consecutively included in the study. Demographic characteristics, FMF family history, the onset age, follow-up periods, disease symptoms and findings of the FMF patients were recorded. Seventy healthy children who had no chronic diseases were included in the study as the control group. RLS, GP, quality of life and sleeping habits were evaluated by standard forms and questionnaires in both FMF patients and control group.

**Results:** The mean age was 8.94 ± 3.89 for the FMF patients and 9.8 ± 3.7 for the controls (p = 0.20) and there was no difference in the genders between groups (p = 0.43). No significant differences were detected among patients with FMF and healthy children regarding GP and RLS diagnosis criteria (p = 0.44, p = 0.23, respectively). Total sleeping score was 48.1 ± 8 in FMF group and 45.3 ± 7 in the control group (p = 0.03). There was no significant difference between the sleeping scores of the patients with and without growing pain in both FMF patients and control group (p = 0.18, p = 0.29, respectively). Similarly, there was no significant difference between the sleeping scores of the patients with and without RLS in both FMF patients (p = 0.86) and control group (p = 0.65). Moreover; there was no significant difference between the sleeping scores of the patients with and without exercise induced leg pain complaints in the patient group (p = 0.22). There was a high ratio of GP in patients with exercise induced leg pain. While, 8 of 27 patients with exercise induced leg pain fulfilled GP criteria, this ratio was 2 of 33 patients without exercise induced leg pain (p = 0.015). Quality of life forms of 22 patients were filled by their parents because the patients were under the age of six. Thirty-eight patients filled these surveys both on their own and with their parents. A negative significant correlation was found between total sleeping score and total parent score of quality of life in the FMF group (r = -0.45, p < 0.01). Total parent score of quality of life was significantly and negatively correlated with the age of the patient, dosage of colchicine and disease severity score.

**Conclusion:** It seems that fulfilling RLS and growing pain criteria are similar in FMF patients and healthy children. Also, it was found that the ratio of fulfilling the growing pain criteria in the patients with exercise induced leg pain complaints was significantly higher when compared to the children without exercise induced leg pain. However, no significant relationship was detected between exercise induced leg pain and RLS. Interestingly, RLS, GP and exercise induced leg pain did not have an effect on the sleep quality.

**Disclosure of Interest**

None Declared.

### P253 The frequency of celiac disease in children with familial Mediterranean fever

#### Yasin Sahin^1^, Kenan Barut^2^, Tufan Kutlu^1^, Fugen Cullu-Cokugras^1^, Sezgin Sahin^2^, Amra Adrovic^2^, Hasret Ayyildiz-Civan^1^, Ozgur Kasapcopur^2^, Tulay Erkan^1^

##### ^1^Pediatric Gastroenterology, Istanbul University, Cerrahpasa Medical School, Istanbul, Turkey; ^2^Pediatric Rheumatology, Istanbul University, Cerrahpasa Medical School, Istanbul, Turkey

###### **Presenting author:** Ozgur Kasapcopur

**Introduction:** Familial Mediterranean fever (FMF) is a chronic autoinflammatory disease that is inherited in an autosomal recessive manner and characterized with recurrent fever, polyserositis attacks such as abdominal and chest pain. Celiac disease is histologically characterized with villous atrophy as a consequence of overactivation of immune system in genetically susceptible individuals upon exposure to gluten. Some similar symptoms and signs like diarrhea, abdominal pain, arthralgia, and arthritis serve as diagnostic challenges in differential diagnosis of these two diseases. Consequently it is possible to misdiagnose celiac disease as FMF or overlook symptoms and signs of celiac disease during follow-up of FMF patients.

**Objectives:** We aimed to screen celiac disease in children with FMF.

**Methods:** This prospective study is conducted from October 2015 to March 2016 in pediatric rheumatology and gastroenterology clinic of a tertiary referral center. We have enrolled 303 children with FMF randomly and the same pediatric gastroenterology specialist performed both the face-to-face negotiation and physical examination for celiac disease. Then, serum levels of total immunoglobulin A (Ig A) and anti-tissue transglutaminase Ig A antibody (anti-tTG A) were measured in all of the patients regardless of a symptom or sign that is suspicious for celiac disease. Positive children for anti-tTG A were further searched for IgA anti-endomysial antibodies (IgA-EmA). For definitive diagnosis, small intestine biopsy was performed to the patients with positive IgA-EmA results.

**Results:** Mean age, height and weight of the 303 children with FMF were 12.06 ± 4.23 years, 148.23 ± 21.19 cm and 43.64 ± 18.90 kg, respectively. Males were constituting 50.2% (n = 152) and females were 49.8% (n = 151) of the cohort. The mean disease duration was measured as 5.47 ± 3.46 years. While the mean hemoglobin and total Ig A levels were 12.70 ± 1.37 g/dL and 140.72 ± 69.4 mg/dL, respectively; the mean anti-tTG A level was 3.22 ± 18.22 U/mL. Merely 9 patients were positive for anti-tTG A level and these patients further evaluated for IgA-EmA positivity. Only one patient had a positive IgA-EmA result and so, has been deserved advanced diagnostic procedures for celiac disease. Although there has been a suspected history with the complaints of abdominal distension since birth, both the gastroduodenoscopy and histology did not reveal anything predicting celiac disease. Moreover, the patient was not carrying the HLA-DQ2/DQ8 allele.

**Conclusion:** Neither clinical findings nor laboratory results were consistent with celiac disease in our 303 FMF children. Hence, we conclude that there is no association between FMF and celiac disease.

**Disclosure of Interest**

None Declared.

### P254 Early phenotypic presentation of Blau syndrome in Arab ethnicity

#### Reem Abdawani^1^, Sana Al Zuhbi^2^, Eiman Abdalla^1^

##### ^1^Child Health, Sultan Qaboos University Hospital, Muscat, Oman; ^2^Ophthalmology, Sultan Qaboos University Hospital, Muscat, Oman

###### **Presenting author:** Reem Abdawani

**Introduction:** Blau syndrome (MIM# 186580) is a rare granulomatous autoinflammatory disorder that affects primarily the skin, joints and eyes. It is a dominantly inherited condition due to mutations in the caspase recruitment domain gene CARD15/NOD2 mapped on the chromosomal region 16q12.1–13. Mutations in this gene have also been associated with early onset sarcoidosis and increased susceptibility to Crohn’s. To date, more than 139 sequence variant mutations have been described in the Infever registry, predominantly from European countries. Several cases have been reported from Japan, China, Mexico and India.

**Objectives:** To describe the first case of Blau syndrome of Arab/Middle Eastern Ethnicity from Sultanate of Oman presenting in infancy.

**Methods:** An 8-month-old child, born to consanguineous parents presented with history of undocumented fever and macular papular skin rash since 2 months of age. He developed extensive severe symmetrical boggy polyarthritis at 4 months of age and was found to have anterior uveitis on routine screening. His skin rash and anterior uveitis were compatible with granulomatous vasculitis (Fig. 1) and granulamouts uveitis (Fig. 2). Treatment consisted of intravenous methylprednsilone, oral prednisolone, subcutaneous methotrexate. However, due to ongoing active arthritis, Adalimumab was introduced at 18 month of age.

**Results:** Genomyic DNA analysis identified a heterozygous missense mutation, NM_022162.2:c.2010C > G (p.Asn670Lys) in exon 4 of the NOD2 gene.

**Conclusion:** The rarity of Blau syndrome has made it challenging to fully characterize the disease. However, with NOD mutation being cataloged regularly in the Infevers registry and the creation of Blau international registry and Eurofever registry, it has provided novel insights to the need for the development of effective targeted therapies.

**Disclosure of Interest**

None Declared.

### P255 Anti-tumor necrosis factor therapy in patients with Blau syndrome: a single-center observational study

#### Ricardo A. Russo, María M. Katsicas

##### Immunology & Rheumatology, HOSPITAL DE PEDIATRÍA GARRAHAN, Buenos Aires, Argentina

###### **Presenting author:** Ricardo A. Russo

**Introduction:** Blau syndrome (BS) is a rare monogenic disease with autosomal-dominant inheritance resulting from mutations in nucleotide oligomerization domain 2 (NOD2). It is phenotypically characterized by multiorgan granulomatous inflammation classically presenting as arthritis, uveitis, and skin rash. Treatment is empiric and consists of nonsteroidal anti-inflammatory drugs, corticosteroids (CS), and immunosuppressive agents. Activation of NF-kB is involved in its pathogenesis, suggesting that biologic agents targeted at blocking tumor necrosis factor may be efficacious.

**Objectives:** To report the effectiveness and safety of TNF-inhibitors (TNFi) in a cohort of patients with Blau syndrome.

**Methods:** Retrospective review of prospectively collected data. Patients with BS who received treatment with TNFi were included. Demographic, clinical and genetic data were collected. Therapeutic response was evaluated using a (yet unvalidated) composite key symptom score that captures active disease in 13 domains (scored as 0, 1, or 2 according to severity): fever, rash, arthritis, tenosynovitis, uveitis, hepatomegaly/splenomegaly, lymphadenopathy, syaladenitis, interstitial lung disease, arterial hypertension, renal disease, pericarditis, and erythrocyte sedimentation rate. The range of the BS disease activity score (BSDAS) is 0 to 26. Adverse events (AEs) were recorded throughout the observation period,

**Results:** In a cohort of 6 patients with BS 4 children have been treated with TNFi. One of them received only one 5 mg/kg injection of infliximab during his last 24 hours before death due to severe multiorgan failure. The remaining 3 patients (2 boys) are the focus of this report. Mutations in NOD2 were identified in 2 patients (R334Q and 1007 fs), while one was wild type. They all had evidence of non-caseous, giant cell-granulomata and exclusion of infections, Crohn´s disease and primary immunodeficiency. Age of onset was (median) 9 months, age at start of TNFi 24 months. They had all failed to respond to CS and methotrexate (MTX). Etanercept (ETA) up to 1 mg/kg/dose twice weekly was the initial TNFi in 2 patients (in whom it was switched to adalimumab (ADA) 20 mg every 2 weeks later on), while ADA was the initial TNFi in one patient. Patients received TNFi for 36 months (ETA 15 months, ADA 21 months). TNFi were administered along with MTX and varying doses of CS. At baseline arthritis was present in 3 patients, uveitis and splenomegaly in 2 patients, rash and syaladenitis in 1 patient each; BSDAS was 5. After 12 months of therapy splenomegaly was present in 2 patients, while arthritis, tenosynovitis, fever, rash, and uveitis occurred in 1 patient each; BSDAS was 4. After 24 months BSDAS was 1. Switching to ADA did not result in better outcomes except for remission of uveitis in 1 patient and lesser need for CS. Upper respiratory tract infections occured in 2 children and Wilms´ tumor developed in 1 patient while on ADA.

**Conclusion:** The use of TNFi was partially effective in the treatment of patients with BS. Long-term use of ADA appears to induce a better response. Malignancies may occur in patients with BS on TNFi. Alternative effective and safe targeted therapies should be designed to control the severe clinical picture of BS.

**Disclosure of Interest**

None Declared.

### P256 The therapeutic challenge of DITRA syndrome

#### Roberta Caorsi^1^, Francesca Minoia^1^, Gianmaria Viglizzo^2^, Alice Grossi^3^, Sabrina Chiesa^1^, Paolo Picco^1^, Angelo Ravelli^1^, Marco Gattorno^1^

##### ^1^Second division of Pediatrics, G. Gaslini Institute, Genova, Italy; ^2^Dermatology division, G. Gaslini Institute, Genova, Italy; ^3^Department of Human Genetics, G. Gaslini Institute, Genova, Italy

###### **Presenting author:** Roberta Caorsi

**Introduction:** DITRA syndrome is a severe autoinflammatory disease, characterised by pustular psoriasis, due to autosomal recessive mutations in the *IL36RN* gene, encoding the IL-36 receptor antagonist. Due to the rarity of this severe condition, the response to treatment is still unclear.

**Objectives:** To describe the clinical characteristics and response to treatment in a patient with severe DITRA syndrome.

**Methods:** The clinical hystory of a DITRA patient has been analysed.

**Results:** The child at the age of 3 years, following an upper-airways viral infection, presented generalised pustular lesions associated with fever and poor general conditions, requiring iv antibiotics and high doses of steroids. The diagnosis of pustular psoriasis was confirmed by a skin biopsy and retinoid acid was started, without a good clinical response; it was therefore replaced with high doses of cyclosporine (4 mg/kg/day). However any attempt to reduce the steroidal treatment was followed by a worsening of the skin lesion, even after the association of retinoid acid with cyclosporine. The Sanger analysis of I*L36RN* revealed a compound heterozygosis (P76L and S113L), confirming the diagnosis of DITRA syndrome. Do to the subsequent worsening of the skin lesion, followed by the appearance of fever and hypovolemic shock, despite high doses of antibiotics and steroids, treatment with anakinra was started at the dosage of 5 mg/kg/day with a quick amelioration of the clinical conditions allowing a progressive reduction of steroidal treatment. After two months of complete wellbeing, when the steroidal treatment was reduced at the dosage of 0.5 mg/kg/day, following a systemic infection, the child presented the reappearance of the skin manifestations with fever. In spite of the antibiotics and the increase of the dosage of anakinra (3 mg/kg twice a day) the clinical conditions worsened requiring high doses of steroidal treatment. Anakinra was therefore withdrawn. In light of the crucial role of the infections in determining the clinical flares and the detection of a reduction of plasmatic levels of IgG, prophylaxis with antibiotics and substitutive treatment with human immunoglobulins were started. Thalidomide was then started at the dosage of 1 mg/kg/day together with oral steroids; however, few weeks later the skin manifestation worsened and the treatment was withdrawn. Due to the inefficacy of the previous treatment, dapsone was then started. The cutaneous flared that followed the beginning of this treatment were less severe with an erythematous rash not associated to pustular lesions and followed by fine desquamation. In order to achieve a complete control of the clinical manifestation, treatment with ustekinumab was started at the dosage of 0.75 mg/kg. In fact this anti-IL12 and 23 monoclonal antibody has been demonstrated to be effective in the control of severe pustular psoriasis. Moreover, the detection of the lymphocyte populations performed in the patient revealed a higher number of Th17 cells with a higher expression of Il17A and IL22 respect to the healthy donor. Following the beginning of treatment with ustekinumab the patient did not display any cutaneous flare and the steroidal treatment has been progressively reduced.

**Conclusion:** This clinical report enlighten the severity of DITRA syndrome. The treatments usually effective for both psoriasis and autoinflammatory diseases are frequently ineffective. The association of dapsone with ustekinumab is promising in our patient, together with the prevention of infections. However a longer period of observation is required to prove the efficacy of this approach, even after the discontinuation of steroidal treatment.

**Disclosure of Interest**

None Declared.

### P257 Nephrotic syndrome as the presenting feature in a child with NLRP3 mutation: more than what meets the eye?

#### Sagar Bhattad^1^, Amit Rawat^1^, Anju Gupta^1^, Deepti Suri^1^, Vignesh Pandiarajan^1^, Ritambhra Nada^2^, Kaara Tiewsoh^1^, Philip Hawkins^3^, Dorota Rowczenio^3^, Surjit Singh^1^

##### ^1^PEDIATRICS, PGIMER, CHANDIGARH,INDIA, CHANDIGARH, India; ^2^Histopathology, PGIMER, CHANDIGARH,INDIA, CHANDIGARH, India; ^3^Medicine, UCL, Royal Free Hospital, London, UK

###### **Presenting author:** Sagar Bhattad

**Introduction:** Long standing uncontrolled inflammation is known to result in amyloidosis in children with auto-inflammatory disease. Nephrotic syndrome as the presenting feature of amyloidosis in the absence of systemic inflammatory features is distinctly uncommon

**Objectives:** To report a child with renal amyloidosis due to *NLRP3* mutation who presented with steroid resistant nephrotic syndrome and paucity of systemic inflammatory features

**Methods:** A 10-year-old girl first born to a non-consanguineous couple was referred to our institute for the management of steroid resistant nephrotic syndrome. Her illness started at 6.5 years of age with edema and decreased urine output and the urinalysis revealed nephrotic range proteinuria. Biochemical investigations showed hypoalbuminemia and hypercholesterolemia. She was instituted on 2 mg/kg oral prednisolone therapy that was given for 6 weeks which was later tapered. She had relapses whenever her prednisolone doses were stepped down to alternate day regimen. Eight months later, she developed cortical venous thrombosis and was treated with injection heparin followed by oral warfarin.

Prior to presentation to our institute, she had received oral cyclophosphamide for 12 weeks followed by mycophenolate mofetil for 6 months. At 10 years of age, she was referred to our Institute. She was noted to have short stature, pallor, grade 3 pan-digital clubbing, and frontal bossing. Her height was 112.5 cm (< -3 WHO Z score) and weight was 17.6 kg (< -3). She also had mild ascites and pitting pedal edema. There was no lymphadenopathy and hepatosplenomegaly. Blood counts revealed white cell counts of 19.5x10^9^/L with neutrophilic predominance (62% polymorphs), anemia (78 g/L), and platelet counts of 567x10^9^/L. She also had an erythrocyte sedimentation rate of 55 mm at first hour and C-reactive protein levels of 19 mg/L (N < 6). Renal functions were deranged at admission (creatinine clearance- 26.4 ml/min). Ultrasound abdomen showed normal-sized kidneys and raised cortical echogenicity. Renal biopsy was performed that showed features of AA amyloidosis.

**Results:** Work up for chronic infections (like tuberculosis); cystic fibrosis and celiac disease was non-contributory. Serologies for human immunodeficiency virus (HIV) and hepatitis C virus were negative. Hepatitis B surface antigen and anti-nuclear antibodies were negative. Her previous medical records were reviewed and a note was made of persistently high platelet counts (580x10^9^/L to 821x10^9^/L) in the blood counts done ever since she had nephrosis. Magnetic resonance imaging of the brain was normal. Pure tone audiometry to assess hearing showed loss of air and bone conduction at 8000 Hz suggestive of mild high frequency sensorineural hearing loss. She continued to develop worsening renal functions and ascites in the follow-up. 3 months later, she succumbed to her illness due to uremia, fluid overload, and acute respiratory distress syndrome.

Genetic studies revealed a mutation in the exon 3 of *NLRP3* gene: **c.1055C > T;p.Ala352Val**. She was finally diagnosed to have renal amyloidosis due to *NLRP3* mutation and Cryopyrin associated periodic fever syndrome (CAPS)

**Conclusion:** Renal amyloidosis in children with CAPS can result in nephrotic syndrome. A high degree of suspicion is required to make a timely diagnosis

**Disclosure of Interest**

None Declared.

### P258 Renal AA amyloidosis – a piece in the diagnostic puzzle of cryopyrinopathy in 10 family members across 4 generations

#### Sarka Fingerhutova^1^, Jana Franova^2^, Leona Prochazkova^3^, Eva Hlavackova^4^, Pavla Dolezalova^1^

##### ^1^DEPARTMENT OF PAEDIATRICS AND ADOLESCENT MEDICINE, GENERAL UNIVERSITY HOSPITAL IN PRAGUE, PRAGUE, Czech Republic; ^2^Department of Paediatric Rheumatology, University Hospital Brno, Brno, Czech Republic; ^3^Department of Rheumatology, St Ann´s Hospital Brno, University Hospital, Brno, Czech Republic; ^4^Department of Allergology and Immunology, St Ann´s Hospital Brno, University Hospital, Brno, Czech Republic

###### **Presenting author:** Sarka Fingerhutova

**Introduction:** Cryopyrin-associated periodic syndromes (CAPS) are autoinflammatory disorders with autosomal dominant inheritance. Although Muckle-Wells Syndrome (MWS) stands in the middle of the CAPS severity spectrum, it is associated with the high risk of severe late complications that include hearing loss, vision impairment and AA amyloidosis affecting about 26% of individuals. Early diagnosis and appropriate treatment with interleukin-1 (IL-1) blockade may prevent their development.

**Objectives:** Analysis of a single paediatric patient trajectory leading to the revelation of multiple members of his family suffering with MWS, description of evolving disease phenotype in patients from different generations. To stress an importance of disease awareness as well as interdisciplinary collaboration in detecting familiar conditions affecting both children and adults.

**Methods:** Multiple-case study

**Results:** A 3-years old Caucasian boy was referred to the allergology clinic by his primary care paediatrician for recurrent flares of non-pruritic urticarial rash from about 6 months of age. There were no other symptoms of note. Detailed family history suggested presence of rash in other family members. Working together, paediatric and adult rheumatologist detected additional 9 individuals suffering with variable combinations of fever episodes with rash (all), episodic arthritis, eye inflammation, hearing loss, headache and AA amyloidosis. (Table 19, patient 3b). The boy's great grandmother died in 52 years of age from renal failure. Although renal biopsy was not performed at the time, presence of other symptoms as reported by her children made clinical diagnosis of MWS and amyloidosis very likely. Genetic analysis of exon 3 of NLRP3 gene in affected individuals revealed heterozygous mutation c.1322C > T causing change of amino acids p.Ala441Val and prompted referral to the autoinflammatory disease centre for biologic therapy.

**Conclusion:** In this family medical care was not primarily seeked by adults who have got used to their long-term and relatively mild symptoms. The diagnostic procedure was initiated by the paediatric referral. Detailed analysis of the symptoms suggests presence of the high risk of severe complications. Hearing loss, proteinuria and/or AA amyloidosis have only been noticed in individuals older than 50 years of age. Importantly, younger members below 30 years have not yet developed any of these symptoms which may now be prevented with long-term biologic therapy. This multi-case report illustrates importance of the high degree of suspicion as well as close collaboration between paediatric and adult specialists in order to establish timely diagnosis and initiate appropriate investigations and treatment.

**Disclosure of Interest**

S. Fingerhutova Speaker Bureau of: Novartis, J. Franova: None Declared, L. Prochazkova: None Declared, E. Hlavackova: None Declared, P. Dolezalova Grant / Research Support from: Abbvie, Roche, Medac, Novartis, Pfizer, Consultant for: Roche, Speaker Bureau of: Pfizer, Novartis, Medac.

**Table 19 (abstract P258).** Patient/Age(yrs)/Onset(yrs)/Fever/Rash/Eyes/Arthritis/Hearing loss/Headache/Proteinuria

1	3	0.3	-	+	-	-	-	-	-
2a	31	2	+	+	+	+	-	+	-
2b	31	3	+	+	+	+	-	-	?
2c	27	7	+	+	+	+	-	-	-
2d	24	?	+	+	+	+	-	-	-
2e	21	6	+	+	+	+	-	-	-
3a	51	2	+	+	+	+	+	+	+
3b	52	4	+	+	+	+	+	-	+
3c	43	?	-	+	+	-	?	?	?
4a	52	?	?	+	+	+	+	?	+

### P259 Insufficient systolic blood pressure recovery and heart rate recovery after graded exercise in children with familial Mediterranean fever

#### Havva Evrengül^1^, Selçuk Yüksel^2^, Mustafa Doğan^3^, Dolunay Gürses^3^, Harun Evrengül^4^

##### ^1^Pediatric Nephrology, Pamukkale University School of Medicine, DENIZLI, Turkey; ^2^Pediatric Rheumatology, Pamukkale University School of Medicine, DENIZLI, Turkey; ^3^Pediatric Cardiology, Pamukkale University School of Medicine, DENIZLI, Turkey; ^4^Cardiology, Pamukkale University School of Medicine, DENIZLI, Turkey

###### **Presenting author:** Selçuk Yüksel

**Introduction:** Familial Mediterranean Fever (FMF) is an autosomal recessive auto inflammatory disease. Long-term effects of sub clinical inflammation in FMF cause cardiac morbidity and mortality. Previous studies showed cardiac autonomic dysfunction in FMF patients. Heart rate recovery (HRR) and systolic blood pressure recovery (SBPR) can be used to evaluate cardiac autonomic function.

**Objectives:** The aim of this study is to evaluate the cardiac autonomic functions in pediatric FMF patients by using HRR and SBPR parameters.

**Methods:** A total of 50 FMF patients and 30 healthy children were included in the study. All patients were evaluated by echocardiography before study in order to eliminate congenital heart defects. All patients underwent a maximal graded exercise test. HRR and SBPR parameters were calculated.

**Results:** There was a significant decrease in HRR1 value in FMF patients (p = 0.03). In addition, SBPR1 and SPBR2 values were obtained higher in the FMF group compared to the control group (0.96 ± 0.12 vs. 0.88 ± 0.12 and 0.95 ± 0.09 vs. 0.91 ± 0.11); and the highness in SBPR1 value was statistically significant (p = 0.02). FMF presence had a negative correlation with HRR1 (r = −0.26, p = 0.03), but a positive correlation with SBPR1 (r = 0.29 p = 0.02). Moreover, there was a negative correlation of the M694V homozygous mutation with HRR1 and HRR2 values (r = -0.43, p = 0.004, r = -0.42, p = 0.005).

**Conclusion:** Cardiac involvement may consist in FMF patients without cardiovascular symptoms. Impaired SBPR and decreased HRR response may indicate increased cardiovascular risk in these patients despite normal exercise test.

**Disclosure of Interest**

None Declared.

### P260 Evaluation of undifferentiated periodic fever with PRINTO-Eurofever criteria at baseline and at follow-up

#### Silvia De Pauli^1^, Serena Pastore^2^, Anna Monica Bianco^3^, Giovanni Maria Severini^3^, Andrea Taddio^1,2^, Alberto Tommasini^2^

##### ^1^Pediatrics, UNIVERSITY OF TRIESTE, Trieste, Italy; ^2^Pediatrics, IRCCS Burlo Garofolo, Trieste, Italy; ^3^Genetics, IRCCS Burlo Garofolo, Trieste, Italy

###### **Presenting author:** Silvia De Pauli

**Introduction:** Subjects with recurrent fever that not fulfill criteria neither for PFAPA nor for well defined Hereditary Periodic Fever syndromes (HPF) are classified as Undifferentiated Periodic Fever (UPF).

**Objectives:** To test usability of PRINTO-Eurofever score (ARD 2015) in a setting of UPF. To study the correlation between PRINTO-Eurofever score and the response to different treatments in patients with UPF.

**Methods:** Retrospective study. We collected clinical and genetic data at diagnosis and follow-up from 26 subjects with UPF referred to our clinic from Jan 2006 to Apr 2016. We assessed Clinical classification (PRINTO-Eurofever) at baseline and follow-up. HPF-like score was correlated with response to treatments received anytime.

**Results:** We enrolled 26 subjects (8 males), mean duration of disease at last follow-up 4.3 yr. (range 1 – 17.8 yr.). Median disease onset was 9.5 mo. (2 – 111 mo.), median age at first visit 4.25 yr. (10 – 173 mo.).

At baseline, 13 subjects could be classified as HPF-like based on PRINTO-Eurofever score (3 FMF, 7 MKD, 1 CAPS, 4 TRAPS). At follow-up, 10 subjects could be classified as HPF-like (4 FMF, 6 MKD, 1 TRAPS): only 6 subjects confirmed their score, 1 case shifted from TRAPS to FMF, 5 became negative for any HPF and 3 subjects became positive for MKD. Overall, 5 patients healed during follow-up, two of whom after tonsillectomy.

20/26 subjects tested negative for genes commonly involved in HPF (MEFV, MVK, TNFRSF1A, NLRP3, NLRP12), whilst 6 had inclonclusive results (heteroxygous mutations: P369S-R408Q, V726A in MEFV and V377I in MVK, variation H304Y in NLRP12 of unknown significance, 2 subjects with homozygous R202Q variant). There is no clear correlation between genetic findings and final classification of patients.

We checked if the response to treatments received anytime correlated with clinical score at follow-up.

Tonsillectomy. 10/26 subjects underwent tonsillectomy before the first visit, 8 of whom without any response. Three of them were classified as HPF-like at follow-up.

Glucocorticoids. Poor response to glucocorticoids tended to be more common in UPF than in HPF-like (n.s).

Colchicine was tried in 12 subjects, with good response in less than half cases. No one of responders had a clinical score supportive of FMF.

**Conclusion:** UPF represent an heterogeneous group of disorders, as concern clinical features, response to treatments and prognosis. A fascinating hypothesis is that classification of UPF by analogy with well defined HPF could help in choosing appropriate treatments and in establishing a correct prognosis. We showed that PRINTO criteria seem not suitable for this purpose. First, PRINTO-scores are not stable uring follow-up of patients with UPF, since in our series most patients changed their score from baseline to last visit. Second, the PRINTO score at last follow-up doesn’t correlate with response to any previous treatment received, including tonsillectomy, glucocorticoids and colchicines, and thus seem not useful to classify patients for clinical purposes. Indeed, more than half patients showed poor response to glucocorticoids, tonsillectomy and colchicines and unexpectedly no patient who responded to colchicines had a clinical score supportive of FMF.

In conclusion, in agreement with recent results from other authors (Yang JA et al, 2016), we confirm the existence of a group of UPF that seem to be different from both PFAPA and HPF.

**Disclosure of Interest**

None Declared.

**Table 20 (abstract P260).** See text for description

	First visit	Follow – up	Recurrence after Tonsillectomy	Poor response to steroids	Response to colchicine
HPF	13	**10**	3	2/7	1/4
UPF	13	**16**	5	7/14	4/8
total	26	**26**	8	9/21	5/12
			n.s.	n.s.	n.s.

### P261 Phenotype of patients with low penetrant mutation Q705K in the NLRP3 gene in russian patients

#### Svetlana O. Salugina^1^, Evgeny Fedorov^1^, Elena Kamenets^2^, Ekaterina Zaharova^2^, Maria Kaleda^1^

##### ^1^Nasonova Research Institute of Rheumatology, Moscow, Russian Federation; ^2^FSBI "Research Centre for Medical Genetics", Moscow, Russian Federation

###### **Presenting author:** Svetlana O. Salugina

**Introduction:** Cryopyrin-associated periodic syndromes (CAPS) are a group of human autoinflammatory diseases (HAIDS) caused by heterozygous mutations in the *NLRP3* gene. The single base substitution NM_004895: c.2113C > A (Q705K) (identified in Infevers db as Q703K) is considered as mutation with low penetrance and presents in population at allele frequency of 4,1%. Its clinical significance is unknown, but heterozygous of this variant patients show CAPS-like phenotype and good response to IL-1 inhibitors treatment.

**Objectives:** To detect individuals with allele c.2113A of the *NLRP3* gene among Russian pts with HAIDS and to describe their phenotype.

**Methods:** 127 pts: 74 pts with suspected HAIDS and 53 pts with systemic juvenile arthritis (soJA) were examined for *NLRP3* mutations.

**Results:** In 9 pts (5 male and 4 female) aged between 3 and 57 heterozygous Q705K variant was found (7,1% of the patient setting). All these pts had features of autoinflammation: persistent fever (9), rash (7), acute-phase markers (8), serositis (3), arthritis (7), most of them polyarticular, nonpersistent, without destruction and functional disability. In 1 case recurrent aseptic meningoencephalitis was observed. In 3 of 9 pts in addition to Q705K pathogenic mutations T350M and V200M in 2pts were detected and diagnosis of CAPS was confirmed. Other 4 pts had clinical diagnosis of soJA and remaining 2 pts had undifferentiated HAIDS (UH). Most of pts (8) received glucocorticoids, 4 of them – methotrexate, 3 – biological drugs (tocilizumab – 2, canakinumab – 1) with good response.

**Conclusion:** All our pts with Q705K variant show phenotypes with autoinflammatory features similar to CAPS. It means that Q705K variant may take a part in autoinflammatory pathways including soJA. For such patients with inefficiency or inadequate response to standard therapy IL-1 inhibitors are considered as a reasonable alternative option.

**Disclosure of Interest**

None Declared.

### P262 Preliminary results of molecular genetic screening of mutations of the NLRP3, TNFRSF1A, MVK genes in patients with systemic juvenile arthritis and suspected autoinflammatory diseases

#### Svetlana O. Salugina^1^, Evgeny Fedorov^1^, Elena Kamenets^2^, Ekaterina Zaharova^2^, Maria Kaleda^1^

##### ^1^Nasonova Research Institute of Rheumatology, Moscow, Russian Federation; ^2^FSBI "Research Centre for Medical Genetics", Moscow, Russian Federation

###### **Presenting author:** Svetlana O. Salugina

**Introduction:**The hereditary autoinflammatory diseases (AIDS) are a group of disorders of nonspecific immunity characterized by recurrent fever and persistence systemic inflammation with multi-organ lesion. AIDS include monogenic and polygenic or multifactorial nosological entities. Now systemic juvenile arthritis (soJA) is considered as a AID too, though its genetic etiology is unclear.

**Objectives:** To define genetic defects in patients with suspected AIDS and soJA.

**Methods:** In the context of AIDS screening survey over the 2012 to 2015 period we examined 127 patients (62 male, 65 female) aged between 6 months and 60 years (M = 9 years). Duration of a disease was between 1,5 and 54 years (M = 3 years). We investigated the *NLRP3, TNFRSF1A* and *MVK* genes whose mutations cause the most common monogenic AIDS (CAPS, TRAPS and HIDS respectively). Among the patient setting were 74 pts with suspected AIDS (the 1st group) and 53 pts with diagnosis of soJA (the 2nd group). Selection criteria were persistent fever, maculopapular rash, arthralgia/arthritis and other manifestations of inflammatory, acute-phase markers without other causes. Coding sequence of the genes was analyzed by Sanger sequencing.

**Results:** Among 127 pts in 21 individuals genetic defects were found (16,5%): 13 are heterozygous for mutations in *NLRP3,* 6 - in *TNFRSF1A* (autosomal dominant mode of inheritance) and 2 are compound heterozygote for mutations in *MVK* (autosomal recessive mode of inheritance). Genetic diagnoses were made: CAPS-MWS (13), TRAPS-syndrome (6) and HIDS (2) accordingly. Mutations detected in 17 cases in the 1st group (22,9%): 12 *NLRP3* defects (V200M, T350M, T438I, Y443H), 4 *TNFRSF1A* defects (H51Y, C59R, C99R) and 1 *MVK* (V377I/R388T); and in 4 cases in the 2nd group (9,4%): 1 (G455T), 2 (P52R, R121Q), 1(N205D/G376R) respectively. Also low penetrant mutation of the *NLRP3* gene Q705K was found in 5 of HAIDS pts and in 4 of soJA pts.

**Conclusion:** Missense and nonsense mutations of the *NLRP3* gene is the most frequent cause of the disease in the analyzed pts (10,2%). Also single base changes of other genes were revealed. Our findings demonstrate reasonability of genetic analysis for pts with autoinflammatory phenotype including soJA pts, especially for those who show deficient or inadequate response to standard therapy for uncovering monogenic AIDS and target treatment.

**Disclosure of Interest**

None Declared.

### P263 Juvenile idiopathic arthritis or autoinflammatory syndromes? High frequency of autoinflammatory syndromes in Russian JIA patients

#### Tatiana Sleptsova^1^, Ekaterina Alexeeva^1,2^, Kirill Savostyanov^1^, Alexander Pushkov^1^, Tatyana Bzarova^1,2^, Saniya Valieva^1^, Rina Denisova^1^, Kseniya Isayeva^1^, Evgeniya Chistyakova^1,2^, Olga Lomakina^1^, Margarita Soloshenko^1^, Elena Kaschenko^1^

##### ^1^Scientific Centre of Children’s Health, Moscow, Russian Federation; ^2^I.M. Sechenov First Moscow State Medical University, Moscow, Russian Federation

###### **Presenting author:** Tatiana Sleptsova

**Introduction:** The main clinical manifestations of monogenic autoinflammatory syndromes and polygenic juvenile idiopathic arthritis (JIA) are similar and presented with fever, myalgia, migratory erythematous rash, arthritis or arthralgia, eye manifestations, and abdominal pain. The right diagnosis is reached using gene analysis and prognosis depends on correct personified therapy.

**Objectives:** to determine the frequency of autoinflammatory syndromes in Russian patients with JIA.

**Methods:** 100 pts (42 boys, 58 girls) at the age from 1 to 17y. (6.9 (3.9;9.4) followed with a diagnosis of JIA (olygoarthritis - 31 pts, polyarthritis - 26, systemic JIA - 38, juvenile ankylosing spondyloitis - 4, juvenile rheumatoid arthritis (seropositive) - 1 pts) were selected according to the clinical manifestations. The median age of disease onset was 2.7 (1.2;6.0)y., disease duration – 4.0 (2.6;5.2)y. The commonest features were fever (70%), arthritis/arthragia (100%), rash (69%), hepato-/splenomegaly (67%), lymphadenopathy (65%), headache (55%), abdominal pain (51%), eye manifestations (23%). Pts’ DNA was sequenced in all coding exons and intronic flanks of the *MVK, MEFV, TNFRSF1A, NLRP3, NLRP12, CECR1, IL1RN, TMEM173, NLRC4, LACC1, NOD2, PSTPIP, LPIN2, PSMB8, TRNT1* genes.

**Results:** In 25/100 pts genetic autoinflammatory syndrome was established. TRAPS was diagnosed in 6 pts (we found mutations c.362G > A (p.R92Q) located in exon 4 in TNFRSF1A); CAPS (1-pathogenic mutation, 7 - NLRP3 gene polymorphism Q705K) - in 8 pts; PASH (*c.22G > A (p.G8R)* in gene *PSMB8*)– in 5, Familial Mediterranean fever - in 3 pts, syndrome Majeed (*c.2264 C > T* in *NOD2*) and SIFD (c.809C > G)– in 1 pts, respectively. NLRC4-associated autoinflammatory syndrome with enterocolitis was established in 1 case. The pt had phenotype with periodic fever, rash, enterocolitis, debuted in the first days of life and mutation *c.928C > T (p.R310X*) in *NLRC4 gene. T*he mutation wasn`t previously described in the databases for mutations*.* Therapy of canakinumab, started at the age of 2 years was effective: no symptoms of the disease for 14 months of treatment.

**Conclusion:** Our results suggests for a relatively frequent incidence of autoinflammatory syndromes in Russian JIA patients: about 25% of pts have genetically confirmed periodic fever syndromes. It is important to researches on this issue in depth.

**Disclosure of Interest**

None Declared.

### P264 Severe neurological involvement of mevalonate kinase deficiency mimicking Leigh encephalopathy

#### Utako Kaneko, Chihaya Imai, Akihiko Saitoh

##### Pediatrics, Niigata University, Niigata, Japan

###### **Presenting author:** Utako Kaneko

**Introduction:** Mevalonate kinase deficiency (MKD) is a multisystemic inflammatory disorder caused by a mutation of *MVK* gene that severely reduces mevalonate kinase activity. Mevalonic aciduria (MA) is severe phenotype of MKD characterized by serious neurologic abnormalities as well as recurrent fever with poor prognosis. The pathogenesis of neurologic impairment of MA is not yet clear. One hypothesis is that neurodegeneration is linked to intrinsic apoptosis pathway triggered by mitochondrial damage and to pyroptosis. ^[1]^

**Objectives:** To investigate the relationship between neurological aspects of MA and mitochondrial dysfunction.

**Methods:** We present a case of MA with severe neurological involvement, mimicking Leigh encephalopathy, which is neurodegenerative disorder associated with dysfunction in mitochondrial energy metabolism.

**Results:** A 1-month-old Japanese male of non-consanguineous background was referred to our hospital due to fever of unknown origin. He developed interstitial pneumonia, elevated liver enzymes, and hypertrophy of myocardium with chronic inflammation. Furthermore, he showed severe developemental delay and failure to thrive.

Laboratory data showed elevation of blood lactate and pyruvate concentration with mild lactic acidemia. The lactate to pyruvate ratio of blood and cerebrospinal fluid increased suggesting mitochondrial dysfunction. Magnetic resonance imaging of brain showed symmetrical T2 hyperintensity of basal ganglia, severe brain atrophy and thinning of corpus callosum, which resembled to Leigh encephalopathy. However, extremely increased urinary excretion of mevalonic acid was revealed, and molecular analysis showed homozygous mutation c.227-1G > A of the *MVK* gene. He was diagnosed as having mevalonate kinase deficiency at the age of 14 months. He was treated with oral corticosteroid, resulting in partial improvement of chronic inflammation, but no change was observed for his severe psychomotor retardation. Our plan is to perform allogenic bone marrow transplantation from his father, who is HLA one-antigen-mismatched and heterozygous carrier of the mutant gene.

**Conclusion:** It was suggested that neuropathology of severe phenotype of MA might be related to secondary mitochondrial dysfunction due to deregulation of mevalonate pathway.

**Reference**

[1] Tricarico PM, Marcuzzi A, et al. Mevalonate kinase deficiency and neuroinflammation: balance between apoptosis and pyroptosis. Int J Mol Sci. 2013;14.

**Disclosure of Interest**

None Declared.

### P265 Severe and refractory childhood-onset polyarteritis nodosa associated with CECR1 mutation

#### Vitor A. Teixeira^1^, Filipa O. Ramos^1,2,3^, Manuela Costa^1^

##### ^1^Serviço de Reumatologia e Doenças Ósseas Metabólicas, Lisbon, Portugal; ^2^Unidade de Reumatologia Pediátrica, Serviço de Reumatologia e Departamento de Pediatria, Hospital de Santa Maria, Centro Hospitalar Lisboa Norte, Lisbon, Portugal; ^3^Instituto de Medicina Molecular, Faculdade de Medicina de Lisboa, Lisbon, Portugal

###### **Presenting author:** Vitor A. Teixeira

**Introduction:** The mechanisms of Polyarteritis Nodosa (PAN) are probably heterogeneous since it has been described in association with cancers and multiple infections. Recently loss-of-function mutations in CECR1 have also been associated with a spectrum of vascular and inflammatory phenotypes, including childhood-onset PAN.

**Objectives:** To report a case of childhood-onset PAN associated with autosomal recessive mutations in CECR1 gene.

**Methods:** This report focus on a single case. Data were collected from the patient clinical registry and we discuss it based on the scientific literature.

**Results:** A female patient, currently 31 years old (y/o), presented at 5 years of age with long-lasting fever, asymmetric polyarthritis and livedo reticularis, leucocytosis, normochromic normocytic anaemia and elevation of ESR (117 mm/hour). Angiographic studies revealed renal and hepatic microaneurysms. Subcutaneous nodules were present and biopsy was compatible with PAN. Infectious causes of PAN were excluded. Throughout her life, she suffered many disease complications such as paresia of the sixth cranial pair (10y/o), ischemic stroke of the left anterior corona radiata and right lateral thalamus, cauda equina syndrome (26y/o), multiple mononeuropathy (29y/o) and two digital amputations due to digital necrosis (27 and 30y/o), all of these occurring despite several treatment strategies, which included long term corticosteroids, aspirin, oral anticoagulants, azathioprine, methotrexate, several cycles of intravenous cyclophosphamide followed by oral cyclophosphamide, intravenous immunoglobulin, iloprost and oral vasodilators. These multiple complications of the disease rendered impossible to tapper corticosteroids and the patient had at a very early age multiple treatment complications including growth restriction, cushingoid habitus, secondary osteoporosis with multiple fractures, metabolic syndrome, recurrent infections and precancerous conditions like Barrett esophagus and cervical intraepithelial dysplasia related to human papillomavirus.

Two autosomal recessive heterozygous mutations were found in our patient localized to gene CECR1: p.Gly47Arg and p.Tyr453Cys, both pathogenic. After many years of frustrating disease control, infliximab (5 mg/kg) was initiated at age 30, moved by successful recent reports. Following the first infusion, there was a mild decreased in ESR from 47 to 27 mm/h, but a major rise of haemoglobin levels from basal 8-10 to 13-15 g/dL; intravenous iron supplementation was discontinued. There was resolution of the patient’s fatigue, an important limiting symptom of her quality of life and she hasn’t had any relapse in a one-year follow-up.

Discussion: This is the first described Portuguese case of childhood onset PAN associated with compound heterozygous mutations in the CECR1 gene, which are associated with lower levels or loss of activity of adenosine deaminase 2, a growth factor for endothelial and leukocyte development and differentiation. These mutations have recently been implicated in the pathogenesis of a few cases of PAN presenting a high variability in the age of onset and clinical presentation. These patients apparently respond well to infliximab, by inhibiting the TNF-alfa, a pro-inflammatory cytokine produced primarily by cells of the macrophage-monocyte lineage and thus thought to participate in the vascular inflammation and endothelial cell death.

**Conclusion:** The identification of mutations in CECR1 should be sought in severe and refractory vasculopathies that overlap PAN since it argues in favour of early-onset of infliximab, and could allow a better management of corticosteroids and other immunomodulating drugs, reducing comorbidities.

**Disclosure of Interest**

None Declared.

### P266 Controlled discontinuation of colchicine therapy in familial Mediterranean fever patients with single MEFV mutation

#### Yonatan Butbul Aviel^1^, Shafe Fahoum^2^, Riva Brik^3^

##### ^1^Department of Pediatrics B, Pediatric Rheumatology Service, Ruth Children's Hospital, Rambam Medical Center, Haifa, Israel; ^2^Department of Pediatrics B, Pediatric Rheumatology Service, Ruth Children's Hospital, Rambam Medical Center, Haifa, Israel, appaport Faculty of Medicine, Technion-lsrael Institute of Technology, Haifa, Israel; ^3^Department of Pediatrics B, Pediatric Rheumatology Service, Ruth Children's Hospital, Rambam Medical Center, Haifa, Israel, Rappaport Faculty of Medicine, Technion-lsrael Institute of Technology, Haifa, Israel

###### **Presenting author:** Yonatan Butbul Aviel

**Introduction:** Familial Mediterranean fever (FMF) traditionally has been considered an autosomal recessive disease; however, the diagnosis remains predominantly clinical, since mutations cannot always be identified on both alleles.

**Objectives:** to evaluate cessation of colchicine therapy in selected group of patients with FMF who possess only 1 or none demonstrable MEFV mutation.

**Methods:** We performed a prospective controlled study evaluated cessation of colchicines therapy in patients that were previously diagnosed and treatd for FMF based on clinical features and did not carry any common MEFV mutation or were heterozygote for one of the mutations.

Patients were included in the study if they were between the age of 2-18 years and were treated with colchicine, had a normal level of serum amyloid A (SAA)and had at least 6 months free of FMF attacks.

SAA levels were evaluated before colchicine cessation and at 3 and 6 months following cessation. Colchicine therapy was resumed in case of FMF attacks reappeared during this period.

**Results:** twelve patients ages 10.7 ± 4 years enrolled in the study. Prior to entering the study patients were treated with colchicine for an average of 36.3 month (7-144 months). The average time with no FMF attacks before enrolment into the study was 12.8 ± 8.6 months and the average follow up after stopping colchicine therapy was 15.3 ± 5.7 months.Five patients were heterozygote for the M694Vmutation.

Five patients (41.6%) had an FMF attack during follow up and needed to renew colchicine therapy, the average time to renew colchicine therapy was 5.3 months (range 1.5-11.4 months) 3 of them (60%) carried the M694V mutation.

There were no differences between the groups of patients that did not relapse and the groups that needed to renew therapy regarding age (10.7 ± 1.6 vs 10.6 ± 6.3 p- 0.97) or levels of SAA at time of enrolment (4 ± 3.6 vs 3.3 ± 2.4 p-0.7). Length of colchicine therapy prior to enrolment showed tendency that didn't reach significance towards longer time in the patients needed to resume therapy (22.3 ± 12.6 vs 53 ± 51 months p-0.18).

**Conclusion:** Cessation of colchicine therapy in selected group of patients who are not homozygous for the common MEFV mutation should be considered. Monitoring SAA levels every 3 months could not predict FMF attacks following cessation of colchicine therapy.

**Disclosure of Interest**

None Declared

### P267 Sacroiliitis in children with familial Mediterranean fever

#### Zeynep Birsin Özçakar^1^, Nilgün Çakar^1^, Nermin Uncu^2^, Banu Acar Celikel^3^, Fatos Yalcinkaya^1^

##### ^1^Pediatric Rheumatology, Ankara University, Ankara, Turkey; ^2^Pediatric Rheumatology, Child Health, Hematology and Oncology Education and Research Hospital, Ankara, Turkey; ^3^Pediatric Rheumatology, Kırıkkale University, Kırıkkale, Turkey

###### **Presenting author:** Zeynep Birsin Özçakar

**Introduction:** Familial Mediterranean fever (FMF) is an autosomal recessive disease, characterised by recurrent, self limited attacks of fever with serositis. Various diseases were reported to be associated with FMF or carriers of *MEFV* mutations.

**Objectives:** The aim of this study was to investigate the frequency and characteristics of sacroiliitis in children with FMF.

**Methods:** Files of FMF patients who had been seen in two reference hospitals in Ankara, in the last two years, were retrospectively evaluated. Patients with FMF and concomitant sacroiliitis were included to the study. All patients had magnetic resonance imaging evidence of sacroiliitis.

**Results:** Among 600 FMF patients 17 (11 females, 6males; mean age: 13,32 ± 4,24 years) (2.8%) of them were found to have sacroiliitis. Mean age at FMF diagnosis and sacroiliitis diagnosis were 106,76 ± 43,17 and 130,76 ± 39,12 months, respectively. FMF diagnosis was done concurrently or afterwards to sacroiliitis in only 6 (35%) patients. Ten patients had isolated sacroiliitis and 7 had associated diseases (5 enthesitis related arthritis, 1 psoriatic arthritis and 1 ulcerative colitis). Eighty-eight percent of the patients carried at least one M694V mutation. Classical FMF attacks were present in 14 patients; the remaining 3 patients had atypical symptoms but had 2 *MEFV* mutations.

**Conclusion:** Sacroiliitis can be seen in patients with FMF during childhood. M694V mutation seems to be a susceptibility factor for its development.

**Disclosure of Interest**

None Declared

## Poster Session: Disease outcome

### P268 Minimal clinically important difference of the juvenile arthritis disease activity score

#### Benedetta Schiappapietra^1,2^, Sergio Davi'^2^, Federica Mongini^2^, Luisa Giannone^2^, Cecilia Bava^2^, Maria Giannina Alpigiani^2^, Alberto Martini^2^, Angelo Ravelli^1,2^, Alessandro Consolaro^1,2^

##### ^1^UNIVERSITA' DI GENOVA, GENOVA, Italy; ^2^Pediatria II, IRCCS G.GASLINI, GENOVA, Italy

###### **Presenting author:** Benedetta Schiappapietra

**Introduction:** An established approach to the measurement of disease activity in juvenile idiopathic arthritis (JIA) is based on the Juvenile Arthritis Disease Activity Score (JADAS). The JADAS consists of 4 variables: physician global rating of overall disease activity; parent/child ratings of wellbeing; count of active joints, assessed in 71 (JADAS71), 27 (JADAS27) or 10 (JADAS10) joints; and erythrocyte sedimentation rate (ESR). The instrument score is yielded by making the arithmetical sum of the scores of the 4 items. The JADAS has been shown to be feasible and to have good metrologic properties. Moreover, a 3-variable version of the JADAS, which does not include the acute phase reactant, was found to correlate closely with the original tool (clinical JADAS, cJADAS).

**Objectives:** To define the minimal clinically important differences (MCID) of all JADAS versions for worsening and improvement using both parent and physician perspectives.

**Methods:** Changes in the JADAS scores in consecutive visits were calculated in a dataset of 1,874 visits of JIA patients seen at the study Unit. Both parent and attending physician were asked to rate in a 5-point Likert scale if the patient was much worsened, slightly worsened, stable, slightly improved, much improved from previous visit. Those visits in which patient was subjectively rated by parents or by the physician as slightly worsened or slightly improved were retained. Subsequent visits with a time interval of more than 6 months were not retained; each patient could contribute for no more than 1 visit for improvement and 1 visit for worsening for each assessor. MCID were defined as the median changes of the JADAS scores of individual patients who had a minimal important improvement or worsening between visits.

**Results:** Table shows calculated MCID for all JADAS versions according to parents and physician.

**Conclusion:** MCID for all JADAS and cJADAS versions were defined according to parent and physician perspective. Differently from the results of previous study, the clinically important change of the score was smaller for improvement than for worsening for both parents and physicians. Parents required a minor change in the score than physician to define both improvement and worsening.

**Disclosure of Interest**

None Declared.

**Table 21 (abstract P268).** See text for description

		JADAS10	JADAS27	JADAS71	cJADAS10	cJADAS27	cJADAS71
Improvement	Parent	-2.7	-2.5	-2.7	-2.5	-2	-2.5
	Physician	-4	-3.5	-4.5	-3.5	-3.5	-4
Worsening	Parent	3.6	3	3.6	3.3	3	3.3
	Physician	4.2	4	4.4	3.5	3.5	3.5

### P269 Influence of FokI vitamin D receptor (VDR) and TNFα-308 tumor necrosis factor gene polymorphism on long term disease outcome in juvenile idiopathic arthritis (JIA)

#### Dragana S. Lazarevic^1^, Jelena Vojinovic^1, 2^, Gordana Susic^3^, Jelena Basic^4^

##### ^1^Pediatric Rheumatology, CLINIC OF PEDIATRICS, CLINICAL CENTER NIS, Nis, Serbia; ^2^Faculty of Medicine, University of Nis, Nis, Serbia; ^3^Institute of Rheumatology, Belgrade, Serbia; ^4^Institute for Biochemistry, Faculty of Medicine, University of Niš, Nis, Serbia

###### **Presenting author:** Dragana S. Lazarevic

**Introduction:** Genetic contribution of TNFα-308 promoter and FokI Vitamin D receptor gene polymorphism on response to biological treatment is not yet well established.

**Objectives:** To investigate possible influence of tumor necrosis factor alpha (TNFα-308) and FokI Vitamin D receptor (VDR) gene polymorphism on long term disease outcome in JIA patients treated with biologics.

**Methods:** We have retrospectively analysed data from our registry of JIA patients treated with biologics in whome 6 years follow-up data could be obtained and genomic DNA extracted to test TNFά–308 promoter and FokI VDR polymorphism. Disease activity was evaluated by ACR Pedi core set criteria for inactive disease.

**Results:** We have evaluated 78 JIA patients in whom there was not significant distribution difference of TNFα-308and FokI Vitamin D receptor (VDR) gene polymorphism among different JIA subtypes. Patients with the -308 GG (p = 0,004) and GA (p = 0,026) genotype achieved clinical response significantly more frequently than those with the -308 AA genotype after 36 month of follow up period. Patients with the FF(p = 0,006) and Ff (p = 0,036) genotypes had a reduction of disease activity and more frequently reached clinical response to biologics with respect to the ff genotypeat the end of the observational period.

There was no influence of distribution of the -308 TNF-α on achieving remission, but there was different distribution of FokI polymorphism on possibility to achieve remission at the end of the observational period. Patients resistant to biologics had significantly more frequent ff genotype, while those achieved remission had significantly more frequent Ff genotype (Χ^2^ = 6,52, p = 0,038). In univariate unconditional logistic regression analysis positive clinical predictor of achieving remission after 24 months under biologics was low dose of steroids (OR 0,749, p = 0,025), while genetic determinats were insuffitient predictors of disease outcome.

**Conclusion:** JIA patients carrying the TNFα-308 AA genotype and those with VDR ff genotype are associated with a poorer response to biological treatment. The present study is the first to demonstrate that patients with Ff genotype and low dose of steroids have better chance to achieve remission.

**Disclosure of Interest**

None Declared.

### P270 Disease activity and damage in juvenile idiopathic arthritis: comparison between “methotrexate” and “biologic” eras

#### Gabriella Giancane^1^, Valentina Muratore^1^, Valentina Marzetti^1^, Neus Quilis^1^, Belen Serrano Benavente^1^, Alessandra Alongi^1^, Adele Civino^2^, Lorenzo Quartulli^2^, Alessandro Consolaro^1^, Alberto Martini^1^, Angelo Ravelli^1^

##### ^1^Pediatria II, Reumatologia, Istituto Giannina Gaslini, Genoa, Italy; ^2^UOC Pediatria, AO "Card.G.Panico", Tricase, Italy

###### **Presenting author:** Gabriella Giancane

**Introduction:** The introduction of biologic agents at the beginning of the 2000s has represented a major advance in the management of juvenile idiopathic arthritis (JIA). These medications have been shown to be effective in a sizeable proportion of patients refractory or intolerant to methotrexate. However, in order to document the impact of the recent therapeutic progress on the prognosis of JIA there is the need to compare the long-term outcomes achieved with previous therapies with those obtained with the newer medications.

**Objectives:** To compare the level of disease activity and the amount of articular and extra-articular damage between patients treated in the “methotrexate” and “biologic” eras.

**Methods:** Data for the “methotrexate” era were extracted from a previous study on disease outcome in 310 patients with disease onset before 2002 and disease duration of ≥5 years (Solari et al. A&R 2008;59:1571-9). Data for the “biologic” era were obtained with the present study by examining all consecutive patients with JIA who had disease onset between January 2002 and June 2011 and a disease duration of ≥5 years. Outcome assessments included joint counts, physician global assessment of overall disease activity on a visual analog scale, Juvenile Arthritis Disease Activity Score-10 (JADAS10), Juvenile Arthritis Multidimensional Assessment Report (*JAMAR*) completed by a parent, and acute phase reactants. The amount of articular and extra-articular damage was assessed through the Juvenile Arthritis Damage Index (JADI).

**Results:** Demographic and clinical features of patients seen in the two study periods were overall comparable. As compared to the older sample, patients treated more recently had received more frequently methotrexate (84.2 vs 64.5%) and biologic medications (61.8% vs 11.3%), and had undergone more commonly intra-articular corticosteroid injections (98% vs 79%). The comparison of disease activity and damage between the two cohorts is presented in the table. All items of articular and extra-articular JADI were decreased in the recent sample, with the exception of temporo-mandibular damage and leg-length discrepancy, whose frequency was comparable in the two datasets.

**Conclusion:** Compared with patients treated in the “methotrexate” era, those treated in the “biologic” era have a higher frequency of inactive disease and less articular and extra-articular damage. These findings highlight the improvement in disease outlook achieved with the newer therapeutic modalities.

**Disclosure of Interest**

None Declared.

**Table 22 (abstract P270).** Comparison of disease activity and damage between the “methotrexate” and “biologic” eras

	“Methotrexate” era^£^ (n = 310) N. (%)	“Biologic” era (n =152) N. (%)
Patients with no active joints	92 (29.8)	96 (63.2)
Patients with no restricted joints	126 (40.8)	101 (66.5)
Patients with physician global assessment = 0	89 (29.6)	94 (61.8)
Patients with inactive disease on JADAS10	64 (20.6)	40 (26.3)
Patients with inactive disease on cJADAS10	73 (23.5)	62 (40.8)
Functional ability score = 0	155 (51.3) ^$^	83 (54.6) ^§^
Patients with JADI-articular = 0	204 (65.8)	124 (81.6)
Patients with JADI-extraarticular = 0	229 (73.9)	128 (84.2)

^£^Data from Solari et al A&R 2008;59:1571-9; ^$^assessed with CHAQ; ^§^assessed with JAFS; JADAS-10: Juvenile Arthritis Disease Activity Score-10; *JADI* Juvenile Arthritis Damage Index

### P271 Development and initial validation of the parent and child versions of the JADAS

#### Giedre Januskeviciute^1^, Pieter van Dijkhuizen^2^, Valentina Muratore^2^, Gabriella Giancane^2^, Benedetta Schiappapietra^2^, Alberto Martini^2^, Angelo Ravelli^2^, Alessandro Consolaro^2^

##### ^1^Klaipeda Children's Hospital, Klaipeda, Lithuania, ^2^Istituto Giannina Gaslini, Genoa, Italy

###### **Presenting author:** Giedre Januskeviciute

**Introduction:** Increasing attention is being paid to parent-/child-reported outcomes in juvenile idiopathic arthritis (JIA). Incorporation of these measures in patient assessment is deemed important as they reflect the parent's and children’s perception of the disease course and effectiveness of therapeutic interventions.

**Objectives:** To develop and to validate a composite parent-centered and child-centered versions of the JADAS, named parJADAS and chiJADAS, respectively.

**Methods:** The parJADAS and chiJADAS include 4 measures: 1) parent/child assessment of disease activity; 2) assessment of pain intensity; 3) self/proxy assessment of joint disease; 4) assessment of morning stiffness (MS). Disease activity and pain are assessed on a 21-numbered circle VAS (0 = best and 10 = worst). The active joint count is based on the count of any swollen or painful joint, irrespective of its type, up to a maximum of 10 joints. MS duration is assessed on a Likert scale, ranging from no MS (0 points) to > 2 hours of MS (10 points).

Validation was conducted on a dataset of 602 children with JIA who underwent 1749 visits at study unit. To account for repeated measurements in a single patient, construct validity was assessed by calculating between-subject and within-subject correlations of parJADAS and chiJADAS with JADAS10, cJADAS10, physician global assessment of disease activity, the number of active joints, parent/child rating of overall well-being and erythrocyte sedimentation rate (ESR). Discriminant ability was evaluated by comparing parJADAS and chiJADAS levels between patients with active or inactive disease according to current criteria, and between patients who were satisfied or not satisfied with disease outcome. Sensitivity to change was tested using standardized response mean (SRM) in 2 subsequent visits performed no more than 6 months apart. Internal consistency was assessed by means of Cronbach‘s alpha coefficient and inter-rater reliability was assessed using the intraclass correlation coefficient (ICC).

**Results:** Between-subject correlations of parJADAS and chiJADAS were high (>0.70) with JADAS10, cJADAS10, parent/child rating of overall well-being, and moderate (0.40-0.70) with the other measurements. Moreover, in the same subject, changes over time of the parJADAS and chiJADAS corresponded to changes in disease activity, as indicated by high within-subject correlations with JADAS10, cJADAS10, physician global assessment of disease activity, parent/child rating of overall well-being, and active joint count. Both parJADAS and chiJADAS discriminated well between inactive and active disease and between satisfied and not satisfied patients (p < 0.001). The responsiveness to clinical change of parJADAS was good (SRM = 0.84). The internal consistency was satisfactory, with Cronbach‘s alpha >0.80 for both parJADAS and chiJADAS. The inter-rater reliability between the parJADAS and the chiJADAS measured at the same visit was high, with ICC 0.92 (95% CI 0.90-0.93).

**Conclusion:** The parJADAS and chiJADAS were found to be valid and reliable for assessment of disease activity in JIA and may, therefore, be suitable for use in clinical practice, observational studies, and therapeutic trials. Both scores may potentially surrogate physician assessments when these are not available.

**Disclosure of Interest**

None Declared.

### P272 Adult outcomes in a large cohort of childhood-onset SLE patients: fertility and pregnancies – the CHILL-NL study

#### N. Groot^1^, W. van Dijk^1^, I. E. M. Bultink^1^, M. Bijl^1^, R. J. E. M. Dolhain^1^, Y. K. O. Teng^1^, E. Zirkzee^1^, K. de Leeuw^1^, R. Fritsch-Stork^1^, S. S. M. Kamphuis^1^

##### ^1^Sophia Children's Hospital - Erasmus University Medical Centre, Rotterdam, Netherlands

###### **Presenting author:** N. Groot

**Introduction:** Systemic lupus erythematosus (SLE) is a rare, lifelong autoimmune disease. Childhood-onset SLE (cSLE) often has its onset during puberty, and is thought to be more severe than SLE. Information regarding long term outcomes like fertility and pregnancies is scarce for this population.

**Objectives:** To investigate effects of cSLE on family planning, fertility and pregnancies in a large cohort of 101 female adults with cSLE.

**Methods:** Adults with cSLE and SLE were seen for a single study visit. Information regarding family planning, fertility and pregnancies were assessed by structured questionnaires and checked with medical records.

**Results:** We studied a cohort of 101 female cSLE patients with median age at study visit of 33 years and median disease duration of 20 years.

26% of patients felt that effects of cSLE limited them in their sexuality. Two third of the patients found the disease to be a restrictive factor in wanting children. They feared complications during pregnancy (51%) and during labour (30%), and problems with raising children (34%). 29% of patients feared their children would be diagnosed with SLE.

A total of 85 pregnancies were reported in 36 cSLE patients (median 2 pregnancies per female), of whom 22% had a history of cyclophosphamide use. Remarkably, time to pregnancy was not delayed, as it was 12 months or less in all but three patients. The 85 pregnancies resulted in 63 live births (76%), 13 miscarriages (15%), and 5 induced abortions (6%). The miscarriage rate in this group was comparable to that of healthy Dutch women.

An impressive 37% of the 67 pregnancies with a duration of at least 20 weeks had a complicated course. Four pregnancies (6%) resulted in stillbirth, where only 0,9% of pregnancies have this outcome in healthy Dutch women. Other complications were pregnancy induced hypertension, pre-eclampsia/HELLP, preterm birth (median gestational age of 31 weeks) and placental abruption. The frequency of pregnancy complications was higher in cSLE than in SLE and the Dutch female population.

**Conclusion:** Family planning is affected in a substantial percentage of females with cSLE. Multiple issues complicated their wish to have children. In this cohort, fertility did not seem to be impaired as no delay was found in the time to pregnancy. In contrast, the frequency of pregnancy complications including stillbirth was remarkably high in cSLE patients.

**Disclosure of Interest**

None Declared.

**Table 23 (abstract P272).** Patient characteristics and pregnancy characteristics

	cSLE: n = 101	SLE n = 37	p*
Ethnicity			
White	71%	68%	
Non White (%)	29%	32%	
Age at study visit (*median (range))*	33 (18 – 65)*	38 (25 – 76)*	0.003
Age at diagnosis in yrs (*median (range))*	14 (5 – 17)*	26 (18 – 63)*	0.000
Disease duration in yrs (*median (range))*	20 (1 – 55)	12 (1 – 34)	0.000
Current SLEDAI-2 K score (*median (range))*	4 (0 – 14)	5 (0 – 10)	
SDI-score (*median (range))*	1 (0 – 8)	1 (0 – 7)	
Patients with SLICC-DI ≥ 1	60% (61/40)	57% (21/37)	
cSLE restrictive in pregnancy wish	66% (63/96)	69% (25/36)	
Patients ever pregnant (after diagnosis of SLE)	36% (36/101)	41% (9/22)	
With history of cyclophosphamide use	22% (8/36)	22% (2/9)	
Time to first pregnancy, months *(median(range))*	2 (0 – 84)	1 (0 – 12)	n.s.
Age at first pregnancy, yrs *(median(range))*	27 (18 – 36)	30 (22 – 32)	n.s.
Total number of pregnancies	85	19	
Miscarriages (<20 weeks)	15% (13/85)	21% (4/19)	
Induced Abortion	6% (5/85)	0	
Any pregnancy complications > 20 weeks	37% (25/67)	27% (4/15)	n.s.
Stillbirth	16% (4/25)	0	
Pregnancy induced hypertension	12% (3/25)	0	
Pre-eclampsia/HELLP	24% (6/25)	0	
Preterm birth *(AD < 37 weeks)*	20% (5/25)	25% (1/4)	
Median gestational age *(range)*	31 wk,28-35	32 wk	
Placental abruption	8% (2/25)	25% (1/4)	
Other	20% (5/25)	50% (2/4)	

if no p-values are given, differences between cSLE and SLE were not statistically significant

### P273 Adult outcomes in a large cohort of childhood-onset SLE patients: education and work participation – the CHILL-NL study

#### Noortje Groot, A. Kardolus, I. E. M. Bultink, M. Bijl, R. J. E. M. Dolhain, Y. K. O. Teng, E. Zirkzee, K. de Leeuw, R. Fritsch-Stork, S. S. M. Kamphuis

##### Sophia Children's Hospital - Erasmus University Medical Centre, Rotterdam, Netherlands

###### **Presenting author:** Noortje Groot

**Introduction:** Systemic lupus erythematosus (SLE) is a rare autoimmune disease which can affect any organ system. Childhood-onset SLE (cSLE) is thought to be more severe than SLE, with more major organ involvement and higher disease activity. Little is known about the effects of cSLE on education and work.

**Objectives:** To investigate effects of cSLE on education, career and work participation in a large cohort (n = 106) of adults with cSLE.

**Methods:** Patients were seen by the CHILL-NL (CHILdhood Lupus in the NetherLands) study team for a study visit containing a structured history and physical examination. Education and work status were assessed by validated questionnaires. A control group of 38 SLE patients was included.

**Results:** 106 cSLE patients (median disease duration 20 yrs) filled in an extensive questionnaire regarding education and work participation. 61% of patients had an SDI of 1 or higher.

Education was affected in 86% of cSLE patients; choice of secondary education was affected for 60%. 58% reported their subsequent choice of career to be affected. When comparing this to the SLE patients, education and career choice were much more affected in cSLE, most logically relating to the early onset of cSLE.

Twenty-seven cSLE patients were students and excluded from analyses regarding work. Despite adjusting their career choice to their disease, 21 of the remaining 79 cSLE patients (27%) had quit their job and 13 (16%) had to reduce their working hours, due to effects of cSLE. Many cSLE patients were unemployed (41%), comparable to the unemployment rate of the SLE patients (45%) but very high compared to the 9% unemployment rate of the Dutch female population. Interestingly, more cSLE patients than SLE patients were work disabled (p = .028), with more partial work disability in the cSLE group (19% cSLE versus 5% SLE). Work disabled cSLE patients were more likely to have damage (SDI ≥ 1, p = .018).

**Conclusion:** CSLE had great impact on education and career. Despite an opportunity to adapt education and career choice to the limitations of their disease, work disability for SLE patients was higher than for SLE patients, and more common for cSLE patients with damage. Better control of disease including prevention of disease damage as well as educating patients about their possibilities to find a compatible career are of important to facilitate participation in our community.

**Disclosure of Interest**

None Declared.

**Table 24 (abstract P273).** Patient Characteristics, education, work participation

		cSLE n = 106	SLE n = 38	p*
Female		93%	95%	
Ethnicity				
White		71%	71%	
Non White		29%	29%	
Age at study visit (*median (range))*		33 (18 – 65)	39 (25 – 76)	0.001
Age at diagnosis, yrs (*median (range))*		14 (4 – 17)	28 (18 – 69)	0.000
Disease duration, yrs (*median (range))*		20 (1 – 55)	11 (1 – 34)	0.000
Current SLEDAI-2 K score (*median (range))*		4 (0 – 14)	4 (0 – 10)	
SDI-score (*median (range))*		1 (0 – 8)	1 (0 – 7)	
Patients with SLICC-DI ≥ 1		61% (65/106)	58% (22/38)	
Course of education negatively affected due to SLE		86% (91/106)	37% (14/38)	0.000
Choice of education negatively affected due to SLE		60% (63/106)	26% (10/38)	0.001
Choice of career negatively affected due to SLE		58% (62/106)	41% (14/38)	
Student		26% (27/106)	0% (0/38)	
Work status (non-students)	Dutch norm	n = 79	n = 38	
Full-time %	23%	17% (13/79)	13% (5/38)	
Part-time %	69%	41% (33/79)	42% (16/38)	
Unemployed %	8%	41% (33/79)	45% (17/38)	
Reduced working hours, (partially) due to SLE		16% (13/79)	16% (6/38)	
Quit working, (partially) due to SLE		27% (21/79)	26% (10/38)	
Never worked, (partially) due to SLE		4% (3/79)	0%	
Work disabled (fully and partially)		53 **%** (42/79)	32% (12/38)	0.028
SLICC-DI ≥ 1		83% (35/42)	67% (8/12)	

*If no *p*-values are given, differences between cSLE and SLE were not statistically significant

### P274 Cross-sectional study in mexican-mestizo patients with juvenile idiopathic arthritis (JIA) from two tertiary referal centers in mexico city. Reality of socio-economical and cultural factors contributing to discapacity

#### Raul Gutiérrez Suárez

##### Pediatric Rheumatology, Shriner Hospital For Children Mexico City, Mexico, Mexico

**Introduction:** Little is known about socio-economical and cultural factors contributing to discapacity in Mexican-Mestizo JIA patients. Some studies have found statistically significant higher proportion of poor functional status, low health-related quality of life and discapacity in comparison with children from other countries. Furthermore, in Mexico a period of ≥ 4 years is the mean time to establish an adequate diagnosis; 3 out of 10 children have developed a serious or irreversible disability at diagnosis and 1 out of 3 children have stopped their treatment because their high cost.

**Objectives:** To assess discapacity related to socio-economical and cultural factors in Mexican-Mestizo JIA patients from two tertiary referal centers in Mexico City.

**Methods:** A cross-sectional analytical study was designed in two tertiary referal centers in Mexico City. Incident and Prevalent JIA Mexican-Mestizo patients according to ILAR criteria were included. Demographics, including socioeconomical and cultural explanatory variables: referal time, time to adequate diagnosis and treatment, family income, Time for Biological treatment, parents education and social security; Clinical variables: disease activity (JADAS-71), functional capacity (CHAQ), articular and extra-articular damage (JADI-A and JADI-E) and Clinical criteria: minimal disease activity (MDA), remission with (RWT) and without treatment (RWOT); were collected. Different logistic regression analysis were conducted previous dichotomisation of the variables by a Receiver operating characteristic curve (ROC) analysis.

**Results:** 152 Mexican-Mestizo JIA patients (57% female) according with ILAR criteria were included. Age at onset (median ± IR); 6.5 (3-11) years; referal time (years): 2.2 (0.1-7); time to adequate diagnosis (years): 2.5 (0.1-7.5) time to adequate treatment; (years): 2.8 (0.2-7. ± 7.5); family income per month; 300 USD (50-1100 USD); Time for Biological treatment (years): 1.5 (0.3-10) JADAS-71: 20.4 (0-46); CHAQ: 1.1 (0-2.8); JADI-A: 15.8 (0-30); JADI-E: 4.5 (0-8). The logistic regression analysis demonstrate the following results:

**Conclusion:** Socio-cultural and economical factors leading a delay in diagnosis and treatment are an important contributor for disability in Mexican-Mestizo children with JIA.

**Disclosure of Interest**

None Declared.

**Table 25 (abstract P274).** See text for description

	Clinical Variables			
Socio-Cultural and economical explanatory variables (Cut-off value)	JADAS-71 (≥20.4) † OR (IC 95%)	CHAQ (≥1.1) † OR (IC 95%)	JADI-A (≥15.8) † OR (IC 95%)	JADI-E (≥4.5) † OR (IC 95%)
Referal time ≥ 2.2 years	NS	2.5 (1.3-27)***	3 (2.0-33) ***	NS
Time to adequate diagnosis ≥2.5 years	NS	2.8 (1.4-30) ***	3.2 (1.8-34) ***	NS
Time to adequate treatment; ≥2.8 years	1.9 (1.0-28.3)**	2.7 (1.5-28) **	3.5 (2.1-35) ***	NS
Family income ≤ 300 USD	NS	3 (1.6-35) ***	2.5 (1.3-28) ***	NS
Time for Biological treatment ≥ 2.5 years	2.0 (1.3-33)***	3.5 (1.6-38) ***	1.5 (1-28)*	1.4 (1-30)*

Cut-off value † obtained by a Receiver operating characteristic curve (ROC) analysis

***p,0.001; **p,0.01; *p,0.05 (likelihood ratio test)

***p,0.001; **p,0.01; *p,0.05

### P275 Baseline predictors of long-term remission in a Nordic juvenile idiopathic arthritis cohort

#### Ellen B. Nordal^1,2^, Veronika G. Rypdal^1,2^, Lillemor Berntson^3^, Maria Ekelund^3,4^, Kristiina Aalto^5^, Suvi Peltoniemi^5^, Marek Zak^6^, Susan Nielsen^6^, Mia Glerup^7^, Troels Herlin^7^, Ellen D. Arnstad^8,9^, Anders Fasth^10^, Marite Rygg^8,11^ and the Nordic Study Group of Pediatric Rheumatology (NoSPeR)

##### ^1^Department of Pediatrics, University Hospital of North Norway, Tromsø, Norway; ^2^Institute of Clinical Medicine, The Arctic University of Norway UIT, Tromsø, Norway; ^3^Department of Women’s and Children’s Health, Uppsala University Hospital, Uppsala, Sweden; ^4^Department of Pediatrics, Ryhov County Hospital, Jonkoping, Sweden; ^5^Department of Pediatrics, Helsinki University Children's Hospital, Helsinki, Finland; ^6^Pediatric Clinic II, Copenhagen University Clinic Rigshospitalet, Copenhagen, Denmark; ^7^Department of Pediatrics, Århus University Hospital, Århus, Denmark; ^8^Department of Laboratory Medicine, Children’s and Women’s Health, NTNU, Trondheim, Norway; ^9^Department of Pediatrics, Levanger Hospital, Levanger, Norway; ^10^Department of Pediatrics, University of Gothenburg, Gothenburg, Sweden; ^11^Department of Pediatrics, St Olavs Hospital, Trondheim, Norway

###### **Presenting author:** Veronika G. Rypdal

**Introduction:** Juvenile idiopathic arthritis (JIA) is a chronic childhood disease that often persists into adult life, and the prognosis varies markedly. Baseline predictors of long-term outcome may identify children who would benefit from early aggressive treatment.

**Objectives:** To evaluate potential baseline predictors of remission eight years after disease onset in a cohort of Nordic children with JIA in a population-based setting.

**Methods:** Consecutive cases of JIA from defined geographical areas in Denmark, Finland, Sweden and Norway with disease onset in 1997-2000, >7 years of follow-up, and a baseline visit within 12 months after disease onset were prospectively included. Logistic regression analysis was performed to assess potential baseline predictors of long-term remission using the Wallace criteria.

**Results:** Among the 389 participants, 67.1% were female and median age at onset of disease was 5.5 (IQR 2.5-9.7) years. Eight years after disease onset 43.7% were in clinical remission off medication. The following variables registered at the first study visit were significant predictors for not achieving remission off medication: high number of active joints, ankle, subtalar and tarsal joint arthritis, high platelet count, ESR and CRP, presence of HLA B27, and anti-nuclear antibodies analyzed by immunofluorescence (ANA-IF), high patient's/parent’s and doctor’s global assessment and CHAQ score. Oligoarticular onset JIA predicted remission. (Table 26).

**Conclusion:** In a prospective Nordic JIA cohort study we identified high number of active joints, foot joint involvement, high ESR, presence of HLA B27 and ANA-IF among significant predictors of not being in long-term drug-free remission.

**Disclosure of Interest**

None Declared.

**Table 26 (abstract P275).** Selected clinical predictors of remission status eight years after disease onset

	N (%)^*^	Total	No remission N (%)^*^	Remission N (%)^*^	OR (95% CI)
Oligo JIA onset	194 (49.9)	389	96 (43.8)	98 (57.6)	0.6 (0.4-0.9)
Number of active joints^†^	1 (0-3)	389	1(0-3)	0(0-2)	1.2 (1.1-1.3)
Tarsal joint arthritis	33 (8.5)	389	28 (12.8)	5 (2.9)	4.8 (1.8-12.8)
ESR (OR pr 10 mm/hour)^†^	14 (8-29)	315	18 (10-39)	11 (6-20)	1.3 (1.2-1.5)
HLA-B27 positive	74 (20.4)	363	52 (24.8)	22 (14.4)	2.0 (1.1-3.4)
ANA-IF	113 (29.8)	379	74 (35.1)	39 (23.2)	1.8 (1.1-2.8)
Patient’s global assessment VAS^†^	1.0 (0.2-3.0)	235	1.8 (0.5-3.5)	0.5 (0.0-2.0)	1.3 (1.1-11.5)
CHAQ score^†^	0.3 (0.0-1.0)	241	0.6 (0-1.2)	0.0 (0.0-0.6)	2.3 (1.5-3.5

^*^Values are numbers N (%) (†median (interquartile range (IQR))). *OR* Odds ratio, *CI* confidence interval, *ESR* Erythrocyte sedimentation rate, *VAS* Visual analogue scale, *CHAQ* Childhood health assessment questionnaire

### P276 Outcome of an inception cohort of juvenile idiopathic arthritis in the 21st century

#### Ana Catarina Duarte, Sandra Sousa, Lídia Teixeira, Ana Cordeiro, Ma José Santos

##### Rheumatology, Hospital Garcia de Orta, Almada, Portugal

###### **Presenting author:** Ana Catarina Duarte

**Introduction:** Juvenile idiopathic arthritis (JIA) activity may persist for many years, with progression into adulthood. The main goal of treatment is to achieve long-lasting remission or the lowest possible disease activity. Major advances in the management of JIA occurred in recent years, particularly due to the emergence of new treatments that can alter the disease course.

**Objectives:** Characterize an inception cohort of JIA patients established in 2000 and evaluate disease outcomes according to International League of Association for Rheumatology (ILAR) categories.

**Methods:** We conducted an analysis of all JIA patients followed in our Rheumatology outpatient clinic since 2000, whose first appointment occurred within 6 months of the diagnosis. We collected demographic features, age at JIA diagnosis, JIA category, duration of follow up, medication and disease activity. Variables associated with clinical remission were assessed.

**Results:** In total, 52 JIA patients were analysed, 27 females, with a mean age of 14.1 (±6.3) years and mean age at diagnosis of 7.6 (±5.1) years. Regarding ILAR categories, 5 patients were classified as systemic, 5 psoriatic arthritis (PsA), 10 enthesis-related arthritis (ERA), 7 polyarthritis (3 rheumatoid factor [RF] positive; 4 negative), 21 oligoarthritis (16 persistent; 5 extended) and 4 undifferentiated arthritis (UA).

Positive antinuclear antibodies (≥1/160) were found in 24 patients, RF in 3 patients and HLAB27 in 8 patients.

Regarding treatment options, 69.2% patients were treated with methotrexate (MTX) and 25% (n = 13) received a biological agent at some point during disease course. From these 13 patients, 2 stopped biological therapy due to clinical remission and 1 due to inefficacy (failure of 2 TNF inhibitors). After a mean follow-up of 5.1 (±4.3) years, 46.1% of the patients are in clinical remission, of which 66.7% are off medication (2 of these was previously treated with biologics). Specific treatment options and clinical outcomes according to ILAR category are shown in the table below.

In the whole cohort, 5 patients were lost to follow-up and 10 patients have already achieved adulthood and maintain follow-up in our department. From these, 2 fulfil criteria for Still´s disease, 2 for rheumatoid arthritis and 6 for spondylarthritis (1 PsA, 4 ankylosing spondylitis and 1 arthritis associated with inflammatory bowel disease).

When evaluating remission rates, no significant differences were found regarding ILAR categories, with 37.5% of the patients in remission classified as oligoarthritis, 20.8% ERA and 16.7% PsA (p = 0.5). Neither demographic characteristics nor disease activity at presentation or immunological phenotype were associated with clinical remission.

**Conclusion:** In this cohort established in the 21st century, almost half of JIA patients achieved clinical remission after a mean follow-up of 5 years. We could not identify any clinical/immunological predictors of remission, although the proportion of oligoarthritis JIA achieving remission is higher than the other categories. Development of new therapeutic agents in association with early rehabilitation can explain the trend towards higher remission rates independently from clinical variables, although the small sample does not allow definitive conclusions.

**Disclosure of Interest**

None Declared.

**Table 27 (abstract P276).** See text for description

Treatment option	Clinical outcome	Systemic	RF+ polyarthritis	RF- polyarthritis	Oligoarthritis	ERA	PsA	UA
MTX monotherapy	Active disease	1	0	3	9	1	0	0
	Remission on medication	0	1	0	4	1	1	0
	Remission off medication	1	1	0	2	1	1	1
Biologics ±MTX	Active disease	2	1	1	3	2	1	1
	Remission on medication	0	0	0	0	0	0	0
	Remission off medication	1	0	0	0	1	0	0
No systemic medication/ only NSAIDs	Active disease	0	0	0	0	2	0	0
	Remission	0	0	0	3	2	2	0

Note: Two UA JIA patients treated with other DMARDs were excluded from the table

### P277 Potential biomarkers of disease activity in juvenile idiopathic arthritis: data from the portuguese register, REUMA.PT.

#### Ana Filipa Mourão^1^, Maria José Santos^1,2^, Mónica Eusébio^3^, Ana Lopes^4^, Filipa Oliveira-Ramos^5^, Manuel Salgado^6^, Paula Estanqueiro^6^, José Melo-Gomes^7^, Fernando Martins^3^, José Costa^8^, Carolina Furtado^9^, Ricardo Figueira^10^, Iva Brito^11^, Jaime C. Branco^4^, João E. Fonseca^1^, Helena Canhão^12^

##### ^1^Rheumatology Research Unit, Instituto de Medicina Molecular da Faculdade de Medicina de Lisboa, Lisbon, Portugal; ^2^Rheumatology, Hospital Garcia de Orta, Almada, Portugal; ^3^Portuguese Society of Rheumatology, Lisbon, Portugal; ^4^CEDOC, Nova Medical School, Lisbon, Portugal; ^5^Rheumatology, Lisbon Medical Centre, Hospital de Santa Maria, Centro Hospitalar de Lisboa Norte, Lisbon, Portugal; ^6^Pediatrics, Centro Hospitalar da Universidade de Coimbra, Coimbra, Portugal; ^7^Rheumatology, Portuguese Institute of Rheumatology, Lisbon, Portugal; ^8^Rheumatology, Unidade de Saude do Alto Minho, ULSAM, Hospital Conde de Bertiandos, Ponte de Lima, Portugal; ^9^Rheumatology, Hospital do Divino Espírito Santo, Ponta Delgada, São Miguel, Portugal; ^10^Rheumatology, Hospital Dr. Nélio Mendonça, Funchal, Portugal, ^11^Rheumatology, Centro Hospitalar São João, Porto, Portugal; ^12^EPIDOC, Nova Medical School, Lisbon, Portugal

###### **Presenting author:** Ana Filipa Mourão

**Introduction:** The classical inflammatory markers, such as C-reactive protein and erythrocyte sedimentation rate (ESR) often inadequately reflect disease activity, but on the other hand, there are few specific biomarkers that can be helpful in managing the different categories of juvenile idiopathic arthritis (JIA). The search for other biomarkers in JIA is attracting increased interest and a number of biomarkers have shown potential for predicting clinical phenotype, disease activity and severity, clinical remission and relapse, response to treatment and disease course over time.

**Objectives:** To assess the serum levels of DKK1, IL1β, INFγ, IL10 and IL17 in patients with JIA, and detect their relation to disease activity.

**Methods:** Demographical and clinical data from 316 patients with JIA registered in the Reuma.pt were collected and a blood sample from each patient was obtained for cytokine determination. Disease activity was evaluated using the Juvenile Arthritis Disease Activity Score in 27 joints (JADAS27) and the Childhood Health Assessment Questionnaire (CHAQ) score. Serum levels of DKK1, IL1β, INFγ, IL10 and IL17 levels were determined by ELISA. Multivariate linear regression for each JIA category was used for the analysis of the relation between DKK1, IL1β, INFγ, IL10 and IL17 levels and JADAS27 (and its individual components) and CHAQ, adjusting for gender, body mass index, age and disease duration.

**Results:** 316 JIA patients, 65% female, with mean age 17.1 ± 9 years and mean disease duration 8.1 ± 1.4 years. The distribution of JIA categories was: 105 persistent oligoarticular, 51 extended oligoarticular, 51 polyarticular rheumatoid factor (RF) negative, 30 polyarticular RF positive, 26 systemic, 36 enthesitis-related arthritis and 17 psoriatic arthritis. The analysis of the 7 JIA categories revealed no relation between the serum levels of these cytokines and disease activity measured by JADAS27. In patients with polyarticular RF positive we have found an inverse relation between DKK1 levels and CHAQ score (ß = -0.003; adjusted p = 0.025) and, in polyarticular RF negative patients an inverse relation between DKK1 levels and ESR (ß = -0.031; adjusted p = 0.03). In patients with psoriatic arthritis there was a positive relation between IL1β, INFγ and IL17 with CHAQ (ß = 0.012 and adjusted p = 0.007; ß = 0.004 and adjusted p = 0.03; ß = 0.034; adjusted p = 0.04; respectively) and an inverse relation between IL10 levels with CHAQ (ß = -0.011; adjusted p = 0.005).

**Conclusion:** Our data reinforce the need of biomarkers studies of disease activity in the different JIA categories. Interestingly, mostly in JIA psoriatic arthritis patients, we have identified potential biomarkers of disease activity. Larger studies are needed in order to validate these results. The incorporation of effective and reliable biomarkers into routine practice might facilitate adoption of a stratified approach to investigation and management, foster the implementation of research into the design of personalized and targeted therapies, and ultimately lead to more rational and effective clinical care.

**Disclosure of Interest**

None Declared.

### P278 Serum receptor activator of NFKB ligand (RANKL) and osteoprotegerin (OPG) in juvenile idiopathic arthritis: relation to disease activity

#### Ana F. Mourão^1,2,3^, Maria Jose Santos^2,4^, Mónica Eusébio^5^, Ana Lopes^6^, Filipa Oliveira-Ramos^2,7^, Manuel Salgado^8^, Paula Estanqueiro^8^, José Melo-Gomes^9^, Fernando Martins^5^, José Costa^10^, Carolina Furtado^11^, Ricardo Figueira^12^, Iva Brito^13^, Jaime C. Branco^1,14^, João E. Fonseca^2,7^, Helena Canhão^15^

##### ^1^Rheumatology, HOSPITAL EGAS MONIZ, CHLO, EPE, Lisbon, Portugal; ^2^Rheumatology, Instituto de Medicina Molecular, UIR, Lisbon, Portugal; ^3^Rheumatology, CEDOC, Nova Medical School, Lisbon, Portugal; ^4^Rheumatology, Hospital Garcia de Orta, Lisbon, Portugal; ^5^Portuguese Society of Rheumatology, Lisbon, Portugal; ^6^Nova Medical School, Lisbon, Portugal; ^7^Rheumatology, Lisbon Medical Centre - Santa maria Hospital, Centro Hospitalar de Lisboa Norte, EPE, Lisbon, Portugal; ^8^Pediatric, Centro Hospitalar de Coimbra, Coimbra, Portugal; ^9^Rheumatology, Portuguese Institute of Rheumatology, Lisbon, Portugal; ^10^Rheumatology, Centro Hospitalar do Alto Minho, ULSAM, Ponte de Lima, Portugal; ^11^Rheumatology, Hospital do Divino Espírito Santo, Ponta Delgada, São Miguel, Portugal; ^12^Rheumatology, Hospital Dr. Nélio Mendonça, Funchal, Madeira, Portugal; ^13^Rheumatology, centro Hospitalar de São João, Porto, Portugal; ^14^CEDOC; ^15^EPIDOC, Nova Medical School, Lisbon, Portugal

###### **Presenting author:** Ana F. Mourão

**Introduction:** Among the TNF family, receptor activator of nuclear factor kB (RANK), receptor activator of nuclear factor kB ligand (RANKL), and osteoprotegerin (OPG), a soluble receptor that inactivates RANKL, are known to interfere in the immune system, bone metabolism, and endocrine functions. The clinical relevance of observations of serum levels of RANKL and OPG in Juvenile idiopathic arthritis (JIA) is not clear.

**Objectives:** To assess the serum levels of RANKL and OPG in JIA patients and to detect their relation to disease activity in the different JIA categories.

**Methods:** Consecutively recruited JIA patients were clinically examined, the Juvenile arthritis disease activity score in 27 joints (JADAS27) calculated and Childhood Health Assessment Questionnaire (CHAQ) used to measure the functional status. Routine laboratory examinations were recorded and serum RANKL and OPG levels were determined by ELISA.Nonparametric tests and multivariate linear regression were used for analysis the relation between the levels of RANKL and OPG versus JADAS27 and CHAQ, adjusting for gender, body mass index, age, disease duration and JIA categories.

**Results:** 316 JIA patients, 65% female, with mean age 17.10 ± 9.01 years and mean disease duration 8.11 ± 1.41 years. The distribution of JIA categories was: 105 persistent oligoarticular, 51 extended oligoarticular, 51 polyarticular RF negative, 30 polyarticular RF positive, 26 systemic, 36 enthesitis-related arthritis and 17 psoriatic arthritis. Taking all JIA patients, there was no relationship between serum RANKL levels and JADAS27 or CHAQ. However, when we analyzed the different JIA categories, in polyarticular RF positive JIA there was a significant positive relation between serum RANKL levels and JADAS27 (ß = 0.226; adjusted p = 0.013) and also between RANKL and CHAQ (ß = 0.027; adjusted p = 0.046). Regarding OPG, considering all the JIA subtypes, there was no correlation with JADAS27 or CHAQ. However, OPG levels were inversely correlated with the number of active joints, one of the components of JADAS27 (ß = -0.007; adjusted p = 0.003).

**Conclusion:** Our data reveal that RANKL levels are associated with disease activity and functional impairment in polyarticular RF positive JIA, reinforcing the attractive role of therapeutic agents targeting RANKL. In all JIA categories, OPG levels were inversely related to the number of active joints, denoting the possible relationship to disease activity.More studies are needed in order to better understand the role of these cytokines in JIA.

**Disclosure of Interest**

None Declared.

### P279 Health professionals’ and nurses’ confidence in managing paediatric rheumatic disease in Australia

#### Andrea Coda^1^, Samuel Cassidy^2^, Kerry West^3^, Gordon Hendry^4^, Debra Grech^5^, Julie Jones^6^, Fiona Hawke^1^, Davinder Singh Grewal^7^

##### ^1^School of Health and Science, Faculty of Health and Medicine, The University of Newcastle, Ourimbah, Australia; ^2^School of Health and Science, Faculty of Health and Medicine, The University of Newcastle, Ourimbah, Australia; ^3^Physiotherapy, The West Meads Children’s Hospital, Westmead, Australia; ^4^Institute for Applied Health Research, School of Health & Life Sciences, Glasgow Caledonian University, Glasgow, UK; ^5^Sydney Children Hospital (SCH) Randwick, Sydney, Austrlia; ^6^Paediatrics and Child Health, The University of Sydney, Sydney, Australia; ^7^The University of NSW, The University of Sydney, Sydney, Australia

###### **Presenting author:** Andrea Coda

**Introduction:** Rheumatic disease (RD) is an important cause of acquired disability in children. The aim of this study was to explore health professionals’ (HP) and nurses’ confidence in the treatment of paediatric rheumatology patients; the perceived adequacy of their undergraduate and postgraduate paediatric rheumatology education; and perceived utility of a number of forms of educational opportunities for these professional groups.

**Objectives:** The aims of this study were to explore: (1) HPs’ and nurses’ confidence in the treatment of paediatric rheumatology patients; (2) the perceived adequacy of their undergraduate and postgraduate paediatric rheumatology education; and (3) the perceived utility of a number of forms of educational opportunities for these professional groups.

**Methods:** Participants were recruited through the ‘Australian Physiotherapy Association’, ‘Occupational Therapy Australia’, ‘Rheumatology Health Professionals Association’, ‘The Australian Psychological Society’, ‘Australian Podiatry Association’, and ‘The Allied To Kids Newsletter’.

**Results:** 117 participants, mostly occupational therapists and physiotherapists, working predominantly in the urban public health care system of NSW were recruited. Most participants treated patients <18 years and has had experience treating RD in children. 77.9% of participants reported being ‘not confident at all’, ‘not confident’ or ‘neutral’ in treating children with RD despite 65.1% of participants reporting having treated >1 paediatric rheumatology case in the past month. Results highlighted that 67.2% of all participants perceived their undergraduate education as being inadequate. ‘Journals’ or ‘texts books’ were used by 49.3% of participants as their primary source of continued professional development (CPD) with 39.3% of participants avoiding the field entirely. Seminars, workshops and skills days were suggested to be of ‘great benefit’.

**Conclusion:** Participants lacked confidence in treating children with RD. Increased accessibility to seminar/workshop opportunities was felt to be of the greatest benefit and is required in order to improve confidence in the recognition, referral and effective management in paediatric rheumatology.

**Disclosure of Interest**

None Declared.

### P280 A survey of parent and carer experiences and expectations of paediatric rheumatology care in New South Wales

#### Andrea Coda^1^, Julie Jones^2^, Debra Grech^3^, Davinder Singh Grewal^4^

##### ^1^School of Health and Science, Faculty of Health and Medicine, The University of Newcastle, Ourimbah, Australia; ^2^Paediatrics and Child Health, The University of Sydney, Sydney, Australia; ^3^Sydney Children Hospital (SCH) Randwick, Sydney, Australia; ^4^The University of NSW, The University of Sydney, Sydney, Australia

###### **Presenting author:** Andrea Coda

**Introduction:** Rheumatic disease (RD) in childhood and adolescence is a significant cause of short and long-term disability. The provision of health care to children with RD is challenging with agreement that a multidisciplinary approach is best practice. Early diagnosis and management by a multidisciplinary team is the gold standard for JIA management and delays in diagnosis may significantly impair the outcomes of children diagnosed with RD with reduced quality of life, increased pain level and worse long-term prognosis for children with RD reported.

**Objectives:** This survey of parent and carers aims to establish the level of care and services currently provided to children diagnosed with Rheumatic Diseases (RD) in New South Wales (NSW), Australia.

**Methods:** The survey included parents and carers of children presenting to paediatric rheumatology (PR) services in NSW. Subjects attending PR clinics in both public and private settings were invited to complete an online or paper survey.

**Results:** Overall 148 surveys were completed. The process of obtaining the diagnosis of RD was described as being ‘difficult’ or ‘very-difficult’ in 56.9% (n = 83) of the surveyed cohort and 41.5% (n = 61) consulted 4 or more different clinicians prior to diagnosis. Between symptom onset and final diagnosis, 42.6% (n = 63) participants reported a delay of 5 months or more, and 16.9% (n = 25) waited longer than 12 months. Ultimately, 91% (n = 134) were referred to a paediatric rheumatologist and 63.6% (n = 94) were seen within 4 weeks from initial referral. More than half of respondents felt that general practitioners and general paediatricians were not aware of RD. Overall, improved knowledge of paediatric rheumatology diseases amongst general practitioners, improved access to PR, improved educational materials for patient’s and families, access to specialty rheumatology nurses and coordinated rheumatology teams would have significantly improved the overall experience of their child’s disease.

**Conclusion:** Children with RD in NSW still experience significant delays from symptom onset to definite diagnosis through consultations with multiple health-care professionals. Reassuringly, when the referral to PR services is made, patients are usually seen faster than other international standards

**Disclosure of Interest**

None Declared.

### P281 Myeloid related proteins 8 and 14 (MRP 8/14) - potential biomarkers of arthritis in children with trisomy 21?

#### Charlene Foley^1^, Orla Killeen^1^, Emma MacDermott^1^, Douglas Veale^2^, Ursula Fearon^3^

##### ^1^National Centre for Paediatric Rheumatology, Our Lady's Children's Hospital Crumlin, Dublin, Ireland; ^2^Rheumatology, St Vincent's University Hospital, Dublin, Ireland; ^3^Rheumatology, Trinity College, Dublin, Ireland

###### **Presenting author:** Charlene Foley

**Introduction:** To date no specific markers exist in clinical practice to predict disease activity & outcome in JIA. MRP 8/14 are calcium-binding proteins secreted by infiltrating phagocytes in synovial inflammation. Studies have shown that their serum (Se) concentrations correlate sensitively & specifically with synovial inflammation in JIA. To date there have been no studies looking specifically at Se or synovial fluid (SF) levels of MRP 8/14 in Down’s Arthropathy (DA).

Research Question(s):

1. Are MRP8/14Se/SF accurate markers of inflammation in DA,

2. Do levels differ in DA compared with JIA?

**Objectives:** To determine the accuracy of standard (CRP and ESR) and novel (MRP 8/14) inflammatory markers, as biomarkers of disease activity in DA compared with their use in JIA.

**Methods:** New cases of JIA & DA attending our centre had blood drawn to measure CRP, ESR & MRP 8/14 levels. Corresponding AJC was documented. Paired SF samples were taken for analysis from children requiring steroid JIs. Se & SF concentrations of MRP 8/14 were determined by sandwich ELISA.

**Results:** SeMRP 8/14, ESR & CRP were measured in DA (n = 34) & JIA (n = 50) patients. In a subgroup, MRP 8/14 levels were also quantified in paired SF, DA (n = 3) & JIA (n = 21). At diagnosis, ESR & CRP levels were raised in a lower percentage of DA cases compared with our JIA cohort, even though on average, a higher AJC was observed in the DA cohort. Se MRP 8/14 levels were significantly higher in JIA compared to DA, levels of which correlated with ESR (r = 0.312, p < 0.05). No correlation between SeMRP 8/14 & systemic clinical markers (CRP & ESR) was observed for DA. We therefore examined if MRP 8/14 levels were higher at the site of inflammation, i.e. in SF from the joints of children with active DA. Preliminary data has shown MRP 8/14 levels are higher in SF compared with Se in DA. Our CRP, ESR & Se/SF MRP 8/14 observations in DA may suggest that there is dissociation between systemic & local inflammation in this cohort.

**Conclusion:** DA is a more challenging condition than JIA in light of confounding illness & the often-associated developmental delay & non-verbal state. In DA a simple biomarker of disease would be invaluable. Our preliminary results suggest that children with DA (& JIA) have elevated SF levels of MRP 8/14 that correlate to disease activity. In JIA, SF concentrations of MRP 8/14 are significantly higher than their paired Se samples, however our results show significant positive correlation between the two. This suggests that SeMRP 8/14 levels are potentially accurate markers of SF levels, & in turn accurate markers of disease activity in JIA. This was not the case in our DA cohort and may suggest a functional role for MRP 8/14 at the site of inflammation in DA.

**Disclosure of Interest**

None Declared.

### P282 Health status evaluation of childhood rheumatic disease

#### Dilek Konukbay^1^, Erkan Demirkaya^2^

##### ^1^Department of Child Health and Disease, Gulhane Military Medical Academy, Ankara, Turkey; ^2^Department of Child Rheumatology, Gulhane Military Medical Academy, Ankara, Turkey

###### **Presenting author:** Dilek Konukbay

**Introduction:** The patient's clinical history and life exeriences are of great importance and guide as well as the diagnosis, treatment planning, monitoring, laboratory and radiological examinations in the evaluation of the health status of rheumatic diseases.

**Objectives:** The objective of the study is to examine the methods used in the evaluation of the health status of children with the childhood rheumatic diseases.

**Methods:** A comprehensive literature survey was conducted, and measurement tools used in the evaluation of the health status of the children with the rheumatic diseases were studied.

**Results:** In the evaluation of children's health; measurement tools and development of these tools which enable an holistic approach to the patient are among the important issues of today as well as the blood-urine tests and radiological examinations. There are two types of measurement tools used for children; general and disease-targeted.

General measurement tools are designed to be relevant to everyone and allow for a relative comparison of the different disease patient groups.

The development of disease-targeted measurement tools focus on the gaps of the general measurement tools for the specific conditions of the disease. Disease-targeted measurements are more sensitive to detect clinically important changes and thus have more applicability in the clinical study.

There are two kinds of health status evaluation scales for the children; the first one is the form that the patient fills the report (patient self-report) and the second one is filled by the family (parent proxy-report) if the child is too small or has cognitive impairment or unable to complete the form due to the severity of the disease.

Filling out these two forms either by the child or the family will play an important role in the evaluation of perspectives for the treatment and guide the provision of holistic care of the disease.

**Conclusion:** Activities related to rheumatic diseases in the the past concentrated more on the disease activity measurement than the assessment emphasis of physical, social and mental function of health as it is today. Measurement tools enable healthcare personnel to evaluate their patients and their health without an invasive procedure.

**Disclosure of Interest**

None Declared.

### P283 The physical activity level, pain, well-being and functional abilities in patients with juvenile idiopathic arthritis

#### Ela Tarakci^1^, Nilay Arman^1^, Kenan Barut^2^, Sezgin Şahin^2^, Amra Adrovic^2^, Ozgur Kasapcopur^2^

##### ^1^Faculty of Health Sciences, Division of Physiotherapy and Rehabilitation, Istanbul University, Istanbul, Turkey; ^2^Medical Faculty of Cerrahpasa, Department of Pediatric Rheumatology, Istanbul University, Istanbul, Turkey

###### **Presenting author:** Ela Tarakci

**Introduction:** Juvenile idiopathic arthritis (JIA) is the most common chronic pediatric rheumatic disease and an important cause of acquired impairment and disability in children and adolescents. Patients with JIA commonly experience acute and chronic pain, decreased mobility, and joint stiffness leading to restrictions on activities and isolation from their peers. Physical activity is essential for the social, emotional, and cognitive development of children and adolescents with JIA.

**Objectives:** The aim of this study was to investigate physical activity level functional ability, pain, and well-being in children and adolescents with JIA and was to assess relations between this parameters.

**Methods:** Cross-sectional study design including patients with JIA aged between 7 and 18 years and healthy controls was used. Patients were diagnosed by a pediatric rheumatologist using the International League of Associations for Rheumatology criteria. Sociodemographic and clinical features were assessed. Physical activity level and energy expenditure were assessed with a 1-day activity diary. Pain and overall well-being were measured using a visual analog scale (VAS). Functional ability was assessed with the Childhood Health Assessment Questionnaire (CHAQ).

**Results:** Fifty-two patients and 48 controls were included with a mean age of 12.13 ± 2.92 and 11.27 ± 1.59 years, respectively. The mean disease duration was 64 months. The JIA group had significantly less time in physical activity (p = 0.001), decrease in energy expenditure (p = 0.04), and higher CHAQ scores (p = 0.001) compared with the control group. In the JIA group, significant relationships were found between the number of involved joint and disease duration (r = 0.44, p = 0.001) and VAS pain (r = 0.30, p = 0.02). Significant relationships were found between VAS overall well-being, CHAQ (r = 0.37, p = 0.001) and VAS pain (r = 0.41, p = 0.001).

**Conclusion:** This study has shown a significantly lower physical activity level, energy expenditure, and functional ability in patients with JIA compared with healthy controls. The results of our study showed that there was no significant relationship between physical activity and functional ability level in patients with JIA. The likely cause for this relationship may be multifactorial and needs to be revealed to improve assessment strategies.

**Disclosure of Interest**

None Declared.

### P284 Outcome measures in rheumatology (OMERACT): developing a new core domain set of outcome measures for juvenile idiopathic arthritis

#### Jane Munro^1^, Alessandro Consolaro^2^, Esi Morgan^3^, Meredith Riebschleger^4^, Jennifer Horonjeff^5^, Vibeke Strand^6^, Clifton Bingham^7^

##### ^1^Rheumatology, Royal Children's Hospital, Melbourne, Australia, ^2^Istituto G. Gaslini, Genova, Italy, ^3^Rheumatology, Cincinnati Children’s Hospital Medical Center, Cincinatti, USA, ^4^Pediatrics & Communicable Diseases, University of Michigan, Michigan, USA, ^5^Columbia University Medical Center., New York, USA, ^6^Immunology/Rheumatology, Stanford University, Stanford, USA, ^7^Rheumatology/Allergy, Johns Hopkins Arthritis Center, Baltimore, USA

###### **Presenting author:** Jane Munro

**Introduction:** JIA is the most common rheumatologic disease of childhood resulting in both joint & extra-articular limitations such as blindness from uveitis. The current JIA Core Set (ACR Pedi 30), which prescribes the items that should be assessed in trials for children with JIA, was developed in 1997 & has not been updated since. Over the past 20 years, both the science of measurement & the treatment of JIA have advanced. The importance of patient/caregiver input into the research process has increasingly been recognized. Since 2015 the OMERACT JIA Core Set Group has been working together to develop an expanded JIA core set to facilitate research & improve clinical practice. The workgroup is an international collaboration of rheumatology researchers, clinicians, patients, parents of children with JIA, measurement specialists, industry partners & regulators. The data-driven iterative consensus process involving all relevant stakeholder groups has been successful in improving the comparability & completeness of studies in several rheumatologic diseases.

**Objectives:** To identify an expanded Core Domain Set to be used in JIA clinical trials including relevant patient outcomes & update the current clinical outcomes by reaching consensus of all relevant stakeholders through the OMERACT Filter 2.0. The core set will also be examined across JIA categories to ensure content relevance.

**Methods:** An OMERACT JIA Core Set Group was formed with initial membership of 24 representatives from USA, Canada, Australia, a& Europe (Italy), a patient representative among the leadership, & two OMERACT Executive Committee members. The OMERACT process will be followed, which is collaborative & peer reviewed. Using multiple strategies including a systematic literature review, online & in-person focus groups with children, adolescents & parents, patient and provider Delphi exercises, data from observational studies & consensus voting at OMERACT conferences, a consensus-based definition for the JIA Core Set & identification of relevant domains to measure will be developed.

**Results:** The systematic review of the literature is currently in process. A MEDLINE search yielded 5956 citations. After initial screening, 823 citations were assigned to reviewers for full-text review, & 3843 citations were excluded from further consideration. The remaining 1290 citations will require adjudication due to disagreements between reviewers. Three forms of qualitative research forms the basis of this work in-person focus groups with parents; an innovative method of online focus groups with patients & parents; & disease impact ideas generation study of 120 parents of JIA patients, identifying 21 provisional domains of disease impact.

**Conclusion:** An international collaboration between clinicians and multiple stakeholders has begun using the OMERACT process to develop an expanded Core Domain Set for assessment of JIA in clinical trials. The initial results of the preliminary phases of the work have supported a need to reconsider the current JIA Core Set to include patient/caregiver-centered outcomes. Data collected thus far will inform the development of the list of domains to be tested in a comprehensive Delphi process for pediatric rheumatology stakeholders: researchers, health care providers and patients/caregivers. Continued participation in the rigorous OMERACT process with broad internationally representative involvement of clinicians, researchers, patients, industry and regulators will help to refine the important outcomes that will ultimately be included in the final JIA Core Set.

**Disclosure of Interest**

None Declared.

### P285 Outcome of Filipino children with lupus nephritis treated with a modified regimen using cyclophosphamide

#### Ma. Theresa M. Collante

##### University of Santo Tomas Hospital, Manila, Philippines

**Introduction:** The current therapeutic strategy for childhood-onset lupus nephritis (LN) involves an induction phase which aims to promote remission, and a maintenance phase to prevent relapses and control disease. Various regimens have been used by different centers worldwide, which may differ in the drug of choice and dosage, as well as in the duration of the induction and maintenance phases, because therapeutic response and report of adverse events vary according to the protocol used and in patient demographics. Although the outcome of lupus nephritis has remarkably improved, a significant proportion still do not attain remission despite various modifications in dose and frequency of the pulses. Achieving the goal of treatment of disease control and maintenance of remission should be hand-in-hand with very minimal side effects, if none is not possible.

**Objectives:** This study evaluated treatment outcome and adverse event occurrence in Filipinos with childhood-onset LN who received 9 monthly and 5 quarterly cyclophosphamide pulses.

**Methods:** A chart review was done on patients seen from year 2006 to 2014 at the University of Santo Tomas Hospital who completed the modified regimen.

**Results:** A total of 19 patients completed the modified cyclophosphamide pulse therapy (94.7% female, mean age 11.2 + 3.7 years at lupus diagnosis, mean nephritis duration upon completion of treatment 30.6 + 5.2 months), with a minimum follow up duration of one year. At 9 months of treatment, 47.4% (9/19) already reached complete remission, and 52.6% (10/19) were in partial remission. Upon completion of 9 monthly and 5 quarterly pulses, 94.7% (18/19) was with complete treatment response. One patient (5.3%) relapsed during the maintenance phase and was in partial remission at the end of the treatment. The random urine protein:creatinine ratio and disease activity were significantly improved in all 19 patients. Treatment failure was not noted in any of the patients at the end of 9 months and at the completion of treatment. Reported adverse events were gastrointestinal symptoms (100%), mild infections (94.7%), alopecia (89.5%), severe infections (10.5%), menstrual irregularities (33.3%), and hematologic disturbances (26.3%). All of the 19 patients are in remission during their most recent follow up. With the above data, we find that extension of the induction therapy by three months using standard dosing while maintaining the total cumulative dose by shortening the maintenance phase resulted in a favorable short-term outcome and adverse event occurrence, which is comparable to outcomes of other Asian cohorts.

**Conclusion:** A modified regimen of 9 monthly and 5 quarterly cyclophosphamide pulses may be an effective therapeutic option for childhood-onset LN.

**Disclosure of Interest**

None Declared.

### P286 Assessment of disease activity and prognosis in a group of JIA patients

#### Margarita Ganeva^1^, Stefan Stefanov^1^, Albena Telcharova^1^, Dimitrina Mihaylova^1^, Radoslava Saraeva^2^, Reni Tzveova^2^, Radka Kaneva^2^, Adelina Tsakova^3^, Katya Temelkova^1^ and GRANT Medical University, Sofia 68/2015

##### ^1^Pediatric Rheumatology, University Children’s Hospital, Medical University Sofia, Sofia, Bulgaria; ^2^Medical Chemistry and Biochemistry, Molecular Medicine Center, Medical University Sofia, Sofia, Bulgaria; ^3^Central Clinical Laboratory, University Hospital "Alexandrovska", Medical University Sofia, Sofia, Bulgaria

###### **Presenting author:** Margarita Ganeva

**Introduction:** JIA is the most common chronic inflammatory joint disease with variable prognosis.

**Objectives:** To assess disease activity and investigate the prognosis in a Bulgarian cohort of children with JIA. Assessment of disease activity in children with JIA includes measurement of laboratory parameters of inflammation. Recently different gene polymorphisms have been shown to influence response to treatment in JIA. We investigated the distribution of specific polymorphisms in a small group of patients.

**Methods:** Samples were collected from 75 patients (50 girls and 25 boys) fulfilling the ILAR criteria for JIA with a median age of 8,40 yrs. MRP 8/14 was measured by Bühlmann MRP8/14 Calprotectin ELISA kit (Bühlmann Laboratories, Switzerland). The levels of IL-6 were measured using immunoturbidimetric assay. CRP and ESR were measured as part of routine clinical assessment. Genotyping of C677T and A1298C polymorphisms of the MTHFR gene was performed in 33 of the 75 patients. The latter group of patients was further subdivided based on the presence or absence of concomitant biological treatment.

**Results:** Oligoarthritis accounts for 56% of the observed patients with the relapse subtype of oligoarthritis being the biggest subgroup – 36%. The highest serum levels of MRP 8/14, ESR and CRP were observed in the polyarthritis subgroup (n = 12) with newly onset disease. Good correlation was found between levels of MRP 8/14 and CRP in the whole group (r = 0.68) with the strongest correlation observed in the polyarthtitis newly onset subgroup (r = 0.92). A strong correlation was found between levels of MRP 8/14 and ESR in the systemic subtype (r = 0.91). The strongest correlation between CRP and IL-6 was noticed in the oligoarthritis newly onset subgroup (r = 0.74). There was no significant difference in the metothrexate dose between the different JIA subtypes with a median dose of 7,68 mg/m^2^ for the whole cohort. The highest percentage – 85.7% of children receiving biological treatment was found in those with polyarthritis experiencing relapse. Genetic polymorphisms C677T and A1298C were investigated in two different groups of patients– 19 patients treated with biological agents and 14 patients treated with methotrexate monotherapy. No statistically significant difference of genotype polymorphism distribution was found between the two groups (chi-square = 1.34 for C677T; chi-square = 3.45 for A1298C). We observed three homozygous patients for the T-allele of C677T. In only one of them side effects (headache) associated with the MTX treatment was noticed.

**Conclusion:** Highest levels of inflammatory markers were detected in the polyarthritis group which corresponds with the poorer prognosis of this group. No relationship between the investigated polymorphisms and the type of treatment was found. A validation by further prospective studies on a larger patient cohort is needed.

**Disclosure of Interest**

None Declared.

**Table 28 (abstract P286).** See text for description

Diagnosis	Number of patients	MRP 8/14 μg/ml	IL-6 pg/ml	ESR mm/h	CRP mg/l	MTX, mg/m^2^	Patients treated with biologics	C677T	A1298C
Oligoarthritis onset	15	5.17	12.85	28.28	8.0	7.67	2	3 CC 2 CT	2 AA 1 AC 2 CC
Oligoarthritis relapse	27	3.87	9.91	17.18	9.38	7.74	14	4 CC 8 CT 1 TT	5 AA 7 AC
Polyarthritis onset	12	**9.71**	50.72	**35.83**	**31.67**	7.74	4	1 CC 1 CT 1 TT	2 AA 1 AC
Polyarthritis relapse	14	8.35	37.48	27.58	23.95	7.61	12	7 CC 3 CT	7 AA 3 AC
Systemic	6	3.35	**67.21**	22.6	14.52	7.65	5	1 CC 1 CT 1 TT	2 AA 1 AC
ERA	1	1.98	8.17	15	1.27	-	1	-	-

### P287 Arterial stiffness by oscillometric device and telomere lenght in juvenile idiopathic artrhitis: a cross-sectional study

#### Maria Mercedes C. Picarelli^1^, Luiz C. Danzmann^2^, Florencia Barbé-Tuana^3^, Lucas K. Grun^3^, Marcus H. Jones^4^

##### ^1^Rheumatology Service, Hospital São Lucas da PUCRS, Porto Alegre, Brazil; ^2^Internal Medicine, UNIVERSIDADE LUTERANA DO BRAZIL, Canoas, Brazil; ^3^Molecular biology, UNIVERSIDADE FEDERAL DO RIO GRANDE DO SUL, Porto Alegre, Brazil; ^4^Pediatrics, Hospital São Lucas da PUCRS, Porto Alegre, Brazil

###### **Presenting author:** Maria Mercedes C. Picarelli

**Introduction:** Introduction: Recent advances in juvenile idiopathic arthritis (AIJ) treatment promoted free disease survival. Cardiovascular disease (DCV) may emerge as an important cause of morbidity and mortality. Pulse wave velocity (VOP) and telomere length (TL) are considered as potential predictors of cardiovascular DCV. There are growing evidences of chronic and persistent inflammatory activity role on these processes. VOP and TL are potential early predictors of DCV and its outcomes.

**Objectives:** To asses pulse wave velocity (PWV) and telomere length (TL) in a Juvenile Idiopathic Arthritis (JIA) population with no cardiovascular disease risk factors, to compare them with a group of sex and age matched healthy controls and to test the correlation of those variables.

**Methods:** 24 JIA patients and 22 controls for TL and 20 controls for PWV were included. PWV was assessed by an oscillometric device. TL was assessed by quantitative proteinase chain reaction (qPCR). Inflammatory activity was assessed by Juvenile Arthritis Disease Activity Score (JADAS-27). Obesity, systemic hypertension, Diabetes and renal impairment and other inflammatory diseases were excluded.

**Results:** Oligoarticular (65, 2%) and polyarticular subtypes were included, with 73,9% females and 82,6% were Caucasian. The average age was 15,5 ± 6,3 years and the median disease duration was 9 [5-19] years. Between cases and controls for LTL there was significant difference in age no differences in sex, ethnics and BMI. The JADAS median was 8 [0, 00 – 20, 1], considered active when above 1, 0. PWV was normal in all patients, JIA and control (5.1 ± 0.20 m/s vs. 4.98 ± 0.06 m/s, *P* = 0, 66).

TL expressed by T/S ratio (amplification telomere product and single copy gene) was significantly reduced between JIA patients and controls (0. 85 ± 0, 34 vs. 1, 67 ± 1, 38, Mann-Whitney test *P =* 0.025). When age adjusted by ANCOVA, the difference remained significant (*P* = 0,032). There was no correlation between TL and age (*P* =0, 449, r = 0, 166), sex (*P* = 0, 521), disease duration (*P* =0, 358, r = -0, 318), JADAS (*P* = 0, 184, r = -0, 287) e VOP (*P* = 0, 843, r = 0, 044) in patients with AIJ.

**Conclusion:** TL was significantly shorter in long disease duration and high to moderate disease activity with no DCV risk factors AIJ patients and compared to controls.VOP was normal and no significant difference between controls were found. No correlations were observed between TL or VOP and disease duration, age and sex.

**Disclosure of Interest**

None Declared.

### P288 Discontinuation of biologics in juvenile idiopathic arthritis after reaching a clinical remission – single centre expirience

#### Marijan Frković, Karla Ištuk, Ika Birkić, Saša Sršen, Marija Jelušić

##### Division of Paediatric Rheumatology and Immunology, University Hospital Centre Zagreb, Zagreb, Croatia

###### **Presenting author:** Marijan Frković

**Introduction:** Biologics are well established in treatment of juvenile idiopathic arthritis (JIA). Despite their safety and efficacy, there are still lot of concerns regarding costs and possible risks of infections and malignancy. Although the time of biologics introduction is well defined in JIA therapy protocols, time of discontiunation, due to clinical inactive disease (CID), and risk of JIA relapse remain unclear.

**Objectives:** To determine the remission period and risk of relaps in JIA patients after discontinuation of biologics.

**Methods:** Retrospective chart review of JIA patients treated with biologics during 2010. – 2015. period at the Division of Paediatric Rheumatology and Immunology, University Hospital Centre Zagreb.

**Results:** Seventy-one JIA patients were treated with biologics (anti TNF, anti IL-6, anti IL-1) during 2010. – 2015. period. Discontinuation of therapy was recorded in 17 (23.9%) patients due to prolonged time of CID (average period 27 months). Mean time of follow up after therapy discontinuation was 22 months. Early relaps, during first 6 months, occured in 5/17 (29.4%) patients, while 1 more patient (5.8%) relapsed during second 6 months after discontinuation of biologics. Eleven patients (64.7%) remained in remission during follow up period.

**Conclusion:** Introduction of biologics is well defined in JIA therapy protocols. On the other hand, their discontinuation after achieving CID is still based on the experience and judgment of the clinicians. Development of appropriate guidelines for the biologics discontinuation in JIA is one of the essential goals.

**Disclosure of Interest**

None Declared.

### P289 Paediatric musculoskeletal matters - an e-resource with global reach; delivering essentials of knowledge and skills to raise awareness and facilitate recognition of rheumatic disease in children

#### Nicola Smith^1^, Sharmila Jandial^2^, Alan Easton^3^, Rachael Quarmby^4^, Raju Khubchandani^5^, Mercedes Chan^6^, Tim Rapley^7^, Helen Foster^1,2^

##### ^1^Musculoskeletal Research Group, Institute of Cellular Medicine, Newcastle University, Newcastle Upon Tyne, UK; ^2^Paediatric Rheumatology, Great North Children’s Hospital, Newcastle Upon Tyne, UK; ^3^BoxModel Digital Media, Newcastle Upon Tyne, UK; ^4^Everything Different, Newcastle Upon Tyne, UK; ^5^Department of Pediatrics, Jaslok Hospital and Research Center, Mumbai, India; ^6^Paediatric Rheumatology, University of Alberta, Edmonton, Canada; ^7^Institute of Health and Society, Newcastle University, Newcastle Upon Tyne, UK

###### **Presenting author:** Nicola Smith

**Introduction:** Delays in access to care are well reported in children with musculoskeletal (MSK) conditions [Foster & Rapley 2010].

**Objectives:** ‘paediatric musculoskeletal matters’ (PMM–www.pmmonline.org) was developed following user-engagement and peer review [Smith 2016] as a free resource targeting non-MSK specialists, with evidence-based content (medical students [Jandial 2014] and primary care [Goff 2014]) and endorsed by British Society for Paediatric and Adolescent Rheumatology (2014), Royal College Paediatrics and Child Health (2015) and National Institute Clinical Healthcare Excellence (2016). Here, we describe reach and uptake since launch of PMM (Nov-2014) and PMM India (Sept-2015).

**Methods:** Data (Google Analytics, e-surveys and 1-1 interviews with users) were analysed using mixed-methods. Ethically approved by Newcastle University.

**Results:** To date, PMM has reached 155 countries (>21,000 users, >100,000 hits). Where job details were provided (n = 243), most users (n = 231) were non-specialists in paediatric MSK (trainees n = 114). On average, users spend 4-minutes and access 4-pages per session. The most accessed pages are: clinical skills, normal development, normal variants and ‘red flags’. Smartphones are the preferred device with android more commonly used in Africa and India. User feedback supported further content to reflect other healthcare systems and signposting to aid learning. ‘PMM India’ highlights successful partnership to develop PMM with local context and further ‘internationalisation’ is planned with additional global partners.

**Conclusion:** Rapid globalization necessitates the development of culturally competent resources. PMM reflects collaborative partnership with reach that is expanding through ‘internationalisaton’ of content to further raise awareness of MSK problems towards improved health outcomes.

**Disclosure of Interest**

None Declared.

### P290 How treat-to-target approach influences short-term outcome of early JIA

#### Radoslav Srp^1^, Katerina Kobrova^1^, Jana Franova^2^, Sarka Fingerhutova^1^, Dana Nemcova^1^, Jozef Hoza^1^, Michal Uher^3^, Melania Saifridova^1^, Lenka Linkova^1^, Pavla Dolezalova^1^

##### ^1^Department of Paediatrics and Adolescent Medicine, General University Hospital and 1st Medical Faculty Charles University in Prague, Prague, Czech Republic; ^2^Department of Pediatrics, Masaryk University Brno and Faculty Hospital Brno, Brno, Czech Republic; ^3^Institute of Biostatistics and Analyses, Faculty of Medicine and the Faculty of Science of the Masaryk University, Brno, Czech Republic

###### **Presenting author:** Radoslav Srp

**Introduction:**

**Objectives:** We aimed to document how our daily clinical practice targeted towards rapid induction of remission affects short-term disease outcome of children with juvenile idiopathic arthritis (JIA).

**Methods:** Consecutive patients attending rheumatology clinics of our Unit were recruited. To become eligible they must have had a definitive diagnosis of JIA according to the ILAR criteria and exhibit active disease requiring either initiation of treatment for new-onset JIA or for the disease relapse. Active disease was defined by the presence of at least one joint with synovitis. Eligible patients were evaluated prospectively every 3 months for 1 year using standardized instruments for treatment response (American College of Rheumatology Pediatric (ACRPedi) response, Juvenile Arthritis Disease Activity Score (JADAS) 71, Clinically Inactive Disease (CID)) and adverse events (laboratory monitoring, Methotrexate Intolerance Severity Score (MISS)).

**Results:** Total of 104 patients fulfilled the study inclusion criteria during the 16-month recruitment period. Their median age at study entry was 8.3 years, 68 patients (65.4%) were girls. JIA ILAR onset subtypes were as follows: oligoarthritis (N = 50. 48%), seronegative polyarthritis (N = 38, 36.5%), systemic JIA with polyarthritis (N = 8, 7.7%), enthesopathic and psoriatic arthritis (N = 8, 7.7%). New onset disease was present in 57 patients (55%), 47 patients (45%) had disease relapse. Mean prior active disease duration was 2.3 months (SD 3.0). Median baseline JADAS was 10.9 (IQR 5.2; 17.0). High disease activity (JADAS ≥ 4.2 in oligoarthritis, ≥10.5 in polyarthritis) was present in 78 children (75%). Standard departmental treatment protocols were used in defined disease scenarios throughout the observational period. They have been based on the ACR 2011 recommendations. Medication at the start of the study included intraarticular triamcinolone hexacetonide (N = 51, 49%), methotrexate (N = 39, 38.6%), biologic therapy (etanercept, adalimumab, tocilizumab) (N = 14, 12.4%). In MTX therapy 82% of patients received parenteral MTX in the median weekly dose of 14.4 mg/m^2^. Disease state was assessed in month 6 and 12. Good clinical response, defined as reaching the minimum of ACR Pedi 70, was reached in 56.6% patients at month 6 and 70.7% patients at month 12. CID according to the JADAS score and to the Wallace criteria was reached in 39.5% and 35.5% of patients at month 6 and in 61.6% and 61.6% of patients at month 12, respectively. Damage index (JADI) evaluated only at month 12 was very low with the mean value of 0.5 (SD 2.39). In the MTX-treated group MTX intolerance defined as MISS ≥ 6 was present in 25.5% of patients at month 6 and in 30.6% of patients at month 12. Nevertheless, it led to the termination of MTX therapy in 2 patients only.

**Conclusion:** We have documented characteristics of a single-centre JIA patient cohort with new-onset or newly relapsing disease treated according to the recent recommendations and systematically followed using standardized disease outcome tools. Despite the expected heterogeneity of the group, the overall short-term outcome was very good with the treatment response of ACRpedi 70 in over 70% of patients and clinically inactive disease in more than 60% of patients after 1 year. Treatments were well tolerated and there were no serious adverse events noted. The sustainability of therapeutic response is currently being evaluated in an ongoing extension of the study.

**Acknowledgements:** The project was supported by the grant agency IGA MZ ČR NT14149-3/2013

**Disclosure of Interest**

R. Srp Grant / Research Support from: Pfizer, K. Kobrova: None Declared, J. Franova Grant / Research Support from: Abbvie, S. Fingerhutova Speaker Bureau of: Novartis, D. Nemcova Grant / Research Support from: Abbvie, Pfizer, J. Hoza: None Declared, M. Uher: None Declared, M. Saifridova: None Declared, L. Linkova: None Declared, P. Dolezalova Grant / Research Support from: Abbvie, Roche, Medac, Novartis, Pfizer, Consultant for: Roche, Speaker Bureau of: Pfizer, Novartis, Medac

### P291 Clinical features and early outcomes of Thai children with juvenile idiopathic arthritis

#### Sirirat Charuvanij, Isree Leelayuwattanakul

##### Department of Pediatrics, Siriraj Hospital, Mahidol University, Bangkok, Thailand

###### **Presenting author:** Sirirat Charuvanij

**Introduction:** There is currently a shortage of pediatric rheumatologists in Thailand and data relating to JIA in Thai children is very limited.

**Objectives:** To report baseline clinical features and early outcomes at 6 months following initial presentation in JIA patients who were treated at a newly established pediatric rheumatology clinic at Siriraj Hospital.

**Methods:** This retrospective descriptive study was conducted in patients who were diagnosed with JIA and followed for at least 6 months during the 2011 to 2015 study period**.** Data collected from a medical chart review included gender, ethnicity, age at disease onset, age at diagnosis, clinical presentations, subtype of JIA, and disease complications. Inactive disease was defined as clinical Juvenile Arthritis Disease Activity Score (cJADAS) < 1.

**Results:** Fifty-four JIA patients were enrolled in this study. Systemic JIA was the most common subtype (33.3%), followed by oligoarticular JIA (22%) and enthesitis-related arthritis (ERA) (22%). The most common presenting symptom was limping (64.8%), with knee joint being the most affected joint (64.8%). Laboratory findings showed anemia in 77.8% of patients, with plain radiograph revealing juxta-articular osteopenia in 40.7%. The medication most used among JIA patients was NSAIDs (81.5%). Only 2 patients received biologic agents within the 6-month evaluation period. Eighteen (33.3%) JIA patients were inactive after 6 months of treatment. Systemic JIA was the most common subtype in patients who developed inactive disease (61.6%). cJADAS decreased significantly from a median of 15 (IQR 11-20) at initial presentation to 4 (IQR 4-7) at the 6-month follow-up (*p* < 0.001). From initial presentation to 6-month follow-up, cJADAS was significantly decreased in the systemic JIA, ERA, oligoarticular JIA, and rheumatoid factor (RF) negative polyarticular JIA subtypes. Systemic JIA subtype showed significant improvement in active joint count (AJC), Physician Global Assessment of severity (PGA), patient/parent global assessment of well-being (PGW), and Childhood Health Assessment Questionnaire (CHAQ) scores. Median PGW score for RF+ polyarticular JIA patients showed the only score increase from initial presentation to 6-month follow-up.

**Conclusion:** One-third of JIA patients had inactive disease following 6 months of treatment. Inactive disease was mostly found in patients with systemic JIA.

**Trial registration identifying number:** N/A

**Disclosure of Interest**

None Declared.

### P292 Comparisons of the outcomes between early and late tocilizumab treatment in systemic juvenile idiopathic arthritis

#### Thita Pacharapakornpong^1^, Sakda A.-O. Vallipakorn^2^, Butsabong Lerkvaleekul^1^, Soamarat Vilaiyuk^1^

##### ^1^Pediatrics, Faculty of medicine Ramathibodi hospital, Mahidol university, Bangkok, Thailand; ^2^Section for clinical epidemiology and biostatistics, Faculty of medicine Ramathibodi hospital, Mahidol university, Bangkok, Thailand

###### **Presenting author:** Soamarat Vilaiyuk

**Introduction:** Around forty percent of systemic Juvenile idiopathic arthritis (SJIA) in Thailand had steroid dependency or failure to respond to conventional therapy, therefore tocilizumab (TCZ)*, a humanized anti-IL-6* receptor antibody, was indicated in these patients. However, due to financial problem, some patients could not receive TCZ treatment as soon as a failure of the conventional treatment occurred, leading to disability and poor quality of life. Therefore, this study focused on the outcomes between early and late TCZ treatment in SJIA patients.

**Objectives:** To compare the outcomes between early and late TCZ treatment in SJIA patients.

**Methods:** This is an observational cohort study. Thirty-three SJIA patients were included in this study. Baseline characteristics and disease severity were collected. Patients were divided into 2 groups; 1) early TCZ treatment, patients who received TCZ as first line therapy or received TCZ as soon as it was indicated, 2) late TCZ treatment, patients who receive TCZ later than 6 months after a failure of conventional treatment. Patients who received only nonsteroidal anti-inflammatory drugs (NSAIDs) and/or disease-modifying antirheumatic drugs (DMARDs) were included into the study as controls. The outcomes of this study were American College of Rheumatology Pediatric (ACR Pedi) 30, 50, 70 responses at 3, 6, 9, 12 month after treatment and remission rate at the end of this study. Descriptive analyses were conducted to determine the outcomes. This abstract is original and previously unpublished work.

**Results:** Thirty-three SJIA patients were included in this study. There were 12 males (37.5%) and 20 females (62.5%). Eleven patients were in early TCZ treatment group, 12 patients were in late TCZ group, and 9 patients were in conventional group as controls. In late TCZ treatment group, median of receiving TCZ was 17.0 months (IQR 8.3-22.8) after a failure of conventional treatment. At 12 months, patients in early TCZ treatment group, late TCZ treatment group, and conventional group achieved ACR Pedi 70 responses at 10 (91%), 6 (50%), 7 (77%), respectively. At the end of this study, early TCZ treatment group had highest remission rate (54.5%), whereas 22% of patients had remission in conventional treatment group and no patient in late TCZ treatment group achieved remission. For growth development, height SD scores in early TCZ treatment group were 0.6, -0.31, -0.25 and height SD scores in late TCZ treatment group were -1.35, -1.8, -2.4 at baseline, 1 year, and 2-year follow-up, respectively.

**Conclusion:** The outcomes of the TCZ treatment in SJIA patients depends on the time of receiving TCZ treatment. Growth impairment could not improve if delay in receiving TCZ. In early TCZ treatment, SJIA patients had better outcomes and more remission rate than late TCZ treatment and conventional therapy.

**Disclosure of Interest**

None Declared.

### P293 Relative contribution of the four components of the JADAS10 in determining the total score

#### Valentina Muratore, Gabriella Giancane, Stefano Lanni, Alessandra Alongi, Maria Giannina Alpigiani, Alberto Martini, Angelo Ravelli, Alessandro Consolaro

##### Pediatria II, Reumatologia, Istituto Giannina Gaslini, Genoa, Italy

###### **Presenting author:** Valentina Muratore

**Introduction:** The Juvenile Arthritis Disease Activity Score (JADAS) is widely used for the measurement of the level of disease activity in children with juvenile idiopathic arthritis (JIA). It is composed of the following four variables: 1) physician global assessment of disease activity (PGA); 2) parent global assessment of well-being (ParGA); 3) active joint count (AJC); 4) erythrocyte sedimentation rate (ESR). Although the four components assess different disease construct, their relative contribution in determining the total score is unclear. It has been argued that the tendency for parents to provide global ratings higher than those of physicians may artificially inflate the score. Furthermore, because acute phase reactants are frequently normal in JIA, their role may be less relevant than that of the other parameters.

**Objectives:** To examine the relative contribution of the four component of the JADAS10 in determining the total score.

**Methods:** The clinical information recorded during visits made in children with JIA from January 1987 to March 2012 was retrieved from the study center database. The JADAS10 score as well the score of each of its four components was computed for each visit. The score range of each component ranges from 0 to 10 and the total JADAS10 score ranges from 0 to 40. The following calculations were made: 1) number (%) of visits in which the score of each component was =0; 2) number (%) of visits in which the score of each component was the highest; 3) number (%) of visits in which the score of each component was =0 and the score of the other components >0; 4) number (%) of visits in which the score of each component was >0 and the score of the other components =0; 5) number (%) of visits in which the score of each component was 0-2.5, 2.5-5, 5-7.5, or >7.5; 6) Spearman’s correlation of the score of each component with the total score.

**Results:** A total of 2,553 visits made in 798 patients were identified. The score of PGA, ParGA, AJC and ESR was =0 in 28.3%, 40.4%, 22.2%, and 65.1% of visits, respectively, and was the highest in 32%, 22.3%, 22.9%, and 5.1% of visits, respectively. For PGA, ParGA, AJC and ESR, the percentage of visits in which the score of was =0 and the score of the other components was >0 was 0.3%, 4.3%, 0.8%, and 26.2%, respectively, and the percentage of visits in which the score was >0 and the score of the other components was =0 was 1%, 8.3%, 1.4%, and 1.5%, respectively. The Spearman’s correlation with the total score was 0.98, 1.0, 0.99, and 0.93 for PGA, ParGA, active joint count and ESR, respectively. The number (%) of visits in which the score of each component was 0-2.5, 2.5-5, 5-7.5 or > 7.5 is shown in the table.

**Conclusion:** PGA and NAJ are the variables that contribute mostly to the JADAS10 score. In our patients, we didn’t find evidence that the ParGA inflates the score. The ESR was =0 in around two third of visit, which suggests that omitting the acute phase reactant from the JADAS10 may not alter its performance.

**Disclosure of Interest**

None Declared.

**Table 29 (abstract P293).** See text for description

	Physician global assessment		Parent global assessment		ESR		Active joint count	
Score	N	%	N	%	N	%	N	%
0-2.5	395	49.5	501	62.8	656	82.2	488	61.2
2.5-5	11	1.4	165	20.7	104	13	159	19.9
5-7.5	129	16.2	94	11.8	30	3.8	47	5.9
7.5-10	140	17.5	38	4.8	8	1	104	13

## Poster Session: Macrophage activation syndrome

### P294 Distinct change patterns of laboratory biomarkers in macrophage activation syndrome complicating systemic juvenile idiopathic arthritis: a latent growth mixture model analysis

#### Alessandra Alongi^1,2^, Francesca Bovis^1^, Francesca Minoia^1^, Sergio Davì^1^, Alberto Martini^1^, Nicolino Ruperto^1^, Randy Q. Cron^3^, Angelo Ravelli^1^

##### ^1^Istituto G. Gaslini, Genova, Italy; ^2^Dipartimento Universitario Pro.Sa.M.I. “G. D’Alessandro”, Università degli Studi di Palermo, Palermo, Italy; ^3^Division of Pediatric Rheumatology, Children’s of Alabama, Birmingham, USA

###### **Presenting author:** Alessandra Alongi

**Introduction:** Macrophage activation syndrome (MAS) is a potentially life-threatening complication of systemic juvenile idiopathic arthritis (sJIA) characterized by heterogeneous organ involvement and severity. However, variables predicting outcome and treatment response have not been fully identified.

**Objectives:** To identify distinct patterns of change in laboratory markers in sJIA-related MAS and their correlation with clinical features and outcome using latent growth mixture modeling (LGMM), a data analytic approach that detects multiple unobserved clusters in heterogeneous populations.

**Methods:** Data from 362 patients diagnosed with MAS were analyzed with multiple-indicator latent growth curve modeling. Hemoglobin, whole blood cells, platelets, ERS, AST, bilirubin, fibrinogen, D-dimer, ferritin and creatinine values collected at three time points (last visit before MAS onset, MAS onset and full-blown MAS) were included in the unconditional model; the relationship between the obtained clusters and demographic features, clinical manifestations at onset and outcome were assessed via the 3-step method.

**Results:** LGMM revealed 5 clusters: patients with hyperferritinemia and hepatic involvement, even preceding MAS onset (class 1, n = 51); patients mainly characterized by elevation of inflammatory markers (class 2, n = 75); patients who developed hepatobiliary involvement and coagulopathy during MAS course (class 3, n = 56); patients who had severe pancytopenia, hepatobiliary and renal dysfunction and exhibited the earliest and greatest elevation of D-dimer and ferritin (class 4, n = 47); patients with milder alterations of white blood cells, platelets, fibrinogen, D-dimer and ferritin (class 5, n = 133). Being in a particular class was predictive of hemorrhagic, CNS, biliary, renal manifestations and organ failure (class-dependent conditional probabilities are shown in table). Classes characterized by hepatobiliary dysfunction (1, 3 and 4) were associated with severe course (admission to intensive care unit or death). Class 4 showed the worst outcomes and the highest prevalence of CNS and kidney involvement and multiorgan failure. Older age and longer duration of sJIA at MAS onset predicted significantly falling of patients in class 3 and 4.

**Conclusion:** Distinct patterns and course of laboratory abnormalities are related to clinical features and outcome in patients with sJIA-associated MAS. Our findings add to the understanding of heterogeneity in this condition and may help to identify clinical subsets characterized by different prognosis.

**Disclosure of Interest**

None Declared.

**Table 30 (abstract P294).** See text for description

Outcomes	Class 1	Class 2	Class 3	Class 4	Class 5	Wald	p-value
Hemorrhagic	-0.11	-0.83	0.31	1.01	-0.37	15.85	<0.01
CNS	0.05	-0.91	-0.20	1.29	-0.22	23.41	<0.01
Heart	0.08	-0.30	-0.09	0.61	-0.30	6.19	0.18
Lung	-0.19	-0.35	-0.21	0.84	-0.07	8.23	0.08
Kidney	-0.25	-0.65	0.02	1.61	-0.74	29.84	<0.01
Heart failure	-414.84	275.82	275.89	277.92	-414.80	1102538.15	<0.01
Lung failure	-552.83	137.95	137.44	139.75	137.68	17872.08	<0.01
Kidney failure	-553.23	137.80	138.19	139.71	137.51	952915.20	<0.01
Icu admission or death	0.47	-1.62	0.63	1.02	-0.51	30.74	<0.01

### P295 Next generation sequencing analysis of familial haemophagocytic lymphohistiocytosis (hlh) related genes in macrophage activation syndrome (MAS) and secondary HLH (SECHLH)

#### Chiara Passarelli^1^, Manuela Pardeo^2^, Elisa Pisaneschi^1^, Antonio Novelli^1^, Fabrizio De Benedetti^2^, Claudia Bracaglia^2^

##### ^1^Unit of Medical Genetics, Laboratory of Cytogenetics and Molecular Genetics, Roma, Italy; ^2^Division of Rheumatology, Ospedale Pediatrico Bambino Gesù IRCCS, Roma, Italy

###### **Presenting author:** Chiara Passarelli

**Introduction:** Macrophage activation syndrome (MAS) is a severe complication of rheumatic disease, particularly of systemic JIA (sJIA). It is currently classified among the secondary forms of HLH (secHLH). Primary HLH (pHLH) is caused by mutation of genes that code for proteins that are involved in cytotoxic functions. Mice carrying heterozygous mutations in more than 1pHLHgene carry a higher risk to develop HLH following viral infection, suggesting that accumulation of partial genetic defects may be relevant in HLH (1).

**Objectives:** To analyse, with next generation sequencing (NGS), genes involved in pHLH in MAS in the context of different rheumatic diseases and in secHLH.

**Methods:** We performed targeted resequencing on all patients using a panel including the 7 principal HLH-related genes (*PRF1, UNC13d, STX11, STXBP2, Rab27a, XIAP, SH2D1A*). Sequencing analysis were performed on the MiSeq® platform (Illumina, San Diego, CA); all variants identified were confirmed by Sanger. The possible functional impact of variants was studied by *in silico* analysis using SIFT and PolyPhen softwares. We took into account only variants with anallelic frequency in the global population <1%, in the dbSNP and Ensembl databases.

**Results:** We studied 25 patients, 20 MAS, 16 of whom developed this complication in the context of sJIA, and 4, in the context of other rheumatic diseases (vasculitis, Crohn’sdisease, systemic lupus erithematosus and Kawasaki disease, respectively) and 5 patients with sec-HLH. We identified at least 1 heterozygous variant in one of the pHLH associated genes in 14 (56%) patients: 10/20 MAS (50%), 4/5 secHLH (80%), with a detection rate of 56%. Nine patients showed variants in one gene, while variants in two or more genes were found in 5 patients. Five patients with MAS showed an heterozygous mutation in *PRF1* gene, the A91V variant was the most frequent (4), and 5 patients showed an heterozygous mutation in *UNC13d* gene. Three of the 20 MAS patients had mutations in two different genes two of them had recurrent episodes of MAS with one presenting a severe disease with a prolonged ICU admission. Three (60%) patients with sec-HLH showed an heterozygous mutation of *Rab27a*, 2 of *UNC13d* gene and 1 of *PRF1*. Two of those patients had mutations in two different genes, one of them presented three episodes of HLH reactivation and the other one presented a severe disease and died. Overall patients carrying mutations in 2 genes showed higher frequency of recurrences (4/5, 80%) compared to patients carrying one mutations or no mutation (6/20, 30%) and higher frequency of severe disease (i.e. needed admission to intensive care unit or died) (4/5, 80% versus 7/20, 35%).

**Conclusion:** Mutations of *PRF1* and *UNC13d* genes are frequently observed in patients with MAS or secondary HLH; Rab27a variants may be more frequent in patients with secHLH. Re-occurrence and severe disease tend to be more frequent and more severe in patients who carry mutations in two genes. These data are consistent with a polygenic model of secHLH and MAS.

**Reference**

1. Sepulveda FE et al. *Polygenic mutations in the cytotoxicity pathway increase susceptibility to develop HLH immunopathology in mice.* Blood 2016.

**Disclosure of Interest**

C. Passarelli: None Declared, M. Pardeo: None Declared, E. Pisaneschi: None Declared, A. Novelli: None Declared, F. De Benedetti Grant / Research Support from: Novartis, Novimmune, Hoffmann- La Roche, SOBI, AbbVie, Pfizer, C. Bracaglia: None Declared.

### P296 IFNGAMMA, IFNGAMMA related chemokines and other biomarkers in macrophage activation syndrome (MAS)

#### Claudia Bracaglia^1^, Denise Pires Marafon^1^, Ivan Caiello^1^, Kathy de Graaf^2^, Florence Guilhot^2^, Walter Ferlin^2^, Sergio Davi'^3^, Grant Schulert^4^, Angelo Ravelli^3^, Alexi A. Grom^4^, Robert Nelson^2^, Cristina de Min^2^, Fabrizio De Benedetti^1^

##### ^1^Division of Rheumatology, Ospedale Pediatrico Bambino Gesù IRCCS, Roma, Italy; ^2^SA Novimmune, Geneva, Switzerland; ^3^University of Genova, Istituto Giannina Gaslini, Genova, Italy; ^4^Division of Pediatric Rheumatology, Cincinnati Children’s Hospital Medical Center, Ohio, USA

###### **Presenting author:** Claudia Bracaglia

**Introduction:** Evidence in animals and humans points to a pivotal role of IFNγ in primary HLH. We have recently generated data in an animal model supporting a role for IFNγ also in MAS and reported high levels of IFNγ and of the IFNγ-related chemokines, CXCL9, CXCL10 in patients with MAS, during systemic JIA (sJIA).

**Objectives:** To evaluate levels of sCD25, IL-18 and neopterin, as well as levels of IFNγ of CXCL9 and CXCL10, and their correlation with laboratory parameters of MAS severity in patients with active sJIA with or without MAS at sampling.

**Methods:** Circulating levels of biomarkers were measured by Luminex assay in 57 samples obtained, from 24 patients with active sJIA and in 37 samples from 20 patients with MAS at sampling at variable severity and treatments.

**Results:** Levels of IFNγ, CXCL9, CXCL10, sCD25, IL-18 and neopterin were significantly elevated in MAS compared to active sJIA without MAS at sampling (p-values <0.0001, except for IL-18 p = 0.012). In patients with MAS, but not in patients with active sJIA without MAS at sampling, laboratory parameters of disease severity were significantly correlated with IFNγ, CXCL9, CXCL10, sCD25 and neopterin (Table 31). No correlation with IL-18 was found (IL-18 levels were available only for a portion of the samples). Interestingly, during active sJIA without MAS at sampling, levels of CXCL9 (median 3889,IQR 965-7142), CXCL10 (764,323-1259) and IL-18 (4405,582-7122) were significantly higher in patients with a history of MAS compared to those of patients without a history of MAS (519,IQR 385-1168; 215,IQR 152-470; 439,312-824, respectively).

**Conclusion:** Levels of IFNγ, CXCL9, CXCL10, sCD25, and neopterin were higher during MAS and correlated with laboratory parameters of severity. IL-18 levels were not correlated with laboratory parameters of MAS severity and the observation that IL-18 levels are higher in patients with a history of MAS is consistent with the hypothesis that high levels of IL-18 may contribute to the predisposition to MAS in sJIA as also suggested by Shimizu et al (1). Elevation of sCD25 is consistent with the presence of T cell activation in MAS. Elevation of neopterin and CXCL9, both of which reflects IFNγ production, and their correlation with laboratory parameters, supports the pathogenic role of IFNγ in MAS. Given the fact that circulating CXCL9 levels appear to reflect tissue IFNγ production, presence of high CXCL9 in patients with a history of MAS, but without MAS at sampling, suggests subclinical activation of the IFNγ pathway in these patients.

**Reference**

1. Shimizu M. Interleukin-18 for predicting the development of macrophage activation syndrome in systemic juvenile idiopathic arthritis. Clin immunol 2015

**Disclosure of Interest**

C. Bracaglia: None Declared, D. Pires Marafon: None Declared, I. Caiello: None Declared, K. de Graaf Employee of: Novimmune, F. Guilhot Employee of: Novimmune, W. Ferlin Employee of: Novimmune, S. Davi': None Declared, G. Schulert: None Declared, A. Ravelli: None Declared, A. Grom Grant / Research Support from: NovImmune, Novartis Pharmaceutical Corporation, Roche Pharmaceuticals, R. Nelson Employee of: Novimmune, C. de Min Employee of: Novimmune, F. De Benedetti Grant / Research Support from: Novartis, Novimmune, Hoffmann- La Roche, SOBI, AbbVie, Pfizer.

**Table 31 (abstract P296).** Correlation between cytokines and laboratory parameters in active MAS (N = 37).

	IFNγ	CXCL9	CXCL10	sCD25	IL-18	Neopterin
	*p* (r)	*p* (r)	*p* (r)	*p* (r)	*p* (r)	*p* (r)
Ferritin (ng/mL)	0.014(0.46)	0.034(0.43)	0.003(0.54)	0.002(0.63)	>0.1(0.35)	>0.1(0.39)
White blood cells (x10^^9^/L)	0.033(-0.47)	0.013(-0.56)	>0.1(-0.32)	>0.1(-0.42)	>0.1(-0.40)	>0.1(0.04)
Platelet (x10^^9^/L)	0.001(-0.55)	0.0001(-0.66)	<0.0001(-0.66)	0.003(-0.57)	>0.1(-0.14)	>0.1(-0.35)
Fibrinogen (mg/dL)	>0.1(-0.32)	0.0014(-0.73)	0.008(-0.60)	0.0005(-0.74)	>0.1(-0.38)	0.008(-0.61)
Triglycerids (mg/dL)	>0.1 (0.20)	>0.1 (0.23)	>0.1(0.13)	>0.1(0.29)	>0.1 (0.02)	>0.1 (0.17)
LDH (U/L)	0.005(0.64)	<0.0001(0.90)	<0.0001(0.94)	0.002(0.69)	>0.1 (-0.23)	0.0001 (0.85)
ALT (U/L)	0.012 (0.52)	0.0007 (0.68)	0.0005 (0.67)	0.008 (0.54)	>0.1 (0.38)	>0.1 (0.14)

Values are expressed as p value (r di Spearman).

### P297 S100A12 as diagnostic tool in the differential diagnosis of SJIA associated MAS vs. primary or acquired HLH

#### Dirk Holzinger^1^, Christoph Kessel^2^, Ndate Fall^3^, Alexei Grom^3^, Wilco de Jager^4^, Sebastiaan Vastert^5^, Raffaele Strippoli^6^, Claudia Bracaglia^7^, Erik Sundberg^8^, Anna Horne^9^, Stephan Ehl^10^, Sandra Ammann^11^, Kai Lehmberg^12^, Fabrizio De Benedetti^13^, Karin Beutel^14^, Dirk Foell^2^

##### ^1^Department of Pediatric Rheumatology and Immunology, University Children’s Hospital Muenster, Muenster, Germany; ^2^Department of Pediatric Rheumatology and Immunology, University Children’s Hospital Muenster, Muenster, Germany; ^3^Divisions of Rheumatology, Cincinnati Children’s Hospital Medical Center, Cincinnati, USA; ^4^Laboratory of Translational Immunolgy, Utrecht, Netherlands; ^5^Department of Pediatric Rheumatology and Immunology, University Medical Center Utrecht, Utrecht, Netherlands; ^6^Department of Cellular Biotechnology and Hematology, Sapienza University of Rome, Rome, Italy; ^7^UO Reumatologia, IRCCS Ospedale Pediatrico Bambino Gesù, Rome, Italy; ^8^Department of Women´s and Children`s Health, Karolinska Institutet, Stockholm, Sweden; ^9^Childhood Cancer Research Unit, Karolinska University Hospital Solna, Stockholm, Sweden; ^10^Center for Chronic Immunodeficiency, University Hospital Freiburg, Freiburg, Germany; ^11^Center for Chronic Immunodeficiency, University Hospital Freiburg, Freiburg, Germany; ^12^Department of Pediatric Hematology and Oncology, University Medical Center Hamburg Eppendorf, Hamburg, Germany; ^13^UO Reumatologia, RCCS Ospedale Pediatrico Bambino Gesù, Rome, Italy; ^14^Department of Pediatric Hematology and Oncology, Children’s Hospital, Technische Universität München, Munich, Germany

###### **Presenting author:** Dirk Holzinger

**Introduction:** Macrophage activation syndrome (MAS) is a severe complication of autoimmune and autoinflammatory disease. MAS is most strongly associated with systemic juvenile idiopathic arthritis (SJIA) but can also be seen in Kawasaki disease, SLE or IBD. Clinically, MAS is strikingly similar to hemophagocytic lymphohistiocytosis (HLH) and the initial differentiation between SJIA-associated MAS and primary HLH or acquired HLH is difficult. Due to recent advances in the description of HLH-related gene defects, most patients with primary HLH can be identified through genetic or functional analysis. Unfortunately these investigations are not always easily available. Since viral infections such as EBV and CMV can trigger both primary and acquired forms of HLH, the presence of a viral trigger in a patient with HLH does not necessarily allow classification of the disease as acquired HLH. S100A12 is an endogenous TLR4 ligand that induces monocyte activation, thereby acting as an amplifier of innate immunity during early inflammation. S100A12 is highly overexpressed in SJIA, and the assessment of S100A12 serum levels helps distinguish SJIA from other febrile illnesses.

**Objectives:** The main goal of this study was to determine whether S100A12 might help distinguish SJIA-associated MAS from HLH.

**Methods:** S100A12 serum levels were assessed in 177 samples obtained from 114 unique patients using the in-house ELISA kit. Of 177 samples, 152 samples were also available for a multiplex immunoassay including 53 cytokines and chemokines. Serum samples were obtained from 9 healthy controls, sJIA patients without MAS (17 active/ 19 remission), SJIA patients with MAS (33 active/ 33 remission), acquired HLH (22 active/ 20 remission) and 33 patients with primary HLH at disease onset. Additional data obtained at the time of serum collection included clinical features, conventional laboratory markers (CRP, ESR, differential blood count, fibrinogen, ferritin, triglycerides) and when available, NK cell function test results and sCD25 levels.

**Results:** Serum levels of S100A12 could differentiate patients with primary or acquired HLH from patients with SJIA-associated MAS. Levels of S100A12 > 1400 ng/ml were only seen in patients with active SJIA, either with MAS (mean ± SD 5470 ± 3042 ng/ml) or without MAS (4150 ± 3251 ng/ml), but not in patients with acquired (451 ± 351 ng/ml) or primary HLH (216 ± 170 ng/ml). Healthy controls were in a lower range (85 ± 44 ng/ml). Although S100A12 levels correlated closely with disease activity in SJIA patients (as determined by JIA core set criteria), no significant difference between SJIA patients with or without MAS was seen.

**Conclusion:** S100A12 serum levels are useful to differentiate between SJIA-associated MAS and inherited or acquired HLH. The combination with conventional laboratory markers, serum cytokine profiles and clinical characteristics might allow creating a diagnostic panel for the differentiation of MAS vs. HLH. Since at the onset of disease SJIA MAS and acquired HLH are difficult to discriminate this might be a helpful diagnostic tool.

**Disclosure of Interest**

None Declared.

### P298 Development and initial validation of the “MH score”, a new diagnostic tool that differentiates primary hemophagocytic lymphohistiocytosis from macrophage activation syndrome

#### Francesca Minoia^1^, AnnaCarin Horne^2^, Francesca Bovis^1^, Sergio Davì^1^, Laura Pagani^1^, Graciela Espada^3^, Yi-jin Gao^4^, Antonella Insalaco^5^, Kai Lehmberg^6^, Helga Sanner^7^, Susan Shenoi^8^, Sheila Weitzman^9^, Nicolino Ruperto^1^, Alberto Martini^1^, Randy Q. Cron^10^, Angelo Ravelli^1^

##### ^1^Istituto G. Gaslini, Genoa, Italy; ^2^Karolinska University Hospital, Stockholm, Sweden; ^3^Hospital de Ninos Ricardo Gutierrez, Buenos Aires, Argentina; ^4^Children’s Hospital of Fudan, Shanghai, China; ^5^Ospedale Pediatrico Bambino Gesù, Rome, Italy; ^6^University Medical Center, Hamburg, Germany; ^7^Rikshospitalet, Oslo, Norway; ^8^Seattle Children's Hospital, Seattle, WA, USA; ^9^Hospital for Sick Children, Toronto, Canada; ^10^University of Alabama, Birmingham, AL, USA

###### **Presenting author:** Francesca Minoia

**Introduction:** It is common view that macrophage activation syndrome (MAS) bears close similarities with primary hemophagocytic lymphohistiocytosis (pHLH). The resemblance of their clinical and laboratory manifestations may make it difficult to differentiate the two conditions, particularly when pHLH occurs at a later age or MAS develops at onset of systemic juvenile idiopathic arthritis (sJIA), when arthritis is not yet present. However, early discrimination is important because pHLH is often more severe than MAS and the therapeutic approaches are different

**Objectives:** To develop and validate a diagnostic score that discriminates pHLH from sJIA-associated MAS

**Methods:** The clinical, laboratory and histopathologic features of 362 patients with sJIA-associated MAS and of 258 patients with pHLH were collected in a multinational collaborative project involving pediatric rheumatologists and pediatric hematologists. 80% of the study population was used to develop the score and the remaining 20% constituted the validation sample. The features with the strongest association with pHLH in univariate analyses (odds ratio > 5) were further scrutinized in multivariate logistic regression procedures. Each variable that entered the best fitting model was then assigned a score, based on its statistical weight. The MH score was made up with the individual scores of the selected variables. The cut-off in the MH score that discriminated best pHLH from MAS was calculated by means of ROC curve analysis. The sensitivity (SE), specificity (SP), area under the curve (AUC) and kappa value of the MH score were calculated for both the developmental and validation samples

**Results:** The following 6 variables entered the best fitted model of logistic regression analysis, that is, were most closely associated with a diagnosis of pHLH: age at disease onset ≤ 1.6 years, neutrophil count ≤ 1400/μl, fibrinogen ≤ 131 mg/dl, splenomegaly, platelet count ≤ 78000/μl, hemoglobin ≤ 8.3 g/dl. The MH score ranged from 0 to 123. Its median value was 97 (IQR 75-123) in pHLH patients and 12 (IQR 11-34) in MAS patients. The probability of a diagnosis of pHLH ranged from < 1% for a score < 11 to > 99% for a score ≥ 123. A cut-off value > 59 revealed the best performance in discriminating pHLH from MAS (SE = 91%, SP = 93%, AUC = 0.92, kappa = 0.85). The strong diagnostic power of the MH score was confirmed in the validation sample

**Conclusion:** The MH score is a powerful tool that facilitates timely discrimination of pHLH from MAS. Its application in routine clinical care may aid practitioners to identify those patients who are more likely to have pHLH and may, thus, deserve diagnostic confirmation with appropriate genetic and functional testing

**Disclosure of Interest**

None Declared.

### P299 Neutralization of interferon-gamma is efficacious in a mouse model of macrophage activation syndrome

#### Giusi Prencipe^1^, Ivan Caiello^1^, Antonia Pascarella^1^, Claudia Bracaglia^1^, Walter G. Ferlin^2^, Laurence Chatel^2^, Raffaele Strippoli^3^, Cristina de Min^2^, Fabrizio De Benedetti^1^

##### ^1^Division of Rheumatology, Bambino Gesù Children's Hospital, Rome, Italy; ^2^NovImmune SA, Geneva, Switzerland; ^3^Department of Cellular Biotechnologies and Haematology, Sapienza University of Rome, Rome, Italy

###### **Presenting author:** Giusi Prencipe

**Introduction:** Macrophage activation syndrome (MAS) is a term used to identify hemophagocytic lymphohistiocytosis (HLH) secondary to rheumatic diseases. It is a severe and potentially fatal condition that occurs in the context of rheumatic diseases, particularly systemic JIA. It is part of secondary HLH forms. While the triggering mechanism behind pHLH is the defect in cytotoxicity, caused by mutations in genes encoding proteins required for lymphocyte and natural killer cell activity, MAS pathogenesis is not clearly understood. Our recent data on patients with MAS showed that levels of interferon-gamma (IFNγ) and of IFNγ-induced chemokines are markedly increased and are correlated with the severity of laboratory abnormalities of MAS (Bracaglia, 2016).

**Objectives:** Based on our results in patients and on data obtained in animal models of pHLH, showing that IFNγ neutralization reverts the disease, in this study, we aim to demonstrate the role of IFNγ and the efficacy of a treatment with an anti-IFNγ antibody in a murine model of MAS.

**Methods:** A mouse model of MAS (Strippoli R, 2012), relying on an exaggerated response to toll-like receptor ligands in mice transgenic for the pro-inflammatory cytokine interleukin-6 (IL-6TG), has been used to evaluate the activation of the IFNγ pathway and the therapeutic potential of a rat neutralizing IFNγ antibody (XMG1.2, BioXcell, USA).

**Results:** LPS-challenged IL-6TG mice showed significantly higher mRNA levels of *Infγ* and higher phospho-STAT1 (Tyr701) protein levels in liver and spleen compared to LPS-challenged wild type (WT) mice. Accordingly, in LPS-challenged IL-6TG mice significantly higher mRNA expression levels of IFNγ-induced chemokines, such as *Cxcl9* and *Cxcl10,* were observed both in liver and in spleen. The up-regulation in the IFNγ pathway in peripheral tissues resulted in significantly higher circulating levels of CXCL9 and CXCL10 in LPS-challenged IL-6TG mice compared to LPS-challenged WT mice. IFNγ neutralization studies revealed that, in LPS-challenged IL-6TG mice, anti-IFNγ treatment significantly improved survival (p = 0.003) and body weight recovery (p = 0.015), compared to control antibody-treated animals. Furthermore, a significant amelioration in laboratory parameters of MAS, including reduction in ferritin levels and increase in fibrinogen levels, was observed in mice treated with anti-IFNγ Ab. Finally, in IL-6TG mice treated with the anti-IFNγ antibody circulating levels of CXCL9, CXCL10 and, notably, of pro-inflammatory cytokines were significantly reduced. The trend in circulating CXCL9 and CXCL10 levels significantly paralleled the circulating levels of pro-inflammatory cytokine, as well as the serum ferritin levels (Spearman r = 0.83, p = 0.0008 and Spearman r = 0.76, p = 0.003, respectively). This association of ferritin levels with circulating levels of CXCL9 and CXCL10 was confirmed also in MAS patients followed longitudinally.

**Conclusion:** These results provide insights into the pathophysiology of MAS, further support the hypothesis that IFNγ is the unique and common mediator of all HLH forms and provide the rationale for a targeted therapy against IFNγ in MAS.

**Disclosure of Interest**

None Declared.

### P300 Innovative use of PK and PD to guide dose selection for a monoclonal antibody aimed at neutralizing the high ifn-gamma activity present in patients with macrophage activation syndrome (MAS)

#### Philippe Jacqmin^1^, Kathy De Graaf^2^, Maria Ballabio^2^, Robert Nelson^2^, Zoë Johnson^2^, Walter Ferlin^2^, Geneviève Lapeyre^2^, Fabrizio de Benedetti^3^, de Min Cristina^2^

##### ^1^MnS, Dinant, Belgium; ^2^NovImmune S.A., Geneva, Switzerland; ^3^Division of Rheumatology, Ospedale Pediatrico Bambino Gesù IRCCS, Roma, Italy

###### **Presenting author:** de Min Cristina

**Introduction:** Data from an animal model of MAS and the observed high IFNγ and IFNγ-related chemokines (CXCL9, CXCL10) levels in MAS/sJIA patients have prompted the design of a study to investigate the therapeutic role of IFNγ neutralization in patients with this disease. An ongoing study in primary HLH (pHLH) shows promising efficacy and favourable safety of NI-0501, an anti-IFNγ antibody, in the control of HLH, known to be driven by high production of IFNγ.

**Objectives:** To describe the use of the PK and PD data of NI-0501 obtained from the ongoing clinical trial in pHLH, to define the NI-0501 dosing strategy to be used to investigate the role of IFNγ neutralization in MAS/sJIA.

**Methods:** In active HLH the measurable circulating IFNγ levels do not account for the total amount of the cytokine present in the body. Following the administration of NI-0501 in pHLH patients, the measurement of “total IFNγ”, namely free and bound to NI-0501, is used as a surrogate for IFNγ production, revealing the high production of this cytokine, despite the relatively low “free IFNγ” levels at baseline in blood. Extrapolations from these data allowed estimation of IFNγ production in MAS/sJIA patients, based on the levels of IFNγ-related chemokines present at baseline.

**Results:** The measurement of total IFNγ levels in pHLH revealed that the IFNγ concentration to be neutralized by NI-0501 was several hundreds fold higher compared to what indicated by the baseline free IFNγ level (median IFNγ at baseline <50 pg/ml; at peak 17’858 pg/ml). Total IFNγ at 48 hours post NI-0501 administration tightly correlates with IFNγ-related chemokines levels (CXCL9: r = 0.6264, p = 0.0008; CXCL10: r = 0.6931, p = 0.0001), suggesting that CXCL9 and CXCL10 concentrations are an excellent marker of the presence of biologically active IFNγ. The amount of IFNγ produced in MAS/sJIA patients was then indirectly estimated on the basis of the total IFNγ concentration in pHLH patients with comparable levels of CXCL9 and CXCL10 following the administration of NI-0501. This information, coupled with modelling and simulation techniques, has allowed to i) determine the NI-0501 dose expected to neutralize rapidly the total amount of IFNγ in the majority of MAS/sJIA patients, ii) identify an appropriate frequency of NI-0501 administration to avoid unnecessary drug accumulation.

**Conclusion:** The methodology applied allowed a precise determination of the dosing strategy to be tested in the future trial, significantly reducing the risk of exposing patients to non-therapeutic NI-0501 doses.

**Disclosure of Interest**

P. Jacqmin Consultant for: Novimmune S.A., K. De Graaf Consultant for: Novimmune S.A., M. Ballabio Employee of: Novimmune S.A., R. Nelson Employee of: Novimmune S.A., Z. Johnson: None Declared, W. Ferlin Employee of: Novimmune S.A., G. Lapeyre Employee of: Novimmune S.A., F. de Benedetti: None Declared, D. M. Cristina Employee of: Novimmune S.A.

### P301 High-dose liposteroid therapy in a patient with juvenile dermatomyositis-associated macrophage activation syndrome

#### Hiroyuki Wakiguchi, Shunji Hasegawa, Reiji Hirano, Fumiko Okazaki, Tamaki Nakamura, Hidenobu Kaneyasu, Shouichi Ohga

##### Department of Pediatrics, Yamaguchi University Graduate School of Medicine, Ube, Japan

###### **Presenting author:** Hiroyuki Wakiguchi

**Introduction:** Macrophage activation syndrome (MAS) is a rare and life-threatening complication of rheumatologic diseases of childhood including systemic-onset juvenile idiopathic arthritis, systemic lupus erythematosus, and Kawasaki disease. Liposteroid, a lipid emulsion containing dexamethasone, was developed in Japan. It has been used for the control of rheumatoid arthritis, hemophagocytic lymphohistiocytosis, graft-versus-host disease, and pulmonary hemosiderosis. This agent might pose greater efficacy and much less risk for systemic adverse effects than dexamethasone, because the lipid emulsions are easily taken up by phagocytes. There are no reports about the clinical utility of liposteroid in juvenile dermatomyositis (JDM)-associated MAS (JDM-MAS).

**Objectives:** To present the case report of liposteroid therapy in a patient with JDM-MAS.

**Methods:** We have observed a patient suffering from JDM-MAS.

**Results:** A 4-year-old girl with JDM was hospitalized for fever, erythema, hepatosplenomegaly, cytopenias, liver dysfunction and coagulopathy. Platelet counts were decreased. Serum ferritin and urinary β_2_-microglobulin levels were elevated. Bone marrow aspiration revealed a large number of hemophagocytosing macrophages. She was diagnosed as having JDM-MAS. Intravenous infusion of high-dose liposteroid and cyclosporine-A led to a drastic improvement of JDM-MAS. No serious adverse events were observed during the liposteroid therapy.

**Conclusion:** The first report of successful liposteroid therapy for JDM-MAS suggested that high-dose liposteroid therapy is one of the treatment options of pediatric MAS.

**Disclosure of Interest**

None Declared.

### P302 Unique serum cytokine profiles in systemic JIA complicated with macrophage activation syndrome

#### Kazuko Yamazaki^1,2^, Tomo Nozawa^1^, Taichi Kanetaka^1^, Shuichi Ito^1^, Shumpei Yokota^1^

##### ^1^Pediatrics, Yokohama City University School of Medicine, Japan, Yokohama; ^2^Pediatrics, Saitama MedicalCenter, Saitama Medical University, Kawagoe, Japan

###### **Presenting author:** Kazuko Yamazaki

**Introduction:** Macrophage activation syndrome (MAS) is a severe and life threatening complication of systemic juvenile idiopathic arthritis (sJIA). Diagnosis of MAS in sJIA is difficult to distinguish from a flare of sJIA or sepsis. Since vascular endotherial and tissue damage are worsen by the hour in MAS, early diagnosis is crucial and treatment should be promptly initiated. The assessment of changes in laboratory data that indicates tissue damage, hemophagocytic activity, endotherial damage and hyper coagulation-fibrinolysis system is necessary. MAS is caused by excessive activation of T cells and macrophages, leading to production of massive pro-inflammatory cytokines, hemophagocytic activity and massive inflammatory responses. The disregulation of the augmentation or degradation of inflammation is considered to be related to the pathology of MAS.

**Objectives:** - To present a case of 2 year old girl diagnosed and treated for sJIA complicated with MAS

-To assess changes over time of the laboratory data and serum cytokine and chemokine profiles of Interleukin(IL)-6, IL-18, Interferon(IFN)-γ, IFN-γ inducible protein (IP)-10/CXCL10, and serum High mobility group box (HMGB)-1 concentration.

**Methods:** We retrospectively reviewed the case record and laboratory findings. IL-6, IL-18, IFN-γ, IP-10 and HMGB-1 of frozen serums were measured by ELISA.

**Results:** A 2 year-old girl presented with 5 days of fever, rash, poor appetite, hip and knee joint pain and cervical lymphadenopathy. Blood investigations had shown neutrophilia, thrombocytosis, and raised inflammatory markers (CRP, ESR, Ferritin). She was initially diagnosed with Kawasaki disease and was treated with IV Immunoglobulins. Because she continuously presented remittent fever, she was treated with high dose corticosteroids as sJIA. The 18 days after the onset, her laboratory data showed anemia, low platelet count, significantly elevated LDH (2,075 IU/L) and ferritin (33,359 ng/dL), and high AST, triglycerides and sIL-2R. She was transferred to our hospital. In the view of remittent fever, typical rash, lymphadenopathy, splenomegaly, arthritis, we diagnosed her as sJIA complicated with MAS and started treatment with dexamethasone palmitate and cyclosporine which led her recovery from MAS. Because the reduction of corticosteroids doses was difficult, she was administered tocilizumab. After 5 months of first episode of MAS, fever, and elevated LDH and ferritin were observed again. We initiated treatment for Beginning of MAS. One month after second episode of MAS, her blood cell count and laboratory inflammation markers were back to normal.

Next, we investigated changes of her serum cytokines, chemokine, and HMGB-1 over time in the course of treatment. The levels of serum IL-6 and IL-18 were extremely high at the onset of the sJIA, and a significant level of serum IL-18 was persistent. Although concentrations of IFN-γ and IP-10 were low at the time of remittent fever, these levels were remarkably elevated at a few days before the diagnosis of MAS, then decreased to undetectable levels at the recovery from MAS. On the other hand, the level of HMGB-1was increased at the time of remittent fever, and decreased during MAS.

**Conclusion:** A Patient with active sJIA complicated with MAS shows distinct serum cytokine profiles. High levels of serum IL-6 and IL-18, and low level of IFN-γ were found at the baseline of active s JIA. Significant increase of serum IFN-γ and its downstream proteins are considered to indicate the beginning of MAS in systemic JIA.

**Disclosure of Interest**

None Declared.

### P303 Macrophage activation syndrome- a case series

#### Kirsty McLellan, Ishbel MacGregor, Neil Martin, Joyce Davidson

##### Royal Hospital for Children, Glasgow, UK

###### **Presenting author:** Kirsty McLellan

**Introduction:** Macrophage activation syndrome (MAS) is a serious and potentially fatal complication of paediatric rheumatological disorders, most commonly seen in systemic onset juvenile idiopathic arthritis (SoJIA). Triggered by infection, modifications in treatment, disease onset or exacerbation, MAS is thought to be caused by a failure of cytotoxic cells to cause T lymphocyte and macrophage apoptosis, resulting in an uncontrolled, proinflammatory ‘cytokine storm’. There is often diagnostic uncertainty as MAS presents with a similar phenotype to sepsis and exacerbation of the underlying inflammatory condition, and the diagnosis is even more difficult to make in those who present with MAS at initial presentation, without an underlying disorder. There are several published diagnostic and classification criteria to aid the clinical diagnosis of MAS; the Haemophagocytic Lymphohistiocytosis (HLH) guidelines^1^ initially developed for the diagnosis of genetically inherited primary HLH, Ravelli et al 2005 criteria^2^ and the subsequent Ravelli et al 2016 Classification criteria^3^ for the diagnosis of MAS in children with SoJIA and the Parodi et al criteria^4^ for the diagnosis of MAS in systemic lupus erythromatosus (SLE).

**Objectives:** To describe all the cases of MAS presenting to a paediatric rheumatology clinical network over a 9 year period, specifically investigating underlying diagnosis, if the episode of MAS was part of the initial presentation or later in the disease course and the applicability of the current published diagnostic and classification criteria.

**Methods:** Retrospective case note review.

**Results:** 19 cases of MAS were identified. 9 were male and 10 female, with an age range of 13 months to 16 years, mean age 10.1 years. All cases had a peak ferritin level of >1070 μg/l. The most common underlying diagnosis was SoJIA in 11, followed by SLE in 4, 1 polyarteritis nodosa, 1 juvenile dermatomyositis, 1 reactive and 1 an immune deficiency. 13 cases of MAS occurred during the initial presenting illness. Only 6 children were known to have an underlying rheumatological diagnosis prior to their MAS episode. Of those 6, 3 had their underlying diagnosis changed during the episode of MAS, 1 from psoriatic arthritis to SoJIA, 1 from polyarticular JIA to SLE and 1 from polyarticular JIA to an underlying MHC class II deficiency. 3 children in the cohort died; 1 directly from MAS, the other 2 from complications of the underlying disorder. Applying the existing diagnostic criteria to our cases; 5 of the 19 cases of MAS fulfilled the HLH criteria^1^, 9/11 of the cases with a final underlying diagnosis of SoJIA fulfilled the original Ravelli et al 2005 criteria^2^, 5/11 fulfilled the latest Ravelli 2016 criteria^3^ and all 4 children with SLE fulfilled the Parodi criteria^4^ for MAS in association with SLE.

**Conclusion:** 68% of the cases of MAS in our series presented without an underlying diagnosis. As MAS is difficult to diagnose in children with underlying rheumatological disorders, this difficulty is heightened in children without an underlying disease whose clinical and laboratory features cannot be applied to the existing diagnostic criteria, all developed for use with known underlying conditions. It is essential that awareness is raised of this serious disorder amongst general paediatricians to avoid delays in diagnosis and treatment which could potentially increase morbidity and mortality. There is a need for the development of diagnostic criteria which can be used in cases where the episode of MAS is part of the initial presentation of the underlying condition. In our series, the majority of cases of MAS present in this way.

**References**

1. Henter et al. Paediatric Blood Cancer 2007;48:124-131

2. Ravelli et al. J Pediatr 2005;146:598-604

3. Ravelli et al. Arthritis&Rheumatology 2016;68:566-576

4. Parodi et al. Arthritis&Rheumatology 2009;60:3388-3399

**Disclosure of Interest**

None Declared.

## Poster Session: Genetics and environment

### P304 Recommendations for European paediatric research – a share initiative

#### Jasmin Kuemmerle-Deschner^1^, Sandra Hansmann^2^, Nico Wulffraat^3^, Andreas Eikelberg^2^, Iris Haug^2^, Sabrina Schuller^2^, Susanne M. Benseler^2,4^ and Single Hub and Access point for paediatric Rheumatology in Europe (SHARE)

##### ^1^UNIKINDERKLINK TUEBINGEN, Tuebingen, Germany; ^2^Paediatrics, Division of Rheumatology, UNIKINDERKLINK TUEBINGEN, Tuebingen, Germany; ^3^Paediatrics, Division of Rheumatology, Wilhelmina Kinderziekenhuis, Utrecht, Netherlands; ^4^Paediatrics, Division of Rheumatology, ALBERTA CHILDRENS HOSPITAL, Calgary, Canada

###### **Presenting author:** Jasmin Kuemmerle-Deschner

**Introduction:** Innovative, clinical and translational European research in rare diseases mandates collaborations across national borders. However, researchers funded to conduct these critically important studies struggle with the dramatic heterogeneity within and across European countries in research including ethics approval process, frameworks for data and sample collection and sharing. Currently there is no EU-wide framework enabling paediatric research.

**Objectives:** To systematically review the published evidence for best practice in international paediatric research and to establish European recommendations for collaborative paediatric research in paediatric rheumatology.

**Methods:** Within the defined, innovative SHARE framework a scoping review was conducted identifying barriers for collaborative paediatric research. Subsequently, an international expert panel defined the search keywords and strategies of collaborative paediatric research and biobanking. A systematic literature review was performed and reported according to PRISMA guidelines. Published evidence was evaluated and synthesized, recommendations were drafted. In an iterative process, recommendations were refined following in-depth discussion, revisions and reviews with leading Ethics Board members and European experts for ethical and legal aspects of paediatric research. The refined recommendations were shared with European national leaders, reviewed and revised. At the final face-to-face consensus meeting all proposed recommendations were reviewed by the 17 international members of the SHARE expert committee including patient representatives. Each proposed recommendation was discussed in-depth using Nominal Group Technique and voted on requiring an agreement of 80% for inclusion.

**Results:** The scoping review identified four key domains of barrier for collaborative paediatric research including legal, process, research partner- and patient-related barriers. The comprehensive systematic literature review returned 1286 publications. Stepwise primary and secondary screening using pre-defined criteria resulted in a total of 223 papers, of which 85 papers were included; the evidence levels were determined. A total of 21 recommendations were drafted grouped into the domains of 1) general principles (recommendation 1.-3.), ethics (recommendation 4.-7.), paediatric aspects (recommendation 8.-9.), consent/assent (recommendation 10.-14.), data and biospecimen banking (recommendation 15.-17.) and privacy/data access (recommendation 18.-21.). Iterative reviews refined all recommendations resulting in an agreement of >80% for all 21 recommendations at the final consensus conference.

**Conclusion:** The innovative SHARE initiative enabled the development of the first European recommendations for pediatric collaborative research enabling data- and biobanking and sharing across borders. These recommendations provide strong support for an urgently needed European legislative framework and evidence-based guidance for its implementation. Children with rheumatic conditions and the generation of many others suffering from rare diseases should no longer be left behind when life-changing research discoveries can be made.

**Disclosure of Interest**

None Declared.

### P305 Association of the IL6 gene polymorphism with the response to methotrexate in patients with juvenile idiopathic arthritis

#### Liliia S. Nazarova^1^, Kseniia V. Danilko^1^, Viktor A. Malievsky^1^, Tatiana V. Viktorova^1,2^

##### ^1^Bashkir State Medical University; ^2^Institute of Biochemistry and Genetics, Ufa, Russian Federation

###### **Presenting author:** Viktor A. Malievsky

**Introduction:** Methotrexate (MTX) is the most widely used disease-modifying anti-rheumatic drug in the treatment of juvenile idiopathic arthritis (JIA); however, no unequivocal predictive single nucleotide polymorphism (SNP) has been found yet [1].

**Objectives:** The goal of this study was to determine whether the *IL6* gene -174G > C (rs1800795) SNP is associated with the response to MTX in patients with JIA.

**Methods:** The study included 276 patients with JIA who were divided into 7 subgroups according to the ILAR classification. The disease was considered more severe if the MTX treatment was required. The response to MTX was analyzed in 176 patients. The response was considered good if patient achieved clinical remission on medication (Wallace), otherwise it was regarded as insufficient. Genotyping was executed using real-time PCR method. Statistical analysis was performed using two-tailed Fisher's exact test (p), odds ratio (OR), 95% confidence interval (95% CI) and logistic regression.

**Results:** The alleles and genotypes distribution of the *IL6* gene -174G > C SNP was similar in patients with (n = 257) and without (n = 16) requirement of the MTX treatment (p = 0.692). When analyzed according to the MTX response there was only a trend toward a higher proportion of the GC genotype and a lower proportion of the GG genotype in JIA patients who had not achieved clinical remission on medication (n = 107) than in those who had achieved (n = 69) (55.1% vs. 40.6%, p = 0.065 for the GC genotype and 32.7% vs. 46.4%, p = 0.081 for the GG genotype, respectively). The same analysis was performed separately for the patients with the three most frequent JIA subtypes: persistent oligoarthritis (n = 110), RF-negative polyarthritis (n = 81) and systemic JIA (n = 28). The C allele frequency was significantly higher, while the G allele and the GG genotype frequencies were significantly lower in persistent oligoarthritis patients with an insufficient response to MTX (n = 20), than in those with a good response (n = 32) (55.0% vs. 29.7%, p = 0.013, OR = 2.90, 95%CI 1.23-6.60 for the C allele; 15.0% vs. 53.1%, p = 0.0083, OR = 0.16, 95%CI 0.04-0.66 for the GG genotype; 45.0% vs. 70.3%, p = 0.013, OR = 0.35, 95%CI 0.15-0.81 for the G allele, respectively). The best model of inheritance was dominant, where the presence of the C allele marked an increased risk of an insufficient response to MTX (CC + GC vs. GG, p = 0.0043, OR = 6.42, 95% CI 1.57-26.31). No significant differences were observed for patients with two other subtypes. On the contrary, there was a trend toward a decrease of the frequency of the CC genotype in children with RF-negative polyarthritis and an insufficient response to MTX (p = 0.058). It should be noted, that the C allele and the CC genotype frequencies were significantly higher, and the G allele frequency was significantly lower in patients with persistent oligoarthritis, than in other children with JIA (45.0% vs. 33.7%, p = 0.0094, for the C allele; 21.8% vs. 10.8%, p = 0.016, for the CC genotype; 55.0% vs. 66.3%, p = 0.0094, for the G allele). Smaller sample sizes could partially explain the lack of the association of the *IL6* gene -174G > C SNP with the response to MTX in patients with RF-negative polyarthritis and systemic JIA.

**Conclusion:** In this study we revealed that the C allele of the *IL6* gene -174G > C SNP may serve as a marker of increased risk of an insufficient response to MTX in patients with persistent oligoarticular JIA.

**Reference**

[1] Van Dijkhuizen EP, Wulffraat NM. Prediction of methotrexate efficacy and adverse events in patients with juvenile idiopathic arthritis: a systematic literature review. Pediatric Rheumatology Online Journal. 2014;12:51.

**Disclosure of Interest**

None Declared.

### P306 PTEN syndrome diagnosed using a next-generation sequencing targeted gene panel in a young boy with macrocephaly, autism and cutaneous vasculitis

#### Angela Mauro^1^, Ebun Omoyinmi^2^, Angela Barnicoat^3^, Paul Brogan^4^

##### ^1^Rheumatology Unit, Second University of Naples, Naples, Italy; ^2^Infection, Inflammation and Rheumatology Section, Institute of Child Health, UCL; ^3^Clinical Genetics Department, Great Ormond Street Hospital; ^4^Infection, Inflammation and Rheumatology Section, Institute of Child Health, UCL, London, UK

###### **Presenting author:** Angela Mauro

**Introduction:** A 3 year-old boy was investigated for skin lesions on lower and upper limbs. His family history was unremarkable. He was born by forceps delivery at 42 weeks following a pregnancy with normal antenatal scans. He walked independently at about 19 months of age. Parents reported that he had been previously diagnosed with an autistic spectrum disorder and had suffered from recurrent upper airway infections, including severe episode of croup requiring an admission to intensive care at the age of 2 years. Screening for immunodeficiency only revealed persistent mild lymphopenia. On physical examination he had macrocephaly (head circumference >97.5th centile), and had an unusual vasculitic rash with distinct circinate pattern. Skin biopsy revealed thrombotic lymphocytic vasculitis with absence of immune-complex deposition on immunofluorescence.

**Objectives:** The presence of both autism, macrocephaly, and unusual vasculitic rash prompted us to screen him using a targeted next generation sequencing gene panel, called the “vasculitis and inflammation” (VIP) panel, described more detail in a separate abstract.

**Methods:** The Agilent SureDesign tool was used to design an NGS panel targeting 113 genes, grouped into 9 broad clinical phenotypes: autoinflammatory disease; monogenic vasculitis/vasculopathy; complement defects; monogenic lupus; HLH; early-onset inflammatory bowel disease; autoimmune lymphoproliferative syndromes; monogenic stroke; and hereditary amyloidosis. The targeted region includes coding exons, conserved non-coding exons, upstream promoter regions, and splice sites. Captured and indexed libraries (QXT Target Enrichment System) were sequenced as a multiplex of 16 samples on an Illumina MiSeq sequencer in paired-end mode. Read alignment, variant calling, and annotation were performed using Agilent SureCall v3.0 software. Validation of the VIP gene panel is described in a separate abstract.

**Results:** The results showed a mutation in the *PTEN* gene, c.650 T > A, p.V217D, which encodes for Phosphatase and tensin homolog protein, mutation of which is associated with Cowden Syndrome (CS), and related entities. CS, also known as "Multiple hamartoma syndrome" is a rare autosomal dominant inherited disorder characterized by hamartomas in the skin, mucous membranes, thyroid gland, and breast tissue. Patients with CS are at increased risk of cancer including breast, thyroid, endometrial, and renal cancers. Mutations in *PTEN*, a tumour suppressor gene, lead to hyperactivity of the mTOR pathway. Clinical clues for possible diagnosis are macrocephaly, and at least one of autism, dermatologic features, vascular features and gastrointestinal polyposis. The association between CS and immune dysregulation has been described in a few cases, and was the reason we included this gene in our VIP gene panel. PTEN is an important regulator of T-cell maturation, and we speculate that lymphocytic vasculitis could be linked to this genetic mutation, although the exact mechanism remains uncertain

**Conclusion:** We conclude that our targeted VIP panel revealed a diagnosis that we hitherto had not considered, and provides a further evidence of the diagnostic utility of this approach.

**Disclosure of Interest**

None Declared.

### P307 DOWN'S ARTHROPATHY - CLINICAL AND RADIOLOGICAL FEATURES OF ARTHRITIS IN CHILDREN WITH TRISOMY 21

#### Charlene Foley^1^, Orla Killeen^1^, Emma MacDermott^1^, Douglas Veale^2^

##### ^1^National Centre for Paediatric Rheumatology, Our Lady's Children's Hospital Crumlin; ^2^Rheumatology, St Vincent's University Hospital, Dublin, Ireland

###### **Presenting author:** Charlene Foley

**Introduction:** Down's Arthropathy (DA) was first reported in the literature in 1984. Crude estimates suggest higher incidence and prevalence rates of DA compared with Juvenile Idiopathic Arthritis (JIA), (JIA prevalence 1/1000, estimated DA prevalence 8.7/1000). Despite this fact, there remains a paucity of data on this condition. DA is rarely recognised at onset, & remains under-diagnosed. As a direct consequence children with DA are presenting with significant joint damage and disability at diagnosis.

**Objectives:** · Perform a musculoskeletal examination on children with Trisomy 21 (T21) aged 0-20 years

**Methods:** Children with T21 were invited to attend a screening clinic. Screening involved completion of a health questionnaire & a comprehensive musculoskeletal examination. DA cases detected were investigated & managed as per normal clinical practice. Data on a convenience sample of 33 newly diagnosed children with JIA was collected to create a comparison group.

**Results:** 503 children with T21 have been screened for DA, 22 new cases have been diagnosed. All of these children had poor language skills or were non-verbal. Only 11% of the parents suspected that their child may have arthritis prior to attending our screening clinics, and this was only after reading our recruitment literature. In total, we now have 33 children attending our centre with DA (combining cases attending pre-dating the start date of the study). This suggests the prevalence of DA in Ireland is 18-21/1000. The majority of children presented with a polyarticular pattern of disease. No cases of uveitis have been observed to date. 88% of the DA cohort had small joint involvement of the hands, significantly higher than that observed in the JIA comparison group. Erosive changes were reported on X-Ray in 29.2% of the DA cohort (9.5% in the JIA Cohort). Methotrexate-associated nausea was a significant barrier to treatment with this DMARD in DA. There was a significant delay in diagnosis of DA, 1.7 years v 0.7 years in the JIA cohort.

**Conclusion:** Children with T21 are at increased risk of developing arthritis. There is a lack of awareness of this risk among health care professionals & the general public at large. This almost certainly contributes to poor recognition of the disease and a delay in diagnosis. The predominant pattern of disease is polyarticular small joint arthritis. Treatment with standard protocols used in JIA is complicated by drug-associated side effects in children with T21. However, a good response to treatment with steroid intra-articular joint injections has been observed. Our study has raised a number of questions. Future research to accurately define this disease & identify best practice with regards to treatment would be invaluable. We advocate that all children with T21 should have annual musculoskeletal examination as part of their health surveillance programme.

**Disclosure of Interest**

None Declared.

### P308 Musculoskeletal anomalies in a cohort of children with trisomy 21

#### Charlene Foley^1^, Orla Killeen^1^, Emma MacDermott^1^, Douglas Veale^2^

##### ^1^National Centre for Paediatric Rheumatology, Our Lady's Children's Hospital Crumlin, Dublin, Ireland; ^2^Rheumatology, St Vincent's University Hospital, Dublin, Ireland

###### **Presenting author:** Charlene Foley

**Introduction:** Musculoskeletal complications of Trisomy 21 (T21) are common. Almost all children with T21 have muscle hypotonia and joint laxity. The combination of this ligamentous laxity and low muscle tone contribute to an increased risk of a number of musculoskeletal disorders, a delay in acquisition of motor milestones and lower levels of physical activity. Inappropriately low expectations of physical activity and motor function from family, health care workers and self, and over-attributing motor difficulties to low tone and hypermobility may lead to missed pathology and misdiagnoses.

**Objectives:** 1. To describe the musculoskeletal anomalies observed in a national cohort of children with T21

2. To calculate the average age children with T21 walked unaided in our cohort.

**Methods:** Over an 18-month period, children with T21 were invited to attend for a musculoskeletal assessment by a paediatric doctor. Relevant musculoskeletal history and clinical findings were documented.

**Results:** 503 children with T21 were examined (56% male). Median age 8.1 years (0.6-19.2 years).

*Musculoskeletal Anomalies and Trisomy 21.* Pes Planus was the most common musculoskeletal anomaly detected, occurring in 91.1% of the children with T21 examined. Just under a quarter of these children did not avail of orthoses (23.6%). A range of other anomalies were observed, inflammatory arthritis (7.1%) and scoliosis (4.8%) occurring most frequently after pes planus. Other spinal abnormalities included the well-documented T21 associated c-spine instability, absent C2 vertebra and spondylolisthesis. Common hip and foot pathologies included dislocations, Perthes disease, slipped upper femoral epiphysis (SUFE) and hallux valgus.

*Ambulation and Trisomy 21.* The median age our cohort walked was 28 months (12-84 months). This is comparable to the literature that reports children with T21 walk at 23 months (range 13-48), compared with 12 months (range 9-17) for the general paediatric population.

**Conclusion:** Children with T21 are at increased risk of a number of potentially debilitating musculoskeletal problems. Early, regular and continuous musculoskeletal assessment of children with Down syndrome is paramount to management of musculoskeletal conditions. The aim is to avoid children presenting with irreversible, preventable joint damage and disability due to delayed or incorrect diagnosis and management of these very treatable conditions.

**Key Message(s)**

- Pes planus is common in children with Trisomy 21, therefore early consideration of orthotics and life-long appropriate supportive footwear is advised.

- When a child with Down syndrome presents with a limp they should always be referred for a hip x-ray.

- A high index of suspicion for pathology should be employed when assessing a child with Down syndrome presenting with change and/or deterioration in function and mobility.

- Inflammatory arthritis in children with Down syndrome is common and potentially erosive and debilitating if left untreated.

- Significantly delayed ambulation is noted in children with T21. Variability exists in the basic biomechanics of the musculoskeletal system in children with Down syndrome in terms of motor control, coordination, and skill. Multidisciplinary team assessment and management should be early, regular and ongoing to ensure these children reach their potential with regards to motor function.

- Compulsory annual musculoskeletal assessment for all children with T21 would enable early detection of potential problems, allowing for timely intervention and in-turn better clinical outcomes.

**Disclosure of Interest**

None Declared.

### P309 Coffin-Siris syndrome with ARID1B mutations: a genetic syndrome linked with arthritis?

#### Sonia Melo Gomes^1,2^, Ebun Omoyinmi^1^, Jane Hurst^3^, Nathalie Canham^4^, Despina Eleftheriou^1,2^, Nigel Klein^1,5^, Sandrine Lacassagne^2^, Paul Brogan^1,2^

##### ^1^Rheumatology, Institute of Child Health, London, UK; ^2^Rheumatology, Great Ormond Street Hospital, London, UK; ^3^Genetics, Great Ormond Street Hospital, London, UK; ^4^Genetics, London Northwest Healthcare NHS Trust, London, UK; ^5^Infectious Diseases, Great Ormond Street Hospital, London, UK

###### **Presenting author:** Sonia Melo Gomes

**Introduction:** Coffin-Siris (CSS) and Nicolaides-Baraitser (NBS) are two overlapping syndromes caused by mutations in genes associated with the chromatin remodeling complex, which are associated with multiple malformations and intellectual disability. Musculo-skeletal changes, such as prominence of inter-phalangeal joints in hands, feet, and knee joints are very common in NBS (up to 75%), and also reported in CSS. These changes are usually considered to be dysplastic in nature rather than inflammatory, however.

**Objectives:** To identify the genetic cause in a child with some clinical features suggestive of NBS and diffuse polyarthritis.

**Methods:** We performed Whole Exome Sequencing (WES) on the proband and immediate family members. WES results were confirmed by conventional Sanger sequencing.

**Results: Case report-** We present the case of a 7 year old boy with a long standing boggy polyarthritis, a previous history of developmental delay, microcephaly (<4th centile), and distinct dysmorphic features that were reminiscent of Nicolaides-Baraitser Syndrome. However, Sanger sequencing of the SMARCA2 gene was negative (wild-type). There were no signs of uveitis. Laboratory tests showed normal inflammatory markers, negative autoantibody screen, and normal complement levels. Synovial biopsy confirmed the presence of a chronic inflammatory synovitis consistent with juvenile idiopathic arthritis. Brain MRI revealed a dysgenetic corpus callosum. Treatment was started with methotrexate (15 mg/m^2^/week, subcutaneously), which failed to control the polyarthritis; etanercept was subsequently added, leading to significant improvement.

Whole exome sequencing (WES) was performed on the patient and immediate family members, revealing a novel, de novo heterozygous missense mutation in exon 20 of the *ARID1B* gene, resulting in a premature stop codon at amino acid position 1802 (c.C5404T; p.R1802X), thus confirming the diagnosis of CSS. Sanger sequencing confirmed the presence of the mutation in the patient and segregation with disease within the family.

**Conclusion:** This case illustrates yet again the power of next-generation sequencing to provide molecular confirmation of a rare diagnosis, CSS caused by a heterozygous mutation in *ARID1B*. The absence of significantly raised inflammatory markers, in addition to a suspected clinical diagnosis of a genetic syndrome known to be associated with skeletal dysplasia almost certainly contributed to this patient’s polyarthritis not being recognized for several years. We propose that inflammatory arthritis may be an important feature of CSS and related syndromes. In general, it is increasingly recognized that some patients with skeletal dysplasia may also have inflammatory arthritis, and early recognition of this and appropriate treatment may reduce morbidity and long-term damage.

**Disclosure of Interest**

None Declared.

## Poster Session: Immunoregulation and basic science

### P310 Whole transcriptome analysis confirms HLA-DRB1 as a strongly regulated gene in systemic juvenile idiopathic arthritis in remission

#### Anastasia Wiener^1^, Boris Hügle^2^, Bernd Denecke^3^, Ivan Costa-Filho^3^, Johannes Peter Haas^2^, Klaus Tenbrock^1^

##### ^1^Pediatrics, RWTH AACHEN UNIVERSITY, Aachen, Germany; ^2^Pediatrics, Deutsches Zentrum für Kinderrheumatologie, Garmisch-Partenkirchen, Germany; ^3^IZKF, RWTH AACHEN UNIVERSITY, Aachen, Germany

###### **Presenting author:** Anastasia Wiener

**Introduction:** Systemic juvenile idiopathic arthritis (sJIA) is an autoinflammatory disease characterized by arthritis and severe systemic inflammation. Treatment with interleukin (IL)-1 antagonists has shown to be effective when introduced early in the disease. No markers that predict response to therapy have been identified yet.

**Objectives:** The objective of this study was a longitudinal whole transcriptome analysis of children with sJIA during the early phase of treatment with IL-1 antagonists to identify novel targets that predict response to therapy.

**Methods:** From the database of the German AIDnet database, patients with sJIA and active systemic disease treated with anakinra and subsequent remission were identified. Clinical data was obtained by retrospective chart review. Whole blood was drawn during active disease before initiation of anakinra and after achievement of remission. RNA was extracted and subjected to Affymetrix HTA 2.0 Arrays followed by intraindividual analysis of regulated genes in each patient and combined analysis of the genes in all the patients including GO analysis of differentially expressed genes. In addition regulatory genomics toolbox motif enrichment analysis was performed to identify transcription factors and pathways that are redulated during therapy.

**Results:** Six children with sJIA with active systemic disease were included in the study. Using a p-value of <0.01 and a fold change of 2 or greater, 742 genes were identified of which most were associated with immune mediated processes. Using a fold change of higher than 3 as a more stringent criterium, still more than 100 genes remained. Our analysis revealed HLADRB1 as the most strongly upregulated gene in remission compared to active disease (FC 6.8). This gene has been recently identified as a risk factor in an association study of 982 children with sJIA and 8,010 healthy control subjects. Moreover besides e.g. S100A8 as known disease marker an upregulation of CD177 during active disease was identified, which is a molecule engaged in neutrophil activation and transmigration and has not been described in previous studies. Motif analysis revealed upregulation of promoters bound by STAT3 and STAT4 in active disease, transcriptionfactors downstream of IL-6 and IL-12 signaling respectively.

**Conclusion:** Our study identifies the strong up-regulation of HLA-DRB1 in patients with sJIA in remission upon treatment with IL-1 antagonists. This provides a functional confirmation of a previous study, which identified HLA-DRB1 as a risk factor in sJIA. Additionally CD177 was observed as a possible new marker in sJIA. Studies with larger patient cohorts are necessary to confirm these results.

**Disclosure of Interest**

None Declared.

### P311 S100A8/A9 regulates immune responses in dendritic cells

#### David Popp^1^, Arjan Boltjes^2^, Frank Rühle^3^, Stefanie Herresthal^4^, Wilco de Jager^5^, Femke van Wijk^2^, Joachim Schultze^4^, Monika Stoll^3^, Luisa Klotz^6^, Thomas Vogl^1^, Johannes Roth^1^

##### ^1^Institute of Immunology, Muenster, Germany; ^2^Division of Pediatrics, UMC Utrecht, Utrecht, Netherlands; ^3^Institute of Human Genetics, Muenster, Germany; ^4^LIMES-Institute, Bonn, Germany; ^5^Multiplex Core Facility, UMC Utrecht, Utrecht, Netherlands; ^6^Institute of Neurology, Muenster, Germany

###### **Presenting author:** David Popp

**Introduction:** In recent years S100A8 and S100A9 proved to be reliable biomarkers in chronic inflammatory disorders especially in systemic and non-systemic forms of juvenile idiopathic arthritis (JIA). They are highly expressed and released at local sites of synovial inflammation. On a molecular level these proteins function as damage-associated molecular pattern molecules (DAMPs), which amplify inflammation by binding to Toll‐like receptor‐4 (TLR‐4). Released from activated phagocytes or necrotic cells the physiologically relevant S100A8/A9 heterodimers stimulate surrounding cells in order to promote pro-inflammatory processes in innate immunity. Activation of dendritic cells (DCs) via TLR-4 is believed to further trigger adaptive immunity. Interestingly, we now identified a novel immune-regulatory function of S100 proteins in monocyte-derived DCs.

**Objectives:** This study aims to demonstrate the underlying mechanisms of immune regulation by S100 proteins in DCs on a molecular as well as functional level.

**Methods:** Human monocyte derived DCs are differentiated with or without exposure to S100A8 for six days prior to activation with LPS. After characterization and determination of the activation status using flow cytometry, the ability of these cells to induce autologous CD4^+^, CD8^+^, and γδ T-cell proliferation is investigated in co-cultures in vitro. In addition cytokines and chemokines, secreted during DC differentiation and DC/T-cell co-culture, where analyzed by Luminex cytokine arrays. The metabolic state of DCs was further examined by using Seahorse XFp Analyzer assays. To identify the molecular mechanisms leading to the observed phenotype the mRNA expression of human DCs at various stages of differentiation was screened by genome-wide gene expression arrays.

**Results:** Our results demonstrate that S100A8 exposure to developing monocyte-derived DCs impairs differentiation and subsequent activation of DCs and therefore leads to an unresponsive, immune-suppressive DC phenotype. This phenotype is characterized by significantly reduced surface expression of cell-type specific activation markers and diminished cellular glycolysis and mitochondrial respiration. Furthermore, suppressive DCs secrete reduced amounts of pro-inflammatory IL-1β and IL-6, IL-12 and IL-18 and chemo-attractant CCL19 and CXCL10 whereas the secretion of many other cytokines and chemokines is not affected. In co-cultures with autologous T-cells, suppressive DCs show significantly reduced potential to induce T-cell proliferation in CD4^+^, CD8^+^, and γδ T-cells. Genome-wide gene expression analysis on RNA level and subsequent studies of the affected transcription factor networks indicated a novel role of the well-known master regulator of LPS-induced genes C/EBPδ for the induction of S100-induced suppressive DCs. We already confirmed the differential S100-induced expression of C/EBPδ on protein level.

**Conclusion:** Taken together, our results represent a novel regulatory mechanism of S100A8 in innate immunity to prevent overwhelming immune responses. A disturbed balance of this regulatory mechanism of S100A8/S100A9 on adaptive immunity and the well-known TLR-4-dependent pro-inflammatory effects of these potent DAMPs on innate immune cells may play a significant role in the inflammatory process of JIA.

**Disclosure of Interest**

None Declared.

### P312 In depth immunophenotypic analysis in juvenile idiopathic arthritis (JIA): a crossectional study

#### Estefania Quesada-Masachs^1^, Daniel Álvarez de la Sierra^2^, Marina Garcia Prat^2^, Ana M. Marín Sánchez^2^, Ricardo Pujol Borrell^2^, Sara Marsal Barril^3^, Mónica Martínez Gallo^2^, Consuelo Modesto Caballero^1^

##### ^1^Paediatric Rheumatology Unit, Hospital Universitario Vall d'Hebron, Barcelona, Spain; ^2^Immunology, Hospital Universitario Vall d'Hebron, Barcelona, Spain; ^3^Rheumatology, Hospital Universitario Vall d'Hebron, Barcelona, Spain

###### **Presenting author:** Estefania Quesada-Masachs

**Introduction:** The potential of expanded flow cytometric analysis of peripheral blood lymphocytes (PBLs) has not been yet been fully exploited to study JIA heterogeneity neither to monitor JIA patients’ on biological treatments.

**Objectives:** To analyze in depth lymphocyte subpopulations in treated JIA patients stratified by age and gender.

**Methods:** 101 patients, 69 pediatric and 32 adult fulfilling JIA (ILAR) criteria attending HUVH rheumatology clinic were included. All patients were treated with either MTX, anti-TNFα, or a combination of both. Controls were sex/age matched volunteers either healthy or attending the clinics of HUVH for non inflammatory conditions (59 controls, 29 pediatric and 30 adult). Demographical, clinical and analytical variables were recorded. Extended immunophenotype following a protocol based on the Human ImmunoPhenotyping Consortium (HIP-C) protocol that discerns 57PBL subpopulations was applied. Bonferrroni’s correction indicated that the cut off for significance was p < 0.00041667.

Human ImmunoPhenotyping Consortium (HIP-C)FITMaN protocol that discerns 57PBL subpopulations was applied. Bonferrroni’s correction indicated that the cut off for significance was p < 0.00041667.

**Results:** Changes common to each group of patients irrespective of treatment are as follows: pediatric patients showed lower percentage of CD8 effector cells (CD45RA + CCR7-) when compared to controls (10.53 ± 5.71 vs 16.92 ± 5.71). Percentage of total CD4 cells was higher on JIA children than in controls (42.21 ± 7.40 vs 36.04 ± 5.22). No differences were found on percentages nor in absolute number of Th1 (CXCR3 + CCR6-), Th2 (CXCR3-CCR6-), or Th17 (CXCR3-CCR6-) CD4 cells. Interestingly all Th subpopulations showed decreased expression of HLA-DR in JIA children when compared with controls (6.06 ± 3.08 vs 10.49 ± 5.02; 0.81 ± 0.51 vs 1.48 ± 0.64; 8.57 ± 3.81 vs 12.6 ± 4.95; respectively). JIA adults presented no significant differences in T cells subpopulations compared to HC, but showed higher percentage of CD21- switch-memory B cells (IgD-IgM-CD27-) than controls (10.82 ± 5.33 vs 4.29 ± 4.33).

Regarding the treatment followed just in JIA patients few differences were observed. Pediatric patients treated with MTX (n = 27) showed a lower proportion of the effector memory (CD45RA-CCR7-) cells both CD4+ and CD8+, when compared to controls, anti-TNF, and anti-TNF plus MTX groups (20.01 ± 7.76 vs 27.64 ± 9.27; 20.01 ± 7.76 vs 27.84 ± 4.27; 20.01 ± 7.76 vs 27.07 ± 10.05; for CD4s and 26.42 ± 7.23 vs 37.10 ± 12.38; 26.42 ± 7.23 vs 42.08 ± 11.59; 26.42 ± 7.23 vs 37.27 ± 12.54 for CD8s, respectively). Percentage of CD25+ lymphocytes was higher in the anti-TNF plus MTX group. The Th1-17 (CCR6 + CXCR3+) CD4 cell subpopulation was significantly increased in anti-TNF and anti-TNFα plus MTX groups compared with controls and MTX (10.45 ± 3.14 vs 6.38 ± 3.89; 10.45 ± 3.14 vs 4.78 ± 2.56 and 9.43 ± 4.63 vs 6.38 ± 3.89; 9.43 ± 4.63 vs 4.78 ± 2.56, respectively). Adult patients treated with anti-TNFα + MTX and MTX presented higher levels of CD21- naïve B cells compared to HC and to anti-TNF groups (4.86 ± 3.24 vs 2.32 ± 1.69; 4.86 ± 3.24 vs 2.95 ± 1.94 and 5.38 ± 1.54 vs 2.32 ± 1.69; 5.38 ± 1.54 vs 2.95 ± 1.94).

**Conclusion:** In depth phenotypic analysis of PBL in JIA showed alterations in cell subpopulations that seem dependent on age group, and also can be a valuable tool for monitoring and comparing JIA treatments.

**Disclosure of Interest**

None Declared.

### P313 Immunological and biochemical parameters in children with rheumatic diseases

#### Iryna Chyzheuskaya^1,2^, Lyudmyla M. Byelyaeva^2^, Rostislav M. Filonovich^1^, Helena K. Khrustaleva^3^, Larisa I. Zajtseva^4^, Tamara M. Yuraga^5^

##### ^1^Pediatrics, 4th City Children's Clinical Hospital, Minsk, Belarus; ^2^Pediatrics, Belarusian Medical Academy of Postgraduate Education, Minsk, Belarus; ^3^Pediatric, Belarusian Medical Academy of Postgraduate Education; ^4^Pediatric, 4th City Children's Clinical Hospital, Minsk, Belarus; ^5^Scientific Research Laboratory, Belarusian Medical Academy of Postgraduate Education, Minsk, Belarus

###### **Presenting author:** Iryna Chyzheuskaya

**Introduction:** The relevance of rheumatic diseases (RD) in childhood is associated with an increase in their prevalence, severity and nature of the disabling high flow rate of adverse outcomes and complications. Of the group of RD in children are more common juvenile idiopathic arthritis (JIA), systemic lupus erythematosus (SLE) and juvenile scleroderma (JS).

**Objectives:** The purpose of research - to determine the immunological and biochemical parameters in children with JIA, SLE and JS.

**Methods:** The study involved 167 patients with RD, among them were 54 boys and 113 girls. Research carried out in the rheumatology department of 4th city clinical hospital of Minsk and the research laboratory of the Belarusian Medical Academy of Postgraduate Education in the period from 2008 to 2015. All patients were divided into 3 clinical groups. In the I group included 115 children with JIA (mean age 11,9 ± 3,4 years), II group consisted of 34 children with JS (mean age 12,4 ± 2,8 years), III - 18 children with SLE (mean age 13,1 ± 1,7 years). All the children were identified content tumor necrosis factor alpha (TNF-alpha), content of primary and secondary lipid peroxidation products, as well as levels of water-soluble and fat-soluble antioxidant capacity of substances in the serum.

**Results:** According to the results of the study found a significant increase in TNF-α in serum in children with JIA, SLE (P <0.01) and JS (P <0.001) when compared with the control group. The individual values of serum TNF-α were elevated in 27 (79.4%) patients with JS, in 98 (85.2%) of children with JIA, all children with SLE. The maximum value of this index were observed in patients with JS with rapidly progressive course of the disease (298,6 ± 19,3 pg/ml) and in children with systemic JIA option (273,98 ± 24,7 pg/ml). Correlation analysis revealed the relationship between TNF-α level and disease activity (r = 0,73; P < 0.001), between TNF-α and CRP (r = 0,64; P < 0.01). The content of TNF-α was significantly higher in patients with high values of rheumatoid factor (RF) than in children with normal RF levels (P < 0.05).

A significant role in the development and progression of RD plays acceleration of lipid peroxidation and antioxidant system inconsistency. The study found a significant (P < 0.05-0.001) increasing of lipid peroxidation products in the blood serum of patients of all clinical groups compared with the control group. Oxidized lipids have antigenic properties, triggering autoimmune processes of tissue damage. During the correlation analysis found a positive correlation between the levels of dienketones and dienkonyugates serum and erythrocyte sedimentation rate (r_s_ = 0,287, P < 0.001).

The study in children of all clinical groups found significant (P < 0.001) reduction in serum ACW and ACL substances when compared to the control group, suggesting disturbance antioxidant protection. A negative correlation between the level of TNF-α and ACL in the blood serum (r_s_ = -0,346, P < 0.05) between the content of ACW and TNF-α in the serum (r_s_ = -0,54, P < 0.001) established.

**Conclusion:** The results indicate that an increase in TNF-α level at RD reflects inflammatory activity of the disease. The observed increase in the content of primary and secondary products of lipid peroxidation in the serum, as well as reducing the level of water-soluble and fat-soluble antioxidant capacity in serum substances in children with JIA, JS and SLE indicates an increase in activity of lipid peroxidation and a decrease in antioxidant defense mechanisms.

**Disclosure of Interest**

None Declared.

### P314 Comorbidity and rheumatic diseases in children

#### Iryna Chyzheuskaya^1,2^, Lyudmyla M. Byelyaeva^2^, Rostislav M. Filonovich^1^, Helena K. Khrustaleva^3^, Larisa I. Zajtseva^4^, Tamara M. Yuraga^5^

##### ^1^Pediatrics, 4th City Children's Clinical Hospital, Minsk, Belarus; ^2^Pediatrics, Belarusian Medical Academy of Postgraduate Education, Minsk, Belarus; ^3^Pediatric, Belarusian Medical Academy of Postgraduate Education, Minsk, Belarus; ^4^Pediatric, 4th City Children's Clinical Hospital, Minsk, Belarus; ^5^Scientific Research Laboratory, Belarusian Medical Academy of Postgraduate Education, Minsk, Belarus

###### **Presenting author:** Iryna Chyzheuskaya

**Introduction:** For rheumatic diseases (RD) is characterized by the frequent combination with comorbid infection, mainly respiratory and urinary systems. Attention to this issue is primarily due to the fact that infectious complications are a leading cause of adverse outcomes in RD. Early inclusion in the treatment of immunosuppressive drugs and genetic engineering of biological drugs has significantly changed the course and outcomes of the disease and at the same time causes the activation of latent fear of current comorbid infections. In this connection the question arises about how to prevent the emergence and worsening of chronic infections, especially bronchopulmonary and urinary systems, complicating the course of the underlying disease and require additional treatment costs.

**Objectives:** to determine the frequency and the role of comorbid infections in children with rheumatic diseases.

**Methods:** The rheumatology department of the 4th City Children's Clinical Hospital of Minsk were examined 167 patients with RD, among them 115 children with juvenile idiopathic arthritis (JIA) (mean age 11,9 ± 3,4 years), 34 children with juvenile scleroderma (JS) (mean age 12,4 ± 2,8 years) and 18 children with systemic lupus erythematosus (SLE) (mean age 13,1 ± 1,7 years).

**Results:** Infection as a cause of the disease most often referred to patients with JIA and SLE. The most common respiratory diseases observed patients with JIA (55,6%) and SLE (61,1%). Patients with JIA often enough noted the presence of nasopharyngeal infections (sinusitis, pharyngitis and bronchitis – 18, 29 and 14 patients respectively), and patients with a JS (24, 15 and 21 patients respectively). Often sick colds 21,7% of children with JIA and 38.9% of children with SLE, as well as 17,6% of children with JS. Sore throat worried children with RD, on average 2 times a year. More than half of the children observed the occurrence of arthralgias against a background of infections this location. About 1/3 of patients responded positively to the question about the relationship of acute illness with acute respiratory viral infection (35,6% with JIA, SLE 38,9 and 17,6% with JS). Among the 16 children with JIA treated with MTX and adalimumab, respiratory tract infection occurred in 18,7%, including serious - at 6,3%. Cold sores on the lips, nose, wings and other parts of the face, most children appear 2-3 times a year, in some cases up to 7-8 times a year. The most prone to relapse of viral infection were patients with SLE (61,1%), less frequently such episodes occurred in children with JIA (42,6%) and JS (32,8%). Urinary tract infection was observed in 17,3% of children with JIA, 27,8% of children with SLE and 23,5% JS. The frequency of urinary tract infection in children with JIA treated with methotrexate and TNF-alpha inhibitors was 4,1% with methotrexate, 6,3% with TNF-alpha. At the same time in the presence of patients in the history of this disease is the risk of infection is significantly increased.

**Conclusion:** The findings suggest first of all about the importance of infection in the initiation of a number of rare-earth and the need for careful history in patients with RD, which will reveal their comorbid infections that can complicate the course of the underlying disease, and to select the optimal treatment strategy.

**Disclosure of Interest**

None Declared.

### P315 Complement activation profiles in juvenile idiopathic arthritis

#### Thomas Giner^1^, Lukas Hackl^1^, Julia Albrecht^1^, Reinhard Würzner^2^, Juergen Brunner^1^

##### ^1^Pediatrics, Medical University Innsbruck, Innsbruck, Austria; ^2^Division of Hygiene and Medical Microbiology, Medical University Innsbruck, Innsbruck, Austria

###### **Presenting author:** Juergen Brunner

**Introduction:** Juvenile idiopathic arthritis (JIA) summarizes a group of phenotypically heterogeneous chronic inflammatory disease of childhood. The innate immunity is playing a role in the pathogenesis of JIA. Complement is activated by three pathways (classical pathway (CP), lectin pathway (LP) and alternative pathway (AP)). In RA a high turnover of C3, C4 and C5 in inflamed joints is discussed. The role of the complement system in the pathogenesis of JIA is still unclear.

**Objectives:** This is a controlled prospective observational study. It is focused on the three pathways of complement system (CS) and its effector, the membrane attack complex (MAC), associated with disease activity and inflammation markers in all subgroups of JIA. (Trial numberUN373).

**Methods:** Peripheral blood samples (PB) (n = 158) of 57 pediatric JIA patients (partially also in longitudinal visits), were analyzed for specific complement pathway activation (COMPL300 ELISA), complement factor H (CFH)-autoantibodies (CFHAb ELISA) and the soluble MAC (sC5b-9 ELISA) in serum (S) and EDTA-plasma (P)- The JIA subgroups were persistent Oligoarthritis (perOA, n = 19), extended Oligoarthritis (extOA, n = 8), rheumatoid factor positive Polyarthritis (PARF+, n = 4) and negative Polyarthritis (PARF-, n = 12) polyarthritis, Enthesitis related arthritis (ERA, n = 4); Psoriatic arthritis (PsA, n = 3) and systemic JIA (sJIA, n = 7). As control group We tested s(n = 118) healthy adults (n = 100) and children (n = 18) without inflammatory diseases were tested. JADAS10 Score defined acute phase of disease.

**Results:** JIA patients within acute phase of disease (n = 53) showed lower capacity in CP (82% [38-97% IQR] vs 104% [97-115% IQR] (p < 0.001)) and AP (34% [2-97% IQR] vs 85% [70-99% IQR] (p < 0.001)) compared to the control group in median. This can be concluded to chronic over activation in the two complement pathways even though The: in plasma 3,46 AU/ml and serum 19,55 AU/mlonly 40% (29/53) in CP and 36% (26/53) in AP were below the published pathological threshold. Also sMAC was elevated (P 2.3 [1.27-3.43IQR] vs 1.2 [0.84-1.84] AU/ml) in patients with decreased AP in acute phase (p < 0.009) compared to the control group. No evidence of CFH-autoantibodies was found in our study group. The sMAC levels were significantly (p < 0,009) higher in sera (15.5 [12.03-20.91 IQR] AU/ml) and plasma (1.75 [0.9-3.46 IQR] AU/ml) compared to the control group (S 7.78 [4.9-10.32 IQR] AU/ml, P 1.22 [0.78-1.81 IQR] AU/ml) in the patients with extended and persistent OA, in PARF+ and ERA but not in PARF-, PsA and sJIA.

**Conclusion:** Special groups of JIA showed increased CS activation with elevated levels of MAC in PB in acute phase of disease. The additional decreased capacity in the CP and AP suppose that the complement system as an additional contributor in pathogenesis and/or course of the acute disease. Therefore the testing of COMPL300 in combination with sMAC could be a helpful biomarker for acute JIA disease and furthermore the pharmaceutical blockage of parts of the complement system might be a therapeutical option in therapy resistant patients.

**Disclosure of Interest**

None Declared.

### P316 Failure to treat chronic uveitis with sirolimus in a patient affected by JMML

#### Serena Pastore^1^, Marta Minute^2^, Fulvio Parentin^1^, Alessandra Tesser^3^, Agostino Nocerino^4^, Andrea Taddio^5^, Alberto Tommasini^6^

##### ^1^Pediatric, IRCCS Burlo Garofolo, Trieste, Italy; ^2^Pediatric, University of Trieste, Trieste, Italy; ^3^University of Trieste, Trieste, Italy; ^4^Pediatric, S. Maria Misericordia Hospital, University of Udine, Udine, Italy; ^5^Pediatric, University of Trieste, IRCCS Burlo Garofolo, Trieste, Italy; ^6^IRCCS Burlo Garofolo, Trieste, Italy

###### **Presenting author:** Serena Pastore

**Introduction:** JMML (juvenile myelomonocytic leukemia) is a clonal proliferation of hematopoietic stem cells that is characterized by the absence of Philadelphia chromosome and affects usually children younger than 2 years. To set the diagnosis the three major criteria (absence of Bcr-Abl translocation, peripheral monocytosis and presence of less than 20% of blast cells in peripheral blood) are needed and they have to be associated with at least two of the five minor criteria (fetal hemoglobin increased for age, immature granulocytes in the peripheral blood, white blood cell count greater than 1 × 109/L, clonal chromosomal abnormality, granulocyte-macrophage colony-stimulating factor hypersensitivity of myeloid progenitors *in vitro*).

**Objectives:** We describe the case of one patient affected with JMML with homozygous Cbl mutation who develops a severe steroid dependent uveitis.

**Methods:** A 7-year-old male patient came to our attention because of a severe steroid dependent uveitis. The boy had been diagnosed at the age of two with JMML due to hepatosplenomegaly, persistent monocytosis, presence of blast cells in the bone marrow and Cbl homozygous mutation: the disease showed an indolent behavior, and was therefore controlled on a strict follow-up, without any treatment. At the age of three he developed a severe left uveitis, that was treated with topical and systemic corticosteroid with complete resolution after one year. Metotrexhate was started as steroid-sparing agent but it was discontinued because of an infectious pneumonia. One year after ocular remission, bilateral uveitis developed and orally steroid therapy was reinitiated. The boy showed a good response to steroids but every prednisone tapering led to uveitis relapse, configuring the disease as corticosteroid-dependent. Moreover the patient developed different adverse effects such as hypertension and cataract. When he came to our attention, he was on corticosteroid treatment (prednisone 10 mg/day) and he presented bilateral uveitis (Tyndall +1 and cells +1), with an initial cataract of the right eye. His physical examination was remarkable for Cushing-like habitus and for hepatosplenomegaly. His blood count demonstrated 1100/mmc monocytes. Emphasizing the indolent behavior of his hematological disease and the fact that Cbl is strictly linked with Ras-mTOR the disorder could be considered similar to Ras-associated autoimmune leukoproliferative disorder (RALD). On this basis, to treat patient’s uveitis, we proposed a trial with sirolimus, a mTOR inhibitor that showed its efficacy both in primitive uveitis and in autoimmune manifestations associated with JMML. Topical treatment with steroidal and mydriatic eye drops was associated. We started with the dosage of 0.5 mg/day and we slowly increased the dosage to 1.5 mg/day, in order to maintain the blood level of the drug between 5 to 15 ng/ml.

**Results:** After two months of treatment the uveitis did not show any improvement, despite the achievement of an adequate blood concentration of the drug, neither the monocyte count decreased; for this reason sirolimus was suspended and methotrexate was reinitiated.

**Conclusion:** In conclusion, we present the case of a JMML patient with a Cbl mutation that developed a severe steroid dependent uveitis that did not respond to sirolimus. Treatment with sirolimus was attempted emphasizing the common pathway of Cbl and Ras, but its inefficacy might imply that this pathway is not involved in the uveitis pathogenesis.

**Disclosure of Interest**

None Declared.

## Poster Session: JIA (oligo, poly, psoriatic) II

### P317 Accelerometer-assessed daily physical activity in relation to participation in gym-classes and sport activities in JIA

#### Mette Nørgaard^1^, Troels Herlin^2^

##### ^1^Department of Physiotherapy, Department of Pediatrics, Aarhus University Hospital, Aarhus N, Denmark; ^2^Department of Pediatrics, Aarhus University Hospital, Aarhus N, Denmark

###### **Presenting author:** Mette Nørgaard

**Introduction:** Over the last decade implementation of targeted therapy has improved the functional ability in children with JIA considerably. Evidence-based benefits of physical activity (PA) have led to less restriction in participation of JIA patients in PA and sports. However, JIA patients still seem to be less physically active, have lower physical capacity, and are more challenged in PA than healthy peers. Subjective assessments of PA are commonly used but often biased in children but only few data exist on objectively measured PA in children with JIA.

**Objectives:** To objectively monitor daily free-living PA and relate to self-reported exercise habits in gym-classes and club-sports in 10-16 year-old children with JIA compared to healthy peers.

**Methods:** 61 patients (females 60.7%), mean age 13.2 (±1.7) years, mean disease duration 70 (±49) months and 118 controls (females 50.8%), mean age 12.4 (±1.7) years completed the questionnaire: Patterns of Physical Activity in Leisure-time, Education and Sports (PPA-LES).

Accelerometer monitoring was assessed using the GT1M ActiGraph, monitoring daily PA during wakening hours for one week. Accelerometry assessments of JIA patients were compared to age and gender related normative Danish reference values (n = 2055) and expressed as mean counts per minute (meanAcc), minutes per days with > 1000 counts/min (Acc1000) and minutes per day >2500 counts/min (Acc2500).

**Results:** Significantly fewer patients participated fully in gym-classes compared to healthy controls (51.5% vs. 76.3%, p < 0.01) and fewer patients reported participation in sport club activities (62% vs. 76%). Of those in the JIA cohort participating in sport 29.5% spent >3 hours per week compared to 48.3% in the control group. But overall no significant difference was found between sport-active patients and controls in the reported time spent in sports (p = 0.202).

As previously reported (1) patients had decreased PA as assessed by accelerometry (PA-Acc) compared to healthy controls, both regarding meanAcc (p < 0.004), Acc1000(p < 0.001) and Acc2500 (p < 0.002).

We found no relation when comparing Z-scores of age and gender controlled values of PA-Acc to the reported participation in gym-classes (Fig. 3). However, when reporting activites in sports we found an increasing level of Z-scores of PA-Acc proportional to the number of hours spent per week in sports (Fig. 3). In fact, patients were even more active than normative controls, when spending more than 5 hrs/week in sports.

**Conclusion:** Patients with JIA were less physically active than healthy controls both when assessing participation in gym-classes and sport club activities by self-reported questionnaire (PPA-LES) and when objectively assessing PA by accelerometry (PA-Acc). However, the reported participation in gym-classes and sports differently reflected the activity recorded by objectively measured PA-Acc. Our results may emphasize the need for accurate measures when assessing PA in children with JIA. 1. Nørgaard M et al. Accelerometry-based monitoring of daily physical activity in children with juvenile arthritis. Scand J Rheumatol 2016; 45(3): 179-187.

**Disclosure of Interest**

None Declared.

### P318 High sensitive CRP as a predictive marker of long-term outcome in juvenile idiopathic arthritis

#### Mikel Alberdi-Saugstrup^1,2,3^, Marek S. Zak^1^, Susan M. Nielsen^1^, Troels Herlin^4^, Ellen Nordal^5^, Lillemor Berntson^6^, Anders Fasth^7^, Marite Rygg^8^, Klaus G. Müller^1,3^ and Nordic Study Group of Pediatric Rheumatology (NoSPeR)

##### ^1^Department of pediatrics and adolscent medicine, Copenhagen University Hospital, Rigshospitalet, Copenhagen; ^2^Department of pediatrics, Naestved hospital, Naestved; ^3^Institute of Inflammation Research, Copenhagen University Hospital, Rigshospitalet, Copenhagen, ^4^Department of Pediatrics, Aarhus University Hospital, Aarhus, Denmark; ^5^Department of Pediatrics, University Hospital of North Norway, Tromsø, Norway; ^6^Department of Women’s and Children’s Health, Uppsala University, Uppsala; ^7^Department of Pediatrics, University of Gothenburg, Gothenburg, Sweden; ^8^Department of Laboratory Medicine, Children’s and Women’s Health, Norwegian University of Science and Technology and Department of Pediatrics, St.Olavs Hospital, Trondheim, Norway

###### **Presenting author:** Mikel Alberdi-Saugstrup

**Introduction:** Juvenile idiopathic arthritis (JIA) is a group of chronic rheumatic diseases in childhood. For some children it is a mild disease, but for others it is a long-lasting and potentially disabling disease. Unfortunately, our insight into prognostic risk factors is still limited and although several predictive markers of outcome have been proposed, results have been inconsistent.

In a large proportion of patients, the level of the standard inflammatory markers, erythrocyte sedimentation rate (ESR) and C-reactive protein (CRP), is within the normal range despite clinical signs of disease activity.

**Objectives:** To evaluate whether C-reactive protein (CRP), including variation within the normal range, is predictive of long-term disease outcome in juvenile idiopathic arthritis (JIA).

**Methods:** Consecutive patients with newly diagnosed JIA were included prospectively from defined geographic areas of the Nordic countries from 1997-2000. Inclusion criteria were availability of a baseline serum sample within 12 months after disease onset and eight-year clinical assessment data. Systemic onset JIA was not included. CRP was measured by high sensitive ELISA (detection limit of 0.2 mg/l).

**Results:** One hundred and thirty participants with a median follow-up time of 97 months (range 95-100) were included. At follow-up, 38% of the patients were in remission off medication. Absence of remission was associated with elevated level of CRP at baseline (odds ratio (OR): 1.33, confidence interval (CI): 1.08-1.63, p = 0.007). By applying a cutoff at the normal upper limit (>10 mg/l) the risk of not achieving remission was increased to an OR of 8.60 (CI: 2.98-24.81, p < 0.001). Variations of CRP within the normal range had no predictive impact on disease activity at follow-up.

Baseline levels of ESR were available in 80 patients (61%) and elevated ESR was associated with absence of remission in a multivariable logistic regression analysis (OR: 2.32, CI: 1.35-4.00, p = 0.002).

**Conclusion:** This results of this study indicate that baseline CRP concentrations above 10 mg/l are predictive of a poor outcome at eight-year follow-up. We could not demonstrate any predictive value of CRP variations within the normal range.

**Disclosure of Interest**

None Declared.

### P319 Single nucleotide polymorphisms in survivin gene are associated to response to methotrexate in juvenile idiopathic arthritis

#### Mojca Zajc Avramovič^1^, Vita Dolžan^2^, Nataša Toplak^1^, Tadej Avčin^1^

##### ^1^Department of Allergology, Rheumatology and Clinical Immunology, PAEDIATRIC CLINIC OF UNIVERSITY CLINICAL CENTRE LJUBLJANA, Ljubljana, Slovenia; ^2^Institute of Biochemistry, Medical Faculty, University of Ljubljana, Ljubljana, Slovenia

###### **Presenting author:** Mojca Zajc Avramovič

**Introduction:** Survivin is an anti-apoptotic protein and its circulating levels were associated with joint destruction and erosions in rheumatoid arthritis (RA). Recently it was suggested to be a marker of severe disease course in adult patients with RA as well as juvenile idiopathic arthritis (JIA). Single nucleotide polymorphisms (SNPs) in the survivin gene can affect the normal protein and its concentration and recently were shown to be associated with disease course in RA. Methotrexate is an important drug in treating JIA, but markers to predict its efficacy are needed.

**Objectives:** To evaluate the effect of SNPs in survivin gene on methotrexate efficacy in JIA.

**Methods:** The data of 116 consecutive patients with JIA treated with MTX at the University Children’s Hospital Ljubljana from June 2011 to January 2015 have been retrospectively reviewed. The disease activity was measured using JADAS 71 score. Non-responders were defined as patients not reaching 30% improvement in JADAS 71 score 6 months after the beginning of treatment with MTX. Genotyping of SNPs in the genes for survivin was performed using real time PCR methods. The following SNPs were analyzed: BIRC 5 G692C rs8073069, BIRC5 T241C rs17878467 and BIRC G31C rs9904341. Two-tailed Fisher exact test was used for statistical analysis.

**Results:** At 6 months 30/116 (25.8%) of patients were defined as non-responders. BIRC5 T241C rs17878467 (p < 0.0001) and BIRC G31C rs9904341 (p = 0.0272) were associated with achieving 30% improvement in JADAS 71 after 6 months. BIRC G31C rs9904341 was also associated with 70% improvement in JADAS 71 score (p < 0.0001) after 6 months of treatment.

**Conclusion:** Our results suggest that SNPs in survivin gene, BIRC5 T241C rs17878467 and BIRC G31C rs9904341 could be markers of MTX response in JIA. To our knowledge this is the first time SNPs in the survivin gene were analyzed in JIA.

**Disclosure of Interest**

None Declared.?>

### P320 Safety and effectiveness of adalimumab ± methotrexate for the treatment of polyarticular juvenile idiopathic arthritis (PJIA): STRIVE registry

#### N. Ruperto^1^, D. J. Lovell^2^, C. Wallace^2^, M. Toth^2^, I. Foeldvari^1^, J. Bohnsack^2^, D. Milojevic^2^, C. Rabinovich^2^, D. Kingsbury^2^, K. Marzan^2^, P. Quartier^3^, K. Minden^1^, E. Chalom^2^, G. Horneff^1^, R. M. Kuester^1^, J. Dare^2^, M. Heinrich^4^, H. Kupper^4^, J. Kalabic^4^, A. Martini^1^, H. I. Brunner^2^ and on behalf of PRINTO and PRCSG

##### ^1^PRINTO-IRCCS, Genova, Italy; ^2^PRCSG, Cincinnati, USA; ^3^Hôpital Necker-Enfants Malades, Paris, France; ^4^AbbVie Deutschland GmbH & Co. KG, Ludwigshafen, Germany

###### **Presenting author:** N. Ruperto

**Introduction:** JIA is the most common chronic inflammatory rheumatic diseases of childhood.

**Objectives:** To evaluate 6-year (y) safety and 2 y effectiveness profile of Adalimumab with or without methotrexate (ADA ± MTX) when used in current clinical practice for treatment of moderately to severely active pJIA.

**Methods:** This is a 6 y interim analysis of an ongoing, multicenter, non-interventional, observational registry of pts with moderately to severely active pJIA with up to10 y safety follow-up. Included pts are treated with ADA ± MTX or MTX alone as part of their routine clinical care enrolled in US, EU, and Australia. MedDRA observational adverse events (AEs) were recorded from first day in registry through last contact, irrespective of duration of registry treatment. Effectiveness was assessed by 27-joint juvenile arthritis disease activity score (JADAS27), based on CRP.

**Results:** As of Jan 2014, enrollment was complete. As of June 1, 2015 cut-off date, 846 pts (ADA ± MTX: 543 and MTX: 303) were treated in the registry; 39 pts rolled over from MTX to ADA ± MTX arm. At registry entry mean pJIA disease duration was 1.4 y and 3.7 y for MTX and ADA ± MTX arms, respectively. At baseline (BL), mean AJC71 was 5.8 and 5.3 for MTX and ADA ± MTX arms, respectively; Childhood Health Assessment Questionnaire Disability Index (CHAQ-DI) was 0.6 for both arms. At data cutoff, mean duration of study exposure was 1.81 y and 2.15 y for MTX and ADA ± MTX arms, respectively. Overall, 206 pts (68%) in MTX and 216 pts (39.8%) in ADA ± MTX arms discontinued registry drug through 6 y. Main reasons for registry drug discontinuation for MTX arm: pts required additional therapy (32.3%), other (11.9%), lack of efficacy (10.9%), AEs (8.3%), or pts achieved JIA remission (7.6%); for ADA ± MTX arm: lack of efficacy (16%), lost to follow-up (7.2%), other (5.9%), and AEs (5.3%). Frequencies and rates of treatment-emergent AEs were similar to those reported for observational AEs **(Table)**. There were no reports of deaths, malignancies, opportunistic infections, active TB, oral candidiasis, or CHF. Mean JADAS27(CRP) improved from 12.2 at BL to 9.7, 5.2, 4.4, 3.5, 2.2 at months 1, 6, and 12, 18, 24 for pts in MTX and from 11.8 at BL to 7.0, 4.-2, 4.2, 3.6, 3.9 in ADA ± MTX arms, respectively (observed data).

**Conclusion:** Overall, ADA ± MTX was well-tolerated in pJIA pts with no new safety signals observed. Discontinuations from registry drug were relatively high through 6 y, but greater in MTX only arm.

**Trial registration identifying number:** CT.gov: NCT00783510

**Disclosure of Interest**

N. Ruperto Consultant for: AbbVie Inc., AstraZeneca, Centocor, Bristol-Myers Squibb, Boehringer-Ingelheim, Pfizer, Regeneron, Hoffman La-Roche, Novartis, UCB, and Genentech, Speaker Bureau of: Genentech Pharmaceuticals, D. Lovell Consultant for: AbbVie Inc., AstraZeneca, Centocor, Bristol-Myers Squibb, Pfizer, Regeneron, Hoffman La-Roche, Novartis, UBC, Xoma, and Genentech, Amgen and Forest Research, Speaker Bureau of: Wyeth Pharmaceuticals, C. Wallace Grant / Research Support from: Pfizer and Amgen, Consultant for: Amgen and Novartis, M. Toth: None Declared, I. Foeldvari Consultant for: AbbVie and Novartis, J. Bohnsack Consultant for: Novartis, D. Milojevic Consultant for: Genentech and Novartis, C. Rabinovich Grant / Research Support from: UCB Pharma, Janssen Research & Development, LLC, Hoffmann-La Roche Inc., and AbbVie, D. Kingsbury Grant / Research Support from: AbbVie, K. Marzan Grant / Research Support from: AbbVie, P. Quartier Grant / Research Support from: AbbVie, Novartis, Pfizer, BMS, Chugai-Roche, Medimmune, Servier, and Swedish Orphan Biovitrum, Consultant for: AbbVie, Novartis, Pfizer, BMS, Chugai-Roche, Medimmune, Servier, and Swedish Orphan Biovitrum, K. Minden Grant / Research Support from: Pfizer and Abbvie, Consultant for: Pfizer, Abbvie, Roche/Chugai, Novartis, Medac and Pharma-Allergan, Speaker Bureau of: Pfizer, Abbvie, Roche/Chugai, Novartis, Medac and Pharma-Allergan, E. Chalom Speaker Bureau of: AbbVie, G. Horneff Grant / Research Support from: AbbVie, Pfizer, and Roche, Speaker Bureau of: AbbVie, Novartis, Pfizer, and Roche, R. Kuester Grant / Research Support from: AbbVie Inc. and Wyeth/Pfizer, J. Dare Grant / Research Support from: AbbVie, AstraZeneca, Bristol-Myers Squibb, Horizon Pharma, Medac, Pfizer, Roche and UCB, M. Heinrich Shareholder of: AbbVie, Employee of: AbbVie, H. Kupper Shareholder of: AbbVie, Employee of: AbbVie, J. Kalabic Shareholder of: AbbVie, Employee of: AbbVie, A. Martini Grant / Research Support from: AbbVie Inc., AstraZeneca, Bristol-Myers Squibb, Janssen Biologics B.V., Eli Lilly and Co., "Francesco Angelini", GlaxoSmithKline, Italfarmaco, Novartis, Pfizer, Roche, Sanofi Aventis, Schwarz Biosciences GmbH, Xoma, and Wyeth Pharmaceuticals, Employee of: GASLINI Hospital, Speaker Bureau of: Astellas, AstraZeneca, Bristol-Myers Squibb, Italfarmaco, and MedImmune, H. Brunner Consultant for: AbbVie Inc., AstraZeneca, Centocor, Bristol-Myers Squibb, Boehringer-Ingelheim, Pfizer, Regeneron, Hoffman La-Roche, Novartis, UCB, and Genentech, Speaker Bureau of: Genentech Pharmaceuticals.

**Table 32 (abstract P320).** See text for description

Table.	MTX		ADA ± MTX	
	N = 303 n (%)	PYs = 1014.6 E (E/100 PYs)	N = 543 n (%)	PYs = 1562.7 E (E/100 PYs)
Any AE	156 (51.5)	470 (46.3)	229 (42.2)	683 (43.7)
At least “possibly drug related” per the investigator	83 (27.4)	171 (16.9)	110 (20.3)	222 (14.2)
Severe AE	14 (4.6)	18 (1.8)	34 (6.3)	55 (3.5)
Serious AE	29 (9.6)	45 (4.4)	66 (12.2)	117 (7.5)
AE leading to discontinuation of study drug or study	25 (8.3)	33 (3.3)	35 (6.4)	56 (3.6)
Infectious AE	85 (28.1)	164 (16.2)	134 (24.7)	226 (14.5)
Serious infectious AE	12 (4.0)	15 (1.5)	26 (4.8)	36 (2.3)
Injection site-related AE	6 (2.0)*	8 (0.8)	28 (5.2)	37 (2.4)

*3 pts experienced injection site-related AEs with etanercept injections. During registry, 47 (15.5%) pts in MTX arm and 38 (7.0%) pts in ADA arm started with biologic DMARD other than ADA. All except one pt in MTX arm had been documented as permanently discontinued registry drug or registry, at time of cut-off date for this analysis.

### P321 Predictors of clinical remission with Etanercept in paediatric patients with extended oligoarticular, enthesitis-related arthritis and psoriatic arthritis: findings from the CLIPPER study

#### Alessandro Consolaro^1^, Gerd Horneff^1^, Ruben Burgos-Vargas^1^, Tamas Constantin^1^, Ivan Foeldvari^1^, Jelena Vojinovic^1^, Joke Dehoorne^1^, Violeta Panaviene^1^, Gordana Susic^1^, Valda Stanevica^1^, Katarzyna Kobusinska^1^, Zbigniew Zuber^1^, Richard Mouy^1^, Ingrida Rumba-Rozenfelde^1^, Pavla Dolezalova^1^, Chantal Job-Deslandre^1^, Nico Wulffraat^1^, Ronald Pederson^2^, Jack Bukowski^2^, Tina Hinnershitz^2^, Bonnie Vlahos^2^, Alberto Martini^1^, Nicolino Ruperto^1*^

##### ^1^Paediatric Rheumatology International Trials Organisation (PRINTO), Genoa, Italy; ^2^Pfizer, Collegeville, PA, USA

###### **Presenting author:** Nicolino Ruperto

**Introduction:** Etanercept (ETN) is approved in the EU for the treatment of children with juvenile idiopathic arthritis (JIA) categories of polyarticular, extended oligoarticular (eoJIA), enthesitis-related arthritis (ERA), and psoriatic arthritis (PsA), but little evidence is currently available regarding predictors of clinical remission.

**Objectives:** To evaluate characteristics that may have predicted the achievement of clinical remission with ETN in the CLIPPER study.

**Methods:** In this ongoing, Phase 3b, open-label study, paediatric patients with eoJIA (2–17 y), ERA (12–17 y), or PsA (12–17 y) received ETN 0.8 mg/kg once weekly (maximum, 50 mg) for up to 96 weeks. Baseline demographic and disease characteristics that were significantly different (p < 0.05) between children in remission or with active disease were analysed post hoc as categorical predictors (i.e., dichotomized continuous characteristics) in univariate logistic regression models; response and disease activity status after 12 weeks of ETN treatment were also considered as predictive factors. Clinical remission was defined with the JIA ACR Wallace 2011 remission criteria or Juvenile Arthritis Disease Activity Score 71-joint reduced count (JADAS71) clinical remission criteria (≤1) persisting for ≥24 weeks.

**Results:** Of the 127 patients enrolled in the trial, 42 (33%) and 54 (43%) achieved JIA ACR or JADAS71 clinical remission over 24 weeks, respectively. In univariate analyses, patients who had lower BMI and were shorter at baseline and those who were younger at the time of disease onset were significantly more likely to achieve JIA ACR and JADAS71 remission (table). Age >11 vs ≤11 years at baseline and C-reactive protein level >2.4 mg/L vs ≤2.4 were significant predictors of JIA ACR remission; HLA-B27+ vs HLA-B27– status and >11 vs ≤11 joints with limitation of motion at baseline were predictors of JADAS71 remission. Induction of JIA ACR and JADAS71 responses at 12 weeks was also predictive of sustained ACR and JADAS71 responses for 24 weeks.

**Conclusion:** Clinical remission after 12 weeks of etanercept treatment was the most important predictor of sustained clinical response over 24 weeks in paediatric patients with eoJIA, ERA, or PsA JIA subtypes.

**Trial registration identifying number:** NCT00962741/NCT01421069

**Disclosure of Interest**

A. Consolaro: None Declared, G. Horneff Grant / Research Support from: AbbVie, Bristol-Myers Squibb, Novartis, Pfizer and Roche, R. Burgos-Vargas Speaker Bureau of: Abbvie, Janssen, Pfizer, and UCB, T. Constantin Consultant for: Abbvie, Novartis, Pfizer and Roche, Speaker Bureau of: Abbvie, Novartis, Pfizer and Roche, I. Foeldvari Consultant for: Novartis, Chugai, Beyer and Roche, J. Vojinovic Speaker Bureau of: Abbvie, Roche, Pfizer, MSD and TEVA, J. Dehoorne Consultant for: AbbVie, V. Panaviene: None Declared, G. Susic: None Declared, V. Stanevica Grant / Research Support from: Pfizer, Consultant for: AbbVie, Roche, K. Kobusinska: None Declared, Z. Zuber: None Declared, R. Mouy Grant / Research Support from: Pizer and Novartis, I. Rumba-Rozenfelde Grant / Research Support from: Pfizer, P. Dolezalova Grant / Research Support from: AbbVie, Roche, Medac, Novartis, Pfizer, Consultant for: Roche, Speaker Bureau of: Pfizer, Novartis, Medac, C. Job-Deslandre: None Declared, N. Wulffraat Grant / Research Support from: AbbVie, Roche, Sobi, Consultant for: Novartis, Pfizer, Sobi, R. Pederson Shareholder of: Pfizer, Employee of: Pfizer, J. Bukowski Shareholder of: Pfizer, Employee of: Pfizer, T. Hinnershitz Shareholder of: Pfizer, Employee of: Pfizer, B. Vlahos Shareholder of: Pfizer, Employee of: Pfizer, A. Martini Grant / Research Support from: Abbott, Abbvie, Amgen, Biogen Idec, Bristol-Myers Squibb, Astellas, Boehringer, Italfarmaco, Janssen, MedImmune, Novartis, Novo Nordisk, Pfizer, Sanofi, Roche, Servier, Takeda, Speaker Bureau of: Abbott, Abbvie, Amgen, Biogen Idec, Bristol-Myers Squibb, Astellas, Boehringer, Italfarmaco, Janssen, MedImmune, Novartis, Novo Nordisk, Pfizer, Sanofi, Roche, Servier, Takeda, N. Ruperto Grant / Research Support from: Abbott, AbbVie, Amgen, Biogen Idec, Astellas, Alter, AstraZeneca, Boehringer, Bristol-Myers Squibb, CD-Pharma, Celgene, Crescendo Bioscience, EMD Serono,Hoffman-La Roche, Italfarmaco, Janssen, MedImmune, Medac, Novartis, Novo Nordisk, Pfizer, Sanofi Aventis, Servier, Takeda, Vertex, Speaker Bureau of: Abbott, AbbVie, Amgen, Biogen Idec, Astellas, Alter, AstraZeneca, Boehringer, Bristol-Myers Squibb, CD-Pharma, Celgene, Crescendo Bioscience, EMD Serono,Hoffman-La Roche, Italfarmaco, Janssen, MedImmune, Medac, Novartis, Novo Nordisk, Pfizer, Sanofi Aventis, Servier, Takeda, Vertex.

**Table 33 (abstract P321).** Significant predictors of sustained clinical responses to ETN for 24 weeks

Baseline characteristic	Sustained JIA ACR remission		Sustained JADAS71 remission	
	n/N (%)	Odds Ratio (95% CI)	n/N (%)	Odds Ratio (95% CI)
BMI, kg/m^2^		>18 vs ≤18		>18 vs ≤18
- ≤18	23/46 (50)	0.31 (0.14, 0.66)	26/46 (57)	0.41 (0.19, 0.85)
- >18	19/81 (24)		28/81 (35)	
Height, cm		>158 vs ≤158		>158 vs ≤158
- ≤158	28/56 (50)	0.25 (0.11, 0.54)	31/56 (55)	0.39 (0.19, 0.80)
- >158	14/71 (20)		23/71 (32)	
Age at onset, mo				
- ≤91	24/41 (59)	>91 vs ≤91	23/41 (56)	>91 vs ≤91
- >91	18/86 (21)	0.19 (0.08, 0.42)	31/86 (36)	0.44 (0.21, 0.94)
12-week clinical outcome				
JIA ACR status		Remission vs active disease		Remission vs active disease
- Remission	11/15 (73)	6.83 (2.02, 23.09)	12/15 (80) 42/108 (39)	6.29 (1.67, 23.60)
- Active disease	31/108 (29)			
JADAS71 status		Remission vs active disease		Remission vs active disease
- Remission	14/18 (78)	9.72 (2.94, 32.11)	14/18 (78) 39/102 (38)	5.65 (1.74, 18.41)
- Active disease	27/102 (27)			

### P322 Serum and plasma MRP8/14 concentrations at disease onset of juvenile idiopathic arthritis in predicting need of medication at one year after diagnosis

#### Paula Keskitalo^1^, Salla Kangas^2^, Paula Vähäsalo^1^

##### ^1^Department of Pediatrics, Oulu University Hospital and University of Oulu, Oulu, Finland; ^2^Medical Research Center Oulu, Oulu University Hospital and University of Oulu, Oulu, Finland

###### **Presenting author:** Paula Keskitalo

**Introduction:** Juvenile idiopathic arthritis (JIA) is a chronic inflammatory joint disease affecting children. At the diagnosis it is difficult to predict disease course. Myeloid-related protein /14 complex (MRP 8/14) level in blood has been suggested to function as a biomarker for predicting response to medication and relapse rate in JIA patients.

**Objectives:** The aim of our prospective, observational cohort study was to analyze whether MRP8/14 levels at the time of diagnosis of JIA can predict disease course at one year after, and to compare plasma and serum samples in prediction.

**Methods:** Serum and plasma MRP8/14 concentrations from 88 patients with established new-onset JIA and from 51 healthy controls were measured by human calprotectin ELISA kit (Hycult biotech). Clinical data were collected during the visits of the patients in the pediatric rheumatology outpatient clinic in Oulu University Hospital (Oulu, Finland) between October 2011 and November 2014. Continuous variables were described as mean and 95% confidence intervals (95% CI). Independent sample t-test and analysis of variance were used to compare continuous variables.

**Results:** Study population consists of 88 new-onset JIA patients (70.5% females) and 51 (45.1% females) healthy controls. Mean age was 6.6 years in the JIA and 8.6 years in the control group (P = 0.008). MRP8/14 levels in serum were significantly higher in the JIA patients (257.3 ng/mL (95% CI: 232.9 - 281.7)) compared to controls (119.9 ng/mL (95% CI: 94.7 - 145.2), P < 0.001). Also MRP8/14 levels in plasma were higher in the JIA patients (228.8 ng/mL (95% CI: 189.2 - 268.3)) compared to the controls (43.7 ng/mL (95% CI: 31.1 - 56.2)), P < 0.001). MRP8/14 levels in plasma and serum were compared between following JIA categories: persistent and extended oligoarthritis (33% and 4.5%), seronegative and seropositive polyarthritis (49% and 3.5%), enthesis-related arthritis (9%) and psoriatic arthritis (1%). The patients with seronegative polyarthritis had significantly higher MRP8/14 serum levels (293.2 ng/mL (95% CI: 257.4 - 328.9)) than patients with persistent oligoarthritis (195.3 ng/mL (95% CI: 160.3 - 230.4), P = 0.002) and respectively higher plasma levels (301.6 ng/mL (95% CI: 257.4 - 328.9) vs 195.3 ng/mL (95% CI: 160.3 - 230.4), P = 0.001).

In average one year after diagnosis (mean 389 days), 34.2% of JIA patients were followed without any disease-modifying antirheumatic drug (DMARD), 54.1% were on synthetic DMARDs (sDMARD) and 11.8% were on biological DMARDs (bDMARD). When we analyzed MRP8/14 levels at the disease onset in those medication groups at one year, we found a significant difference between the non-medicated and the sDMARD groups in serum (209.1 ng/mL (95% CI: 177.3 – 240.8) vs 297.1 ng/mL (95% CI: 260.8 – 333.2), P = 0.003) and in plasma (150.1 ng/mL (95% CI: 102.7 – 197.4) vs 287.4 ng/mL (95% CI: 226.1 – 348. 6), P = 0.004). Between bDMARD users in serum (267.1 ng/mL (95% CI: 192.5 – 341.5)) and in plasma (249.6 ng/mL (95% CI: 113.7 – 385.4) and sDMARD or no-medication group we didn´t find any significant differences in MRP8/14 levels.

**Conclusion:** Serum and plasma MRP8/14 levels established at new-onset of JIA seem to predict the disease course one year later. Both serum and plasma samples can be used in prediction of needed medication.

**Disclosure of Interest**

None Declared.

### P323 CD4+ T cell DNA methylation in oligoarticular juvenile idiopathic arthritis stratified by age at diagnosis, and persistent or extended disease course

#### Raul A. Chavez Valencia^1^, David Martino^1^, Jane Munro^2^, Anne-Louise Ponsonby^1^, Rachel Chiaroni-Clarke^1^, Braydon Meyer^1^, Roger C. Allen^2^, Jonathan D. Akikusa^2^, Jeffrey M. Craig^1^, Richard Saffrey^1^, Justine A. Ellis^1^

##### ^1^Murdoch Childrens Research Institute, Melbourne, Australia; ^2^Royal Childrens Hospital, Melbourne, Australia

###### **Presenting author:** Raul A. Chavez Valencia

**Introduction:** Significant disease heterogeneity exists amongst children diagnosed with oligoarticular juvenile idiopathic arthritis (oJIA). Sources of heterogeneity might include age at diagnosis, and persistent vs extended disease (≤4 or >4 joints 6 months post diagnosis respectively). DNA methylation is an epigenetic modification, and is able to sub-classify cases in other immune mediated diseases. However, use of DNA methylation as a biomarker to aid sub-classification or prognosis has not been investigated in JIA.

**Objectives:** We explored whether differences to CD4+ T cell DNA methylation are associated with oJIA, and whether these might be impacted by case heterogeneity due to age at diagnosis, or disease extension.

**Methods:** 57 oJIA cases and 57 age- and sex-matched controls were selected from the CLARITY biobank [1]. One case was later removed due to inactive disease at the time of blood collection. Remaining cases had active disease, and were unexposed to biological disease modifying anti-rheumatic drugs or methotrexate. 35 cases were diagnosed at ≤6 years, and 21 were diagnosed at >6 years of age. 39 cases were diagnosed with persistent, and 17 with extended oJIA. Of these 17 extended cases, 11 had blood collected prior to disease extension. CD4+ T-cells were flow sorted from stored peripheral blood mononuclear cells, and genomic DNA extracted. Bisulphite converted DNA was then applied to Illumina HumanMethylation450 arrays to generated genome-scale DNA methylation data. Data was pre-processed using Minfi [2], and differential methylation analysis carried out using RUV [3]. Covariates were identified and adjusted for using empirically detected negative control probes. A false discovery rate (FDR) adjusted p value of 0.1 or less was considered supportive of differential methylation. Differences in methylation (∆β) values were calculated. Cluster analysis using multidimensional scaling was used to identify principal components of the data.

**Results:** Comparing all oJIA cases to all controls, no significant differences in DNA methylation were identified. Interestingly, the first principal component of the data was defined by age. Stratifying by age at diagnosis, case-control comparison of young pairs identified one significantly different probe (p_adj_ = 0.01, ∆β = -3.4%), located in the enhancer region of a long non-coding RNA. Another probe located in an immune related gene reached near significance (p_adj_ = 0.3, ∆β = -1.2%). No probes were identified as significantly different in the older diagnosed analysis.

Comparing persistent to extended cases, the top differentially methylated array probe was near significant (p_adj_ = 0.19, ∆β = 4.8%). Cluster analysis using as few as 58 array probes identified extended oJIA cases prior to extension.

**Conclusion:** Sites of differential case-control methylation identified for younger, but not older diagnosed oJIA may provide insight into differing disease processes and assist with future refinement of JIA subtype classification. Additionally, DNA methylation differences that are detectable prior to disease extension may hold promise as biomarkers of disease prognosis.

1. Ellis, J.A., et al., *CLARITY - ChiLdhood Arthritis Risk factor Identification sTudY.* Pediatr Rheumatol Online J, 2012. **10**:37

2. Aryee, M.J., et al., *Minfi: a flexible and comprehensive Bioconductor package for the analysis of Infinium DNA methylation microarrays.* Bioinformatics, 2014. **30**:1363-9

3. Maksimovic, J., et al., *Removing unwanted variation in a differential methylation analysis of Illumina HumanMethylation450 array data.* Nucleic Acids Res, 2015. **43**:106

**Disclosure of Interest**

None Declared.

### P324 Change in expert consensus based on availability of additional laboratory data over the course of MAS

#### Sergio Davì^1^, Francesca Minoia^1^, AnnaCarin Horne^2^, Nico Wulffraat^3^, Carine Wouters^4^, Carol Wallace^5^, Yosef Uziel^6^, Gary Sterba^7^, Rayfel Schneider^8^, Ricardo Russo^9^, Athimalaipet V. Ramanan^10^, Jana Pachlopnik Schmid^11^, Seza Ozen^12^, Kim E Nichols^13^, Paivi Miettunen^14^, Daniel J. Lovell^15^, Kai Lehmberg^16^, Toshiyuki Kitoh^17^, Raju Khubchandani^18^, Norman T. Ilowite^19^, Jan-Inge Henter^2^, Alexei A Grom^15^, Fabrizio De Benedetti^20^, Edward M. Behrens^21^, Tadej Avcin^22^, Maurizio Aricò^23^, Alberto Martini^1^, Nicolino Ruperto^1^, Randy Q. Cron^24^, Angelo Ravelli^1^

##### ^1^Istituto Giannina Gaslini, Genoa, Italy; ^2^Karolinska Institute, Stockholm, Sweden; ^3^Wilhelmina Children’s Hospital, Utrecht, Netherlands; ^4^University Hospital Leuven, Leuven, Belgium; ^5^Seattle Children’s Hospital, Seattle, USA; ^6^Meir Medical Centre, Kfar Saba, Israel; ^7^Mount Sinai Medical Center, Miami, USA; ^8^Hospital for Sick Children, Toronto, Canada; ^9^Hospital de Pediatria Juan P Garrahan, Buenos Aires, Argentina; ^10^Bristol Royal Hospital for Children, Bristol, UK; ^11^University Children’s Hospital, Zurich, Switzerland; ^12^Hacettepe University, Ankara, Turkey; ^13^14St Jude Children’s Research Hospital, Memphis, USA; ^14^University of Calgary, Calgary, Canada; ^15^Cincinnati Children’s Hospital Medical Center, Cincinnati, USA; ^16^University Medical Center, Hamburg, Germany; ^17^Aichi Medical University, Nagakute, Japan; ^18^Jaslok Hospital and Research Centre, Mumbai, India; ^19^Albert Einstein College of Medicine and Children’s Hospital at Montefiore, New York, USA; ^20^Ospedale Pediatrico Bambino Gesù, Rome, Italy; ^21^The Children’s Hospital of Philadelphia, Philadelphia, USA; ^22^University Children’s Hospital, Ljubljana, Slovenia; ^23^Azienda Sanitaria Provinciale 7, Ragusa, Italy; ^24^University of Alabama at Birmingham, Birmingham, USA

###### **Presenting author:** Sergio Davì

**Introduction:** The 2016 classification criteria for macrophage activation syndrome (MAS) complicating systemic juvenile idiopathic arthritis (sJIA) have recently been published. In the course of the project, 28 experts were asked to classify a number of patient profiles as having or not having MAS, based on clinical and laboratory features recorded at MAS onset or on change in laboratory values between last visit before MAS and MAS onset. There was, however, a high proportion of patients diagnosed by the treating physician as having MAS, but classified by the experts as not having MAS. This discordance could be due to the lack of awareness by the experts of information about patients’ clinical course over time, which was available to the treating physician.

**Objectives:** To investigate whether the accessibility to additional information about course over time increased the number of patients diagnosed as MAS by the experts.

**Methods:** 26 of the 28 experts involved in the MAS Classification Criteria project agreed to re-evaluate the profiles of discordant patients enriched with additional information about course over time of laboratory tests. For 28 patients originally assessed on the basis of on clinical and laboratory features at MAS onset, laboratory values at visit pre-MAS and change in values between visit pre-MAS and MAS onset were provided. For 41 patients originally evaluated on the basis of change in values between visit pre-MAS and MAS onset, laboratory values at full-blown stage of MAS were provided.

**Results:** In the first set of profiles, the experts did not change the diagnosis in 66.2% of patients, changed the diagnosis to MAS in 18.4% of patients, and changed the diagnosis to non-MAS in 9.1% of patients. In the second set of profiles, 58.7% of patients had unchanged diagnosis, 36.5% had diagnosis changed to MAS, and 2.1% had diagnosis changed to non-MAS. Overall, 8 patients previously diagnosed as non-MAS with >80% consensus and 1 patient with no consensus among experts had the diagnosis changed to MAS with >80% consensus. In addition, 26 patients previously diagnosed as non-MAS with >80% consensus did not achieve >80% consensus as non-MAS.

**Conclusion:** The availability of additional laboratory data over the course of MAS led the experts to change their diagnosis for a number of patients. Our analysis confirms that the change in laboratory values over time may be more relevant for making a timely diagnosis of MAS than the absolute values at a single point in time.

**Disclosure of Interest**

None Declared.

### P325 Oral health and anti-citrullinated peptide antibodies (ACPA) in juvenile idiopathic arthritis

#### Sriharsha Grevich^1,2^, Peggy Lee^3^, Sarah Ringold^1,2^, Brian Leroux^3^, Hannah Leahey^4^, Megan Yuasa^4^, Jessica Foster^4^, Jeremy Sokolove^5^, Lauren Lahey^6^, William Robinson^5^, Joshua Newson^4^, Anne Stevens^1,2,4^

##### ^1^Pediatrics, University of Washington, Seattle, WA, USA; ^2^Seattle Children's Hospital, Seattle, WA, USA; ^3^School of Dentistry, University of Washington, Seattle, WA, USA; ^4^Seattle Children's Research Institute, Seattle, WA, USA; ^5^Medicine, Stanford University, Stanford, CA, USA; ^6^Stanford University, Stanford, CA, USA

###### **Presenting author:** Sriharsha Grevich

This abstract is not included here as it has already been published.

### P326 Are parent, adolescent and adult measures of functional ability comparable in adolescents with juvenile idiopathic arthritis?

#### Stephanie J. W. Shoop^1,2^, Kimme L. Hyrich^1,2^, Suzanne M. M. Verstappen^1^, Wendy Thomson^2,3^, Janet E. McDonagh^4^ and CAPS

##### ^1^Arthritis Research UK Centre for Epidemiology, The University of Manchester, Manchester, UK; ^2^NIHR Manchester Musculoskeletal Biomedical Research Unit, Central Manchester University Hospitals NHS Foundation Trust and University of Manchester Partnership, Manchester, UK; ^3^Arthritis Research UK Centre for Genetics and Genomics, The University of Manchester, Manchester UK; ^4^Centre for MSK Research, Faculty of Medical and Human Sciences, The University of Manchester, Manchester, UK

###### **Presenting author:** Sriharsha Grevich

**Introduction:** Measuring functional ability in adolescents with juvenile idiopathic arthritis (JIA) presents a unique problem. The questions on the parent-assessed Childhood Health Assessment Questionnaire (P-CHAQ) are not always applicable to adolescents and the adolescent version (A-CHAQ) has not been formally validated. Since adolescence parallels transfer to adult care, use of the adult Health Assessment Questionnaire (HAQ) may be preferable to continuously capture a functional ability measure over this period. However, it is unclear how the HAQ compares with the two CHAQ tools.

**Objectives:** To compare the agreement between the P-CHAQ, A-CHAQ and HAQ in adolescents with JIA at initial presentation to rheumatology.

**Methods:** Adolescents aged 11 to 17 years were recruited as part of a UK multicentre inception cohort of young people with juvenile-onset arthritis: the Childhood Arthritis Prospective Study (CAPS). Adolescents met inclusion criteria for analysis if they had a physician’s diagnosis of JIA and had themselves completed the A-CHAQ and HAQ and their guardian completed a P-CHAQ at baseline. Wilcoxen signed-rank tests were used to compare median scores, pairwise correlations between the scores were assessed using Spearman’s correlations and percent agreement was assessed with agreement defined as scores within 0.25 points.

**Results:** A total of 107 adolescents had complete data on all three functional ability measures. Median age at diagnosis was 13 years (IQR 12 to 15) and 61% of the cohort were female. Median disease duration at diagnosis was seven months (IQR 5 to 14) and the most common subtype was oligoarticular JIA (40%).

Median scores on the two CHAQ measures were similar at 0.625 (both IQR 0.125 to 1.25, p = 0.97). However, the median HAQ was lower, at 0.5 (IQR 0 to 1.125, p < 0.001 compared to A-CHAQ and P-CHAQ). Strong correlations were observed between all pairs of functional ability scores. Despite having marginally different median scores, the strongest correlation was between the HAQ and the A-CHAQ (0.91), with the lowest between the two CHAQ tools (0.83) (Table 34).

There was relatively high agreement between scores, with the highest agreement between the two CHAQ tools (78%) and lowest between the HAQ and P-CHAQ (71%). Where discordant, the majority of HAQ scores fell below those from either CHAQ. Discordance between CHAQ scores was more evenly distributed with 12% P-CHAQ scores falling below and 10% exceeding C-CHAQ scores, respectively (Table 34).

**Conclusion:** There was strong correlation and good concordance between the P-CHAQ, A-CHAQ and HAQ in adolescents with JIA. The strong correlations and concordances between the HAQ and either CHAQ tool indicate the utility of HAQ in adolescents with JIA. Further work is needed to understand which domains drive the lower HAQ scores in this population.

**Disclosure of Interest**

None Declared.

**Table 34 (abstract P326).** Comparisons of the P-CHAQ, A-CHAQ and HAQ in adolescents with JIA

Score	Median score (IQR)	Comparison	Correlation	Percent agreement (%)	Percent discordant scores (%)	
					Higher	Lower
P-CHAQ	0.625 (0.125, 1.25)	Vs. A-CHAQ	0.83	78	12	10
A-CHAQ	0.625 (0.125, 1.25)	Vs. HAQ	0.91	74	22	5
HAQ	0.5 (0, 1.125)	Vs. P-CHAQ	0.86	71	7	22

### P327 Clinical characteristics of the initial patients enrolled in the childhood arthritis and rheumatology research alliance (CARRA) registry

#### Timothy Beukelman^1^, Yuki Kimura^2^, Marc Natter^3^, Norm Ilowite^4^, Kelly Mieszkalski^5^, Grendel Burrell^5^, Brian Best^5^, Helen Bristow^6^, Shannon Carr^6^, Anne Dennos^6^, Rachel Kaufmann^6^, Laura Schanberg^6^ and for the CARRA Registry Investigators

##### ^1^University of Alabama at Birmingham, Birmingham, AL, USA; ^2^Hackensack University Medical Center, Hackensack, NJ, USA; ^3^Harvard, Boston, MA, USA; ^4^Montefiore, New York, NY, USA; ^5^CARRA, Durham, NC, USA; ^6^Duke, Durham, NC, USA

###### **Presenting author:** Timothy Beukelman

This abstract is not included here as it has already been published.

### P328 A multicentric study for the validation of the chronic arthitis score (CASCO) among a cohort of children with musculoskeletal complaints

#### Ilaria Parissenti^1^, Antonella Insalaco^2^, Andrea Taddio^3^, Angela Mauro^4^, Manuela Pardeo^2^, Francesca Ricci^1^, Gabriele Simonini^4^, Marco Cattalini^1^

##### ^1^Clinical and Experimental Sciences, Pediatric Clinic, University of Brescia, Brescia, Italy; ^2^Pediatric Rheumatology, Ospedale Pediatrico Bambino Gesù, Rome, Italy; ^3^Institute of Child and Maternal Health, IRCCS "Burlo Garofolo" and University of Trieste, Trieste, Italy; ^4^Pediatric Rheumatology, Anna Meyer Children's Hospital, University of Florence, Florence, Italy

###### **Presenting author:** Marco Cattalini

**Introduction:** Musculoskeletal pain is a common complaint in children, but the differential diagnosis may cover a wide range of diseases, and only a minority of patients will turn out to have a chronic inflammatory condition.

**Objectives:** We recently developed a score (CASco) to calculate the probability a patients with musculoskeletal complaints will receive a diagnosis of chronic arthritis^1^. To validate this score we performed this study. As a secondary objective we analyzed the distribution in our cohort of the different causes and manifestations of musculoskeletal pain.

**Methods:** 199 patients referred for muskuloskeletal complaints in a time frame of 6 months were recruited. At the time of recruitment we applied a standardized questionnaire to collecte detailed clinical information, focusing on the joint swelling pattern, the precipitating factors of pain, the duration of morning stiffness and the frequency of pain, which are the variables that had previously been shown to be significantly associated with a positive CASco. The score was calculated by I.P. while the other investigators were blinded to the results of the score. Once the final diagnosis was confirmed we compared the result of the CASco score with the clinical diagnosis, to calculate the sensibility and sensitivity of the score.

**Results:** The final diagnosis were distributed as follows: chronic inflammatory arthritis (17%), infections-related arthritis (17%) and non-inflammatory disorders (66%). *Pain frequency*: Joint pain was recorded in 92% of patients. The patients with chronic arthritis showed a dichotomic pain distribution (persistent pain: 62%; absence of pain: 23%), while the presence of recurrent pain with more than one episode per month was significantly associated with non-inflammatory disorders and the single episode of pain resulted the typical presentation of children with infections-related arthritis. *Pain precipitating factors*: rest was associated with chronic arthritis, a prior infection with children with infections-related arthritis and activity with non-inflammatory disorders. *Joint swelling pattern*: 74% of children with chronic arthritis had daily persistent joint swelling in one or more joints. By contrast, in the other groups the clinical presentation at onset was characterized in most cases by the absence of joint swelling (56% of children with infections-related arthritis and 80% of children with non-inflammatory disorders). *Morning stiffness*: was reported more frequently in patients with chronic arthritis (53% of patients) compared with the other two groups (15% and 8%). All the mentioned differences were statistically significant (p < 0.001). Among children with chronic arthritis who suffered from morning stiffness, this symptom usually lasted for less than one hour (33%). In this data set the predictive score correctly identified 185/199 patients with a precision of 93%. The Score revealed high sensitivity (88%) and specificity (94%).

**Conclusion:** we confirmed the CASco score as an instrument with good sensitivity and specificity. Of course, the CASco is not intended as a substitute of a clinical evaluation, still its use in the daily clinical routine may help the pediatrician to correctly address the differential diagnosis in a child with musculoskeletal complaints, rationalizing time and resources and promptly identifying those patients who would benefit the most from a pediatric rheumatology evaluation.

**Reference**

^1^ Cattalini M, Parissenti I, Tononcelli E, Lancini F, Cantarini L, Meini A. Developing a Predictive Score for Chronic Arthritis among a Cohort of Children with Musculoskeletal Complaints-The Chronic Arthritis Score Study. J Pediatr. 2015 Nov 23.

**Disclosure of Interest**

None Declared.

### P329 Correct identification of postural problems as a cause of musculoskeletal complaints

#### Paola Montesano^1^, Ilaria Parissenti^1^, Francesca Ricci^1^, Barbara Bonafini^1^, Veronica Medeghini^1^, Francesca Lancini^2^, Marco Cattalini^1^

##### ^1^Clinical and Experimental Sciences, Pediatric Clinic, University of Brescia, Brescia, Italy; ^2^Physiatry, Spedali Civili di Brescia, Brescia, Italy

###### **Presenting author:** Marco Cattalini

**Introduction:** Musculoskeletal pain is one of the most common complaints in the pediatric population, affects between 10% and 20% of children and it is one of the leading reasons of referring to pediatric rheumatologist. The most common origin of the pain is a non inflammatory condition, including orthopedic diseases, hypermobility, “growing pains” and postural disorders. Recently, we developed a score, called CASco^1^, that can determine the probability a child with musculoskeletal complaints will receive a final diagnosis of inflammatory arthritis.

**Objectives:** The aim of our study was to verify if a pediatric rheumatologist would be able to correctly diagnose those patients with musculoskeletal complaints secondary to postural abnormalities

**Methods:** All children referred for musculoskeletal complaints, with a medical history suggestive for a "mechanical" origin of the pain were recruited. The CASco score was calculated and the patients went through a standardazied evaluation to detect postural problems. All the patients were then referred to the physiatrist, who was considered the "gold standard" for the diagnosis of postural problems

**Results:** We enrolled 14 children referred to our pediatric rheumatology department for musculoskeletal. The postural examination confirmed the presence of postural alterations in 10/14 children. The CASco score was negative (i.e. not indicative for inflammatory arthritis) in 12/14 patients. All these patients were finally referred to the physiatrist that found alterations at physical evaluation and reach a final diagnosis of postural disorders in 14/14 children.

**Conclusion:** The study is still in progress, but the preliminary results suggest that pediatricians still need to develop proper skills to identify postural problems, that may be a cause of musculoskeletal complaints. The use of the CASco score, as a support instrument to rule out inflammatory disorders, may be of valuable help to the pediatrician. With all the proper instruments pediatricians, and pediatric rheumatologists among them, will be able to precociously differentiate patients with musculoskeletal complaints due to postural problem, and refer them to the physiatrist.

^1^ Cattalini M et al. “Developing a Predictive Score for Chronic Arthritis among a Cohort of Children with Musculoskeletal Complaints-The Chronic Arthritis Score Study”, J Pediatr. 2016 Feb;169:188-93

**Disclosure of Interest**

None Declared.

### P330 Long-term efficacy and safety of biologic therapy in children with juvenile idiopathic arthritis or non-infectious uveitis

#### Margaux Gerbaux^1^, Phu-Quoc Lê^1^, Laurence Goffin^1^, Valérie Badot^2^, Céline La^2^, Laure Caspers^3^, François Willermain^3^, Alina Ferster^1^

##### ^1^Department of Hematology and Oncology, Hôpital Universitaire Des Enfants Reine Fabiola, Université Libre de Bruxelles, Brussels, Belgium; ^2^Department of Rheumatology, Hôpital Erasme, Université Libre de Bruxelles, Brussels, Belgium; ^3^Department of Ophtalmology, Hôpital Saint Pierre, Université Libre de Bruxelles, Brussels, Belgium

###### **Presenting author:** Margaux Gerbaux

**Introduction:** Juvenile idiopathic arthritis (JIA) and idiopathic uveitis are rare diseases associated with severe complications. Currently, in addition to the standard treatment strategies, biological agents result in a major step in the improvement of their management.

**Objectives:** The aim of this study is to assess long-term efficacy and safety of biologic therapy in children with JIA or non-infectious uveitis refractory to first-line immunosuppression treatment.

**Methods:** This is a retrospective study on children (16 years or younger) with JIA or non-infectious uveitis treated with biological agents between 2000 and 2015 in one pediatric hemato-oncology unit (Hôpital Universitaire Des Enfants Reine Fabiola, Brussels). Patients could receive more than one type of biologic agent during the follow-up.

JIA disease activity was evaluated (at baseline, after 6 months, 15 months, 2 years and then yearly) according to a modified pediatric ACR (Pedi ACR) core set criteria with 3 items (the level of C-reactive protein (CRP), the number of active joints and the number of limited joints) and by comparison of the duration of morning stiffness, the number of tender joints and the erythrocyte sedimentation rate (ESR). The improvement of intraocular inflammation was primarily assessed by anterior chamber cells (Tyndall grade) according to the definition of the Standardization of Uveitis Nomenclature (SUN) criteria but also according to the degree of inflammation in intermediate and posterior chamber and by the number of flares. Adverse events were classified according to the Common Terminology Criteria for Adverse Events.

**Results:** Of the 29 patients included, 24 had JIA (11 associated with uveitis), 4 had idiopathic uveitis and 1 had uveitis associated with Behçet’s disease. Median age at diagnosis was 50 months (range: 29-96) and mean age at the start of biologic treatment was 100 months (SD: 50).

26 patients were treated with anti-TNF (Etanercept, Infliximab or Adalimumab), 2 with Abatacept, 2 with Canakimumab and 4 with Tocilizumab. All patients had been previously treated with methotrexate. The median duration of biological treatment was 45 months (range: 21-82).

The rate of patient’s improvement was encouraging with 85/70/60/45%, 85/75/75/65%, 83/83/78/61%, 93/87/80/80%, 92/83/83/75% and 91/82/82/82% according to the modified Pedi ACR core set criteria 30/50/70/90 and 64, 62, 82, 63, 67 and 67% according to Tyndall at 6 months, 15 months, 2, 3, 4 and 5 years. Individual data regarding articular condition (duration of morning stiffness, number of active, tender and limited joints) decreased significantly (p < 0.05) at each evaluation until 4 years of treatment. The level of CRP and ESR decreased significantly (p < 0.05) at each evaluation, from 6 month to 7 years. The number of flares as well as the level of inflammation in anterior, intermediate and posterior chamber at 6 months, 15 months and 2 years of treatment decreased significantly (p < 0.05). The rate of patient achieving both articular and ophthalmic remission was 65%.

Severe adverse events occurred in 17% of the patients and were reversible for 80% of them.

**Conclusion:** In our studied population, biologic therapy is effective and safe. Their effects occur early and persist in long-term evaluation. American and European authorities approved these treatments in JIA but not yet in non-infectious uveitis. Additional data with longer follow-up and larger number of patients as well as randomized studies in uveitis are required.

**Disclosure of Interest**

None Declared.

### P331 Kaposiform hemangioendothelioma arising in the leg mimicking juvenile idiopathic arthritis

#### Maria Ceci^1^, Francesco Licciardi^1^, Marco Turco^1^, Francesca Santarelli^2^, Davide Montin^2^, Claudia Toppino^2^

##### ^1^Department of Pediatrics, University of Turin, Regina Margherita Children's Hospital, Turin, Italy; ^2^Department of Pediatrics, University of Turin, Regina Margherita Children's Hospital, Turin, Italy

###### **Presenting author:** Maria Ceci

**Introduction:** Kaposiform hemangioendothelioma (KHE) is a rare vascular neoplasia that usually arises in children as a superficial or deep soft tissue mass of the extremities with variable clinical presentation. KHE typically presents as an ill-defined, red to purple, indurated plaque and is often complicated by the Kasabach–Merritt phenomenon (KMP), a condition of severe thrombocytopenia and consumptive coagulopathy.

**Objectives:** Herein we describe a pediatric patient with atypical KHE sent to rheumatological evaluation for suspected Juvenile Idiopathic Arthritis (JIA).

**Methods:** A 6 year old girl was referred to our Rheumatology Center for a chronic painful swelling of the right ankle. No history of trauma was reported, remote anamnesis was silent. Ankle swelling had appeared 2 years before but in the last months the patient had mild knee swelling as well.

At physical examination there were no signs of arthritis except for an ankle perimalleolar swelling, nodular subcutaneous lesions were palpable at right foot medial side, skin was normal. Knee and ankle range of motion were normal.

Laboratory exams were all within the normal range, without signs of phlogosis or thrombocytopenia. Ultrasound revealed the presence of three hypoechoic and vascularized nodules, identified as synovial cysts or ganglia. MRI showed a T1 hypointense, T2 hyperintense tissue with multiple confluent nodules and strong enhancement. The tissue was perischeletric and subfascial and stretched from the ankle to the peri-meniscal tissue of the knee.

**Results:** A biopsy was performed and KHE was diagnosed.

**Conclusion:** Differential diagnosis in JIA is often difficult as heterogeneous lesions may present with peritendineal or joint swelling.

Even if soft tissue tumors are rare in children, their presence should always be excluded when physical examination and imaging are atypical for JIA.

In this case the presence of palpable subcutaneaus noduli hypoechoic and vascularized at ultrasound could be firstly considered as “synovial cysts”: MRI revealed no signs of concomitant articular synovisits, so this hypothesis was rejected.

In case of atypical arthritis, MRI should always be performed for his help in diagnosing other condition mimicking JIA such as atypical KHE or other neoplastic lesions.

When tumor is suspected biopsy remains the diagnostic gold standard.

**Disclosure of Interest**

None Declared.

### P332 Hypocomplementemia in children with juvenile idiopatic arthritis treated with tocilizumab: personal records

#### Maria Cristina Maggio, Clotilde Alizzi, Bruno Papia, Beatrice Vergara, Umberto Corpora, Luca Messina, Giovanni Corsello

##### University Department Pro.Sa.M.I. “G. D’Alessandro”, University of Palermo, Palermo, Italy

###### **Presenting author:** Maria Cristina Maggio

**Introduction:** The relieve of a reduction in complement levels was recently reported in adults with Rheumatoid Arthritis treated with tocilizumab (TCZ). However, there are no data in children with Juvenile Idiopathic Arthritis (JIA) treated with TCZ.

**Objectives:** We evaluated complement levels in JIA children treated with TCZ for a systemic or a polyarticular form, and correlated them to clinical, biochemical parameters and to the response to the treatment.

**Methods:** 11 patients with active JIA (5 with polyarticular JIA; 6 with systemic JIA; 4 M/7 F); mean age 14 ± 7SDS) were treated with TCZ (7 after anti-TNF failure and 4 naives). All the patients were followed for the evaluation of clinical therapy response and of laboratory parameters. Laboratory parameters (leucocytes and thrombocytes count, liver enzymes, C3 and C4 levels) episodes of infections, allergic reactions, and autoimmune diseases were evaluated. 7/11 received DMARDs associated with TCZ.

**Results:** All the patients had normal complement levels pre-TCZ treatment: C3:90-180 mg/dl; C4: 10-40 mg/dl. 7/11 patients showed an early (within 3 months of treatment) hypocomplementemia. The reduction of C3 and C4 were coupled, even if in 3/7 children C3 was less reduced than C4. The complement depletion did not increase at the 6th month, while persisted during the ongoing therapy.

1/11 patients did not respond adequately to TCZ and 2/11 showed a flare of the disease during TCZ treatment. In these patients, C3 and C4 were in the normal range with a significant direct correlation with CRP, ESR, SAA. In 3 patients the treatment was stopped (2 for adverse reactions, 1 for the poor response to the treatment) with normalization of C3/C4 factors in the follow-up. In all patients no signs for the development of infection were observed; one patient showed leukopenia.

**Conclusion:** In our patients C3 and C4 decrease has a significant correlation with clinical response, while we did not observe hypocomplementemia in children and in the periods of lack in response to TCZ. The levels of C3 and C4 were more related to the efficacy than PCR or ESR values, which in 1 patient decreased in presence of clear clinical worsening. It can be hypothesized that complement factors are consumed during the clearance of immune complexes (anti-IL6Ab-IL6); however the reduction in complement levels is believed to be linked to the inhibition by TCZ of IL6 stimulation of hepatocyte acute phase protein synthesis and of complement upregulation. The number of children studied is too small to reach conclusions, but the authors hypothesize that the dosage of the C3 and C4 can be a rapid and sensitive marker of the response to anti-IL6 treatment, could be included in the follow-up of these patients and must be correlate to clinical outcome.

**Disclosure of Interest**

None Declared.

### P333 DMARD withdrawal in juvenile idiopathic arthritis patients in clinical remission. A single center observational study

#### Maria Tsinti, Vasiliko Dermentzoglou, Panagiotis Tziavas, Elena Tsitsami

##### Pediatric Rheumatology Unit, 1st Department of Pediatrics, PEDIATRIC HOSPITAL PAIDON AGHIA SOFIA, University of Athens, Medical School, Athens, Greece

###### **Presenting author:** Maria Tsinti

**Introduction:** Treatment with biologic and non-biologic DMARDs leads into clinical remission most juvenile idiopathic arthritis (JIA) patients. The potential of adverse effects from prolonged treatment must be balanced with the risk of disease flare after withdrawal of DMARDs.

**Objectives:** To estimate the likelihood of maintaining clinical remission (CR) after discontinuation of treatment with DMARDs in patients with JIA.

**Methods:** A retrospective chart review was conducted in a cohort of 272 patients with non-systemic JIA treated with MTX +/– anti-TNFα. In 29 patients; age at last follow up 8,7 (3,6-16,5) years, (24 girls; 22 oligo-, 6 polyarthritis, 1 ERA; 20/29 ANA + ve) with longstanding CR, defined according to Wallace criteria, treatment was withdrawn; initially by increasing dose intervals and by treatment discontinuation after 3 months. MTX was the first drug to withdraw. The median duration of patient observation was 63 (30-70) months. Disease activity was measured by JADAS. Data, expressed as median value and range, were analyzed by GraphPad prism 7, Mann Whitney non-parametric t-test for continuous variables.

**Results:** Nine out of 29 patients were treated with MTX and anti-TNFα (6 polyarthritis, 2 oligo + uveitis). All patients flared after MTX withdrawal. Combined treatment was re-initiated. Among the remaining patients, treated with MTX alone, 14/20 (70%) flared within 9,5 (1-25) months after treatment withdrawal. In 4/14, disease flare was more severe than that manifested before treatment cessation. The median duration of remission on treatment did not differ between patients with sustained remission; 24,5 (20–48) months; time to follow-up 15 (3-26)months;and those who flared; 28,5 (22-84) months (p = 0,25). Total duration of treatment with DMARDs prior to discontinuation was 35 (20-91) months for those who flared and 31,5 (20-55) months for those who remained into remission (p = 0,5). The median duration from the time of diagnosis of JIA to the initiation of MTX was similar between the 2 groups 13,3 (2,5-23) months in those who sustained remission 14,8 (1,2-86) in those who flared (p = 0,9). The category of JIA, sex, and age at diagnosis were not associated with the risk of relapse.

**Conclusion:** The majority of JIA patients relapsed after treatment discontinuation, despite longstanding remission on-medication. Withdrawal of both drugs was impossible; all patients relapsed after MTX discontinuation. Our data support the notion that clinical remission does not indicate biological inactivity. Parameters evaluated in everyday clinical care cannot predict the outcome of treatment withdrawal

**Disclosure of Interest**

None Declared.

### P334 Discontinuation of biologic therapy in jia patients in croatia, two centre- 8 year experience

#### Marija Perica^1^, Mandica Vidović^2^, Lovro Lamot^2^, Miroslav Harjaček^2^, Lana Tambić Bukovac^3^

##### ^1^Department of Pediatric and Adolescent Rheumatology, Children's Hospital Srebrnjak, Zagreb, Croatia; ^2^Department of Pediatric and Adolescent Rheumatology, Clinical Hospital Centre Sisters of Charity, Zagreb, Croatia; ^3^Department of Pediatric and Adolescent Rheumatology, Children’s Hospital Srebrnjak, Zagreb, Croatia

###### **Presenting author:** Marija Perica

**Introduction:** The introduction of biologic agents has revolutionized the treatment of juvenile idiopathic arthritis (JIA) due to their efficacy, speed of onset and tolerability. A numerous clinical practice guidelines and consensus statements on the criteria for biologic therapy (BT) introduction have been developed, however, the consensus on cessation of biologic agents has not been harmonized.

**Objectives:** Presentation of our experience on discontinuation of biologic therapy in JIA patients.

**Methods:** We conducted a retrospective two centre analysis of patients with JIA diagnosis according to ILAR criteria, treated with BT from January 2008 to May 2016. Demographic information, duration of the treatment, number of biologic agents used and discontinuation rate were extracted using medical charts. Successful discontinuation was defined as cessation of the drug due to disease control according to Wallace criteria and musculoskeletal ultrasound inactive disease.

**Results:** Total of 92 patients (87% female, 13% male) with different JIA subtypes, non-responders or intolerant to syntetic DMARDs, were treated with one or more biologicals. Median disease duration, from onset to the introduction of first BT, was 3.4 years (0.4-13 years). Patients were diagnosed with poli JIA in 68.5%, oligo JIA in 18.5%, ERA in 6.4%, systemic JIA in 3.3% and psoriatic JIA in 3.3%. In 88 patients first biologic drug was anti-TNF agent (etanercept in 54 pts, adalimumab in 18 pts, infliximab in 16 pts) and 3 pts were initially treated with tocilizumab and one patient with anakinra.

By the May 2016, 4 patients were lost from the follow up, 9 patients were transferred to adult rheumatology department while on BT, and 52 patient were still on BT. In 27 patients (29.3%) BT was successfully withdrawn, out of which 13 (14.1%) discontinued all medications. Majority of the patients were on the first line BT (20 pts; 74%), and another 7 patients were on the second line BT at the time of BT cessation. In another 37 patients BT discontinuation lasted for a short period of time (2-6 months), after which BT was reintroduced due to relapse. Median duration of BT before successful discontinuation was 2.96 years (1-7.2 years). The probability of the successful withdrawal of BT was associated with a shorter disease duration at the beginning of the treatment.

**Conclusion:** The development of optimal timeline and modality of discontinuation of BT after documentation of inactive disease is needed. In our retrospective study probability of successful withdrawal of BT was associated with shorter disease duration at the beginning of the treatment. These data correlate with current literature overview. Future studies will hopefully identify other predictors of the successful discontinuation of biologic therapy.

**Disclosure of Interest**

None Declared.

### P335 Clinical analysis of 265 juvenile idiopathic arthritis cases

#### Mustafa Çakan, Nuray Aktay Ayaz, Gonca Keskindemirci

##### Clinic of Pediatric Rheumatology, Kanuni Sultan Suleyman Research and Training Hospital, İstanbul, Turkey

###### **Presenting author:** Nuray Aktay Ayaz

**Introduction:** Juvenile idiopathic arthritis (JIA) is the most common cause of chronic arthritis in children.

**Objectives:** This study was conducted to analyse the clinical features and remission rates of JIA patients that are being followed-up at our pediatric rheumatology unit.

**Methods:** The files of 378 patients that were diagnosed as JIA between May 2010 and February 2016 were retrospectively analysed. The cases that were diagnosed during the last 6 months and that were dropped off regular follow-up (total 113 cases) were discarded from the study and finally 265 cases were analyzed. ILAR classification criteria were used for subgroup analysis.

**Results:** There were 87 enthesitis related arthritis (ERA), 87 oligoarticular arthritis (81 persistent, 6 extended), 36 RF(-) polyarticular arthritis, 35 systemic arthritis(sJIA), 10 RF (+) polyarticular arthritis, 5 psoriatic arthritis (PsA), 5 undifferentiated arhtritis (UA). Mean follow-up duration was 24.9 ± 15.5 months.

Male and female ratios were very close to each other in the total cohort (male: 136, female: 129). While ERA and UA groups had a significant male predominance, all other groups had a female predominance. Thirteen patients had diagnosis of FMF and 12 patients had a history of psoriasis in the first degree relatives. Mean age at diagnosis was 9.9 ± 4.9 years. The mean time elapsed from the first complaint to the diagnosis was 6.7 ± 6.7 months. sJIA patients were the earliest diagnosed group (1.2 ± 1.5 months) while RF (+) polyJIA patients had the longest duration of symptoms until diagnosis (11.5 ± 6.5 months). HLA-B27 positivity was found in 45% of ERA patients. Uveitis was found in 12 patients (4.5%). The majority of the uveitis cases had persistent oligoJIA (5 cases) and ERA (4 cases). ANA positivity was observed in 27.2% of all cases and 5 of the uveitis cases were ANA (+) and 7 of them were ANA (-).

Initial treatment consisted of intraarticular injection in 60 cases, methotrexate po in 120 cases, methotrexate sc in 90 cases and sulfasalazine in 41 cases. Drug side effect was observed in 27 patients (%10). The most common ones were nausea and vomiting in 8 cases with po methotrexate and 8 cases of toxic hepatitis with sc methotrexate. Biological agent was used in 69 cases (%26). RF (+) polyJIA (60%), PsA (60%) and RF(-) polyJIA (47,2%) were the groups that most commonly needed a biological agent. Persistent oligoJIA (9,8%) and sJIA (20%) patients were the groups that least needed a biological agent. Nine patients needed second biological agent and 3 patients were RF(-) polyJIA and 4 patients were sJIA cases that showed a chronic polyarticular course. Four cases needed third or fourth biological agent and 3 of them were sJIA cases that showed a chronic polyarticular course. When we look at remission rates, 183 cases (69%) were under remission while 82 cases (31%) were active during enrollment. sJIA (89%) and persistent oligoJIA (78%) cases were the groups that most commonly achieved remission, while RF(+) polyJIA (80%) and RF(-) polyJIA (50%) and extended oligoJIA (50%) patients were the most active groups.

**Conclusion:** JIA is a heterogeneous group of disorders that present with chronic arthritis. Patients with polyarticular onset and sJIA patients that follow a chronic polyarticular course may need a more aggressive treatment.

**Disclosure of Interest**

None Declared.

**Table 35 (abstract P335).** See text for description

JIA sub-group	Frequency	Sex (male/female)	Age at diagnosis (years)	Diagnosis delay (months)	Active /Remission
Oligo-JIA (extended)	6 (2,3%)	3/3	5,9	5	3/3
Oligo-JIA (persistent)	81 (30,6%)	27/54	7,6	5,3	18/63
RF (+) Poly-JIA	10 (3,8%)	4/6	12,1	11,5	8/2
RF (-) Poly-JIA	36 (13,5%)	13/23	9,5	9,6	18/18
Enthesitis related arthritis (ERA)	87 (32,8%)	68/19	13,2	8,7	27/60
Psoriatic arthritis	5 (1,9%)	2/3	6,6	5,2	2/3
Systemic JIA (sJIA)	35 (13,2%)	15/20	8,1	1,2	4/31
Undifferentiated	5 (1,9%)	4/1	10,8	8	2/3
Total	265	136/129	9.9	6,7	82/183

### P336 Giving children a voice through art: to understand and educate about the impact of juvenile idiopathic arthritis by creating art and telling digital stories

#### Paivi Miettunen^1^, Michael Lang^2^, Catherine Laing^2^, Susanne Benseler^1^, Tommy Gerschman^1^, Nadia Luca^1^, Heinrike Schmeling^1^, Anastasia Dropol^2^, Jaymi Taiani^3^, Nicole Johnson^1^, Brian Rusted^4^

##### ^1^Paediatrics, University of Calgary, Calgary, Canada; ^2^University of Calgary, Calgary, Canada; ^3^McCaig Institute, Calgary, Canada; ^4^Department of Art, University of Calgary, Calgary, Canada

###### **Presenting author:** Paivi Miettunen

**Introduction:** Although nearly 20,000 Canadian children are affected by juvenile idiopathic arthritis (JIA), little awareness for this condition exists in the community. JIA is often a life-long disease, and children suffer from “invisible challenges”- chronic pain and fatigue, limited participation in sports and extracurricular activities, daily medications, blood tests and other painful procedures. There is a knowledge gap about how children cope with such stresses. Art therapy decreases patients’ stress and storytelling results in psychosocial benefits for patients. This combined approach has not been previously studied in JIA.

**Objectives:** An innovative art/ visual story telling program for children with JIA consisted of the children creating an individual art work followed by “illness experience narrative” as a digital story. The specific aims were: 1) To understand if such an expressive opportunity improves children’s health status; 2) To publicize children’s art/stories to increase community education about JIA.

**Methods:** Participants: A prospective cohort of 10 children and adolescents (8 -18 years), affected by JIA (Alberta Children’s Hospital, Calgary, Canada) participated in one-day art workshop(s) that were not illness specific, but focused on visual art skills. Each child subsequently produced a digital story about their illness experience using art, talking, writing and videoclips, assisted by a research assistant. To assess health related quality of life (HRQL), Pediatric Quality of Life Inventory- Rheumatology Module (Peds QL-R) was used. The higher scores indicate better HRQL, with “0” indicating the worst and “100” the best HRQL. It was administered 1 week prior and 1 week post completion of the project. Individual structured interviews with the children were conducted following the creation of digital story. Social media/public venues were used to exhibit children’s expressive creations. This mixed methods study used philosophical hermeneutics for the qualitative methodology, which has been successfully used in healthcare situations where knowledge is expected to emerge from dialogue in a form of an unpredictable discovery rather than a controlled outcome.

**Results:** A total of 10 children and adolescents were included (4 males and 6 females). Each child created an individual art work (a sculpture, painting or collage) that was subsequently used as a point of discussion in the digital story narrative. The pre-project PedsQL-R mean (range) scores for the 5 subsections were as follows: 1) Pain and Hurt: 79 (19-100), 2) Daily Activities: 94 (55-100) 3) Treatment: 77(32-100), 4) Worry: 84.3 (33-100), 5) Communication: 60 (0-100). The scores were similar following the project. Post project interviews confirmed that creation of art/digital stories was viewed by all children as psychologically beneficial. Each patient consented to have their art work/story publicly shared (YouTube, TELUS SPARK, website, etc.) with the goal of improving community awareness and knowledge about JIA. An unexpected result was spontaneous written sharing by the parents of these children on the emotional benefits of creating both art and digital stories.

**Conclusion:** This project revealed presence of psychosocial stress and impaired functioning in JIA patients and confirmed the psychological benefits of creating art and of knowing “how to tell your story” from JIA patients’ perspective. The novel use of social/public media allowed for increased public education about JIA.

**Disclosure of Interest**

None Declared.

### P337 A long-term study of the course and outcome of juvenile idiopathic arthritis by applying the contemporary assessment tools

#### Panagiota Nalbanti, Maria Trachana, Polyxeni Pratsidou, Grigoris Pardalos, Vasiliki Tzimouli, Anna Taparkou, Maria Stavrakidou, Fotios Papachristou, Florence Kanakoudi-Tsakalidou

##### 1st Department of Pediatrics, Aristotle University, Thessaloniki, Hippokration Hospital, Thessaloniki, Greece

###### **Presenting author:** Panagiota Nalbanti

**Introduction:** Juvenile Idiopathic Arthritis (JIA) is a chronic disease, highly heterogeneous in clinical expression. Treatment is targeted at the taming of the disease activity and the optimal outcome. Due to the lack of specific criteria, the treatment efficacy is evaluated by the quantitative assessment of the disease status, in certain visit intervals.

**Objectives:** A long-term follow up study of JIA patients to depict the disease status in predefined time points, by using the widely accepted contemporary assessment tools.

**Methods:** 108 patients with JIA had been recorded in an electronic registry and followed up during the period 2001-2014. All met the study entry criteria. The assessment tools used were JADAS (Juvenile Arthritis Disease Activity Score), ACRpedi, Clinical Remission (CR) on/off medication (Wallace criteria), CHAQ (Childhood Health Assessment Questionnaire) and JADI (Juvenile Arthritis Damage Index–Articular [JADI-A]) /Extra-articular, [JADI-E], respectively).

**Results:** The mean patient follow-up duration was 8.25 years and the patient distribution regarding the disease course was as follows: persistent oligoarthritis 31.5%, extended oligoarthritis 27%, polyarthritis RF negative 25.9%, systemic JIA 7.4%, psoriatic 4.6%, enthesitis related arthritis 2.8% and polyarthritis RF positive 0.9%. The follow-up mean time was 98.9 ± 35.96 months. JADAS differed significantly in all patients between the first and the last visit (12.83 ± 11.83 *vs* 2.18 ± 4.29, p < 0.01). There was a positive association between the baseline JADAS and the early indication of biologics. The assessment of physical function by CHAQ, recorded a Disability Index > 0.50 in 37% of the patients at baseline but in only 6.5% of them at the last visit (p < 0.01). The development of JIA-associated uveitis was recorded in 21.3% of the patients, 30.36 ± 21.41 months after the disease onset. The overall percent of the disease activity period was 30.81 ± 17.98% and longer in patients with uveitis (p < 0.001). The mean duration of disease activity was significantly longer in patients with a polyarticular course, with extra-articular manifestations (uveitis) and in the receivers of biologics. The achievement of CR on medication was observed in 95.4% of the patients and lasted 25.12 ± 17.09 months, while CR off medication was evident in 37%, respectively. However, 45% of them relapsed. JADI-A/JADI-E was recorded in 4.6% and 7.4% of the patients, respectively.

**Conclusion:** The implementation of contemporary quantitative assessment tools contributes to a more objective evaluation of each patient’s disease status at the visits. These successive assessments lead to tailored treatment and optimal disease outcome.

**Disclosure of Interest**

None Declared.

### P338 Attitudes and applications of the myeloid related protein (MRP8/14) in the management of juvenile idiopathic arthritis: a single centre experience

#### Peter Bale^1^, Emily Robinson^1,2^, Jason Palman^2^, Clarissa Pilkington^1,2^, Elizabeth Ralph^3^, Kimberly Gilmour^3^, Clare Heard^1^, Lucy R. Wedderburn^1,2,4^

##### ^1^Paediatric Rheumatology, Great Ormond Street Hospital, London, UK; ^2^Institute of Child Health, London, UK; ^3^Immunology Department, Great Ormond Street Hospital, London, UK; ^4^Arthritis Research UK Centre for Adolescent Rheumatology, London, UK

###### **Presenting author:** Peter Bale

**Introduction:** Juvenile idiopathic arthritis (JIA) is the most common childhood inflammatory rheumatological condition. It is a heterogenous condition with variability in clinical presentation and response to treatment. One of the most challenging aspects of management is identifying true disease remission and when to stop medication. The Wallace Criteria are the current gold standard in clinical practice. Unfortunately, many patients meeting these criteria still have disease flare after stopping treatment. Myeloid related protein (MRP8/14), a calcium binding protein, member of the S100 family, has previously been shown to be a useful biomarker of subclinical disease activity with good correlation between concentration in synovial fluid and serum. It has also been proposed to predict which patients will respond to Methotrexate. The test remains a research test but is now available in a clinical setting in some centres.

**Objectives:** To assess the clinician’s attitude towards the use of measuring MRP8/14 in JIA patients and its practical application in one large Paediatric Rheumatology Centre.

**Methods:** An initial online questionnaire was conducted using the website Survey Monkey© inviting consultants, trainees and clinical nurse specialists in the Paediatric Rheumatology department to answer questions on the use of MRP testing. We then conducted a single centre audit assessing the use of the MRP test against evidence based recommendations following its availability in our Centre in March 2015. The audit assessed the reasons for requesting, medications relating to the request, time to result and the impact of the result in assisting clinical decision. All sample information was collected from screening electronic notes, investigation request forms and result reporting systems between January and March 2016.

**Results:** There was an 80% response rate on the survey. All responders were aware of the indications for requesting the test. Only 20% reported an intention to request before treatment commenced. All reported an intention to request MRP relating to Methotrexate withdrawal to assist risk stratification. Additionally, some would request MRP in relation to stopping other medications: Etanercept (50%), Adalimumab (42.9%), Tocilizumab (25.6%). If a patient was in clinical remission with a favourable MRP result (<4000 ng/mL, in locally performed assay) clinicians would stop Methotrexate (47%), Biologic (40%) or both (13%). The MRP test was requested 60 times, in 54 patients with available results in 98.3%. 88.3% of tests were requested according to evidence based recommendations. 90% of requests were related to Methotrexate. Result availability prior to next clinic appointment was 87.7%. The test assisted clinical decision making in 54.2% of cases (n = 24), these included: Methotrexate not being stopped due to high risk result (69.2%), Methotrexate withdrawal due to a low risk result (7.7%) and initiation of Methotrexate in likely responders (15.4%). Of the patients who had a clinical flare off Methotrexate, all had a high risk result (>6000 ng/ml) prior to withdrawal.

**Conclusion:** The MRP8/14 serum test was felt to be useful to support clinical decision making. The test was used appropriately according to recommendations in the majority of requests. All physicians surveyed stated their intention to use the investigation, all had good knowledge of its indications and had explained the research aspects of the test. This report supports the effective use of the serum MRP8/14 test in the management of children with JIA.

**Disclosure of Interest**

None Declared.

### P339 Temporomandibular joint involvement in a western switzerland cohort of JIA patients

#### Raffaella Carlomagno^1^, Yara Barrense-Dias^2^, Antonarakis Gregory^3^, Dhouib Amira^4^, Scolozzi Paolo^3^, Hanquinet Sylviane^4^, Hofer Michaël^1^

##### ^1^Pediatric rheumatology of Western Switzerland, CHUV and HUG, Lausanne and Geneva, Switzerland; ^2^Pediatric rheumatology of Western Switzerland, CHUV, Lausanne, Switzerland; ^3^Maxillo-facial surgery; ^4^Pediatric radiology, HUG, Geneva, Switzerland

###### **Presenting author:** Raffaella Carlomagno

**Introduction:** Temporomandibular joint (TMJ) involvement in JIA patients has been particularly studied during the last decades, both for its mild clinical presentation and its long-term consequences on mandibular growth. TMJ arthritis frequency varies widely in the literature, depending on the chosen diagnostic method, although the gold standard is enhanced MRI (1,2). A multidisciplinary TMJ consultation has been developed in Western Switzerland since 2010, where JIA patients with suspected TMJ arthritis are evaluated by a team including a pediatric rheumatologist, pediatric radiologist, orthodontist, and maxillofacial surgeon, by means of a clinical and MRI examination. Depending on clinical and radiological findings, treatment by intra-articular corticosteroids or arthroscopic lavage with physiological serum is proposed.

**Objectives:** The aim of this study was to describe our cohort of patients participating in the multidisciplinary TMJ consultation.

**Methods:** JIA patients presenting with TMJ pain, dysfunction, mandibular asymmetry, reduced mouth opening or prior to starting orthodontic treatment were addressed to the TMJ multidisciplinary consultation. Patient characteristics were analyzed using the JIR-Cohort database.

**Results:** 58 JIA patients were included in the study, 47 (81%) females and 11 (19%) males. Their mean age at JIA diagnosis was 8.7 years, whilst mean age at first TMJ consultation was 12.5 years. Pain was the chief concern justifying a TMJ consultation in the majority of cases (45/58, 78%), followed by mandibular asymmetry (6/58, 10%), dysfunction (3/58, 5%), or other complaints such as headache/otalgia (2/58, 3%) and prior to starting orthodontic treatment (2/58, 3%). Distribution of JIA subtypes was: persistent oligoarticular 13/58 (22%), extended oligoarticular 10/58 (17%), polyarticular FR+ 4/58 (7%), polyarticular FR- 10/58 (17%), enthesitic 17/58 (29%), systemic 2/58 (3%), undifferentiated 2/58 (3%). 31 patients had ANA+ (53%), and 8 had a history of uveitis (13%). Systemic treatment used were NSAIDs only in 17 patients (29%), 7 (12%) DMARDs, 19 (33%) biologic agents (BA) and 15 (26%) >1 BA. MRI results were classified into 4 groups depending on inflammatory findings (I) and articular damage (D), as follows: A (I+, D+), B (I+, D-), C (I-, D+), D (I-, D-). 20 patients were included in group A (34%), 5 in groups B and C respectively (9% each) and 28 in group D (48%). TMJ involvement (I+ and/or D+) was reported in 30/58 patients (52%), from which 25/58 (43%) had active inflammatory arthritis (group A and B). Treatment in these 2 groups were intra-articular physiological serum (10/25, 40%) and intra-articular corticosteroids (9/25, 36%). No TMJ treatment was administrated in 38/58 patients (65%).

**Conclusion:** The majority of patients were referred to our multidisciplinary consultation because of pain, and a TMJ active arthritis was found in a large number of them. Moreover, more than half of the patients referred to the TMJ consultation had consistent treatment with at least one BA. No correlation was found between TMJ involvement and a specific JIA subtype, nor with history of uveitis; on the contrary, a significant association with ANA+ was described in our cohort. TMJ involvement should be screened even in case of minor symptoms or signs in JIA patients, particularly those with severe disease; when TMJ arthritis is suspected, MRI should be performed rapidly.

**Disclosure of Interest**

None Declared.

### P340 The methylene tetrahydrofolate reductase C677T polymorphism in children with juvenile idiopathic arthritis

#### Nataliya Panko, Salah Shokry, Liudmila Rakovska

##### V.N. Karazin Kharkiv National University, Kharkiv, Ukraine

###### **Presenting author:** Salah Shokry

**Introduction:** The methylene tetrahydrofolate reductase C677T (MTHFR C677T) encodes the rate limiting enzyme in the methyl cycle and therefore, it has an important role in the biochemical reactions associated with the transfer of a methyl group such as metabolism of methotrexate.

**Objectives:** Goal of our research was to study the MTHFR C677T gene polymorphism to determine its peculiarities of clinical presentation of Juvenile Idiopathic Arthritis (JIA).

**Methods:** The study included 9 patients with JIA within 2 – 18 years old with methotrexate therapy more than 6 months. For determination of genotype MTHFR C677T gene we used PCR, for statistic processing of the material Stagraphics 3.0 and Student-Fischer test were used. Patients were divided into 2 groups according to their genotype of MTHFR 677 gene: 4 patients had genotype with CC-alleles, 5 patients – CT-alleles. Among studied patients TT-genotype was not determined**.**

**Results:** Patients with heterozygotic (CT) polymorphism of MTHFR C677T gene had relatively longer duration of the morning stiffness in comparison with those who were presented with CC genotype (p < 0.05). As a concomitant diseases Autoimmune Thyroiditis in patients with CT polymorphism was noticed more frequent than in children from other group (p < 0.05). In Patients with CT-alleles of MTHFR C677T gene joints lesion was characterized by symmetrical affection of hip joints (60%, p < 0.001), metacarpophalangeal joints (40%, p < 0.05) and asymmetrical involvement of wrist joints (40%, p < 0.05). 60% of persons with heterozygotic polymorphism of MTHFR C677T gene were presented with affection of temporo-mandibular joints (p < 0.001). Joints syndrome included deformity along or with limitation of movement in affected joints (p < 0.05) in children with CT genotype. More frequent III functional stage of affected joints in patient with CT-alleles was diagnosed (p < 0.05). 75% of patients with CC-alleles had a normal Ultrasound imaging of the affected joints (p < 0.001) while patients from the other group had changes. All of our patients with CT gene polymorphism had negative rate of anti-cyclic citrullinated peptide antibodies (p < 0.001) and they were associated with neutral homozygotic polymorphism of MTHFR A1298C gene.

**Conclusion:** Patients with MTHFR C677T gene polymorphism had worst and more severe progression of JIA, which leaded to insufficiency of affected joints. Those patients had negative A-CCP result, unlike patients from the MTHFR C677C group.

**Disclosure of Interest**

None Declared.

### P341 Clinical and demographic characteristics of cohort of children with juvenile idiopathic arthritis in 6 health care institutions in Bogotá, Colombia

#### Sally Pino^1,2,3^, Adriana Diaz-Maldonado^2,4,5^, Pilar Guarnizo^2,6,7^

##### ^1^Reumatología Pediátrica, Hospital Universitario Infantil de San José, Bogotá, Colombia; ^2^Reumatología Pediátrica, Care For Kids. Cuidado y Atención Reumatológica para niños, Bogotá, Colombia; ^3^Reumatología Pediátrica, Centro Integral de Reumatología. CIREI, Bogotá, Colombia; ^4^Reumatología Pediátrica, Hospital de la Misericordia, Bogotá, Colombia; ^5^Reumatología Pediátrica, Instituto de Ortopedia Infantil Roosvelt, Bogotá, Colombia; ^6^Reumatología Pediátrica, Fundación Cardio Infantil, Bogotá, Colombia; ^7^Reumatología Pediátrica, Riesgo de Fractura S.A. CAYRE, Bogotá, Colombia

###### **Presenting author:** Sally Pino

**Introduction:** Juvenile idiopathic arthritis (JIA) is the fifth most frequent chronical disease of the infancy. The incidence of JIA ranges from around 20 to 50 cases per 100.000 person-years and its prevalence ranging from 16 to 110 cases per 100.000 (1). In Latin America, there are different variations of the data from case series according to the region. Therefore, it is important to know the behavior of JIA in Colombia.

**Objectives:** To describe clinical and demographic characteristics of cohort of children with juvenile idiopathic arthritis (JIA) in 6 health care institutions in Bogotá, Colombia.

**Methods:** This retrospective study is composed by patients diagnosed with JIA who have been attended in 6 health care institutions in Bogotá, Colombia since 2010. The demographic and clinical characteristics were collected from chart reviews. They included age, gender, origin, subtype of JIA, age of onset, rheumatoid factor (RF), antinuclear antibodies (ANA), human leukocyte antigen B27 (HLA-B27), remission, frequency of uveitis and current treatment. Dichotomous and nominal variables were described in percentage and continuous variables using median and standard deviation.

**Results:** Five hundred sixty eight patients were studied. The 57.22% were female patients with a mean age of 13.59 ± 1,22 years. The 65.4% of patients were located in Bogotá. The most frequent JIA subtype was polyarticular rheumatoid factor (RF)-negative JIA (24%), followed by oligoarticular (23.06%), enthesitis related arthritis (ERA) (19.19%), polyarticular rheumatoid factor (RF) –positive(14%) and systemic arthritis (10.74%) and Psoriatic arthritis 2.46%.

Positive HLA-B27 was observed in 89,6% of ERA patients, and positive ANA was 37.1% of the oligoarticular JIA patients. Male patients were the most frequent in ERA (78,9%) while female patients were in polyarticular subtype, psoriatic arthritis and undifferentiated arthritis. For systemic arthritis and oligoarticular JIA the gender was proportional.

Regarding to age of onset, the oligoarticular subtype was diagnosed at earliest years (mean age of 6.63 years); systemic and psoriatic arthritis presented at mean age of 8.31 years and 9.17 years, respectively; undifferentiated arthritis and polyarticular JIA the mean age were 10.37 and 10. 86 years.

Only, the 3.8% of the patients presented uveitis (8 cases in oligoarticular JIA where 3 cases were positive ANA, 5 cases in ERA, 4 cases in polyarticular and 3 cases in psoriatic arthritis which correspond to 25% of this kind of arthritis).

**Conclusion:** In the cohort of children with JIA that were attended in Bogota, the mayor prevalence was in female gender and the polyarticular with negative RF.

In ERA, male gender was more frequent with a high positive level of HLA-B27. Uveitis presented low frequency in the analyzed population being oligoarticular JIA the most frequent without relation to ANA.

**Disclosure of Interest**

None Declared.

### P342 Surface electromyography in the evaluation of temporomandibular involvement in juvenile idiopathic arthritis

#### Sofia Torreggiani^1^, Paolo Cressoni^2,3^, Umberto Garagiola^2,3^, Giancarla Di Landro^1^, Giampietro Farronato^2,3^, Fabrizia Corona^1^, Giovanni Filocamo^1^

##### ^1^Reumatologia Pediatrica, IRCCS Fondazione Ca' Granda Ospedale Maggiore Policlinico, Milano, Italy; ^2^Maxillo-Facial and Odontostomatologic Unit, IRCCS Fondazione Ca' Granda Ospedale Maggiore Policlinico, Milano, Italy; ^3^Department of Biomedical, Surgical and Dental Sciences, University of Milan, Milano, Italy

###### **Presenting author:** Sofia Torreggiani

**Introduction:** Juvenile idiopathic arthritis (JIA) frequently affects also the temporomandibular joints (TMJ), potentially leading to condylar lesions. Condylar erosion can progress insidiously and alter the masticatory function and, in some cases, also the craniofacial profile. Even though bone alterations are best detected by Computed Tomography (CT), such investigation should be prescribed carefully, since it exposes the patient to ionizing radiation. In the last years, surface electromyography (SEMG) has been increasingly used to evaluate temporomandibular disorders, but its diagnostic accuracy is still questioned.

**Objectives:** Our aim is to assess whether, in patients affected by JIA, SEMG findings of masticatory muscles correlate with condylar erosion as documented by cone beam CT scans of the TMJ.

**Methods:** We retrospectively reviewed the medical records of JIA patients who were addressed to an orthodontist due to the presence of signs and symptoms of temporomandibular involvement. Patients who performed both a cone beam CT scan of the TMJ and a SEMG of masseter and temporal muscles within a 6 month period were included in the study. Severity of TMJ condylar erosion was classified from grade 0 (absence of erosion) to grade 4 (extensive erosion). Each of the masseter and temporal muscles was classified as normotonic, hypotonic or hypertonic.

Cone beam CT scan was considered pathologic if the grade of erosion was > 0. SEMG was considered pathologic if at least three of the examined muscles showed altered tone. Using CT as the gold standard to detect condylar lesions, we calculated sensitivity and specificity of SEMG examination.

**Results:** Eighteen patients were included in the study, 13 females and 5 males. Mean age at onset of JIA was 5,9 ± 3,8 years. The most represented JIA category was polyarticular JIA (39%). Mean disease duration when CT scan was performed was 9,1 ± 6,1 years; mean disease duration when SEMG was performed was 9,6 ± 6,3 years. Cone beam CT scan of TMJ showed different grades of bone erosion in 14 patients out of 18. SEMG was completely normal only in one patient, while the other patients presented hypertonus or hypotonus in at least one of the masticatory muscles. Results are summarized in Table 36. Neither increased nor decreased muscle tone in any of the masticatory muscles examined showed a clear association with the severity of bone erosion of ipsilateral or contralater condyle. Considering cone beam CT scan as the gold standard, SEMG showed low sensitivity (57%) and specificity (25%).

**Conclusion:** In our study, SEMG findings of hypertonus or hypotonus did not show a clear correlation with the severity of condylar erosion as seen in CT scans. SEMG had poor sensitivity and specificity in detecting condylar damage. Nevertheless, it should be considered that our population is small and CT and SEMG were performed late in the disease course: further observations may better show the utility of SEMG for diagnosing and managing TMJ disease in JIA.

**Disclosure of Interest**

None Declared.

**Table 36 (abstract P342).** Results

	Muscle tone (number of patients)	Right condyle erosion (median)	Left condyle erosion (median)		Muscle tone (number of patients)	Right condyle erosion (median)	Left condyle erosion (median)
**Right temporal muscle**	Normal (5)	1	1	Right masseter	Normal (5)	3	1
	Hypo (7)	1	1		Hypo (6)	0,5	1,5
	Hyper (6)	2,5	1		Hyper (7)	1	1
**Left temporal muscle**	Normal (7)	3	1	Left masseter	Normal (7)	1	0
	Hypo (6)	0,5	0,5		Hypo (5)	1	2
	Hyper (5)	1	1		Hyper (6)	2	1

### P343 Juvenile idiopathic arthritis in relation to perinatal and maternal characteristics

#### Susan Shenoi^1^, Samantha Bell^2^, Parveen Bhatti^3^, Lee Nelson^4^, Beth A. Mueller^5^

##### ^1^Department of Pediatric Rheumatology, Seattle Children's Hospital, Seattle, WA, USA; ^2^Department of Epidemiology, University of Washington, School of Public Health, Seattle, WA, USA; ^3^Department of Epidemiology, University of Washington, School of Public Health, Seattle WA, USA; ^4^Public Health Sciences Division, Fred Hutchison Cancer Research Center, Seattle, WA, USA; ^5^Department of Epidemiology, University of Washington, School of Public Health, Seattle, WA, USA

###### **Presenting author:** Susan Shenoi

**Introduction:** Juvenile Idiopathic Arthritis (JIA) is a heterogeneous group of chronic inflammatory arthritis occurring in children with onset before 16 years of age, and is the leading cause of acquired short and long-term disability in childhood. The etiology of JIA is largely unknown, however there is increasing evidence that autoimmune diseases, including JIA, may be associated with maternal reproductive or early childhood exposures.

**Objectives:** We explored juvenile idiopathic arthritis (JIA), and JIA categories in relation to selected infant and maternal characteristics.

**Methods:** This case-control study used ICD-9 billing codes from hospital records to identify 1518 JIA cases born in Washington (WA) State and diagnosed at a quaternary pediatric center from 1997-2010. Controls (n = 6072) were randomly selected from birth records of children without JIA, frequency matched on birth year. Review of medical records further refined case ascertainment based on International League of Associations of Rheumatologist (ILAR)criteria (N = 1,234) and allowed categorization of cases into JIA ILAR categories. Multivariable logistic regression was used to estimate adjusted odds ratios (OR) and 95% confidence intervals (CI) for the associations of JIA and its categories with maternal and early life exposures as measured in the birth certificates.

**Results:** ORs were decreased for JIA in relation to high birthweight (OR 0.81, 95%CI 0.67-0.98 for 4000+ grams), and greater maternal parity was associated with decreased odds ratio for JIA (most marked for persistent oligoarticular JIA, OR 0.32, 95%CI 0.15; 0.71, p for trend = 0.0001). Prior fetal loss (except for oligoarticular JIA) was associated with increased OR for JIA categories.

Strengths of the study include: 1) population based case-control design with linkage of cases identified from outpatient & inpatient medical records maintained by pediatric rheumatologists to the Washington State birth registry database to allow identification of a large number of cases. 2) Exposure information recorded prior to disease onset hence not subject to recall bias. 3) Retrospective chart review identified cases that would have been misclassified if only ICD-9 codes were used

Limitations of the study include: 1) Small numbers in some categories could mask certain category specific associations. 2) Potential inaccuracies in maternal reproductive characteristics. 3) Referral bias 4) Potential for misclassification (may have biased results towards null) as some cases may have moved from WA State, been diagnosed elsewhere, and older cases may have received care from adult rheumatologists.

**Conclusion:** We observed associations of selected maternal factors with JIA, some of which varied across JIA categories. The maternal parity observations could be consistent with the hygiene and microchimerism hypotheses.

**Trial registration identifying number:** NA

**Disclosure of Interest**

None Declared.

### P344 Characteristics of patients with juvenile idiopathic arthritis in a us healthcare claims database

#### T. A. Simon^1^, A. Baheti^2^, N. Ray^2^, Z. Guo^1^

##### ^1^Bristol-Myers Squibb, Princeton, USA; ^2^Mu Sigma, Bangalore, India

###### **Presenting author:** T. A. Simon

**Introduction:** Abatacept, the first selective co-stimulation modulator approved and used for the treatment of juvenile idiopathic arthritis (JIA), has a mechanism of action that is fundamentally different from that of other biologic disease modifiers (BDM).

**Objectives:** The purpose of this study is to describe the baseline characteristics of patients with a diagnosis of JIA in a US healthcare claims (HCC) database treated with abatacept and those treated with other BDMs.

**Methods:** Patients <18 years of age and diagnosed with JIA in the Truven Health MarketScan® database between 1 July 2006 and 30 September 2014 were eligible for inclusion in the analysis. Patients were required to have at least 180 days of continuous health plan enrolment prior to, and ≥1 day following, a diagnosis of JIA based on two International Classification of Diseases, Ninth Revision, Clinical Modification codes (714.3x) within 90 days.

The abatacept cohort includes patients initiating abatacept or another BDM who may be initiating a biologic for the first time or switching from one biologic to another. Baseline characteristics including at least 15 co-morbid conditions and concomitant medications were captured within the 6-month period prior to the diagnosis of JIA.

**Results:** 13,602 patients with a diagnosis of JIA were identified in the US HCC database, with an average follow-up of 2.26 years (maximum 8.26 years); 343 abatacept and 3507 other BDM users were identified. Overall, abatacept users were slightly older and more likely to have asthma and a cardiovascular disease; other BDM users were more likely to have uveitis reported in the 6-month baseline period. Abatacept users also had a slightly higher mean number of outpatient visits. The table is limited to characteristics that had a minimum of 2% difference between the groups.

**Conclusion:** Overall, patients with JIA who are treated with abatacept are similar to patients treated with other BDMs, with the exception of slight differences observed in a few co-morbid conditions. Patients with JIA treated with abatacept in the US HCC database are slightly younger, with less uveitis at baseline, compared with a population of abatacept-treated patients in a JIA registry (mean age 13 years and 15% history of uveitis).^1^

Healthcare claims data are another source of data to monitor the use of medications in the real world.

**Reference**

1. Lovell DJ, et al. *ACR/ARHP Annual Scientific Meeting*, 2015. Poster 1446

**Disclosure of Interest**

T. Simon Shareholder of: Bristol-Myers Squibb, Employee of: Bristol-Myers Squibb, A. Baheti : None Declared, N. Ray: None Declared, Z. Guo Shareholder of: Bristol-Myers Squibb, Employee of: Bristol-Myers Squibb

**Table 37 (abstract P344).** See text for description

	JIA patients N = 13,602 n (%)	Orencia-treated patients n = 343 n (%)	Other biologic-treated patients n = 3507 n (%)
Female, n (%)	9679 (71.2)	284 (82.8)	2563 (73.1)
Age, mean (SD)	10.4 (4.6)	12.0 (3.8)	11.2 (4.4)
Asthma, n (%)	873 (6.4)	28 (8.2)	187 (5.3)
Cardiovascular disease, n (%)	618 (4.5)	24 (7.0)	171 (4.9)
Uveitis, n (%)	1302 (9.6)	32 (9.3)	467 (13.3)
Biologic DMARDs, n (%)	1732 (12.7)	168 (49.0)	1564 (44.6)
Non-biologic DMARDs, n (%)	3321 (24.4)	156 (45.5)	1548 (44.1)
Inpatient visits, mean (SD)	0.08 (0.36)	0.10 (0.45)	0.09 (0.38)
Outpatient visits, mean (SD)	8.70 (7.8)	11.66 (10.4)	9.6 (8.5)

.

### P345 Pharmacokinetics, safety and tolerability of tofacitinib in paediatric patients from two to less than eighteen years of age with juvenile idiopathic arthritis

#### Nicolino Ruperto^1^, Hermine I. Brunner^2^, Anasuya Hazra^3^, Thomas Stock^3^, Ronnie Wang^4^, Charles Mebus^4^, Christine Alvey^4^, Manisha Lamba^4^, Sriram Krishnaswami^4^, Umberto Conte^5^, Min Wang^5^, Nikolay Tzaribachev^6^, Ivan Foeldvari^7^, Gerd Horneff^8^, Daniel Kingsbury^9^, Elena Koskova^10^, Elzbieta Smolewska^11^, Richard K. Vehe^12^, Zbigniew Zuber^13^, Alberto Martini^1^, Daniel Lovell^2^

##### ^1^Istituto G. Gaslini, Pediatria II, PRINTO, Genoa, Italy; ^2^Pediatric Rheumatology Collaborative Study Group, Cincinnati Children’s Hospital Medical Center, Cincinnati, USA; ^3^Pfizer Inc, Collegeville, USA; ^4^Pfizer Inc, Groton, USA; ^5^Pfizer Inc, New York, USA; ^6^Pediatric Rheumatology, Klinikum Bad Bramstedt, Bad Bramstedt, Germany; ^7^Hamburger Zentrum für Kinder- und Jugendrheumatologie, Hamburg, Germany; ^8^Asklepios Klinik, Sankt Augustin, Germany; ^9^Randall Children’s Hospital, Portland, USA; ^10^Institute of Rheumatic Diseases, Piestany, Slovakia; ^11^Department of Pediatric Cardiology and Rheumatology, Medical University of Lodz, Lodz, Poland; ^12^University of Minnesota Masonic Children’s Hospital, Minneapolis, USA; ^13^St Louis Children’s Hospital ODS Rheumatology and Neurology, Krakow, Poland

###### **Presenting author:** Thomas Stock

**Introduction:** Tofacitinib is an oral Janus kinase inhibitor for the treatment of rheumatoid arthritis (RA).

**Objectives:** To characterise the pharmacokinetics (PK), safety and taste acceptability of tofacitinib following multiple oral doses in patients (pts) 2– < 18 years (yrs) old with active juvenile idiopathic arthritis (JIA).

**Methods:** Data were obtained from an open-label, non-randomised, multicentre, Phase 1 study where JIA pts were given 5 mg adult equivalent (based on body weight) of tofacitinib (tablet or solution) twice daily (BID) for 5 days. There were 3 cohorts (COH) based on pt age: COH1, 12– < 18 yrs; COH2, 6– < 12 yrs; and COH3, 2– < 6 yrs, with a target enrolment per group of ≥8 JIA pts for N = ≥24 evaluable pts completing the study. Pts were enrolled in a stepwise approach beginning with the older age COH first. Subsequent younger-age COH were enrolled following confirmation of safety and PK from the previous COH. PK parameters of tofacitinib were calculated using noncompartmental analysis of plasma concentration (conc)-time data. Taste acceptability of the solution formulation was listed and categorically summarised (frequency and %).

**Results:** 26 pts (COH1 [N = 8], COH2 [N = 9] and COH3 [N = 9]) were included in this analysis. Pts’ age ranged from 2–17 years; all were white race except for one; there were 17 females and 9 males. Baseline disease characteristics were similar across all COH. All exposure metrics including geometric mean (GM) area under the conc-time curves within a dosing interval (AUC_tau_), maximum (C_max_), minimum (C_min_) and predose (C_trough_) conc were lower in COH2 relative to those in COH1; however, due to higher doses in COH3 (modified after interim analysis of COH1 and 2), the mean AUC_tau_ in COH3 was comparable to COH1. GM apparent volume of distribution (Vz/F) decreased with age (COH1 = 104.9 L, COH2 = 71.0 L, COH3 = 51.4 L). Average terminal half-life (t_½_) was COH1 = 2.62 h, COH2 = 1.95 h and COH3 = 1.77 h. GM tofacitinib CL/F was 53%, 39% and 12% higher in COH1, COH2 and COH3 pts, respectively, vs adult RA pts (18.4 L/h) receiving tofacitinib 5 mg BID. GM CL/F and V/F parameters were similar between males and females. No serious adverse events or new safety signals were identified.

**Conclusion:** Tofacitinib clearance decreased with body weight in children as expected. PK results from this study established body weight-based dosing regimens for pts aged 2–18 years to be used in the upcoming efficacy and safety studies of tofacitinib in JIA pts. Tofacitinib, administered over 5 days as multiple dose tablets or solution formulation, was well tolerated in this study in JIA pts. Overall, pts found the taste of the tofacitinib solution formulation to be acceptable. These data were presented previously.^1^

**Reference**

1. Ruperto N et al. European League Against Rheumatism, London, UK, 8-11 June 2016 (Abstract: AB0879)

**Acknowledgements:** This study was supported by Pfizer Inc. Editorial support was provided by C Cridland of Complete Medical Communications and funded by Pfizer Inc.

**Trial registration identifying number:** NCT01513902

**Disclosure of Interest**

N. Ruperto Consultant for: Pfizer Inc, Speaker Bureau of: Pfizer Inc, H. Brunner Consultant for: Pfizer Inc, A. Hazra Shareholder of: Pfizer Inc, Employee of: Pfizer Inc, T. Stock Shareholder of: Pfizer Inc, Employee of: Pfizer Inc, R. Wang Shareholder of: Pfizer Inc, Employee of: Pfizer Inc, C. Mebus Shareholder of: Pfizer Inc, Employee of: Pfizer Inc, C. Alvey Shareholder of: Pfizer Inc, Employee of: Pfizer Inc, M. Lamba Shareholder of: Pfizer Inc, Employee of: Pfizer Inc, S. Krishnaswami Shareholder of: Pfizer Inc, Employee of: Pfizer Inc, U. Conte Shareholder of: Pfizer Inc, Employee of: Pfizer Inc, M. Wang Shareholder of: Pfizer Inc, Employee of: Pfizer Inc, N. Tzaribachev Grant / Research Support from: Pfizer Inc, I. Foeldvari: None Declared, G. Horneff Grant / Research Support from: Pfizer Inc, D. Kingsbury Grant / Research Support from: Pfizer Inc, E. Koskova: None Declared, E. Smolewska: None Declared, R. Vehe Grant / Research Support from: Pfizer Inc, Z. Zuber: None Declared, A. Martini: None Declared, D. Lovell Consultant for: Pfizer Inc

### P346 Early prediction for sustained long-term efficacy of the 1st biologic agent in polyarticular juvenile idiopathic arthritis; a multi-center study in Japan

#### Tomohiro Kubota^1^, Masaki Shimizu^2^, Junko Yasumura^3^, Yasuo Nakagishi^4^, Toshitaka Kizawa^5^, Masato Yashiro^6^, Hiroyuki Wakiguchi^1^, Tsuyoshi Yamatou^1^, Yuichi Yamasaki^1^, Syuji Takei^1,7^, Yoshifumi Kawano^1^

##### ^1^Pediatrics, Kagoshima University Hospital, Kagoshima, Japan; ^2^Pediatrics, Kanazawa University, Kanazawa, Japan; ^3^Pediatrics, Graduate School of Biomedical and Health Sciences, hiroshima, Japan; ^4^Pediatrics, Hyogo Prefectural Kobe Children’s Hospital, Kobe, Japan; ^5^Pediatrics, Sapporo Medical University School of Medicine, Sapporo, Japan; ^6^Pediatrics, Okayama University Hospital, Okayama, Japan; ^7^School of Health Sciences, Faculty of Medicine, Kagoshima University, Kagoshima, Japan

###### **Presenting author:** Tomohiro Kubota

**Introduction:** It is very important for pJIA patients to control disease activity in the early phase of the disease and to switch biologic agents if the initial treatment is ineffective. However, in pJIA patients, there have been few studies designed to assess the target of treatment or predictive markers in which the initial treatments were continuously effective.

**Objectives:** To examine clinical parameters to predict sustained long-term efficacy of the 1st biologic agents by 3 months after initiating the therapy.

**Methods:** Polyarticular JIA (pJIA) patients with treated with biologic agents between March 2005 and October 2014 at five medical centers for pediatric rheumatology in Japan were involved in this study. Patients were divided into two groups; patients who maintained clinical remission with 1st biologic agent (non-switching group) and patients who eventually switched to other biologics during the disease course (switching group). Clinical findings and laboratory data were retrospectively obtained from medical records and compared in both patients’ groups.

**Results:** Thirty-two pJIA pantents, 8 boys and 24 girls, mean disease duration was 12 months at initiating the 1st biologic agents, were enrolled in this study. The 1st biologic agents used was adalimumab (ADA) in 9 (28%), etanercept (ETA) in 8 (25%), inflixmab (INF) in 2 (6%), and tocilizmab (TCZ) in 13 (41%). Twenty-four patients (75%) maintained clinical remission with the first biologic agent for 40 months in median, (range 24–119 months) (non-switching group), while 8 patients (25%) eventually switched to the 2nd biologic agent at 9.5 months in median (range 6–18 months) (switching group). Among the clinical data after starting the 1st biologic agent, DAS28-ESR score showed significant difference between the two groups at 3 months, 1.87 in non-switching group and 2.94 in switching group (p = 0.009), and its cut-off value was 2.49 (p = 0.0003).

**Conclusion:** Patients with pJIA who attained a DAS28-ESR <2.49 at three months after initiating the 1st biologic agent may sustain clinical remission for more than two years without switching to the 2nd biologic agent.

**Disclosure of Interest**

None Declared.

### P347 Children and adolescents referred to the pain clinic from the clinic of pediatric rheumatology with longstanding pain, a follow-up study

#### Ulrika Järpemo Nykvist^1^, Bo Magnusson^2^, Rikard Wicksell^3^, Karin Palmblad^4^, Gunnar L. Olsson^3^

##### ^1^Behavioral Medicine Pain Treatment Service, Karolinska Institute, Stockholm, Sweden; ^2^Ped rheum unit, Astrid Lindgren Children´s hospital, Karolinska University hospital, Stockholm, Sweden; ^3^Behavioral Medicine Pain Treatment Service, Department of Physiology and Pharmacology Karolinska Institute, Stockholm, Sweden; ^4^Ped rheum unit, Astrid Lindgren Children´s hospital, Department of Women´s and Children’s health, Karolinska Institute, Stockholm, Sweden

###### **Presenting author:** Ulrika Järpemo Nykvist

**Introduction:** Scientific follow-up of longstanding pain in children are scarce in the literature. The Pain Treatment service receives many referrals from the unit of rheumatology, Astrid Lindgren Children´s hospital.

**Objectives:** The aim of the study is to follow up pain intensity, function and quality of life of the referred patients.

**Methods:** Data was collected from the referral information and clinical records; blood samples, clinical examination and radiology. Data was also collected from Behavioural Medicine Pain Treatment Service, Karolinska University Hospital´s clinical records concerning what kind of treatment they received, short or long term, individually or in a group setting. Standard self-report pre- and post- treatment questionnaires were also retrieved. A letter was sent to the patients including questions about pain and validated instruments to measure the impact of pain on life (Disabkids, CHAQ).

**Results:** 79 patients, 7-16 years old, with a median age of 14, 14 boys and 65 girls were referred 2009-2014.

52 patients showed signs of arthritis in the clinical examination before referral to the pain clinic. 20 had signs of arthritis on radiology-examinations, 40 had no signs and in 19 cases information was missing. No one had a positive rheumatoid factor, 48 were tested negative and in 31 children information was missing. 11 had a positive ANA, 40 negative. 6 tested positive on the HLA-27-test and 35 negative. Out of the 53 who received treatment, 37 were judged by an experienced pediatric rheumatologist as having arthritis.

**Conclusion:** A spectrum of patients with JIA, possible JIA or no true inflammatory disorder at all are reffered for pain treatment from the pediatric rheumatology unit. The results of treatment varies depending on motivation and underlining disorder. It is imortant to define criteria for patients to be or not to be reffered to a pain clinic.

**Disclosure of Interest**

None Declared.

### P348 Pulmonary function test in children with juvenile idiopathic arthritis

#### Vahid Ziaee^1,2^, Mohammadreza Modaressi^1^, Mohammad-Hassan Moradinejad^3^

##### ^1^Pediatrics, Tehran University of Medical Sciences, Tehran, Islamic Republic of Iran; ^2^Pediatric Rheumatology Research Group, Rheumatology Research Center, Tehran, Islamic Republic of Iran; ^3^Pediatric Rheumatology, Children's Medical Center, Pediatric Center of Excellence, Tehran, Islamic Republic of Iran

###### **Presenting author:** Vahid Ziaee

**Introduction:** Juvenile Idiopathic Arthritis is the most common cause of chronic arthritis in children. Pulmonary involvement has been reported in rheumatoid arthritis with restrictive pattern, frequently. But there are a few reports on study of pulmonary function test (PFT) in children with JIA.

**Objectives:** The aim of this study was to evaluate pulmonary function parameters in children with JIA in comparison with normal children.

**Methods:** This cross sectional study was performed on 40 JIA patients recruited from the Rheumatology Clinic of the Children’s Medical Center Hospital, the Pediatrics Center of Excellence in Iran. Diagnosis of JIA was made according to the ILAR classification criteria for JIA. PFT was performed by using Spirolab II (RDSM Co., Italy). They did not have any history of a recent or chronic respiratory tract infection, or history of respiratory allergic diseases. Control group were sex-age match children without any chronic diseases and recent respiratory infection.

**Results:** Cases group were 17 male (42.5%) and 23 female (57.5%) with mean age 11.2 (±3.89) years (range 5-23 years). From 40 patients, 8 cases were new patients (less than 6 months from beginning of treatment) and 32 cases were old patients (more than 6 months). All of them didn’t have any respiratory or cardiac symptoms. 21 patients (52.5%) had normal PFT and 1 patient (2.5%) had obstructive pattern and 18 patients had restrictive pattern in PFT. From 18 patients with restrictive pattern, 9 cases (22.5%) had mild, 7 cases (17.5%) moderate and 2 patients (5%) had severe restrictive pattern in PFT. From 32 old cases, 15.6% were in remission, 28.1% were uncontrolled and 56.3% were controlled on medication. Restrictive pattern in PFT was less common in controlled group in comparison 2 other groups (P = 0.05).

All pulmonary parameters were significantly lower in case group in comparison to control group (P < 0.01) except FEV1 ratio (P = 0.3). (Table 38).

**Conclusion:** PFT should be considered in all new patients with JIA before started medication and after medication in regular interval, if treatment last more than 6 months.

**Disclosure of Interest**

None Declared.

**Table 38 (abstract P348).** Pulmonary function parameters in patients with JIA and control group

Parameter	group	Mean	Std. Deviation	Mean Difference	CI95%	P. value
FVC	Case	1.63	0.70	1.06	0.66 _ 1.47	<0.001
	Control	2.70	1.oo			
FEV1	Case	1.50	0.60	0.87	0.53_ 1.20	<0.001
	Control	2.37	0.81			
FEV1 ratio	Case	92.41	7.27	-2.17	-6.97_2.64	0.3
	Control	90.24	12.75			
PEF	Case	3.55	1.22	1.60	0.91_2.30	<0.001
	Control	5.16	1.73			

*FVC* Forced Vital Capacity, *FEV1* Forced Expiratory Volume in the 1st second, *FEV1%* FEV1/FVC ratio, *PEF* Peak Expiratory Flow, *CI95%* 95% Confidence Interval of the Difference

### P349 Growth dynamics of children with juvenile idiopathic arthritis during therapy with adalimumab and etanercept

#### Valentina Seraya, Elena Zholobova, Alisa Vitebskaya

##### I.M.SECHENOV FIRST MOSCOW STATE MEDICAL UNIVERSITY, Moscow, Russian Federation

###### **Presenting author:** Valentina Seraya

**Introduction:** Juvenile idiopathic arthritis (JIA) is a severe progressive disease affecting mainly the joints that develops in children under the age of 16. In juvenile arthritis often occur growth disorders. It is important to study the influence of biological therapy on growth.

**Objectives:** to assess the impact of adalimumab (ADA) and etanercept (ETA) in the growth rate in children with JIA.

**Methods:** The study included 50 children diagnosed with JIA between the ages of 6 to 17 years, all children with high clinical and laboratory disease activity. Of these, 14 children had systemic JIA, 15 children - oligoarticular, 11 of them with uveitis, and 21 patients had polyarticular JIA, of which 2 with uveitis. All patients before biological therapy obtained active antirheumatic therapies including glucocorticoids (GC): orally - 15 children (30%); in the form of pulse therapy 14 (28%); 10 patients (20%) was obtained in a massive long GC topical therapy (retrobulbar, subconjunctivally); 11 children (22%) received no GC therapy. The study evaluated growth SDS, growth rate and growth rate SDS prior of the therapy with ADA and ETA and after 6, 12, and after 24 month of therapy.

**Results:** The average age of debut was 4,1 ± 2,9 years (8 months to 11,4 years). Of the 25 children receiving ADA in 21 patients (84%) initially growth SDS were normal (growth SDS -0,61 ± 1,43), in 4 children (16%) there was a growth delay of up to -3,52 SDS. Initial average growth rate was 4,9 ± 2,3 cm / year, SDS growth rate of 0,01 ± 1,96. During treatment with adalimumab marked an increase in growth rate and in the growth rate SDS: from 2,14 ± 4.13 in the first 6 months (p = 0.047), to 2,04 ± 4,63 after 12 months and 1,40 ± 3,47 to 24 months of treatment. Growth SDS after 24 months therapy with adalimumab has increased significantly and amounted -0,39 ± 1,50.

Of the 25 children receiving etanercept, in 19 patient’s (76%) growth SDS initially was normal (the SDS growth -1.40 to 2.37). 6 children (24%) had moderate stunting (growth SDS -4.47 to -2.04). The initial growth rate was 3,3 ± 2,7 cm/yr, growth rate SDS -1,70 ± 2,78. In the first 6 months of therapy the growth rate increased to 6,5 ± 2,8 cm/yr, growth rate SDS increased to 0,65 ± 2,85 (p = 0.048). After 12 months, the growth rate was 6,7 ± 3,3sm / year, growth rate SDS 1,13 ± 3,97. After 24 months of treatment, the growth rate amounted to 5,4 ± 2,7 cm/yr, growth rate SDS was 0,10 ± 2,15. Growth SDS after 24 months therapy with etanercept has increased insignificantly and amounted -0,22 ± 1,68. The greatest increase in growth rate as adalimumab and etanercept recorded on the first 6 months of therapy. When comparing the growth rates and growth SDS in both groups showed no significant differences. A statistically significant increase in growth SDS observed on therapy with adalimumab.

**Conclusion:** Treatment TNF-α blockers increases the growth rate not only in the first year, but in the subsequent period of treatment. Timely biological therapy leads to stunting prevention in children with normal growth and reduces the already existing growth retardation.

**Disclosure of Interest**

None Declared.

### P350 Comic book as an educational tool for children with juvenile idiopathic arthritis

#### Veronica Moshe^1^, Gil Amarilyo^2^, Liora Harel^2^, Phillip J Hashkes^3^, Amir Mendelson^4^, Noa Rabinowicz^4^, Yonit Reis^4^, Yosef Uziel^1^

##### ^1^Pediatric Rheumatology Unit, Department of Pediatrics, Meir Medical Center, Sackler Faculty of Medicine, Tel Aviv University, Kfar Saba, Israel; ^2^Pediatric Rheumatology Unit, Schneider Children's Medical Center, Petah Tikva, Sackler Faculty of Medicine, Tel Aviv University, Tel Aviv, Israel; ^3^Pediatric Rheumatology Unit, Zedek Medical Center, Shaare Hebrew University, Medical School, Jerusalem, Israel; ^4^Pediatric Rheumatology Unit, Department of Pediatrics, Meir Medical Center, Kfar-Saba, Sackler Faculty of Medicine, Tel Aviv University, Tel Aviv, Israel

###### **Presenting author:** Veronica Moshe

**Introduction:** Knowledge is a very important tool in coping with chronic diseases. A comic book format was found to increase disease knowledge among patients with diabetes.

**Objectives:** To examine whether the comic book "Neta and the Medikids Explain JIA" would improve disease related knowledge and compliance of patients with juvenile idiopathic arthritis (JIA).

**Methods:** JIA patients answered 20 multiple choice knowledge questions about their disease, before and after reading the comic book. Demographic, clinical and health related quality of life data were recorded and correlated to knowledge.

**Results:** We studied 83 patients, mean age 14 ± 3.3 (8-18) years, 67% females, 33% males, 83% Jewish, 17% non-Jewish. 39% had oligoarthritis, 13% systemic and 48% polyarticular JIA. 46% had active disease, 40% were treated with biologics/DMARDs, 34% were in remission on medication. Among the 53 patients who completed both before and after questionnaires, correct answers increased from 62.5% to 81% (P < 0.001). Non-Jewish patients had an initial lower score of 48%, which increased to 79%.

27 patients who also completed the questionnaires one year after reading the book retained their knowledge (78.5%). We did not find a statistical significant correlation between knowledge and age, gender, ethnicity, disease subtype, and Child Health Questionnaire quality of life scores. The adherence to medication use, physiotherapy and follow-up rheumatologic visits were high at baseline; thus these did not change after reading the book.

**Conclusion:** The comic booklet "Neta and the Medikids Explain JIA" is a good educational tool for increasing disease related knowledge in children with JIA.

**Disclosure of Interest**

None Declared.

### P351 Temporomandibular joint magnetic resonance imaging findings correlated with subjective and objective symptoms in patients with juvenile idiopathic arthritis

#### Zane Dāvidsone^1^, Arina Lazareva^2^, Ruta Šantere^2^, Dace Bērziņa^2^, Valda Staņēviča^1^

##### ^1^Paediatrics, Riga Stradiņš University, Riga, Latvia; ^2^Paediatrics, Children`s University Hospital, Riga, Latvia

###### **Presenting author:** Zane Dāvidsone

**Introduction:** Juvenile idiopathic arthritis (JIA) is the most common rheumatic disease in children with frequent temporomandibular joint involvement (17-87%) [Argyropoulou, 2009]*.* The golden standard for TMJ arthritis diagnostics still is magnetic resonance imaging (MRI) with contrast enhancement [Müller*,* 2009]. TMJ arthritis can be asymptomatic even up to 71%, while TMJ clinical symptoms are with high specifity but low sensitivity [Cannizzaro, 2011; Twilt, 2004].

**Objectives:** To evaluate how TMJ MRI findings are correlating with subjective and objective symptoms in patients with JIA**.**

**Methods:** We analysed 78 JIA patients treated in Children`s University Clinical hospital who had MRI exam for TMJ during the period from 2010. to 2015. Most of them where with subjective and/or objective symptoms of TMJ involvement, but 12 were asymptomatic. Data where analysed using STATA program. Pearson`s correlation coeficient (r) and Fisher`s exact test where used. P < 0.05

**Results:** There were 78 JIA patients with mean age of 14.78 years (±1.14); 53(68%) girls and 25(32%) boys. JIA subtypes were as follows: seronegative polyarthritis 44(56%), seropositive polyarthritis 5(6.4%), olygoarthritis extended 6(56%), olygoarthritis persistent 2(2.6%), arthritis with enthesitis 16(20.5%), undifferentiated 3(4.5%), and 2(2.5%) systemic arthritis patients. Isolated MRI signs of active arthritis where in 7(58.3%) asymptomatic patients (r = 4.5, p = 0.034), but in 18%(27.3%) symptomatic patients. Isolated chronic damage signs had 1 symptomatic patient. Both active and chronic signs had 3(25%) asymptomatic patients and 38(58%) symptomatic (r = 4.3, p = 0.038). There where no MRI signs of arthritis in 2(17%) asymptomatic and 9(14%) symptomatic patients. Subjective complaints weakly correlated with a number of MRI signs (r = 0.27, p = 0.0173), objective signs count moderately correlated with MRI TMJ arthritis signs (r = 0,39, p = 0,0005). From 60 patients who had subjective complaints, 100% had also objective signs, but 6(31.6%) patients without subjective complaints had objective symptoms of TMJ involvement.

**Conclusion:** 1. Asymptomatic patients had MRI signs of arthritis rather often (83%)2. There where less patients with combination of active and chronic signs in asymptomatic patient`s group- only 25% compared with 58% symptomatic patients. 3. Objective signs had stronger correlation with MRI findings than subjective complaints and they can be found in the absence of subjective complaints.

**Disclosure of Interest**

None Declared.

## Poster Session: Juvenile dermatomyositis II

### P352 Comparison of treatment and outcome of children with juvenile dermatomyositis followed at two European tertiary care referral centers

#### Giulia Camilla Varnier^1^, Alessandro Consolaro^1^, Clarissa Pilkington^2^, Susan Maillard^2^, Cristina Ferrari^1^, Silvia Zaffarano^1^, Alberto Martini^1^, Angelo Ravelli^1^ and Juvenile Dermatomyositis Research Group and European Federation of Immunological Societies

##### ^1^IRCCS G.GASLINI, GENOVA, ITALY, Genova, Italy; ^2^Paediatric Rheumatology, Great Ormond Street Hospital, London, UK

###### **Presenting author:** Giulia Camilla Varnier

**Introduction:** The treatment of juvenile dermatomyositis (JDM) is likely variable across different pediatric rheumatology centers as no uniform therapeutic protocols are available. Furthermore, although it is common view that the recent therapeutic advances have markedly improved the disease prognosis, the impact of the newer treatment modalities on long-term outlook is still insufficiently documented.

**Objectives:** To compare the treatment approaches and disease outcomes of children with JDM followed in two large European tertiary care centers.

**Methods:** To be included in the study, patients had to be diagnosed with JDM between 2000 and 2015 at the Istituto Giannina Gaslini (IGG), Genoa, Italy or the Great Ormond Street Hospital (GOSH), London, UK within 6 months from disease onset and to have at least 6 months of follow-up. Data about treatment interventions and study outcomes were collected at fist observation and then every 3 months until 24 months. Outcomes included muscle strength/endurance, disease activity level and state, cumulative damage, and physical function.

**Results:** A total of 127 patients were included, 39 at IGG and 88 at GOSH. Demographic data, including age of onset and disease duration at first visit, were almost identical between patients followed at the two centers. At baseline, UK patients had more severe disease than Italian patients, as shown by lower median MMT-8 (46 vs. 67) and CMAS (23 vs. 34), higher DAS (15 vs. 12) and physician global assessment (5.5 vs 4.5), worse functional ability (1.6 vs. 0.8), and greater frequency of high disease activity (48.8% vs. 33.4%). Corticosteroids were used with equal frequency at GOSH and IGG (96.6% vs. 97.2%), whereas methotrexate (98.9% vs. 88.9%) and cyclophosphamide (34.1% vs. 2.8%) were administered more commonly at GOSH than at IGG, and cyclosporine (0% vs. 30.5%), iv Ig (9.1% vs. 22.2%) and hydroxychloroquine (8% vs. 55.5%) were prescribed less frequently at GOSH than at IGG. The proportion of patients who had normal muscle strength, no disease activity, no damage, and normal physical function at 6, 12 and 24 months is shown in the table.

**Conclusion:** Our study confirms the marked improvement in disease prognosis achieved with the current therapeutic approaches, which include marked reduction in cumulative damage. There were, however, several differences in the use of medications across the two hospitals, which underscores the need of international consensus efforts aimed to harmonize the therapeutic protocols for JDM.

**Disclosure of Interest**

None Declared.

**Table 39 (abstract P352).** See text for description

	6 months		12 months		24 months	
	GOSH (n = 88)	IGG (n = 36)	GOSH (n = 81)	IGG (n = 35)	GOSH (n = 71)	IGG (n = 34)
MMT-8 = 80	22/75 (29.3)	9/30 (30)	32/75 (42.7)	16/32 (50)	37/67 (55.2)	24/33 (72.7)
CMAS = 52	25/81 (30.9)	6/21 (28.6)	28/70 (40)	10/20 (50)	40/71 (56.3)	14/17 (82.3)
DAS = 0	15/86 (17.4)	6/32 (18.7)	24/74 (32.4)	10/34 (29.4)	40/71 (56.3)	17/29 (58.6)
MD global = 0	13/60 (21.7)	4/18 (22.2)	18/59 (30.5)	8/19 (42.1)	37/62 (59.7)	16/22 (72.7)
Normal physical function	21/59 (35.6)	8/13 (61.5)	28/64 (43.7)	9/10 (90)	36/59 (61)	14/17 (82.3)
MDI = 0	72/84 (85.7)	26/28 (92.9)	64/74 (86.5)	24/28 (85.7)	61/70 (87)	20/22 (90.9)
Inactive disease	13/86 (15.2)	11/35 (31.4)	28/74 (37.8)	17/33 (51.5)	51/70 (72.9)	23/30 (76.7)

### P353 Galectin-9, CXCL10 and TNFRII, biomarkers for disease activity in juvenile dermatomyositis, are myositis specific and may reflect highly activated endothelium

#### Judith Wienke^1^, Felicitas Bellutti Enders^2^, Lucas L. van den Hoogen^1^, Jorre S. Mertens^1^, Timothy R. Radstake^1^, Henny G. Hotten^1^, Ruth Fritsch^3^, Wilco de Jager^1^, Lucy Wedderburn^4^, Kiran Nistala^4^, Clarissa Pilkington^4^, Berent Prakken^5^, Annet van Royen-Kerkhof^5^, Femke van Wijk^1^

##### ^1^Laboratory of translational immunology, UMC UTRECHT, Utrecht, Netherlands; ^2^Pediatrics, Centre Hospitalier Universitaire Vaudois, Lausanne, Switzerland; ^3^Rheumatology & clinical immunology, UMC UTRECHT, Utrecht, Netherlands; ^4^Pediatric rheumatology, UCL Institute of Child Health, London, UK; ^5^Pediatric rheumatology & immunology, UMC UTRECHT, Utrecht, Netherlands

###### **Presenting author:** Judith Wienke

**Introduction:** Juvenile dermatomyositis (JDM) is a rare, but severe chronic systemic autoimmune disease in children, characterized by muscle weakness and a typical skin rash. Clinical evaluation of disease activity remains challenging. Recently, we identified and validated three proteins as robust biomarkers for disease activity in JDM, in two independent cohorts from the Netherlands and the United Kingdom: Galectin-9 (Gal-9), CXCL10 and TNFRII.(1) Endothelial cells are known to be important producers of these three proteins, and endothelial dysfunction has been implicated in the pathogenesis of JDM. We therefore further investigated endothelial function and activation, as reflected in peripheral blood serum, in patients with JDM compared to other pediatric and adult systemic autoimmune diseases.

**Objectives:** To assess the disease specificity of the biomarker potential of Gal-9, CXCL10 and TNFRII and investigate the peripheral profile of endothelial function and activation in JDM compared to related systemic autoimmune diseases.

**Methods:** We performed a multiplex immunoassay in patient’s serum of pediatric patients with JDM, lupus erythematosous (LE), mixed connective tissue disease (MCTD) and Morphea and adult patients with DM, systemic LE (SLE) and/or antiphospholipid syndrome (APS). The panel of 28 analytes comprised previously identified biomarkers for JDM and a combination of proteins reflecting endothelial function and activation. Data were analyzed using principal component analysis, hierarchical clustering and non-parametric ANOVA.

**Results:** Measurement of Gal-9, TNFRII and CXCL10 in this third JDM cohort confirmed their strength as biomarkers for disease activity. Comparison with other diseases revealed that their potential to discriminate between active disease and remission is specific for (J)DM. Principal component analysis of all measured analytes, including endothelial markers, separated the cohort into three main clusters of patients: myositis patients, SLE/APS patients and pediatric Morphea patients. Interestingly, all patients with myositis clustered together, regardless of their principal diagnosis ((S)LE, MCTD, JDM). The proteins mainly responsible for this separation were Gal-9, CXCL10, TNFRII, soluble VCAM-1 and ICAM-1, and CCL2. This indicates that the combination of these proteins is specific for myositis in general, rather than for JDM or systemic inflammation alone. In addition, we found evidence for a highly activated endothelial state and a disturbed balance between angiogenic (VEGF, angiopoeitin-1) and angiostatic (soluble VEGF-R1 and angiopoietin-2) proteins in JDM patients with active disease.

**Conclusion:** We confirmed the biomarker potential of Galectin-9, CXCL10 and TNFRII, which highly correlate with disease activity in juvenile dermatomyositis and seem specific for myositis, independent of the background disease. Introduction of these biomarkers into clinical practice will help to personalize treatment. The high levels of these proteins combined with the endothelial activation profile in patients with active disease points toward the involvement of both endothelial activation and dysfunction in the pathogenesis juvenile dermatomyositis.

**Reference**

1. Bellutti Enders FBE et al. Correlation of CXCL10, tumor necrosis factor receptor type II, and galectin 9 with disease activity in juvenile dermatomyositis. Arthritis Rheumatol. 2014 Aug;66(8):2281-9

**Disclosure of Interest**

None Declared

### P354 Effectiveness of rituximab therapy on severe calcinosis in 4 children with JDM

#### Mohammad Alhemairi, Mohammed Muzaffer

##### KING ABDUL AZIZ UNIVERSITY, JEDDAH, Saudi Arabia

###### **Presenting author:** Mohammed Muzaffer

**Introduction:** Juvenile dermatomyositis (JDM) is a rare systemic autoimmune vasculopathy that affects mainly skin and muscles.

Calcinosis is a dystrophic calcification which occurs in 20-40% of JDM patients. It is an important sequelae of JDM which may cause significant morbidity and mortality and can be difficult to treat.

There is no standard curative treatment for calcinosis but different agents were used with variable efficacy.

We report the favorable outcome of rituximab on severe calcinosis in 4 JDM patients and present their clinical data

**Objectives:** To report the observed effectivness of riuximab on JDM and associated calcinosis

**Methods:** A retrospective chart review of 4 children with JDM and severe calcinosis who were diagnosed at King Abdul aziz University Hospital,Jeddah,Saudi Arabia and received rituximab for relapsing or polycyclic JDM course.

Diagnosis and follow up of calcinosis was clinically and by X-ray.

Review data included :age of patients at onset of JDM symptoms and diagnosis,clinical and laboratory criteria at diagnosis,disease course and duration of follow up.

Data about calcinosis onset,sites,severity and its progression were also included.

Further data about rituximab therapy included :dosage,side effects,other treatment used before,during or after this drug and outcome and duration of follow up of calcinosis after therapy.

**Results:** 4 patients(2 male,2 female),interval between onset of symptoms was 6-12 months,course of JDM was polycyclic or relapsing,duration of follow up was 5-7 years.

Calcinosis was present in one patient at diagnosis,at 9,13 and 16 months after diagnosis.All patients had calcinosis affecting elbows,hands,thighs,knees,ankles and buttocks. Calcinosis was severe causing ulceration, recurrent skin infections, joint limitation,severe disability and inability to walk.It was not improving despite improvement in muscle and skin function with different DMARDs and /or biphosphnates, colchicine and warfarin

Reason to start rituximab was inadequate disease control, disability and recurrent relapses. Only one patient given 2 courses of rituximab; others given one course.

All patients received steroids and more than one DMARD before starting rituximab and were continued thereafter, follow up after rituximab was 3 to 5 years.

All patients had improvement in disease activity and frequency of admission especially due to complications of calcinosis. One patient had complete clearance of calcinosis for the last 5 years, others had significant improvement in calcinosis with no new lesions,decreased sites and density and decreased calcinosis related contractures. There was no serious side effects to rituximab.

**Conclusion:** In our cohort of 4 patients with JDM and severe calcinosis,there was significant improvement and/or clearance of calcinosis after rituximab therapy when used with other DMARDs as well as improvement in disease activity.Further clinical studies are required to evaluate the efficacy of rituximab in JDM and associated calcinosis.

**Disclosure of Interest**

None Declared

### P355 A Bayesian model for disease activity in patients with juvenile dermatomyositis

#### Pieter Van Dijkhuizen^1,2^, Claire T. Deakin^3^, Stefania Simou^3^, Lucy R. Wedderburn^3,4^, Maria De Iorio^5^

##### ^1^Paediatric rheumatology, University Medical Centre Utrecht, Wilhelmina Children's Hospital, Utrecht, Netherlands; ^2^Paediatric rheumatology, Istituto Giannina Gaslini, Genova, Italy; ^3^Paediatric rheumatology, University College London Institute of Child Health, London, UK; ^4^Paediatric rheumatology, Great Ormond Street Hospital for Children, London, UK; ^5^Department of Statistical Science, University College London, London, UK

###### **Presenting author:** Pieter Van Dijkhuizen

**Introduction:** Juvenile Dermatomyositis (JDM) is the most common form of juvenile idiopathic inflammatory myositis. It is a heterogeneous, systemic inflammatory disorder, involving mainly skeletal muscles and the skin, with a fluctuating disease course. Knowledge about clinical signs and symptoms determining a worse disease course is scarce.

**Objectives:** To develop a model associating clinical signs and symptoms with disease activity parameters in patients with JDM.

**Methods:** Data were available from an ongoing national longitudinal cohort study, enrolling newly diagnosed cases of JDM from across the UK. The model was developed in 340 subjects, contributing 2725 visits.

A Bayesian multivariate and multivariable linear mixed regression model was developed, using as outcome the disease activity parameters creatine kinase level (CK), childhood myositis activity score (CMAS), manual muscle testing in 8 muscle groups (MMT) and the physician’s global assessment of disease activity (PGA). Clinical signs and symptoms were used as covariates.

The goodness of fit of the model was tested in a random sample of subjects, whereas the out-of-sample predictions were tested in a random sample of subjects not used for the model development.

**Results:** The majority of included patients was female (69.4%), the median age at diagnosis was 7.4 years (interquartile range [IQR] 4.5-10.5) and patients were enrolled shortly after diagnosis (median of 0.2 years [IQR 0.1-1.1]).

The model’s goodness of fit and out-of-sample predictions were accurate and followed observed patterns over time well. Various clinical signs and symptoms were shown to be associated with the disease activity parameters.

Of signs and symptoms at diagnosis, only arthritis was associated with a higher CMAS and MMT score.

Presence of myalgia and dysphonia at the visit was associated with higher disease activity with regard to all outcome parameters, as expected. Several cutaneous and soft tissue signs and symptoms, such as periorbital rash, rash on the trunk, rash over large joints, nail fold changes and facial swelling, were associated with higher disease activity with regard to various outcome parameters.

In addition, presence of contractures was associated with a higher disease activity with regard to the CMAS, MMT and PGA. Joint swelling was associated with higher disease activity regarding the CMAS and PGA, but there was a tendency for CK levels to be lower.

Patients with haematuria had higher CK values, but tended to have a lower PGA.

Calcinosis was mostly seen as long-term outcomes of the disease, as evidenced by an association with higher CMAS scores. However, the PGA was higher in these patients too.

Patients with a higher CMAS value tended to have a higher MMT value as well, since the estimated correlation between the by-patient intercepts for CMAS and MMT was 0.54 (95% credible region [CR] 0.42-0.65). Correlations among other outcome parameters were low (≤0.23).

Given that a visit was estimated to be in remission according to PRINTO criteria, the estimated median probability that PGA was pathological, whereas the other three outcome parameters were non-pathological, was 51% (95% CR 0-99%). It was 6% (95% CR 0-92%), 1% (95% CR 0-35%) and 1% (95% CR 0-64%) for CK, CMAS and MMT, respectively.

**Conclusion:** The model elucidated various clinical signs and symptoms associated with disease activity, which can be used to stratify patients according to their prognosis and guide treatment decisions accordingly. Furthermore, the model brought to light similarities and dissimilarities among the four outcome parameters, useful in the discussion of the set of outcome parameters for JDM.

**Disclosure of Interest**

None Declared

### P356 Audit of intravenous immunoglobulin protocols used for the treatment of juvenile dermatomyositis

#### Qiong Wu^1^, Tania Amin^1^, Stefania Simou^2^, Lee Dossetter^1^, Lucy R. Wedderburn^1,2^, Clarissa Pilkington^1^ and Juvenile Dermatomyositis Research Group (JDRG)

##### ^1^Paediatric Rheumatology Department, Great Ormond Street Hospital for Children, London, UK; ^2^Infection, Immunity, Inflammation, and Physiological Medicine Programme, Institute of Child Health, University College London, London, UK

###### **Presenting author:** Qiong Wu

**Introduction:** Children with poorly controlled Juvenile Dermatomyositis (JDM) can develop permanent loss of muscle function and skin scarring. For some children with severe disease, after failure of 2nd line medications, Intravenous Immunoglobulin (IVIG) can successfully control inflammation. However, in certain cases, it can precipitate serious side effects including aseptic meningitis and renal impairment. Various IVIG infusion protocols have been trialled at a number of tertiary Paediatric Rheumatology centres, and in 2010, the Children's Arthritis and Rheumatology Research Alliance (CARRA) recommended loading IVIG at 0, 2 and 4 weeks before stretching to monthly infusions. However, little data comparing the complication rate of different protocols have been published.

**Objectives:** We audited protocols which have been used at Great Ormond Street Hospital, and compare complications profile of these protocols.

**Methods:** Children with JDM treated with IVIG were identified using the UK JDM Cohort and Biomarker Study (JDCBS) database. Dates of IVIG infusions, complications and disease activity measures during first 24 months of IVIG were collected.

**Results:** Twenty children who received IVIG between 2008 and 2015 were identified. Ten were treated using the CARRA protocol (age range 2-14 years, median age 7 years, M:F ratio 1:2.3), while 10 were treated with less intensive protocols, most commonly monthly infusions (age range 3-13 years, median age 7 years, M:F ratio 1:2.3). Prior to 2010, no children were treated with the CARRA protocol, but since 2010, 56% of children have been treated with this protocol. At baseline, CARRA-treated group had mean physician visual analogue scale (phyVAS) of 5/10, parent VAS (parVAS) of 8/10 and MMT8 of 28/80. 70% of children had skin rash, 75% had nailfold abnormalities, 0% had calcinosis and 20% had ulceration. There was a mean of 5 months from diagnosis prior to first IVIG infusion. The less intensive protocol group had physVAS of 4/10, parVAS of 3/10 and MMT8 of 67/80. All children had skin rash, 89% had nailfold abnormalities, 33% had calcinosis and 18% had ulceration. There was a mean of 32 months from diagnosis prior to first IVIG infusion. In both groups, children were treated with a range of disease modifying anti-rheumatic drugs prior to and/or alongside IVIG infusions. A number of children were also pulsed with IV steroids in combination with IVIG. Two out of the 10 children on CARRA protocol experienced complications, while 7 out of the 10 children on less intensive regimens reported complications. At 24 months, both groups showed improvements in physVAS, parVAS, MMT8, skin rash and nailfold abnormalities.

**Conclusion:** These preliminary data do not suggest an increased complication rate of 0, 2, 4 week IVIG loading in the treatment of JDM compared to monthly infusions. A larger multi-centre study to audit the complication profile of various IVIG regimens is warranted.

**Disclosure of Interest**

None Declared

### P357 Efficacy and safety of tumour necrosis factor-αlfa antagonists in a large cohort of juvenile dermatomyositis patients

#### Raquel Campanilho-Marques^1,2,3,4^, Claire Deakin^1^, Stefania Simou^1^, Lucy R. Wedderburn^1,5^, Clarissa A. Pilkington^2^ on behalf of Juvenile Dermatomyositis Research Group (JDRG)

##### ^1^Infection, Inflammation and Rheumatology Section, Institute of Child Health - UCL, London, UK; ^2^Rheumatology, Great Ormond Street Hospital for Children NHS Trust, London, UK; ^3^Rheumatology, Santa Maria Hospital - CHLN, Lisbon, Portugal; ^4^Rheumatology, Instituto Português de Reumatologia, Lisbon, Portugal; ^5^Arthritis Research UK Centre for Adolescent Rheumatology, UCL, UCLH, GOSH NHS Trust, London, UK

###### **Presenting author:** Raquel Campanilho-Marques

**Introduction:** Some patients with juvenile dermatomyositis (JDM) have a disease course which is refractory to multiple drug treatments. There is evidence that prolonged disease activity is associated with increased mortality and morbidity. High levels of anti-TNFα have been reported in JDM patients with a long disease course, suggesting it may play a significant role in refractory disease. There are no published clinical trials of this therapy but some are in progress.

**Objectives:** To evaluate the efficacy and safety of anti-TNFα treatment in UK JDM Cohort and Biomarker Study patients.

**Methods:** Data were analysed from children who were recruited to the UK JDM Cohort and Biomarker Study, met Bohan-Peter criteria and were on anti-TNF treatment at the time of analysis, and had had at least 3 months of therapy. Childhood Myositis Assessment Scale (CMAS), Manual Muscle Testing (MMT8), muscle enzymes and physicians global assessment (PGA) were recorded. Skin disease was assessed using modified skin Disease activity score (DAS).

**Results:** 66 patients with JDM actively treated with anti-TNF agents were analyzed. 41 patients were female (62%). The current mean (±SD) age of these patients was 16.8 ± 5.6 years old, the mean age at diagnosis was 7.31 ± 4.04 years old and the mean disease duration was of 9.6 ± 4.6. Mean disease duration at the beginning of anti-TNF treatment was 3.49 ± 2.80 years and mean duration of anti-TNF therapy was of 2.76 ± 2.09 years. Muscle involvement significantly improved, with median [IQR] CMAS and MMT8 values at initiation of anti-TNF therapy of 45.50 [39.75-52.25] and 74 [59.5-79.5] respectively, and at current evaluation (or date of anti-TNF treatment completion) of 53 [50-53] and 79 [74.580] (p < 0.0001 and p = 0.0097; Mann Whitney test), respectively. For skin involvement the initial modified DAS was 4 [2-5] and final 1 [0-3] (p < 0.0001; Mann Whitney test). Assessing global disease activity the initial PGA was 2.9 [1.3-4.3] and final 0.5 [0-1.45] (p < 0.0001; Mann Whitney test). Sixteen patients (24%) switched their anti-TNF treatment. 62.5% of the switches were due to therapy failure, 25% due to adverse events and 12.5% for patient preference in subcutaneous administration. Of 21 adverse reactions registered, 7 were considered severe (anaphylactic reactions on infliximab infusion). One patient died due to small bowel perforation (not felt to be related to the use of TNF antagonists). The remaining adverse reactions were not severe and 77% (n = 10) of them were due to infections causes. In 4 of the mild to moderate adverse reactions the drug had to be discontinued and switched to another TNF antagonist, while in the remaining patients temporarily withholding the drug proved sufficient. No tuberculosis case was registered.

**Conclusion:** This study is one of the largest to explore the efficacy and safety of TNF antagonist treatment in a large independent cohort of JDM patients. Both muscle and skin involvement appeared to improve after anti-TNF treatment.

**Disclosure of Interest**

None Declared

### P358 Development and validation of a composite disease activity score for juvenile dermatomyositis

#### Silvia Rosina^1^, Alessandro Consolaro^1^, Pieter van Dijkhuizen^1^, Kiran Nistala^2^, Nicola Ruperto^1^, Clarissa Pilkington^2^, Angelo Ravelli^1^

##### ^1^Rheumatology, Giannina Gaslini Institute, Genova, Italy; ^2^Rheumatology, Great Ormond Street Hospital, London, UK

###### **Presenting author:** Silvia Rosina

**Introduction:** Juvenile dermatomyositis (JDM) is a multisystem vasculopathic disease that primarily affects the skin and muscles. Most tools for assessment of disease activity in JDM are centered on physician’s evaluation, whereas parent’s or child’s views are largely neglected. Furthermore, existing instruments are frequently lengthy and complex.

**Objectives:** To develop a composite disease activity score for JDM and provide preliminary evidence of its validity.

**Methods:** A panel of experts devised the composite disease activity score, named Juvenile DermatoMyositis Activity Index (JDMAI), based on their clinical experience and a literature review. The JDMAI is composed of 4 clinical measures or domains: 1) physician’s global assessment of overall disease activity on a 0-10 VAS; 2) parent’s/child’s global assessment of child’s wellbeing on a 0-10 VAS; 3) muscle strength/endurance; 4) skin disease activity. Eight versions of the JDMAI were tested, which differed in the tools used to assess items 3 and 4. For item 3, two versions included the hybrid MMT/CMAS (hMC) with score in deciles (0-10), two the hMC with its original score (0-100), two the MMT-8 (0-80), and two the CMAS (0-52). For item 4, four versions included physician’s global rating of skin disease activity on a 10-cm VAS (0 = no activity, 10 = maximum activity) and four included the cutaneous domain of the Disease Activity Score (DAS) (0-9). Validation was conducted on 275 patients included in a multinational dataset, evaluated at baseline and at 6, 12, and 24 months. Construct validity was assessed by calculating between-subject and within-subject correlations with JDM outcome measures not included in the JDMAI; internal consistency was assessed with Cronbach α and responsiveness to change with standardized response mean (SRM). Discriminant ability was determined in a different multinational dataset of 136 patients, by assessing the JDMAI score in patients rated in remission, low, moderate, or high disease activity by the attending physician.

**Results:** In between-subject exercise, all JDMAI versions showed strong (r > 0.7) correlations with CHAQ (0.72-0.82), physician’s global rating of muscle disease activity on a 10-cm VAS (0.77-0.87), muscle DAS (0.76-0.86) and total DAS (0.68-0.90), and moderate correlations (r = 0.4-0.7) with pain VAS (0.50-0.57) and Myositis Damage Index (MDI) (0.51-0.60). Owing to the interrelatedness of longitudinal data from an individual patient, within-subject correlations were higher and were all strong (r = 0.76-0.97). SRM was good (1.09-1.57) and was higher for JDMAI 1 and 2. Cronbach’s alpha was fair (0.70 and 0.69) for JDMAI 1 and 2, and poor for other JDMAI versions. All JDMAI versions discriminated strongly between patients in different disease activity states (Kruskal-Wallis test, p < 0.001).

**Conclusion:** Overall, the JDMAI1 and JDMAI2 revealed the best measurement properties in validation analyses. The JDMAI1 (score range: 0-40) may be preferred over the JDMAI2 as it weights equally its 4 components, whereas in the JDMAI2 (score range: 0-39) items 1 to 3 are scored on a 0-10 scale and skin disease on a 0-9 scale.

**Disclosure of Interest**

None Declared

**Table 40 (abstract P358).** See text for description

JDMAI 1	JDMAI 2	JDMAI 3	JDMAI 4	JDMAI 5	JDMAI 6	JDMAI 7	JDMAI 8
Physician’s global assessment (0-10)							
Parent’s global assessment (0-10)							
hMC in deciles (0-10)*		hMC (0-100)*		MMT-8 (0-80)*		CMAS (0-52)*	
Skin VAS (0-10)	Skin DAS (0-9)	Skin VAS (0-10)	Skin DAS (0-9)	Skin VAS (0-10)	Skin DAS (0-9)	Skin VAS (0-10)	Skin DAS (0-9)
0-40	0-39	0-130	0-129	0-110	0-109	0-82	0-81

See text for abbreviations. *: scores were reversed for consistency with other parameters

### P359 Evaluation predictors of the cumulative dose and duration of corticosteroid and methotrexate treatment in juvenile dermatomyositis

#### Sirisucha Soponkanaporn^1,2^, Stefania Simou^1^, Claire T. Deakin^1^, Lucy R. Wedderburn^1^ on behalf of the UK Juvenile Dermatomyositis Research Group (JDRG)

##### ^1^Infection Inflammation and Rheumatology Section, Institute of Child Health, University College London, London, UK; ^2^Paediatrics, Faculty of Medicine Ramathibodi Hospital, Mahidol University, Bangkok, Thailand

###### **Presenting author:** Sirisucha Soponkanaporn

**Introduction:** Juvenile dermatomyositis (JDM) is a heterogeneous autoimmune-mediated disease characterised by muscle weakness, rash and other organ features. Corticosteroids and methotrexate are the mainstay of treatment. However, some patients are still unable to discontinue the treatment and some have high cumulative dose of these drugs.

**Objectives:** This study investigated whether myositis-specific autoantibodies (MSA), demographic data or clinical features at first presentation can predict the cumulative dose and duration of corticosteroid and methotrexate treatment in JDM.

**Methods:** 230 of the patients enrolled in the Juvenile Dermatomyositis Cohort and Biomarker Study (JDCBS) were tested for autoantibodies and treated at Great Ormond Street Hospital, between January 2000 and January 2016. Inclusion criteria were positive tests for anti-TIF1ɣ**,** anti-NXP-2, anti-MDA-5, anti-Mi-2, anti-SRP or anti-Jo-1. Patients were excluded if they were in groups with insufficient numbers for statistical analysis, incomplete records, follow-up period less than 2 years, or intolerance with adverse events from the treatment. Linear regression analysis was performed to assess MSA and demographic variables as predictors of the treatment course. The strength of the association was described by the standardised coefficient (β). Kaplan-Meier survival analysis was used to compare the proportion of patients being off treatment over time across MSA subgroups.

**Results:** 81 and 80 patients fulfilled criteria for analysis of corticosteroid and methotrexate treatment, respectively. The median age at disease onset was 5.6 years (IQR 3.7-8.9) and the median follow up period was 79.3 months (IQR 51.9-112.7). Anti-TIF1**ɣ**-positive patients tended to have a longer duration of corticosteroid usage and a higher cumulative dose of corticosteroids and methotrexate compared to other MSA subtypes. However, the differences were not statistically significant. Older age at disease onset correlated significantly with lower cumulative dose of corticosteroids (standardised β -0.404, 95% confidence interval [CI] -46.39, -15.01, P < 0.001) and methotrexate (standardised β -0.298, 95%CI -329.27, -45.24, P 0.010), as well as shorter duration of methotrexate usage (standardised β -0.279, 95%CI -151.16, -14.55, P 0.018). Only female gender was found to be a predictor of shorter duration of corticosteroid usage (standardised β -0.235, 95%CI -725.88, -17.94, P 0.040).

**Conclusion:** Although MSA did not show significant associations with the outcome tested, there was a trend in particular MSA subgroups. To illustrate, anti-TIF1ɣ autoantibodies appeared to be associated with longer duration of corticosteroid and methotrexate usage than other MSA subgroups. In contrast, patients with anti-MDA-5 autoantibodies appeared to have a trend towards shorter duration of corticosteroid usage and lower cumulative dose of corticosteroids. Only age at disease onset and gender were found to be predictors of treatment outcome. These findings may improve clinicians awareness of which patients need higher doses and longer term treatment and provide clinicians with a stratified approach to counseling patients and family.

**Disclosure of Interest**

None Declared

### P360 The relationship between myositis specific autoantibodies and disease phenotypes (and capillaroscopy findings) in patients with juvenile dermatomyositis: case series

#### Zehra S. Arıcı^1^, Gökçen D. Tuğcu^2^, Ezgi D. Batu^1^, Hafize E. Sönmez^1^, Deniz Doğru-Ersöz^2^, Yelda Bilginer^1^, Beril Talim^3^, Nural Kiper^2^, Seza Özen^1^

##### ^1^Department of Pediatric Rheumatology, Hacettepe University Faculty of Medicine, Ankara, Turkey; ^2^Department of Pediatric Pulmonology, Hacettepe University Faculty of Medicine, Ankara, Turkey; ^3^Department of Pediatric Pathology, Hacettepe University Faculty of Medicine, Ankara, Turkey

###### **Presenting author:** Zehra S. Arıcı

**Introduction:** Juvenile dermatomyositis (JDM) is a heterogeneous disease. It is believed that autoantibodie**s** are important markers to predict prognosis. It has been reported that anti-NXP2 autoantibodies are associated with calsinosis and anti-MDA5 autoantibodies are associated with interstitial lung disease

**Objectives:** In this study, we investigated the relationships among JDM specific autoantibodies, phenotype and capillaroscopy findings in a single center.

**Methods:** In our 37 JDM patients, clinical and laboratory data were collected. Specific autoantibody panel was viewed. Capillaroscopy was performed in all patients. All patients were evaluated by a specialist pulmonologist and required examinations were performed.

**Results:** In this study, 22 girls (59,5%) and 15 boys (40,5%) with JDM were evaluated. The mean age of onset symptoms was 8,4 years (2,4-15 years). The mean time from disease onset to treatments was 7 months (1-25 months). The mean duration of patient follow-up was 4,8 years (0,7-15,5 years).

Anti-MDA5 was found in four patients (10,8%). Interstitial lung disease was observed in one patient with strong positive anti-MDA5 and led to treatment change. There was no interstitial lung disease in other three patients with mild to moderate positive anti- MDA5. None of the patients with anti-MDA5 negative had lung disease.

Anti-NXP2 was found in six patients (16,2%). One patient with Anti-NXP2 positive had deep skin ulcers at the disease initial. Four patients with Anti-NXP2 positive had calcinosis.

There were 9 patients with calcinosis (25%). Two of them had anti-MDA5 antibodies. Five patients had NXP2 antibodies. Anti-TIF1γ was found one patient with calcinosis. No antibodies were positive in one patient. The mean time from disease onset to treatments was significantly longer patients with calcinosis (10-27 months) than without (1-4 months).

In five of seven patients with anti-TIF1-γ positive, there were persistent skin findings. These were heliotrope rash and Gottron's papules. In one of patient with anti-TIF1-γ negative, there were serious skin ulcers. This patient had anti-NXP2 positivity.

There was no significant relationship between autoantibodies and capillaroscopy findings.

**Conclusion:** This study indicates that the most important factor in preventing calcinosis is early and effective treatment. Anti-NXP2 was the most common autoantibody in our small JDM patients with calcinosis. It has been shown that the relationship between Anti-TIF1-γ and resistant skin lesions in our patients.

**Disclosure of Interest**

None Declared

## Poster Session: Miscellaneous rheumatic diseases

### P361 Arthritis, subcutaneous nodules, hoarseness or peripheral osteolysis can all be symptoms of farber disease (acid ceramidase deficiency): data from an expanding cohort of patients

#### Alexander Solyom^1^, Boris Hügle^2^, Balahan Makay^3^, Bo Magnusson^4^, Ezgi Batu^5^, John Mitchell^6^, Ariana Kariminejad^7^, Fatemeh Hadipour^8^, Zahra Hadipour^8^, Marta Torcoletti^9^, Carlo Agostoni^9^, Maja Di Rocco^10^, Pranoot Tanpaiboon^11^, Andrea Superti-Furga^12^, Luisa Bonafé^12^, Nur Arslan^3^, Norberto Guelbert^13^, Mikhail Kostik^14^, Karoline Ehlert^15^, Giedre Grigelioniene^4^, Ratna Puri^16^, Seza Ozen^5^, Edward Schuchman^17^

##### ^1^Plexcera Therapeutics LLC, New York, USA; ^2^German Center for Pediatric Rheumatology, Garmisch-Partenkirchen, Germany; ^3^Dokuz Eylul University Hospital, Izmir, Turkey; ^4^Karolinska University Hospital, Stockholm, Sweden; ^5^Hacettepe University Hospital, Ankara, Turkey; ^6^Montreal Children's Hospital, Montreal, Canada; ^7^Kariminejad-Najmabadi Pathology & Genetics Center, Tehran, Islamic Republic of Iran; ^8^Sarem Cell Research Center & Hospital, Tehran, Islamic Republic of Iran; ^9^IRCCS Policlinico, University of Milan, Milan, Italy; ^10^Istituto Giannina Gaslini, Genoa, Italy; ^11^Children's National Medical Center, Washington DC, USA; ^12^Lausanne University Hospital (CHUV), Lausanne, Switzerland; ^13^Children's Hospital of Cordoba, Cordoba, Argentina, ^14^State Pediatric Medical Academy, St. Petersburg, Russian Federation; ^15^University Medical Center Greifswald, Greifswald, Germany; ^16^Sir Ganga Ram University Hospital, New Delhi, India; ^17^Icahn School of Medicine at Mt. Sinai, New York, USA

###### **Presenting author:** Alexander Solyom

**Introduction:** Farber disease (Farber lipogranulomatosis; acid ceramidase deficiency) is a rare, autosomal recessively inherited lysosomal storage disorder, resulting from a mutation in the *ASAH1* gene causing a deficiency of acid ceramidase, and the accumulation of its pro-inflammatory and pro-apoptotic lipid substrate, ceramide. The cardinal symptoms of Farber disease are arthritis, subcutaneous nodules and a hoarse or weak voice. The phenotypic spectrum is very broad and primarily includes symptoms which may lead to evaluation by a pediatric or adult rheumatologist. Many patients show pronounced signs of inflammation (arthritis/synovitis, pain, elevated C-reactive protein (CRP) and/or erythrocyte sedimentation rate (ESR), even recurring fevers). Peripheral osteolysis may also be a sign of Farber disease. The clinical similarity between moderate Farber phenotypes and polyarticular juvenile idiopathic arthritis (JIA) poses problems in the differential diagnosis. Currently, Farber disease can be treated with hematopoietic stem cell transplantation, which has shown variable results and carries a severe burden for the patients. Enzyme replacement therapy for Farber disease is being developed, with expected availability for clinical trials from late 2016.

**Objectives:** To collect clinical information on Farber disease patients relevant to diagnostics, treatment, care and prognosis.

**Methods:** Since 2014, an international cohort of Farber patients has been assembled. Prospective follow-up and retrospective chart review was used to gather data.

**Results:** 40 patients with Farber disease have been included in the cohort to date, with ages ranging from infancy to 60 years of age. The cardinal symptoms of polyarticular arthritis, subcutaneous nodules, and a hoarse or weak voice were seen in almost all patients, although there is great variability in the severity of symptoms, as well as in the time elapsed between the appearance of each symptom. Almost all moderately or mildly affected patients were previously diagnosed with JIA, and received treatments ranging from nonsteroidal anti-inflammatory drugs to disease modifying anti-rheumatic drugs and biologics.

**Conclusion:** Farber disease is probably more common than previously thought. In particular, early-onset polyarticular JIA patients with an inadequate response to conventional therapies should be evaluated for acid ceramidase deficiency and *ASAH1* mutation testing. Further efforts to better define the natural history of Farber disease, to advance physician awareness, diagnostics, treatment, and to address the needs of patients and families are planned.

**Disclosure of Interest**

A. Solyom Employee of: Plexcera Therapeutics LLC, B. Hügle: None Declared, B. Makay: None Declared, B. Magnusson: None Declared, E. Batu: None Declared, J. Mitchell: None Declared, A. Kariminejad: None Declared, F. Hadipour: None Declared, Z. Hadipour: None Declared, M. Torcoletti: None Declared, C. Agostoni: None Declared, M. Di Rocco: None Declared, P. Tanpaiboon: None Declared, A. Superti-Furga: None Declared, L. Bonafé: None Declared, N. Arslan: None Declared, N. Guelbert: None Declared, M. Kostik: None Declared, K. Ehlert: None Declared, G. Grigelioniene: None Declared, R. Puri: None Declared, S. Ozen: None Declared, E. Schuchman Shareholder of: Plexcera Therapeutics LLC

### P362 Polyautoimmunity in Colombian juvenile patients

#### Clara Malagon^1^, Pilar Gomez^2^, Angela C. Mosquera^1^, Tatiana Gonzalez^3^, Ricardo Yepez^2^, Camilo Vargas^4^ and GRIP study group

##### ^1^Pediatric Rheumatology, El Bosque University, Bogota, Colombia; ^2^Pediatric Rheumatology, Universidad Libre, Cali, Colombia; ^3^Pediatric Rheumatology, Universidad de Cartagena, Cartagena, Colombia; ^4^Pediatric Rheumatology, Universidad del Valle, Cali, Colombia

###### **Presenting author:** Clara Malagon

**Introduction:** Polyautoimmunity is defined as the presence of more than one autoimmune disease (AiD) in a single patient and multiple auto immune syndrome when three or more autoimmune diseases coexist. The fact that many AiDs share a similar pathogenic mechanisms including: susceptibility genes, epigenetic factors and aberrant immune responses may explain the development of multiple AiDs in a host. AiDs may cause similar symptoms, determine important morbidity and have the tendency to developed in clusters is important for clinicians to have an high index of clinical suspiction and search periodically clinical and serological test to identify those associations of AIDs.

**Objectives:** From de clinical observation of several juvenile patients developed more than one AIDs during follow up at pediatric rheumatology clinics, the members of the GRIP study group developed a registry and study the data obtained to determine their demographic and clinical features

**Methods:** GRIP study group developed a registry of patients followed at 10 pediatric rheumatology clinics in 4 Colombian cities who developed more than one AiD according to international validated diagnostic criteria for organ specific and systemic AIDs.The clinical charts were reviewed and the demographic, family history, clinical, serological and histopathological data were collected in an electronic database. Patients with features of undefined auto immune disease, overlap syndrome or mixed connective tissue disease were excluded to avoid bias. AiDs were reported on a chronologic order and the first one was named Heralding AiD. The intervals between them were calculated on months or defined as simultaneous when were identified with a interval less than 4 weeks. The information is updated as needed if the patient developed additional AiD after was included at the registry. Data was studied using a program SSPS 15 version.

**Results:** N = 216 . The sex ratio was female 9.3: male 1 and was similar on patients who developed 2,3 or 4 AiDs. 166/216 patients develop two AiDs at the time of the data analysis. 46/216 3 diseases and four patients associated four AiDs.The age of onset varies from 0-16 years for the first AiDs and were also similar in the three groups. The interval between AiDs were longer between 1o to 2o with a shorter period to develop additional AiDs. 27% develop simultaneously the first two AiDs, 28% the 2o and 3o and 50% the 3o and 4o. 32% had a positive history of familial autoimmunity and it was similar between groups. 22 AiDs were identified in different associations. The most common systemic AiD was SLE while Hashimoto disease was the most frequent organ specific disease but uncommon diseases as Myasthenia gravis, Rupus, Kikuchi Fujimoto or autoimmune pancreatitis were also present.The most common heralding diseases were: SLE, Hashimoto, APLS, autoimmune citopenias and JIA. The most common associations of 2 AIDs were: LES + APLS(49), JIA + Hashimoto(13), LES+ Hashimoto(12), APLS + LES (12), for 3 AiDs : SLE+ APLS + Hashimoto(5), JIA + APLS + Hashimoto (2), JIA + Hashimoto + ALPS (2) and 4 AiDs different associations on each patient.

**Conclusion:** Polyautoimmunity is present in juvenile as well adults patients. Some associations are common and should be routinely searched. Other associations had not been reported or are considered unusual.Systemic AiDs and isolated Autoimmune citopenias, endocrinopathies, nephropathies, dermatologic diseases and uveitis may herald the development of additional AiDs after a variable period of time, including when they are on remission.New symptoms, changes of functional test of target organs or changes of autoantibody profile may alert about polyautoimmunity. Common associations of AID´s should be searched on a regular basis.

**Disclosure of Interest**

None Declared

### P363 PANDAS and PANS: underestimated and unrecognized neuropsychiatric syndromes in children

#### Falcini Fernanda^1^, Gemma Lepri^1^, Alessandra Ferrari^2^, Donato Rigante^3^, Marco Matucci-Cerinic^1^, Antonella Meini^2^

##### ^1^Section of Rheumatology, Transition Care, UNIVERSITY OF FLORENCE, Florence, Italy; ^2^Pediatric Clinic, University of Brescia and Spedali Civili di Brescia, Brescia, Italy; ^3^Institute of Pediatrics, Università Cattolica Sacro Cuore, Roma, Italy

###### **Presenting author:** Falcini Fernanda

**Introduction:** The term PANDAS (Pediatric Autoimmune Neuropsychiatric Disorders associated with Streptococcal Infections) identifies a group of pts with abrupt and dramatic TIC/OCD onset GAS infection related. In PANS (Pediatric acute-onset neuropsychiatric syndrome), other agents may trigger the disease, including *Mycoplasma pneumonia, Influenza viruses, EBV, Borrelia Burgdorferi, Herpes simplex, and VVZ.* In both conditions tics, OCD and additional neuropsychiatric symptoms are not explained by other known neurologic or medical disorders. Due to disputes about PANDAS/PANS, many children are underdiagnosed and often receive ineffective and harmful antipsycotic drugs.

**Objectives:** 1. To highlight the knowledge of PANDAS/PANS aimed to an earlier diagnosis and treatment. 2. To report our experience in a large cohort of PANDAS/PANS pts.

**Methods:** Between May 2009 through January 2016, we recruited 333 pts, (243 M,93 F) with tics/OCD starting before puberty, mean age at onset77 months ± 35.6SD. In 310 clinical manifestations were GAS related, in 23 to other infectious agents. Demographic, familiar data about neurologic disorders, laboratory data were assessed. All pts underwent EEG, ECG, echocardiography and neuropsychiatric consultation before or after our first evaluation. Brain MRI was performed in 122/333 patients.

**Results:** 317/325 pts(94.1%) were full-term born, 78/322 (21.6%) after Caesarean section, most were breastfed. All had a normal weight, height and psychological development. 8/333 pts were adopted children. The clinical onset was dramatically sudden and unexpected in all pts. The early symptoms, tics/OCD were underestimated and endorsed to the birth of brother/sister, family events, school pressure. Histories were characterized by remissions and recurrences of symptoms. Inflammatory parameters: ESR, CRP, fibrinogen were in all normal, both at onset, at our first clinical evaluation, and at follow-up. Thyroid function (except 1 pt), coeliac disease screening, EEG, ECG and echocardiography were regular. Brain MRI was normal in all tested patients. ASLO and antiDNasiB titers were evaluated in all pts at onset and at the first assessment, both were increased in PANDAS pts. ANAs were positive at a low titer in two PANDAS pts, negative in PANS. Anti-dsDNA, anti-ENA, anti-cardiolipin antibodies, and LAC were negative in PANDAS and in PANS pts. Most pts have a good performance both at primary and high level school. Therapy: all pts received amoxicillin + clavulanic acid 80/100 mg/Kg for 2-4 weeks and then Benzathine benzylpenicillin 600.000/1.200.000 every 21 days, magnesium and Vit D supplementation. All were evaluated by a Psycologist and 25 were treated by a Psychotherapist. Psychotropic drugs were used in 28/333 pts before our first assessment, all ineffective before starting antibiotic therapy. Eight pts received IVIg according to schedule suggested by S. Sweedo with different results. All pts have been evaluated every 6 months. In the group with only tics we observed a remission of symptoms by 2 to 6 months while OCD had amelioration by 6-12 months.

**Conclusion:** Our data show that PANDAS/PANS affect mainly males with higher familiar predisposition. The age at onset is comparable in both groups, while OCD are more common and severe in PANS than in PANDAS. PANS pts have different triggers: *Mycoplasma, Borrelia burgdorferi* and VZV, and all responded to the specific therapy. The knowledge of PANDAS/PANS is mandatory to early recognize children at the disease onset avoiding to delay diagnosis and therapy. Doctors have a moral obligation to know and to identify these two entities, so that patients and their families can avoid unnecessary calvary through numerous specialists with progressive worsening of clinical symptoms.

**Disclosure of Interest**

None Declared

### P364 Type I interferon score in peripheral blood of patients with pediatric rheumatic diseases

#### Gian Marco Moneta^1^, Ivan Caiello^1^, Emiliano Marasco^1^, Rebecca Nicolai^1^, Manuela Pardeo^1^, Claudia Bracaglia^1^, Antonella Insalaco^1^, Luisa Bracci-Laudiero^1,2^, Fabrizio De Benedetti^1^

##### ^1^Division of Rheumatology, Bambino Gesù Children’s Hospital, Rome, Italy; ^2^Institute of Translational Pharmacology, CNR, Rome, Italy

###### **Presenting author:** Gian Marco Moneta

**Introduction:** Type I interferons (IFNs) are proteins that provide protection from viral infections, through induction of hundreds of genes implicated in antiviral response, the so-called “IFN signature”. In the last years, the term “type I interferonopathy (IFNpathy)” has been used to identify a group of monogenic diseases in which the causative mutation determines a constitutive up-regulation of type I IFN activity directly relevant for pathogenesis. A restricted set of 6 genes has been proposed and widely used to identify patients with potential type I IFNpathies, which is commonly referred to as type I IFN score.

**Objectives:** In this study, we aim to investigate the type I IFN score in peripheral blood of patients affected by paediatric rheumatic diseases characterized by the presence of systemic inflammation.

**Methods:** We evaluated the expression of type I interferon-stimulated genes (ISGs) (IFI27, IFI44L, IFIT1, ISG15, RSAD2, SIGLEC1), by real time PCR in patients with systemic lupus erythematosus (SLE) (n = 14), juvenile dermatomyositis (JDM) (n = 7), systemic juvenile idiopathic arthritis (sJIA) (n = 2), chronic recurrent multifocal osteomyelitis (CRMO) (n = 3), adenosine deaminase deficiency (DADA2) (n = 3), CANDLE syndrome (n = 1), NLRC4-related disease (n = 1), familial mediterranean fever (FMF) (n = 2), undefined autoinflammatory diseases (UAD) (n = 11) and in healthy donors (HDs) (n = 4). The median fold change of the ISGs, when compared with the median of the combined HD, was used to create the type I IFN score. The mean interferon score of the controls plus two SD above the mean was calculated. Scores higher than this value (1,61) were defined as positive.

**Results:** We found that a significant up-regulation of the type I IFN score characterized the peripheral blood of SLE, DADA2 and UAD patients compared to HDs (*p* < 0.05). In SLE patients the type I IFN score was significantly related to the disease activity as measured by the SLEDAI score (*p* < 0.01). In JDM, 2 patients with active disease had high type I IFN score. As expected, the CANDLE patient had elevated type I IFN score. We analysed the expression levels of the ISGs in other rheumatic disorders such as sJIA, CRMO, FMF but did not find significant differences with HDs. Interestingly, type I IFN score was elevated in the NLCR4 patient. Some of the UAD patients showed a strong up-regulation of the type I IFN score and clinical manifestations in common with the typical IFNpathies including variable combination of cerebral calcifications, lipodystrophy, interstitial lung disease, cerebral stroke, recurrent fever, low levels of C3 and mild positivity of antinuclear antibodies. Also variable expression of the ISGs was observed: in 2 patients a fold change of IFI27 of 937 and 530 compared to controls, while the expression of the other 5 genes was much lower. We found that one UAD patient with a high type I IFN score carried a *de novo* undescribed gain of function mutation in the STAT1 gene.

**Conclusion:** Up-regulation of type I IFN score characterized different systemic inflammatory disorders, in addition to classical type I IFNpathies. In the group of UAD we found patients with high expression of the ISGs and characterized by clinical manifestations in common with the typical interferonopathies.

**Disclosure of Interest**

None Declared

**Table 41 (abstract P364).** See text for description

Rheumatic disease	Median of type I IFN score (IQR)	Percentage of positive patients
SLE (n = 14)	8.8 (1.9-14.7)	78.5
JDM (n = 7)	0.7 (0.3-9.5)	28.5
sJIA (n = 2)	0.12 (0.11-0.12)	0
CRMO (n = 3)	0.5 (0.2-3.5)	33.3
DADA2 (n = 3)	3.9 (3.6-10.4)	100
CANDLE (n = 1)	4.9	
NLRC4 (n = 1)	2.5	
FMF (n = 2)	0.5 (0.32-0.68)	0
UAD (n = 11)	2.3 (1.4-5.5)	81.8

### P365 Comparison analysis of non-bacterial and tuberculous osteomyelitis

#### Olga Kopchak^1,2^, Mikhail Kostik^1^, Alexander Mushkin^3^, Alexey Maletin^3^

##### ^1^Hospital Pediatry, Saint Petersburg State Pediatric Medical University, Saint Petersburg, Russian Federation; ^2^Pediatry, Kirov’s regional children’s hospital, Kirov, Russian Federation; ^3^Pediatric Surgery, FGBU Saint-Petersburg Science-Research Institute of Phthisiopulmonology Ministry of public health, Saint Petersburg, Russian Federation

###### **Presenting author:** Olga Kopchak

**Introduction:** Non-bacterial and tuberculous osteomyelitis (NBO and TBO, relatively) belong to the group of primary chronic osteomyelitis, requiring an accurate differentiation for choosing the adequate treatment strategy. The clinical signs and the radiology features of bone lesions are similar in these diseases. In the countries where BCG vaccination is mandatory the tuberculin skin tests (TST) sensitivity is currently considered as quite low for the diagnosis of specific bone lesions.The aim of our study: to compare clinical and laboratory features, which could discriminate the NBO and TBO.

**Objectives:** The study includes data of 95 children (52 - with NBO and 43 - with TBO) aged less than 18 years old with focal bone destructions, identified on the basis of complex radiological methods – X-ray, CT, MRI and bone scan

**Methods:** All patients underwent closed or open bone biopsy. Patients with positive bone culture differ from *M. tuberculosis* (MBT) were excluded from present study. The diagnosis of TBO based on the positive bacteriological tests incl. molecular-genetic test (*M. tuberculosis complex* have been identified in 26/43 cases), and/or the typical morphological signs of specific granulomatosis and necrotic inflammation. The NBO diagnosis was made upon detection of non-specific inflammation morphology and negative bone bacteriological tests. We evaluated the number of bone lesions, the diagnostic delay and laboratory data – hemoglobin level, WBC, platelets, monocytes, ESR and CRP level.

**Results:** The main demographic data and the results of comparison between NBO and TBO are in the Table 44. Where were no differences in gender distribution between NBO patients, while among the TBO patients boys were affected more frequently. We have found differences between NBO and TBO in onset age - 8.4 (5.4; 11.0) and 2.2 years (1.3; 3.2) (p = 0.0000001) accordingly and in diagnostic delay - 6.3 (2.0; 17.8) and 2.9 months (1.7; 7.2) (p = 0.06). There were no any significant differences in quantity of leukocyte, platelets, hemoglobin level, ESR and CRP, exclude the monocytes. Multifocal lesion and presence of concomitant arthritis was more typical for NBO rather than TBO. We have found the differences in frequency of axial and peripheral bone lesions between patients with NBO and TBO regarding the spine, tibia, fibula, foot bones, pelvis and clavicle.

**Conclusion:** The revealed differences which can optimize the differential diagnosis of NBO and TBO, which might reduce the diagnostic delay and encourage the adequate treatment strategy.

**Disclosure of Interest**

None Declared

### P366 Symptomatic treatment of inflammation in acid ceramidase deficiency (Farber disease) with biologics

#### Balahan Makay^1^, Ezgi D. Batu^2^, Boris Hügle^3^, Nur Arslan^4^, Alexander Solyom^5^, John Mitchell^6^, Edward Schuchman^7^, Seza Ozen^2^, Bo Magnusson^8^

##### ^1^Pediatric Rheumatology, Dokuz Eylul University Hospital, Izmir, Turkey; ^2^Pediatric Rheumatology, Hacettepe University Hospital, Ankara, Turkey; ^3^German Center for Pediatric Rheumatology, Garmisch-Partenkirchen, Germany; ^4^Pediatric Metabolism and Nutrition, Dokuz Eylul University Hospital, Izmir, Turkey; ^5^Clinical Research, Plexcera Therapeutics LLC, New York, USA; ^6^Pediatric Endocrinology, Montreal Children's Hospital, Montreal, Canada; ^7^Genetics and Genomic Sciences, Icahn School of Medicine at Mt. Sinai, New York, USA; ^8^Pediatric Rheumatology, Karolinska University Hospital, Stockholm, Sweden

###### **Presenting author:** Balahan Makay

**Introduction:** Farber disease (Farber lipogranulomatosis; acid ceramidase deficiency) is a rare, autosomal recessively inherited lysosomal storage disorder, resulting from a mutation in the *ASAH1* gene causing a deficiency of acid ceramidase and the accumulation of its pro-inflammatory and pro-apoptotic lipid substrate, ceramide. Many patients show pronounced signs of inflammation: arthritis/synovitis on physical examination and with imaging, pain, elevated C-reactive protein (CRP) and erythrocyte sedimentation rate (ESR), and even recurring fever. The clinical similarity between moderate Farber phenotypes and polyarticular juvenile idiopathic arthritis (JIA) suggests that Farber disease is likely to be misdiagnosed as JIA. A partial response to anti-inflammatory treatment in Farber patients may also contribute to misdiagnosis. Differential diagnosis can be assisted in most cases by the presence of comparatively early-onset, progressive symmetric arthritis, even a small number of subcutaneous nodules, and an unusual, hoarse cry or voice (due to nodule formation on the larynx) in Farber patients. Currently, Farber disease can be treated with hematopoietic stem cell transplantation, which has shown variable results and carries a severe burden for the patients. Enzyme replacement therapy is currently being developed for Farber disease.

**Objectives:** Here we report the clinical response of 5 patients with biochemically and genetically confirmed Farber disease to treatment with an interleukin 6 receptor inhibitor.

**Methods:** Data was gathered retrospectively by chart review. The patients vary in age from infancy to 10 years.

**Results:** All patients demonstrated some clinical response to interleukin-6 receptor (IL-6R) inhibition measureable by changes in standard inflammatory markers (e.g., ESR), and/or improvement of symptoms such as pain, physical impairment, feeding ability and general medical condition. The degree to which each patient responded was variable, in large part due to the difference in disease severity, and in no case has the disease ceased to progress. One patient, severely affected from infancy including CNS involvement, died in spite of treatment.

**Conclusion:** The information gathered from this cohort, along with the knowledge of the pro-inflammatory role of ceramide, indicates that Farber disease patients who are severely affected or show signs of arthritis, pain, or progressive physical impairment can benefit from anti-inflammatory therapy (preferably IL-6R inhibition), even though it cannot halt the progression of the disease or resolve disease symptoms. Farber disease should be included in the differential diagnosis of JIA, especially in patients with early-onset polyarticular subtype or inadequate response to conventional therapies.

**Disclosure of Interest**

B. Makay: None Declared, E. Batu: None Declared, B. Hügle: None Declared, N. Arslan: None Declared, A. Solyom Employee of: Plexcera Therapeutics LLC, J. Mitchell: None Declared, E. Schuchman Shareholder of: Plexcera Therapeutics LLC, S. Ozen: None Declared, B. Magnusson: None Declared

### P367 Multiple auto immune syndrome (MAIS) in Colombian juvenile patients

#### Clara Malagon^1^, Pilar Gomez^2^, Catalina Mosquera^1^, Tatiana Gonzalez^3^, Ricardo Yepez^2^, Camilo Vargas^4^ and GRIP study group

##### ^1^Pediatric Rheumatology, El Bosque University, Bogota, Colombia; ^2^Pediatric Rheumatology, Universidad Libre, Cali, Colombia; ^3^Pediatric Rheumatology, Universidad de Cartagena, Cartagena; ^4^Pediatric Rheumatology, Universidad del Valle, Cali, Colombia

###### **Presenting author:** Clara Malagon

**Introduction:** Multiple auto immune syndrome (MAiS) is defined as the presence of three or more autoimmune diseases in a single patient. Similar genetic, epigenetic factors, and disregulation of immune response may explain the development of multiple AIDs in a host

**Objectives:** Members of the GRIP study group organized a registry of juvenile patients who developed polyautoimmunity.The demographic and clinical features y patients with MAiS were determined.

**Methods:** One year ago, GRIP study group developed a registry of patients followed at 10 pediatric rheuma-tology clinics in 4 Colombian cities who developed polyautoimmunity. The clinical charts of MAiS patients were reviewed and the demographic, clinical, serological and histopathological (when was available) data were collected in an electronic database. The data was analysed using a SSPS 15 version program

**Results:** N = 46 . The sex ratio was female 14.3: male 1 with a mean age of onset of 11 years (3- 16 y). 34% had a positive history of familial autoimmunity. Sequential development of 3 AiDs was observed on 72% and 50% on patients with 4 diseases and all patients developed AiDs on the first two decades of life. 22 AiDs were identified in different associations.The most common systemic AiD were: SLE, Hashimoto, APLS and autoimmune citopenias. Hashimoto was the most frequent organ specific disease and other endocrinopaties as diabetes mellitus type 1, Graves disease and polyglandular autoimmune endocrinopathies were also identified. Sjogren syndrome was present as the second, third or fourth autoimmune disease after a variable period of time.The most common heralding diseases were: SLE, Hashimoto, APLS, autoimmune citopenias and JIA but all of them were also present as second or third disease developed by those patients. Sclerodermia, vitiligo, psoriasis were dermatologic diseases associated to multiple auto immune diseases. 2/4 patients developed Sjogren syndrome as the 4th AiDs and the other 2 patients with MAiS associated Hashimoto or ALPS as the fourth autoimmune disease.

**Conclusion:** New symptoms, changes of functional test of target organs or changes of autoantibody profile may alert about associations of three or more autoimmune diseases including an a early age on life with a variable intervals of time.A definitive predominance of female was observed and a positive family history was also present on this group of patients. A long clinical follow up and a high index of suspiction is needed to identify those patients.

**Disclosure of Interest**

None Declared

### P368 Sleep and restless leg syndrome in adolescents with idiopathic musculoskeletal pain

#### Rita A. Amorim^1^, Claudio A. Len^1^, Juliana Molina^1^, Gustavo Moreira^2^, Flávia H. Santos^3^, Melissa Fraga^1^, Livia Keppeke^1^, Vanessa M. Silva^1^, Camila Hirotsu^2^, Sergio Tufik^2^, Maria Teresa Terreri^1^

##### ^1^Pediatrics, Universidade Federal de São Paulo, São Paulo, Brazil; ^2^Sleep Institute, Universidade Federal de São Paulo, São Paulo, Brazil; ^3^University of Minho, Braga, Portugal

###### **Presenting author:** Claudio A. Len

**Introduction:** Idiopathic musculoskeletal pain (IMP) is related to a negative impact in the quality of life of children and adolescents sleep problems and psychosocial factors seem to be involved in its pathogenesis, which is not fully known. Restless legs syndrome (RLS), periodic limb movements (PML) and sleep problems were observed in adults with chronic musculoskeletal pain. However, the literature related to these problems in children and adolescents is scarce and there are no studies on the prevalence of RLS and PML in adolescents with IMP.

**Objectives:** To evaluate the presence of RLS, PML and the sleep disorders in female adolescents with IMP according a specific questionnaire and polysomnography, and to compare these data with healthy adolescents without pain.

**Methods:** We evaluated 26 IMP female adolescents followed up in a pain clinic of a tertiary hospital and 25 healthy controls matched for age and school level. The study protocol included questionnaires about demographic and socioeconomic aspects, evaluation of the criteria for SPI according to the International Restless Legs Syndrome Study Group, assessment of sleep quality through the questionnaire Sleep Disturbance Scale for Children (SDSC) and adolescents and polysomnography.

**Results:** The average age of patients with IMP was 13.9 ± 1.6 years and the average age of controls was 14.4 ± 1.4 years. A patient in the control group (4%) and 9 patients with IMP (34.6%) met the criteria for RLS (p = 0.011). We did not observe history of RLS in family members of both groups. Regarding the SDSC, we did not observe statistically significant differences between both groups with respect to all components: initiation and maintenance (p = 0.290), respiratory (p = 0.576), wake up disorders (p = 0.162), sleep/wake transition (p = 0.258), excessive daytime sleepiness (p = 0.594) and hyperhidrosis (p = 0.797). In polysomnography, the neurophysiological and respiratory parameters and the number of PLM were similar in both groups. Three patients with IMP (11.5%) presented RLS and periodic limb movements simultaneously, which was not observed in the control group.

**Conclusion:** In our study, adolescents with pain presented criteria for RLS more frequently when compared with adolescents without pain complaints. However, we did not observe abnormalities in sleep quality these patients according questionnaires and polysomnography.

**Disclosure of Interest**

None Declared

### P369 Experience of a pediatric musculoskeletal pain clinic: multidisciplinary assessment, referral and treatment adherence

#### Vinícius L. Braga, Maria Beatriz Fonseca, Claudio A. Len, Melissa Fraga, Vania Schinzel, Maria Teresa R. Terreri

##### Pediatrics, Universidade Federal de São Paulo, São Paulo, Brazil

###### **Presenting author:** Claudio A. Len

**Introduction:** The treatment of children and adolescents with idiopathic musculoskeletal pain is complex and requires the participation of a multidisciplinary team composed of physicians, physical therapists, psychologists, nutritionists, social workers, among others. In our pain clinic patients are individually assessed by all professionals, who meet to design a treatment plan. With this, there are several internal referrals, as well as recommendations on changes in lifestyle (sleep pattern and physical activities). However, the adherence to treatment is still poor in many cases.

**Objectives:** To evaluate treatment aspects of patients followed in a pediatric pain clinic: referrals to professionals and adherence, as well as the results of this holistic model focused on the patient and the caregivers.

**Methods:** Data from medical records of patients referred and followed in the pain clinic were entered into a spreadsheet. Only patients consulted by doctors on two or more occasions were included. All patients are referred for evaluation by professionals of the multidisciplinary team, which determines the flowchart to facilitate the longitudinal follow-up. For outcome assessment we used a visual analog scale of pain. To evaluate adherence to treatment, the rate of presence was assessed in the consultations of different therapeutic modalities.

**Results:** 263 patients were referred for musculoskeletal pain clinic. Of these, 99 patients (37.6%) were not consulted or had only a medical consult. Among those not included, 44.9% were referred and not even really make the first appointment. Of the 164 patients included, 73.8% were female. The average age in the first consult was 10.6 years, ranging from 3 to 17 years, and there was a predominance of adolescents. Once forwarded to the multidisciplinary assessment, 96.0% attended a consult with the social worker, 83.3% consult with nutritionist, 71.7% with the physical therapist (without Rolfing) and 65.3% to psychological consult. Of the 49 patients referred for physical therapy with Rolfing, 89.9% were consulted. Of the 43 patients referred for dental assessment to evaluate temporomandibular joint, 82.6% attended the consultation. The patients were present in 100% of scheduled consultations with the social worker, 89.6% of the consults with the pediatric rheumatologist, 89.6% with physical therapists, 83.6% with the odontologists, 83.4% with physiotherapy (Rolfing) and 78.5% of the scheduled consultations with psychologists. The nutrition team had the highest percentage of absences and only 73.3% of patients were present to the appointments. The number of consults on average per patient in psychology, physical therapy with Rolfing, physical therapy without Rolfing, nutritionist, odontologist and social worker were, respectively, 9.15; 7.98; 7.27; 6.34; 5.67 and 3.26 consults per patient.Pediatric rheumatologists attended each patient, on average, 9 times. A patient at the pain clinic had, on average, 26.5 consults during the multidisciplinary follow up. Concerning the pain score from the point of view of the patients, the average score on the visual analogue scale of first consultation was from 5.4, to a decrease to 4.0 in the last.

**Conclusion:** Therapeutic success depends on the influence of multiple factors. The present study demonstrates the difficulty of following the treatment, which was highest among pediatric rheumatology follow up. This difficulty is less frequently observed in other therapeutic modalities. We observed a decrease in the average pain score, which indicates the success of the treatment. However, success exists only in those who had good adherence.

**Disclosure of Interest**

None Declared

### P370 Brain activation in adolescents with idiopathic musculoskeletal pain: differences between predictable and unpredictable stimuli

#### Juliana Molina^1^, Claudio A. Len^1^, Liliana Jorge^2^, Liana Guerra^2^, Flávia H. Santos^3^, Maria Teresa Terreri^1^, Edson Amaro Junior^2^

##### ^1^Pediatrics, Universidade Federal de São Paulo, São Paulo, Brazil; ^2^Brain Institute, Hospital Israelita Albert Einstein, São Paulo, Brazil; ^3^University of Minho, Braga, Portugal

###### **Presenting author:** Claudio A. Len

**Introduction:** Idiopathic musculoskeletal pain (IMP) affects approximately 12-35% of children and adolescents in school age. The negative impact of this condition in the quality of life is high, mainly in the physical, emotional, social and scholar aspects. It’s etiopathogeny remains unknown and comprises several hypothesis. Some studies about functional magnetic resonance (fMRI) showed that adult patients with musculoskeletal pain syndromes tolerate a smaller amount inducted pain (produced by pressure stimuli) and exhibit differences in brain activation patterns in pain-related areas. However, there are no studies using fMRI in pediatric patients with IMP.

**Objectives:** The aim of this study was to evaluate, through fMRI techniques, brain activation in adolescents with IMP during an experimental paradigm of pain.

**Methods:** 10 adolescents with IMP followed in a multiprofessional pain clinic in a tertiary hospital and 10 healthy adolescents, aged 14 to 17 years, were included in the protocol. The fMRI tests were performed in 3 T scanner (Magnetom Trio, Siemens) with an event-related design paradigm, factorial and with parameterization of the conditions of each stage/task. Pressoric stimuli were performed in nondominant hand thumb, divided into two parts: fixed predictable and variable unpredictable pain. Data were processed and analyzed by the FSL software.

**Results:** The IMP group showed a reduced pain threshold (IMP = 3.7 kg/cm^2^ versus Healthy adolescents = 4.45 kg/cm^2^, p = 0.005). The intragroup analysis results showed that the IMP group presented greater brain activation during the variable unpredictable pain stimuli paradigm, especially in the lingual gyrus (p = <0.001), frontal lobe and precentral gyrus (p = <0.001e p = 0.03) and temporal gyrus (p = <0.001) when compared to fixed predictable pain stimuli. IMP adolescents showed greater activation during variable unpredictable pain stimuli especially in areas related to processing of emotional information, decision-making, executive functions and areas of somatosensory integration.

**Conclusion:** Based on these results, it is reasonable to assume that several brain areas correlated to cognitive, emotional and behavioral aspects, related to pain perception in comparison were recruited longer than expected in IMP adolescents when compared to the same brain areas from adolescents without chronic pain.

**Disclosure of Interest**

None Declared

### P371 Experience of a pediatric musculoskeletal pain clinic: demographic and clinical data

#### Maria Beatriz Fonseca, Vinícius L. Braga, Claudio A. Len, Melissa Fraga, Vania Schinzel, Maria Teresa R. Terreri

##### Pediatrics, Universidade Federal de São Paulo, São Paulo, Brazil

###### **Presenting author:** Claudio A. Len

**Introduction:** Many children and adolescents are referred to Pediatric Rheumatology clinics for evaluation of musculoskeletal pain without apparent cause. In these cases the pain may be localized or diffuse. The treatment of the patients should be individualized and it generally involves the participation of a multiprofessional trained team, who gives orientation to the patients and their families with regard to and pain coping and control. The number of specialized pain clinics is low, especially in countries with low-income population.

**Objectives:** To present the demographic and clinical features of patients followed in the first specialized musculoskeletal pain clinic of a public university hospital in our country.

**Methods:** Data from medical records of patients who were referred and followed in the pain clinic were included into a spreadsheet. Only patients consulted by doctors on two or more occasions were taken into account. Furthermore, we evaluated the recorded data in the multidisciplinary clinic system.

**Results:** Results: 263 patients were referred for musculoskeletal pain clinic. Of these, 99 patients (37.6%) were not consulted or had only a medical evaluation. In 6 cases there were problems in the filling in of the data and the patients were excluded. Thus, we included 164 patients for the statistical analysis, 121 (73.8%) female and 43 male (26.2%). The duration between onset of symptoms and the first visit in the pain clinic was about 2.4 years. The average age in the first consult was 10.6 years, ranging from 3 to 17 years, and the average follow-up time was 3.1 years. Regarding the diagnosis, 127 patients (77.4%) presented pain amplification syndromes, 34 (20.7) fulfilled the ACR fibromyalgia criteria, 32 (14.0%) presented joint hypermobility, 12 (7.3%) had orthopedic problems, 6 (3.6%) complex regional pain syndrome and 5 (3%) presented temporomandibular joint dysfunction 5. With respect to comorbidities, 4 showed obesity (2.4%) and one hypothyroidism. 39.4% of the patients presented arthralgia, but no patient developed arthritis. 62.8% reported headache, 32.9% abdominal pain and 38.0% sleep problems. The average pain VAS score was 5.4 (0 – 10).

**Conclusion:** As in the literature, most patients were female and there was a delay in the appointment of the first consult in the pain clinic. Headache was the most common symptom in addition to musculoskeletal pain.

**Disclosure of Interest**

None Declared

### P372 New diagnostic criteria of acute rheumatic fever: prevalence of silent carditis in a pediatric population

#### Clotilde Alizzi, Maria Cristina Maggio, Maria Cristina Castiglione, Alessandra Tricarico, Giovanni Corsello

##### Azienda Ospedaliera di Rilievo Nazionale e di Alta specializzazione "Civico-G.Di Cristina-Benfratelli", Palermo, Italy

###### **Presenting author:** Clotilde Alizzi

**Introduction:** Acute rheumatic fever and its sequel, chronic rheumatic heart disease, are important global health issues with an annual incidence of about 500.000 new cases and a prevalence of 34 million people worldwide affected by rheumatic heart disease. During the 20th century the incidence of ARF and the prevalence declined substantially in Europe, North America, and developed nations in other geographic locations. In Italy the incidence is about 4,1:100.000. Acute rheumatic fever is a systemic inflammatory response to group A streptococcal infection, which typically affects children and occurs two or three weeks after a throat infection. Although arthritis is the most common sign, carditis which commonly affects the mitral and aortic valves, is the most specific and severe one, for the eventual risk of chronic rheumatic cardiopathy. Other less common clinical features include chorea, rash (erythema marginatum), and subcutaneous nodules. Diagnosis requires demonstration of the presence of major and minor criteria and laboratory evidence of a recent streptococcal throat infection. In the 1992 AHA revised Jones criteria statement, the diagnosis of carditis was clinical, based on the auscultation of typical murmurs that indicate mitral or aortic valve regurgitation. The Australian and New Zealand Diagnostic Criteria, published on Circulation in 2015, extend the 1992 Jones criteria for acute rheumatic fever by including echocardiographic evidence of silent carditis and a wider spectrum of joint manifestations as major criteria. Subclinical carditis is characterised by the absence of classic auscultatory findings of valvular dysfunction and the by the echocardiographic evidence of mitral or aortic valvulitis.

**Objectives:** The aim of our study was to define the prevalence of silent carditis in a casistic of pediatric patients affected by Acute Rheumatic fever followed from 2012 to 2016.

**Methods:** The study included 18 patients who were diagnosed with ARF according to the Jones criteria. The patients with echocardiographic findings of carditis and no clinical signs of cardiopathy, were diagnosed as having SC. Silent carditis was defined as the presence of the following findings on Doppler echocardiography: isolated mild mitral or aortic regurgitant jets. The patients diagnosed with subclinical carditis were followed up and re-evaluated at 3, 6, and 12 months.

**Results:** Acute rheumatic fever was diagnosed in 18 patients, and 7 of these (40%) had silent carditis (two patients also had chorea). All patients who underwent diagnosis of SC had C-Reactive protein values greater than upper limit of normal for laboratory. Our patients had an average age, at the time of diagnosis, of 7,4 years old. The echocardiography/Doppler findings were: isolated mild mitral regurgitation in 6 patients and isolated mild aortic regurgitant jets in one patient. We observed a complete recovery in two patients with mitralic insufficiency and in the case of aortic regurgitation. All patients receive prophylaxis therapy with benzilpenicillin each 21 days.

**Conclusion:** The high prevalence (42%) of silent carditis in our casistic emphasize the role of Doppler echocardiography in patients who undergo suspected diagnosis of acute rheumatic fever, for an early detection of silent carditis. Patients affering to a third-level reference center could have a more severe disease causing an eventual selection bias in collecting our data. We also observed an high prevalence of recovery (42%) from silent carditis, during the follow up.

**Disclosure of Interest**

None Declared

### P373 Children with cystic fibrosis and musculoskeletal complaints seen in the rheumatology clinic: a single centre experience

#### Emily Boulter^1^, Andre Schultz^2,3,4^, Kevin Murray^1^

##### ^1^Rheumatology and Metabolic Medicine, Perth, Australia; ^2^Respiratory Medicine, Princess Margaret Hospital, Perth, Australia; ^3^Telethon Kids Institute, Perth, Australia; ^4^School of Paediatric and Child Health, University of Western Australia, Perth, Australia

###### **Presenting author:** Emily Boulter

**Introduction:** Musculoskeletal and rheumatic manifestations of cystic fibrosis (CF) are well described, including cystic fibrosis-related arthropathy (CFA), hypertrophic pulmonary osteoarthropathy (HPOA), antibiotic associated arthralgia (particularly ciprofloxacin-related arthropathy), osteopaenia and osteoporosis, as well as coincidental incidence of rheumatic conditions such as juvenile idiopathic arthritis and biomechanical issues such as postural abnormalities.(1) The prevalence of musculoskeletal symptoms in CF patients increases with age and occurs more frequently in adults, however children may also suffer from musculoskeletal complications of CF.(1)

**Objectives:** To describe the cohort of children with cystic fibrosis referred to the rheumatology clinic at our tertiary paediatric centre with musculoskeletal symptoms over an eleven-year period.

**Methods:** A search of the rheumatology department electronic clinic letter database was performed using the search terms ‘cystic fibrosis’ and ‘CF’ for an 11 year period from January 2004 to April 2016. Identified cases were then reviewed using a combination of clinic letters and clinical notes from the medical records.

**Results:** 26 patients were identified; 54% were female (n = 14) with a median age of 11 years (range 3-17 years) at first referral to Rheumatology. The median duration of musculoskeletal symptoms prior to assessment in the rheumatology clinic was 6.5 months (range 0.5-24 months). The commonest presenting symptoms were joint pain 81% (n = 21), joint swelling 27% (n = 7), limp or non-weight bearing 15% (n = 4) and back pain 15% (n = 4). The commonest diagnoses were non-specific musculoskeletal pain (e.g. Associated with hypermobility, mechanical or postural issues) 42% (n = 11), CFA 31% (n = 8) and reactive arthritis 12% (n = 3). Two patients had a diagnosis of possible juvenile idiopathic arthritis, with CFA as the differential diagnosis. Diagnoses were considered to be directly (e.g. CFA) or indirectly (e.g. reactive arthritis, ciprofloxacin-related arthropathy) related to the patient’s CF diagnosis in 46% (n = 12) of cases. Other diagnoses were Perthe’s disease, growing pains, patellofemoral dysfunction, complex regional pain syndrome, avascular necrosis of the hip, congenital elbow dislocation and Osgood-Schlatter disease. Five children had more than one musculoskeletal diagnosis. No patients were diagnosed with HPOA.

**Conclusion:** CFA was the second commonest diagnosis among our cohort; many patients in our cohort had musculoskeletal symptoms unrelated to CF. As musculoskeletal complaints in children and adolescents are a common cause for presentation to medical services (2), it is unsurprising that children with CF suffer from musculoskeletal issues seen in the general population, as well as those associated with CF. No patients in our cohort were diagnosed with HPOA. As the median age of onset of HPOA is 20 years(3), the lack of HPOA cases may reflect the improved respiratory health of the patient cohort compared to historical cohorts where HPOA was commonly reported.

**References**

1. Koch AK, Bromme S, Wollschlager B, Horneff G, Keyszer G. Musculoskeletal manifestations and rheumatic symptoms in patients with cystic fibrosis (CF) no observations of CF-specific arthropathy. J Rheumatol. 2008 Sep;35(9):1882-91.

2. De Inocencio J. Epidemiology of musculoskeletal pain in primary care. Arch Dis Child. 2004 May;89(5):431-4.

3. Quon BS, Aitken ML. Cystic fibrosis: what to expect now in the early adult years. Paediatr Respir Rev. 2012 Dec;13(4):206-14.

**Disclosure of Interest**

None Declared

### P374 Vitamin D levels in a large cohort of patients with pediatric autoimmune neuropsychiatric disorders associated with streptococcal infections (PANDAS): a possible role in neurological disorders?

#### Fernanda Falcini^1^, Gemma Lepri^1^, Stefano Stagi^2^, Eleonora Bellucci^1^, Marco Matucci-Cerinic^1^

##### ^1^Section of Rheumatology, ^2^Health Sciences Department, Anna Meyer Children’s University Hospital, University of Florence, Florence, Italy

###### **Presenting author:** Fernanda Falcini

**Introduction:** Vitamin D (vitD) is known for its essential role in calcium homeostasis and bone health. It is now considered as a potent neurosteroid hormone, with a pivotal role on the brain development and normal brain function. VitD ligand-receptor, a receptor that mediates the majority of vitamin D biological actions, has been found throughout the body including the central nervous system (CNS). Vitamin D deficiency is commonly observed in patients with severe mental illness such as schizophrenia. PANDAS is an autoimmune disorder characterized by obsessive-compulsive symptoms and/or tics triggered by group-A beta-hemolytic Streptococcus infections with relapsing/remitting course due to recurrence of infections itself. Despite the action of 25-hydroxyvitamin D [25(OH)D] on immune and CNS system, to our knowledge, no studies have been performed in patients with PANDAS.

**Objectives:** To evaluate 25(OH)D levels in a large cohort of children and adolescents with PANDAS 2. To compare the results with a healthy control group.

**Methods:** We measured plasma 25(OH)D levels in one hundred seventy-nine patients with PANDAS (49 females, 132 males, mean age at diagnosis 101.4 ± 30.1 months) and an age, sex, and body mass index-matched control group of 179 subjects.

**Results:** Patients with PANDAS showed a higher percentage of reduced 25(OH)-vitamin D levels (<30 ng/mL) in comparison with controls (94.6% *vs.* 82.5%; p < 0.005). In addition, PANDAS patients have lower levels of 25(OH)D than controls (20.4 ± 6.9 *vs.* 24.8 ± 7.3 ng/mL, p < 0.0001). This difference has been confirmed also considering the winter (13.7 ± 3.25 vs. 21.4 ± 5.9) and summer (21.8 ± 6.5 *vs.* 32.5 ± 8.7) period. Yet, serum 25(OH)-vitamin D levels correlated with the number of streptococcal infections before the diagnosis (p < 0.005) and with their recurrences (p < 0.005).

**Conclusion:** PANDAS patients have reduced 25(OH)D values that seem to be associated with streptococcal infections, and the probability of its recurrences. So, reduced 25(OH)D levels may be a cause significantly important in PANDAS etiopathogenesis. A regular supplementation of VitD in PANDAS patients is recommended as it should improve the course of the disease and prevent or reduce the recurrences of infections and the relapses of neurological symptoms. This hypothesis should be further explored in future studies.

**Disclosure of Interest**

None Declared

### P375 Collaboration across the atlantic – a cultural clash or opportunity?

#### Ingrid H. R. Grein^1,2^, Noortje Groot^2,3^, Gecilmara Pileggi^4^, Natália B. F. Pinto^4^, Aline L. de Oliveira^4^, Nico Wulffraat^2^

##### ^1^Pediatric Rheumatology Department, Hospital Pequeno Príncipe, Curitiba, Brazil; ^2^Pediatric Immunology and Rheumatology Department, Wilhemina Children’s Hospital/University Medical Centre Utrecht, Utrecht, Netherlands; ^3^Pediatric Immunology Department, Sophia Children's Hospital - Erasmus MC, Rotterdam, Netherlands; ^4^Pediatric Immunology and Rheumatology Department, Ribeirão Preto Medical School - University of São Paulo, São Paulo, Brazil

###### **Presenting author:** Ingrid H. R. Grein

**Introduction:** International collaboration is extremely valuable for medical care and science, especially when studying rare diseases. Collaborative projects across developed countries are ongoing. But why stop the international collaborations at high developed countries borders? There is a huge potential for scientific collaboration with developing countries as well.

**Objectives:** To show two examples of such ongoing collaborations, thus highlighting the potential of collaboration between developed and developing countries.

**Methods:** Nowadays, distance and language are barriers that can be overcome thanks to technology and existing societies such as PRES. Other challenges remain, so collaborating partners must be conscious of their cultural, economic and legal differences. Sharing new ideas, knowledge and study protocols, as well as teaching and learning new techniques are important in these projects. Fellowship programs aimed at teaching care and research are excellent stepping stones to initiate a high quality long-lasting collaboration. The two following projects between The Netherlands and Brazil were born after a fellowship program.

**Results:** The inclusion of a sufficient amount of patients to study of immunogenicity and safety of Human Papilloma Virus (HPV) and Varicella vaccines in childhood-systemic lupus erithematosus (cSLE) and juvenile dermatomyositis (JDM) is a challenge. Both diseases are rare, and the rates of HPV vaccination are still low in Europe. These difficulties were reflected by the numbers of inclusion for the HPV project, even after United Kingdom and Slovenia were involved. However, when 15 Brazilian centers adopted the study protocol, they had included 304 patients (260 jSLE, 44 JDM) within a year’s time. The data and samples collected in Brazil are shipped to the Netherlands, where analysis will take place. A previous project, initiated in Brazil, involved varicella vaccination in patients with pediatric rheumatic diseases. Over a period of 5 years, 54 patients (42 Juvenile Idiopathic Arthritis (JIA), 5 juvenile Systemic Scleroderma (jSScl) and 7 JDM) had been included. The samples were shipped to the Netherlands, where the cellular responses after varicella vaccination were analyzed.

**Conclusion:** The collaboration between The Netherlands and Brazil has resulted in two fruitful projects involving many patients. Sharing study protocols and data, good communication, as well as training scientists in laboratory techniques have shown to be essential in its success. The results of this collaboration may be extremely beneficial for the science, as well as for the participating countries.

**Disclosure of Interest**

None Declared

**Table 42 (abstract P375).** See text for description

Brazilian collaborating Centers (Brazilian State: Center's Name)	
**Bahia:** Hospital Universitário Prof. Edgard Santos	**São Paulo (SP):** Faculdade de Medicina de Ribeirão Preto
**Brasília:** Hospital da Criança de Brasília	Faculdade de Medicina de Botucatu
**Ceará:** Hospital Infantil Albert Sabin	Faculdade de Ciências Médicas de Campinas
**Espírito Santo:** Hospital Estadual Infantil	Instituto da Criança da Faculdade de Medicina
**Paraná:** Hospital Pequeno Príncipe	da Universidade de SP
**Rio de Janeiro (RJ):** Universidade Estadual do RJ	Universidade Federal de SP
Universidade Federal do RJ	Faculdade de Medicina da Santa Casa de SP
Instituto de Puericultura e Pediatria	Hospital Infantil Darcy Vargas
Martagão Gesteira	

### P376 Gastrointestinal diseases in children with rheumatic diseases

#### Iryna Chyzheuskaya^1,2^, Lyudmila Belyaeva^1^, Rostislav Filonovich^2^, Helena Khrustaleva^1^, Larisa Zajtseva^2^

##### ^1^Belarusian Medical Academy of Postgraduate Education, Minsk, Belarus; ^2^4th City Children's Clinical Hospital of Minsk, Minsk, Belarus

###### **Presenting author:** Iryna Chyzheuskaya

**Introduction:** Etiological factors in the development of inflammatory diseases of the upper with rheumatic diseases of the gastrointestinal tract in children may include: the disease itself, treatment with nonsteroidal anti-inflammatory drugs, corticosteroids and cytotoxic drugs, as well as infection with Helicobacter pylori.

**Objectives:** The purpose of research was to assess the condition of the upper gastrointestinal tract in children with rheumatic diseases.

**Methods:** The rheumatology department of 4th city clinical hospital of Minsk were examined 167 patients with rheumatic diseases, including 54 boys and 113 girls. In the I group included 115 children with juvenile idiopathic arthritis (JIA) (mean age 11,9 ± 3,4 years), II group consisted of 34 children with juvenile scleroderma (JS) (mean age 12,4 ± 2,8 years), III group consisted of 18 children with systemic lupus erythematosus (SLE) (mean age 13,1 ± 1,7 years).

All children performed endoscopic examination of the upper gastrointestinal tract with excisional biopsy of mucous gastric antrum for morphological verification of the diagnosis.

**Results:** The majority of patients with rheumatic diseases without complaints from the gastrointestinal tract.

Pathology of the esophagus, stomach, duodenum was detected in 80% (92) children with JIA, at 88.2% (30) children with JS and 72.2% (13) with SLE. The most commonly in children with JIA and SLE observed inflammatory changes in the gastric mucosa and 12 duodenal ulcer (superficial gastroduodenitis in the acute stage), children with JS –inflammatory changes in the esophageal mucosa.

All patients who have been identified comorbidity gastroduodenal zone, as the main pathogenetic therapy received nonsteroidal anti-inflammatory drugs, cytotoxic agents and steroids, either as monotherapy or in combined treatment. For the treatment of gastroenterological diseases used proton pump blockers, prokinetics and symptomatic therapy.

**Conclusion:** In our study, the majority of children of the three subgroups (JIA, JS and SLE) was diagnosed comorbid condition as chronic gastroduodenitis.

**Disclosure of Interest**

None Declared

### P377 Inflammatory arthritis and recessive multiple epiphyseal dysplasia in a same patient: case report

#### Jaanika Ilisson^1^, Chris Pruunsild^1,2^

##### ^1^Children's Clinic, Tartu University Hospital, Tartu, Estonia; ^2^Children’s Clinic, Institute of Clinical Medicine, Tartu University, Tartu, Estonia

###### **Presenting author:** Jaanika Ilisson

**Introduction:** Multiple epiphyseal dysplasia (MED) is a genetically and phenotypically heterogeneous disease resulting in disorganized epiphyseal ossification of the long bones. So far six different subtypes have been described, among them only one with recessive inheritance (1, 2). All subjects are at risk of developing early osteoarthritis (OA) but inflammatory arthritis has also been described in patients with MED (3). Only four cases of recessive MED (rMED) with homozygosity for the mutation c.1957 T > A p.Cys653Ser in SLC26A gene have been described (4, 5).

**Objectives:** To describe a case of inflammatory arthritis in a nine year old girl with rMED

**Methods:** Description of a case according to the data of medical records.

**Results:** The patient was on a regular orthopaedic observation because of congenital hip dysplasia which was diagnosed at the age of three months and was treated with cast immobilisation until the age of 1 year and 7 months. First episode of arthritis (right knee joint) developed at the age of 8 years with normal inflammatory markers in blood. The episode was managed with nonsteroidal anti-inflammatory drugs (NSAID). Repeated episodes of arthritis with morning stiffness developed during next month affecting left hip, knees, ankles and talonacivular joints. The joint aspirate from left knee also showed inflammation (leukocyte count 6900 x10^6^/L). The effect of NSAID treatment was insufficient and oral methotrexate (MTX) was started 4 months after the first arthritis episode. During the differential diagnosis process x-ray investigations revealed double-laired patella and symmetric flattened epiphyses of long bones which led to the suspicion of MED. The diagnosis was genetically confirmed with the detection of homozygotic mutation in SLC26A gene (c.1957 T > A p.Cys653Ser). After the diagnosis of rMED patient’s brother (age 16 years) was also diagnosed with the same disease showing characteristic radiological changes in long bones but without any serious joint complaints and arthritis episodes. The girl continues treatment with MTX with positive effect and regular physiotherapy.

**Conclusion:** Although OA is the most common form of arthritis in patients with MED, inflammatory arthritis may also develop, needing adequate treatment to prevent long term joint damage.

Written informed consent has been obtained from the parent for the case report.

**References**

1. Unger S, Bonafé L, Superti-Furga A. Multiple epiphyseal dysplasia: clinical and radiographic features, differential diagnosis and molecular basis. Best Pract Res Clin Rheumatol. 2008;22(1):19-32.

2. Anthony S, Munk R, Skakun W, Masini M. Multiple epiphyseal dysplasia. J Am Acad Orthop Surg. 2015;23(3):164-72.

3. Scuccimarri R, Azouz EM, Duffy KN, Fassier F, Duffy CM. Inflammatory arthritis in children with osteochondrodysplasias. Ann Rheum Dis. 2000;59(11):864-9.

4. Mäkitie O, Savarirayan R, Bonafé L, Robertson S, Susic M, Superti-Furga A, et al. Autosomal recessive multiple epiphyseal dysplasia with homozygosity for C653S in the DTDST gene: double-layer patella as a reliable sign. Am J Med Genet A. 2003;122A(3):187-92.

5. Hinrichs T, Superti-Furga A, Scheiderer WD, Bonafé L, Brenner RE, Mattes T. Recessive multiple epiphyseal dysplasia (rMED) with homozygosity for C653S mutation in the DTDST gene--phenotype, molecular diagnosis and surgical treatment of habitual dislocation of multilayered patella: case report. BMC Musculoskelet Disord. 2010;11:110.

**Disclosure of Interest**

None Declared

### P378 Comparison analysis of non-bacterial and acute hematogenous osteomyelitis

#### Mikhail Kostik^1^, Olga Kopchak^1,2^, Alexander Mushkin^3^, Alexey Maletin^3^

##### ^1^Hospital Pediatry, Saint Petersburg State Pediatric Medical University, Saint Petersburg, Russian Federation; ^2^Pediatry, Kirov’s regional children’s hospital, Kirov, Russian Federation; ^3^Pediatric Surgery, FGBU Saint-Petersburg Science-Research Institute of Phthisiopulmonology Ministry of public health, Saint Petersburg, Russian Federation

###### **Presenting author:** Olga Kopchak

**Introduction:** Non-bacterial and acute hematogenous osteomyelitis (NBO and AHO, relatively) are inflammatory diseases of the musculoskeletal system, especially encountered with children and teenagers. In some cases the diseases may have a similar clinical manifestation, therefore require a differential diagnostics, due to fundamentally different plan of approach for treatment.

**Objectives:** To compare clinic-laboratory features of NBO and AHO.

**Methods:** The study includes data of 52 patients with NBO and 47 with AHO under the age of 18 years old. The NBO diagnosis based of clinical finding, laboratory changes, the presence of foci of bone destruction discovered during radiological methods of research, bone biopsy, negative results of bacteriological research of biopsy samples. The AHO diagnosis based in the presence of positive results of bacteriological research of bone biopsy samples.

A comparative analysis was performed on the basis of estimating the number of bone lesions, laboratory data - hemoglobin level, WBC, platelets, ESR, CRP level, identification of infectious agent and the time of diagnosis.

**Results:** The onset age disease of patients is 8,4 (5,4; 11,0) and 11,0 (6,2; 12,9) with NBO and AHO relatively (p = 0,08). NBO took place with equal frequency in both boys and girls, while AHO patients boys were affected - 61,7% (p = 0,17). The time from disease onset to diagnosis was significantly different in the compared groups and was 6,3 (2,0; 17,8) months for patients with NBO and 0,1 (0,03; 0,17) months for patients with AHO (p = 0,0000001). There was not found any difference in the level of hemoglobin and platelet. There was revealed the tendency to the higher level of leukocytes at AHO 12,6 x 10^9^/l(8,4; 15,5), in comparison with NBO, which is - 7,8 x 10^9^/l (6,7; 9,2). There were revealed the differences in ESR - 39,5 (25,0; 58,0) mm/h and 26,0 (11,0; 41,0, p = 0,009), CRP 65,0 mg/l (20,0; 146,0) and 8,2 mg/l (4,3; 34,0, p = 0,01) for patients with AHO and NBO relatively. In a samples of bone biopsy of 29/43(67,4%) patients with AHO was identified the infectious agent, while in cases with NBO 100% of results of bacteriological study of bone punctate were sterile. The disease was accompanied by symptoms of arthritis in 69,2% (NBO) and in 23,4% of cases (AHO), (p = 0,000005). The fever was noted at 43/47 (91,5%) and 20/52 (38,5%) patients with AHO and NBO (p = 0,0000001). Monofocal lesion was met at all the patients with AHO, while with NBO was met only at 10/52 (19,2%) of patients (p = 0,0000001).

**Conclusion:** The analysis of clinic-laboratory features of NBO and AHO was performed. The above mentioned analysis revealed the significant differences between groups regarding disease activity, number of lesions and diagnostic delay.

**Disclosure of Interest**

None Declared

### P379 Clinical relevance of low-titer antinuclear antibodies in children

#### Olivier Gilliaux^1^, Francis Corazza^2^, Christophe Lelubre^3^, Alina Ferster^4^

##### ^1^Department of Pediatrics, Hôpital Civil Marie Curie - CHU de Charleroi, Charleroi, Belgium; ^2^Laboratory of Immunology, CHU Brugmann, Brussels, Belgium; ^3^Department of Internal Medicine, Hôpital Civil Marie Curie - CHU de Charleroi, Charleroi, Belgium; ^4^Department of Pediatric Hematology and Oncology, Hôpital Universitaire des Enfants Reine Fabiola, Université Libre de Bruxelles (ULB), Brussels, Belgium

###### **Presenting author:** Olivier Gilliaux

**Introduction:** Antinuclear antibodies (ANA) are autoantibodies often associated with rheumatological or autoimmune diseases. Numerous studies have tried to determine the clinical relevance of positivity of ANA analysis but this task is difficult. Clinical relevance varies depending on the titer of ANA. Higher it is, more relevant it is. Cut-off is generally set up to 1/160.

**Objectives:** The purpose of this study is to analyze medical records of patients with low-titer ANA (less than 1/320) and to find out what clinical manifestations can be associated with higher clinical relevance.

**Methods:** This is a retrospective study on children tested positive for ANA during the year 2014. Every patient was classified according to the final diagnosis: ANA-associated disease or incidental ANA. For the purpose of this study, we focused on patients with low-titer ANA on their first positive analysis.

**Results:** A total of 1050 ANA results were analyzed. 199 were positive, 19 were excluded. Complete medical datas were reviewed in the remaining 180 patients. Among them,129 had a low-titer ANA on their first test. 105 (81.3%) had ANA-associated disease and 24 (18.7%) had incidental ANA. Clinical relevance of low-titer ANA positivity was higher when prescribed by pediatric endocrinologist, rheumatologist, hematologist or oncologist or when patients presented with more than one arthritis or with morning stiffness. It was lower when cutaneous manifestations were present, when prescribed by physician trainee, by general pediatrician or by pediatric pneumologist, and when prescribed for suspicion of malignancy.

**Conclusion:** Clinical relevance of low-titer ANA is depending on the prescribing physician’s speciality. Some clinical manifestations are more associated with ANA-associated diseases while others are associated with incidental ANA. No relationship was found between biological findings and clinical relevance of ANA analysis.

**Disclosure of Interest**

None Declared

### P380 A web based survey to pediatric rheumatologists from Latin-America: a snapshot reality focused on juvenile idiopathic arthritis patients

#### Raul Gutiérrez Suárez^1^ on behalf of PANLAR Pediatric Rheumatology Study Group, Zoilo Morel^2^, Graciela Espada^3^, Clara Malagon^4^, Claudia Saad-Magalhães C^5^, Luis Lira^6^, Mabel Ladino^7^, Ruth Eraso^8^, Ivonne Arroyo^9^, Flavio Sztajnbok^10^, Clovis Silva^11^, Carlos Rose^12^ and PANLAR Pediatric Rheumatology Study Group

##### ^1^Pediatric Rheumatology, Shriner Hospital For Children Mexico City, Mexico, Mexico; ^2^Hospital de Clínicas. Universidad Nacional de Asunción, Asunción, Paraguay; ^3^Rheumatology Section., Children ´s Hospital Ricardo Gutierrez, Bueno Aires, Argentina; ^4^Pediatric Rheumatology, Universidad El Bosque, Bogotá, Colombia; ^5^Pediatric Rheumatology Division, UNESP, Botucatu Medical School. Brazil., Botucatu, Brazil; ^6^Pediatric Rheumatology, Chilean Society of Rheumatology, Santiago, Chile; ^7^Pediatric Rheumatology, Hospital San Juan de Dios, University of Chile, Santiago, Chile; ^8^Pediatric Rheumatology Division, Hospital Pablo Tobón Uribe, Antioquia University, Antioquia, Colombia; ^9^Pediatric Rheumatology, Hospital San Juan, San Juan, Puerto Rico; ^10^Pediatric Rheumatology Division, Adolescent Health Care Unit, Universida de do Estado do Rio de Janeiro, Rio de Janeiro, Brazil; ^11^Pediatric Rheumatology Unit, Faculdade de Medicina da Universidade de São Paulo (FMUSP), São Paulo, Brazil; ^12^Pediatric Rheumatology Division., Nemours/Alfred I. duPont Hospital for Children, Wilmington, DE, USA

###### **Presenting author:** Raul Gutiérrez Suárez

**Introduction:** Developing countries face serious socio-economical problems, and Pediatric Rheumatic diseases are not seen as priority by health programs.

**Objectives:** To assess Pediatric Rheumatologists (PR's) from Latin America (LA) focused on JIA patients including access to drug therapy and other health services.

**Methods:** A 54 questions web-based survey was conducted in LA PR's. The survey includes 3 principal domains directed to PR's (demographics, practice status and training history) and 2 domains focused on JIA patients (functional status and access to treatment).

**Results:** A total of 169 (47%) of 356 PR's responded the survey from 18 LA countries including: Argentina, Bolivia, Brazil, Chile, Colombia, Cuba, Ecuador, El Salvador, Guatemala, Mexico, Nicaragua, Paraguay, Peru, Puerto Rico, Dominican Republic, Uruguay and Venezuela. There were: 16 countries without PR's, Sixty eight percent of LA PR's works in countries with larger populations and training centers as Argentina, Brazil, Colombia and Mexico. Forty five percent of PR's has been in practice for less than 10 years. Twenty percent of LA children are seen by a Rheumatologist untrained in Pediatrics. Most PR's work in urban areas and under the Public Health System. Ninety percent of the PR's labor time is spent in patient care and only 10% in research. In almost 50% of countries, PR's are solo practitioners.

In regard to JIA patients: ILAR subtypes frequencies matches other caucasian series. The median time from the beginning of first symptoms to the diagnosis was 6 (3-11) months. A Steinbrocker functional class III and IV was observed in 18% of patients at diagnosis. Full adherence to treatment is around 61%; DMARDs requirement: 70%; Biologics use: 40%. Access to biological therapy was graded as “very difficult” in 20% and covered by State or Social Security in 80% of the reported cases.

**Conclusion:** The Survey data suggest a need to promote the access to PR's specialized care in LA countries, increasing the number of PR's and improving the PR's training. Furthermore, to improve earlier referral for a prompt diagnosis, in order to provide adequate and effective treatment preventing prevalent disability in JIA patients from LA countries.

**Disclosure of Interest**

None Declared

